# Partner Bereavement and Risk of Herpes Zoster: Results from Two Population-Based Case-Control Studies in Denmark and the United Kingdom

**DOI:** 10.1093/cid/ciw840

**Published:** 2016-12-15

**Authors:** Sigrun A. J. Schmidt, Mogens Vestergaard, Henrik S. Pedersen, Henrik C. Schønheyder, Sara L. Thomas, Liam Smeeth, Kathryn E. Mansfield, Henrik T. Sørensen, Harriet J. Forbes, Sinéad M. Langan

**Affiliations:** 1 Department of Clinical Epidemiology, Aarhus University Hospital,; 2 Research Unit for General Practice, and; 3 Section for General Practice, Department of Public Health, Aarhus University,; 4 Department of Clinical Microbiology, Aalborg University Hospital, and; 5 Department of Clinical Medicine, Aalborg University, Denmark;; 6 Faculty of Epidemiology and Population Health, London School of Hygiene and Tropical Medicine, United Kingdom

**Keywords:** bereavement, grief, herpes zoster, shingles, psychological stress.

## Abstract

**Background.:**

Psychological stress is commonly thought to increase the risk of herpes zoster by causing immunosuppression. However, epidemiological studies on the topic are sparse and inconsistent. We conducted 2 parallel case-control studies of the association between partner bereavement and risk of zoster using electronic healthcare data covering the entire Danish population and general practices in the UK Clinical Practice Research Datalink.

**Methods.:**

We included patients with a zoster diagnosis from the primary care or hospital-based setting in 1997–2013 in Denmark (n = 190671) and 2000–2013 in the United Kingdom (n = 150207). We matched up to 4 controls to each case patient by age, sex, and general practice (United Kingdom only) using risk-set sampling. The date of diagnosis was the index date for case patients and their controls. We computed adjusted odds ratios with 99% confidence intervals for previous bereavement among case patients versus controls using conditional logistic regression with results from the 2 settings pooled using random-effects meta-analysis.

**Results.:**

Overall, the adjusted odds ratios for the association between partner bereavement and zoster were 1.05 (99% confidence interval, 1.03–1.07) in Denmark and 1.01 (.98–1.05) in the United Kingdom. The pooled estimates were 0.72, 0.90, 1.10, 1.08, 1.02, 1.04, and 1.03 for bereavement within 0–7, 8–14, 15–30, 31–90, 91–365, 366–1095, and >1095 days before the index date, respectively.

**Conclusions.:**

We found no consistent evidence of an increased risk of zoster after partner death. Initial fluctuations in estimates may be explained by delayed healthcare contact due to the loss.

## Summary

Psychological stress is thought to be a risk factor for herpes zoster. However, 2 large population-based studies in Denmark and the United Kingdom found no consistent increase in risk of herpes zoster after partner bereavement.

It is commonly thought that severe psychological stress can provoke reactivation of latent herpesviruses, including the varicella zoster virus, which causes herpes zoster (HZ) [1]. This belief is supported by immunological studies demonstrating activation of the hypothalamic-pituitary-adrenal axis and inhibition of natural killer cell activity, phagocytosis, and cytotoxic T-cell activity in response to stress [1–3]. However, epidemiological data assessing stress as a risk factor for HZ are sparse and inconsistent [4–8].

Five studies have examined the association between negative life events and HZ, with some reporting at least a 40% increase in relative risk up to 4 years after the event [4–7], whereas others report no association [8]. This lack of consistent evidence may be explained by the difficulty of measuring psychological stress, given variation among persons in the types of life events perceived as stressful. Indeed, various measures of stress were employed (eg, health events in partners [8] or the Geriatric Scale of Recent Life Events [5, 6]) in the previous studies.

The death of a loved one is considered extremely stressful [9]. It is likely to affect most persons gravely regardless of coping mechanisms [10], making it a useful model for studying the effects of psychological stress. We therefore examined whether partner bereavement was associated with HZ in 2 parallel case-control studies in Denmark and the United Kingdom.

## METHODS

### Data Sources

Denmark and the United Kingdom have publicly funded healthcare systems [11, 12]. Primary healthcare is delivered by general practitioners, who act as gatekeepers to specialized secondary care provided at hospitals. Prescription drugs are partially or fully reimbursed, although reimbursement schemes differ slightly between the countries.

In Denmark, we used nationwide registries to obtain data on all inpatient and outpatient contacts with nonpsychiatric hospitals (the Danish National Patient Registry [13]) and psychiatric hospitals (the Danish Psychiatric Central Research Registry [14]); prescriptions dispensed at community pharmacies (the Danish National Prescription Registry [15]; patients receiving care for diabetes (the Danish National Diabetes Registry [16]); education (the Population Education Registry [17]); and general demographic data, for example, civil status and vital status (the Civil Registration System [18]).

The main data source for the UK study was the Clinical Practice Research Datalink (CPRD), which contains electronic primary healthcare records for approximately 7% of the UK population [19]. Sixty percent of participating practices allow linkage with hospital inpatient data (the Hospital Episode Statistics database [20]) and individual-level social data (the Index of Multiple Deprivation [21]), which were also used in the present study. Further details about the data sources are provided in Supplementary Appendix 1.

The Danish study was approved by the Danish Data Protection Agency (record number: 2013-41-1719). Danish legislation does not require approval by an ethical review board or informed consent from patients for registry-based studies. The British study was approved by the CPRD Independent Scientific Advisory Committee (record number: 15_248) and the London School of Hygiene and Tropical Medicine Ethics Committee (record number: 11219). Study protocols, including complete code lists, are available as supplementary data.

### Study Population

We included persons with a first-time diagnosis of HZ recorded in general practice or with a primary (first-listed) hospital-based diagnosis of HZ between 1997 and 2013 in Denmark and between 2000 and 2013 in the United Kingdom. Hospital-based diagnoses were available in both settings. However, while general practitioners in the CPRD register reasons for patient contact using Read Codes, diagnoses of HZ are not recorded in primary care in Denmark. As a surrogate measure for HZ treated in this setting, we therefore used the Danish National Prescription Registry to identify prescriptions for systemic acyclovir, valacyclovir, and famciclovir at tablet doses most likely to represent treatment for HZ (800 mg acyclovir in packages with 35 pills or a 500-mg tablet dose of valacyclovir or famciclovir) [22].

Persons with any previous prescription for 1 of the 3 antivirals were ineligible, because repeated use is more common for competing indications (ie, reactivating herpes simplex). Given that herpes simplex is most frequent in young persons [23], we included only individuals aged ≥40 years in Denmark as well as in the United Kingdom (for comparability). To avoid including patients with long-term HZ-related complications, persons were ineligible if they had any previous diagnosis of postherpetic neuralgia from general practice (not available in Denmark) or the hospital-based setting or if they had a hospital-based HZ diagnosis not recorded as the primary diagnosis for the hospital contact. In the UK study, we also required that persons had been registered with their current general practice for ≥12 months before the index date to exclude past history of HZ recorded shortly after registration [24]. The index date for case patients was the earliest of the following: date of primary care diagnosis (date a relevant antiviral drug was dispensed in Denmark), date of hospital admission, or start of outpatient clinic follow-up.

We individually matched up to 4 population controls to each case patient by age, sex, and general practice (the United Kingdom only) using risk-set sampling [25] from the Civil Registration System in Denmark and from the CPRD in the United Kingdom. We gave preference to controls who were closest in age to the case patient, allowing a 2-month difference in Denmark and up to 1 year in the United Kingdom, where only year of birth was available. Controls were assigned the same index date as their case patient, and we applied the same inclusion criteria. In the United Kingdom, we excluded inactive controls (persons with no consultation record in the CPRD in the period 6 months before to 12 months after the index date) after matching [26]. In the main analysis, case patients and controls were included regardless of partner/civil status. Because the HZ vaccine was introduced in September 2014 in Denmark and in September 2013 in the United Kingdom, the vast majority of study participants were unvaccinated.

### Partner Bereavement

The full exposure definitions are summarized in Supplementary Appendix 2. In the Danish study, we identified partners using an algorithm developed by Statistics Denmark, a government-funded institution responsible for collecting, processing, and publishing data for various scientific purposes [27]. The algorithm combines data on civil status, kinship, exact address, birth year, and sex registered in the Civil Registration System to identify partners (married persons, same-sex couples living in a registered partnership, and nonmarried cohabitating couples). Because the personal identifiers for the couple are available, it was possible to accurately identify the vital status of case patients’ and controls’ current or previous partners.

In the United Kingdom, we adapted a previously described method to identify partners in the CPRD based on the “family number,” which identifies persons in a practice who live in the same household or who are otherwise associated (eg, live in the same institution) [28]. Cohabitees were classified as partners if they were persons of the opposite sex, with an age gap of ≤10 years, and with no younger adult in the household within ≤15 years of age of either person in the couple [28]. We applied these age criteria to avoid misclassifying the death of a child as partner bereavement. We did not consider cohabitees to be partners if the case patient or control had codes in the primary care record indicating residence in a communal establishment before the index date, if both individuals in the couple were aged ≥95 years, and/or if the same family number was used for >10 persons registered with the practice. We used the death date in the deceased partner’s primary care record as the date of bereavement.

To explore whether the association between bereavement and HZ depended on whether the death of a partner was unforeseen, we computed their age-adjusted Charlson Comorbidity Index score, in both UK and Danish data. This index assigns 0–6 points to various chronic diseases according to their ability to predict death, with additional points given according to age [29]. Based on the total score, we categorized risk of partner death as low (0–3 points), intermediate (4–6 points), or high (≥7 points). We excluded records within the month before death to avoid including diagnoses coded retrospectively at death (eg, the cause of death). As an alternative measure in the UK study, we also examined primary care and hospital records for terminal disease among partners before time of death (Supplementary Appendix 2).

### Statistical Analysis

We used conditional logistic regression to compute unadjusted odds ratios (ORs) associating previous partner bereavement with HZ. We selected 99% confidence intervals (CIs) as a measure of precision and based interpretations on clinical significance of the point estimates rather than dichotomizing to statistical significance according to an arbitrary significance level [30]. Given the risk-set sampling of controls, the ORs provide an unbiased estimate of the incidence rate ratios [25]. In multivariable analyses, we also adjusted for potential risk factors for HZ [26], including previous records of rheumatoid arthritis, systemic/subacute lupus erythematosus, inflammatory bowel disease, chronic obstructive pulmonary disease, asthma, diabetes, chronic kidney disease, human immunodeficiency virus infection, hematopoietic stem cell or bone marrow transplantation, solid organ transplantation, or other cellular immune deficiency at any time before index date; leukemia, lymphoma or myeloma within 2 years before the index date; and prescription records for oral glucocorticoids, other immunosuppressant drugs, or inhaled glucocorticoids within 90 days before the index date (see Supplementary Appendix 2 for definitions used).

We hypothesized that an increase in risk of HZ would be most pronounced within the first 3 months after bereavement. Within this 3-month period there could further be some variation related to the time from bereavement to decline in immunity and onset of HZ. To detect discrete fluctuations in the OR, we therefore examined the association between HZ and partner bereavement within 0–7, 8–14, 15–30, 31–90, 91–365, 366–1095, or >1095 days before the index date. Persons who had not previously experienced partner death provided the reference in all comparisons. As the Danish and UK studies were designed to resemble each other closely, we pooled the main results using DerSimonian and Lairds’ random-effects model [31]. We used the *I*^2^ statistic to estimate the percentage of inconsistency between study estimates that cannot be explained by chance alone [32].

In stratified analyses, we examined whether ORs for bereavement within 0–30 days before the index date depended on risk of partner death (based on their Charlson Comorbidity Index and records of terminal disease), age, or sex. We also determined whether ORs were higher among persons with medical records indicating depression or anxiety within 90 days before the index date, because we hypothesized that bereavement may provoke or exacerbate these conditions [10] and thereby cause HZ [26].

### Sensitivity Analyses

We performed several planned sensitivity analyses, described in more detail in Supplementary Appendix 3. Briefly, we first repeated the stratified analyses using a 90-day exposure window. Second, we excluded single subjects from the reference group. Third, we adjusted for individual-level measures of socioeconomic status, as it may be associated both with inequality in life expectancy (and thus probability of partner death) and with timely healthcare seeking for HZ. We used highest level of achieved education in Denmark (available for 90%) and quintiles of the patient-level Index of Multiple Deprivation scores in the United Kingdom (available for 60%). Fourth, in the UK study, we examined the impact of adjusting for smoking status, alcohol consumption, and body mass index. Because data were missing for 12% of subjects in this analysis, we used both a complete-case approach and multiple imputation by chained equations [33]. Valid lifestyle data were not available in the Danish study [34]. Finally, we repeated the Danish analyses after excluding case patients identified based on prescriptions for which the indication code did not state HZ. We did not use indication codes for the main analyses due to incomplete and unspecific coding. We performed all analyses using the Stata statistical software package (StataCorp).

## RESULTS

**Figure 1.**
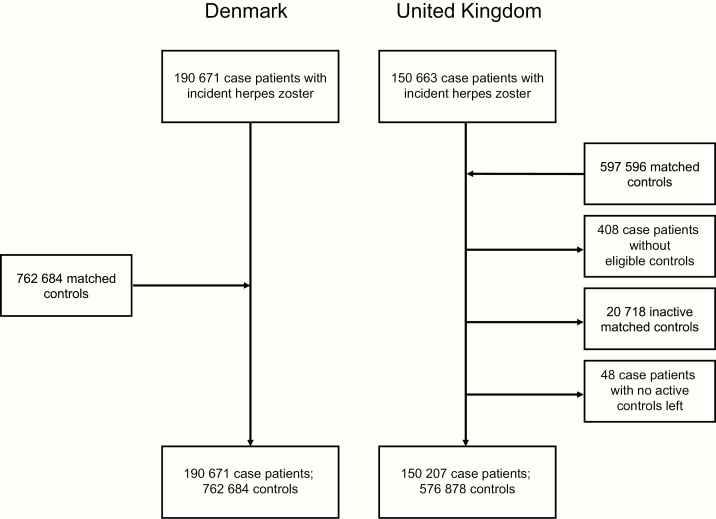
Flowchart for the studies. Inclusion criteria for case patients and controls were age ≥40 years; no previous Read Code or *International Classification of Diseases*, 10th revision, code for postherpetic neuralgia; no previous prescription for systemic acyclovir, valacyclovir, or famciclovir (Denmark only); and registration with current general practice for ≥12 months before the index date (United Kingdom only).

We included 190671 HZ case patients and 762684 controls in the Danish study and 150207 HZ case patients and 576878 controls in the UK study (Figure 1). Median age was approximately 65 years, and >60% were women (Table 1). The relative distribution of HZ risk factors among case patients versus controls was very similar in the 2 studies, although absolute numbers differed, particularly for asthma, chronic kidney disease, and inhaled glucocorticoids (Supplementary Appendix 3).

**Table 1.** Distribution of Matching Factors Among Herpes Zoster Case Patients and Controls

Factor	Denmark, No. (%)^a^		United Kingdom, No. (%)^a^	
	Case Patients (n = 190671)	Controls (n = 762684)	Case Patients (n = 150207)	Controls (n = 576878)
Sex				
Female	125526 (65.8)	502104 (65.8)	90501 (60.3)	354057 (61.4)
Male	65145 (34.2)	260580 (34.2)	59706 (39.7)	222821 (38.6)
Age at index date, median (IQR), y	64 (53–75)	64 (53–75)	65 (55–75)	65 (55–75)
Age group at index date				
40–49 y	34838 (18.3)	139352 (18.3)	20844 (13.9)	77009 (13.3)
50–59 y	41898 (22.0)	167592 (22.0)	33632 (22.4)	127508 (22.1)
60–69 y	45662 (23.9)	182648 (23.9)	38437 (25.6)	150110 (26.0)
70–79 y	39264 (20.6)	157056 (20.6)	34767 (23.1)	136694 (23.7)
80–89 y	23968 (12.6)	95872 (12.6)	19454 (13.0)	75566 (13.1)
≥90 y	5041 (2.6)	20164 (2.6)	3073 (2.0)	9991 (1.7)
Socioeconomic status (practice level)				
1 (least deprived)	…	…	29889 (19.9)	114855 (19.9)
2	…	…	29529 (19.7)	113395 (19.7)
3	…	…	32306 (21.5)	124101 (21.5)
4	…	…	30580 (20.4)	117216 (20.3)
5 (most deprived)	…	…	27903 (18.6)	107311 (18.6)

Abbreviation: IQR, interquartile range.

^a^Data represent No. (%) of case patients or controls, unless otherwise specified.

**Figure 2.**
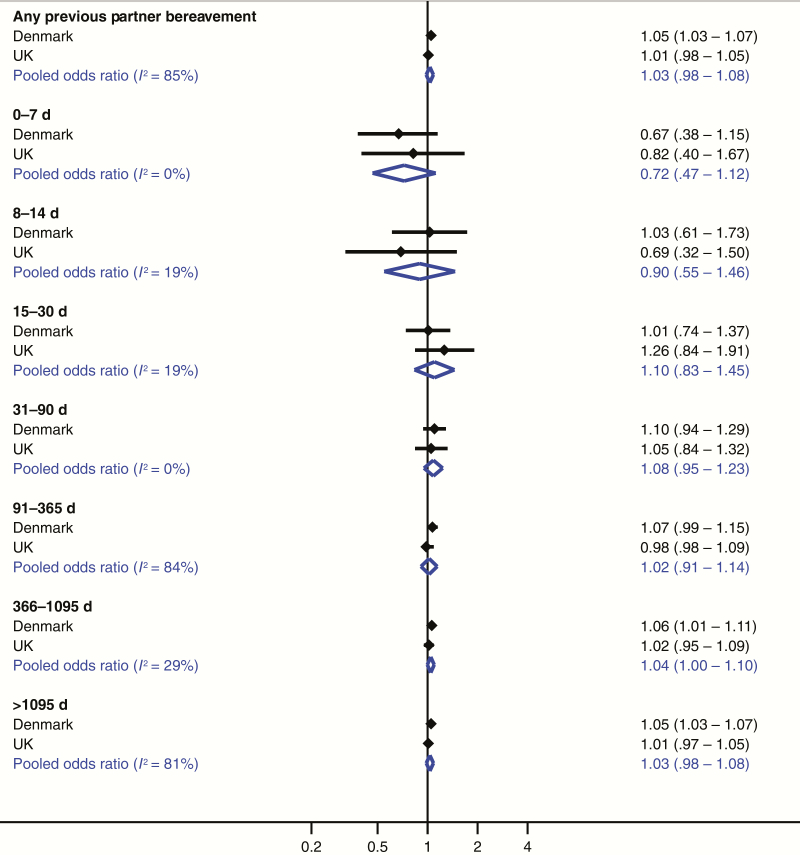
Pooled adjusted odds ratios (99% confidence intervals) from meta-analysis of the association between partner bereavement and herpes zoster in the Danish and UK studies. Odds ratios were adjusted for rheumatoid arthritis, lupus erythematosus, inflammatory bowel disease, chronic obstructive pulmonary disease, asthma, diabetes, chronic kidney disease, human immunodeficiency virus infection, hematopoietic stem cell/bone marrow transplantation, solid organ transplantation, other cellular immune deficiency, leukemia, lymphoma, myeloma, oral glucocorticoids, other immunosuppressant drugs, and inhaled glucocorticoids.

The adjusted OR for any previous partner bereavement was 1.05 (99% CI, 1.03–1.07) in Denmark and 1.01 (.98–1.05) in the United Kingdom (Table 2). Unadjusted and adjusted ORs were very similar. The *I*^2^ statistics from the meta-analysis were <20% within 0–90 days before the index date (Figure 2). Although we found evidence of statistical heterogeneity for remaining exposure windows, the effect estimates were similar, and neither study supported a substantial increase in relative risk. We therefore combined the estimates for all periods. The pooled adjusted ORs were 0.72 (99% CI, .47–1.12), 0.90 (.55–1.46), 1.10 (.83–1.45), 1.08 (.95–1.23), 1.02 (.91–1.14), 1.04 (1.00–1.10), and 1.03 (.98–1.06) within 0–7, 8–14, 15–30, 31–90, 91–365, 366–1095, and >1095 days before the index date, respectively. In both settings, the suggestion of an initial decrease in the OR observed within 14 days before the index date was followed by a compensatory increase within 15–90 days.

**Table 2.** Odds Ratio for Association Between Partner Bereavement and Herpes Zoster

Bereavement Status	Denmark				United Kingdom			
	Case Patients, No. (%)	Controls, No. (%)	Unadjusted OR (99% CI)	Adjusted OR (99% CI)^a^	Case Patients, No. (%)	Controls, No. (%)	Unadjusted OR (99% CI)	Adjusted OR (99% CI)^a^
Never bereaved	157076 (82.4)	633082 (83.0)	Reference	Reference	141774 (94.4)	544495 (94.4)	Reference	Reference
Bereaved, by duration of bereavement								
Total	33595 (17.6)	129602 (17.0)	1.06 (1.04–1.08)	1.05 (1.03–1.07)	8433 (5.6)	32383 (5.6)	1.01 (.98–1.05)	1.01 (.98–1.05)
0–7 d	26 (0.01)	159 (0.02)	0.66 (.38–1.15)	0.67 (.38–1.15)	16 (0.01)	77 (0.01)	0.81 (.40–1.64)	0.82 (.40–1.67)
8–14 d	31 (0.02)	126 (0.02)	1.00 (.60–1.68)	1.03 (.61–1.73)	13 (0.01)	74 (0.01)	0.69 (.32–1.50)	0.69 (.32–1.50)
15–30 d	90 (0.05)	367 (0.05)	1.00 (.73–1.35)	1.01 (.74–1.37)	52 (0.03)	165 (0.03)	1.24 (.82–1.87)	1.26 (.84–1.91)
31–90 d	343 (0.2)	1273 (0.2)	1.09 (.94–1.28)	1.10 (.94–1.29)	171 (0.1)	629 (0.1)	1.06 (.85–1.32)	1.05 (.84–1.32)
91–365 d	1572 (0.8)	5977 (0.8)	1.07 (.99–1.15)	1.07 (.99–1.15)	746 (0.5)	2956 (0.5)	0.98 (.88–1.09)	0.98 (.88–1.09)
366–1095 d	3989 (2.1)	15322 (2.0)	1.06 (1.01–1.11)	1.06 (1.01–1.11)	1755 (1.2)	6712 (1.2)	1.02 (.95–1.09)	1.02 (.95–1.09)
>1095 d	27544 (14.4)	106378 (13.9)	1.06 (1.03–1.08)	1.05 (1.03–1.07)	5680 (3.8)	21770 (3.8)	1.02 (.97–1.06)	1.01 (.97–1.05)

Abbreviations: CI, confidence interval; OR, odds ratio.

^a^Adjusted for rheumatoid arthritis, lupus erythematosus, inflammatory bowel disease, chronic obstructive pulmonary disease, asthma, diabetes, chronic kidney disease, human immunodeficiency virus infection, hematopoietic stem cell/bone marrow transplantation, solid organ transplantation, other cellular immune deficiency, leukemia, lymphoma, myeloma, oral glucocorticoids, other immunosuppressant drugs, and inhaled glucocorticoids.

**Figure 3.**
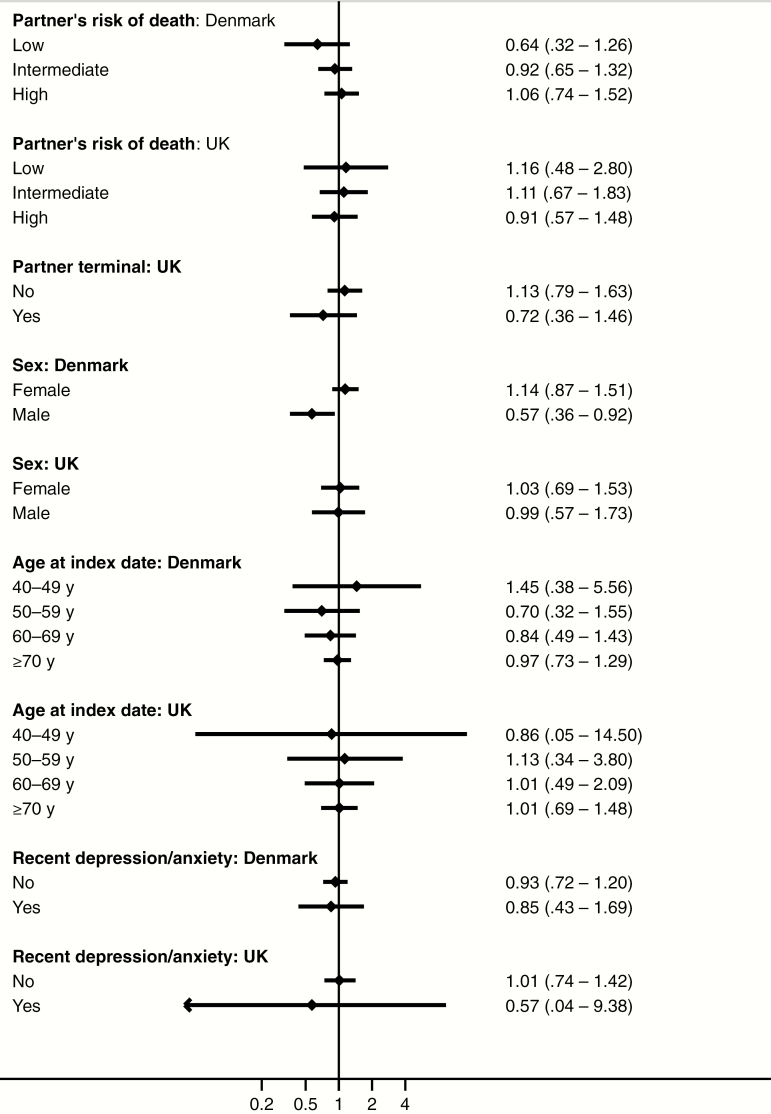
Adjusted odds ratios (99% confidence intervals) for herpes zoster among persons experiencing partner bereavement within the previous 30 days compared with those who had never been bereaved, according to subgroups based on partner’s risk of death, sex, age at index date, and recent diagnosis of depression or anxiety. Odds ratios were adjusted for rheumatoid arthritis, lupus erythematosus, inflammatory bowel disease, chronic obstructive pulmonary disease, asthma, diabetes, chronic kidney disease, human immunodeficiency virus infection, hematopoietic stem cell/bone marrow transplantation, solid organ transplantation, other cellular immune deficiency, leukaemia, lymphoma, myeloma, oral glucocorticoids, other immunosuppressant drugs, and inhaled glucocorticoids. The partner’s risk of death was computed using the age-adjusted Charlson Comorbidity Index score and categorized as low (0–3 points), intermediate (4–6 points), or high (≥7 points).

We found no substantial variation in estimates after stratifying by risk of partner death, age, sex, or recent depression/anxiety (Figure 3 and Supplementary Appendix 3). However, meaningful comparisons were hampered by very wide CIs. Results were robust in all sensitivity analyses (Supplementary Appendix 3).

## DISCUSSION

This large population-based study using data from Denmark and the United Kingdom found no evidence of a substantially increased relative risk of HZ after partner bereavement. Data from the 2 settings showed similar distribution of well-known risk factors for HZ and effect estimates of similar magnitude for risk of HZ after bereavement.

Our findings corroborate a recent self-controlled case series [8]. Among 39811 persons experiencing death or an intensive care unit stay lasting >14 days for a previously healthy spouse (insurance cobeneficiaries within 5 years of age and of opposite sex), 59 persons were diagnosed with HZ within the following 90 days, compared with 78 in the control period 31–120 days before exposure, yielding an incidence ratio of 0.76 (95% CI, .54–1.06). Furthermore, the proportion of outpatient healthcare contacts attributed to HZ was not higher than in the control period (relative risk 0.99; 95% CI, .70–1.39). In contrast, in 3 case-control studies, which included 101–389 HZ case patients and 101–511 controls, ORs ranged between 2.64 and 3.40 for self-reported negative life events in the previous 3–6 months [4, 5, 7]. Similarly, in a cohort study of 4162 elderly volunteers, an increased hazard ratio of HZ (1.38; 95% CI, .96–1.97) was observed among persons who reported negative life events in the prior 1–4 years [6]. The discrepancy between the results from these interview-based studies and our study, as well as the previous self-controlled case series, may be explained by important methodological differences, including use of aggregate measures for negative life events [4–7], potential self-selection bias [4–7], lack of interviewer blinding [4, 5, 7], potential recall bias [4, 5, 7], and limited sample sizes [4–7].

Immunological studies show that bereavement is associated with functional cellular immune deficiency [3]. Our study suggests that this effect may not be clinically significant for triggering HZ, because the overall upper confidence limit was only 7% in Denmark and the early fluctuations in ORs are compatible with delayed healthcare seeking among bereaved persons. Nevertheless, it is possible that other types of psychological stress, such as that associated with psychiatric illness, elicit different immune responses than those observed after the, predominantly acute, stress of partner bereavement [3]. For example, it has been demonstrated that persons with major depression have reduced cell-mediated immunity against the varicella zoster virus [35–37].

Major strengths of our study include the large study size, use of prospectively collected data from 2 separate tax-supported healthcare systems, and availability of detailed data on temporality of exposure and outcome. However, several limitations need to be considered. We believe that delayed healthcare contact immediately after loss explains the potential transient decrease in the ORs within 14 days after bereavement. We anticipated that such delay could introduce bias in the Danish study, because patients who present late with HZ may not be prescribed antivirals [22], thus omitting them from study inclusion. Another concern for the prescription-based algorithm is misclassification of herpes simplex, which might be provoked by acute stress [1]. Nevertheless, the similarity between results observed in Denmark and the United Kingdom, including the initial decrease in the OR, suggests that such these biases are negligible.

Misclassification of partner bereavement is possible, in particular in the United Kingdom where data used for identifying partners were less detailed than in Denmark. Use of the general practice family number to identify cohabitating persons may have affected the completeness of our algorithm, as some partners may not be registered with the same general practice. Nevertheless, our results remained robust after excluding single persons from the reference group. Furthermore, the prevalence of previous bereavement was remarkably similar in the Danish and UK data. The only difference was a lower prevalence in the United Kingdom >1 year before the index date, which is consistent with the shorter observation period in the CPRD.

A previous study reported that, according to contemporary national representative household surveys in England, 99% of cohabitating persons aged ≥60 years who are of the opposite sex and have an age difference of <10 years identify themselves as partners [28]. Although these data support a high accuracy of our algorithm, some couples may have represented cohabitating friends or siblings. Still, such misclassification would capture bereavement of a significant person in someone’s life, which is also likely to be stressful.

Finally, imprecise estimates limited identification of effect measure modification by the partner’s risk of death. Expectation of death is also difficult to categorize and associated psychological distress could depend on the type of chronic disease [10]. Furthermore, because the majority of partners were considered at intermediate or high risk of death, the time of bereavement may not mark the beginning of the stressful period.

In conclusion, we found no evidence of a substantial increase in the risk of HZ after partner bereavement. The observed decrease in the relative risk of HZ within 14 days after bereavement followed by corresponding increased risks within subsequent months is compatible with delayed healthcare contact due to the loss.

## Supplementary Data

Supplementary materials are available at *Clinical Infectious Diseases* online. Consisting of data provided by the authors to benefit the reader, the posted materials are not copyedited and are the sole responsibility of the authors, so questions or comments should be addressed to the corresponding author.

## Supplementary Material

Supplementary_Appendix_3

Supplementary_Appendix_2

Supplementary_Appendix_5

Supplementary_Appendix_4

Supplementary_Appendix_1

## Notes

***Author contributions.*** All authors participated in designing the study. Analyses were performed by S. A. J. S. for the UK study and by H. S. P. (sampling and initial data management) and S. A. J. S. (final data management and analyses) for the Danish study. All authors participated in the discussion and interpretation of the results. S. A. J. S. wrote the initial manuscript draft. All authors critically revised the manuscript for intellectual content and approved the final version. S. A. J. S. is the guarantor.

***Financial support.*** This work was supported by Christian and Ottilia Brorsons’ grant for young scientists; Torben and Alice Frimodts’ Fund; a National Institute for Health Research (NIHR) Clinician Scientists Award (grant NIHR/CS/010/014 to S. M. L.); the Lundbeck Foundation (grant R155-2012–11280); the Program for Clinical Research Infrastructure, established by the Lundbeck Foundation; the Novo Nordisk Foundation; and the Wellcome Trust (L. S.).

***Potential conflicts of interest***. S. A. J. S. reports grants from Christian and Ottilia Brorsons’ Grant for young scientists and Torben and Alice Frimodts’ Fund, during the conduct of the study. S. L. T. reports a grant from NIHR, for work outside the submitted work. L. S. reports grants from Wellcome Trust and BHF during the conduct of the study; reports grants from Wellcome Trust, MRC, NIHR, GSK, and the European Union, personal fees from GSK, and other support from AstraZeneca, outside the submitted work; and is a trustee of the British Heart Foundation. H. T. S. did not report receiving fees, honoraria, grants ,or consultancies; the Department of Clinical Epidemiology, however, is involved in studies with funding from various companies as research grants to (and administered by) Aarhus University, but none of these studies are related to the present study. S. M. L. reports grants from NIHR, during the conduct of the study. All other authors declare no potential conflicts. The authors have submitted the ICMJE Form for Disclosure of Potential Conflicts of Interest. Conflicts that the editors consider relevant to the content of the manuscript have been disclosed.
