# Supplementary material for: Partner Bereavement and Risk of Herpes Zoster: Results from Two Population-Based Case-Control Studies in Denmark and the United Kingdom
Source: Clin Infect Dis. 2016 Dec 15;64(5):572–9. doi: 10.1093/cid/ciw840 (PMC5850543; doi:10.1093/cid/ciw840)
Supplement: Supplementary_Appendix_3 [file ciw840_suppl_supplementary_appendix_3.doc]

**Supplementary Appendix 3.** **Additional results, including subgroup and sensitivity analyses**

Additional results showing the distribution of herpes zoster risk factors and lifestyle factors among cases and controls is presented in Table 3a below.

In the main analysis, we stratified the results for the 30-day period prior to index date. The results are shown in detail in Table 3b. As we hypothesized that an effect of the immune function could potentially be delayed, we also performed a sensitivity analysis using a 90-day exposure window. The results of this analysis supported the main conclusion, as shown in Table 3c.

We performed analyses including only persons with partners in the reference group. In total, 323,301 (33.9%) and 336,417 (46.3%) participants had no partner in the Danish and British data, respectively. Exclusion of these persons from the reference group did not affect the estimates materially (Table 3d).

We additionally adjusted for the individual-level measures of socioeconomic status, using highest achieved education in Denmark and quintiles of Index of Multiple Deprivation score in the UK. Socioeconomic status was evenly distributed among cases and controls (Table 3e) and adjustment for these measures had no substantial effect on the results (Table 3f).

Smoking and alcohol has been associated with suppression of the immune system and could thus potentially increase the risk of herpes zoster [1,2]. Furthermore, these health-related behaviours are associated with increased risk of death from many causes and could potentially be shared by partners. We therefore examined the impact of adjusting for smoking status, alcohol consumption and obesity in the British data. As 12% had missing data on at least one of these variables, we performed analyses using both complete-case analysis and multiple imputation. Missing data were more frequent among males, in the upper age categories, among patients without the included risk factors for herpes zoster, and among controls. For multiple imputation, we assumed that incomplete data were missing at random and used the method of chained equations to create 20 imputed datasets [3]. We used multinomial models for the imputation procedure and included as predictors the outcome (case status), age, sex, and all variables from the main outcome model, as well as other lifestyle related diseases, including myocardial infarction, other ischemic heart disease, congestive heart failure, hypertension, cerebrovascular disease, peripheral artery disease, alcoholic liver disease (including portal hypertension) and pancreatitis. We included no non-normally distributed continuous variables. We evaluated the distributions of observed and imputed values for comparability and found them to be consistent. Adjustment for lifestyle factors did not change the results, regardless of the analytical method used to account for missing data (Table 3g).

Because we were concerned about inclusion of other herpes infections in the prescription-based definition in the Danish data, we examined the robustness of our algorithm in a sensitivity analysis excluding cases identified through prescriptions where the indication code did not explicitly state herpes zoster. As indications are recorded since April 2004, this analysis also ensured 10 years of prescription data for ascertaining true first-time prescriptions for antiviral drugs. The indication codes were included only in the sensitivity analysis because of problems with incomplete data (23% included from April 2004 and onwards had no indication recorded) and unspecific coding (e.g.*,* “herpes infection”), and because the validity of indication codes for research has not been examined. The results of this sensitivity analysis were similar to the main analysis (Table 3h).

**Table 3a. Characteristics of herpes zoster cases and matched controls. Values are numbers (%)**

|  | **Denmark** | | **The UK** | |
| --- | --- | --- | --- | --- |
|  | **Cases**  **(n=190,671)** | **Controls**  **(n=762,684)** | **Cases**  **(n=150,207)** | **Controls**  **(n=576,878)** |
| **Herpes zoster risk factors^a^** |  |  |  |  |
| Rheumatoid arthritis | 4,091 (2.1) | 9,157 (1.2) | 3,904 (2.6) | 9,970 (1.7) |
| Systemic/subacute lupus erythematosus | 464 (0.2) | 664 (0.1) | 454 (0.3) | 1,024 (0.2) |
| Inflammatory bowel disease | 2,710 (1.4) | 7,324 (1.0) | 2,176 (1.4) | 6,316 (1.1) |
| Chronic obstructive pulmonary disease | 10,966 (5.8) | 31,252 (4.1) | 9,473 (6.3) | 28,325 (4.9) |
| Asthma | 2,875 (1.5) | 8,019 (1.1) | 11,214 (7.5) | 35,654 (6.2) |
| Chronic kidney disease | 2,826 (1.5) | 6,235 (0.8) | 11,099 (7.4) | 37,370 (6.5) |
| Diabetes | 18,827 (9.9) | 68,492 (9.0) | 14,486 (9.6) | 53,016 (9.2) |
| Type I | 383 (0.2) | 1,336 (0.2) | 276 (0.2) | 873 (0.2) |
| Type II | 17,144 (9.0) | 62,647 (8.2) | 13,509 (9.0) | 49,930 (8.7) |
| Unknown | 1,300 (0.7) | 4,509 (0.6) | 701 (0.5) | 2,213 (0.4) |
| Inhaled glucocorticoids | 11,255 (5.9) | 31,515 (4.1) | 12,879 (8.6) | 38,911 (6.7) |
| Solid organ transplantation | 195 (0.1) | 158 (0.02) | 247 (0.2) | 318 (0.06) |
| HIV infection | 361 (0.2) | 364 (0.04) | 126 (0.08) | 147 (0.03) |
| Leukemia | 782 (0.4) | 807 (0.1) | 369 (0.2) | 558 (0.1) |
| Lymphoma | 1,222 (0.6) | 1,034 (0.1) | 700 (0.5) | 778 (0.1) |
| Myeloma | 486 (0.3) | 327 (0.04) | 286 (0.2) | 254 (0.04) |
| Stem cell/bone marrow transplantation | 692 (0.4) | 486 (0.1) | 220 (0.1) | 114 (0.02) |
| Other unspecified cellular immune deficiencies | 283 (0.1) | 382 (0.1) | 315 (0.2) | 636 (0.1) |
| Oral glucocorticoids | 8,881 (4.7) | 16,247 (2.1) | 7,397 (4.9) | 16,043 (2.8) |
| Other immunosuppressive treatment | 4,309 (2.3) | 6,482 (0.8) | 3,035 (2.0) | 5,589 (1.0) |
| **Lifestyle factors** |  |  |  |  |
| Body mass index category |  |  |  |  |
| Underweight | – | – | 2,696 (1.8) | 10,186 (1.8) |
| Normal weight | – | – | 50,696 (33.8) | 192,086 (33.3) |
| Overweight | – | – | 53,145 (35.4) | 199,973 (34.7) |
| Obese | – | – | 33,667 (22.4) | 126,021 (21.8) |
| Missing | – | – | 10,003 (6.6) | 48,612 (8.4) |
| Smoking status |  |  |  |  |
| Non-smoker | – | – | 55,570 (37.0) | 215,386 (37.3) |
| Current smoker | – | – | 32,768 (21.8) | 132,857 (23.0) |
| Ex-smoker | – | – | 60,844 (40.5) | 219,653 (38.1) |
| Missing | – | – | 1,025 (0.7) | 8,982 (1.6) |
| Alcohol use |  |  |  |  |
| Non-drinker | – | – | 14,936 (9.9) | 58,753 (10.2) |
| Current drinker | – | – | 109,461 (72.9) | 413,351 (71.7) |
| Ex-drinker | – | – | 15,331 (10.2) | 55,662 (9.6) |
| Missing | – | – | 10,479 (7.0) | 49,112 (8.5) |

^a^ Defined at any time prior to index date, except for leukemia, lymphoma and myeloma (any diagnosis within prior two years) and inhaled glucocorticoids, oral glucocorticoids, and other immunosuppressive treatment (any record within prior 90 days).

**Table 3b.** **Odds ratios for the association between partner bereavement within previous 30 days and herpes zoster, subgroup analysis**

|  | **Denmark** | | | | **The UK** | | | |
| --- | --- | --- | --- | --- | --- | --- | --- | --- |
|  | **Cases, number (%)** | **Controls, number (%)** | **Unadjusted odds ratio (99% CI)** | **Adjusted odds ratio (99% CI)**^a^ | **Cases, number (%)** | **Controls, number (%)** | **Unadjusted odds ratio (99% CI)** | **Adjusted odds ratio (99% CI)**^a^ |
| Partner’s risk of death^b^ |  |  |  |  |  |  |  |  |
| Low | 17 (0.01) | 107 (0.01) | 0.64 (0.33 to 1.26) | 0.64 (0.32 to 1.26) | 11 (0.01) | 39 (0.01) | 1.13 (0.47 to 2.72) | 1.16 (0.48 to 2.80) |
| Intermediate | 65 (0.03) | 294 (0.04) | 0.90 (0.63 to 1.28) | 0.92 (0.65 to 1.32) | 34 (0.02) | 122 (0.02) | 1.08 (0.66 to 1.79) | 1.11 (0.67 to 1.83) |
| High | 65 (0.03) | 251 (0.03) | 1.05 (0.73 to 1.51) | 1.06 (0.74 to 1.52) | 36 (0.02) | 155 (0.03) | 0.91 (0.57 to 1.47) | 0.91 (0.57 to 1.48) |
| Partner terminal |  |  |  |  |  |  |  |  |
| No | – | – | – | – | 65 (0.04) | 227 (0.04) | 1.12 (0.78 to 1.61) | 1.13 (0.79 to 1.63) |
| Yes | – | – | – | – | 16 (0.01) | 89 (0.02) | 0.71 (0.35 to 1.43) | 0.72 (0.36 to 1.46) |
| Sex |  |  |  |  |  |  |  |  |
| Female | 112 (0.06) | 436 (0.06) | 1.13 (0.86 to 1.49) | 1.14 (0.87 to 1.51) | 54 (0.04) | 208 (0.04) | 1.02 (0.69 to 1.52) | 1.03 (0.69 to 1.53) |
| Male | 35 (0.02) | 216 (0.03) | 0.56 (0.35 to 0.90) | 0.57 (0.36 to 0.92) | 27 (0.02) | 108 (0.02) | 0.97 (0.56 to 1.70) | 0.99 (0.57 to 1.73) |
| Age at index date (years) |  |  |  |  |  |  |  |  |
| 40–49 | 5 (0.003) | 14 (0.002) | 1.44 (0.38 to 5.54) | 1.45 (0.38 to 5.56) | 1 (0.0006) | 5 (0.0009) | 0.80 (0.05 to 13.51) | 0.86 (0.05 to 14.50) |
| 50–59 | 13 (0.01) | 70 (0.01) | 0.75 (0.34 to 1.63) | 0.70 (0.32 to 1.55) | 6 (0.003) | 20 (0.003) | 1.15 (0.35 to 3.82) | 1.13 (0.34 to 3.80) |
| 60–69 | 29 (0.02) | 138 (0.02) | 0.85 (0.50 to 1.44) | 0.84 (0.49 to 1.43) | 16 (0.01) | 62 (0.01) | 1.02 (0.50 to 2.11) | 1.01 (0.49 to 2.09) |
| ≥70 | 100 (0.05) | 430 (0.06) | 0.94 (0.71 to 1.25) | 0.97 (0.73 to 1.29) | 58 (0.04) | 229 (0.04) | 0.99 (0.68 to 1.45) | 1.01 (0.69 to 1.48) |
| Recent depression/anxiety |  |  |  |  |  |  |  |  |
| No | 129 (0.07) | 580 (0.08) | 0.92 (0.72 to 1.19) | 0.93 (0.72 to 1.20) | 80 (0.05) | 310 (0.05) | 1.01 (0.73 to 1.40) | 1.01 (0.74 to 1.42) |
| Yes | 18 (0.01) | 72 (0.01) | 0.82 (0.42 to 1.62) | 0.85 (0.43 to 1.69) | 1 (0.0006) | 6 (0.001) | 0.58 (0.04 to 9.37) | 0.57 (0.04 to 9.38) |
| ^a^Adjusted for rheumatoid arthritis, lupus erythematosus, inflammatory bowel disease, chronic obstructive pulmonary disease, asthma, diabetes, chronic kidney disease, HIV infection, hematopoietic stem cell/bone marrow transplantation, solid organ transplantation, other cellular immune deficiency, leukemia, lymphoma, myeloma, oral glucocorticoids, other immunosuppressant drugs and inhaled glucocorticoids.  ^b^Measured by the age-adjusted Charlson Comorbidity Index with the total score categorized as low (0–3 points), intermediate (4–6 points) or high (≥7 points). | | | | | | | | |

**Table 3c.** **Odds ratios for the association between partner bereavement within previous 90 days and herpes zoster, subgroup analysis**

|  | **Denmark** | | | | **The UK** | | | |
| --- | --- | --- | --- | --- | --- | --- | --- | --- |
|  | **Cases, number (%)** | **Controls, number (%)** | **Unadjusted odds ratio (99% CI)** | **Adjusted odds ratio (99% CI)^a^** | **Cases, number (%)** | **Controls, number (%)** | **Unadjusted odds ratio (99% CI)** | **Adjusted odds ratio (99% CI)^a^** |
| Partner’s risk of death^b^ |  |  |  |  |  |  |  |  |
| Low | 59 (0.03) | 298 (0.04) | 0.80 (0.55 to 1.15) | 0.79 (0.55 to 1.14) | 26 (0.02) | 122 (0.02) | 0.84 (0.48 to 1.47) | 0.82 (0.47 to 1.44) |
| Intermediate | 233 (0.12) | 871 (0.11) | 1.09 (0.90 to 1.32) | 1.10 (0.91 to 1.34) | 112 (0.07) | 374 (0.06) | 1.16 (0.88 to 1.53) | 1.18 (0.89 to 1.57) |
| High | 198 (0.10) | 756 (0.10) | 1.07 (0.87 to 1.31) | 1.07 (0.87 to 1.32) | 114 (0.08) | 449 (0.08) | 0.99 (0.76 to 1.30) | 0.99 (0.75 to 1.30) |
| Partner terminal |  |  |  |  |  |  |  |  |
| No | – | – | – | – | 187 (0.12) | 711 (0.12) | 1.02 (0.83 to 1.26) | 1.02 (0.82 to 1.26) |
| Yes | – | – | – | – | 65 (0.04) | 234 (0.04) | 1.09 (0.76 to 1.57) | 1.11 (0.77 to 1.60) |
| Sex |  |  |  |  |  |  |  |  |
| Female | 366 (0.19) | 13,11 (0.17) | 1.24 (1.06 to 1.44) | 1.25 (1.07 to 1.46) | 183 (0.12) | 638 (0.11) | 1.13 (0.91 to 1.40) | 1.13 (0.91 to 1.40) |
| Male | 124 (0.07) | 614 (0.08) | 0.70 (0.54 to 0.90) | 0.70 (0.54 to 0.91) | 69 (0.05) | 307 (0.05) | 0.86 (0.61 to 1.21) | 0.86 (0.61 to 1.22) |
| Age at index date (years) |  |  |  |  |  |  |  |  |
| 40–49 | 18 (0.01) | 44 (0.01) | 1.65 (0.80 to 3.40) | 1.65 (0.80 to 3.41) | 2 (0.001) | 14 (0.002) | 0.54 (0.08 to 3.82) | 0.58 (0.08 to 4.10) |
| 50–59 | 37 (0.02) | 189 (0.02) | 0.79 (0.50 to 1.26) | 0.76 (0.47 to 1.21) | 16 (0.01) | 71 (0.01) | 0.86 (0.42 to 1.75) | 0.84 (0.41 to 1.71) |
| 60–69 | 112 (0.06) | 402 (0.05) | 1.12 (0.85 to 1.48) | 1.11 (0.84 to 1.47) | 49 (0.03) | 172 (0.03) | 1.12 (0.73 to 1.70) | 1.10 (0.72 to 1.68) |
| ≥70 | 323 (0.17) | 1,290 (0.17) | 1.01 (0.86 to 1.19) | 1.03 (0.88 to 1.22) | 185 (0.12) | 688 (0.12) | 1.05 (0.85 to 1.30) | 1.06 (0.85 to 1.31) |
| Recent depression/anxiety |  |  |  |  |  |  |  |  |
| No | 422 (0.22) | 1,726 (0.23) | 1.01 (0.88 to 1.17) | 1.02 (0.89 to 1.18) | 247 (0.16) | 909 (0.16) | 1.06 (0.88 to 1.28) | 1.06 (0.88 to 1.28) |
| Yes | 68 (0.04) | 199 (0.03) | 1.12 (0.78 to 1.61) | 1.15 (0.80 to 1.66) | 5 (0.003) | 36 (0.01) | 0.48 (0.14 to 1.64) | 0.48 (0.14 to 1.64) |
| ^a^Adjusted for rheumatoid arthritis, lupus erythematosus, inflammatory bowel disease, chronic obstructive pulmonary disease, asthma, diabetes, chronic kidney disease, HIV infection, hematopoietic stem cell/bone marrow transplantation, solid organ transplantation, other cellular immune deficiency, leukemia, lymphoma, myeloma, oral glucocorticoids, other immunosuppressant drugs and inhaled glucocorticoids.  ^b^Measured by the age-adjusted Charlson Comorbidity Index with the total score categorized as low (0–3 points), intermediate (4–6 points) or high (≥7 points). | | | | | | | | |

**Table 3d. Odds ratios (99% confidence intervals) for the association between partner bereavement and herpes zoster. Sensitivity analysis including only persons with partners in the reference group**

|  | **Denmark** | | **The UK** | |
| --- | --- | --- | --- | --- |
|  | **Unadjusted odds ratio** | **Adjusted odds ratio^a^** | **Unadjusted odds ratio** | **Adjusted odds ratio^a^** |
| Never bereaved | (reference) | (reference) | (reference) | (reference) |
| Bereaved | 1.06 (1.04 to 1.09) | 1.06 (1.04 to 1.08) | 0.99 (0.96 to 1.03) | 0.99 (0.95 to 1.02) |
| 0–7 days | 0.67 (0.39 to 1.15) | 0.67 (0.39 to 1.16) | 0.79 (0.39 to 1.61) | 0.80 (0.39 to 1.63) |
| 8–14 days | 1.01 (0.60 to 1.69) | 1.03 (0.61 to 1.74) | 0.68 (0.31 to 1.47) | 0.67 (0.31 to 1.46) |
| 15–30 days | 1.00 (0.74 to 1.36) | 1.01 (0.74 to 1.37) | 1.21 (0.80 to 1.83) | 1.23 (0.82 to 1.86) |
| 31–90 days | 1.10 (0.94 to 1.29) | 1.11 (0.94 to 1.30) | 1.04 (0.83 to 1.29) | 1.03 (0.82 to 1.29) |
| 91–365 days | 1.08 (1.00 to 1.16) | 1.08 (1.00 to 1.16) | 0.96 (0.87 to 1.07) | 0.96 (0.86 to 1.07) |
| 366–1095 days | 1.07 (1.02 to 1.12) | 1.06 (1.01 to 1.12) | 1.00 (0.93 to 1.07) | 0.99 (0.93 to 1.07) |
| >1095 days | 1.06 (1.04 to 1.09) | 1.06 (1.03 to 1.08) | 0.99 (0.95 to 1.04) | 0.99 (0.95 to 1.03) |
| ^a^Adjusted for rheumatoid arthritis, lupus erythematosus, inflammatory bowel disease, chronic obstructive pulmonary disease, asthma, diabetes, chronic kidney disease, HIV infection, hematopoietic stem cell/bone marrow transplantation, solid organ transplantation, other cellular immune deficiency, leukemia, lymphoma, myeloma, oral glucocorticoids, other immunosuppressant drugs and inhaled glucocorticoids. | | | | |

**Table 3e. Measures of patient-level socioeconomic status among cases and matched controls**

|  | **Cases**  **n (%)** | **Controls**  **n (%)** |
| --- | --- | --- |
| **Denmark** |  |  |
| Highest achieved education |  |  |
| Long (>15 years) | 33,006 (17.3) | 121,098 (15.9) |
| Medium (>10–15 years) | 72,508 (38.0) | 295,076 (38.7) |
| Short (≤10 years) | 66,494 (34.9) | 269,752 (35.4) |
| Missing | 18,663 (9.8) | 76,758 (10.1) |
| **The UK** |  |  |
| Quintile of Index of Multiple Deprivation score |  |  |
| 1 (least deprived) | 23,452 (15.6) | 90,245 (15.6) |
| 2 | 23,400 (15.6) | 89,782 (15.6) |
| 3 | 19,261 (12.8) | 74,420 (12.9) |
| 4 | 15,471 (10.3) | 59,239 (10.3) |
| 5 (most deprived) | 11,107 (7.4) | 42,102 (7.3) |
| Missing | 57,516 (38.3) | 221,090 (38.3) |

**Table 3f. Odds ratios (99% confidence intervals) for the association between partner bereavement and herpes zoster, adjusting for risk factors for herpes zoster and socioeconomic status^a^**

|  | **Denmark** | **The UK** |
| --- | --- | --- |
| Never bereaved | (reference) | (reference) |
| Bereaved | 1.06 (1.04 to 1.08) | 1.00 (0.95 to 1.04) |
| 0–7 days | 0.77 (0.44 to 1.36) | 0.43 (0.13 to 1.45) |
| 8–14 days | 1.14 (0.65 to 1.99) | 0.75 (0.29 to 1.94) |
| 15–30 days | 1.05 (0.76 to 1.45) | 1.28 (0.77 to 2.12) |
| 31–90 days | 1.10 (0.92 to 1.31) | 1.05 (0.79 to 1.38) |
| 91–365 days | 1.11 (1.02 to 1.20) | 0.93 (0.81 to 1.07) |
| 366–1095 days | 1.09 (1.03 to 1.15) | 1.01 (0.93 to 1.11) |
| >1095 days | 1.05 (1.03 to 1.08) | 1.00 (0.95 to 1.05) |
| ^a^Adjusted for rheumatoid arthritis, lupus erythematosus, inflammatory bowel disease, chronic obstructive pulmonary disease, asthma, diabetes, chronic kidney disease, HIV infection, hematopoietic stem cell/bone marrow transplantation, solid organ transplantation, other cellular immune deficiency, leukemia, lymphoma, myeloma, oral glucocorticoids, other immunosuppressant drugs, inhaled glucocorticoids and patient level socioeconomic status (quintiles of the Index of Multiple Deprivation Score in the UK and highest achieved education in Denmark) | | |

**Table 3g. Odds ratios (99% confidence intervals) for the association between partner bereavement and herpes zoster, adjusting for risk factors for herpes zoster and lifestyle factors in the British data^a^**

|  | **Complete-case analysis** | **Multiple imputation** |
| --- | --- | --- |
| Never bereaved | (reference) | (reference) |
| Bereaved | 1.01 (0.97 to 1.04) | 1.01 (0.98 to 1.05) |
| 0–7 days | 0.81 (0.38 to 1.76) | 0.82 (0.40 to 1.68) |
| 8–14 days | 0.58 (0.24 to 1.40) | 0.69 (0.32 to 1.50) |
| 15–30 days | 1.28 (0.82 to 1.99) | 1.27 (0.84 to 1.92) |
| 31–90 days | 1.03 (0.81 to 1.31) | 1.05 (0.84 to 1.32) |
| 91–365 days | 1.01 (0.90 to 1.13) | 0.98 (0.89 to 1.10) |
| 366–1095 days | 1.02 (0.94 to 1.10) | 1.02 (0.95 to 1.09) |
| >1095 days | 1.00 (0.96 to 1.05) | 1.01 (0.97 to 1.06) |
| ^a^Adjusted for rheumatoid arthritis, lupus erythematosus, inflammatory bowel disease, chronic obstructive pulmonary disease, asthma, diabetes, chronic kidney disease, HIV infection, hematopoietic stem cell/bone marrow transplantation, solid organ transplantation, other cellular immune deficiency, leukemia, lymphoma, myeloma, oral glucocorticoids, other immunosuppressant drugs, inhaled glucocorticoids, body mass index category, smoking status and alcohol use. | | |

**Table 3h.** Adjusted odds ratios (99% confidence intervals)^a^ for the association between partner bereavement and herpes zoster. Sensitivity analyses examining the robustness of the prescription-based definition of herpes zoster in the Danish data

|  | **Main analysis (entire study population)** | **Excluding those with indication codes not explicitly stating herpes zoster** |
| --- | --- | --- |
| Never bereaved | (reference) | (reference) |
| Bereaved | 1.05 (1.03 to 1.07) | 1.04 (1.01-1.08) |
| 0–7 days | 0.67 (0.38 to 1.15) | 0.70 (0.31-1.58) |
| 8–14 days | 1.03 (0.61 to 1.73) | 1.13 (0.54-2.35) |
| 15–30 days | 1.01 (0.74 to 1.37) | 0.98 (0.61-1.60) |
| 31–90 days | 1.10 (0.94 to 1.29) | 1.07 (0.83-1.38) |
| 91–365 days | 1.07 (0.99 to 1.15) | 1.08 (0.96-1.21) |
| 366–1095 days | 1.06 (1.01 to 1.11) | 1.07 (0.99-1.15) |
| >1095 days | 1.05 (1.03 to 1.07) | 1.04 (1.01-1.07) |
| ^a^Adjusted for rheumatoid arthritis, lupus erythematosus, inflammatory bowel disease, chronic obstructive pulmonary disease, asthma, diabetes, chronic kidney disease, HIV infection, hematopoietic stem cell/bone marrow transplantation, solid organ transplantation, other cellular immune deficiency, leukemia, lymphoma, myeloma, oral glucocorticoids, other immunosuppressant drugs and inhaled glucocorticoids. | | |

**References**

1. Sopori M. Effects of cigarette smoke on the immune system. Nat Rev Immunol **2002**; 2:372–377.

2. Nelson S, Kolls JK. Alcohol, host defence and society. Nat Rev Immunol **2002**; 2:205–209.

3. White IR, Royston P, Wood AM. Multiple imputation using chained equations: Issues and guidance for practice. Stat Med **2011**; 30:377–399.
