# Supplementary material for: Partner Bereavement and Risk of Herpes Zoster: Results from Two Population-Based Case-Control Studies in Denmark and the United Kingdom
Source: Clin Infect Dis. 2016 Dec 15;64(5):572–9. doi: 10.1093/cid/ciw840 (PMC5850543; doi:10.1093/cid/ciw840)
Supplement: Supplementary_Appendix_2 [file ciw840_suppl_supplementary_appendix_2.doc]

**Supplementary Appendix 2. Variable definitions**

The exposure definitions are described in detail in Table 2a below, including the definitions for risk of partner’s death. The Danish Civil Registration System contains exact addresses and kinship status (the unique civil personal register number of parents, siblings and children) for the entire population. This information made it possible to deduce with greater precision the relationship between persons living in the same household, compared to the UK study, which had only information on the age and sex of persons sharing a family practice number. Because of the more detailed data available in Denmark, we were more confident in using an algorithm allowing an age difference of up to 15 years between persons, whereas we included persons with a 10-year age difference or less in the UK. We did not consider consultations for partner bereavement, because such records may depend on health seeking behavior. Among those identified as bereaved by our algorithm in the UK study, only 10% had a recorded consultation for partner bereavement within the subsequent year, suggesting that bereavement codes were too incomplete to meet our study objective. Furthermore, we assumed that basing the timing of partner death on consultations for partner bereavement would be less accurate than that derived from our algorithm.

We used previously described methods to identify risk factors for herpes zoster [1], with minor modifications. We considered records of rheumatoid arthritis, systemic/subacute lupus erythematosus, inflammatory bowel disease, chronic obstructive pulmonary disease, asthma, diabetes (type I, type II or unknown type), chronic kidney disease, human immunodeficiency virus infection, hematopoietic stem cell or bone marrow transplantation, solid organ transplantation or other cellular immune deficiency (e.g.*,* primary immunodeficiency) ever before the index date; any record of leukemia, lymphoma or myeloma within two years before the index date; and any prescription records of oral glucocorticoids, other immunosuppressant drugs (e.g.*,* methotrexate or chemotherapy) or inhaled glucocorticoids within 90 days before the index date. The definition of chronic kidney disease included codes for chronic kidney disease stage 3 or higher, renal failure, chronic uremia, dialysis or renal transplantation. Solid organ transplantation included other types of organ transplants (not renal transplants). For chronic obstructive pulmonary disease, we included diagnoses of chronic bronchitis and emphysema and required that patients were aged 35 years or older at first diagnosis

[2]. To capture active asthma, we required that patients with a record of asthma also had an asthma-related prescription within the year before index date. Asthma patients were also required not to be classified as having chronic obstructive pulmonary disease. In the UK study, we defined any history of diabetes as (1) a definite diabetes diagnosis, (2) a possible diagnosis if followed by subsequent antidiabetic prescription or (3) two or more antidiabetic prescriptions (except women treated with metformin alone at age 20 to 39 years, as that may represent treatment of polycystic ovarian syndrome). In the Danish study, we used the Danish National Diabetes Registry, which uses a similar algorithm (see S1 Appendix). Because the Danish National Diabetes Registry does not differentiate between types of diabetes [3] and due to difficulties in identifying the type of diabetes using diagnosis codes in primary care

[4], we adjusted for any type of diabetes in analyses. However, for descriptive purposes, we aimed to classify the type of diabetes as type I, type II, or unknown based on information on age at first record, age at first treatment and type of treatment, as in previous CPRD studies

[1,5]. We defined type I diabetes as (1) age at first diagnosis ≤35 years and exclusive treatment with insulin prior to index date or (2) ≥2 insulin prescriptions at age ≤35 years but no diabetes diagnosis. Type II diabetes was defined as (1) age at first diabetes diagnosis >35 years or (2) exclusive treatment with oral anti-diabetics at age >35 years. We classified remaining patients with diabetes as having unknown type.

We also identified recent diagnoses of depression or anxiety. Previous studies demonstrate an increase in the use of symptoms of depression rather than diagnoses in electronic health records from primary care following the introduction of the Quality Outcomes Framework [6]. To accommodate this trend, we included both diagnoses and symptoms of depression from the Clinical Practice Research Datalink. In the Danish data, we supplemented with prescription records for antidepressants to capture conditions treated in general practice

[7]. We excluded tricyclic antidepressant prescriptions, because they are also used to treat neuralgia and insomnia.

We included data on patient-level socioeconomic status, measured as highest achieved education in Denmark (short [≤10 years], medium [>10–15 years] or long [>15 years]) and quintiles of the Index of Multiple Deprivation score in the UK.

We used diagnosis codes as well the additional details file in the Clinical Practice Research Datalink to retrieve data on smoking status (current smoker, ex-smoker, non-smoker) and alcohol consumption (current drinker, ex-drinker, non-drinker). We used only the additional details file for the calculation of body mass index (BMI), as medical Read codes are rarely used to record this information. Per convention, we categorized BMI according to the World Health Organization’s classification as underweight (<18.5 kg/m^2^), normal weight (18.5–24.9 kg/m^2^), overweight (25–29.9 kg/m^2^), obese (≥30 kg/m^2^) [8]. The categorization of the lifestyle variables was pragmatically based on status recorded closest to the index date. When possible, we used the nearest record within –1 year to +1 month, +1 months to +1 years, before –1 year, or within +1 year from index date, listed in the order of priority [1].

The data sources used to define each variable are shown in Table 2b. Codes used to identify herpes zoster cases are shown in Table 2c. Code lists for remaining study variables are available in the study protocols (see Supplementary Appendices 4 and 5).

**Table 2a. Exposure definitions used in Danish and UK studies on the association between partner bereavement and herpes zoster**

|  | **Denmark** | **The UK** |
| --- | --- | --- |
| **Partners** | 1. Married persons 2. Persons in a registered partnership 3. Cohabitating persons, definition 1:  - Exact same address - ≥1 cohabitating common child  1. Cohabitating persons, definition 2:  - Exact same address - No cohabitating common children (except step children) - Opposite sex - Age difference of <15 years - Not closely related based on patient identifiers for children and parents - No other adults living on the same address | Persons who fulfil all the following criteria:   - Same family practice number (people living in the same household or who are otherwise associated) - Opposite sex - Age difference of ≤10 years - No person in the household within 15 years of either of the couple   Except if ≥1 of the following:   - Case/control has a code indicating residence in a communal establishment before the index date - Both persons in the couple are aged ≥95 years - The family practice number is used for >10 persons |
| **Death** | Date of partner death in the Civil Registration System | Date of partner death in the Clinical Practice Research Datalink |
| **Age-adjusted Charlson Comorbidity Index** | Records in the Danish National Patient Registry and the Danish National Diabetes Registry at 1 month before the date of death | Records in the Clinical Practice Research Datalink and the Hospital Episodes Statistics database at 1 month before the date of death |
| **Terminal disease** | Not available | Records for e.g. delivery of end of life care or terminal illness in the Clinical Practice Research Datalink and the Hospital Episodes Statistics database |

**Table 2b. Overview of data sources used to define patient characteristics**

| **Variable** | **Denmark** | **The UK** |
| --- | --- | --- |
| Rheumatoid arthritis | ICD-8 and ICD-10 codes in the Patient Registry | Read codes in CPRD;  ICD-10 codes in HES |
| Systemic/subacute lupus erythematosus | ICD-8 and ICD-10 codes in the Patient Registry | Read codes in CPRD;  ICD-10 codes in HES |
| Inflammatory bowel disease | ICD-8 and ICD-10 codes in the Patient Registry | Read codes in CPRD;  ICD-10 codes in HES |
| Chronic obstructive pulmonary disease | ICD-8 and ICD-10 codes in the Patient Registry | Read codes in CPRD;  ICD-10 codes in HES |
| Asthma | ICD-8 and ICD-10 codes in the Patient Registry and ATC codes in the Prescription Registry | Read and product codes in CPRD;  ICD-10 codes in HES |
| Diabetes | Inclusion in the National Diabetes Registry; ICD-8 and ICD-10 codes in the Patient Registry and ATC codes in the Prescription Registry for subtyping | Read and product codes in CPRD;  ICD-10 codes in HES |
| Chronic kidney disease | ICD-8 and ICD-10, surgery and treatment codes in the Patient Registry | Read codes in CPRD;  ICD-10 and OPCS codes in HES |
| Human immunodeficiency virus infection | ICD-8 and ICD-10 codes in the Patient Registry | Read codes in CPRD;  ICD-10 codes in HES |
| Hematopoietic stem cell or bone marrow transplantation | ICD-10 codes and treatment codes in the Patient Registry | Read codes in CPRD;  ICD-10 and OPCS codes in HES |
| Solid organ transplantation | ICD-8 and ICD-10 codes and surgery codes in the Patient Registry | Read codes in CPRD;  ICD-10 and OPCS codes in HES |
| Other cellular immune deficiency | ICD-8 and ICD-10 codes in the Patient Registry | Read codes in CPRD;  ICD-10 codes in HES |
| Leukemia | ICD-8 and ICD-10 codes in the Patient Registry | Read codes in CPRD;  ICD-10 codes in HES |
| Lymphoma | ICD-8 and ICD-10 codes in the Patient Registry | Read codes in CPRD;  ICD-10 codes in HES |
| Myeloma | ICD-8 and ICD-10 codes in the Patient Registry | Read codes in CPRD;  ICD-10 codes in HES |
| Oral glucocorticoids | ATC codes in the Prescription Registry | CPRD product codes |
| Other immunosuppressant drugs | Treatment codes in the Patient Registry; ATC codes in the Prescription Registry | Read codes in CPRD;  ICD-10 and OPCS codes in HES |
| Inhaled glucocorticoids | ATC codes in the Prescription Registry | CPRD product codes |
| Depression and anxiety | ICD-8 and ICD-10 codes in the Patient Registry and the Psychiatric Central Registry; ATC codes in Prescription Registry | Read codes in CPRD;  ICD-10 codes in HES |
| Socioeconomic status | Population Education Registry | IMD 2010, providing data on both individual and practice level |
| Smoking status | Not available | Read codes and additional file data (entity type 4) in CPRD;  ICD-10 codes in codes in HES |
| Alcohol consumption | Not available | Read codes and additional file data (entity type 5) in CPRD;  ICD-10 codes in HES |
| Body mass index | Not available | Additional file data (entity types 104 and 140) in CPRD |

Abbreviations: ATC = Anatomical Therapeutic Chemical; CPRD = Clinical Practice Research Datalink; HES = Hospital Episode Statistics Database; ICD = International Classification of Diseases

**Table 2c. Codes used to identify herpes zoster cases. All lower level codes are included unless stated otherwise**

| **Denmark** |  |
| --- | --- |
| Hospital diagnoses of herpes zoster | ICD-8: 053; ICD-10: B02 (except B022), G051I, G051M, H031F, H131M, H192D, H192J, H220C, H621B |
| Antiviral treatment in general practice |  |
| Acyclovir | ATC code: J05AB01; Zoster-specific doses identified by Nordic article numbers 005404, 007109, 044597, 057554, 078015, 082158, 106864, 397653, 434183, 447144, 470021, 480533, 496455, 515258, 516328, and 560359 |
| Valacyclovir | ATC code: J05AB011; Zoster-specific doses identified by excluding prescriptions with Nordic article number 030449, 172940, 447695, 498063, or 534343 |
| Famciclovir | ATC code: J05AB09; Zoster-specific doses identified by Nordic article numbers 088196, 455584, 494756, and 550906 |
| Hospital diagnoses of post-herpetic neuralgia | ICD-8: no code available; ICD-10: G530, B022 |
| **The UK** |  |
| Hospital diagnoses of herpes zoster | ICD-10: B02 (except B022) |
| Herpes zoster in general practice | Medical codes: 390, 516, 7331, 8936, 14718, 14793, 18918, 21069, 21471, 25320, 27403, 27546, 31681, 33810, 38531, 39692, 43235, 44944, 47375, 50537, 51692, 52126, 52319, 55940, 57895, 62558, 63739, 69405, 70197, 71464, 105157 |
| Hospital diagnoses of post-herpetic neuralgia | ICD-10: G530, B022 |
| Post-herpetic neuralgia in general practice | Medical codes: 1598, 7584, 10223, 11498, 17180, 31709 |

Abbreviations: ATC = Anatomical Therapeutic Chemical; ICD = International Classification of Diseases

**References**

1. Forbes HJ, Bhaskaran K, Thomas SL, Smeeth L, Clayton T, Langan SM. Quantification of risk factors for herpes zoster: population based case-control study. BMJ **2014**; 348:g2911.

2. Quint JK, Müllerova H, DiSantostefano RL, et al. Validation of chronic obstructive pulmonary disease recording in the Clinical Practice Research Datalink (CPRD-GOLD). BMJ Open **2014**; 4:e005540.

3. Carstensen B, Kristensen JK, Marcussen MM, Borch-Johnsen K. The National Diabetes Register. Scand J Public Health **2011**; 39:58–61.

4. Royal College of General Practitioners, *Coding, Classification and Diagnosis of*

*Diabetes*, NHS, Editor. 2011.

5. Mulnier HE, Seaman HE, Raleigh VS, Soedamah-Muthu SS, Colhoun HM, Lawrenson RA. Mortality in people with type 2 diabetes in the UK. Diabet. Med. **2006**; 23:516–521.

6. Rait G, Walters K, Griffin M, Buszewicz M, Petersen I, Nazareth I. Recent trends in the incidence of recorded depression in primary care. Br J Psychiatry **2009**; 195:520–524.

7. Katon W, Pedersen HS, Ribe AR, et al. Effect of depression and diabetes mellitus on the risk for dementia: a national population-based cohort study. JAMA Psychiatry **2015**; 72:612–619.

8. WHO. Obesity: preventing and managing the global epidemic. Report of a WHO Consultation. WHO Technical Report Series 894. Geneva: World Health Organization, 2000.
