# Supplementary material for: Partner Bereavement and Risk of Herpes Zoster: Results from Two Population-Based Case-Control Studies in Denmark and the United Kingdom
Source: Clin Infect Dis. 2016 Dec 15;64(5):572–9. doi: 10.1093/cid/ciw840 (PMC5850543; doi:10.1093/cid/ciw840)
Supplement: Supplementary_Appendix_5 [file ciw840_suppl_supplementary_appendix_5.pdf]

Psychological stress and risk of herpes zoster

Sigrún Alba Jóhannesdóttir Schmidt
Department of Clinical Epidemiology
Aarhus University Hospital
Olof Palmes Allé 43-45
8200 Aarhus N
Denmark
E-mail: saj@clin.au.dk

Sinéad Langan
Department of Non-communicable Disease Epidemiology
Faculty of Epidemiology & Population Health
London School of Hygiene and Tropical Medicine
Keppel Street
London WC1E 7HT
United Kingdom
E-mail: Sinead.Langan@lshtm.ac.uk

Harriet Forbes
Department of Non-communicable Disease Epidemiology
Faculty of Epidemiology & Population Health
London School of Hygiene and Tropical Medicine
Keppel Street
London WC1E 7HT
United Kingdom
E-mail: Harriet.Forbes@lshtm.ac.uk

Sara Thomas
Department of Infectious Disease Epidemiology
Faculty of Epidemiology & Population Health
London School of Hygiene and Tropical Medicine
Keppel Street
London WC1E 7HT
United Kingdom
E-mail: sara.thomas@lshtm.ac.uk

Liam Smeeth
Department of Non-communicable Disease Epidemiology
Faculty of Epidemiology & Population Health
London School of Hygiene and Tropical Medicine
Keppel Street
London WC1E 7HT
United Kingdom
E-mail: liam.smeeth@lshtm.ac.uk

Kathryn Mansfield
Department of Non-communicable Disease Epidemiology

Faculty of Epidemiology & Population Health
London School of Hygiene and Tropical Medicine
Keppel Street
London WC1E 7HT
United Kingdom
E-mail: kathryn.mansfield@lshtm.ac.uk

Henrik Toft Sørensen
Department of Clinical Epidemiology
Aarhus University Hospital
Olof Palmes Allé 43-45
8200 Aarhus N
Denmark
E-mail: hts@clin.au.dk

Mogens Vestergaard
Research Unit and Section for General Practice
Department of Public Health, Aarhus University
Bartolins Allé 2
8000 Aarhus C
Denmark
E-mail: mv@ph.au.dk

Henrik Søndergaard Pedersen
Research Unit and Section for General Practice
Department of Public Health, Aarhus University
Bartolins Allé 2
8000 Aarhus C
Denmark
E-mail: henrikp@ph.au.dk

Henrik Carl Schønheyder
Department of Clinical Microbiology and Department of Clinical Medicine
Aalborg University Hospital
Mølleparksvej 10
9000 Aalborg
Denmark
E-mail: hcs@rn.dk

A. Lay Summary (Max. 200 words)

Herpes zoster, also known as shingles, is a frequent skin infection caused by reactivation of varicella zoster virus (VZV), which lies latent in the sensory ganglia after the primary infection chickenpox. Reactivation of the virus occurs when the immunity towards VZV wanes below a certain critical level. An increased risk of herpes zoster is thus observed among patient with immunosuppression, including those with inherited immunodeficiency and immunosuppression induced by drugs (*e.g.* chemotherapy), radiotherapy, or other disease (*e.g.* human immunodeficiency virus [HIV] or cancer).

Psychological stress may result in suppression of the immune system and thus possibly an increased risk of herpes zoster. However, data from large epidemiological studies on psychological stressors as risk factors for herpes zoster are sparse and conflicting.

Using data from the UK Clinical Practice Research Datalink (CPRD) and Hospital Episode Statistics (HES), we will investigate whether individuals diagnosed with herpes zoster have more frequent history of psychological stress than individuals without zoster, including partner bereavement, depressive disorder, anxiety disorder, and stress or adjustment disorder. We will explore the impact of recentness and severity of stress. Data will be published together with results from parallel analyses conducted on Danish data. The study is a health research study.

B. Technical Summary (Max. 200 words)

We will use CPRD and HES data to conduct a case-control study of the association between psychological stress and the risk of herpes zoster.

We will identify all adults with a first-time diagnosis of herpes zoster between 2000 to 2014. We will use incidence density sampling to select four controls per case matched by age, sex and practice. The date of herpes zoster diagnosis will be considered the index date for cases and their matched controls.

We will identify history of partner bereavement, depressive disorder, anxiety disorder, and stress or adjustment disorder prior to index date. We will categorise bereavement according to whether the partner death was foreseen, as measured by the age-adjusted Charlson Comorbidity Index and codes indicating terminal disease. For the psychiatric diagnoses, we will categorise severity as severe (requiring inpatient admission), moderate (requiring referral to other mental health service) or mild (remaining patients) based on healthcare records within 90 days before index date.

Using conditional logistic regression, we will compute odds ratios with 99% confidence intervals for the association between each psychological stressor and herpes zoster adjusting for risk factors for herpes zoster. We will examine the robustness of our results through several sensitivity analyses.

C. Objectives, Specific Aims and Rationale

The objective of this study is to use data from CPRD and HES to examine the association between various psychological stressors and the risk of herpes zoster. Specifically, we will examine the risk

of herpes zoster following partner bereavement, depressive disorder, anxiety disorder, or stress or adjustment disorder, focusing on the recentness and severity of these stressors. The results from our study will contribute to the understanding of the epidemiology of herpes zoster and may confirm the presumed suppressive effect of psychological stress on varicella-zoster cell-mediated immunity.

D. Background

Herpes zoster, also known as shingles, is a frequent neurocutaneous infection caused by reactivation of VZV, which lies latent in the sensory ganglia after the primary infection chickenpox.[1] Reactivation occurs when the cell-mediated immunity towards VZV wanes below a certain critical level.[1] Accordingly, the risk of herpes zoster is increased among elderly because of immunosenescence, among patients with inherited cell-mediated immunodeficiency, and among patients with immunosuppression acquired by drugs (*e.g.* patients receiving corticosteroids or chemotherapy), radiotherapy, or the disease (*e.g.* HIV or haematological cancer).[1,2]

Psychological stress is associated with activation of the hypothalamic-pituitary-adrenal axis and thereby suppression of the immune system.[3,4] It is therefore possible that psychological stress increases the risk of herpes zoster. For example, it has been demonstrated that VZV-specific cell-mediated immunity is reduced in patients with major depression[5] and that it correlates negatively with the severity of depressive symptoms.[6] Furthermore, the immunological response to the zoster vaccine is reduced in patients with untreated depression compared with individuals treated with antidepressants and individuals without depression.[7] However, data from large epidemiological studies on the association between psychological stressors and risk of herpes zoster are sparse and conflicting.

Previous epidemiological studies on psychiatric disease and risk of herpes zoster have considered primarily depression without examining the association by timing and severity.[2,8-15] Studies on negative life events, such as partner bereavement, are also sparse[15-19] and interpretation is hampered by study limitations, such as potential selection bias (non-response and volunteer bias),[15,17-19] lack of interviewer blinding,[15,17] and small study size.[15,17-19] Furthermore, various exposure windows and definitions have been employed[15-19] and only one study performed detailed analyses according to time passed between the life event and diagnosis of herpes zoster.[16]

E. Study Type

The study is hypothesis testing. The null hypothesis is that psychological stress is not associated with an increased risk of being diagnosed with herpes zoster.

F. Study Design

We propose to conduct a matched case-control study using data from CPRD linked to HES. This design is efficient for examining the wide range of psychological stressors considered in this study.

G. Sample Size

Table 1 provides power calculations for selected psychological stressors, assuming a case: control ratio of 4, an alpha-level of 0.05, and inclusion of 144,000 incident cases of herpes zoster.[2] Stata 14 was used for all calculations.

Table 1. Power calculation for the proposed study

Exposure	Prevalence	Power to detect an OR of 1.1	Power to detect an OR of 1.2	Power to detect an OR of 1.3
Any depression diagnosis	4%[2]	100%	100%	100%
Referral to psychiatrist, any	5.1%[20]	100%	100%	100%
Referral to psychologist/psychiatrist, past year	1.1%[20]	93%	100%	100%
Spousal bereavement past 30 days	0.4%[21]	56%	98%	100%

H. Data Linkage Required (if applicable)

The CPRD data will be linked to inpatient HES data to obtain data on diagnoses of herpes zoster, exposures and covariates in the hospital-based setting. Identification of diagnoses in the hospital-based setting is important for completeness of variables and thus the study validity. Furthermore, we will obtain data on socioeconomic status (for the area of residence through linkage to the Index of Multiple Deprivation, version 2010. The HES data will be included in the main analysis to increase completeness of diagnoses and the accuracy of timing, although only 60% of patients from the CPRD have data available in the HES database. Socioeconomic status as measured by the Index of Multiple Deprivation will be included in the multivariable models in a sensitivity analysis restricting to patients without missing data on this variable (approximately 60%).

I. Study Population

We will use CPRD and HES to identify all patients with a record of herpes zoster between 1 January 2000 and 31 December 2014 (or 31 March 2014 if a more up to date version of HES does not become available during the study) and at least 12 months of follow-up before diagnosis. We will restrict to patients aged 18 years or older because of differences in the epidemiology of herpes zoster in children and adults. For inclusion of cases with hospital diagnosis of herpes zoster, we will only consider those with a first-listed (primary) diagnosis of herpes zoster. The date of diagnosis in the CPRD or admission in HES will be considered the index date. In case of diagnosis in both CPRD and HES, we will consider the earliest diagnosis. We will only consider patients with no previous codes for herpes zoster or postherpetic neuralgia in the CPRD or HES (Read codes or ICD-10 codes in any position). Thus, if the earliest mention of herpes zoster in the data is a secondary code in HES, we will exclude the patient from the study. We expect to identify approximately 144,000 incident cases of herpes zoster.[2] We will define herpes zoster according to a code list used in a previously published study on risk factors for herpes zoster conducted by our research group (Supplementary Appendix).[2] The list will be updated slightly to include new codes.

J. Selection of comparison group(s) or controls

We will use incidence density sampling to randomly select up to four controls per case matched by practice, sex and age (within 1 year). Controls have to be registered in the practice at the index date

of the case and for at least 12 months before and should have no history of herpes zoster or post-herpetic neuralgia. Matching by practice controls for practice-level socioeconomic status. Controls will be assigned an index date identical to that of their case. We will exclude inactive controls, defined as individuals with no contact with their practice (*i.e.* no consultation records of any kind, including repeat prescriptions, face to face consultations, and telephone consultations) from six months before to 12 months after the index date.

K. Exposures, Outcomes and Covariates

Definitions of exposures and covariates are described in the following. The definition of the outcome, herpes zoster, is described in section I. Preliminary code lists for exposures are provided in the Supplementary Appendix. When necessary, we will differentiate between codes of psychiatric disease that indicate definite, possible (e.g. "depression interim review") or non-specific/other diagnoses. Code lists for covariates will be based on modified versions of lists in Caliber[22] and the previous study on herpes zoster risk factors.[2]

Partner bereavement

We will define partner bereavement as the death of a cohabitee prior to the index date, as previously described by Shah *et al*.[23] Cohabitees will be identified through CPRD using the family practice number, which identifies people who are living in the same household or otherwise are associated (*e.g.* live in the same institution). To avoid including the death of children in families, we will restrict to couples of the opposite sex, with an age gap of 10 years or less and with no younger adult in the household within 15 years of either of the couple.[23] Also, we will exclude couples where 1) a patient has codes in the primary care record that indicate residence in a communal establishment before the index date and/or 2) both individuals in the couple are 95 years of age or older. Through comparison with contemporary national representative household surveys in England, it has been shown that among persons aged ≥60 years, couples of the opposite sex with an age difference of less than 10 years who live together, 99.4% identify themselves as married or cohabitating.[23] Because the validity of our algorithm is expected to be lower for younger persons, we will restrict the study population to persons aged 40 years or older. Also, we will stratify the results by age.

We will use the CPRD death date in the deceased partner's primary care record as the date of bereavement. In analyses, we will consider any prior bereavement as well as differentiate between whether partner bereavement occurred within 0–7 days, 8–14 days, 15–30 days, 31–90 days, 91–365 days, 366–1095 days or >1095 days before the index date. The reference group for the analyses will be persons who have not previously experienced partner death.

We will aim to explore whether the association between bereavement and herpes zoster depends on whether the death of partner was unforeseen. We will ascertain the primary care (CPRD) and hospital record (HES) for deceased partners at one month before their date of death and compute their age-adjusted Charlson Comorbidity Index (ACCI), which is widely used to predict mortality.[24] The original Charlson Comorbidity Index assigns 0 to 6 points to a range of chronic diseases according to their ability to predict death.[24] In the age-adjusted index, additional points are given according to age (1 point: 50–59 years; 2 point: 60–69 years; 3 points: 70–79 years; 4 points: 80–89 years; 5 points: 90–99 years).[24] Based on the total ACCI score, we will the group study participants

into three categories of risk of partner death: lowest risk (0–5 points), intermediate risk (6–8 points), or highest risk (9–16 points). The CCI has previously been used to estimate risk of death in studies of bereavement.[21,25] As the ACCI includes a wide range of diseases, which may not all help partners to predict the expectedness of death, we will perform analyses according to whether partners had a previous healthcare record of terminal disease (as indicated by *e.g.* stay at hospice or metastatic cancer).

Diagnoses or symptoms of psychiatric disease or stress

Using CPRD and HES data, we will identify any history of depressive disorder, anxiety disorder (phobic anxiety disorders and other anxiety disorders) or acute stress and adjustment disorder in the electronic medical record prior to the index date. Since 2004, there has been an increasing use of symptoms rather than diagnosis codes for depression in CPRD.[26] Thus, to increase the completeness of our codes lists, we will include both Read codes for diagnoses and symptoms of depression and anxiety disorders.

The reference group for all analyses will be persons with no record of any of the psychiatric diseases or symptoms prior to index date. We will examine the association by timing and severity of these diseases. Based on the most recent record of psychological stress prior to index date, we will define timing as current (≤90 days before index date), recent (>90 days to 365 days before index date), former (>365 days before index date), and no (no previous record) psychological distress. Furthermore, we will subdivide the current group into newly diagnosed (first-ever record 90 days or less before the index date) and chronic (others). We will categorise severity as severe (requiring inpatient admission for >1 day), moderate (requiring referral from general practice to other mental health service) or mild (remaining patients) based on healthcare records within 90 days before index date. For the definition of severe disease, we will only consider those admitted with one of the psychiatric diseases (depressive disorder, anxiety disorder, or acute stress and adjustment disorder) as primary diagnosis for more than one day.

Covariates

From CPRD and HES, we will retrieve data on potential risk factors prior to index date index date (as defined in the study by Forbes *et al.*)[2]: Rheumatoid arthritis; systemic lupus erythematosus; inflammatory bowel disease; chronic obstructive pulmonary disease; asthma; chronic kidney disease (including those with kidney transplant or kidney dialysis); diabetes (type I, type II, or missing); human immunodeficiency virus infection, haematopoietic stem cell transplantation, myeloma, or “other unspecified cellular immune deficiencies” (for example, pancytopenia); recent history (less than two years before index date) of leukaemia, lymphoma, or bone marrow transplant; use of oral corticosteroids, other immunosuppressive treatments or use of inhaled corticosteroids within prior three months; smoking status (current smoker, ex-smoker, non-smoker); alcohol consumption (current drinker, ex-drinker, non-drinker); and body mass index (underweight (<18.5 kg/m^2), normal weight (18.5–24.9 kg/m^2), overweight (25–29.9 kg/m^2), obese (≥30 kg/m^2)). Read codes for BMI category will not be used, because they are rarely recorded. The categorisation of lifestyle variables will be pragmatically based on status recorded closest to the index date, with records within -1 year to +1 month from index date regarded as the best, +1 months to +1 years

from index date being second best, the nearest before -1 year from index date as the third best, and within +1 year from index date being least best.[2]

L. Data/ Statistical analysis

We will keep the analysis of bereavement and the psychiatric diseases separate, as we intend to publish them separately (see section Q).

We will characterise the population by case-control status and exposures. We will use conditional logistic regression to compute unadjusted odds ratios, associating the various measures of psychological stress with herpes zoster. We will consider the psychiatric diseases both together and separately. Definitions and reference groups described in section K will be applied. We will use 99% confidence intervals to account for the multiple measures included and to reduce the chance of detecting clinically insignificant associations. Please see amendment regarding choise of precision by the end of the protocol.

Given the density case-control study design, the odds ratios provide an unbiased estimate of the incidence rate ratios.[27] To account for potential confounding, we will fit two multivariable conditional logistic regression models. The first model will include all the aforementioned pre-specified covariates except for lifestyle factors and the second model will additionally account for lifestyle factors. The second model will include only those with complete data for all variables (estimated 90% of cases and controls).[2] We will control for age, sex and practice-level socioeconomic status by conditioning on matching factors.

We will examine for presence of effect measure modification by stratifying the results for current psychological stress by categories of age (<50, 50–59, 60–69, and ≥70 years) and sex. For bereavement, we will use both a 30-day and 90-day exposure window in the stratified analyses. To examine whether any association between bereavement and herpes zoster is mediated through depression or anxiety, we will also stratify the results by recent diagnoses of these conditions.

To quantify the effects of the psychological stress factors in absolute terms, we will compute the age-specific rate of zoster for each factor by multiplying the age-specific effect estimates by the age-specific rate of zoster in the general population in 2010 (estimated from CPRD).

We will perform a number of sensitivity analyses to address potential limitations of our data:

1. We will repeat analyses for the association between bereavement and herpes zoster after restricting to persons with evidence of having a partner, to examine whether using only persons who have a partner in the reference group affects the results.
2. We will test the completeness of our bereavement algorithm by examining how many of the bereaved subjects are identified by using CPRD codes for partner bereavement. Bereavement codes will however not be used in the main analysis, because they may be coded differentially (depending on consulting behaviour) and because timing of partner death is assumed to be less accurate than that derived from our algorithm.
3. We will examine the impact of using various cut-offs between current and recent (7, 14, 15, 30, and 180 days) psychiatric stress instead of 90 days.
4. We will perform analyses separating definite (with and without symptoms included), possible and unspecific/other diagnoses of psychiatric disease.

5. Under the assumption that patients who receive pharmacological therapy are more likely to have severe depression, we will change the severity definition to include the following categories: very severe (requiring inpatient admission for >1 day), severe (written prescription for an antidepressant recorded within 2 weeks to 90 days prior to index date), moderate (requiring referral from general practice to other mental health service) or mild (remaining patients) based on healthcare records within 2 weeks to 90 days before index date. The 2-weekiSimilarly, we will perform analyses after excluding those with previous antidepressant prescription from the reference group for psychiatric diseases.
6. We will repeat analyses examining each association for persons with a record of that psychological stressor only (e.g. 'depression only'), as some persons may have overlapping conditions.
7. We will perform the analysis after multiple imputation of missing information on lifestyle factors using chained equations,[28] as described by Forbes *et al.*[2]
8. We will additionally adjust for Index of Multiple Deprivation score (categorised in quintiles) among the approximately 60% who have agreed their electronic medical records linked to other dataset.

All analyses will be performed using STATA statistical software package.

M. Plan for addressing confounding

We will use multivariable conditional regression to adjust for risk factors listed in section K and (through conditioning) for matching factors (age, sex and practice). These covariables were selected based on the literature.

N. Plan for addressing missing data

Please see section L.

O. Limitations of the study design, data sources and analytical methods

There are some potential limitations to the proposed study, which we discuss in the following.

Partner bereavement and psychiatric diseases may affect disease perception and health-seeking behaviour, and thus the chance of having herpes zoster diagnosed. For example, depressive patients may have a stronger perception of mild skin symptoms.[29] On the other hand, patients experiencing psychological stress may not have energy to seek treatment for minor health problems. It is therefore difficult to predict in which direction such bias would affect our results. We will partly account for health-seeking behaviour by including data on participants' socioeconomic status. Also, due to the painful symptoms, we believe that individuals with herpes zoster would present to their general practitioner.[30,31]

Some misclassification of partner bereavement is possible, as our definition of partners requires that couples are registered with the same general practice and have an age difference of 10 years or less. Also, the nature of the relationship may be misclassified, as some 'partners' may be otherwise

associated (*e.g.* friends). Finally, we could miss some partners if the family practice identifier is used inconsistently in some practices or if the partner of a case or control uses a different general practitioner, who does not report in the CPRD. However, we do not believe that this misclassification is differential. Thus, if we find an association between bereavement and herpes zoster, it cannot be explained by such misclassification. Also, the sensitivity analysis restricting to partners only would limit such bias.

Expectation of death is difficult to categorise using data from electronic medical records and the psychological distress associated with expected death from a spouse may vary depending on the type of chronic disease. As we rely completely on the age-adjusted Charlson Comorbidity Index and utilisation of health services, the effect of probability of death may represent a mixed group of patients. This mix could possibly attenuate any true difference between groups of expected and unexpected partner death. We will explore the effect of expected death in various sensitivity analyses, which may point to limitations in our main definition.

We may underestimate use of prescription medications, because the electronic records do not include prescriptions written by hospital physicians. However, prescription treatment initiated by specialists is typically repeated by the general practitioner, so this likely affects mainly first-time prescriptions. Also, written prescriptions do not necessarily correspond to use. Nonetheless, we believe this is a minor issue, as we are interested in using prescriptions as a proxy measure of underlying psychological stress rather than the effect of the drug in itself.

Although we will adjust for various comorbidities and lifestyle factors, we may have residual confounding as partners typically share health characteristics and psychological stress may be associated with unhealthy lifestyle.

Misclassification of herpes zoster is possible, as it is usually a clinical diagnosis. However, because other validation studies report a high positive predictive value of physician-diagnosed herpes zoster,[32-36] we believe that the impact of misclassification on our results will be small.

Finally, we will not include outpatient HES data and inpatient HES data and the Index of Multiple Deprivation scores are only available for approximately 60% of the study population. Because herpes zoster is treated primarily in general practice, this lack of data will have only minor effect on the study population sample. However, it may result in misclassification of exposure and residual confounding. The sensitivity analysis restricting to the study subjects with HES inpatient data and the Index of Multiple Deprivation scores may aid in uncovering such bias.

P. Patient or user group involvement (if applicable)

Patients have not been involved in the planning of the study to date. We aim to disseminate any important findings to the public and appropriate patient groups as part of the publication process.

Q. Plans for disseminating and communicating study results, including the presence or absence of any restrictions on the extent and timing of publication

We intend to publish our findings in a peer-reviewed journal and to present them at relevant scientific conferences. Parallel analyses will be conducted on nationwide Danish registry data. All

approvals have been obtained for conducting the Danish study. We aim to publish the results from UK and Denmark together in two papers on partner bereavement and psychiatric disease (remaining exposures) respectively. This distinction will be made because partner bereavement is considered a natural response to a life event, whereas the psychiatric diseases (e.g. depression) are considered pathological reactions. Necessary approvals for use of the Danish data have already been obtained.

Amendments

- Originally, we stated that we used 99% confidence intervals ”to account for the multiple measures included and to reduce the chance of detecting clinically insignificant associations” (L. Data/ Statistical analysis). However, we want to specify that in reality we consider the choice of precision arbitrary. We will base our interpretations on clinical significance of the point estimates rather than dichotomising to statistical significance according to an arbitrary significance level.[37]
- Final code lists used are attached as an appendix.

R. References

1 Wilson JF. Herpes zoster. *Ann Intern Med* 2011; **154**: ITC31–15.

2 Forbes HJ, Bhaskaran K, Thomas SL, Smeeth L, Clayton T, Langan SM. Quantification of risk factors for herpes zoster: population based case-control study. *BMJ* 2014; **348**: g2911.

3 Reiche EMV, Nunes SOV, Morimoto HK. Stress, depression, the immune system, and cancer. *Lancet Oncol* 2004; **5**: 617–25.

4 Glaser R, Kiecolt-Glaser JK. Stress-induced immune dysfunction: implications for health. *Nat Rev Immunol* 2005; **5**: 243–51.

5 Irwin M, Costlow C, Williams H, *et al.* Cellular immunity to varicella-zoster virus in patients with major depression. *J Infect Dis* 1998; **178 Suppl 1**: S104–8.

6 Irwin MR, Levin MJ, Carrillo C, *et al.* Major depressive disorder and immunity to varicella-zoster virus in the elderly. *Brain Behav Immun* 2011; **25**: 759–66.

7 Irwin MR, Levin MJ, Laudenslager ML, *et al.* Varicella zoster virus-specific immune responses to a herpes zoster vaccine in elderly recipients with major depression and the impact of antidepressant medications. *Clin Infect Dis* 2013; **56**: 1085–93.

8 Hata A, Kuniyoshi M, Ohkusa Y. Risk of Herpes zoster in patients with underlying diseases: a retrospective hospital-based cohort study. *Infection* 2011; **39**: 537–44.

9 Joesoef RM, Harpaz R, Leung J, Bialek SR. Chronic medical conditions as risk factors for herpes zoster. *Mayo Clin Proc* 2012; **87**: 961–7.

10 Ogunjimi B, Buntinx F, Bartholomeeusen S, *et al.* Herpes zoster is associated with herpes simplex and other infections in under 60 year-olds. *J Infect* 2015; **70**: 171–7.

11 Hoover DR, Cohen M, Mutimura E, Cohen HW, Anastos K. Prevalence of shingles and association with PTSD and depression among HIV infected women in Rwanda. *Translational Science 2012 Meeting abstracts* 2012; : 1–70.

12 Temmerman G, Van Nimmen L. Investigation of zona (Herpes zoster) in 1966 by the research group of the general practitioners of Northern Belgium. Results and conclusions. Tijdschrift voor Geneeskunde. 1974; **30**: 411–26.

13 Tseng HF, Smith N, Marcy SM, Sy LS, Chao CR, Jacobsen SJ. Risk factors of herpes zoster among children immunized with varicella vaccine: results from a nested case-control study. *Pediatr Infect Dis J* 2010; **29**: 205–8.

14 Yang Y-W, Chen YH, Lin H-W. Risk of herpes zoster among patients with psychiatric diseases: a population-based study. *J Eur Acad Dermatol Venereol* 2011; **25**: 447–53.

15 Lasserre A, Blaizeau F, Gorwood P, *et al.* Herpes zoster: family history and psychological stress-case-control study. *J Clin Virol* 2012; **55**: 153–7.

16 Harpaz R, Leung JW, Brown CJ, Zhou FJ. Psychological stress as a trigger for herpes zoster: might the conventional wisdom be wrong? *Clin Infect Dis* 2015; **60**: 781–5.

17 Schmader K, Studenski S, MacMillan J, Grufferman S, Cohen HJ. Are stressful life events risk factors for herpes zoster? *J Am Geriatr Soc* 1990; **38**: 1188–94.

18 Schmader K, George LK, Burchett BM, Hamilton JD, Pieper CF. Race and stress in the incidence of herpes zoster in older adults. *J Am Geriatr Soc* 1998; **46**: 973–7.

19 Schmader K, George LK, Burchett BM, Pieper CF. Racial and psychosocial risk factors for herpes zoster in the elderly. *J Infect Dis* 1998; **178 Suppl 1**: S67–70.

20 Spoendlin J, Bichsel F, Voegel JJ, Jick SS, Meier CR. The association between psychiatric diseases, psychotropic drugs and the risk of incident rosacea. *Br J Dermatol* 2014; **170**: 878–83.

21 Graff S, Christensen B, Fenger-Grøn M, *et al.* Long-term risk of atrial fibrillation after the death of a spouse: A nationwide population-based case-control study.

22 Denaxas SC, George J, Herrett E, *et al.* Data resource profile: cardiovascular disease research using linked bespoke studies and electronic health records (CALIBER). *Int J Epidemiol* 2012; **41**: 1625–38.

23 Shah SM, Carey IM, Harris T, DeWilde S, Victor CR, Cook DG. Do good health and material circumstances protect older people from the increased risk of death after bereavement? *Am J Epidemiol* 2012; **176**: 689–98.

24 Charlson M, Szatrowski TP, Peterson J, Gold J. Validation of a combined comorbidity index. *J Clin Epidemiol* 1994; **47**: 1245–51.

25 Shah SM, Carey IM, Harris T, DeWilde S, Victor CR, Cook DG. The effect of unexpected bereavement on mortality in older couples. *Am J Public Health* 2013; **103**: 1140–5.

26 Rait G, Walters K, Griffin M, Buszewicz M, Petersen I, Nazareth I. Recent trends in the incidence of recorded depression in primary care. *Br J Psychiatry* 2009; **195**: 520–4.

27 Pearce N. What does the odds ratio estimate in a case-control study? *Int J Epidemiol* 1993; **22**: 1189–92.

28 White IR, Royston P, Wood AM. Multiple imputation using chained equations: Issues and guidance for practice. *Stat Med* 2011.

29 Abram K, Silm H, Maaroos H-I, Oona M. Subjective disease perception and symptoms of depression in relation to healthcare-seeking behaviour in patients with rosacea. *Acta Derm Venerol* 2008; **89**: 488–91.

30 Lu P-J, Euler GL, Jumaan AO, Harpaz R. Herpes zoster vaccination among adults aged 60 years or older in the United States, 2007: uptake of the first new vaccine to target seniors. *Vaccine* 2009; **27**: 882–7.

31 Østergaard K, Damgaard M, Kristiansen TB, Madsen KG. Herpes zoster incidence in persons above 50 years of age (article in Danish). *Ugeskr Laeg* 2009; **171**: 2194–7.

32 Oxman MN, Levin M, Johnson GR, *et al.* A vaccine to prevent herpes zoster and postherpetic neuralgia in older adults. *N Engl J Med* 2005; **352**: 2271–84.

33 Rübben A, Baron JM, Grussendorf-Conen EI. Routine detection of herpes simplex virus and varicella zoster virus by polymerase chain reaction reveals that initial herpes zoster is frequently misdiagnosed as herpes simplex. *Br J Dermatol* 1997; **137**: 259–61.

34 Scott FT, Johnson RW, Leedham-Green M, Davies E, Edmunds WJ, Breuer J. The burden of Herpes Zoster: a prospective population based study. *Vaccine* 2006; **24**: 1308–14.

35 Weinmann S, Chun C, Schmid DS, *et al.* Incidence and clinical characteristics of herpes zoster among children in the varicella vaccine era, 2005-2009. *J Infect Dis* 2013; **208**: 1859–68.

36 Rimland D, Moanna A. Increasing incidence of herpes zoster among Veterans. *Clin Infect Dis* 2010; **50**: 1000–5.

37 Rothman KJ. Six persistent research misconceptions. *J Gen Intern Med* 2014; **29**: 1060–4.

Supplementary appendix

Table 1. *Preliminary* list of CPRD codes

Medcode	Readterm	Group (if applicable)*
Herpes zoster		
390	Herpes zoster	
516	Shingles	
7331	Ramsey Hunt Syndrome	
8936	Ophthalmic herpes zoster infection	
14718	Herpes zoster with ophthalmic complication	
14793	Herpes zoster otitis externa	
18918	Herpes zoster ophthalmicus	
21069	Herpes zoster with unspecified complication	
21471	Herpes zoster NOS	
25320	Herpes zoster with dermatitis of eyelid	
27403	Geniculate herpes zoster	
27546	Herpes zoster with keratoconjunctivitis	
31681	Herpes zoster - otitis externa	
33810	Herpes zoster with other ophthalmic complication	
38531	Herpes zoster with other specified complication NOS	
39692	Polyneuropathy in herpes zoster	
43235	Herpes zoster with other specified complication	
44944	Herpes zoster with meningitis	
47375	Zoster encephalitis	
50537	Herpes zoster with other CNS complications	
51692	Encephalitis due to herpes zoster	
52126	Herpes zoster with other central nervous system complication	
52319	Disseminated zoster	
55940	Herpes zoster iridocyclitis	
57895	Herpes zoster meningitis	
62558	Infective otitis externa due to herpes zoster	
63739	Herpes zoster with other CNS complication NOS	
69405	Herpes zoster encephalitis	
70197	[X]Zoster without complications	
71464	Meningitis due to herpes zoster virus	

Chronic zoster complications		
1598	Post-herpetic neuralgia	
7584	Post-herpetic trigeminal neuralgia	
10223	Postherpetic neuralgia	
11498	Postherpetic trigeminal neuralgia	

17180	Postzoster neuralgia	
31709	Postherpetic polyneuropathy	

Residence in a communal establishment (for partner definition)		
21280	Resident in part III accomodation	
36096	Part 3 accomodation	
27425	Part III accommodation	
13360	Nursing/other home	
13359	Lives in a nursing home	
11419	Lives in an old peoples home	
15840	Lives in a welfare home	
27968	Residential institution	
24956	Lives in a residential home	
59548	Lives in an old peoples home	
68005	Lives in a welfare home	
49681	Lives in care home	

Partner bereavement (for sensitivity analysis)		
207	Death of spouse	
11251	Death of wife	
12076	Husband died	
12325	Death of husband	
17802	Husband died	
25097	Death of husband	
28440	Death of partner	
36947	Partner dying	

Depression		
324	Depressive disorder NEC	Definite (1)
543	[X]Depression NOS	Definite (1)
595	Endogenous depression	Definite (1)
1055	Agitated depression	Definite (1)
1908	O/E - depressed	Definite (1)
1996	Depressed	Definite (1)
2147	Poor self esteem	Definite (1)
2560	Depressive psychoses	Definite (1)
2930	C/O - feeling unhappy	Definite (1)
2970	[X]Depressive episode, unspecified	Definite (1)
3291	[X]Depressive disorder NOS	Definite (1)
3292	[X]Recurrent depressive disorder	Definite (1)
4323	Chronic depression	Definite (1)
4639	[X]Depressive episode	Definite (1)
4824	C/O - feeling depressed	Definite (1)

5879	Agitated depression	Definite (1)
6482	Recurrent depression	Definite (1)
6546	Endogenous depression first episode	Definite (1)
6854	[X]Other depressive episodes	Definite (1)
6932	Endogenous depression - recurrent	Definite (1)
6950	Endogenous depression first episode	Definite (1)
7011	Single major depressive episode NOS	Definite (1)
7412	Loss of confidence	Definite (1)
8826	[X]SAD - Seasonal affective disorder	Definite (1)
8928	Low mood	Definite (1)
9183	Masked depression	Definite (1)
9211	[X]Moderate depressive episode	Definite (1)
9667	[X]Severe depressive episode without psychotic symptoms	Definite (1)
9796	Symptoms of depression	Definite (1)
10015	Depressed mood	Definite (1)
10438	Depressive symptoms	Definite (1)
10610	Single major depressive episode	Definite (1)
10667	[X]Mild depression	Definite (1)
10720	[X]Atypical depression	Definite (1)
10825	Seasonal affective disorder	Definite (1)
11252	[X]Major depression, recurrent without psychotic symptoms	Definite (1)
11329	[X]Endogenous depression without psychotic symptoms	Definite (1)
11717	[X]Mild depressive episode	Definite (1)
12099	[X]Severe depressive episode with psychotic symptoms	Definite (1)
14709	Recurrent major depressive episodes, moderate	Definite (1)
15099	Recurrent major depressive episode	Definite (1)
15155	Single major depressive episode, moderate	Definite (1)
15219	Single major depressive episode, severe, without psychosis	Definite (1)
16506	Single major depressive episode, mild	Definite (1)
16861	[X]Recurrent severe episodes of psychotic depression	Definite (1)
18510	[X]Single episode of psychogenic depression	Definite (1)
19696	[X]Recurrent episodes of psychogenic depression	Definite (1)
22806	[X]Single episode major depression w'out psychotic symptoms	Definite (1)
23731	[X]Endogenous depression with psychotic symptoms	Definite (1)
24112	[X]Single episode of psychotic depression	Definite (1)
24117	[X]Single episode of major depression and psychotic symptoms	Definite (1)
24171	Recurrent major depressive episodes, severe, with psychosis	Definite (1)
25435	Loss of capacity for enjoyment	Definite (1)
25563	Recurrent major depressive episode NOS	Definite (1)
25697	Recurrent major depressive episodes, severe, no psychosis	Definite (1)
26028	Sad mood	Definite (1)
27491	Atypical depressive disorder	Definite (1)
28756	[X]Seasonal depressive disorder	Definite (1)

29342	Recurrent major depressive episodes, mild	Definite (1)
29520	[X]Recurrent depressive disorder, current episode moderate	Definite (1)
29784	[X]Recurrent depressive disorder, current episode mild	Definite (1)
30740	Loss of interest	Definite (1)
31757	[X]Recurr severe episodes/psychogenic depressive psychosis	Definite (1)
32159	Single major depressive episode, severe, with psychosis	Definite (1)
32941	[X]Recurr severe episodes/major depression+psychotic symptom	Definite (1)
33469	[X]Recurr depress disorder cur epi severe without psyc sympt	Definite (1)
34390	Single major depressive episode, unspecified	Definite (1)
35671	Recurrent major depressive episodes, unspecified	Definite (1)
36616	[X]Monopolar depression NOS	Definite (1)
41989	[X]Single episode agitated depressn w'out psychotic symptoms	Definite (1)
44300	[X]Recurrent depressive disorder, unspecified	Definite (1)
47009	[X]Recurrent depress disorder cur epi severe with psyc symp	Definite (1)
47731	[X]Other recurrent depressive disorders	Definite (1)
52678	[X]Single episode of psychogenic depressive psychosis	Definite (1)
53148	Loss of hope for the future	Definite (1)
56609	[X]Single episode of masked depression NOS	Definite (1)
59386	[X]Single episode vital depression w'out psychotic symptoms	Definite (1)
59869	Loss of interest in previously enjoyable activity	Definite (1)
73991	[X]Vital depression, recurrent without psychotic symptoms	Definite (1)
98252	[X]Major depression, moderately severe	Definite (1)
98346	[X]Major depression, mild	Definite (1)
98414	[X]Major depression, severe without psychotic symptoms	Definite (1)
98417	[X]Major depression, severe with psychotic symptoms	Definite (1)
5987	[X] Reactive depression NOS	Definite (1)
7604	[X]Single episode of reactive depression	Definite (1)
8478	Reactive depressive psychosis	Definite (1)
8851	[X]Recurrent episodes of depressive reaction	Definite (1)
8902	[X]Recurrent episodes of reactive depression	Definite (1)
9055	[X]Single episode of depressive reaction	Definite (1)
17770	Psychotic reactive depression	Definite (1)
37764	[X]Recurrent severe episodes/reactive depressive psychosis	Definite (1)
101422	Feeling low or worried	Definite (1)
12122	Depression medication review	Possible depression (2)
12399	Depression annual review	Possible depression (2)
30405	Depression interim review	Possible depression (2)
44848	Depression management programme	Possible depression (2)
30583	Depression - enhanced services administration	Possible depression (2)
42931	On depression register	Possible depression (2)
48970	Exception reporting: depression quality indicators	Possible depression (2)
51258	Depression monitoring administration	Possible depression (2)

65435	Depression - enhanced service completed	Possible depression (2)
71009	Depression monitoring first letter	Possible depression (2)
72966	Depression monitoring second letter	Possible depression (2)
85852	Depression monitoring telephone invite	Possible depression (2)
88644	Depression monitoring verbal invite	Possible depression (2)
91105	Depression monitoring third letter	Possible depression (2)
96995	On full dose long term treatment depression - enh serv admin	Possible depression (2)
28970	Excepted from depression quality indicators: Patient unsuita	Possible depression (2)
30405	Depression interim review	Possible depression (2)
30483	Patient given advice about management of depression	Possible depression (2)
100977	Suspected depression	Possible depression (2)
11055	[X]Schizoaffective disorder, depressive type	Non-specific/other (3)
20785	[X]Post-schizophrenic depression	Non-specific/other (3)
35274	[X]Schizoaffective psychosis, depressive type	Non-specific/other (3)
41022	[X]Schizophreniform psychosis, depressive type	Non-specific/other (3)
8584	[X]Depressive neurosis	Non-specific/other (3)
7737	[X]Neurotic depression	Non-specific/other (3)
7953	[X]Dysthymia	Non-specific/other (3)
10290	[X]Depressive personality disorder	Non-specific/other (3)
10455	Depressive personality disorder	Non-specific/other (3)
15220	[X]Persistant anxiety depression	Non-specific/other (3)
1131	Neurotic depression reactive type	Non-specific/other (3)
21887	Senile dementia with depression	Non-specific/other (3)
27677	Presenile dementia with depression	Non-specific/other (3)
27759	[X] Senile dementia, depressed or paranoid type	Non-specific/other (3)
41089	Senile dementia with depressive or paranoid features NOS	Non-specific/other (3)
43292	Arteriosclerotic dementia with depression	Non-specific/other (3)
44674	Senile dementia with depressive or paranoid features	Non-specific/other (3)
2972	Postviral depression	Non-specific/other (3)
29527	[D]Postoperative depression	Non-specific/other (3)
46244	Drug-induced depressive state	Non-specific/other (3)
2639	Postnatal depression	Non-specific/other (3)
103677	[X]Antenatal depression	Non-specific/other (3)
2923	Puerperal depression	Non-specific/other (3)
4979	[X]Postpartum depression NOS	Non-specific/other (3)
13307	[X]Postnatal depression NOS	Non-specific/other (3)
19054	[X]Recurrent brief depressive episodes	Non-specific/other (3)
55384	Recurrent major depressive episodes, in full remission	Non-specific/other (3)
56273	Recurrent major depressive episodes,partial/unspec remission	Non-specific/other (3)
22116	[X]Recurrent depressive disorder, currently in remission	Non-specific/other (3)
43324	Single major depressive episode, partial or unspec remission	Non-specific/other (3)
2716	H/O: depression	Non-specific/other (3)
19439	Depression resolved	Non-specific/other (3)
57409	Single major depressive episode, in full remission	Non-specific/other (3)

Phobic anxiety		
1723	Claustrophobia	Definite (1)
1907	Phobic disorders	Definite (1)
2300	Phobia unspecified	Definite (1)
2366	Dental phobia	Definite (1)
2571	[X]Agoraphobia	Definite (1)
3076	Agoraphobia with panic attacks	Definite (1)
7222	[X]Phobia NOS	Definite (1)
9386	[X]Phobic anxiety disorders	Definite (1)
9785	[X]Specific (isolated) phobias	Definite (1)
9944	Phobic anxiety	Definite (1)
11280	[X]Claustrophobia	Definite (1)
11602	[X]Social phobias	Definite (1)
12508	[X]Needle phobia	Definite (1)
12635	[X]Simple phobia	Definite (1)
12838	Agoraphobia without mention of panic attacks	Definite (1)
14729	Phobic disorder NOS	Definite (1)
14890	[X]Panic disorder with agoraphobia	Definite (1)
16199	Social phobia, fear of eating in public	Definite (1)
16638	Social phobic disorders	Definite (1)
16729	[X]Agoraphobia without history of panic disorder	Definite (1)
18248	[X]Animal phobias	Definite (1)
18603	Social phobia, fear of public washing	Definite (1)
20802	Flying phobia	Definite (1)
25749	Phobia counselling	Possible phobia (2)
27685	[X]Other phobic anxiety disorders	Definite (1)
28106	Acrophobia	Definite (1)
28938	Animal phobia	Definite (1)
31957	Social phobia, fear of public speaking	Definite (1)
42788	[X]Social neurosis	Definite (1)
34064	[X]Phobic anxiety disorder, unspecified	Definite (1)
42788	[X]Social neurosis	Definite (1)
63521	Antiphobic therapy	Possible phobia (2)
67898	[X]Phobic state NOS	Definite (1)
67965	[X]Acrophobia	Definite (1)
4167	Fear of flying	Definite (1)
5347	Fear	Definite (1)
10390	Fear of death	Definite (1)
18672	Specific fear	Definite (1)
26331	O/E - fearful mood	Definite (1)
28129	Examination fear	Definite (1)
31672	Fear of crowds	Definite (1)
53067	Encounter for fear	Definite (1)
3407	H/O: anxiety state	Non-specific/other (3)

Other anxiety disorder		
462	Panic attack	Definite (1)
636	Anxiety states	Definite (1)
655	Anxiety with depression	Definite (1)
962	[X]Anxiety neurosis	Definite (1)
1758	Chronic anxiety	Definite (1)
4069	Panic disorder	Definite (1)
4081	[X]Panic state	Definite (1)
4534	Anxiety state NOS	Definite (1)
4634	Recurrent anxiety	Definite (1)
4659	Generalised anxiety disorder	Definite (1)
5385	[X]Other anxiety disorders	Definite (1)
6408	[X]Panic attack	Definite (1)
6939	Anxiety state unspecified	Definite (1)
7749	[X]Mild anxiety depression	Definite (1)
7999	Anxiety counselling	Possible anxiety (2)
8205	[X]Panic disorder [episodic paroxysmal anxiety]	Definite (1)
9125	Anxiety management training	Possible anxiety (2)
10344	[X]Generalized anxiety disorder	Definite (1)
11913	[X]Mixed anxiety and depressive disorder	Definite (1)
22159	Acknowledging anxiety	Possible anxiety (2)
23838	[X]Anxiety disorder, unspecified	Definite (1)
24066	[X]Other specified anxiety disorders	Definite (1)
25638	[X]Anxiety NOS	Definite (1)
26295	Reducing anxiety	Possible anxiety (2)
28167	[X]Anxiety hysteria	Definite (1)
28381	Alleviating anxiety	Possible anxiety (2)
44321	[X]Other mixed anxiety disorders	Definite (1)
962	[X]Anxiety neurosis	Definite (1)
50191	[X]Anxiety state	Definite (1)
62935	Recognising anxiety	Possible anxiety (2)
131	Anxiousness	Definite (1)
514	Tension - nervous	Definite (1)
2509	[D]Nervousness	Definite (1)
2524	Worried	Definite (1)
3328	General nervous symptoms	Definite (1)
3586	nerves	Definite (1)
5902	Anxiousness - symptom	Definite (1)
10723	[D]Nervous tension	Definite (1)
11890	C/O - panic attack	Definite (1)
19000	O/E - panic attack	Definite (1)
20089	General nervous symptom NOS	Definite (1)
20163	Apprehension	Definite (1)
20375	[V]"Worried well"	Definite (1)

28408	Worried well	Definite (1)
29608	nervousness	Definite (1)
93401	Anxious	Definite (1)
35825	[X]Anxiety reaction	Definite (1)
17853	[D]Nerves	Definite (1)
8725	O/E - nervous	Definite (1)
13124	O/E - anxious	Definite (1)
19129	Frightened	Definite (1)
20773	[X]Organic anxiety disorder	Non-specific/other (3)
3407	H/O: anxiety state	Non-specific/other (3)

Severe stress and adjustment disorder		
276	Acute reaction to stress	
4171	[X]Post - traumatic stress disorder	
7813	[X]Psychic shock	
10535	[X]Acute reaction to stress	
11098	[X]Reaction to severe stress, and adjustment disorders	
11607	[X]Acute stress reaction	
15551	Acute stupor state due to acute stress reaction	
20245	Acute situational disturbance	
21559	[X]Crisis state	
21753	[X]Other reactions to severe stress	
23869	Stress reaction causing mixed disturbance of emotion/conduct	
24847	Acute posttrauma stress state	
26138	Acute stress reaction NOS	
29707	Other acute stress reaction NOS	
31515	[X]Reaction to severe stress, unspecified	
32182	[X]Traumatic neurosis	
32387	Other post-traumatic stress disorder	
36374	[X]Acute crisis reaction	
38640	Other acute stress reactions	
42737	Acute fugue state due to acute stress reaction	
43550	Combat fatigue	
70779	[X]Combat fatigue	
2826	Adjustment reaction	
6075	Adjustment reaction with aggression	
8250	[D]State of emotional shock and stress, unspecified	
11336	[X]Adjustment disorders	
15665	Adjustment reaction with disturbance of other emotion NOS	
16415	Adjustment reaction with predominant disturbance of conduct	
19921	Other adjustment reaction with withdrawal	
23462	Other adjustment reactions NOS	
27390	Adjustment reaction due to hospitalisation	

35632	Adjustment reaction with physical symptoms
35914	Adjustment reaction with antisocial behaviour
37669	Adjustment reaction NOS
41455	Other adjustment reactions
48588	Adjustment reaction with mixed disturbance of emotion
56020	[X]State of emotional shock and stress, unspecified
56924	Adjustment reaction with anxious mood
66398	Adjustment reaction with destructiveness
101725	[X]Chron post-traumatic stress disorder follow military comb
101785	[X]Acute post-traumatic stress disorder follow military comb
104891	Adjustment reaction with predominant disturbance conduct NOS
32182	[X]Traumatic neurosis
36228	[X]Culture shock
58013	Culture shock
2775	Grief reaction
21197	[X]Grief reaction
56924	Adjustment reaction with anxious mood
11940	Acute panic state due to acute stress reaction
1533	Brief depressive reaction
16632	Prolonged depressive reaction
28248	[X]Prolonged single episode of reactive depression
28863	[X]Single episode of reactive depressive psychosis
36246	Brief depressive reaction NOS

Referral to mental health service	
1690	Refer to psychologist
2189	Psychiatric referral
5338	Referral to psychogeriatrician
9868	Referral to psychiatric nurse
10002	Referral to psychiatrist
10236	Referral to community mental health team
10669	Referral to psychotherapist
10828	Referral to mental health counsellor
10967	Refer to psychologist
11270	Referral to psychologist
11449	Referral to mental health crisis team
11958	Referral to psychotherapist
12053	Referral to mental health team
12365	Referral to older age community mental health team
12447	Referral to primary care mental health gateway worker
13677	Private referral to psychiatrist
13703	Referral to non NHS mental health community service
18956	Referral to bereavement counsellor

24504	Refer to mental health worker
25950	Referral to clinical physiologist
26178	Referral to psychosis early intervention service
27640	Psychiatric self-referral
28925	Referral for guided self-help for anxiety
30861	Referral to nurse behavioural therapist
32841	Referral for guided self-help for depression
34532	Private referral to psychogeriatrician
34538	Referral to nurse psychotherapist
36728	Referral to liaison psychiatrist
40894	Referral to psychiatrist for mental handicap
41040	Referral to primary care mental health graduate worker
41071	Referral for mental health self-help literature
41267	Referral to complementary therapist
42932	Referral to bereavement counsellor
44910	Referral to rehabilitation psychiatrist
84212	Referral to primary care mental health team
95163	Emergency voluntary psychiatric admission Mental Health Act
95936	Urgent referral to psychiatrist
97420	Referral to improving access to psychological therapies prog
99630	Referral for cognitive behavioural therapy
100992	Improving Access to Psychological Therapies pro phobia scale
105244	Referral to IAPT (imp acc to psych thera) programme declined
105357	Private referral to psychologist
102636	Patient initiated encounter with mental health crisis team
10308	Under care of mental health team
108558	Under care of mental health in-reach team
32602	Under care of mental health counsellor
11425	Seen by mental health counsellor

BNF codes for antidepressant drugs (for sensitivity analysis)	
BNF section 4.3.2	Monoamine oxidase inhibitors
BNF section 4.3.4.	Other antidepressants
BNF section 4.3.3.	Serotonin re-uptake inhibitors (SSRIs) antidepressants
BNF section 4.3.1.	Tricyclic antidepressants (TCAs)

*We will not include psychiatric disorders limited to childhood. Organic depression/anxiety will be included in the ”non-specific/other” group, they are not classified with depression according to the ICD-10. Similarly, codes which are unspecific with regard to timing (codes indicating e.g. remission) will be categorised as non-specific/other. Furthermore, ”non-specific/other depression” includes also codes for schizoaffective disorder with depression, post-schizophrenic depression, drug-induced depression, depression in association with pregnancy, and dysthymia.

Table 2. *Preliminary* list of HES codes

ICD-10 code	Text descriptor	Group (if applicable)
Herpes zoster		
B02	Zoster [herpes zoster]	
Chronic zoster complications		
G530	Postzoster neuralgia	
Depression		
F320	Mild depressive episode	Definite (1)
F321	Moderate depressive episode	Definite (1)
F322	Severe depressive episode without psychotic symptoms	Definite (1)
F323	Severe depressive episode with psychotic symptoms	Definite (1)
F328	Other depressive episodes	Definite (1)
F329	Depressive episode, unspecified	Definite (1)
F331	Recurrent depressive disorder, current episode moderate	Definite (1)
F332	Recurrent depressive disorder, current episode severe without psychotic symptoms	Definite (1)
F333	Recurrent depressive disorder, current episode severe with psychotic symptoms	Definite (1)
F330	Recurrent depressive disorder, current episode mild	Definite (1)
F334	Recurrent depressive disorder, currently in remission	Non-specific/other (3)
F338	Other recurrent depressive disorders	Definite (1)
F339	Recurrent depressive disorder, unspecified	Definite (1)
F25	Schizoaffective disorder	Non-specific/other (3)
F250	Schizoaffective disorder, manic type	Non-specific/other (3)
F251	Schizoaffective disorder, depressive tyoe	Non-specific/other (3)
F252	Schizoaffective disorder, mixed manio-depressive type	Non-specific/other (3)
F258	Schizoaffective disorder, other type	Non-specific/other (3)
F259	Schizoaffective disorder, unspecified	Non-specific/other (3)
F204	Post-schizophrenic depression	Non-specific/other (3)
F0003	Dementia in early onset Alzheimers disease with depressive symptoms	Non-specific/other (3)
F0013	Dementia in late onset Alzheimers disease with depressive symptoms	Non-specific/other (3)
F0023	Dementia in atypical Alzheimers disease with depressive symptoms	Non-specific/other (3)
F0093	Dementia in Alzheimers disease with depressive symptoms, without further specification	Non-specific/other (3)
F0103	Vascular dementia with acute debut with depressive symptoms	Non-specific/other (3)
F0113	Multi-infarction dementia with depressive symptoms	Non-specific/other (3)
F0123	Subcortical vascular dementia with depressive symptoms	Non-specific/other (3)
F0133	Mixed cortical and subcortical vascular dementia with depressive symptoms	Non-specific/other (3)

F0183	Other vascular dementia with depressive symptoms	Non-specific/other (3)
F0193	Vascular dementia with depressive symptoms, without further specification	Non-specific/other (3)
F0203	Dementia in Picks disease with depressive symptoms	Non-specific/other (3)
F0213	Dementia in Creutzfeldt-Jakobs disease with depressive symptoms	Non-specific/other (3)
F0223	Dementia in Huntingtons disease with depressive symptoms	Non-specific/other (3)
F0233	Dementia in Parkinsons disease with depressive symptoms	Non-specific/other (3)
F0243	Dementia in HIV with depressive symptoms	Non-specific/other (3)
F0283	Dementia with depressive symptoms in other disease	Non-specific/other (3)
F0393	Dementia with depressive symptoms, without further specification	Non-specific/other (3)
F0632	Organic depressive mental disease	Non-specific/other (3)
F1x54	Drug-induced depression	Non-specific/other (3)
F341	Dysthymia	Non-specific/other (3)
F530	Puerperal depression	Non-specific/other (3)
F3810	Recurrent brief depressive disorder	Non-specific/other (3)

Phobic anxiety		
F40.0	Agrophobia	Definite (1)
F40.1	Social phobias	Definite (1)
F40.2	Specific (isolated phobias)	Definite (1)
F40.8	Other phobic anxiety disorders	Definite (1)
F40.9	Phobic anxiety disorders, unspecified	Definite (1)

Other anxiety disorders		
F41.0	Panic disorders (episodic paroxysmal anxiety)	Definite (1)
F41.1	Generalized anxiety disorder	Definite (1)
F41.2	Mixed anxiety and depressive disorder	Definite (1)
F41.3	Other mixed anxiety disorders	Definite (1)
F41.8	Other specified anxiety disorders	Definite (1)
F41.9	Anxiety disorder, unspecified	Definite (1)
F064	Organic anxiety disorder	Non-specific/other (3)

Stress and adjustment disorder		
F430	Acute stress reaction	
F431	Post-traumatic stress disorder	
F432	Adjustment disorder	
F438	Other reactions to severe stress	
F439	Reaction to severe stress, unspecified	

Appendix: Final code lists

Herpes zoster	3
Longer-term zoster complications	5
Depression	6
Anxiety disorder	10
Partner bereavement	13
Residence in communal establishment	14
Stress and adjustment disorder	16
Referral to mental health service	18
Antidepressants	20
Rheumatoid arthritis	36
Lupus erythematosus	39
Chronic obstructive pulmonary disease	42
Asthma	45
Antiasthmatics	48
Inhaled steroids	71
Chronic kidney disease	79
Diabetes	85
Antidiabetics	101
Human immunodeficiency virus infection	119
Lymphoma	122
Leukaemia	131
Myeloma	136
Haematopoietic stem cell transplantation	137
Other immunosuppressive disease	139
Oral glucocorticoids	141
Other immunosuppressants	146
Solid organ transplant	164
Alcohol use	167
Smoking	173
Myocardial infarction	181
Other ischaemic heart disease	184
Hypertension	192

Alcoholic liver disease 195

Pancreatitis 197

Peripheral artery disease 198

Heart failure 204

Cerebrovascular disease 206

Charlson Comorbidity Index 215

Herpes zoster

Medcode	Readterm
390	Herpes zoster
516	Shingles
7331	Ramsey Hunt Syndrome
8936	Ophthalmic herpes zoster infection
14718	Herpes zoster with ophthalmic complication
14793	Herpes zoster otitis externa
18918	Herpes zoster ophthalmicus
21069	Herpes zoster with unspecified complication
21471	Herpes zoster NOS
25320	Herpes zoster with dermatitis of eyelid
27403	Geniculate herpes zoster
27546	Herpes zoster with keratoconjunctivitis
31681	Herpes zoster - otitis externa
33810	Herpes zoster with other ophthalmic complication
38531	Herpes zoster with other specified complication NOS
39692	Polyneuropathy in herpes zoster
43235	Herpes zoster with other specified complication
44944	Herpes zoster with meningitis
47375	Zoster encephalitis
50537	Herpes zoster with other CNS complications
51692	Encephalitis due to herpes zoster
52126	Herpes zoster with other central nervous system complication
52319	Disseminated zoster
55940	Herpes zoster iridocyclitis
57895	Herpes zoster meningitis
62558	Infective otitis externa due to herpes zoster
63739	Herpes zoster with other CNS complication NOS
69405	Herpes zoster encephalitis
70197	[X]Zoster without complications
71464	Meningitis due to herpes zoster virus
105157	Hutchinson's sign - herpes zoster involving nose tip

ICD-10 code	ICD term
B02	Herpes zoster
B02.0	Zoster encephalitis
B02.1	Zoster meningitis
B02.3	Zoster ocular disease
B02.7	Disseminated zoster

B02.8	Zoster with other complications
B02.9	Zoster without complications

Longer-term zoster complications

Medcode	Readterm
1598	Post-herpetic neuralgia
7584	Post-herpetic trigeminal neuralgia
10223	Postherpetic neuralgia
11498	Postherpetic trigeminal neuralgia
17180	Postzoster neuralgia
31709	Postherpetic polyneuropathy

ICD-10 code	ICD term
B02.2	Zoster other nervous complication
G53.0	PHN or Ramsey Hunt

Depression

Medcode	Readterm	Subgroup
324	Depressive disorder NEC	Definite
543	[X]Depression NOS	Definite
595	Endogenous depression	Definite
1055	Agitated depression	Definite
1908	O/E - depressed	Definite
1996	Depressed	Definite
2147	Poor self esteem	Definite
2560	Depressive psychoses	Definite
2930	C/O - feeling unhappy	Definite
2970	[X]Depressive episode, unspecified	Definite
3291	[X]Depressive disorder NOS	Definite
3292	[X]Recurrent depressive disorder	Definite
4323	Chronic depression	Definite
4639	[X]Depressive episode	Definite
4824	C/O - feeling depressed	Definite
5879	Agitated depression	Definite
6482	Recurrent depression	Definite
6546	Endogenous depression first episode	Definite
6854	[X]Other depressive episodes	Definite
6932	Endogenous depression - recurrent	Definite
6950	Endogenous depression first episode	Definite
7011	Single major depressive episode NOS	Definite
7412	Loss of confidence	Definite
8826	[X]SAD - Seasonal affective disorder	Definite
8928	Low mood	Definite
9183	Masked depression	Definite
9211	[X]Moderate depressive episode	Definite
9667	[X]Severe depressive episode without psychotic symptoms	Definite
9796	Symptoms of depression	Definite
10015	Depressed mood	Definite
10438	Depressive symptoms	Definite
10610	Single major depressive episode	Definite
10667	[X]Mild depression	Definite
10720	[X]Atypical depression	Definite
10825	Seasonal affective disorder	Definite
11252	[X]Major depression, recurrent without psychotic symptoms	Definite
11329	[X]Endogenous depression without psychotic symptoms	Definite
11717	[X]Mild depressive episode	Definite
12099	[X]Severe depressive episode with psychotic symptoms	Definite
14709	Recurrent major depressive episodes, moderate	Definite

15099	Recurrent major depressive episode	Definite
15155	Single major depressive episode, moderate	Definite
15219	Single major depressive episode, severe, without psychosis	Definite
16506	Single major depressive episode, mild	Definite
16861	[X]Recurrent severe episodes of psychotic depression	Definite
18510	[X]Single episode of psychogenic depression	Definite
19696	[X]Recurrent episodes of psychogenic depression	Definite
22806	[X]Single episode major depression w'out psychotic symptoms	Definite
23731	[X]Endogenous depression with psychotic symptoms	Definite
24112	[X]Single episode of psychotic depression	Definite
24117	[X]Single episode of major depression and psychotic symptoms	Definite
24171	Recurrent major depressive episodes, severe, with psychosis	Definite
25435	Loss of capacity for enjoyment	Definite
25563	Recurrent major depressive episode NOS	Definite
25697	Recurrent major depressive episodes, severe, no psychosis	Definite
26028	Sad mood	Definite
27491	Atypical depressive disorder	Definite
28756	[X]Seasonal depressive disorder	Definite
29342	Recurrent major depressive episodes, mild	Definite
29520	[X]Recurrent depressive disorder, current episode moderate	Definite
29784	[X]Recurrent depressive disorder, current episode mild	Definite
30740	Loss of interest	Definite
31757	[X]Recurr severe episodes/psychogenic depressive psychosis	Definite
32159	Single major depressive episode, severe, with psychosis	Definite
32941	[X]Recurr severe episodes/major depression+psychotic symptom	Definite
33469	[X]Recurr depress disorder cur epi severe without psyc sympt	Definite
34390	Single major depressive episode, unspecified	Definite
35671	Recurrent major depressive episodes, unspecified	Definite
36616	[X]Monopolar depression NOS	Definite
41989	[X]Single episode agitated depressn w'out psychotic symptoms	Definite
44300	[X]Recurrent depressive disorder, unspecified	Definite
47009	[X]Recurrent depress disorder cur epi severe with psyc symp	Definite
47731	[X]Other recurrent depressive disorders	Definite
52678	[X]Single episode of psychogenic depressive psychosis	Definite
53148	Loss of hope for the future	Definite
56609	[X]Single episode of masked depression NOS	Definite
59386	[X]Single episode vital depression w'out psychotic symptoms	Definite
59869	Loss of interest in previously enjoyable activity	Definite
73991	[X]Vital depression, recurrent without psychotic symptoms	Definite
98252	[X]Major depression, moderately severe	Definite
98346	[X]Major depression, mild	Definite
98414	[X]Major depression, severe without psychotic symptoms	Definite
98417	[X]Major depression, severe with psychotic symptoms	Definite

5987	[X] Reactive depression NOS	Definite
7604	[X]Single episode of reactive depression	Definite
8478	Reactive depressive psychosis	Definite
8851	[X]Recurrent episodes of depressive reaction	Definite
8902	[X]Recurrent episodes of reactive depression	Definite
9055	[X]Single episode of depressive reaction	Definite
17770	Psychotic reactive depression	Definite
37764	[X]Recurrent severe episodes/reactive depressive psychosis	Definite
101422	Feeling low or worried	Definite
12122	Depression medication review	Possible
12399	Depression annual review	Possible
43239	Excepted from depression quality indicators: Informed dissent	Possible
28970	Excepted from depression quality indicators: Patient unsuita	Possible
30405	Depression interim review	Possible
30483	Patient given advice about management of depression	Possible
30583	Depression - enhanced services administration	Possible
42931	On depression register	Possible
44848	Depression management programme	Possible
48970	Exception reporting: depression quality indicators	Possible
51258	Depression monitoring administration	Possible
65435	Depression - enhanced service completed	Possible
71009	Depression monitoring first letter	Possible
72966	Depression monitoring second letter	Possible
85852	Depression monitoring telephone invite	Possible
88644	Depression monitoring verbal invite	Possible
91105	Depression monitoring third letter	Possible
96995	On full dose long term treatment depression - enh serv admin	Possible
100977	Suspected depression	Possible
11055	[X]Schizoaffective disorder, depressive type	Unspecific/other
20785	[X]Post-schizophrenic depression	Unspecific/other
35274	[X]Schizoaffective psychosis, depressive type	Unspecific/other
41022	[X]Schizophreniform psychosis, depressive type	Unspecific/other
8584	[X]Depressive neurosis	Unspecific/other
7737	[X]Neurotic depression	Unspecific/other
7953	[X]Dysthymia	Unspecific/other
10290	[X]Depressive personality disorder	Unspecific/other
10455	Depressive personality disorder	Unspecific/other
15220	[X]Persistant anxiety depression	Unspecific/other
1131	Neurotic depression reactive type	Unspecific/other
21887	Senile dementia with depression	Unspecific/other
27677	Presenile dementia with depression	Unspecific/other
27759	[X] Senile dementia, depressed or paranoid type	Unspecific/other
41089	Senile dementia with depressive or paranoid features NOS	Unspecific/other

43292	Arteriosclerotic dementia with depression	Unspecific/other
44674	Senile dementia with depressive or paranoid features	Unspecific/other
2972	Postviral depression	Unspecific/other
29527	[D]Postoperative depression	Unspecific/other
46244	Drug-induced depressive state	Unspecific/other
2639	Postnatal depression	Unspecific/other
103677	[X]Antenatal depression	Unspecific/other
2923	Puerperal depression	Unspecific/other
4979	[X]Postpartum depression NOS	Unspecific/other
13307	[X]Postnatal depression NOS	Unspecific/other
19054	[X]Recurrent brief depressive episodes	Unspecific/other
55384	Recurrent major depressive episodes, in full remission	Unspecific/other
56273	Recurrent major depressive episodes,partial/unspec remission	Unspecific/other
22116	[X]Recurrent depressive disorder, currently in remission	Unspecific/other
101153	[X]Recurr major depr ep, severe with psych, psych in remiss	Unspecific/other

ICD-10 code	ICD term	Subgroup
F32	Depressive episode	Definite
F32.0	Mild depressive episode	Definite
F32.1	Moderate depressive episode	Definite
F32.2	Severe depressive episode without psychotic symptoms	Definite
F32.3	Severe depressive episode with psychotic symptoms	Definite
F32.8	Other depressive episodes	Definite
F32.9	Depressive episode, unspecified	Definite
F33	Recurrent depressive disorder	Definite
F33.0	Recurrent depressive disorder, current episode mild	Definite
F33.1	Recurrent depressive disorder, current episode moderate	Definite
F33.2	Recurrent depressive disorder, current episode severe without psychotic symptoms	Definite
F33.3	Recurrent depressive disorder, current episode severe with psychotic symptoms	Definite
F33.4	Recurrent depressive disorder, currently in remission	Unspecific/other
F33.8	Other recurrent depressive disorders	Definite
F33.9	Recurrent depressive disorder, unspecified	Definite
F25	Schizoaffective disorder	Unspecific/other
F25.0	Schizoaffective disorder, manic type	Unspecific/other
F25.1	Schizoaffective disorder, depressive type	Unspecific/other
F25.2	Schizoaffective disorder, mixed manio-depressive type	Unspecific/other
F25.8	Schizoaffective disorder, other type	Unspecific/other
F25.9	Schizoaffective disorder, unspecified	Unspecific/other
F20.4	Post-schizophrenic depression	Unspecific/other
F34.1	Dysthymia	Unspecific/other
F53.0	Puerperal depression	Unspecific/other

Anxiety disorder

Medcode	Readterm	Subgroup
1723	Claustrophobia	Definite
1907	Phobic disorders	Definite
2300	Phobia unspecified	Definite
2571	[X]Agoraphobia	Definite
3076	Agoraphobia with panic attacks	Definite
7222	[X]Phobia NOS	Definite
9386	[X]Phobic anxiety disorders	Definite
9785	[X]Specific (isolated) phobias	Definite
9944	Phobic anxiety	Definite
11280	[X]Claustrophobia	Definite
11602	[X]Social phobias	Definite
12635	[X]Simple phobia	Definite
12838	Agoraphobia without mention of panic attacks	Definite
14729	Phobic disorder NOS	Definite
14890	[X]Panic disorder with agoraphobia	Definite
16199	Social phobia, fear of eating in public	Definite
16638	Social phobic disorders	Definite
16729	[X]Agoraphobia without history of panic disorder	Definite
18248	[X]Animal phobias	Definite
18603	Social phobia, fear of public washing	Definite
25749	Phobia counselling	Possible
27685	[X]Other phobic anxiety disorders	Definite
28106	Acrophobia	Definite
28938	Animal phobia	Definite
31957	Social phobia, fear of public speaking	Definite
42788	[X]Social neurosis	Definite
34064	[X]Phobic anxiety disorder, unspecified	Definite
63521	Antiphobic therapy	Possible
67898	[X]Phobic state NOS	Definite
67965	[X]Acrophobia	Definite
31672	Fear of crowds	Definite
462	Panic attack	Definite
655	Anxiety with depression	Definite
962	[X]Anxiety neurosis	Definite
1758	Chronic anxiety	Definite
4069	Panic disorder	Definite
4081	[X]Panic state	Definite
4534	Anxiety state NOS	Definite
4634	Recurrent anxiety	Definite
4659	Generalised anxiety disorder	Definite

5385	[X]Other anxiety disorders	Definite
6408	[X]Panic attack	Definite
6939	Anxiety state unspecified	Definite
7749	[X]Mild anxiety depression	Definite
7999	Anxiety counselling	Possible
8205	[X]Panic disorder [episodic paroxysmal anxiety]	Definite
9125	Anxiety management training	Possible
10344	[X]Generalized anxiety disorder	Definite
11913	[X]Mixed anxiety and depressive disorder	Definite
20773	[X]Organic anxiety disorder	Unspecific/other
22159	Acknowledging anxiety	Possible
23838	[X]Anxiety disorder, unspecified	Definite
24066	[X]Other specified anxiety disorders	Definite
25638	[X]Anxiety NOS	Definite
28167	[X]Anxiety hysteria	Definite
35825	[X]Anxiety reaction	Definite
44321	[X]Other mixed anxiety disorders	Definite
50191	[X]Anxiety state	Definite
62935	Recognising anxiety	Possible
2366	Dental phobia	Possible
12508	[X]Needle phobia	Possible
20802	Flying phobia	Possible
4167	Fear of flying	Possible
636	Anxiety states	Possible
11890	C/O - panic attack	Possible
19000	O/E - panic attack	Possible
26295	Reducing anxiety	Possible
28381	Alleviating anxiety	Possible

icd	icd_description	Subgroup
F40	Phobic anxiety disorders	Definite
F40.0	Agrophobia	Definite
F40.1	Social phobias	Definite
F40.2	Specific (isolated phobias)	Definite
F40.8	Other phobic anxiety disorders	Definite
F40.9	Phobic anxiety disorders, unspecified	Definite
F41	Other anxiety disorders	Definite
F41.0	Panic disorders (episodic paroxysmal anxiety)	Definite
F41.1	Generalized anxiety disorder	Definite
F41.2	Mixed anxiety and depressive disorder	Definite
F41.3	Other mixed anxiety disorders	Definite

F41.8	Other specified anxiety disorders	Definite
F41.9	Anxiety disorder, unspecified	Definite
F06.4	Organic anxiety disorder	Unspecific/other

Partner bereavement

Medcode	Readterm
207	Death of spouse
11251	Death of wife
12076	Husband died
12325	Death of husband
17802	Husband died
25097	Death of husband
28440	Death of partner
36947	Partner dying

Residence in communal establishment

Medcode	Readterm
1123	In prison
7101	Seen in old people's home
7653	Seen in nursing home
10993	Discharge to nursing home
11419	Lives in an old peoples home
13359	Lives in a nursing home
13360	Nursing/other home
15840	Lives in a welfare home
17782	Seen in institution
21280	Resident in part III accomodation
24494	[V]Institution resident
24816	Residential care
24828	Nursing home care
24956	Lives in a residential home
27360	Part III accomodation arranged
27425	Part III accommodation
27936	Delayed discharge to nursing home
27968	Residential institution
28655	[V]Imprisonment
36096	Part 3 accomodation
41505	Patient in institution-perm.
42191	Discharge to residential home
43915	Discharge to private nursing home
46303	[X]Unspecified fall, occurrence in residential institution
46642	Other residential care homes managed by local authority
47055	[V]Imprisonment and other incarceration
49681	Lives in care home
50792	Seen in Part 3 accomodation
52249	Lives on council site
52682	Prison medical examination
53140	Local authority residential care
53439	[V]Prison medical
55276	Lives in a community
59548	Lives in an old peoples home
66122	Part 3 accomodation arranged
68005	Lives in a welfare home
69028	Discharge to part III residential home
73083	Nursing home visit note
73321	Nursing home
93998	Residential home visit note

94070	Provision of continuing care in nursing home
95555	Lives in a commune
95795	FP22 - removal from residential institute
99148	Other residential care home man voluntary/private agents
101003	Home visit request by residential institution
102493	Admission to nursing home
102598	Discharge to nursing home
107443	Care home visit for initial patient assessment
107602	Care home visit for follow-up patient review
107757	Care home visit

Stress and adjustment disorder

Medcode	Readterm
276	Acute reaction to stress
1533	Brief depressive reaction
2775	Grief reaction
2826	Adjustment reaction
4171	[X]Post - traumatic stress disorder
6075	Adjustment reaction with aggression
7813	[X]Psychic shock
8250	[D]State of emotional shock and stress, unspecified
10535	[X]Acute reaction to stress
11098	[X]Reaction to severe stress, and adjustment disorders
11336	[X]Adjustment disorders
11607	[X]Acute stress reaction
11940	Acute panic state due to acute stress reaction
15551	Acute stupor state due to acute stress reaction
15665	Adjustment reaction with disturbance of other emotion NOS
16415	Adjustment reaction with predominant disturbance of conduct
16632	Prolonged depressive reaction
19921	Other adjustment reaction with withdrawal
20245	Acute situational disturbance
21197	[X]Grief reaction
21559	[X]Crisis state
21753	[X]Other reactions to severe stress
23462	Other adjustment reactions NOS
23869	Stress reaction causing mixed disturbance of emotion/conduct
24847	Acute posttrauma stress state
26138	Acute stress reaction NOS
27390	Adjustment reaction due to hospitalisation
28248	[X]Prolonged single episode of reactive depression
28863	[X]Single episode of reactive depressive psychosis
29707	Other acute stress reaction NOS
31515	[X]Reaction to severe stress, unspecified
32182	[X]Traumatic neurosis
32387	Other post-traumatic stress disorder
35632	Adjustment reaction with physical symptoms
35914	Adjustment reaction with antisocial behaviour
36228	[X]Culture shock
36246	Brief depressive reaction NOS
36374	[X]Acute crisis reaction
37669	Adjustment reaction NOS
38640	Other acute stress reactions

41455	Other adjustment reactions
42737	Acute fugue state due to acute stress reaction
43550	Combat fatigue
48588	Adjustment reaction with mixed disturbance of emotion
56020	[X]State of emotional shock and stress, unspecified
56924	Adjustment reaction with anxious mood
58013	Culture shock
66398	Adjustment reaction with destructiveness
70779	[X]Combat fatigue
101725	[X]Chron post-traumatic stress disorder follow military comb
101785	[X]Acute post-traumatic stress disorder follow military comb
104891	Adjustment reaction with predominant disturbance conduct NOS

icd	icd_description
F43	Reaction to severe stress, and adjustment disorders
F43.0	Acute stress reaction
F43.1	Post-traumatic stress disorder
F43.2	Adjustment disorder
F43.8	Other reactions to severe stress
F43.9	Reaction to severe stress, unspecified

Referral to mental health service

Medcode	Readterm
1690	Refer to psychologist
2189	Psychiatric referral
5338	Referral to psychogeriatrician
9868	Referral to psychiatric nurse
10002	Referral to psychiatrist
10236	Referral to community mental health team
10669	Referral to psychotherapist
10828	Referral to mental health counsellor
10967	Refer to psychologist
11270	Referral to psychologist
11449	Referral to mental health crisis team
11958	Referral to psychotherapist
12053	Referral to mental health team
12365	Referral to older age community mental health team
12447	Referral to primary care mental health gateway worker
13677	Private referral to psychiatrist
13703	Referral to non NHS mental health community service
18956	Referral to bereavement counsellor
24504	Refer to mental health worker
25950	Referral to clinical physiologist
26178	Referral to psychosis early intervention service
27640	Psychiatric self-referral
28925	Referral for guided self-help for anxiety
30861	Referral to nurse behavioural therapist
32841	Referral for guided self-help for depression
34532	Private referral to psychogeriatrician
34538	Referral to nurse psychotherapist
36728	Referral to liaison psychiatrist
40894	Referral to psychiatrist for mental handicap
41040	Referral to primary care mental health graduate worker
41071	Referral for mental health self-help literature
41267	Referral to complementary therapist
42932	Referral to bereavement counsellor
44910	Referral to rehabilitation psychiatrist
84212	Referral to primary care mental health team
95163	Emergency voluntary psychiatric admission Mental Health Act
95936	Urgent referral to psychiatrist
97420	Referral to improving access to psychological therapies prog
99630	Referral for cognitive behavioural therapy
105357	Private referral to psychologist

102636	Patient initiated encounter with mental health crisis team
10308	Under care of mental health team
108558	Under care of mental health in-reach team
32602	Under care of mental health counsellor
11425	Seen by mental health counsellor
105244	Referral to IAPT (imp acc to psych thera) programme declined

Antidepressants

MAO inhibitors

Prodcode	Product name
2883	Moclobemide 150mg tablets
3349	Nardil 15mg tablets (Archimedes Pharma UK Ltd)
3356	Parstelin Tablet (GlaxoSmithKline Consumer Healthcare)
3783	Tranylcypromine 10mg tablets
3955	Tranylcypromine with trifluoperazine Tablet
4321	Phenelzine 15mg tablets
5187	Moclobemide 300mg tablets
5832	Manerix 300mg tablets (Meda Pharmaceuticals Ltd)
9206	Manerix 150mg tablets (Meda Pharmaceuticals Ltd)
10787	Parnate 10mg Tablet (Goldshield Pharmaceuticals Ltd)
12207	Isocarboxazid 10mg tablets
12503	Marplan 10mg Tablet (Cambridge Laboratories Ltd)
18290	Marsilid 25mg Tablet (Roche Products Ltd)
23728	MARSILID 50 MG TAB
24890	Trifluoperazine with tranylcypromine 1mg + 10mg Tablet
25945	Iproniazid 25mg
41654	Tranylcypromine 10mg tablets (AMCo)
41731	Isocarboxazid 10mg Tablet (Cambridge Laboratories Ltd)
41747	Moclobemide 150mg tablets (Teva UK Ltd)
55620	Flupentixol Liquid

SSRIs

Prodcode	Product name
22	Fluoxetine 20mg capsules
50	Paroxetine 20mg tablets
67	Citalopram 20mg tablets
252	Prozac 20mg/5ml liquid (Eli Lilly and Company Ltd)
418	Prozac 20mg capsules (Eli Lilly and Company Ltd)
476	Citalopram 10mg tablets
488	Sertraline 50mg tablets
513	Citalopram 40mg/ml oral drops sugar free
527	Paroxetine 10mg/5ml oral suspension sugar free
603	Escitalopram 10mg tablets
648	Cipralex 10mg tablets (Lundbeck Ltd)
727	Sertraline 100mg tablets
785	Cipralex 5mg tablets (Lundbeck Ltd)
815	Cipramil 40mg/ml drops (Lundbeck Ltd)
841	Seroxat 20mg tablets (GlaxoSmithKline UK Ltd)
1397	Paroxetine 30mg tablets

1575	Seroxat 30mg tablets (GlaxoSmithKline UK Ltd)
1612	Lustral 50mg tablets (Pfizer Ltd)
1712	Cipramil 20mg tablets (Lundbeck Ltd)
2290	Fluvoxamine 100mg tablets
2408	Cipramil 40mg tablets (Lundbeck Ltd)
2548	Fluoxetine 20mg/5ml oral solution
2880	Fluvoxamine 50mg tablets
2897	Faverin 50mg tablets (BGP Products Ltd)
3391	Dutonin 100mg tablets (Bristol-Myers Squibb Pharmaceuticals Ltd)
3601	Seroxat 20mg/10ml liquid (GlaxoSmithKline UK Ltd)
3861	Cipramil 10mg tablets (Lundbeck Ltd)
4011	Nefazodone 200mg tablets
4075	Fluoxetine 60mg capsules
4297	Dutonin 200mg tablets (Bristol-Myers Squibb Pharmaceuticals Ltd)
4352	Lustral 100mg tablets (Pfizer Ltd)
4554	Nefazodone 100mg tablets
4770	Citalopram 40mg tablets
4907	Prozac 60mg capsules (Eli Lilly and Company Ltd)
6218	Escitalopram 20mg tablets
6360	Cipralex 20mg tablets (Lundbeck Ltd)
6405	Escitalopram 5mg tablets
7328	Sertraline 50mg/5ml oral suspension
9496	Dutonin tablets treatment initiation pack (Bristol-Myers Squibb Pharmaceuticals Ltd)
12123	Faverin 100mg tablets (BGP Products Ltd)
14740	Oxactin 20mg capsules (Discovery Pharmaceuticals Ltd)
19183	Fluoxetine 20mg capsules (A A H Pharmaceuticals Ltd)
19470	Fluoxetine 20mg capsules (Ranbaxy (UK) Ltd)
20152	Escitalopram 10mg/ml oral drops sugar free
23334	FAVERIN
26016	Citalopram 20mg tablets (Sandoz Ltd)
26056	Cipralex 10mg/ml oral drops (Lundbeck Ltd)
29756	Paxoran 20mg Tablet (Ranbaxy (UK) Ltd)
29786	Ranflutin 20mg capsules (Ranbaxy (UK) Ltd)
30258	Fluoxetine 20mg/5ml oral solution (Teva UK Ltd)
32401	Sertraline 50mg tablets (A A H Pharmaceuticals Ltd)
32546	Paxoran 10mg Tablet (Ranbaxy (UK) Ltd)
32848	Citalopram 10mg tablets (Actavis UK Ltd)
32899	Paroxetine 20mg tablets (Actavis UK Ltd)
33071	Felicium 20mg capsules (Opus Pharmaceuticals Ltd)
33410	Fluoxetine 20mg capsules (Zentiva)
33720	Citalopram 10mg tablets (IVAX Pharmaceuticals UK Ltd)
33779	Prozit 20mg/5ml oral solution (Pinewood Healthcare)
33978	Paroxetine 20mg tablets (Generics (UK) Ltd)

34202	Fluoxetine 20mg capsules (Genus Pharmaceuticals Ltd)
34216	Fluoxetine 20mg/5ml oral solution (A A H Pharmaceuticals Ltd)
34288	Fluoxetine 20mg capsules (Generics (UK) Ltd)
34294	Fluoxetine 20mg capsules (IVAX Pharmaceuticals UK Ltd)
34351	Paroxetine 20mg tablets (IVAX Pharmaceuticals UK Ltd)
34356	Citalopram 20mg tablets (A A H Pharmaceuticals Ltd)
34413	Citalopram 10mg tablets (Zentiva)
34415	Citalopram 20mg tablets (Generics (UK) Ltd)
34419	Paroxetine 20mg tablets (A A H Pharmaceuticals Ltd)
34436	Citalopram 10mg tablets (Generics (UK) Ltd)
34456	Fluoxetine 20mg capsules (Teva UK Ltd)
34466	Citalopram 40mg tablets (Sandoz Ltd)
34498	Citalopram 10mg Tablet (Neo Laboratories Ltd)
34499	Citalopram 10mg tablets (Sandoz Ltd)
34586	Citalopram 10mg tablets (A A H Pharmaceuticals Ltd)
34587	Paroxetine 30mg tablets (A A H Pharmaceuticals Ltd)
34603	Citalopram 40mg tablets (Generics (UK) Ltd)
34722	Citalopram 20mg Tablet (Neo Laboratories Ltd)
34822	Citalopram 20mg tablets (Zentiva)
34849	Fluoxetine 20mg capsules (Tillomed Laboratories Ltd)
34856	Fluoxetine 60mg capsules (Generics (UK) Ltd)
34871	Citalopram 20mg tablets (Actavis UK Ltd)
34966	Citalopram 20mg tablets (Teva UK Ltd)
34970	Citalopram 20mg tablets (Niche Generics Ltd)
35021	Paroxetine 10mg tablets
35112	Seroxat 10mg tablets (GlaxoSmithKline UK Ltd)
36746	Citalopram 40mg tablets (A A H Pharmaceuticals Ltd)
36893	Fluoxetine 20mg/5ml oral solution sugar free
37256	Prozep 20mg/5ml oral solution (Chemidex Pharma Ltd)
38890	Fluoxetine 20mg Capsule (Milpharm Ltd)
40165	Paroxetine 30mg tablets (Actavis UK Ltd)
40726	Escitalopram 20mg/ml oral drops sugar free
40892	Paroxetine 20mg tablets (Genus Pharmaceuticals Ltd)
41062	Cipralex 20mg/ml oral drops (Lundbeck Ltd)
41528	Citalopram 10mg tablets (Teva UK Ltd)
42107	Fluoxetine 20mg capsules (Niche Generics Ltd)
42387	Sertraline 50mg tablets (Actavis UK Ltd)
42499	Fluoxetine 10mg tablets
42660	Citalopram 10mg tablets (Almus Pharmaceuticals Ltd)
42803	Fluoxetine 20mg/5ml oral solution (IVAX Pharmaceuticals UK Ltd)
43518	Fluvoxamine 100mg tablets (IVAX Pharmaceuticals UK Ltd)
43519	Citalopram 40mg Tablet (Neo Laboratories Ltd)
44861	Fluvoxamine 100mg tablets (Actavis UK Ltd)

44944	Sertraline 100mg tablets (Teva UK Ltd)
45223	Citalopram 40mg tablets (Niche Generics Ltd)
45224	Fluoxetine 20mg capsules (Sandoz Ltd)
45247	Fluoxetine 20mg capsules (Fannin UK Ltd)
45286	Citalopram 10mg tablets (Niche Generics Ltd)
45304	Citalopram 40mg tablets (Teva UK Ltd)
45316	Fluoxetine 20mg capsules (Wockhardt UK Ltd)
45329	Fluoxetine 20mg capsules (Actavis UK Ltd)
45915	Sertraline 50mg tablets (Almus Pharmaceuticals Ltd)
46926	Citalopram 40mg tablets (Zentiva)
46977	Citalopram 40mg tablets (Actavis UK Ltd)
48026	Citalopram 20mg tablets (Almus Pharmaceuticals Ltd)
48045	Fluvoxamine 100mg tablets (A A H Pharmaceuticals Ltd)
48220	Prozac 20mg capsules (Lexon (UK) Ltd)
49165	Citalopram 10mg tablets (Alliance Healthcare (Distribution) Ltd)
49519	Sertraline 100mg/5ml oral suspension
52100	Citalopram 10mg tablets (Arrow Generics Ltd)
52354	Citalopram 20mg tablets (DE Pharmaceuticals)
52408	Citalopram 10mg tablets (Kent Pharmaceuticals Ltd)
52607	Citalopram 20mg tablets (Bristol Laboratories Ltd)
52824	Citalopram 10mg tablets (PLIVA Pharma Ltd)
53394	Citalopram 20mg tablets (Alliance Healthcare (Distribution) Ltd)
53787	Citalopram 10mg tablets (Bristol Laboratories Ltd)
54081	Sertraline 25mg/5ml oral suspension
54826	Sertraline 150mg/5ml oral suspension
54827	Citalopram 10mg/5ml oral suspension
54933	Sertraline 100mg tablets (PLIVA Pharma Ltd)
55023	Paroxetine 20mg tablets (Medreich Plc)
55033	Citalopram 40mg tablets (DE Pharmaceuticals)
55146	Sertraline 100mg tablets (A A H Pharmaceuticals Ltd)
55488	Sertraline 50mg tablets (Teva UK Ltd)
55537	Seroxat 30mg tablets (Lexon (UK) Ltd)
56009	Citalopram 20mg tablets (Arrow Generics Ltd)
56292	Citalopram 40mg/ml oral drops sugar free (Actavis UK Ltd)
56355	Citalopram 10mg tablets (Waymade Healthcare Plc)
57532	Prozac 20mg capsules (Waymade Healthcare Plc)
57936	Citalopram 40mg/ml oral drops sugar free (A A H Pharmaceuticals Ltd)
58476	Citalopram 20mg tablets (Aurobindo Pharma Ltd)
58664	Sertraline 50mg tablets (Generics (UK) Ltd)
58723	Sertraline 50mg tablets (Accord Healthcare Ltd)
59193	Citalopram 10mg tablets (Ranbaxy (UK) Ltd)
59288	Paroxetine 10mg tablets (Actavis UK Ltd)
59358	Fluoxetine 20mg capsules (Milpharm Ltd)

59600	Sertraline 100mg tablets (Almus Pharmaceuticals Ltd)
59650	Citalopram 10mg tablets (Aurobindo Pharma Ltd)
60138	Fluoxetine 20mg orodispersible tablets sugar free
60534	Fluoxetine 20mg dispersible tablets sugar free
60568	Citalopram 20mg tablets (Waymade Healthcare Plc)
60619	Fluoxetine 20mg/5ml oral solution (Kent Pharmaceuticals Ltd)
60839	Citalopram 40mg tablets (Almus Pharmaceuticals Ltd)
60888	Citalopram 10mg tablets (Sigma Pharmaceuticals Plc)
60962	Fluoxetine 20mg capsules (Alliance Healthcare (Distribution) Ltd)
61335	Prozac 20mg capsules (Mawdsley-Brooks & Company Ltd)
61503	Sertraline 100mg tablets (Actavis UK Ltd)
62155	Fluoxetine 20mg capsules (Phoenix Healthcare Distribution Ltd)
62335	Olena 20mg dispersible tablets (AMCo)
62692	Sertraline 100mg tablets (Bristol Laboratories Ltd)
62693	Sertraline 50mg tablets (Bristol Laboratories Ltd)
62819	Sertraline 12.5mg/5ml oral suspension
62927	Sertraline 50mg tablets (Wockhardt UK Ltd)
62950	Sertraline 100mg tablets (Accord Healthcare Ltd)
63441	Citalopram 10mg tablets (Rivopharm (UK) Ltd)
63481	Sertraline 50mg tablets (Aurobindo Pharma Ltd)
63827	Dutonin 50mg tablets (Bristol-Myers Squibb Pharmaceuticals Ltd)
63916	Escitalopram 10mg tablets (Actavis UK Ltd)

TCAs

Prodcode	Product name
49	Amitriptyline 25mg tablets
74	Dosulepin 75mg tablets
83	Amitriptyline 10mg tablets
84	Dosulepin 25mg capsules
114	Lofepramine 70mg tablets
182	Tryptizol 10mg/ml Injection (Merck Sharp & Dohme Ltd)
487	Amitriptyline 25mg modified-release capsules
595	Amitriptyline 25mg / Perphenazine 2mg tablets
873	AMITRIPTYLINE 100 MG TAB
1169	Prothiaden 25mg capsules (Teofarma)
1208	Triptafen tablets (AMCo)
1310	Imipramine 10mg tablets
1453	Triptafen m 2mg+10mg Tablet (Goldshield Pharmaceuticals Ltd)
1730	Trazodone 100mg capsules
1809	Imipramine 25mg tablets
1888	Amitriptyline 50mg tablets
1940	Dothapax 25 capsules (Ashbourne Pharmaceuticals Ltd)

2039	Trimipramine 25mg tablets
2093	Gamanil 70mg tablets (Merck Serono Ltd)
2320	Prothiaden 75mg tablets (Teofarma)
2486	Lentizol 25mg modified-release capsules (Pfizer Ltd)
2525	Amitriptyline 75mg modified-release capsules
2531	Surmontil 50mg capsules (Sanofi)
2532	Surmontil 25mg tablets (Sanofi)
2533	TRIMIPRAMINE 50 MG TAB
2579	Tofranil 10mg Tablet (Novartis Pharmaceuticals UK Ltd)
2936	Motival 10mg/500microgram tablets (Sanofi)
2985	Lentizol 50mg modified-release capsules (Pfizer Ltd)
3083	Mianserin 10mg tablets
3183	Nortriptyline 10mg tablets
3194	Clomipramine 10mg capsules
3195	CLOMIPRAMINE 25 MG TAB
3196	Trimipramine 50mg capsules
3351	Amoxapine 50mg tablets
3355	Trazodone 50mg capsules
3490	Amitriptyline 10mg / Perphenazine 2mg tablets
3554	Doxepin 25mg capsules
3652	Amoxapine 100mg tablets
3657	Anafranil 25mg capsules (Novartis Pharmaceuticals UK Ltd)
3668	IMIPRAMINE 100 MG TAB
3670	Clomipramine 25mg capsules
3771	AMITRIPTYLINE 75 MG TAB
3777	Amitriptyline 10mg/5ml sugar free oral solution
3842	Doxepin 10mg capsules
3903	Nortriptyline 25mg tablets
3925	Clomipramine 50mg capsules
4003	Molipaxin 150mg tablets (Zentiva)
4020	Trazodone 150mg tablets
4118	Nortriptyline 10mg Capsule
4149	PROTRIPTYLINE HCl 10 MG TAB
4194	Molipaxin 100mg capsules (Zentiva)
4218	Lofepramine 70mg/5ml oral suspension sugar free
4310	Trimipramine 10mg tablets
4329	Mianserin 20mg tablets
4411	Amoxapine 150mg tablets
4682	Amitriptyline 50mg modified-release capsules
4690	Amitriptyline 50mg/5ml oral solution sugar free
4874	Molipaxin 50mg capsules (Zentiva)
5073	Doxepin 50mg capsules
6054	Dosulepin 25mg/5ml oral solution sugar free

6255	Mianserin 30mg tablets
6312	Amitriptyline 25mg/5ml oral solution sugar free
6442	Trazodone 50mg/5ml oral solution sugar free
6894	Perphenazine 2mg with Amitriptyline 25mg tablet
7059	Doxepin 75mg capsules
7468	Bolvidon 10mg Tablet (Organon Laboratories Ltd)
7475	AMOXAPINE 25 MG TAB
7515	Anafranil 10mg capsules (Novartis Pharmaceuticals UK Ltd)
7573	IMIPRAMINE 25 MG CAP
7677	Allegron 10mg tablets (King Pharmaceuticals Ltd)
7678	Nortriptyline 25mg Capsule
7693	Anafranil 50mg capsules (Novartis Pharmaceuticals UK Ltd)
7751	Tryptizol 25mg Tablet (Merck Sharp & Dohme Ltd)
7755	Concordin 10 Tablet (Merck Sharp & Dohme Ltd)
7756	Protriptyline 5mg tablet
7780	Nortriptyline 10mg / Fluphenazine 500microgram tablets
7784	IMIPRAMINE 50 MG TAB
7816	Concordin 5 Tablet (Merck Sharp & Dohme Ltd)
7894	Anafranil SR 75mg tablets (Novartis Pharmaceuticals UK Ltd)
7910	Tofranil 25mg tablets (Novartis Pharmaceuticals UK Ltd)
7979	Pertofran 25mg Tablet (Novartis Pharmaceuticals UK Ltd)
7981	Desipramine 25mg tablets
8055	Imipramine 25mg/5ml oral solution
8144	Bolvidon 20mg Tablet (Organon Laboratories Ltd)
8174	Molipaxin 50mg/5ml oral liquid (Sanofi)
8250	AMITRIPTYLINE S/F 25 MG/5ML SYR
8332	Tryptizol 50mg Tablet (Merck Sharp & Dohme Ltd)
8493	Motipress tablets (Sanofi-Synthelabo Ltd)
8585	Bolvidon 30mg Tablet (Organon Laboratories Ltd)
8640	Allegron 25mg tablets (King Pharmaceuticals Ltd)
8661	Clomipramine 75mg modified-release tablets
8719	Anafranil 25mg/5ml syrup (Novartis Pharmaceuticals UK Ltd)
8720	Clomipramine 25mg/5ml oral solution
8726	Tryptizol 10mg Tablet (Merck Sharp & Dohme Ltd)
8826	TOFRANIL 50 MG TAB
8831	Tryptizol mr 75mg Modified-release capsule (Merck Sharp & Dohme Ltd)
8878	Tryptizol 10mg/5ml sugar free Oral solution (Merck Sharp and Dohme Ltd)
8928	Surmontil 10mg tablets (Sanofi)
10413	Sinequan 10mg capsules (Pfizer Ltd)
10649	IMIPRAMINE 75 MG TAB
10948	Dosulepin 75mg/5ml oral solution sugar free
11187	Protriptyline 10mg tablet
11956	Norval 20mg Tablet (Bencard)

11963	Limbitrol 10 Capsule (Roche Products Ltd)
12111	Vivalan 50mg Tablet (AstraZeneca UK Ltd)
12125	Sinequan 50mg capsules (Pfizer Ltd)
12129	Sinequan 25mg capsules (Pfizer Ltd)
12192	Norval 30mg Tablet (Bencard)
12194	NORTRIPTYLINE 10 MG ELI
12227	Butriptyline 25mg tablets
12309	Viloxazine hcl 50mg tablets
12353	Aventyl 25mg Capsule (Eli Lilly and Company Ltd)
12368	Norval 10mg Tablet (Bencard)
12549	Aventyl 10mg/5ml Liquid (Eli Lilly and Company Ltd)
12710	Trazodone 150mg modified-release tablets
13318	ANAFRANIL 25 MG INJ
13496	AMITRIPTYLINE 200 MG TAB
13621	Molipaxin CR 150mg tablets (Aventis Pharma)
14398	Asendis 50mg Tablet (Wyeth Pharmaceuticals)
14519	Sinequan 75mg capsules (Pfizer Ltd)
14521	SINEQUAN 15 MG TAB
14534	Limbitrol 5 Capsule (Roche Products Ltd)
14578	Nortriptyline 30mg / Fluphenazine 1.5mg tablets
15380	Asendis 25mg Tablet (Wyeth Pharmaceuticals)
15632	Dothapax 75 tablets (Ashbourne Pharmaceuticals Ltd)
16323	Perphenazine 2mg with Amitriptyline 10mg tablet
17183	Aventyl 10mg Capsule (Eli Lilly and Company Ltd)
17319	Amoxapine 25mg tablets
18342	Amitriptyline 25mg / Chlordiazepoxide 10mg capsules
18932	Evadyne 25mg Tablet (Wyeth Pharmaceuticals)
19168	Dosulepin 25mg/5ml mixture
19181	Trazodone 100mg capsules (Generics (UK) Ltd)
19186	Dosulepin 75mg tablets (Actavis UK Ltd)
19779	Amitriptyline 10mg/ml injection
20026	Domical 25mg Tablet (Berk Pharmaceuticals Ltd)
20061	PERPHENAZINE 8 MG TAB
20571	Fluphenazine with nortriptyline 500microgramswith10mg Tablet
20712	AMITRIPTYLINE S/R
20715	LIMBITROL 5
21081	Amitriptyline 12.5mg / Chlordiazepoxide 5mg capsules
21157	Thaden 75mg tablets (Opus Pharmaceuticals Ltd)
21357	Asendis 100mg Tablet (Wyeth Pharmaceuticals)
21819	Prepadine 75mg tablets (Teva UK Ltd)
21820	Prepadine 25mg capsules (Teva UK Ltd)
22006	TRYPTIZOL
22070	Amitriptyline 10mg/5ml Oral solution (Rosemont Pharmaceuticals Ltd)

22872	DOXEPIN HCL
23426	Dosulepin 25mg capsules (A A H Pharmaceuticals Ltd)
23497	AMITRIPTYLINE 300 MG TAB
24134	Amitriptyline 25mg tablets (Kent Pharmaceuticals Ltd)
24141	Amitriptyline 10mg tablets (Actavis UK Ltd)
24145	Amitriptyline 25mg tablets (Actavis UK Ltd)
24147	Amitriptyline 25mg tablets (Teva UK Ltd)
24152	Amitriptyline 10mg tablets (Teva UK Ltd)
24680	Elavil 10mg Tablet (DDSA Pharmaceuticals Ltd)
24700	Prondol 15mg Tablet (Wyeth Pharmaceuticals)
24723	Asendis 150mg Tablet (Wyeth Pharmaceuticals)
25036	CLOMIPRAMINE HYDROCHLORIDE
25045	TRIMIPRAMINE
25070	LOFEPRAMINE
25085	TRIMIPRAMINE
25444	Lomont 70mg/5ml oral suspension (Rosemont Pharmaceuticals Ltd)
26213	Domical 10mg Tablet (Berk Pharmaceuticals Ltd)
26715	BOLVIDON 60 MG TAB
26822	PROTHIADEN
27008	Domical 50mg Tablet (Berk Pharmaceuticals Ltd)
27476	Iprindole hc 15mg
27568	SURMONTIL
27616	PROTHIADEN
27733	Iprindole hc 30mg
27876	AMITRIPTYLINE
29339	Trazodone 50mg capsules (Generics (UK) Ltd)
29857	Trazodone 150mg tablets (Teva UK Ltd)
29875	Dosulepin 25mg capsules (Generics (UK) Ltd)
30375	Anafranil 25mg/2ml solution for injection ampoules (Novartis Pharmaceuticals UK Ltd)
30376	Thaden 25mg capsules (Opus Pharmaceuticals Ltd)
30738	AMITRIPTYLINE S/F
30983	Trazodone 150mg tablets (Generics (UK) Ltd)
31672	Prondol 30mg Tablet (Wyeth Pharmaceuticals)
31824	Dosulepin 25mg capsules (IVAX Pharmaceuticals UK Ltd)
31826	Dosulepin 75mg tablets (IVAX Pharmaceuticals UK Ltd)
32121	Dosulepin 75mg tablets (A A H Pharmaceuticals Ltd)
32439	Amitriptyline 25mg Tablet (Sussex Pharmaceutical Ltd)
32457	Butriptyline 50mg tablets
32863	Imipramine 10mg tablets (Teva UK Ltd)
33074	Praminil 10mg Tablet (DDSA Pharmaceuticals Ltd)
33090	Amitriptyline 10mg tablets (A A H Pharmaceuticals Ltd)
33164	Dosulepin 25mg capsules (Sandoz Ltd)
33624	Amitriptyline 50mg tablets (Teva UK Ltd)

34003	Trazodone 50mg capsules (A A H Pharmaceuticals Ltd)
34046	Lofepramine 70mg tablets (A A H Pharmaceuticals Ltd)
34058	Dosulepin 75mg tablets (Teva UK Ltd)
34107	Amitriptyline 50mg tablets (Wockhardt UK Ltd)
34129	Amitriptyline 25mg tablets (Wockhardt UK Ltd)
34182	Amitriptyline 50mg tablets (Kent Pharmaceuticals Ltd)
34197	Amitriptyline 25mg Tablet (Berk Pharmaceuticals Ltd)
34222	Imipramine 10mg tablets (Actavis UK Ltd)
34223	Dosulepin 25mg capsules (Teva UK Ltd)
34224	Amitriptyline 25mg/5ml oral solution sugar free (Rosemont Pharmaceuticals Ltd)
34245	Clomipramine 25mg capsules (A A H Pharmaceuticals Ltd)
34251	Amitriptyline 50mg/5ml oral solution sugar free (Rosemont Pharmaceuticals Ltd)
34274	Amitriptyline 50mg tablets (A A H Pharmaceuticals Ltd)
34355	Imipramine 25mg tablets (Actavis UK Ltd)
34401	Amitriptyline 10mg tablets (Wockhardt UK Ltd)
34421	Trazodone 50mg capsules (Zentiva)
34470	Trazodone 150mg tablets (Zentiva)
34474	Amitriptyline 25mg Tablet (Regent Laboratories Ltd)
34503	Amitriptyline 25mg tablets (IVAX Pharmaceuticals UK Ltd)
34525	Dosulepin 75mg tablets (Generics (UK) Ltd)
34578	Lofepramine 70mg tablets (IVAX Pharmaceuticals UK Ltd)
34580	Trazodone 100mg capsules (A A H Pharmaceuticals Ltd)
34634	Amitriptyline 50mg tablets (Actavis UK Ltd)
34641	Dosulepin 25mg capsules (Sovereign Medical Ltd)
34643	Dosulepin 25mg capsules (Almus Pharmaceuticals Ltd)
34672	Lofepramine 70mg tablets (Sterwin Medicines)
34731	Amitriptyline 10mg tablets (Kent Pharmaceuticals Ltd)
34745	Dosulepin 25mg capsules (Actavis UK Ltd)
34782	Amitriptyline 25mg tablets (A A H Pharmaceuticals Ltd)
34813	Imipramine 25mg tablets (A A H Pharmaceuticals Ltd)
34866	Clomipramine 10mg capsules (A A H Pharmaceuticals Ltd)
34872	Imipramine 25mg Tablet (C P Pharmaceuticals Ltd)
34916	Amitriptyline 10mg Tablet (Berk Pharmaceuticals Ltd)
34950	Lofepramine 70mg tablets (Actavis UK Ltd)
35258	Sinepin 25mg capsules (Marlborough Pharmaceuticals Ltd)
35493	Sinepin 50mg capsules (Marlborough Pharmaceuticals Ltd)
38274	Clomipramine 50mg/5ml oral suspension
38827	Triptafen-M tablets (Mercury Pharma Group Ltd)
39145	Nortriptyline 10mg/5ml Liquid
40396	Amitriptyline 50mg Tablet (Berk Pharmaceuticals Ltd)
40777	Doxepin 25mg/5ml oral suspension
41408	Imipramine 25mg tablets (Teva UK Ltd)
41563	Clomipramine 25mg capsules (IVAX Pharmaceuticals UK Ltd)

41597	Clomipramine 50mg capsules (IVAX Pharmaceuticals UK Ltd)
41609	Trazodone 50mg capsules (Teva UK Ltd)
41627	Lofepramine 70mg Tablet (Teva UK Ltd)
41628	Clomipramine 10mg capsules (IVAX Pharmaceuticals UK Ltd)
41681	Imipramine 10mg tablets (A A H Pharmaceuticals Ltd)
41709	Trazodone 100mg capsules (Teva UK Ltd)
41710	Trazodone 100mg capsules (Zentiva)
41729	Amitriptyline 25mg Tablet (Celltech Pharma Europe Ltd)
42078	Amitriptyline 25mg tablets (Almus Pharmaceuticals Ltd)
42228	Trimipramine 10mg tablets (A A H Pharmaceuticals Ltd)
42247	Imipramine 25mg/5ml oral solution sugar free
42394	Amitriptyline 25mg Tablet (Crosspharma Ltd)
42734	Dosulepin 75mg tablets (Almus Pharmaceuticals Ltd)
43024	Dosulepin 100mg/5ml oral solution
43534	Lofepramine 70mg/5ml Oral suspension (Rosemont Pharmaceuticals Ltd)
43561	Clomipramine 10mg capsules (Teva UK Ltd)
44853	Dosulepin 25mg capsules (Kent Pharmaceuticals Ltd)
45226	Trimipramine 25mg tablets (A A H Pharmaceuticals Ltd)
45233	Amitriptyline 10mg tablets (IVAX Pharmaceuticals UK Ltd)
45242	Amitriptyline 10mg Tablet (Sussex Pharmaceutical Ltd)
45318	Clomipramine 50mg capsules (A A H Pharmaceuticals Ltd)
45350	Clomipramine 25mg capsules (Teva UK Ltd)
45737	Dosulepin 25mg/5ml Oral solution (Rosemont Pharmaceuticals Ltd)
46801	Amitriptyline 10mg/5ml oral solution
46818	Amitriptyline 10mg/5ml oral suspension
46970	Amitriptyline 50mg tablets (IVAX Pharmaceuticals UK Ltd)
47363	Mianserin 20mg Tablet (Berk Pharmaceuticals Ltd)
48065	Amitriptyline oral solution
48216	Nortriptyline 25mg tablets (A A H Pharmaceuticals Ltd)
50722	Dosulepin 25mg/5ml oral solution
51758	Prothiaden 25mg capsules (Stephar (U.K.) Ltd)
52867	Amitriptyline 10mg tablets (Accord Healthcare Ltd)
53161	Clomipramine 50mg/5ml oral solution
53187	Clomipramine 50mg capsules (Kent Pharmaceuticals Ltd)
53808	Trimipramine 10mg tablets (Phoenix Healthcare Distribution Ltd)
54877	Amitriptyline 25mg tablets (Accord Healthcare Ltd)
55137	Trazodone 150mg/5ml oral suspension
55138	Trazodone 250mg/5ml oral solution
55139	Amitriptyline 25mg tablets (Alliance Healthcare (Distribution) Ltd)
55289	Asendis 50mg tablets (Mercury Pharma Group Ltd)
55491	Amitriptyline 10mg tablets (Almus Pharmaceuticals Ltd)
55970	Nortriptyline 10mg tablets (King Pharmaceuticals Ltd)
56229	Lofepramine 70mg/5ml oral solution

56501	Tofranil 25mg tablets (Lexon (UK) Ltd)
56703	Lofepramine 70mg tablets (Sandoz Ltd)
57107	Amitriptyline 10mg tablets (Phoenix Healthcare Distribution Ltd)
57226	Trazodone 25mg/5ml oral suspension
57926	Dosulepin 75mg/5ml oral solution
57972	Amitriptyline 10mg tablets (Alliance Healthcare (Distribution) Ltd)
57978	Trimipramine 25mg tablets (Waymade Healthcare Plc)
58450	Feprapax 70mg tablets (Ashbourne Pharmaceuticals Ltd)
59161	Amitriptyline 10mg tablets (Waymade Healthcare Plc)
59820	Amitriptyline 50mg/5ml oral solution sugar free (Wockhardt UK Ltd)
59931	Trazodone 50mg/5ml oral solution sugar free (A A H Pharmaceuticals Ltd)
60355	Amitriptyline 25mg tablets (Phoenix Healthcare Distribution Ltd)
60410	Amitriptyline 25mg/5ml oral solution sugar free (Wockhardt UK Ltd)
60591	Lofepramine 70mg tablets (Teva UK Ltd)
60929	Protriptyline 5mg tablets
61657	Trazodone 75mg/5ml oral solution
61835	Amitriptyline 10mg tablets (DE Pharmaceuticals)
61842	Trazodone 50mg/5ml oral solution
62620	Clomipramine 10mg capsules (Generics (UK) Ltd)
62681	Dosulepin 75mg tablets (Sandoz Ltd)
63276	Nortriptyline 25mg tablets (Alliance Healthcare (Distribution) Ltd)

Other

Prodcode	Product name
301	Venlafaxine 37.5mg tablets
470	Venlafaxine 75mg modified-release capsules
600	Flupentixol 1mg tablets
623	Efexor 37.5mg tablets (Wyeth Pharmaceuticals)
742	Mirtazapine 30mg tablets
1222	Venlafaxine 75mg tablets
1474	Efexor XL 75mg capsules (Pfizer Ltd)
2275	Flupentixol 500microgram tablets
2356	Reboxetine 4mg tablets
2617	Venlafaxine 50mg tablets
2654	Venlafaxine 150mg modified-release capsules
3353	L-TRYPTOPHAN 500 MG CAP
3951	Fluanxol 1mg tablets (Lundbeck Ltd)
3953	Fluanxol 500microgram tablets (Lundbeck Ltd)
3954	OPTIMAX TAB
4422	Tryptophan 500mg tablets
4726	Zispin 30mg tablets (Organon Laboratories Ltd)
5611	Optimax 500mg tablets (Merck Serono Ltd)

5710	Efexor XL 150mg capsules (Pfizer Ltd)
6274	Efexor 50mg tablets (Wyeth Pharmaceuticals)
6421	Mirtazapine 15mg orodispersible tablets
6481	Mirtazapine 45mg orodispersible tablets
6488	Mirtazapine 30mg orodispersible tablets
6795	Mirtazapine 15mg tablets
6846	Zispin SolTab 15mg orodispersible tablets (Merck Sharp & Dohme Ltd)
6854	Mirtazapine 45mg tablets
6895	Duloxetine 60mg gastro-resistant capsules
7122	Duloxetine 30mg gastro-resistant capsules
8844	Tryptophan with ascorbic acid and pyridoxine powder
8845	OPTIMAX 6 GM POW
9182	Efexor 75mg tablets (Wyeth Pharmaceuticals)
9534	Nefazodone Starter pack
10083	Zispin SolTab 30mg orodispersible tablets (Merck Sharp & Dohme Ltd)
12221	Pacitron 500mg Tablet (Rorer Pharmaceuticals Ltd)
13151	Cymbalta 30mg gastro-resistant capsules (Eli Lilly and Company Ltd)
13237	Venlafaxine 37.5mg/5ml oral suspension
14849	Cymbalta 60mg gastro-resistant capsules (Eli Lilly and Company Ltd)
15163	Edronax 4mg tablets (Pfizer Ltd)
15268	Zispin SolTab 45mg orodispersible tablets (Merck Sharp & Dohme Ltd)
16154	Mirtazapine 15mg/ml oral solution sugar free
20504	Optimax wv Tablet (E. Merck)
27565	FLUANXOL
31168	MIRTAZAPINE
33337	Mirtazapine 45mg tablets (A A H Pharmaceuticals Ltd)
39359	Venlafaxine 75mg modified-release tablets
39360	Venlafaxine 150mg modified-release tablets
39770	Tifaxin XL 75mg capsules (Genus Pharmaceuticals Ltd)
39809	Tifaxin XL 150mg capsules (Genus Pharmaceuticals Ltd)
40048	ViePax XL 75mg tablets (Dexcel-Pharma Ltd)
40049	ViePax XL 150mg tablets (Dexcel-Pharma Ltd)
40054	Venlafaxine 225mg modified-release tablets
40059	Venlalic XL 75mg tablets (DB Ashbourne Ltd)
40062	Venlalic XL 150mg tablets (DB Ashbourne Ltd)
40092	Vensir XL 150mg capsules (Morningside Healthcare Ltd)
40160	Mirtazapine 30mg tablets (Actavis UK Ltd)
40277	Vensir XL 75mg capsules (Morningside Healthcare Ltd)
40295	Valdoxan 25mg tablets (Servier Laboratories Ltd)
40407	Venlalic XL 225mg tablets (DB Ashbourne Ltd)
40494	Agomelatine 25mg tablets
40514	Venaxx XL 150mg capsules (AMCo)
40515	Venaxx XL 75mg capsules (AMCo)

40517	Vexarin XL 150mg capsules (Generics (UK) Ltd)
40764	ViePax 37.5mg tablets (Dexcel-Pharma Ltd)
40815	Tardcaps XL 75mg capsules (IXL Pharma Ltd)
40817	Tardcaps XL 150mg capsules (IXL Pharma Ltd)
40917	ViePax 75mg tablets (Dexcel-Pharma Ltd)
41033	Rodomel XL 75mg capsules (Teva UK Ltd)
41299	Politid XL 75mg capsules (Actavis UK Ltd)
41314	Rodomel XL 150mg capsules (Teva UK Ltd)
42600	Vexarin XL 75mg capsules (Generics (UK) Ltd)
43203	Venlafaxine 75mg modified-release capsules (Sandoz Ltd)
43234	Mirtazapine 45mg orodispersible tablets (Teva UK Ltd)
43235	Mirtazapine 45mg orodispersible tablets (A A H Pharmaceuticals Ltd)
43236	Mirtazapine 45mg orodispersible tablets (Actavis UK Ltd)
43237	Mirtazapine 15mg orodispersible tablets (Teva UK Ltd)
43239	Mirtazapine 15mg tablets (A A H Pharmaceuticals Ltd)
43241	Mirtazapine 15mg orodispersible tablets (Aurobindo Pharma Ltd)
43242	Mirtazapine 15mg tablets (Genus Pharmaceuticals Ltd)
43246	Mirtazapine 15mg orodispersible tablets (Genus Pharmaceuticals Ltd)
43247	Mirtazapine 45mg orodispersible tablets (Genus Pharmaceuticals Ltd)
43248	Mirtazapine 15mg orodispersible tablets (Focus Pharmaceuticals Ltd)
43250	Mirtazapine 30mg orodispersible tablets (A A H Pharmaceuticals Ltd)
43253	Mirtazapine 15mg orodispersible tablets (A A H Pharmaceuticals Ltd)
43256	Mirtazapine 45mg orodispersible tablets (Focus Pharmaceuticals Ltd)
43257	Mirtazapine 15mg tablets (Teva UK Ltd)
43334	Venlafaxine 150mg modified-release capsules (Sandoz Ltd)
43673	Politid XL 150mg capsules (Actavis UK Ltd)
43968	Foraven XL 75mg capsules (Forum Products Ltd)
44936	Venlaneo XL 150mg capsules (Kent Pharmaceuticals Ltd)
44937	Venlaneo XL 75mg capsules (Kent Pharmaceuticals Ltd)
45664	Depefex XL 150mg capsules (Chiesi Ltd)
45806	Venlafaxine 37.5mg modified-release tablets
45818	Venlalic XL 37.5mg tablets (DB Ashbourne Ltd)
45959	Depefex XL 75mg capsules (Chiesi Ltd)
46668	Mirtazapine 15mg tablets (Arrow Generics Ltd)
47945	Mirtazapine 30mg tablets (A A H Pharmaceuticals Ltd)
47966	Mirtazapine 15mg/ml oral solution sugar free (Rosemont Pharmaceuticals Ltd)
48185	Mirtazapine 30mg orodispersible tablets (Almus Pharmaceuticals Ltd)
48199	Ranfaxine XL 75mg capsules (Ranbaxy (UK) Ltd)
48698	Mirtazapine 15mg orodispersible tablets sugar free
49511	Venlablue XL 75mg capsules (Bluefish Pharmaceuticals AB)
49820	Mirtazapine 45mg orodispersible tablets sugar free
50081	Venlablue XL 150mg capsules (Bluefish Pharmaceuticals AB)
50592	Fluanxol 1mg tablets (Sigma Pharmaceuticals Plc)

50892	Zispin SolTab 15mg orodispersible tablets (Necessity Supplies Ltd)
50934	Venlafaxine 150mg/5ml oral solution
51280	Efexor XL 150mg capsules (Waymade Healthcare Plc)
51361	Venlafaxine 37.5mg tablets (Ranbaxy (UK) Ltd)
51383	Duloxetine 60mg gastro-resistant capsules (Sigma Pharmaceuticals Plc)
51699	Venlafaxine 37.5mg/5ml oral solution
52074	Alventa XL 75mg capsules (Consilient Health Ltd)
52516	Alventa XL 150mg capsules (Consilient Health Ltd)
52716	Tonpular XL 75mg capsules (Wockhardt UK Ltd)
53321	Mirtazapine 15mg/ml oral solution sugar free (A A H Pharmaceuticals Ltd)
53326	Venlafaxine 75mg/5ml oral solution
53543	Zispin SolTab 30mg orodispersible tablets (Necessity Supplies Ltd)
53648	Mirtazapine 30mg orodispersible tablets (Actavis UK Ltd)
53699	Mirtazapine 15mg tablets (Actavis UK Ltd)
54012	Mirtazapine 15mg orodispersible tablets sugar free (Sandoz Ltd)
54342	Mirtazapine 15mg tablets (Medreich Plc)
54644	Mirtazapine 15mg tablets (Pfizer Ltd)
54686	Tryptophan 500mg capsules
54747	Optimax 500mg capsules (Merck Serono Ltd)
54792	Mirtazapine 30mg tablets (Alliance Healthcare (Distribution) Ltd)
55424	Venlafaxine
55482	Mirtazapine 15mg orodispersible tablets (Generics (UK) Ltd)
55501	Venlafaxine 150mg Modified-release capsule (Hillcross Pharmaceuticals Ltd)
56209	Mirtazapine 30mg tablets (Phoenix Healthcare Distribution Ltd)
56457	Venlafaxine 75mg tablets (Teva UK Ltd)
56662	Venlafaxine 37.5mg tablets (A A H Pharmaceuticals Ltd)
57751	Tonpular XL 150mg capsules (Wockhardt UK Ltd)
58291	Mirtazapine 15mg orodispersible tablets (Pfizer Ltd)
58625	Mirtazapine 45mg tablets (Actavis UK Ltd)
58681	Venladex XL 75mg tablets (Dexcel-Pharma Ltd)
58726	Venladex XL 150mg tablets (Dexcel-Pharma Ltd)
58837	Venlafaxine 37.5mg modified-release capsules
59035	Venlablue XL 37.5mg capsules (Bluefish Pharmaceuticals AB)
59563	Venlafaxine 75mg modified-release capsules (Kent Pharmaceuticals Ltd)
59593	Fluanxol 500microgram tablets (Lexon (UK) Ltd)
59694	Mirtazapine 30mg orodispersible tablets (Phoenix Healthcare Distribution Ltd)
59753	Sunveniz XL 150mg tablets (Sun Pharmaceuticals UK Ltd)
59923	Venlafaxine 37.5mg tablets (Bristol Laboratories Ltd)
59953	Mirtazapine 15mg tablets (Almus Pharmaceuticals Ltd)
59954	Mirtazapine 45mg tablets (Almus Pharmaceuticals Ltd)
60370	Zispin SolTab 15mg orodispersible tablets (Mawdsley-Brooks & Company Ltd)
60449	Venlafaxine 75mg tablets (A A H Pharmaceuticals Ltd)
60538	Mirtazapine 30mg tablets (DE Pharmaceuticals)

60549	Venlafaxine 150mg modified-release capsules (Kent Pharmaceuticals Ltd)
60843	Sunveniz XL 75mg tablets (Sun Pharmaceuticals UK Ltd)
60895	Venlafaxine 37.5mg tablets (Teva UK Ltd)
61236	Bonilux XL 150mg capsules (Sandoz Ltd)
61547	Mirtazapine 15mg/ml oral solution sugar free (DE Pharmaceuticals)
61856	Mirtazapine 15mg orodispersible tablets (Consilient Health Ltd)
62688	Duloxetine 30mg gastro-resistant capsules (Sigma Pharmaceuticals Plc)
62734	Venlafaxine 150mg/5ml oral suspension
63216	Cymbalta 60mg gastro-resistant capsules (Mawdsley-Brooks & Company Ltd)
63268	Venlafaxine 75mg/5ml oral suspension
63370	Duloxetine 30mg gastro-resistant capsules (Mawdsley-Brooks & Company Ltd)
63403	Mirtazapine 30mg tablets (Teva UK Ltd)
63763	Duloxetine 60mg gastro-resistant capsules (A A H Pharmaceuticals Ltd)
63859	Venlafaxine 75mg tablets (Waymade Healthcare Plc)

Rheumatoid arthritis

Medcode	Readterm
844	Rheumatoid arthritis
4186	Juvenile rheumatoid arthritis - Still's disease
5723	Rheumatoid nodule
6916	Seronegative rheumatoid arthritis
8350	Flare of rheumatoid arthritis
9707	Seropositive errosive rheumatoid arthritis
9954	Rheumatoid lung
12019	Seropositive rheumatoid arthritis, unspecified
18155	Rheumatoid bursitis
21358	Rheumatoid arthritis of shoulder
21533	Pauciarticular juvenile rheumatoid arthritis
23552	Felty's syndrome
23834	Adult Still's Disease
27557	Juvenile rheumatoid arthritis NOS
28853	Fibrosing alveolitis associated with rheumatoid arthritis
30548	Rheumatoid vasculitis
31054	Rheumatoid arthritis - multiple joint
31209	Myopathy due to rheumatoid arthritis
31360	Juvenile rheumatoid arthritis
31724	Rheumatoid lung
32001	Adult-onset Still's disease
33264	O/E-hands-rheumatoid spindling
36276	Monarticular juvenile rheumatoid arthritis
37431	Rheumatoid arthropathy + visceral/systemic involvement NOS
41941	Rheumatoid arthritis of PIP joint of finger
42299	Rheumatoid arthritis of MCP joint
43816	Rheumatoid carditis
44203	Other rheumatoid arthritis of spine
44743	Rheumatoid arthritis of cervical spine
46436	Rheumatoid lung disease
47831	Acute polyarticular juvenile rheumatoid arthritis
48832	Rheumatoid arthritis of wrist
49067	Rheumatoid arthritis of hip
49227	Other rheumatoid arthropathy + visceral/systemic involvement
49787	Rheumatoid myocarditis
50644	Juvenile rheumatoid arthropathy unspecified
50863	Rheumatoid arthritis of knee
51238	Rheumatoid arthritis of 1st MTP joint
51239	Rheumatoid arthritis of ankle
53621	Rheumatoid nodule

56202	[X]Seropositive rheumatoid arthritis, unspecified
56838	Caplan's syndrome
59738	Rheumatoid arthritis of elbow
62401	Polyneuropathy in rheumatoid arthritis
63198	Rheumatoid arthritis of DIP joint of finger
63365	Rheumatoid arthritis of distal radio-ulnar joint
70221	[X]Other specified rheumatoid arthritis
70658	Rheumatoid arthritis of talonavicular joint
71784	Rheumatoid arthritis of other tarsal joint
73619	Rheumatoid arthritis of subtalar joint
93715	[X]Other seropositive rheumatoid arthritis
99414	Rheumatoid arthritis of lesser MTP joint
100776	Rheumatoid arthritis of sacro-iliac joint
100914	Rheumatoid arthritis of acromioclavicular joint
106440	[X]Rheumatoid arthritis+involvement/other organs or systems
107112	Rheumatoid arthritis of IP joint of toe
107791	Rheumatoid arthritis of tibio-fibular joint
107963	Rheumatoid arthritis of sternoclavicular joint
27603	Rheumatoid arthritis and other inflammatory polyarthropathy
6639	H/O: rheumatoid arthritis
102088	Delivery of rehabilitation for rheumatoid arthritis
105507	Rheumatoid arthritis annual review

icd	icd_description
I52.8	Rheumatoid carditis
J99.0	Rheumatoid lung disease
M05	Seropositive rheumatoid arthritis
M05.0	Felty's syndrome
M05.1	Rheumatoid lung disease
M05.2	Rheumatoid vasculitis
M05.3	Rheumatoid arthritis with involvement of oth organs and sys
M05.8	Other seropositive rheumatoid arthritis
M05.9	Seropositive rheumatoid arthritis, unspecified
M06.0	Seronegative rheumatoid arthritis
M06.1	Adult-onset Still's disease
M06.2	Rheumatoid bursitis
M06.3	Rheumatoid nodule
M06.8	Other specified rheumatoid arthritis
M06.9	Rheumatoid arthritis, unspecified
M08.0	Juvenile rheumatoid arthritis
M08.2	Juvenile arthritis with systemic onset

M08.3	Juvenile polyarthritis (seronegative)
M08.4	Pauciarticular juvenile arthritis

Lupus erythematosus

Medcode	Readterm
4125	Lupus erythematosus
7522	Lupus erythematosus NOS
7871	Systemic lupus erythematosus
11920	Systemic lupus erythematosus with pericarditis
20007	Disseminated lupus erythematosus
22205	Lupus nephritis
25390	Subacute cutaneous lupus erythematosus
29519	Systemic lupus erythematosus with organ or sys involv
31564	Lung disease with systemic lupus erythematosus
33449	Lupus erythematosus chronicus
36942	Drug-induced systemic lupus erythematosus
40797	Lupus erythematosus migrans
42719	Systemic lupus erythematosus NOS
44095	Polyneuropathy in disseminated lupus erythematosus
44984	Lupus erythematosus tumidus
46148	Lupus erythematosus profundus
47672	Nephrotic syndrome in systemic lupus erythematosus
51798	Systemic lupus activity measure
58706	[X]Other forms of systemic lupus erythematosus
63955	Lupus erythematosus unguium mutilans
65391	Lupus erythematosus nodularis
99435	Neonatal lupus erythematosus
101433	Cerebral lupus
41148	Renal tubulo-interstitial disorder in SLE
57675	Libman-Sacks disease
108072	Myopathy due to disseminated lupus erythematosus

icd	icd_description
L93.1	Subacute cutaneous lupus erythematosus
L93.2	Other local lupus erythematosus
M32	Systemic lupus erythematosus
M32.0	Drug-induced systemic lupus erythematosus
M32.1	Systemic lupus erythematosus with organ or sys involv
M32.8	Other forms of systemic lupus erythematosus
M32.9	Systemic lupus erythematosus, unspecified

Inflammatory bowel disease

Medcode	Readterm
593	Crohn's disease
704	Ulcerative colitis
1784	Ulcerative colitis and/or proctitis
1796	Inflammatory bowel disease
5133	Idiopathic proctocolitis
5749	H/O: ulcerative colitis
6538	Crohn's colitis
6650	Ulcerative proctocolitis
8347	Ulcerative proctitis
9359	Crohn's disease of the small bowel NOS
11286	Regional enteritis - Crohn's disease
12575	Juvenile arthritis in Crohn's disease
15207	Idiopathic proctocolitis NOS
15773	Regional ileocolitis
17641	Arthropathy in ulcerative colitis
20480	Arthropathy in Crohn's disease
20688	Crohn's disease of the large bowel NOS
22516	Exacerbation of ulcerative colitis
24550	Other idiopathic proctocolitis
24858	Ulcerative rectosigmoiditis
28476	Crohn's disease of the terminal ileum
29616	Orofacial Crohn's disease
30433	Ulcerative (chronic) enterocolitis
33456	Ulcerative proctocolitis NOS
36913	Exacerbation of Crohn's disease of small intestine
39037	Exacerbation of Crohn's disease of large intestine
39278	Crohn's disease of the ileum NOS
42822	Ulcerative (chronic) ileocolitis
43090	Other idiopathic proctocolitis NOS
44426	Regional enteritis of the large bowel
48732	Ulcerative ileocolitis
51576	Regional enteritis of the small bowel
51578	Granulomatous enteritis
52449	Regional enteritis NOS
53743	[X]Other ulcerative colitis
59994	Crohn's disease NOS
62628	Regional enteritis of the colon
63036	Regional enteritis of the jejunum
64773	Regional enteritis of the rectum
66238	Crohn's disease of the ileum unspecified

69959	[X]Other Crohn's disease
71083	Juvenile arthritis in ulcerative colitis
71945	Regional enteritis of the duodenum
107313	Management of inflammatory bowel disease

icd	icd_description
K50	Crohn's disease [regional enteritis]
K50.0	Crohn's disease of small intestine
K50.1	Crohn's disease of large intestine
K50.8	Other Crohn's disease
K50.9	Crohn's disease, unspecified
K51	Ulcerative colitis
K51.0	Ulcerative (chronic) enterocolitis
K51.1	Ulcerative (chronic) ileocolitis
K51.2	Ulcerative (chronic) proctitis
K51.3	Ulcerative (chronic) rectosigmoiditis
K51.4	Pseudopolyposis of colon
K51.5	Mucosal proctocolitis
K51.8	Other ulcerative colitis
K51.9	Ulcerative colitis, unspecified
M07.4	Arthropathy in Crohn's disease [regional enteritis]
M07.5	Arthropathy in ulcerative colitis
M09.1	Juvenile arthritis in Crohn's disease [reg enteritis]
M09.2	Juvenile arthritis in ulcerative colitis

Chronic obstructive pulmonary disease

Medcode	Readterm
794	emphysema
998	chronic obstructive airways disease
1001	chronic obstructive pulmonary disease
1446	acute exacerbation of chronic obstructive airways disease
3243	chronic bronchitis
4084	Airways obstructn irreversible
5710	chronic obstructive airways disease nos
5798	Chronic asthmatic bronchitis
5909	Chronic wheezy bronchitis
7884	chron obstruct pulmonary dis wth acute exacerbation, unspec
9876	severe chronic obstructive pulmonary disease
10802	moderate chronic obstructive pulmonary disease
10863	mild chronic obstructive pulmonary disease
10980	centrilobular emphysema
11019	admit copd emergency
11150	Mucopurulent chronic bronchitis
11287	chronic obstructive pulmonary disease annual review
12166	other specified chronic obstructive airways disease
14798	emphysematous bronchitis
15157	chronic bronchitis nos
15626	chronic catarrhal bronchitis
16410	Other emphysema NOS
18476	copd follow-up
18501	copd self-management plan given
18621	chronic obstructive pulmonary disease follow-up
19003	emergency copd admission since last appointment
19106	copd accident and emergency attendance since last visit
21061	chronic obstruct pulmonary dis with acute lower resp infectn
23492	chronic bullous emphysema nos
23618	Chronic tracheitis
24248	mixed simple and mucopurulent chronic bronchitis
25603	simple chronic bronchitis
26306	chronic bullous emphysema
27819	obstructive chronic bronchitis
28743	Number of COPD exacerbations in past year
33450	emphysema nos
37247	chronic obstructive pulmonary disease nos
37959	fetid chronic bronchitis
40159	purulent chronic bronchitis
40788	Other emphysema

42624	coad follow-up
44525	obstructive chronic bronchitis nos
45089	Chronic tracheobronchitis
45770	chronic obstructive pulmonary disease disturbs sleep
45771	chronic obstructive pulmonary disease does not disturb sleep
45777	chronic obstructive pulmonary disease clini management plan
46036	multiple copd emergency hospital admissions
46578	panlobular emphysema
56860	segmental bullous emphysema
60188	giant bullous emphysema
61118	simple chronic bronchitis nos
61513	mucopurulent chronic bronchitis nos
63479	MacLeod's unilateral emphysema
65733	[x]other specified chronic obstructive pulmonary disease
66043	other chronic bronchitis
66058	[X]Other emphysema
67040	other specified chronic obstructive pulmonary disease
68066	other chronic bronchitis nos
68662	Zonal bullous emphysema
93568	very severe chronic obstructive pulmonary disease
98283	copd structured smoking assessment declined - enh serv admin
98284	refer copd structured smoking assessment - enhanc serv admin
99536	bullous emphysema with collapse
99948	copd patient unsuitable for pulmonary rehab - enh serv admin
101042	issue of chronic obstructive pulmonary disease rescue pack
102685	chronic obstructive pulmonary disease 3 monthly review
103007	chronic obstructive pulmonary disease 6 monthly review
103400	referred for copd structured smoking assessment
103494	History of chronic obstructive pulmonary disease
103558	Preferred place of care for next exacerbation of COPD
103678	Chronic obstructiv pulmonary disease medication optimisation
103864	COPD patient unsuitable for pulmonary rehabilitation
104117	COPD self-management plan agreed
104169	COPD self-management plan review
104265	GP OOH service notified of COPD care plan
104481	Has chronic obstructive pulmonary disease care plan
104608	End stage chronic obstructive airways disease
104710	On COPD (chr obstruc pulmonary disease) supportv cre pathway
104985	On chronic obstructive pulmonary disease supprtv cre pathway
104998	Chronic obstructve pulmonry disease rescue pack not indicatd
105457	Chronic obstructive pulmonary disease care pathway
106805	Chronic asthma with fixed airflow obstruction
106945	Chronic obstructive pulmonary disease rescue pack declined

icd	icd_description
J41	Simple and mucopurulent chronic bronchitis
J41.0	Simple chronic bronchitis
J41.1	Mucopurulent chronic bronchitis
J41.8	Mixed simple and mucopurulent chronic bronchitis
J42	Unspecified chronic bronchitis
J43	Emphysema
J43.0	MacLeod's syndrome
J43.1	Panlobular emphysema
J43.2	Centrilobular emphysema
J43.8	Other emphysema
J43.9	Emphysema, unspecified
J44	Other chronic obstructive pulmonary disease
J44.0	Chronic obstruct pulmonary dis with acute lower resp infec
J44.1	Chron obstruct pulmonary dis wth acute exacerbation, unspec
J44.8	Other specified chronic obstructive pulmonary disease
J44.9	Chronic obstructive pulmonary disease, unspecified

Asthma

Medcode	Readterm
78	asthma
185	acute exacerbation of asthma
232	asthma attack
233	severe asthma attack
1208	childhood asthma
1555	bronchial asthma
2290	allergic asthma
3018	mild asthma
3366	severe asthma
3458	occasional asthma
3665	late onset asthma
4442	asthma unspecified
4606	exercise induced asthma
4836	nocturnal cough / wheeze
4892	status asthmaticus nos
5267	intrinsic asthma
5627	hay fever with asthma
5867	exercise induced asthma
6707	extrinsic asthma with asthma attack
7058	emergency admission, asthma
7146	extrinsic (atopic) asthma
7191	asthma limiting activities
7229	asthma prophylactic medication used
7416	asthma disturbing sleep
7731	pollen asthma
8335	asthma attack nos
9018	number of asthma exacerbations in past year
10487	asthma - currently active
11022	asthma trigger
11370	asthma confirmed
12987	late-onset asthma
13064	asthma severity
13065	moderate asthma
13173	asthma not disturbing sleep
13174	asthma not limiting activities
13175	asthma disturbs sleep frequently
14777	extrinsic asthma without status asthmaticus
15248	hay fever with asthma
16070	asthma nos
16667	asthma control step 2

16785	asthma control step 1
18224	asthma control step 3
18323	intrinsic asthma with asthma attack
19519	asthma treatment compliance unsatisfactory
19520	asthma treatment compliance satisfactory
20860	asthma control step 5
20886	asthma control step 4
21232	allergic asthma nec
22752	occupational asthma
24479	emergency asthma admission since last appointment
24506	further asthma - drug prevent.
24884	asthma causes daytime symptoms 1 to 2 times per week
25181	asthma restricts exercise
25796	mixed asthma
26501	asthma never causes daytime symptoms
26503	asthma causes daytime symptoms most days
26504	asthma never restricts exercise
26506	asthma severely restricts exercise
26861	asthma sometimes restricts exercise
27926	extrinsic asthma with status asthmaticus
29325	intrinsic asthma without status asthmaticus
29645	asthma control step 0
30815	asthma causing night waking
31167	asthma night-time symptoms
31225	asthma causes daytime symptoms 1 to 2 times per month
38143	asthma never disturbs sleep
38144	asthma limits walking up hills or stairs
38145	asthma limits walking on the flat
38146	asthma disturbs sleep weekly
39478	wood asthma
39570	asthma causes night symptoms 1 to 2 times per month
40823	brittle asthma
41017	aspirin induced asthma
41020	absent from work or school due to asthma
42824	asthma daytime symptoms
45073	intrinsic asthma nos
45782	extrinsic asthma nos
47337	asthma accident and emergency attendance since last visit
47684	detergent asthma
58196	intrinsic asthma with status asthmaticus
73522	work aggravated asthma
102301	asthma trigger - seasonal
102341	asthma trigger - pollen

102395	asthma causes symptoms most nights
102400	asthma causes night time symptoms 1 to 2 times per week
102449	asthma trigger - respiratory infection
102713	asthma limits activities 1 to 2 times per month
102871	asthma trigger - exercise
102888	asthma limits activities 1 to 2 times per week
102952	asthma trigger - warm air
103321	asthma trigger - animals
32727	Hyperreactive airways disease
103612	Asthma never causes night symptoms
103813	Asthma trigger - cold air
103944	Asthma trigger - airborne dust
103945	Asthma trigger - damp
103952	Asthma trigger - emotion
103955	Asthma trigger - tobacco smoke
103998	Asthma limits activities most days
93353	Sequoiosis (red-cedar asthma)
9552	change in asthma management plan
9663	step up change in asthma management plan
10043	asthma annual review
10274	asthma medication review
13176	asthma follow-up
47993	Does not have asthma management plan
105420	Asthma self-management plan review
105674	Asthma self-management plan agreed
107167	Number days absent from school due to asthma in past 6 month
99793	patient has a written asthma personal action plan
7378	asthma management plan given
18223	step down change in asthma management plan
25791	asthma clinical management plan

icd	icd_description
J45	Asthma
J45.0	Predominantly allergic asthma
J45.1	Nonallergic asthma
J45.8	Mixed asthma
J45.9	Asthma, unspecified
J46	Status asthmaticus

Antiasthmatics

Prodcode	Product name
17894	accolate
1973	accolate tablets 20mg [astrazenec]
1861	aerobec autohaler 100micrograms/actuation [meda]
2159	aerobec autohaler 50micrograms/actuation [meda]
4499	aerobec forte autohaler 250micrograms/actuation [meda]
39200	aerobec forte autohaler 250micrograms/actuation [meda]
10360	aerocrom aerosol inhaler [castlemead]
18314	aerocrom syncroner [castlemead]
34134	aerolin 400 aerosol inhaler 100micrograms/actuation [3m]
20680	aerolin autohaler
942	aerolin autohaler breath actuated inhaler 100micrograms/actuation [3m]
30240	aerolin autohaler cfc free breath actuated inhaler 100micrograms/actuation [3m]
3322	aerolin inh 400 100 mcg aer
12479	aerolin inh auto refil 100 mcg aer
26654	aerolin inhaler auto refil
26716	airomir autohaler cfc free b/a
5740	airomir autohaler cfc free breath actuated inhaler 100micrograms/actuation [ivax]
2655	airomir cfc free inhaler 100micrograms/inhalation [teva]
8508	alupent aerosol inhaler 750micrograms/inhalation [boeh ingl]
8149	alupent aerosol refill 750micrograms/inhalation [boeh ingl]
12470	alupent expectorant mixture [boeh ingl]
22082	alupent expectorant tablets [boeh ingl]
2901	alupent sugar free oral solution 10mg/5ml [boeh ingl]
8321	alupent tablets 20mg [boeh ingl]
6839	alvesco cfc free inhaler 160micrograms/actuation [nycomed]
21224	alvesco cfc free inhaler 80micrograms/actuation [nycomed]
8610	aminophylline 1 gm sup
218	aminophylline 100 mg cap
18308	aminophylline 100 mg sup
25022	aminophylline 150 mg sup
20171	aminophylline 180 mg sup
27842	aminophylline 2 ml inj
22080	aminophylline 20 ml inj
10289	aminophylline 200 mg sup
8470	aminophylline 225 mg sup
15025	aminophylline 25 mg sup
24117	aminophylline 300 mg sup
27593	aminophylline 350 mg sup
8653	aminophylline 360 mg sup
20225	aminophylline 500 mg inj

19350	aminophylline 62.5 mg sup
6988	aminophylline hydrate modified release tablet 100mg
17002	aminophylline hydrate modified release tablet 225mg
16994	aminophylline hydrate modified release tablet 350mg
14991	aminophylline injection 250mg/10ml
42910	aminophylline injection 250mg/10ml [martindale]
10561	aminophylline injection 250mg/ml
42511	aminophylline injection 25mg/ml [celltech]
25937	aminophylline intramuscular 500 mg inj
8057	aminophylline modified release tablet 100mg
555	aminophylline modified release tablet 225mg
30596	aminophylline modified release tablet 225mg [actavis]
29273	aminophylline modified release tablet 225mg [hillcross]
4514	aminophylline modified release tablet 350mg
23572	aminophylline sr tablets 225mg [ivax]
25125	aminophylline suppository 360mg
8056	aminophylline tablets 100mg
17140	aminophylline tablets 200mg
13529	amnivent sustained release tablets 225mg [ashbourne]
4601	asmabec clickhaler dry powder inhaler 100micrograms [focus]
14567	asmabec clickhaler dry powder inhaler 250micrograms [focus]
9577	asmabec clickhaler dry powder inhaler 50micrograms [focus]
9477	asmabec spacehaler 100micrograms/actuation [celltech]
14590	asmabec spacehaler 250micrograms/actuation [celltech]
19389	asmabec spacehaler 50micrograms/actuation [celltech]
16433	asmanex twisthaler dry powder inhaler 200micrograms/actuation [m s d]
17590	asmanex twisthaler dry powder inhaler 400micrograms/actuation [m s d]
1087	asmasal clickhaler dry powder inhaler 95micrograms [focus]
9651	asmasal spacehaler 100micrograms/inhalation [celltech]
21859	asmaven aerosol inhaler 100micrograms [berk]
25784	atimos modulite cfc free inhaler 12micrograms/actuation [chiesi]
19805	atrovent
2994	atrovent aerocaps 40mcg [boeh ingl]
43090	atrovent aerocaps inhalation powder capsules 40mcg [boeh ingl]
9681	atrovent aerohaler 40mcg [boeh ingl]
43105	atrovent aerohaler inhalation powder capsules with device 40mcg [boeh ingl]
534	atrovent aerosol inhaler 20micrograms/actuation [boeh ingl]
1697	atrovent autohaler breath actuated inhaler 20micrograms/actuation [boeh ingl]
6512	atrovent cfc free inhaler 20micrograms/actuation [boeh ingl]
20720	atrovent forte
3306	atrovent forte aerosol inhaler 40micrograms/actuation [boeh ingl]
19737	atrovent nebuliser solution (1ml vial)
1962	atrovent udvs nebuliser solution 0.25mg/ml [boeh ingl]

6911	atrovent udvs nebuliser solution 250micrograms/1ml [boeh ingl]
7140	atrovent udvs nebuliser solution 500micrograms/2ml [boeh ingl]
14527	bambec tablets 10mg [astrazenec]
13575	bambec tablets 20mg [astrazenec]
7192	bambuterol tablets 10mg
12144	bambuterol tablets 20mg
19031	bdp spacehaler 100micrograms/actuation [celltech]
14524	bdp spacehaler 250micrograms/actuation [celltech]
18394	bdp spacehaler 50micrograms/actuation [celltech]
13815	beclazone aerosol inhaler 100micrograms/actuation [actavis]
1100	beclazone aerosol inhaler 100micrograms/actuation [ivax]
1885	beclazone aerosol inhaler 200micrograms/actuation [ivax]
4803	beclazone aerosol inhaler 250micrograms/actuation [actavis]
1551	beclazone aerosol inhaler 250micrograms/actuation [ivax]
9599	beclazone aerosol inhaler 50micrograms/actuation [actavis]
2992	beclazone aerosol inhaler 50micrograms/actuation [ivax]
895	beclazone easi-breathe breath actuated inhaler 100micrograms/actuation [ivax]
1243	beclazone easi-breathe breath actuated inhaler 250micrograms/actuation [ivax]
1725	beclazone easi-breathe breath actuated inhaler 50micrograms/actuation [ivax]
20763	becloforte
1236	becloforte aerosol inhaler 250micrograms/actuation [a & h]
3363	becloforte diskhaler 400micrograms/actuation [a & h]
2892	becloforte disks (refill pack) 400micrograms/actuation [a & h]
1552	becloforte easi-breathe breath actuated inhaler 250micrograms/actuation [a & h]
3119	becloforte integra inhaler with compact spacer 250micrograms/actuation [glaxo]
8111	becloforte vm pack 250micrograms/actuation [a & h]
38	beclometasone aerosol inhaler 100micrograms/actuation
28640	beclometasone aerosol inhaler 100micrograms/actuation [actavis]
26063	beclometasone aerosol inhaler 100micrograms/actuation [aps]
21482	beclometasone aerosol inhaler 100micrograms/actuation [gen (uk)]
25204	beclometasone aerosol inhaler 100micrograms/actuation [hillcross]
33849	beclometasone aerosol inhaler 100micrograms/actuation [neolab]
1259	beclometasone aerosol inhaler 200micrograms/actuation
34794	beclometasone aerosol inhaler 200micrograms/actuation [hillcross]
1242	beclometasone aerosol inhaler 250micrograms/actuation
34315	beclometasone aerosol inhaler 250micrograms/actuation [actavis]
30210	beclometasone aerosol inhaler 250micrograms/actuation [aps]
29325	beclometasone aerosol inhaler 250micrograms/actuation [gen (uk)]
33258	beclometasone aerosol inhaler 250micrograms/actuation [hillcross]
34859	beclometasone aerosol inhaler 250micrograms/actuation [neolab]
41412	beclometasone aerosol inhaler 400micrograms/actuation
3018	beclometasone aerosol inhaler 50micrograms/actuation
32874	beclometasone aerosol inhaler 50micrograms/actuation [actavis]

34739	beclometasone aerosol inhaler 50micrograms/actuation [aps]
31774	beclometasone aerosol inhaler 50micrograms/actuation [gen (uk)]
34919	beclometasone aerosol inhaler 50micrograms/actuation [hillcross]
34428	beclometasone aerosol inhaler 50micrograms/actuation [neolab]
1734	beclometasone breath actuated inhaler 100micrograms/actuation
27679	beclometasone breath actuated inhaler 100micrograms/actuation [aps]
2600	beclometasone breath actuated inhaler 250micrograms/actuation
28073	beclometasone breath actuated inhaler 250micrograms/actuation [aps]
2160	beclometasone breath actuated inhaler 50micrograms/actuation
30238	beclometasone breath actuated inhaler 50micrograms/actuation [aps]
4759	beclometasone capsules (for inhalation) 100micrograms
9233	beclometasone capsules (for inhalation) 200micrograms
7653	beclometasone capsules (for inhalation) 400micrograms
15326	beclometasone cfc free inhaler 100micrograms/actuation
14321	beclometasone cfc free inhaler 200micrograms/actuation
21005	beclometasone cfc free inhaler 250micrograms/actuation
16584	beclometasone cfc free inhaler 50micrograms/actuation
46157	beclometasone cyclocaps capsules (for inhalation) 200micrograms [teva]
41269	beclometasone cyclocaps capsules (for inhalation) 400micrograms [teva]
4365	beclometasone disc 100micrograms
2893	beclometasone disc 200micrograms
2148	beclometasone disc 400micrograms
5522	beclometasone dry powder inhaler 100micrograms/actuation
5521	beclometasone dry powder inhaler 200micrograms/actuation
5804	beclometasone dry powder inhaler 250micrograms/actuation
11497	beclometasone dry powder inhaler 400micrograms/actuation
5992	beclometasone dry powder inhaler 50micrograms/actuation
9921	beclometasone extrafine particle cfc free breath actuated inhaler 100micrograms/actuation
11732	beclometasone extrafine particle cfc free breath actuated inhaler 50micrograms/actuation
3150	beclometasone extrafine particle cfc free inhaler 100micrograms/actuation
10090	beclometasone extrafine particle cfc free inhaler 50micrograms/actuation
37470	beclometasone extrafine particle with formoterol cfc free inhaler 100micrograms + 6micrograms/actuation
35652	beclometasone inhalation powder blisters (refill) 100micrograms
35113	beclometasone inhalation powder blisters (refill) 200micrograms
35288	beclometasone inhalation powder blisters (refill) 400micrograms
35580	beclometasone inhalation powder blisters with device 100micrograms
35293	beclometasone inhalation powder blisters with device 200micrograms
35107	beclometasone inhalation powder blisters with device 400micrograms
19401	beclometasone inhaler with compact spacer 250micrograms/actuation
7964	beclometasone nebuliser suspension 50micrograms/ml
15706	beclometasone vortex metered dose inhaler 100micrograms/actuation
9571	beclometasone vortex metered dose inhaler 250micrograms/actuation

11198	beclometasone vortex metered dose inhaler 50micrograms/actuation
3556	beclometasone with salbutamol aerosol inhaler 50micrograms + 100micrograms/inhalation
19121	beclometasone with salbutamol capsules (for inhalation) 100micrograms + 200micrograms
19376	beclometasone with salbutamol capsules (for inhalation) 200micrograms + 400micrograms
22225	beclomethasone /salbutamol
2229	becodisks disc 100micrograms [a & h]
883	becodisks disc 200micrograms [a & h]
1951	becodisks disc 400micrograms [a & h]
35106	becodisks diskhaler inhalation powder 100micrograms [a & h]
35430	becodisks diskhaler inhalation powder 200micrograms [a & h]
35118	becodisks diskhaler inhalation powder 400micrograms [a & h]
35408	becodisks inhalation powder (refill) 100micrograms [a & h]
35071	becodisks inhalation powder (refill) 200micrograms [a & h]
35299	becodisks inhalation powder (refill) 400micrograms [a & h]
20707	becotide 100
99	becotide 100 aerosol inhaler 100micrograms/actuation [a & h]
1258	becotide 200 aerosol inhaler 200micrograms/actuation [a & h]
27525	becotide 50
1406	becotide 50 aerosol inhaler 50micrograms/actuation [a & h]
896	becotide easi-breathe breath actuated inhaler 100micrograms/actuation [a & h]
1727	becotide easi-breathe breath actuated inhaler 50micrograms/actuation [a & h]
19563	BECOTIDE FOR NEBULISER
1269	becotide nebuliser suspension 50micrograms/ml [a & h]
24219	becotide rotacaps
3947	becotide rotacaps 100micrograms [a & h]
1537	becotide rotacaps 200micrograms [a & h]
3075	becotide rotacaps 400micrograms [a & h]
9356	becotide rotahaler insufflator [a & h]
3437	becotide rotahaler type 4 insufflator [a & h]
19736	becotide susp for nebulisation
1794	berotec aerosol inhaler 100micrograms/actuation [boeh ingl]
2020	berotec aerosol inhaler 200micrograms/actuation [boeh ingl]
13365	berotec nebuliser solution 5mg/ml [boeh ingl]
22827	betamethasone .1 mg pel
21465	betamethasone .1 mg tab
25087	betamethasone /clioquinol
24205	betamethasone /neomycin
22180	betamethasone 0.1%/neomycin 0.5% een dro
29322	betamethasone loz
27970	betamethasone sod phos 0.1%/neomycin 0.5
24660	betamethasone valerate
27083	betamethasone valerate .1 mg tab
7724	betamethasone valerate aerosol inhaler 100micrograms/actuation

30805	betamethasone valerate pel
3065	bextasol aerosol inhaler [a & h]
10831	biophylline syrup 125mg/5ml [lorex]
24418	biophylline tablets 350mg [lorex]
24674	biophylline tablets 500mg [lorex]
26829	brelomax tablets 2mg [abbott]
14482	bricanyl 2.5 mg inj
235	bricanyl aerosol inhaler [astrazenec]
26987	bricanyl compound tablets [astrazenec]
15483	bricanyl expectorant [astrazenec]
35744	bricanyl injection 2.5mg/5ml [astrazenec]
35522	bricanyl injection 500micrograms/1ml [astrazenec]
13307	bricanyl injection 500micrograms/ml [astrazenec]
17901	bricanyl nebule 2.5 ml
2758	bricanyl refill canister [astrazenec]
4222	bricanyl respirator solution 10mg/ml [astrazenec]
3764	bricanyl respules (5mg/2ml) 2.5 mg/ml inh
43085	bricanyl respules unit dose nebuliser solution 5mg/2ml [astrazenec]
4541	bricanyl sa tablets 7.5mg [astrazenec]
7954	bricanyl spacer inhaler [astrazenec]
3584	bricanyl sugar free oral solution 1.5mg/5ml [astrazenec]
3534	bricanyl tablets 5mg [astrazenec]
907	bricanyl turbohaler 500micrograms [astrazenec]
42886	bricanyl turbohaler dry powder inhaler 500micrograms [astrazenec]
4640	bricanyl unit dose nebuliser solution 5mg/2ml [astrazenec]
12486	bronchodil aerosol inhaler 500micrograms/dose [viatris]
25820	bronchodil elixir 10mg/5ml [viatris]
15075	bronchodil tablets 20mg [viatris]
35724	budelin novolizer inhalation powder (refill) 200micrograms [meda]
35631	budelin novolizer inhalation powder with device 200micrograms [meda]
8433	budesonide aerosol inhaler 100micrograms/actuation
909	budesonide aerosol inhaler 200micrograms/actuation
14700	budesonide aerosol inhaler 400micrograms/actuation
959	budesonide aerosol inhaler 50micrograms/actuation
10321	budesonide capsules (for inhalation) 400micrograms
39102	budesonide cfc free inhaler 100micrograms
39879	budesonide cfc free inhaler 200micrograms
35602	budesonide dry powder inhalation cartridge (refill) 200micrograms
35510	budesonide dry powder inhalation cartridge with device 200micrograms
7788	budesonide dry powder inhaler 100micrograms/actuation
2092	budesonide dry powder inhaler 200micrograms/actuation
1642	budesonide dry powder inhaler 400micrograms/actuation
4801	budesonide nebuliser suspension 0.5mg/2ml

4942	budesonide nebuliser suspension 1mg/2ml
3570	budesonide refill canister 200micrograms/actuation
947	budesonide refill canister 50micrograms/actuation
16054	budesonide refillable breath actuated dry powder inhaler 200micrograms/actuation
10218	budesonide with formoterol dry powder inhaler 100micrograms + 6micrograms/actuation
6796	budesonide with formoterol dry powder inhaler 200micrograms + 6micrograms/actuation
6746	budesonide with formoterol dry powder inhaler 400micrograms + 12micrograms/actuation
8511	cam sugar free liquid 4mg/5ml [cambhealth]
27558	choledyl
3187	choledyl syrup 62.5mg/5ml [parke]
4591	choledyl tablets 100mg [parke]
4592	choledyl tablets 200mg [parke]
22669	choline theophyllinate 270 mg tab
18988	choline theophyllinate syrup 62.5mg/5ml
18288	choline theophyllinate tablets 100mg
7832	choline theophyllinate tablets 200mg
10102	ciclesonide cfc free inhaler 160micrograms/actuation
7356	ciclesonide cfc free inhaler 80micrograms/actuation
13290	clenil modulite cfc free inhaler 100micrograms/actuation [chiesi]
16151	clenil modulite cfc free inhaler 200micrograms/actuation [chiesi]
16148	clenil modulite cfc free inhaler 250micrograms/actuation [chiesi]
16158	clenil modulite cfc free inhaler 50micrograms/actuation [chiesi]
19732	cobutolin inh
26873	cobutolin tablets 2mg [actavis]
556	combivent aerosol inhaler 20mcg + 100mcg [boeh ingl]
3305	combivent udvs nebuliser solution 2.5ml [boeh ingl]
1422	cromogen aerosol inhaler 5mg/inhalation [ivax]
1728	cromogen easi-breathe breath actuated inhaler 5mg/inhalation [ivax]
22550	duovent
2722	duovent aerosol inhaler 40micrograms + 100micrograms/actuation [boeh ingl]
2862	duovent autohaler breath actuated inhaler [boeh ingl]
16207	duovent udvs nebuliser solution [boeh ingl]
17654	easyhaler beclometasone dry powder inhaler 200micrograms/actuation [orion]
17670	easyhaler budesonide dry powder inhaler 100micrograms/actuation [orion]
27188	easyhaler budesonide dry powder inhaler 200micrograms/actuation [orion]
30649	easyhaler budesonide dry powder inhaler 400micrograms/actuation [orion]
35725	easyhaler formoterol dry powder inhaler 12micrograms/actuation [orion]
13181	easyhaler salbutamol dry powder inhaler 100micrograms/actuation [orion]
16577	easyhaler salbutamol dry powder inhaler 200micrograms/actuation [orion]
15561	ephedrine 11mg/theophylline 120mg 11 mg tab
26420	exirel 10 mg tab
8504	exirel 15 mg tab
12563	exirel aerosol inhaler [3m]

23787	exirel capsules 10mg [3m]
8012	exirel capsules 15mg [3m]
25821	exirel syrup 7.5mg/5ml [3m]
26744	expulin decongestant sugar free linctus [shire]
4842	fenoterol aerosol inhaler 100micrograms/actuation
5185	fenoterol aerosol inhaler 200micrograms/actuation
15441	fenoterol hydrobromide .5 % sol
8339	fenoterol hydrobromide complete unit inh
3786	fenoterol with ipratropium bromide aerosol inhaler 100micrograms + 40micrograms/actuation
12808	fenoterol with ipratropium bromide breath actuated inhaler 100micrograms + 40micrograms/actuation
18299	fenoterol with ipratropium bromide unit dose nebulising solution 1.25mg + 500micrograms/4ml
3927	filair aerosol inhaler 100micrograms/actuation [meda]
3743	filair aerosol inhaler 50micrograms/actuation [meda]
3993	filair forte aerosol inhaler 250micrograms/actuation [meda]
4926	flixotide accuhaler 100micrograms/inhalation [a & h]
911	flixotide accuhaler 250micrograms/inhalation [a & h]
2440	flixotide accuhaler 500micrograms/inhalation [a & h]
5580	flixotide accuhaler 50micrograms/inhalation [a & h]
42928	flixotide accuhaler dry powder inhaler 100micrograms/inhalation [a & h]
42994	flixotide accuhaler dry powder inhaler 250micrograms/inhalation [a & h]
43074	flixotide accuhaler dry powder inhaler 500micrograms/inhalation [a & h]
42985	flixotide accuhaler dry powder inhaler 50micrograms/inhalation [a & h]
1676	flixotide aerosol inhaler 125micrograms/actuation [a & h]
1412	flixotide aerosol inhaler 250micrograms/actuation [a & h]
3289	flixotide aerosol inhaler 25micrograms/actuation [a & h]
1518	flixotide aerosol inhaler 50micrograms/actuation [a & h]
3989	flixotide disc 100micrograms [a & h]
1424	flixotide disc 250micrograms [a & h]
1426	flixotide disc 500micrograms [a & h]
8635	flixotide disc 50micrograms [a & h]
36090	flixotide diskhaler (refill) inhalation powder 100micrograms [a & h]
35611	flixotide diskhaler (refill) inhalation powder 250micrograms [a & h]
35374	flixotide diskhaler (refill) inhalation powder 500 micrograms [a & h]
35986	flixotide diskhaler (refill) inhalation powder 50micrograms [a & h]
35225	flixotide diskhaler inhalation powder 100micrograms [a & h]
35461	flixotide diskhaler inhalation powder 250micrograms [a & h]
35392	flixotide diskhaler inhalation powder 500 micrograms [a & h]
36290	flixotide diskhaler inhalation powder 50micrograms [a & h]
3988	flixotide diskhaler-community pack 100 mcg
3753	flixotide diskhaler-community pack 250 mcg
8450	flixotide diskhaler-community pack 50 mcg
5718	flixotide evohaler cfc free inhaler 125micrograms/actuation [a & h]
5683	flixotide evohaler cfc free inhaler 250micrograms/actuation [a & h]

5309	flixotide evohaler cfc free inhaler 50micrograms/actuation [a & h]
5551	flixotide nebules unit dose nebulising suspension 500micrograms/2ml [a & h]
4132	fluticasone aerosol inhaler 125micrograms/actuation
2951	fluticasone aerosol inhaler 250micrograms/actuation
2723	fluticasone aerosol inhaler 25micrograms/actuation
4688	fluticasone aerosol inhaler 50micrograms/actuation
5975	fluticasone cfc free inhaler 125micrograms/actuation
5822	fluticasone cfc free inhaler 250micrograms/actuation
5223	fluticasone cfc free inhaler 50micrograms/actuation
4131	fluticasone disc 100micrograms
7638	fluticasone disc 250micrograms
7891	fluticasone disc 500micrograms
7602	fluticasone disc 50micrograms
5885	fluticasone dry powder inhaler 100micrograms/inhalation
7948	fluticasone dry powder inhaler 250micrograms/inhalation
2282	fluticasone dry powder inhaler 500micrograms/inhalation
9164	fluticasone dry powder inhaler 50micrograms/inhalation
35772	fluticasone inhalation powder blisters (refill) 100micrograms
35905	fluticasone inhalation powder blisters (refill) 250micrograms
36462	fluticasone inhalation powder blisters (refill) 500 micrograms
37447	fluticasone inhalation powder blisters (refill) 50micrograms
35638	fluticasone inhalation powder blisters with device 100micrograms
36401	fluticasone inhalation powder blisters with device 250micrograms
35700	fluticasone inhalation powder blisters with device 500 micrograms
36021	fluticasone inhalation powder blisters with device 50micrograms
27915	fluticasone prop disk refill
11478	fluticasone unit dose nebulising suspension 2mg/2ml
17465	fluticasone unit dose nebulising suspension 500micrograms/2ml
11588	fluticasone with salmeterol cfc free inhaler 125micrograms + 25micrograms/actuation
11618	fluticasone with salmeterol cfc free inhaler 250micrograms + 25micrograms/actuation
12994	fluticasone with salmeterol cfc free inhaler 50micrograms + 25micrograms/actuation
13273	fluticasone with salmeterol dry powder inhaler 100micrograms + 50micrograms/inhalation
13040	fluticasone with salmeterol dry powder inhaler 250micrograms + 50micrograms/inhalation
11410	fluticasone with salmeterol dry powder inhaler 500micrograms + 50micrograms/inhalation
10968	foradil capsules (for inhalation) 12mcg [nov/ciba]
6526	formoterol fumarate capsules (for inhalation) 12mcg
14306	formoterol fumarate cfc free inhaler 12micrograms/actuation
7133	formoterol fumarate dry powder inhaler 12micrograms/actuation
9711	formoterol fumarate dry powder inhaler 6 micrograms/actuation
37432	fostair cfc free inhaler 100micrograms + 6micrograms/actuation [chiesi]
7477	franol plus tablets [sanofi/ave]
2609	franol tablets [sanofi s]
19597	intal

24387	intal
314	intal aerosol inhaler [aventis]
4100	intal autohaler 5mg/inhalation [aventis]
38501	intal cfc free inhaler 5mg [sanofi/ave]
2610	intal compound capsules (for inhalation) [rhone]
7972	intal fisonair aerosol inhaler 5mg/inhalation [aventis]
19643	intal nebuliser solution (2ml)
1629	intal nebuliser solution 10mg/ml [aventis]
28977	intal spincaps
1683	intal spincaps inhalation powder capsules [aventis]
2260	intal spinhaler insufflator [aventis]
10361	intal syncroner 5 mg inh
4647	intal syncroner 5mg/inhalation [aventis]
7580	intal whistle
35557	ipramol steri-neb unit dose nebulising solution 500micrograms + 2.5mg/2.5ml [ivax]
25020	ipratropium bromide (forte)
1409	ipratropium bromide aerosol inhaler 20micrograms/dose
4268	ipratropium bromide aerosol inhaler 40micrograms/metered inhalation
6081	ipratropium bromide breath actuated inhaler 20micrograms/dose
8333	ipratropium bromide capsules (for inhalation) 40mcg
11779	ipratropium bromide capsules for inhalation + inhaler 40mcg
6522	ipratropium bromide cfc free inhaler 20micrograms/actuation
37791	ipratropium bromide inhalation solution 250micrograms/ml
23961	ipratropium bromide inhalation solution 250micrograms/ml [galen]
20803	ipratropium bromide nebuliser solution
1410	ipratropium bromide nebuliser solution 0.25mg/ml
6772	ipratropium bromide unit dose nebuliser solution 250micrograms/ml
30229	ipratropium bromide unit dose nebuliser solution 250micrograms/ml [galen]
6719	ipratropium bromide unit dose nebuliser solution 500micrograms/2ml
1411	ipratropium bromide unit dose nebulising solution 250micrograms/ml
40177	ipratropium bromide unit dose nebulising solution 250micrograms/ml [hillcross]
26616	ipratropium bromide with fenoterol hydrobromide aerosol inhaler 40micrograms + 100micrograms/actuation
27505	ipratropium bromide with fenoterol hydrobromide breath actuated inhaler 40micrograms + 100micrograms/actuation
9270	ipratropium bromide with fenoterol hydrobromide unit dose nebulising solution 500micrograms + 1.25mg/4ml
2152	ipratropium bromide with salbutamol aerosol inhaler 20mcg + 100mcg
11046	ipratropium bromide with salbutamol unit dose nebulising solution 500micrograms + 2.5mg/2.5ml
33461	iso-autohaler aerosol inhaler [3m]
42921	isoprenaline sulphate aerosol inhaler 400micrograms/actuation
10490	isoprenaline sulphate aerosol inhaler 80micrograms/actuation
21769	lasma tablets 300mg [pharmax]
7935	maxivent aerosol inhaler 100micrograms/inhalation [ashbourne]

23269	maxivent steripoule unit dose nebulising solution 2.5mg/2.5ml [ashbourne]
25339	maxivent steripoule unit dose nebulising solution 5mg/2.5ml [ashbourne]
8872	medihaler -iso aerosol [3m]
2259	medihaler -iso forte aerosol inhaler [3m]
28241	min-i-jet aminophylline injection 250mg/10ml [ucb]
16018	mometasone furoate dry powder inhaler 200micrograms/actuation
10254	mometasone furoate dry powder inhaler 400micrograms/actuation
17874	monovent syrup 1.5mg/5ml [lagap]
41832	monovent syrup 1.5mg/5ml [sandoz]
622	montelukast (as sodium salt) chewable tablet 4mg
5957	montelukast (as sodium salt) chewable tablet 5mg
7088	montelukast (as sodium salt) granules 4mg/sachet
808	montelukast (as sodium salt) tablets 10mg
8608	nedocromil sodium aerosol inhaler 2mg/inhalation
13256	nedocromil sodium cfc free inhaler 2mg/inhalation
33434	noradran syrup 7.5mg + 15mg/5ml [norma]
14739	norphyllin sr tablets 225mg [ivax]
23741	novolizer budesonide breath actuated dry powder inhaler 200micrograms/actuation [meda]
10331	nuelin liquid 60mg/5ml [3m]
2995	nuelin sa tablets 175mg [meda]
5261	nuelin sa-250 tablets [meda]
7841	nuelin tablets 125mg [3m]
32812	numotac tablets 10mg [3m]
461	orciprenaline aerosol inhaler 750micrograms/inhalation
8151	orciprenaline aerosol refill 750micrograms/inhalation
32283	orciprenaline injection 0.5mg/ml
2490	orciprenaline sugar free oral solution 10mg/5ml
7943	orciprenaline tablets 20mg
31262	orciprenaline with bromhexine hydrochloride mixture
1974	oxis 12 turbohaler dry powder inhaler 12micrograms/actuation [astrazenec]
1975	oxis 6 turbohaler dry powder inhaler 6 micrograms/actuation [astrazenec]
2437	oxitropium bromide aerosol inhaler 100micrograms/actuation
9658	oxitropium bromide breath actuated inhaler 100micrograms/actuation
3039	oxivent aerosol inhaler 100micrograms/actuation [boeh ingl]
3850	oxivent autohaler breath actuated inhaler 100micrograms/actuation [boeh ingl]
12699	pecram sustained release tablets 225mg [novartis]
27040	phyllocontin continus
8806	phyllocontin continus forte tablets 350mg [napppharm]
590	phyllocontin continus tablets 225mg [napppharm]
39040	phyllocontin forte continus tablets 350mg [napppharm]
180	phyllocontin sup
12463	pirbuterol 15 mg tab
16236	pirbuterol acetate aerosol inhaler

22661	pirbuterol capsules 10mg
8252	pirbuterol capsules 15mg
25829	pirbuterol syrup 7.5mg/5ml
11993	pro-vent capsules 300mg [wellcome]
3758	pulmadil aerosol inhaler [3m]
10858	pulmadil auto aerosol inhaler [3m]
27583	pulmicort
454	pulmicort aerosol inhaler 200micrograms [astrazenec]
39099	pulmicort cfc free inhaler 100micrograms [astrazenec]
40057	pulmicort cfc free inhaler 200micrograms [astrazenec]
26665	pulmicort complete
3442	pulmicort complete 200 mcg inh
3188	pulmicort complete 50 mcg inh
23675	pulmicort l.s. refil
1680	pulmicort ls aerosol inhaler 50micrograms [astrazenec]
4545	pulmicort ls refill canister 50micrograms [astrazenec]
2124	pulmicort refil 200 mcg inh
8251	pulmicort refil 50 mg inh
20812	pulmicort refill
2125	pulmicort refill canister 200micrograms [astrazenec]
1959	pulmicort respules nebuliser suspension 0.5mg/2ml [astrazenec]
1956	pulmicort respules nebuliser suspension 1mg/2ml [astrazenec]
960	pulmicort turbohaler dry powder inhaler 100micrograms/actuation [astrazenec]
956	pulmicort turbohaler dry powder inhaler 200micrograms/actuation [astrazenec]
908	pulmicort turbohaler dry powder inhaler 400micrograms/actuation [astrazenec]
14757	pulvinal beclometasone dipropionate dry powder inhaler 100micrograms/actuation [chiesi]
13037	pulvinal beclometasone dipropionate dry powder inhaler 200micrograms/actuation [chiesi]
14736	pulvinal beclometasone dipropionate dry powder inhaler 400micrograms/actuation [chiesi]
13038	pulvinal salbutamol dry powder inhaler 200micrograms/actuation [chiesi]
4413	qvar autohaler cfc free breath actuated inhaler 100micrograms/actuation [ivax]
3220	qvar autohaler cfc free breath actuated inhaler 50micrograms/actuation [ivax]
2335	qvar cfc free inhaler 100micrograms/actuation [ivax]
3546	qvar cfc free inhaler 50micrograms/actuation [ivax]
18848	qvar easi-breathe cfc free breath actuated inhaler 100micrograms/actuation [ivax]
14294	qvar easi-breathe cfc free breath actuated inhaler 50micrograms/actuation [ivax]
15165	reproterol aerosol inhaler 500micrograms/dose
36677	reproterol elixir 10mg/5ml
22790	reproterol respirator solution 10mg/ml
22663	respacal tablets 2mg [ucb]
18421	respontin nebules unit dose nebulising solution 250micrograms/ml [glaxo]
23567	respontin nebules unit dose nebulising solution 250micrograms/ml [glaxo]
18140	respontin nebules unit dose nebulising solution 500micrograms/2ml [glaxo]
8572	rimiterol aerosol inhaler

18937	sabidal sr 270 270 mg tab
1093	salamol aerosol inhaler 100micrograms/actuation [ivax]
5170	salamol cfc free inhaler 100micrograms/inhalation [ivax]
5889	salamol cfc free inhaler 100micrograms/inhalation [kent]
13996	salamol cfc free inhaler 100micrograms/inhalation [sandoz]
957	salamol easi-breathe breath actuated inhaler 100micrograms/actuation [ivax]
5516	salamol easi-breathe cfc free breath actuated inhaler 100micrograms/actuation [ivax]
5898	salamol steri-neb unit dose nebulising solution 2.5mg/2.5ml [numark]
5837	salamol steri-neb unit dose nebulising solution 5mg/2.5ml [numark]
31845	salapin syrup 2mg/5ml [pinewood]
18622	Salbulin 2mg Tablet (3M Health Care Ltd)
862	salbulin aerosol inhaler [3m]
31290	salbulin cfc free
4665	salbulin cfc free inhaler 100micrograms/actuation [3m]
38226	salbulin mdpi novolizer dry powder inhalation cartridge (refill) 100micrograms [meda]
38136	salbulin mdpi novolizer dry powder inhalation cartridge with device 100micrograms [meda]
4055	salbulin syrup 2mg/5ml [3m]
3254	salbulin tablets 4mg [3m]
25073	salbutamol
10958	salbutamol .25 mg inj
2395	salbutamol 2 mg/5ml syr
3838	salbutamol 400mcg/beclometh.100mcg r/cap inh
8	salbutamol aerosol inhaler 100micrograms/inhalation
34311	salbutamol aerosol inhaler 100micrograms/inhalation [berk]
44713	salbutamol aerosol inhaler 100micrograms/inhalation [celltech]
34702	salbutamol aerosol inhaler 100micrograms/inhalation [cp pharm]
33588	salbutamol aerosol inhaler 100micrograms/inhalation [gen (uk)]
31933	salbutamol aerosol inhaler 100micrograms/inhalation [hillcross]
28508	salbutamol aerosol inhaler 100micrograms/inhalation [ivax]
33089	salbutamol aerosol inhaler 100micrograms/inhalation [kent]
1698	salbutamol breath actuated inhaler 100micrograms/actuation
30230	salbutamol breath actuated inhaler 100micrograms/actuation
882	salbutamol capsules (for inhalation) 200micrograms
30204	salbutamol capsules (for inhalation) 200micrograms
2850	salbutamol capsules (for inhalation) 400micrograms
34029	salbutamol capsules (for inhalation) 400micrograms
1741	salbutamol cfc free breath actuated inhaler 100micrograms/actuation
17	salbutamol cfc free inhaler 100micrograms/inhalation
33817	salbutamol cfc free inhaler 100micrograms/inhalation [actavis]
30118	salbutamol cfc free inhaler 100micrograms/inhalation [aps]
34310	salbutamol cfc free inhaler 100micrograms/inhalation [hillcross]
34619	salbutamol cfc free inhaler 100micrograms/inhalation [kent]
46551	salbutamol cfc free inhaler 100micrograms/inhalation [neolab]

25218	salbutamol cfc/free b/a
18968	salbutamol concentrate for solution for infusion 5mg/5ml
38097	salbutamol cyclocaps 200micrograms [du pont]
38416	salbutamol cyclocaps 400micrograms [du pont]
33373	salbutamol cyclocaps capsules (for inhalation) 200micrograms [teva]
32050	salbutamol cyclocaps capsules (for inhalation) 400micrograms [teva]
30212	salbutamol cyclohaler
27793	salbutamol cyclohaler type 5 insufflator [bms]
3163	salbutamol disc 200micrograms
5753	salbutamol disc 400micrograms
38214	salbutamol dry powder inhalation cartridge (refill) 100micrograms
38079	salbutamol dry powder inhalation cartridge with device 100micrograms
7017	salbutamol dry powder inhaler 100micrograms/actuation
2978	salbutamol dry powder inhaler 200micrograms/actuation
6462	salbutamol dry powder inhaler 95micrograms
9805	salbutamol infusion 100micrograms/ml
22512	salbutamol inhaler
1346	salbutamol injection 0.05mg/ml
15613	salbutamol injection 500micrograms/1ml
9384	salbutamol modified release capsules 4mg
696	salbutamol modified release capsules 8mg
3994	salbutamol modified release tablet 4mg
2869	salbutamol modified release tablet 8mg
22467	salbutamol respirator soln
7965	salbutamol respirator solution 5mg/ml
20675	salbutamol rotahaler complete unit
3443	salbutamol spacehaler 100micrograms/inhalation [celltech]
28881	salbutamol sugar free oral solution [hillcross]
282	salbutamol sugar free oral solution 2mg/5ml
41691	salbutamol sugar free oral solution 2mg/5ml [sandoz]
21102	salbutamol sugar free syrup 2mg/5ml [lagap]
881	salbutamol tablets 2mg
34618	salbutamol tablets 2mg [actavis]
41548	salbutamol tablets 2mg [aps]
41549	salbutamol tablets 2mg [cp pharm]
860	salbutamol tablets 4mg
34938	salbutamol tablets 4mg [actavis]
32102	salbutamol tablets 4mg [hillcross]
42497	salbutamol tablets 8mg
20781	salbutamol u.dose nebulising 2.5mg/2.5ml
1630	salbutamol unit dose nebulising solution 2.5mg/2.5ml
34162	salbutamol unit dose nebulising solution 2.5mg/2.5ml [galen]
40709	salbutamol unit dose nebulising solution 2.5mg/2.5ml [hillcross]

1711	salbutamol unit dose nebulising solution 5mg/2.5ml
34018	salbutamol unit dose nebulising solution 5mg/2.5ml [galen]
45863	salbutamol unit dose nebulising solution 5mg/2.5ml [gen (uk)]
14525	salbutamol vortex metered dose inhaler 100micrograms/inhalation
11307	salbutamol with beclometasone aerosol inhaler 100mcg + 50mcg
18456	salbutamol with beclometasone capsules (for inhalation) 200micrograms + 100micrograms
14561	salbutamol with beclometasone capsules (for inhalation) 400micrograms + 200micrograms
12909	salbutamol with ipratropium bromide aerosol inhaler 100micrograms + 20micrograms/actuation
12822	salbutamol with ipratropium bromide unit dose nebulising solution 2.5mg + 500micrograms/2.5ml
40655	salbuvent aerosol inhaler 100micrograms/actuation [pharmacia]
3189	salbuvent inh inh
27340	salbuvent injection 0.5mg/ml [pharmacia]
31082	salbuvent respirator solution 5mg/ml [pharmacia]
10353	salbuvent rondo
1635	salbuvent syrup 2mg/5ml [pharmacia]
20838	salbuvent tablets 2mg [pharmacia]
29267	salbuvent tablets 4mg [pharmacia]
43046	salipraneb unit dose nebulising solution 500micrograms + 2.5mg/2.5ml [breath]
465	salmeterol aerosol inhaler 25micrograms/actuation
7270	salmeterol cfc free inhaler 25micrograms/actuation
3297	salmeterol disc 50micrograms
719	salmeterol dry powder inhaler 50micrograms/actuation
35503	salmeterol inhalation powder blisters (refill) 50micrograms
35542	salmeterol inhalation powder blisters with device 50micrograms
6569	salmeterol with fluticasone cfc free inhaler 25micrograms + 125micrograms/actuation
5864	salmeterol with fluticasone cfc free inhaler 25micrograms + 250micrograms/actuation
6616	salmeterol with fluticasone cfc free inhaler 25micrograms + 50micrograms/actuation
6938	salmeterol with fluticasone dry powder inhaler 50micrograms + 100micrograms/inhalation
5942	salmeterol with fluticasone dry powder inhaler 50micrograms + 250micrograms/inhalation
5558	salmeterol with fluticasone dry powder inhaler 50micrograms+ 500micrograms/inhalation
665	seretide 100 accuhaler dry powder inhaler [glaxo]
5161	seretide 125 evohaler cfc free inhaler [a & h]
638	seretide 250 accuhaler dry powder inhaler [glaxo]
5172	seretide 250 evohaler cfc free inhaler [a & h]
5143	seretide 50 evohaler cfc free inhaler [a & h]
3666	seretide 500 accuhaler dry powder inhaler [glaxo]
2224	serevent accuhaler 50micrograms/actuation [glaxo]
549	serevent aerosol inhaler 25micrograms/actuation [glaxo]
35825	serevent diskhaler (refill) inhalation powder 50micrograms [glaxo]
910	serevent diskhaler 50micrograms [glaxo]
35165	serevent diskhaler inhalation powder 50micrograms [glaxo]
7268	serevent evohaler cfc free inhaler 25micrograms/actuation [glaxo]
695	singulair tablets 10mg [m s d]

863	slo-phyllin capsules 125mg [lipha]
15284	slo-phyllin capsules 125mg [merck ser]
2757	slo-phyllin capsules 250mg [lipha]
6315	slo-phyllin capsules 250mg [merck ser]
1097	slo-phyllin capsules 60mg [lipha]
11719	slo-phyllin capsules 60mg [merck ser]
37615	sodium cromoglicate aerosol inhaler 1mg/inhalation
964	sodium cromoglicate aerosol inhaler 5mg/inhalation
2158	sodium cromoglicate breath actuated inhaler 5mg/inhalation
2911	sodium cromoglicate capsules (for inhalation) 20mg
38471	sodium cromoglicate cfc free inhaler 5mg
14603	sodium cromoglicate inhaler and spacer 5mg/actuation
15765	sodium cromoglicate inhaler and spacer 5mg/inhalation
8498	sodium cromoglicate nebuliser solution 10mg/ml
20180	sodium cromoglicate with isoprenaline capsules (for inhalation)
8267	sodium cromoglicate with salbutamol aerosol inhaler
24380	sodium cromoglicate with salbutamol inhaler and spacer
24898	spacehaler bdp spacehaler 100micrograms/actuation [celltech]
20825	spacehaler bdp spacehaler 250micrograms/actuation [celltech]
28761	spacehaler bdp spacehaler 50micrograms/actuation [celltech]
22430	spacehaler salbutamol spacehaler 100micrograms/inhalation [celltech]
6050	spiriva capsules (for inhalation) 18 micrograms [boeh ingl]
35000	spiriva inhalation powder capsules (refill) 18 micrograms [boeh ingl]
34995	spiriva inhalation powder capsules with device 18 micrograms [boeh ingl]
36869	spiriva respimat inhalation solution 2.5 micrograms/actuation [boeh ingl]
3585	steri-neb cromogen nebuliser solution 10mg/ml [ivax]
1415	steri-neb ipratropium unit dose nebulising solution 250micrograms/ml [ivax]
6758	steri-neb ipratropium unit dose nebulising solution 250micrograms/ml [ivax]
23709	steri-neb ipratropium unit dose nebulising solution 500micrograms/2ml [ivax]
2149	steri-neb salamol 2.5 mg inh
4634	steri-neb salamol unit dose nebulising solution 2.5mg/2.5ml [ivax]
1414	steri-neb salamol unit dose nebulising solution 5mg/2.5ml [ivax]
40637	steripoule ipratropium unit dose nebuliser solution 250micrograms/ml [galen]
40832	steripoule ipratropium unit dose nebuliser solution 500micrograms/2ml [galen]
42279	steripoule salbutamol unit dose nebuliser solution 2.5mg/2.5ml [galen]
40599	steripoule salbutamol unit dose nebuliser solution 5mg/2.5ml [galen]
7013	symbicort turbohaler dry powder inhaler 100micrograms + 6micrograms/actuation [astrazenec]
6325	symbicort turbohaler dry powder inhaler 200micrograms + 6micrograms/actuation [astrazenec]
6780	symbicort turbohaler dry powder inhaler 400micrograms + 12micrograms/actuation [astrazenec]
27944	tedral elixir [parke]
12274	tedral tablets [parke]
1620	terbutaline aerosol inhaler 250micrograms/actuation
1619	terbutaline dry powder inhaler 500micrograms

35861	terbutaline injection 2.5mg/5ml
35862	terbutaline injection 500micrograms/1ml
14483	terbutaline injection 500micrograms/ml
8522	terbutaline modified release tablet 7.5mg
1628	terbutaline refill canister 250micrograms/actuation
8676	terbutaline respirator solution 10mg/ml
3763	terbutaline respules inh
7711	terbutaline spacer inhaler 250micrograms/actuation
7953	terbutaline sugar free oral solution 1.5mg/5ml
38419	terbutaline syrup 1.5mg/5ml [hillcross]
42867	terbutaline syrup 1.5mg/5ml [sandoz]
10825	terbutaline tablets 5mg
5308	terbutaline unit dose nebuliser solution 5mg/2ml
37612	terbutaline unit dose nebulising solution 2.5mg/ml [galen]
17875	terbutaline with guaifenesin expectorant
7731	theo-dur tablets 200mg [astrazenec]
7730	theo-dur tablets 300mg [astrazenec]
1832	theograd tablets 350mg [abbott]
8955	theophylline 100 mg tab
32893	theophylline 100mg/lysine 74mg mg tab
273	theophylline 200 mg cap
15409	theophylline 3 mg sol
10432	theophylline 300 mg sup
10744	theophylline 80 mg eli
10433	theophylline liquid 60mg/5ml
879	theophylline modified release capsules 125mg
2147	theophylline modified release capsules 250mg
12240	theophylline modified release capsules 300mg
880	theophylline modified release capsules 60mg
3388	theophylline modified release tablet 175mg
1833	theophylline modified release tablet 200mg
7733	theophylline modified release tablet 250mg
7732	theophylline modified release tablet 300mg
9092	theophylline modified release tablet 350mg
1834	theophylline modified release tablet 400mg
38120	theophylline modified release tablet 500mg
15365	theophylline sugar free elixir 10mg/5ml
10723	theophylline syrup 125mg/5ml
4593	theophylline tablets 125mg
19953	theophylline with ephedrine and caffeine tablets
15153	theophylline with ephedrine hydrochloride tablets 120mg + 11mg
26860	theophylline with ephedrine sulphate tablets 120mg + 15mg
20817	tilade aerosol

8215	tilade aerosol inhaler 2mg/inhalation [sanofi/ave]
25119	tilade cfc free inhaler 2mg/inhalation [sanofi/ave]
3688	tilade mint inhaler 2mg/inhalation [sanofi/ave]
10597	tilade mint syncroner 2mg/inhalation [sanofi/ave]
746	tiotropium capsules (for inhalation) 18 micrograms
35011	tiotropium inhalation powder capsules (refill) 18 micrograms
35014	tiotropium inhalation powder capsules with device 18 micrograms
36864	tiotropium inhalation solution 2.5 micrograms/actuation
42103	tulobuterol sugar free syrup 1mg/5ml
19799	tulobuterol tablets 2mg
19735	uniphyllin continus
31758	uniphyllin continus
1423	uniphyllin continus prolonged release tablet 200mg [napppharm]
5941	uniphyllin continus prolonged release tablet 300mg [napppharm]
5453	uniphyllin continus prolonged release tablet 400mg [napppharm]
1801	ventide aerosol inhaler [a & h]
16625	ventide rotacaps [a & h]
17696	ventmax sr modified release capsules 4mg [opus]
22313	ventmax sr modified release capsules 8mg [opus]
1882	ventodisks disc 200micrograms/blister [a & h]
1950	ventodisks disc 400micrograms/blister [a & h]
26525	ventolin
27573	ventolin
7452	ventolin .25 mg inj
4497	ventolin accuhaler 200micrograms/actuation [glaxo]
42858	ventolin accuhaler dry powder inhaler 200micrograms/actuation [glaxo]
31	ventolin aerosol inhaler 100micrograms/inhalation [glaxo]
24645	ventolin concentrate for solution for infusion 5mg/5ml [a & h]
10458	ventolin cr tablets 4mg [a & h]
12042	ventolin cr tablets 8mg [a & h]
958	ventolin easi-breathe breath actuated inhaler 100micrograms/actuation [a & h]
898	ventolin evohaler 100micrograms/inhalation [glaxo]
42830	ventolin evohaler cfc free inhaler 100micrograms/inhalation [glaxo]
8429	ventolin i/v 5 mg inj
17185	ventolin injection 500micrograms/1ml [a & h]
28577	ventolin injection 50micrograms/ml [a & h]
19642	ventolin nebules
674	ventolin nebules unit dose nebulising solution 2.5mg [a & h]
1957	ventolin nebules unit dose nebulising solution 5mg [a & h]
19653	ventolin respirator
510	ventolin respirator solution 5mg/ml [a & h]
23688	ventolin rotacaps
2851	ventolin rotacaps 200micrograms [a & h]

1952	ventolin rotacaps 400micrograms [a & h]
19649	ventolin rotahaler
4908	ventolin rotahaler insufflator [a & h]
19726	ventolin s/r
8636	ventolin s/r 8 mg spa
856	ventolin syrup 2mg/5ml [a & h]
4171	ventolin tablets 2mg [a & h]
987	ventolin tablets 4mg [a & h]
1961	volmax tablets 4mg [a & h]
1960	volmax tablets 8mg [a & h]
17701	zafirlukast
7132	zafirlukast tablets 20mg
58269	AirSalb 100micrograms/dose inhaler CFC free (Sandoz Ltd)
50018	Aminophylline 250mg/10ml solution for injection ampoules
57228	Aminophylline 250mg/10ml solution for injection ampoules (A A H Pharmaceuticals Ltd)
58600	Aminophylline 250mg/10ml solution for injection ampoules (AMCo)
61346	Aminophylline 360mg suppositories (Special Order)
57249	Asmavent 100micrograms/dose inhaler CFC free (Kent Pharmaceuticals Ltd)
50810	Atrovent 20micrograms/dose inhaler CFC free (DE Pharmaceuticals)
57557	Atrovent 20micrograms/dose inhaler CFC free (Lexon (UK) Ltd)
60920	Atrovent 20micrograms/dose inhaler CFC free (Sigma Pharmaceuticals Plc)
55132	Atrovent 500micrograms/2ml nebuliser liquid UDVs (Waymade Healthcare Plc)
47943	Beclazone easi-breathe (roi) 100microgram/actuation Pressurised inhalation (Ivax Pharmaceuticals Ireland)
57589	Becloforte 250micrograms/dose inhaler (Dowelhurst Ltd)
62030	Beclometasone 100micrograms/dose / Formoterol 6micrograms/dose dry powder inhaler
62518	Beclometasone 100micrograms/dose inhaler CFC free (Ennogen Healthcare Ltd)
63585	Beclometasone 50micrograms/dose inhaler (Almus Pharmaceuticals Ltd)
56471	Becodisks 200microgram (Mawdsley-Brooks & Company Ltd)
56462	Becodisks 400microgram (Waymade Healthcare Plc)
62341	Becotide 50 inhaler (Dowelhurst Ltd)
50701	Becotide Rotahaler (GlaxoSmithKline UK Ltd)
32533	BRONTISOL INH
18537	Budesonide 200microgram inhalation powder capsules
61975	Budesonide 500micrograms/2ml nebuliser liquid unit dose vials (Almus Pharmaceuticals Ltd)
32461	CHOLINE THEOPHYLLINATE 90 MG TAB
48340	Clenil Modulite 100micrograms/dose inhaler (Mawdsley-Brooks & Company Ltd)
49412	Clenil Modulite 200micrograms/dose inhaler (Mawdsley-Brooks & Company Ltd)
49367	Clenil Modulite 50micrograms/dose inhaler (Mawdsley-Brooks & Company Ltd)
51903	Combivent nebuliser liquid 2.5ml UDVs (DE Pharmaceuticals)
49904	Combivent nebuliser liquid 2.5ml UDVs (Lexon (UK) Ltd)
25674	CROMOGEN STERI-NEB 10 MG/ML SOL
61782	DuoResp Spiromax 160micrograms/dose / 4.5micrograms/dose dry powder inhaler (Teva UK Ltd)

61666	DuoResp Spiromax 320micrograms/dose / 9micrograms/dose dry powder inhaler (Teva UK Ltd)
56477	Flixotide 100micrograms/dose Accuhaler (Waymade Healthcare Plc)
56474	Flixotide 125micrograms/dose Evohaler (DE Pharmaceuticals)
57555	Flixotide 125micrograms/dose Evohaler (Dowelhurst Ltd)
57525	Flixotide 250micrograms/dose Accuhaler (Stephar (U.K.) Ltd)
56484	Flixotide 250micrograms/dose Accuhaler (Waymade Healthcare Plc)
51815	Flixotide 250micrograms/dose Evohaler (Waymade Healthcare Plc)
16305	Flixotide 2mg/2ml Nebules (GlaxoSmithKline UK Ltd)
56499	Flixotide 500micrograms/dose Accuhaler (Waymade Healthcare Plc)
57579	Flixotide 50micrograms/dose Accuhaler (DE Pharmaceuticals)
56475	Flixotide 50micrograms/dose Accuhaler (Sigma Pharmaceuticals Plc)
53057	Flixotide 50micrograms/dose Evohaler (Lexon (UK) Ltd)
51209	Fluticasone 125micrograms/dose / Formoterol 5micrograms/dose inhaler CFC free
49868	Fluticasone 250micrograms/dose / Formoterol 10micrograms/dose inhaler CFC free
49772	Fluticasone 250micrograms/dose Evohaler (Sigma Pharmaceuticals Plc)
51270	Fluticasone 50micrograms/dose / Formoterol 5micrograms/dose inhaler CFC free
59899	Fluticasone furoate 184micrograms/dose / Vilanterol 22micrograms/dose dry powder inhaler
59439	Fluticasone furoate 92micrograms/dose / Vilanterol 22micrograms/dose dry powder inhaler
50036	Flutiform 125micrograms/dose / 5micrograms/dose inhaler (Napp Pharmaceuticals Ltd)
48666	Flutiform 250micrograms/dose / 10micrograms/dose inhaler (Napp Pharmaceuticals Ltd)
50689	Flutiform 50micrograms/dose / 5micrograms/dose inhaler (Napp Pharmaceuticals Ltd)
61644	Fostair NEXThaler 100micrograms/dose / 6micrograms/dose dry powder inhaler (Chiesi Ltd)
53174	Ipratropium bromide 500micrograms/2ml nebuliser liquid unit dose vials (A A H Pharmaceuticals Ltd)
32222	Isoprenaline hc 500micrograms + 50micrograms/metered Pressurised inhalation
28614	ISOPRENALINE SULPHATE COMPLETE UNIT INH
31570	ISOPRENALINE SULPHATE REFILL VIAL INH
54641	Isoprenaline sulphate with sodium cromoglicate inhalation Capsule
31354	ISOPRENALINE/PHENYLEPHRINE 400-DOSE COMP INH
41830	Medihaler -duo Inhalation powder (3M Health Care Ltd)
20824	MEDIHALER-DUO INH
23385	MEDIHALER-EPI
21875	MEDIHALER-ISO
20673	MEDIHALER-ISO FORTE
62490	Montelukast 10mg tablets (A A H Pharmaceuticals Ltd)
59819	Montelukast 10mg tablets (Actavis UK Ltd)
62410	Montelukast 10mg tablets (Alliance Healthcare (Distribution) Ltd)
60331	Montelukast 10mg tablets (Ranbaxy (UK) Ltd)
59263	Montelukast 10mg tablets (Teva UK Ltd)
56604	Montelukast 4mg chewable tablets sugar free (Actavis UK Ltd)
56756	Montelukast 4mg granules sachets sugar free (A A H Pharmaceuticals Ltd)
63457	Montelukast 5mg chewable tablets sugar free (Accord Healthcare Ltd)
59968	Montelukast 5mg chewable tablets sugar free (Teva UK Ltd)
59898	Nedocromil 2mg/dose inhaler

63644	Nedocromil 2mg/dose inhaler with spacer
47638	Neovent 25micrograms/dose inhaler CFC free (Kent Pharmaceuticals Ltd)
15413	ORCIPRENALINE SULPHATE 5 % SOL
56482	Oxis 12 Turbohaler (Waymade Healthcare Plc)
57558	Oxis 6 Turbohaler (Lexon (UK) Ltd)
52732	Pulmicort 0.5mg Respules (Necessity Supplies Ltd)
50037	Pulmicort 0.5mg Respules (Waymade Healthcare Plc)
60937	Pulmicort 200 Turbohaler (Dowelhurst Ltd)
56498	Pulmicort 200 Turbohaler (Waymade Healthcare Plc)
49711	Pulmicort 200micrograms/dose inhaler (AstraZeneca UK Ltd)
51480	Qvar 100 Autohaler (DE Pharmaceuticals)
52806	Qvar 100 Autohaler (Lexon (UK) Ltd)
54399	Qvar 100 Autohaler (Sigma Pharmaceuticals Plc)
53480	Qvar 100 Autohaler (Stephar (U.K.) Ltd)
50287	Qvar 100 inhaler (DE Pharmaceuticals)
51681	Qvar 100 inhaler (Sigma Pharmaceuticals Plc)
51234	Qvar 100 inhaler (Waymade Healthcare Plc)
50129	Qvar 100micrograms/dose Easi-Breathe inhaler (DE Pharmaceuticals)
48709	Qvar 100micrograms/dose Easi-Breathe inhaler (Sigma Pharmaceuticals Plc)
54207	Qvar 50 inhaler (DE Pharmaceuticals)
51415	Qvar 50 inhaler (Mawdsley-Brooks & Company Ltd)
56493	Qvar 50micrograms/dose Easi-Breathe inhaler (Sigma Pharmaceuticals Plc)
17695	QVAR AUTOHALER
17702	QVAR AUTOHALER
17726	QVAR CFC FREE
59573	Relvar Ellipta 184micrograms/dose / 22micrograms/dose dry powder inhaler (GlaxoSmithKline UK Ltd)
59327	Relvar Ellipta 92micrograms/dose / 22micrograms/dose dry powder inhaler (GlaxoSmithKline UK Ltd)
60923	Salamol 100micrograms/dose Easi-Breathe inhaler (DE Pharmaceuticals)
48547	Salamol 100micrograms/dose inhaler CFC free (Arrow Generics Ltd)
61591	Salbutamol 100micrograms/dose inhaler CFC free (Phoenix Healthcare Distribution Ltd)
49591	Salbutamol 100micrograms/dose inhaler CFC free (Sandoz Ltd)
59409	Salbutamol 100micrograms/dose inhaler CFC free (Waymade Healthcare Plc)
48410	Salbutamol 2.5mg/2.5ml / Ipratropium bromide 500micrograms/2.5ml nebuliser liquid ampoules
48607	Salbutamol 2.5mg/2.5ml / Ipratropium bromide 500micrograms/2.5ml nebuliser liquid unit dose vials
61330	Salbutamol 2.5mg/2.5ml nebuliser liquid unit dose vials (Alliance Healthcare (Distribution) Ltd)
49369	Salbutamol 200microgram inhalation powder blisters
50315	Salbutamol 200microgram inhalation powder blisters with device
52543	Salbutamol 400microgram inhalation powder blisters
52799	Salbutamol 400microgram inhalation powder blisters with device
60601	Salbutamol 5mg/2.5ml nebuliser liquid unit dose vials (Alliance Healthcare (Distribution) Ltd)
54742	Salmeterol 25micrograms/dose inhaler CFC free (A A H Pharmaceuticals Ltd)
53982	Seebri Breezhaler 44microgram inhalation powder capsules with device (Novartis Pharmaceuticals UK Ltd)
62126	Seretide 100 Accuhaler (DE Pharmaceuticals)

53283	Seretide 100 Accuhaler (Waymade Healthcare Plc)
51027	Seretide 125 Evohaler (DE Pharmaceuticals)
51151	Seretide 125 Evohaler (Lexon (UK) Ltd)
53230	Seretide 250 Accuhaler (DE Pharmaceuticals)
50560	Seretide 250 Accuhaler (Sigma Pharmaceuticals Plc)
61280	Seretide 250 Accuhaler (Waymade Healthcare Plc)
48739	Seretide 250 Evohaler (DE Pharmaceuticals)
63252	Seretide 250 Evohaler (Lexon (UK) Ltd)
51909	Seretide 250 Evohaler (Necessity Supplies Ltd)
50886	Seretide 250 Evohaler (Stephar (U.K.) Ltd)
49000	Seretide 250 Evohaler (Waymade Healthcare Plc)
51593	Seretide 500 Accuhaler (DE Pharmaceuticals)
55677	Seretide 500 Accuhaler (Lexon (UK) Ltd)
51861	Seretide 500 Accuhaler (Mawdsley-Brooks & Company Ltd)
51394	Seretide 500 Accuhaler (Waymade Healthcare Plc)
50051	Serevent 25micrograms/dose Evohaler (Waymade Healthcare Plc)
56478	Serevent 50micrograms/dose Accuhaler (DE Pharmaceuticals)
57544	Serevent 50micrograms/dose Accuhaler (Waymade Healthcare Plc)
48396	Singulair 10mg tablets (Necessity Supplies Ltd)
22892	SODIUM CROMOGLYCATE 5 MG INH
7579	SODIUM CROMOGLYCATE COMPLETE UNIT INH
7578	SODIUM CROMOGLYCATE INSUF CARTS 20 MG
7577	SODIUM CROMOGLYCATE SPINHALER UNIT
9860	Space chamber type 3 device Device (Pari Medical Ltd)
6525	Space chamber type 3 with child mask 0-1 years Device (Pari Medical Ltd)
6220	Space chamber type 3 with child mask 1-4 years Device (Pari Medical Ltd)
17583	Space chamber type 3 with child mask 4-7 years Device (Pari Medical Ltd)
51967	Spiriva 18microgram inhalation powder capsules (Mawdsley-Brooks & Company Ltd)
50292	Spiriva 18microgram inhalation powder capsules (Sigma Pharmaceuticals Plc)
50577	Spiriva 18microgram inhalation powder capsules with HandiHaler (DE Pharmaceuticals)
59638	Spiriva 18microgram inhalation powder capsules with HandiHaler (Sigma Pharmaceuticals Plc)
50103	Spiriva 18microgram inhalation powder capsules with HandiHaler (Waymade Healthcare Plc)
61582	Spiriva Respimat 2.5micrograms/dose solution for inhalation cartridge with device (Waymade Healthcare Plc)
50945	Symbicort 100/6 Turbohaler (Mawdsley-Brooks & Company Ltd)
49114	Symbicort 100/6 Turbohaler (Sigma Pharmaceuticals Plc)
51570	Symbicort 200/6 Turbohaler (DE Pharmaceuticals)
51759	Symbicort 200/6 Turbohaler (Mawdsley-Brooks & Company Ltd)
53491	Symbicort 200/6 Turbohaler (Sigma Pharmaceuticals Plc)
53237	Symbicort 400/12 Turbohaler (DE Pharmaceuticals)
50739	Symbicort 400/12 Turbohaler (Mawdsley-Brooks & Company Ltd)
7617	TEDRAL SA TAB
24023	Theodrox Tablet (3M Health Care Ltd)

48484	Theophylline 250mg/5ml oral suspension
51430	Theophylline 60mg/5ml oral suspension
25093	THEOPHYLLINE S/R
13781	Ventide Rotahaler (GlaxoSmithKline UK Ltd)
49370	Ventodisks 200microgram (GlaxoSmithKline UK Ltd)
49368	Ventodisks 200microgram with Diskhaler (GlaxoSmithKline UK Ltd)
48742	Ventodisks 400microgram (GlaxoSmithKline UK Ltd)
48809	Ventodisks 400microgram with Diskhaler (GlaxoSmithKline UK Ltd)
48490	Ventolin 100micrograms/dose Evohaler (DE Pharmaceuticals)
48741	Ventolin 100micrograms/dose Evohaler (Mawdsley-Brooks & Company Ltd)
48519	Ventolin 100micrograms/dose Evohaler (Waymade Healthcare Plc)
53019	Ventolin 2.5mg Nebules (Mawdsley-Brooks & Company Ltd)
50956	Ventolin 200micrograms/dose Accuhaler (DE Pharmaceuticals)
57524	Ventolin 200micrograms/dose Accuhaler (Dowelhurst Ltd)
50557	Ventolin 200micrograms/dose Accuhaler (Lexon (UK) Ltd)
50503	Ventolin 200micrograms/dose Accuhaler (Mawdsley-Brooks & Company Ltd)
53297	Ventolin 200micrograms/dose Accuhaler (Sigma Pharmaceuticals Plc)
57694	Vertine 25micrograms/dose inhaler CFC free (Teva UK Ltd)
15032	SODIUM CROMOGLYCATE 10 MG/ML SOL
17691	QVAR CFC FREE
24291	FRANOL PLUS
19360	ISO-AUTOHALER COMPLETE UNIT
33149	ISO-AUTOHALER REFIL
19358	DUO-AUTOHALER COMPLETE
59626	Duo-autohaler Inhalation powder (3M Health Care Ltd)
33135	DUO-AUTOHALER REFIL
10407	phyllocontin continus paediatric tablets 100mg [napppharm]
14162	singulair paediatric chewable tablet 4mg [m s d]
5594	singulair paediatric chewable tablet 5mg [m s d]
14200	singulair paediatric granules 4mg/sachet [m s d]
26079	uniphyllin paediatric continus
18484	ventide paediatric rotacaps [a & h]
24207	aminophylline paed 50 mg sup
57621	Singulair Paediatric 4mg granules sachets (Mawdsley-Brooks & Company Ltd)
8705	ephedrine hcl 24mg/theophylline 120mg mg tab
28786	ephedrine hcl 25mg/aminophylline 130mg mg cap
26724	ephedrine hcl/aminophylline e/c tab
18948	EPHEDRINE /GUAIPHENESIN /PHENOBARB/ THEO 4.75 MG ELI
24035	EPHEDRINE 15MG/THEOPHYLLINE 120MG 15 MG TAB
28394	ALUPENT .5 MG INJ
13319	Nethaprin dospan Tablet (Marion Merrell Dow Ltd)

Inhaled steroids

Prodcode	Product name
638	salmeterol xinafoate/fluticasone propionate
665	salmeterol xinafoate/fluticasone propionate
1801	beclometasone dipropionate/salbutamol
3556	beclometasone dipropionate/salbutamol
3666	salmeterol xinafoate/fluticasone propionate
5143	salmeterol xinafoate/fluticasone propionate
5161	salmeterol xinafoate/fluticasone propionate
5172	salmeterol xinafoate/fluticasone propionate
6325	budesonide/formoterol fumarate dihydrate
6780	budesonide/formoterol fumarate dihydrate
7013	budesonide/formoterol fumarate dihydrate
11307	beclometasone dipropionate/salbutamol
14561	beclometasone dipropionate/salbutamol
16625	beclometasone dipropionate/salbutamol
18456	beclometasone dipropionate/salbutamol
18484	beclometasone dipropionate/salbutamol
19121	beclometasone dipropionate/salbutamol
19376	beclometasone dipropionate/salbutamol
27915	fluticasone
3437	Becotide rotahaler type 4 insufflator Inhalation powder (Allen and Hanburys Ltd)
6839	Alvesco 160 inhaler (Takeda UK Ltd)
7356	Ciclesonide 80micrograms/dose inhaler CFC free
9356	Becotide rotahaler insufflator Inhalation powder (Allen and Hanburys Ltd)
10102	Ciclesonide 160micrograms/dose inhaler CFC free
10254	Mometasone 400micrograms/dose dry powder inhaler
16018	Mometasone 200micrograms/dose dry powder inhaler
16433	Asmanex 200micrograms/dose Twisthaler (Merck Sharp & Dohme Ltd)
17590	Asmanex 400micrograms/dose Twisthaler (Merck Sharp & Dohme Ltd)
21224	Alvesco 80 inhaler (Takeda UK Ltd)
22225	BECLOMETHASONE /SALBUTAMOL
46157	Beclometasone 200 Cyclocaps (Teva UK Ltd)
47943	Beclazone easi-breathe (roi) 100microgram/actuation Pressurised inhalation (Ivax Pharmaceuticals Ireland)
48340	Clenil Modulite 100micrograms/dose inhaler (Mawdsley-Brooks & Company Ltd)
48709	Qvar 100micrograms/dose Easi-Breathe inhaler (Sigma Pharmaceuticals Plc)
49367	Clenil Modulite 50micrograms/dose inhaler (Mawdsley-Brooks & Company Ltd)
49412	Clenil Modulite 200micrograms/dose inhaler (Mawdsley-Brooks & Company Ltd)
49711	Pulmicort 200micrograms/dose inhaler (AstraZeneca UK Ltd)
49772	Fluticasone 250micrograms/dose Evohaler (Sigma Pharmaceuticals Plc)
49868	Fluticasone 250micrograms/dose / Formoterol 10micrograms/dose inhaler CFC free

50037	Pulmicort 0.5mg Respules (Waymade Healthcare Plc)
50129	Qvar 100micrograms/dose Easi-Breathe inhaler (DE Pharmaceuticals)
50287	Qvar 100 inhaler (DE Pharmaceuticals)
50701	Becotide Rotahaler (GlaxoSmithKline UK Ltd)
51209	Fluticasone 125micrograms/dose / Formoterol 5micrograms/dose inhaler CFC free
51234	Qvar 100 inhaler (Waymade Healthcare Plc)
51270	Fluticasone 50micrograms/dose / Formoterol 5micrograms/dose inhaler CFC free
51415	Qvar 50 inhaler (Mawdsley-Brooks & Company Ltd)
51480	Qvar 100 Autohaler (DE Pharmaceuticals)
51681	Qvar 100 inhaler (Sigma Pharmaceuticals Plc)
51815	Flixotide 250micrograms/dose Evohaler (Waymade Healthcare Plc)
52732	Pulmicort 0.5mg Respules (Necessity Supplies Ltd)
52806	Qvar 100 Autohaler (Lexon (UK) Ltd)
53057	Flixotide 50micrograms/dose Evohaler (Lexon (UK) Ltd)
53480	Qvar 100 Autohaler (Stephar (U.K.) Ltd)
54207	Qvar 50 inhaler (DE Pharmaceuticals)
54399	Qvar 100 Autohaler (Sigma Pharmaceuticals Plc)
56462	Becodisks 400microgram (Waymade Healthcare Plc)
56471	Becodisks 200microgram (Mawdsley-Brooks & Company Ltd)
56474	Flixotide 125micrograms/dose Evohaler (DE Pharmaceuticals)
56475	Flixotide 50micrograms/dose Accuhaler (Sigma Pharmaceuticals Plc)
56477	Flixotide 100micrograms/dose Accuhaler (Waymade Healthcare Plc)
56484	Flixotide 250micrograms/dose Accuhaler (Waymade Healthcare Plc)
56493	Qvar 50micrograms/dose Easi-Breathe inhaler (Sigma Pharmaceuticals Plc)
56498	Pulmicort 200 Turbohaler (Waymade Healthcare Plc)
56499	Flixotide 500micrograms/dose Accuhaler (Waymade Healthcare Plc)
57525	Flixotide 250micrograms/dose Accuhaler (Stephar (U.K.) Ltd)
57555	Flixotide 125micrograms/dose Evohaler (Dowelhurst Ltd)
57579	Flixotide 50micrograms/dose Accuhaler (DE Pharmaceuticals)
57589	Becloforte 250micrograms/dose inhaler (Dowelhurst Ltd)
59439	Fluticasone furoate 92micrograms/dose / Vilanterol 22micrograms/dose dry powder inhaler
59899	Fluticasone furoate 184micrograms/dose / Vilanterol 22micrograms/dose dry powder inhaler
60937	Pulmicort 200 Turbohaler (Dowelhurst Ltd)
61644	Fostair NEXThaler 100micrograms/dose / 6micrograms/dose dry powder inhaler (Chiesi Ltd)
61664	Clenil Modulite 250micrograms/dose inhaler (Waymade Healthcare Plc)
61975	Budesonide 500micrograms/2ml nebuliser liquid unit dose vials (Almus Pharmaceuticals Ltd)
62030	Beclometasone 100micrograms/dose / Formoterol 6micrograms/dose dry powder inhaler
62341	Becotide 50 inhaler (Dowelhurst Ltd)
62518	Beclometasone 100micrograms/dose inhaler CFC free (Ennogen Healthcare Ltd)
63585	Beclometasone 50micrograms/dose inhaler (Almus Pharmaceuticals Ltd)
38	beclometasone dipropionate
99	beclometasone dipropionate
454	budesonide

883	beclometasone dipropionate
895	beclometasone dipropionate
896	beclometasone dipropionate
908	budesonide
909	budesonide
911	fluticasone propionate
947	budesonide
956	budesonide
959	budesonide
960	budesonide
1100	beclometasone dipropionate
1236	beclometasone dipropionate
1242	beclometasone dipropionate
1243	beclometasone dipropionate
1258	beclometasone dipropionate
1259	beclometasone dipropionate
1269	beclometasone dipropionate
1406	beclometasone dipropionate
1412	fluticasone propionate
1424	fluticasone propionate
1426	fluticasone propionate
1518	fluticasone propionate
1537	beclometasone dipropionate
1551	beclometasone dipropionate
1552	beclometasone dipropionate
1642	budesonide
1676	fluticasone propionate
1680	budesonide
1725	beclometasone dipropionate
1727	beclometasone dipropionate
1734	beclometasone dipropionate
1861	beclometasone dipropionate
1885	beclometasone dipropionate
1951	beclometasone dipropionate
1956	budesonide
1959	budesonide
2092	budesonide
2124	budesonide
2125	budesonide
2148	beclometasone dipropionate
2159	beclometasone dipropionate
2160	beclometasone dipropionate
2229	beclometasone dipropionate

2282	fluticasone propionate
2335	beclometasone dipropionate
2440	fluticasone propionate
2600	beclometasone dipropionate
2723	fluticasone propionate
2892	beclometasone dipropionate
2893	beclometasone dipropionate
2951	fluticasone propionate
2992	beclometasone dipropionate
3018	beclometasone dipropionate
3065	betamethasone valerate
3075	beclometasone dipropionate
3119	beclometasone dipropionate
3150	beclometasone dipropionate
3188	budesonide
3220	beclometasone dipropionate
3289	fluticasone propionate
3363	beclometasone dipropionate
3442	budesonide
3546	beclometasone dipropionate
3570	budesonide
3743	beclometasone dipropionate
3753	fluticasone
3838	beclomethasome
3927	beclometasone dipropionate
3947	beclometasone dipropionate
3988	fluticasone
3989	fluticasone propionate
3993	beclometasone dipropionate
4131	fluticasone propionate
4132	fluticasone propionate
4365	beclometasone dipropionate
4413	beclometasone dipropionate
4499	beclometasone dipropionate
4545	budesonide
4601	beclometasone dipropionate
4688	fluticasone propionate
4759	beclometasone dipropionate
4801	budesonide
4803	beclometasone dipropionate
4926	fluticasone propionate
4942	budesonide
5223	fluticasone propionate

5309	fluticasone propionate
5521	beclometasone dipropionate
5522	beclometasone dipropionate
5551	fluticasone propionate
5558	salmeterol xinafoate/fluticasone propionate
5580	fluticasone propionate
5683	fluticasone propionate
5718	fluticasone propionate
5804	beclometasone dipropionate
5822	fluticasone propionate
5864	salmeterol xinafoate/fluticasone propionate
5885	fluticasone propionate
5942	salmeterol xinafoate/fluticasone propionate
5975	fluticasone propionate
5992	beclometasone dipropionate
6569	salmeterol xinafoate/fluticasone propionate
6616	salmeterol xinafoate/fluticasone propionate
6746	budesonide/formoterol fumarate dihydrate
6796	budesonide/formoterol fumarate dihydrate
6938	salmeterol xinafoate/fluticasone propionate
7602	fluticasone propionate
7638	fluticasone propionate
7653	beclometasone dipropionate
7724	betamethasone valerate
7788	budesonide
7891	fluticasone propionate
7948	fluticasone propionate
7964	beclometasone dipropionate
8111	beclometasone dipropionate
8251	budesonide
8433	budesonide
8450	fluticasone
8635	fluticasone propionate
9164	fluticasone propionate
9233	beclometasone dipropionate
9477	beclometasone dipropionate
9571	beclometasone dipropionate
9577	beclometasone dipropionate
9599	beclometasone dipropionate
9921	beclometasone dipropionate
10090	beclometasone dipropionate
10218	budesonide/formoterol fumarate dihydrate
10321	budesonide

11198	beclometasone dipropionate
11410	salmeterol xinafoate/fluticasone propionate
11478	fluticasone propionate
11497	beclometasone dipropionate
11588	salmeterol xinafoate/fluticasone propionate
11618	salmeterol xinafoate/fluticasone propionate
11732	beclometasone dipropionate
12994	salmeterol xinafoate/fluticasone propionate
13037	beclometasone dipropionate
13040	salmeterol xinafoate/fluticasone propionate
13273	salmeterol xinafoate/fluticasone propionate
13290	beclometasone dipropionate
13815	beclometasone dipropionate
14294	beclometasone dipropionate
14321	beclometasone dipropionate
14524	beclometasone dipropionate
14567	beclometasone dipropionate
14590	beclometasone dipropionate
14700	budesonide
14736	beclometasone dipropionate
14757	beclometasone dipropionate
15326	beclometasone dipropionate
15706	beclometasone dipropionate
16054	budesonide
16148	beclometasone dipropionate
16151	beclometasone dipropionate
16158	beclometasone dipropionate
16305	fluticasone propionate
16584	beclometasone dipropionate
17465	fluticasone propionate
17654	beclometasone dipropionate
17670	budesonide
18394	beclometasone dipropionate
18537	budesonide
18848	beclometasone dipropionate
19031	beclometasone dipropionate
19389	beclometasone dipropionate
19401	beclometasone dipropionate
19563	beclometasone
19736	beclometasone
20707	beclometasone
20763	beclometasone
20812	budesonide

20825	beclometasone dipropionate
21005	beclometasone dipropionate
21482	beclometasone dipropionate
23675	budesonide
23741	budesonide
24219	beclometasone
24898	beclometasone dipropionate
25204	beclometasone dipropionate
26063	beclometasone dipropionate
26665	budesonide
27188	budesonide
27525	beclometasone
27583	budesonide
27679	beclometasone dipropionate
28073	beclometasone dipropionate
28640	beclometasone dipropionate
28761	beclometasone dipropionate
29325	beclometasone dipropionate
30210	beclometasone dipropionate
30238	beclometasone dipropionate
30649	budesonide
31774	beclometasone dipropionate
32874	beclometasone dipropionate
33258	beclometasone dipropionate
33849	beclometasone dipropionate
34315	beclometasone dipropionate
34428	beclometasone dipropionate
34739	beclometasone dipropionate
34794	beclometasone dipropionate
34859	beclometasone dipropionate
34919	beclometasone dipropionate
35071	beclometasone dipropionate
35106	beclometasone dipropionate
35107	beclometasone dipropionate
35113	beclometasone dipropionate
35118	beclometasone dipropionate
35225	fluticasone propionate
35288	beclometasone dipropionate
35293	beclometasone dipropionate
35299	beclometasone dipropionate
35374	fluticasone propionate
35392	fluticasone propionate
35408	beclometasone dipropionate

35430	beclometasone dipropionate
35461	fluticasone propionate
35510	budesonide
35580	beclometasone dipropionate
35602	budesonide
35611	fluticasone propionate
35631	budesonide
35638	fluticasone propionate
35652	beclometasone dipropionate
35700	fluticasone propionate
35724	budesonide
35772	fluticasone propionate
35905	fluticasone propionate
35986	fluticasone propionate
36021	fluticasone propionate
36090	fluticasone propionate
36290	fluticasone propionate
36401	fluticasone propionate
36462	fluticasone propionate
37432	beclometasone dipropionate/formoterol fumarate dihydrate
37447	fluticasone propionate
37470	beclometasone dipropionate/formoterol fumarate dihydrate
39099	budesonide
39102	budesonide
39200	beclometasone dipropionate
39879	budesonide
40057	budesonide
41269	beclometasone dipropionate
41412	beclometasone dipropionate
42928	fluticasone propionate
42985	fluticasone propionate
42994	fluticasone propionate
43074	fluticasone propionate

Chronic kidney disease

Medcode	Readterm
512	Chronic renal failure
6712	End stage renal failure
8330	End-stage renal disease
12479	Chronic kidney disease stage 4
12566	Chronic kidney disease stage 3
12585	Chronic kidney disease stage 5
12720	Chronic renal impairment
53852	End stage renal failure
89332	predicted stage chronic kidney disease
94793	Chronic kidney disease stage 3 with proteinuria
94965	Chronic kidney disease stage 3A
95121	Chronic kidney disease stage 2 without proteinuria
95122	Chronic kidney disease stage 4 with proteinuria
95123	Chronic kidney disease stage 3 without proteinuria
95145	CKD stage 3 with proteinuria
95175	Chronic kidney disease stage 3A without proteinuria
95176	CKD stage 3A without proteinuria
95177	Chronic kidney disease stage 3B without proteinuria
95178	Chronic kidney disease stage 3B with proteinuria
95179	Chronic kidney disease stage 3B
95180	CKD stage 3B with proteinuria
95188	CKD stage 3 without proteinuria
95405	Chronic kidney disease stage 5 without proteinuria
95406	Chronic kidney disease stage 4 without proteinuria
95408	Chronic kidney disease stage 3A with proteinuria
95508	Chronic kidney disease stage 5 with proteinuria
95571	CKD stage 3A with proteinuria
97587	CKD stage 4 without proteinuria
97683	CKD stage 5 without proteinuria
99160	CKD stage 5 with proteinuria
99312	CKD stage 4 with proteinuria
100633	CKD stage 3B without proteinuria
105151	chronic kidney disease stage 5
2994	Peritoneal dialysis
2995	Acquired arteriovenous fistula
2996	Haemodialysis NEC
3205	Creation of arteriovenous fistula NEC
8037	Insertion of ambulatory peritoneal dialysis catheter
9765	Ligation of acquired arteriovenous fistula
11773	Dialysis for renal failure

18779	Repair of acquired arteriovenous fistula
20073	Renal dialysis
20196	H/O: renal dialysis
22252	[V]Renal dialysis status
23773	Removal of ambulatory peritoneal dialysis catheter
24151	Arteriovenous shunt
25521	Creation of brachial-cephalic fistula
28158	Kidney dialysis with complication without blame
28269	Creation of radial-cephalic fistula
30709	Insertion of temporary peritoneal dialysis catheter
30756	Continuous ambulatory peritoneal dialysis
31478	Removal of infected arteriovenous shunt
31549	Compensation for renal failure
35921	Failure of sterile precautions during perfusion
36442	Placement ambulatory dialysis apparatus - compens renal fail
44422	H/O: kidney dialysis
45160	[V]Aftercare involving peritoneal dialysis
46145	[V]Aftercare involving renal dialysis NOS
46438	[X] Peritoneal dialysis associated peritonitis
48022	Other specified compensation for renal failure
48639	Mechanical complication of dialysis catheter
48713	mechanical complication of arterio-venous surgical fistula
49028	h/o: kidney recipient
52088	[V]Preparatory care for dialysis
54844	[X]Failure sterile precautions dur kidney dialys/other perf
56760	Placement ambulatory apparatus compensation renal failure
58618	Arteriovenous shunt NOS
59194	Placement ambulatory apparatus- compensate renal failure OS
59315	Stenosis of arteriovenous dialysis fistula
60302	Creation of graft fistula for dialysis
60446	Care of haemodialysis equipment
60498	Reversing haemodialysis lines
60743	[V]Aftercare involving intermittent dialysis
63038	[V]Unspecified aftercare involving intermittent dialysis
63063	Insertion of arteriovenous prosthesis
63190	Attention to arteriovenous shunt
63305	Thrombectomy of arteriovenous fistula
63488	[V]Other specified aftercare involving intermittent dialysis
63502	Peritoneal dialysis bag procedure
64636	Compensation for renal failure NOS
64828	Peritoneal dialysis NEC
65089	Placement other apparatus- compensate for renal failure NOS
65398	Other specified arteriovenous shunt

66714	Renal dialysis with complication without blame
69266	Failure of sterile precautions during kidney dialysis
69427	Accid cut puncture perf h'ge - perfusion NOS
69760	[X]Other dialysis
71124	Haemofiltration
72336	Priming haemodialysis lines
74905	Haemodialysis training
83513	Placement other apparatus for compensation for renal failure
86419	dialysis fluid glucose level
88597	Automated peritoneal dialysis
96131	Banding of arteriovenous fistula
96184	Accid cut puncture perf h'ge - kidney dialysis
96347	Ligation of arteriovenous dialysis fistula
98888	peritoneal dialysis sample
99692	[v]aftercare involving extracorporeal dialysis
100235	Ligation of acquired arteriovenous fistula
101124	continuous ambulatory peritoneal dialysis associated perit
101736	dialysis fluid urea level
101756	Thomas intravascular shunt for dialysis
101912	dialysis training
104586	flushing of peritoneal dialysis catheter
104719	peritoneal dialysis training
105436	anaphylactoid reaction due to haemodialysis
105742	aneurysm of anastomotic site of dialysis av fistula
105760	ruptured aneurysm of dialysis vascular access
106720	thrombosis of dialysis arteriovenous fistula
106975	haemorrhage of dialysis arteriovenous fistula
107082	occlusion of dialysis arteriovenous fistula
107188	aneurysm of dialysis arteriovenous fistula
107217	arteriovenous fistula thrombosis
107220	aneurysm of needle site of dialysis arteriovenous fistula
107260	infection of dialysis arteriovenous fistula
107719	ligation of arteriovenous dialysis graft
107746	stenosis of dialysis vascular access
107900	disorders associated with peritoneal dialysis
108116	occlusion of dialysis vascular access
108213	infection of dialysis arteriovenous graft
108423	rupture of dialysis arteriovenous graft
16929	Anaemia secondary to renal failure
25394	Anaemia secondary to chronic renal failure
28684	Hypertensive heart and renal disease with renal failure
32423	Hypertensive renal disease with renal failure
53940	[X]Other chronic renal failure

57987	Hyperten heart&renal dis+both(congestv)heart and renal fail
107901	placement other apparatus- compensate for renal failure os
70712	det.ren.func.after ren.transpl
72004	Excision of rejected transplanted kidney
93366	Interventions associated with transplantation of kidney
103429	post-transplantation of kidney examination, recipient
103649	transplantation surgery
104049	interventions associated with transplantation of kidney nos
104050	os interventions associated with transplantation of kidney
105328	Cadaveric renal transplant
105724	unexplained episode of renal transplant dysfunction
105787	Xenograft renal transplant
106620	chronic rejection of renal transplant
106866	vascular complication of renal transplant
107183	discussion about kidney transplantation
107752	urological complication of renal transplant
109455	Allotransplantation of kidney from cadaver NEC
2997	Transplantation of kidney
5504	Transplantation of kidney NOS
5911	[V]Kidney transplanted
11553	Kidney transplant failure and rejection
11745	Transplantation of kidney from live donor
17253	Renal transplant planned
18774	Renal transplant with complication, without blame
24361	Transplantation of kidney from cadaver
26862	Exploration of renal transplant
48057	Renal tubulo-interstitial disordrs in transplant rejectn
48121	Transplant nephrectomy
54990	Kidney transplant with complication, without blame
55151	Autotransplant of kidney
66705	Allotransplantation of kidney from live donor
70874	Other specified transplantation of kidney
89924	Allotransplantation of kidney from cadaver, heart-beating
90952	Pre-transplantation of kidney work-up, recipient
96133	Allotransplantation kidney from cadaver, heart non-beating
98364	Allotransplantation of kidney from cadaver
100693	[X]Renal tubulo-interstitial disorders/transplant rejection
105811	renal transplant rejection
10081	Chronic uraemia

icd	icd_description
I12.0	Hypertensive renal disease with renal failure

I13.1	Hypertensive heart and renal disease with renal failure
I13.2	Hyper heart and renal dis both (cong) heart and renal fail
I77.0	arteriovenous fistula, acquired
N16.5	renal tubulo-interstitial disorders in transplant rejection
N18.0	End-stage renal disease
N18.3	Chronic kidney disease, stage 3
N18.4	Chronic kidney disease, stage 4
N18.5	chronic kidney disease, stage 5
N18.8	Other chronic renal failure
N18.9	Chronic renal failure, unspecified
N19	Unspecified kidney failure
T82.4	mechanical complication of vascular dialysis catheter
T86.1	kidney transplant failure and rejection
Y60.2	during kidney dialysis or other perfusion
Y61.2	during kidney dialysis or other perfusion
Y62.2	during kidney dialysis or other perfusion
Y84.1	kidney dialysis
Z49	care involving dialysis
Z49.0	preparatory care for dialysis
Z49.1	extracorporeal dialysis
Z49.2	other dialysis
Z94.0	kidney transplant status
Z99.2	dependence on renal dialysis

opcs	description
L742	creation of arteriovenous fistula nec
L746	creation of graft fistula for dialysis
M01	Transplantation of kidney
M011	Autotransplantation of kidney
M012	Allotransplantation of kidney from live donor
M013	Allotransplantation of kidney from cadaver NEC
M014	Allotransplantation of kidney from cadaver heart beating
M015	Allotransplantation of kidney from cadaver heart non-beating
M018	Other specified transplantation of kidney
M019	Unspecified transplantation of kidney
M026	Excision of rejected transplanted kidney
M027	excision of transplanted kidney nec
M084	Exploration of transplanted kidney
M172	Pre-transplantation of kidney work-up - recipient
M174	Post-transplantation of kidney examination - recipient
X401	Renal dialysis

X402	Peritoneal dialysis NEC
X403	Haemodialysis NEC
X404	Haemofiltration
X405	Automated peritoneal dialysis
X406	Continuous ambulatory peritoneal dialysis
X41	Placement of ambulatory apparatus for compensation for renal failure
X411	Insertion of ambulatory peritoneal dialysis catheter
X412	Removal of ambulatory peritoneal dialysis catheter
X421	Insertion of temporary peritoneal dialysis catheter

Diabetes

Medcode	Readterm	Diabetes category
54846	Diabetes monitoring deleted	Possible
72333	Listed for Diabetology admissn	Possible
103743	Diabetes care plan declined	Possible
103761	Insulin alert pat information booklet information discussed	Possible
103816	Insulin alert patient information booklet given	Possible
104374	Provision of diabetes clinical summary	Possible
104669	Prof judgemnt not to engage pt wt insulin alert requirements	Possible
105207	Referral to community diabetes clinic	Possible
105446	Insulin administration education	Possible
105937	Referral to community diabetes specialist nurse declined	Possible
106218	Declined diabetic retinopathy screening	Possible
106269	Diabetic retinopathy screening administrative status	Possible
106327	Excluded from diabetic retinopathy screening	Possible
106328	Excluded diabetc retinop screen as under care ophthalmolgist	Possible
106329	Excluded from diabetic retinopathy screening as blind	Possible
106332	Eligible for diabetic retinopathy screening	Possible
106350	Excluded from diabetic retinopathy screening as moved away	Possible
106351	Excluded from diabetic retinop screen as no longer diabetic	Possible
106352	Excluded from diabetic retinopathy screening as deceased	Possible
106441	Ineligible for diabetic retinopathy screening	Possible
106445	Excluded from diabetic retinopathy screen physical disorder	Possible
106679	Provision of written information about diabetes and driving	Possible
106722	Diabetic foot screening invitation second letter	Possible
106723	Diabetic foot screening invitation first letter	Possible
106738	Diabetic foot screening invitation	Possible
106778	Excluded frm diabetic retinopathy screen as terminal illness	Possible
106953	Referral to DAFNE diabetes structured educn prog declined	Possible
107361	Education about diabetes and driving	Possible
107414	Diabetes structured education programme not available	Possible
107452	Further diabetic monitoring	Possible
107597	Excluded from diabetic retinopthy screen as learn disability	Possible
107739	Advice about diabetes and driving	Possible
107793	Diabetic foot screening invitation third letter	Possible
109520	Eligibility permanently inactive for diabetic retinop screen	Possible
109521	Eligibility temporarily inactive for diabetic retinop screen	Possible
104287	Referral to community diabetes service	Possible
608	Follow-up diabetic assessment	Possible
2379	Seen in diabetic clinic	Possible
3550	Diabetic monitoring	Possible
7777	Referral to diabetologist	Possible

8306	Referral to diabetes nurse	Possible
8414	Pt advised re diabetic diet	Possible
8618	Seen by diabetic liaison nurse	Possible
9145	DNA - Did not attend diabetic clinic	Possible
9897	Diabetes monitoring admin.	Possible
11041	Excepted from diabetes qual indicators: Patient unsuitable	Possible
11348	Excepted from diabetes quality indicators: Informed dissent	Possible
11977	Referral to diabetes nurse	Possible
12030	Diabetes monitoring 3rd letter	Possible
12225	Refer, diabetic liaison nurse	Possible
12483	Advice about blood glucose control	Possible
12507	Seen by diabetic liaison nurse	Possible
13067	Diabetic monitoring NOS	Possible
13070	Initial diabetic assessment	Possible
13191	Diabetes clinic administration	Possible
13192	Diabetes monitor. check done	Possible
13194	Diabetes monitoring 1st letter	Possible
13195	Diabetes monitoring 2nd letter	Possible
13197	Attends diabetes monitoring	Possible
13678	Referral to diabetic liaison nurse	Possible
17478	Self monitoring of blood glucose	Possible
17846	Self monitoring of urine glucose	Possible
18824	Diabetic foot examination declined	Possible
20900	Diabetes monitored	Possible
22130	Diabetes monitoring default	Possible
26603	Refuses diabetes monitoring	Possible
28574	Exception reporting: diabetes quality indicators	Possible
31141	Diabetes monitor.phone invite	Possible
31240	Diabetes monitor.verbal invite	Possible
31241	Diabetes monitoring admin.NOS	Possible
32739	Seen in community diabetes specialist clinic	Possible
32770	Glucose tol. test diabetic	Possible
34541	Private referral to diabetologist	Possible
36669	Diabetic monitoring not required	Possible
38103	Seen in diabetic nurse consultant clinic	Possible
38129	Seen in community diabetic specialist nurse clinic	Possible
42217	Self monitoring of blood and urine glucose	Possible
46521	Seen by diabetologist	Possible
47011	Referral to diabetes structured education programme	Possible
47058	Discharged from care of diabetes specialist nurse	Possible
50937	Referral to diabetes preconception counselling clinic	Possible
55123	Date diabetic treatment stopp.	Possible
58133	Discharge by diabetic liaison nurse	Possible

58159	Insulin therapy declined	Possible
58639	Patient held diabetic record declined	Possible
61470	Diabetic monitoring - higher risk albumin excretion	Possible
66475	Diabetic monitoring - lower risk albumin excretion	Possible
69163	Referral to multidisciplinary diabetic clinic	Possible
82474	Referral to community diabetes specialist nurse	Possible
93657	Referral to DESMOND diabetes structured education programme	Possible
93704	Referral to DAFNE diabetes structured education programme	Possible
93854	Diabetes structured education programme declined	Possible
93870	Referral to XPERT diabetes structured education programme	Possible
94330	Referral to diabetes special interest general practitioner	Possible
94955	Did not attend XPERT diabetes structured education programme	Possible
94956	Did not complete XPERT diabetes structured education program	Possible
95093	Did not complete DESMOND diabetes structured educat program	Possible
95094	Did not complete diabetes structured education programme	Possible
95159	Did not attend DESMOND diabetes structured education program	Possible
95553	Did not attend diabetes structured education programme	Possible
95813	Seen in multidisciplinary diabetic clinic	Possible
97281	Seen by general practitioner special interest in diabetes	Possible
97809	Did not complete DAFNE diabetes structured education program	Possible
99277	Did not attend DAFNE diabetes structured education programme	Possible
100422	Discharged from diabetes shared care programme	Possible
100791	Insulin treatment stopped	Possible
101190	Declined consent for diabetes year of care programme	Possible
101455	Diabetes monitor invitation by SMS (short message service)	Possible
101456	Diabetic dietary review declined	Possible
101834	Excepted from diabetes qual indicators: service unavailable	Possible
102316	Suspected diabetes mellitus	Possible
102490	Diabetic assessment of erectile dysfunction	Possible
12682	Patient offered diabetes structured education programme	Possible
12703	Education score - diabetes	Possible
13057	Health education - diabetes	Possible
26605	Attended diabetes structured education programme	Possible
32619	Patient diabetes education review	Possible
34528	Diabetes well being questionnaire	Possible
67664	Education score - diabetes	Possible
68546	Diabetes clinic satisfaction questionnaire	Possible
68818	DTSQ - Diabetes treatment satisfaction questionnaire	Possible
93390	Attended DAFNE diabetes structured education programme	Possible
93491	DAFNE diabetes structured education programme completed	Possible
93529	DESMOND diabetes structured education programme completed	Possible
93631	XPERT diabetes structured education programme completed	Possible
94011	Attended XPERT diabetes structured education programme	Possible

94186	Diabetes structured education programme completed	Possible
94699	Diabetes treatment satisfaction questionnaire	Possible
100436	Education in self management of diabetes	Possible
38130	Diabetes wellbeing questionnaire	Possible
91164	CSQ - Diabetes clinic satisfaction questionnaire	Possible
97824	DWBQ - Diabetes wellbeing questionnaire	Possible
98954	Diabetes treatment satisfaction questionnaire	Possible
30648	Did not attend diabetic retinopathy clinic	Possible
55140	Self monitoring urine ketones	Possible
102434	Diabetic erectile dysfunction review	Possible
102549	Insulin dose	Possible
102768	Did not attend diabetes foot screening	Possible
6430	Attending diabetes clinic	Possible
46963	Insulin-dependent diabetes mellitus with renal complications	Type I diabetes
102946	Insulin-dependent diabetes mellitus with renal complications	Type I diabetes
104453	Type 1 diabetic dietary review	Type I diabetes
105337	Type I diabetes mellitus - poor control	Type I diabetes
108007	Type I diabetes mellitus with multiple complications	Type I diabetes
108724	Type I diabetes mellitus with gastroparesis	Type I diabetes
109051	Insulin dependent diabetes mellitus with gangrene	Type I diabetes
109837	Type I diabetes mellitus with renal complications	Type I diabetes
109878	Diet advice for insulin-dependent diabetes	Type I diabetes
1038	Insulin dependent diabetes mellitus	Type I diabetes
1549	Type 1 diabetes mellitus	Type I diabetes
1647	Insulin dependent diabetes mellitus	Type I diabetes
6509	Insulin dependent diabetes mellitus with retinopathy	Type I diabetes
6791	Insulin dependent diabetes mellitus - poor control	Type I diabetes
10418	Type 1 diabetes mellitus with nephropathy	Type I diabetes
10692	Type 1 diabetes mellitus with ketoacidosis	Type I diabetes
12455	Type I diabetes mellitus	Type I diabetes
17545	Type I diabetes mellitus with diabetic cataract	Type I diabetes
17858	Type 1 diabetes mellitus	Type I diabetes
18230	Type 1 diabetes mellitus with neuropathic arthropathy	Type I diabetes
18387	Type 1 diabetes mellitus with retinopathy	Type I diabetes
18505	IDDM-Insulin dependent diabetes mellitus	Type I diabetes
18642	Type 1 diabetes mellitus with arthropathy	Type I diabetes
18683	Type 1 diabetes mellitus with ulcer	Type I diabetes
21983	Type 1 diabetes mellitus with renal complications	Type I diabetes
22871	Type 1 diabetes mellitus with exudative maculopathy	Type I diabetes
24423	Type I diabetes mellitus	Type I diabetes
24490	Diabetes mellitus, juvenile type, no mention of complication	Type I diabetes
24694	Insulin dependent diabetes mellitus with mononeuropathy	Type I diabetes
26855	Unstable insulin dependent diabetes mellitus	Type I diabetes

30294	Type 1 diabetes mellitus with persistent microalbuminuria	Type I diabetes
30323	Type 1 diabetes mellitus with persistent proteinuria	Type I diabetes
31310	Insulin dependent diabetes maturity onset	Type I diabetes
32359	Perceived control of insulin-dependent diabetes	Type I diabetes
35288	Type 1 diabetes mellitus - poor control	Type I diabetes
38076	Insulin lipohypertrophy	Type I diabetes
38161	Type I diabetes mellitus with retinopathy	Type I diabetes
39070	Type 1 diabetes mellitus with hypoglycaemic coma	Type I diabetes
39809	Insulin dependent diab mell with neuropathic arthropathy	Type I diabetes
40023	Diabetes mellitus, juvenile type, with hyperosmolar coma	Type I diabetes
40682	Type 1 diabetes mellitus maturity onset	Type I diabetes
40837	Type 1 diabetes mellitus with ketoacidotic coma	Type I diabetes
41049	Type 1 diabetes mellitus with retinopathy	Type I diabetes
41716	Insulin dependent diabetes mellitus with polyneuropathy	Type I diabetes
42567	Diabetes mellitus, juvenile type, with ketoacidotic coma	Type I diabetes
42729	Type I diabetes mellitus with hypoglycaemic coma	Type I diabetes
42831	Type 1 diabetes mellitus with neurological complications	Type I diabetes
43493	Insulin site lipohypertrophy	Type I diabetes
43921	Unstable type 1 diabetes mellitus	Type I diabetes
44260	Insulin dependent diabetes mellitus with diabetic cataract	Type I diabetes
44440	Insulin dependent diabetes mellitus with hypoglycaemic coma	Type I diabetes
44443	Insulin dependent diabetes mellitus with ulcer	Type I diabetes
45276	Insulin dependent diabetes mellitus with multiple complicat	Type I diabetes
45914	Type 1 diabetes mellitus - poor control	Type I diabetes
46301	Type 1 diabetes mellitus with polyneuropathy	Type I diabetes
46850	Type I diabetes mellitus - poor control	Type I diabetes
47582	Type 1 diabetes mellitus with renal complications	Type I diabetes
47649	Type 1 diabetes mellitus with ophthalmic complications	Type I diabetes
47650	Type 1 diabetes mellitus with multiple complications	Type I diabetes
49146	Type I diabetes mellitus with neurological complications	Type I diabetes
49276	Insulin-dependent diabetes mellitus with ophthalmic comps	Type I diabetes
49554	Type 1 diabetes mellitus with diabetic cataract	Type I diabetes
49949	Unstable type I diabetes mellitus	Type I diabetes
50960	Pre-existing diabetes mellitus, insulin-dependent	Type I diabetes
51261	Insulin dependent diabetes mellitus	Type I diabetes
51957	Type I diabetes mellitus with ulcer	Type I diabetes
52104	Insulin dependent diabetes mellitus with multiple complicatn	Type I diabetes
52283	Insulin-dependent diabetes mellitus with neurological comps	Type I diabetes
53200	Diabetes mellitus, juvenile type, with ketoacidosis	Type I diabetes
54008	Type 1 diabetes mellitus with neuropathic arthropathy	Type I diabetes
54600	Unstable insulin dependent diabetes mellitus	Type I diabetes
55239	Type 1 diabetes mellitus with gastroparesis	Type I diabetes
56448	Insulin-dependent diabetes without complication	Type I diabetes

57621	Insulin dependent diabetes mellitus with nephropathy	Type I diabetes
60107	Unstable type I diabetes mellitus	Type I diabetes
60208	Type I diabetes mellitus with neuropathic arthropathy	Type I diabetes
60499	Insulin dependent diabetes mellitus with gangrene	Type I diabetes
61344	Type I diabetes mellitus with renal complications	Type I diabetes
61829	Type 1 diabetes mellitus with neurological complications	Type I diabetes
62209	Type I diabetes mellitus with ketoacidosis	Type I diabetes
62352	Type I diabetes mellitus with arthropathy	Type I diabetes
62613	Type I diabetes mellitus without complication	Type I diabetes
63017	Type I diabetes mellitus maturity onset	Type I diabetes
64446	Insulin dependent diab mell with peripheral angiopathy	Type I diabetes
65616	Insulin dependent diabetes mellitus with arthropathy	Type I diabetes
66145	Type I diabetes mellitus with ketoacidotic coma	Type I diabetes
66872	Type I diabetes mellitus with nephropathy	Type I diabetes
67853	Diabetes mellitus, juvenile, + neurological manifestation	Type I diabetes
68105	Type 1 diabetes mellitus with mononeuropathy	Type I diabetes
68390	Type 1 diabetes mellitus with ulcer	Type I diabetes
68792	Diabetes mellitus, juvenile type, + unspecified complication	Type I diabetes
69043	Dietary advice for type I diabetes	Type I diabetes
69124	IDDM with peripheral circulatory disorder	Type I diabetes
69676	Type 1 diabetes mellitus without complication	Type I diabetes
69748	Diabetes mellitus, juvenile type, + ophthalmic manifestation	Type I diabetes
69993	Type 1 diabetes mellitus with gangrene	Type I diabetes
70448	Diabetes mellitus, juvenile +peripheral circulatory disorder	Type I diabetes
70766	Type 1 diabetes mellitus with hypoglycaemic coma	Type I diabetes
72702	Insulin dependent diabetes mellitus - poor control	Type I diabetes
85660	Diabetes type 1 review	Type I diabetes
91942	Type I diabetes mellitus with multiple complications	Type I diabetes
91943	Type I diabetes mellitus with polyneuropathy	Type I diabetes
93468	Type 1 diabetes mellitus with peripheral angiopathy	Type I diabetes
93875	Insulin dependent diabetes mellitus with retinopathy	Type I diabetes
93878	Type I diabetes mellitus with ulcer	Type I diabetes
93922	Diabetes mellitus, juvenile type, with renal manifestation	Type I diabetes
95343	Type I diabetes mellitus with retinopathy	Type I diabetes
95992	Type I diabetes mellitus without complication	Type I diabetes
96235	Type I diabetes mellitus maturity onset	Type I diabetes
97446	Type 1 diabetes mellitus maturity onset	Type I diabetes
97474	Unstable type 1 diabetes mellitus	Type I diabetes
97849	Insulin dependent diabetes maturity onset	Type I diabetes
97894	Type I diabetes mellitus with exudative maculopathy	Type I diabetes
98071	Insulin-dependent diabetes mellitus with ophthalmic comps	Type I diabetes
98392	Maturity onset diabetes in youth type 1	Type I diabetes
98704	Insulin dependent diabetes mellitus with ulcer	Type I diabetes

99231	Type I diabetes mellitus with mononeuropathy	Type I diabetes
99311	Type I diabetes mellitus with ophthalmic complications	Type I diabetes
99716	Insulin dependent diabetes mellitus with hypoglycaemic coma	Type I diabetes
99719	Insulin-dependent diabetes without complication	Type I diabetes
100770	Insulin dependent diabetes mellitus with diabetic cataract	Type I diabetes
101311	Insulin dependent diabetes mellitus with polyneuropathy	Type I diabetes
101735	Insulin-dependent diabetes mellitus with neurological comps	Type I diabetes
102112	Type I diabetes mellitus with gangrene	Type I diabetes
102163	Insulin dependent diabetes mellitus with nephropathy	Type I diabetes
102620	Type I diabetes mellitus with persistent microalbuminuria	Type I diabetes
102704	Type I diabetic dietary review	Type I diabetes
102740	Type 1 diabetes mellitus with ophthalmic complications	Type I diabetes
103902	Type II diabetes mellitus with arthropathy	Type II diabetes
104323	Type II diabetes mellitus with gangrene	Type II diabetes
104639	Type II diabetes mellitus with peripheral angiopathy	Type II diabetes
105784	Type 2 diabetes mellitus without complication	Type II diabetes
106061	Type II diabetes mellitus with ketoacidotic coma	Type II diabetes
106528	Type II diabetes mellitus with ketoacidosis	Type II diabetes
107701	Hyperosmolar non-ketotic state in type II diabetes mellitus	Type II diabetes
108005	Type 2 diabetes mellitus with multiple complications	Type II diabetes
109103	Type II diabetes mellitus without complication	Type II diabetes
109197	Type II diabetes mellitus with neuropathic arthropathy	Type II diabetes
109865	Type 2 diabetes mellitus with polyneuropathy	Type II diabetes
506	Non-insulin dependent diabetes mellitus	Type II diabetes
758	Type 2 diabetes mellitus	Type II diabetes
1407	Insulin treated Type 2 diabetes mellitus	Type II diabetes
4513	Non-insulin dependent diabetes mellitus	Type II diabetes
5884	NIDDM - Non-insulin dependent diabetes mellitus	Type II diabetes
8403	Non-insulin dependent diabetes mellitus - poor control	Type II diabetes
12640	Type 2 diabetes mellitus with nephropathy	Type II diabetes
12736	Type 2 diabetes mellitus with gangrene	Type II diabetes
14803	Diabetes mellitus, adult onset, no mention of complication	Type II diabetes
14889	Maturity onset diabetes	Type II diabetes
17262	Non-insulin-dependent diabetes mellitus with retinopathy	Type II diabetes
17859	Type 2 diabetes mellitus	Type II diabetes
18143	Type II diabetes mellitus with arthropathy	Type II diabetes
18209	Type 2 diabetes mellitus with renal complications	Type II diabetes
18219	Type II diabetes mellitus	Type II diabetes
18264	Insulin treated Type II diabetes mellitus	Type II diabetes
18278	Insulin treated Type 2 diabetes mellitus	Type II diabetes
18390	Type 2 diabetes mellitus with persistent microalbuminuria	Type II diabetes
18425	Type 2 diabetes mellitus with polyneuropathy	Type II diabetes
18496	Type 2 diabetes mellitus with retinopathy	Type II diabetes

18777	Type 2 diabetes mellitus with renal complications	Type II diabetes
22884	Type II diabetes mellitus	Type II diabetes
24458	Type II diabetes mellitus - poor control	Type II diabetes
24693	Non-insulin dependent diabetes mellitus with arthropathy	Type II diabetes
24836	Type 2 diabetes mellitus with nephropathy	Type II diabetes
25041	Dietary advice for type II diabetes	Type II diabetes
25591	Type 2 diabetes mellitus with exudative maculopathy	Type II diabetes
25627	Type 2 diabetes mellitus - poor control	Type II diabetes
26054	Type 2 diabetes mellitus with persistent proteinuria	Type II diabetes
29979	Non-insulin-dependent diabetes mellitus without complication	Type II diabetes
32627	Type 2 diabetes mellitus with ketoacidosis	Type II diabetes
33807	Diabetes mellitus, adult with gangrene	Type II diabetes
34268	Type 2 diabetes mellitus with neurological complications	Type II diabetes
34450	Hyperosmolar non-ketotic state in type 2 diabetes mellitus	Type II diabetes
34912	Non-insulin dependent diabetes mellitus with ulcer	Type II diabetes
35105	Diabetes mellitus, adult onset, with renal manifestation	Type II diabetes
35385	Type 2 diabetes mellitus with neuropathic arthropathy	Type II diabetes
36633	Hyperosmolar non-ketotic state in type 2 diabetes mellitus	Type II diabetes
36695	Diabetes mellitus autosomal dominant type 2	Type II diabetes
37648	Insulin treated non-insulin dependent diabetes mellitus	Type II diabetes
37806	Type 2 diabetes mellitus with peripheral angiopathy	Type II diabetes
39317	Diabetes mellitus, adult onset, + neurological manifestation	Type II diabetes
40401	Non-insulin dependent diabetes mellitus with gangrene	Type II diabetes
40962	Non-insulin dependent d m with neuropathic arthropathy	Type II diabetes
41389	Diabetes mellitus, adult onset, + ophthalmic manifestation	Type II diabetes
42762	Type 2 diabetes mellitus with retinopathy	Type II diabetes
43139	Diabetes mellitus, adult onset, with hyperosmolar coma	Type II diabetes
43227	Type II diabetes mellitus with multiple complications	Type II diabetes
43785	Non-insulin dependent diabetes mellitus with hypoglyca coma	Type II diabetes
44779	Type 2 diabetes mellitus with diabetic cataract	Type II diabetes
44982	Type 2 diabetes mellitus with diabetic cataract	Type II diabetes
45467	Non-insulin dependent diabetes mellitus with polyneuropathy	Type II diabetes
45913	Type 2 diabetes mellitus - poor control	Type II diabetes
45919	Type 2 diabetes mellitus with neurological complications	Type II diabetes
46150	Type 2 diabetes mellitus with gangrene	Type II diabetes
46624	Maturity onset diabetes in youth	Type II diabetes
46917	Type 2 diabetes mellitus with hypoglycaemic coma	Type II diabetes
47315	Type II diabetes mellitus - poor control	Type II diabetes
47321	Type 2 diabetes mellitus with ophthalmic complications	Type II diabetes
47409	Type II diabetes mellitus with polyneuropathy	Type II diabetes
47816	Type II diabetes mellitus with neuropathic arthropathy	Type II diabetes
47954	Type 2 diabetes mellitus without complication	Type II diabetes
48192	Type II diabetes mellitus with diabetic cataract	Type II diabetes

49074	Type 2 diabetes mellitus with ulcer	Type II diabetes
49655	Type II diabetes mellitus with retinopathy	Type II diabetes
49869	Type 2 diabetes mellitus with arthropathy	Type II diabetes
50225	Type II diabetes mellitus with renal complications	Type II diabetes
50429	Non-insulin-dependent diabetes mellitus with ophthalm comps	Type II diabetes
50527	Type II diabetes mellitus with polyneuropathy	Type II diabetes
50609	Pre-existing diabetes mellitus, non-insulin-dependent	Type II diabetes
50813	Type II diabetes mellitus with mononeuropathy	Type II diabetes
51756	Type 2 diabetes mellitus with ketoacidotic coma	Type II diabetes
52303	Non-insulin-dependent diabetes mellitus with renal comps	Type II diabetes
53392	Type II diabetes mellitus without complication	Type II diabetes
54212	Non-insulin-dependent d m with peripheral angiopath	Type II diabetes
54856	Diabetes mellitus, adult onset, with ketoacidosis	Type II diabetes
54899	Type II diabetes mellitus with peripheral angiopathy	Type II diabetes
55075	Type II diabetes mellitus with ulcer	Type II diabetes
55842	Non-insulin-dependent diabetes mellitus with neuro comps	Type II diabetes
56268	Type II diabetes mellitus with hypoglycaemic coma	Type II diabetes
56803	NIDDM with peripheral circulatory disorder	Type II diabetes
57278	Type II diabetes mellitus with renal complications	Type II diabetes
58604	Type II diabetes mellitus with retinopathy	Type II diabetes
59253	Type 2 diabetes mellitus with arthropathy	Type II diabetes
59365	Non-insulin dependent diabetes mellitus with nephropathy	Type II diabetes
59725	Type II diabetes mellitus with ophthalmic complications	Type II diabetes
59991	Maturity onset diabetes in youth type 2	Type II diabetes
60699	Type 2 diabetes mellitus with peripheral angiopathy	Type II diabetes
60796	Type II diabetes mellitus with persistent proteinuria	Type II diabetes
61071	Type 2 diabetes mellitus with hypoglycaemic coma	Type II diabetes
62107	Type II diabetes mellitus with gangrene	Type II diabetes
62146	Non-insulin-dependent diabetes mellitus with multiple comps	Type II diabetes
62674	Type 2 diabetes mellitus with mononeuropathy	Type II diabetes
63357	Diabetes mellitus, adult, + peripheral circulatory disorder	Type II diabetes
63371	Diabetes mellitus, adult, + other specified manifestation	Type II diabetes
63690	Type 2 diabetes mellitus with gastroparesis	Type II diabetes
63762	Diabetes mellitus, adult onset, + unspecified complication	Type II diabetes
64571	Type II diabetes mellitus with nephropathy	Type II diabetes
64668	Insulin treated Type II diabetes mellitus	Type II diabetes
65267	Type 2 diabetes mellitus with multiple complications	Type II diabetes
65704	Type 2 diabetes mellitus with ulcer	Type II diabetes
66965	Type 2 diabetes mellitus with neuropathic arthropathy	Type II diabetes
67905	Type II diabetes mellitus with neurological complications	Type II diabetes
68843	Diabetes mellitus, adult onset, with ketoacidotic coma	Type II diabetes
69278	Non-insulin depend diabetes mellitus with diabetic cataract	Type II diabetes
70316	Type 2 diabetes mellitus with ophthalmic complications	Type II diabetes

72320	Non-insulin dependent diabetes mellitus with mononeuropathy	Type II diabetes
83532	Diabetes type 2 review	Type II diabetes
85991	Type II diabetes mellitus with persistent microalbuminuria	Type II diabetes
91646	Type II diabetes mellitus with ulcer	Type II diabetes
93727	Type II diabetes mellitus with diabetic cataract	Type II diabetes
95351	Type II diabetes mellitus with mononeuropathy	Type II diabetes
95636	Latent autoimmune diabetes mellitus in adult	Type II diabetes
98616	Type II diabetes mellitus with neurological complications	Type II diabetes
98723	Type II diabetes mellitus with hypoglycaemic coma	Type II diabetes
100964	Type II diabetes mellitus with ophthalmic complications	Type II diabetes
101801	Type II diabetic dietary review	Type II diabetes
102201	Type II diabetes mellitus with nephropathy	Type II diabetes
102611	Type 2 diabetic dietary review	Type II diabetes
13196	Fundoscopy - diabetic check	Diabetes, unspecified
103798	Diabetic medicine	Diabetes, unspecified
107423	Diabetes self-management plan review	Diabetes, unspecified
9974	Seen in diabetic eye clinic	Diabetes, unspecified
10824	Seen in diabetic foot clinic	Diabetes, unspecified
61557	Diabetology D.V. requested	Diabetes, unspecified
103762	Insulin passport completed	Diabetes, unspecified
103772	Insulin passport given	Diabetes, unspecified
103817	Informed dissent not to carry insulin passport	Diabetes, unspecified
105585	Diabetes clinical pathway	Diabetes, unspecified
108655	Insulin passport not checked	Diabetes, unspecified
45499	Kimmelstiel - Wilson disease	Diabetes, unspecified
103935	No evidence of diabetic nephropathy	Diabetes, unspecified
104254	Subcutaneous infusion with insulin pump	Diabetes, unspecified
104858	Insulin passport checked	Diabetes, unspecified
105302	Proteinuric diabetic nephropathy	Diabetes, unspecified
105740	O/E - Left diabetic foot at increased risk	Diabetes, unspecified
105741	O/E - Right diabetic foot at increased risk	Diabetes, unspecified
106360	Erectile dysfunction due to diabetes mellitus	Diabetes, unspecified
107331	Conversion to insulin in secondary care	Diabetes, unspecified
107464	Diabetes Year of Care annual review	Diabetes, unspecified
107508	Conversion to insulin by diabetes specialist nurse	Diabetes, unspecified
107881	Clinical diabetic nephropathy	Diabetes, unspecified
108993	Diabetes self-management plan agreed	Diabetes, unspecified
108634	In-house diabetic foot screening	Diabetes, unspecified
711	Diabetes mellitus	Diabetes, unspecified
1323	Diabetic retinopathy	Diabetes, unspecified
1682	Diabetes mellitus with ketoacidosis	Diabetes, unspecified
1684	Diabetic on oral treatment	Diabetes, unspecified
2340	Diabetic amyotrophy	Diabetes, unspecified

2342	Diabetic neuropathy	Diabetes, unspecified
2378	Diabetic - poor control	Diabetes, unspecified
2471	Nephrotic syndrome in diabetes mellitus	Diabetes, unspecified
2475	Diabetic nephropathy	Diabetes, unspecified
2478	Brittle diabetes	Diabetes, unspecified
2986	Preproliferative diabetic retinopathy	Diabetes, unspecified
3286	Proliferative diabetic retinopathy	Diabetes, unspecified
3837	Diabetic maculopathy	Diabetes, unspecified
5002	Diabetic polyneuropathy	Diabetes, unspecified
6125	Diabetic annual review	Diabetes, unspecified
7059	Admit diabetic emergency	Diabetes, unspecified
7069	Background diabetic retinopathy	Diabetes, unspecified
7328	Cellulitis in diabetic foot	Diabetes, unspecified
7563	Diabetic on diet only	Diabetes, unspecified
7795	Diabetes mellitus with neuropathy	Diabetes, unspecified
8836	Diabetes management plan given	Diabetes, unspecified
8842	Diabetic on insulin	Diabetes, unspecified
9013	Unstable diabetes	Diabetes, unspecified
9835	O/E - diabetic maculopathy present both eyes	Diabetes, unspecified
9881	Mixed diabetic ulcer - foot	Diabetes, unspecified
9958	Hb. A1C - diabetic control	Diabetes, unspecified
10098	Other specified diabetes mellitus with other spec comps	Diabetes, unspecified
10099	Advanced diabetic maculopathy	Diabetes, unspecified
10642	Dietary advice for diabetes mellitus	Diabetes, unspecified
10659	Diabetic cataract	Diabetes, unspecified
10755	Non proliferative diabetic retinopathy	Diabetes, unspecified
10977	Diabetic peripheral neuropathy screening	Diabetes, unspecified
11018	Diabetic retinopathy 12 month review	Diabetes, unspecified
11047	Conversion to insulin	Diabetes, unspecified
11094	Under care of diabetic foot screener	Diabetes, unspecified
11129	O/E - left eye background diabetic retinopathy	Diabetes, unspecified
11433	O/E - right eye background diabetic retinopathy	Diabetes, unspecified
11471	Diabetes medication review	Diabetes, unspecified
11599	Pan retinal photocoagulation for diabetes	Diabetes, unspecified
11626	Diabetic retinopathy NOS	Diabetes, unspecified
11663	Neuropathic diabetic ulcer - foot	Diabetes, unspecified
11677	Refer to diabetic foot screener	Diabetes, unspecified
11930	Under care of diabetes specialist nurse	Diabetes, unspecified
12213	Patient on maximal tolerated therapy for diabetes	Diabetes, unspecified
12247	Diabetic foot examination not indicated	Diabetes, unspecified
12262	Diabetic retinopathy screening refused	Diabetes, unspecified
12307	Diabetes care by hospital only	Diabetes, unspecified
12506	Diabetes: practice programme	Diabetes, unspecified

12675	Diabetes: shared care programme	Diabetes, unspecified
13069	Has seen dietician - diabetes	Diabetes, unspecified
13071	Diabetic - good control	Diabetes, unspecified
13074	Diabetic diet	Diabetes, unspecified
13078	Diabetic weight reducing diet	Diabetes, unspecified
13097	O/E - right eye proliferative diabetic retinopathy	Diabetes, unspecified
13099	O/E - right eye preproliferative diabetic retinopathy	Diabetes, unspecified
13100	O/E - no right diabetic retinopathy	Diabetes, unspecified
13101	O/E - left eye proliferative diabetic retinopathy	Diabetes, unspecified
13102	O/E - right eye diabetic maculopathy	Diabetes, unspecified
13103	O/E - left eye preproliferative diabetic retinopathy	Diabetes, unspecified
13104	O/E - no left diabetic retinopathy	Diabetes, unspecified
13108	O/E - left eye diabetic maculopathy	Diabetes, unspecified
13279	Other specified diabetes mellitus with renal complications	Diabetes, unspecified
14049	Hb. A1C - diabetic control NOS	Diabetes, unspecified
14050	HbA1 - diabetic control	Diabetes, unspecified
15690	Diabetes mellitus with ketoacidotic coma	Diabetes, unspecified
16230	Diabetes mellitus with neurological manifestation	Diabetes, unspecified
16490	Diabetic treatment changed	Diabetes, unspecified
16491	Diabetes mellitus with polyneuropathy	Diabetes, unspecified
16502	Diabetes mellitus with renal manifestation	Diabetes, unspecified
16881	[V]Dietary counselling in diabetes mellitus	Diabetes, unspecified
17067	Autonomic neuropathy due to diabetes	Diabetes, unspecified
17095	O/E - Right diabetic foot at risk	Diabetes, unspecified
17247	Diabetic mononeuritis NOS	Diabetes, unspecified
17313	Diabetic iritis	Diabetes, unspecified
17817	Subcutaneous injection of insulin	Diabetes, unspecified
17869	Diabetic-uncooperative patient	Diabetes, unspecified
17886	Diabetic - follow-up default	Diabetes, unspecified
18056	Foot abnormality - diabetes related	Diabetes, unspecified
18142	Diabetic cheiroarthropathy	Diabetes, unspecified
18167	Annual diabetic blood test	Diabetes, unspecified
18311	Diabetic retinopathy screening	Diabetes, unspecified
18662	Diabetic retinopathy 6 month review	Diabetes, unspecified
18747	Diabetic retinopathy screening not indicated	Diabetes, unspecified
19381	Referral to diabetic eye clinic	Diabetes, unspecified
19739	Diabetic retinopathy screening offered	Diabetes, unspecified
20696	Injection sites - diabetic	Diabetes, unspecified
21482	Diabetes mellitus with hyperosmolar coma	Diabetes, unspecified
21689	Diabetic lipid lowering diet	Diabetes, unspecified
22023	Diabetic - poor control NOS	Diabetes, unspecified
22573	Diabetes mellitus NOS with neurological manifestation	Diabetes, unspecified
22823	Diabetic foot examination	Diabetes, unspecified

22967	Retinal abnormality - diabetes related	Diabetes, unspecified
24327	Ischaemic ulcer diabetic foot	Diabetes, unspecified
24363	Diabetic stabilisation	Diabetes, unspecified
24571	Asymptomatic diabetic neuropathy	Diabetes, unspecified
25636	Diabetic diet - poor compliance	Diabetes, unspecified
26604	Diabetic diet - good compliance	Diabetes, unspecified
26664	O/E - Left diabetic foot at risk	Diabetes, unspecified
26666	O/E - Right diabetic foot at low risk	Diabetes, unspecified
26667	O/E - Left diabetic foot at low risk	Diabetes, unspecified
27891	Diabetic Charcot arthropathy	Diabetes, unspecified
27921	Foot abnormality - diabetes related	Diabetes, unspecified
28769	Diabetic on insulin and oral treatment	Diabetes, unspecified
28856	Transition of diabetes care options discussed	Diabetes, unspecified
28873	Diabetic 6 month review	Diabetes, unspecified
29041	Date diabetic treatment start	Diabetes, unspecified
30477	High risk proliferative diabetic retinopathy	Diabetes, unspecified
31053	[D]Widespread diabetic foot gangrene	Diabetes, unspecified
31156	O/E - Left diabetic foot at moderate risk	Diabetes, unspecified
31157	O/E - Right diabetic foot at moderate risk	Diabetes, unspecified
31171	O/E - Right diabetic foot at high risk	Diabetes, unspecified
31172	O/E - Left diabetic foot at high risk	Diabetes, unspecified
31790	Polyneuropathy in diabetes	Diabetes, unspecified
32403	Diabetes mellitus with gangrene	Diabetes, unspecified
32556	Diabetes with gangrene	Diabetes, unspecified
33254	Diabetes mellitus with ophthalmic manifestation	Diabetes, unspecified
33343	Diabetes mellitus with other specified manifestation	Diabetes, unspecified
34152	Diabetic peripheral angiopathy	Diabetes, unspecified
34283	Diabetes mellitus NOS with ophthalmic manifestation	Diabetes, unspecified
35107	Diabetes mellitus with nephropathy NOS	Diabetes, unspecified
35116	O/E - Left diabetic foot - ulcerated	Diabetes, unspecified
35316	O/E - Right diabetic foot - ulcerated	Diabetes, unspecified
35321	Non-urgent diabetic admission	Diabetes, unspecified
35383	Diabetic patient unsuitable for digital retinal photography	Diabetes, unspecified
35399	Diabetes mellitus with peripheral circulatory disorder	Diabetes, unspecified
35785	Chronic painful diabetic neuropathy	Diabetes, unspecified
36798	Continuous subcutaneous infusion of insulin	Diabetes, unspecified
37315	Diabetic mononeuropathy	Diabetes, unspecified
38078	Understands diet - diabetes	Diabetes, unspecified
38617	Other specified diabetes mellitus with ketoacidosis	Diabetes, unspecified
38986	Diabetes mellitus with no mention of complication	Diabetes, unspecified
39420	Myasthenic syndrome due to diabetic amyotrophy	Diabetes, unspecified
41686	[X]Other specified diabetes mellitus	Diabetes, unspecified
42505	Diabetes mellitus NOS with ketoacidosis	Diabetes, unspecified

43453	Diabetes mellitus autosomal dominant	Diabetes, unspecified
43857	Lipoatrophic diabetes mellitus	Diabetes, unspecified
43951	Diabetic - cooperative patient	Diabetes, unspecified
44033	Diabetic mononeuritis multiplex	Diabetes, unspecified
45491	Diabetes mellitus with unspecified complication	Diabetes, unspecified
46290	Other specified diabetes mellitus with multiple comps	Diabetes, unspecified
47032	Diabetes care plan agreed	Diabetes, unspecified
47144	O/E - diabetic maculopathy absent both eyes	Diabetes, unspecified
47328	O/E - right eye stable treated prolif diabetic retinopathy	Diabetes, unspecified
47341	Diabetic crisis monitoring	Diabetes, unspecified
47370	Diabetology D.V. done	Diabetes, unspecified
47377	Other specified diabetes mellitus with ophthalmic complicatn	Diabetes, unspecified
47584	Advanced diabetic retinal disease	Diabetes, unspecified
48078	Acute painful diabetic neuropathy	Diabetes, unspecified
48310	[V]Admitted for commencement of insulin	Diabetes, unspecified
49640	O/E - left chronic diabetic foot ulcer	Diabetes, unspecified
49884	Diabetic pre-pregnancy counselling	Diabetes, unspecified
50175	Diabetic foot risk assessment	Diabetes, unspecified
50972	Diabetes mellitus NOS with no mention of complication	Diabetes, unspecified
51939	[V]Admitted for conversion to insulin	Diabetes, unspecified
52041	O/E - left eye stable treated prolif diabetic retinopathy	Diabetes, unspecified
52212	[X]Diabetes mellitus	Diabetes, unspecified
52237	Patient held diabetic record issued	Diabetes, unspecified
52630	O/E - sight threatening diabetic retinopathy	Diabetes, unspecified
53630	Insulin coma	Diabetes, unspecified
53634	[D]Gangrene of toe in diabetic	Diabetes, unspecified
55431	Pre-existing diabetes mellitus, unspecified	Diabetes, unspecified
57333	Diabetic cheiropathy	Diabetes, unspecified
57389	Patient consent given for addition to diabetic register	Diabetes, unspecified
57723	Referral to diabetic register	Diabetes, unspecified
59288	Other specified diabetes mellitus with coma	Diabetes, unspecified
59903	Diabetic amyotrophy	Diabetes, unspecified
61021	Diabetic digital retinopathy screening offered	Diabetes, unspecified
61461	Informed consent for diabetes national audit	Diabetes, unspecified
61523	Other specified diabetes mellitus with neurological comps	Diabetes, unspecified
61670	Diab mellit insulin-glucose infus acute myocardial infarct	Diabetes, unspecified
62384	O/E - right chronic diabetic foot ulcer	Diabetes, unspecified
63412	Diabetes clinical management plan	Diabetes, unspecified
64142	Referral for diabetic retinopathy screening	Diabetes, unspecified
64283	Other specified diabetes mellitus with unspecified comps	Diabetes, unspecified
64357	Diabetes mellitus NOS with unspecified complication	Diabetes, unspecified
64449	Unspecified diabetes mellitus with multiple complications	Diabetes, unspecified
65025	Diabetes mellitus NOS with peripheral circulatory disorder	Diabetes, unspecified

65062	Diabetes mellitus NOS with ketoacidotic coma	Diabetes, unspecified
65463	High risk non proliferative diabetic retinopathy	Diabetes, unspecified
66274	Insulin needles changed for each injection	Diabetes, unspecified
69152	Insulin needles changed less than once a day	Diabetes, unspecified
70821	Diabetes mellitus NOS with other specified manifestation	Diabetes, unspecified
72345	Diabetes mellitus NOS with hyperosmolar coma	Diabetes, unspecified
83485	Insulin dose changed	Diabetes, unspecified
90301	Insulin needles changed daily	Diabetes, unspecified
95994	Diabetic foot screen	Diabetes, unspecified
96010	Insulin treatment initiated	Diabetes, unspecified
96143	Insulin initiation - enhanced services administration	Diabetes, unspecified
99628	[X]Glomerular disorders in diabetes mellitus	Diabetes, unspecified
100292	[X]Unspecified diabetes mellitus with renal complications	Diabetes, unspecified
101177	Diabetic dietary review	Diabetes, unspecified
101728	Diabetic on subcutaneous treatment	Diabetes, unspecified
101881	Impaired vision due to diabetic retinopathy	Diabetes, unspecified
102767	Pre-conception advice for diabetes mellitus	Diabetes, unspecified
44312	Informed dissent for diabetes national audit	Diabetes, unspecified
45250	Under care of diabetic liaison nurse	Diabetes, unspecified
54601	Under care of diabetologist	Diabetes, unspecified

icd	icd_description	diab_cat
E10	Insulin-dependent diabetes mellitus	Type I diabetes
E10.0	Insulin-dependent diabetes mellitus with coma	Type I diabetes
E10.1	Insulin-dependent diabetes mellitus with ketoacidosis	Type I diabetes
E10.2	Insulin-dependent diabetes mellitus with renal complications	Type I diabetes
E10.3	Insulin-dependent diabetes mellitus with ophthalmic comps	Type I diabetes
E10.4	Insulin-dependent diabetes mellitus with neurological comps	Type I diabetes
E10.5	Insulin-dependent diabetes mellitus with periph circ comps	Type I diabetes
E10.6	Insulin-dependent diabetes mellitus with other spec comps	Type I diabetes
E10.7	Insulin-dependent diabetes mellitus with multiple comps	Type I diabetes
E10.8	Insulin-dependent diabetes mellitus with unspec comps	Type I diabetes
E10.9	Insulin-dependent diabetes mellitus without complications	Type I diabetes
E11	Non-insulin-dependent diabetes mellitus	Type II diabetes
E11.0	Non-insulin-dependent diabetes mellitus with coma	Type II diabetes
E11.1	Non-insulin-dependent diabetes mellitus with ketoacidosis	Type II diabetes
E11.2	Non-insulin-dependent diabetes mellitus with renal comps	Type II diabetes
E11.3	Non-insulin-dependent diabetes mellitus with ophthalm comps	Type II diabetes
E11.4	Non-insulin-dependent diabetes mellitus with neuro comps	Type II diabetes
E11.5	Non-insulin-depend diabetes mellitus with periph circ comp	Type II diabetes
E11.6	Non-insulin-depend diabetes mellitus with other spec comp	Type II diabetes

E11.7	Non-insulin-dependent diabetes mellitus with multiple comps	Type II diabetes
E11.8	Non-insulin-dependent diabetes mellitus with unspec comps	Type II diabetes
E11.9	Non-insulin-depend diabetes mellitus without complication	Type II diabetes
E14	Unspecified diabetes mellitus	Diabetes, unspecified
E14.0	Unspecified diabetes mellitus with coma	Diabetes, unspecified
E14.1	Unspecified diabetes mellitus with ketoacidosis	Diabetes, unspecified
E14.2	Unspecified diabetes mellitus with renal complications	Diabetes, unspecified
E14.3	Unspecified diabetes mellitus with ophthalmic complications	Diabetes, unspecified
E14.4	Unspecified diabetes mellitus with neurological comps	Diabetes, unspecified
E14.5	Unspecified diabetes mellitus with periph circulatory comps	Diabetes, unspecified
E14.6	Unspecified diabetes mellitus with other specified comps	Diabetes, unspecified
E14.7	Unspecified diabetes mellitus with multiple complications	Diabetes, unspecified
E14.8	Unspecified diabetes mellitus with unspecified complications	Diabetes, unspecified
E14.9	Unspecified diabetes mellitus without complications	Diabetes, unspecified
G59.0	Diabetic mononeuropathy	Diabetes, unspecified
G63.2	Diabetic polyneuropathy	Diabetes, unspecified
H28.0	Diabetic cataract	Diabetes, unspecified
H36.0	Diabetic retinopathy	Diabetes, unspecified
M14.2	Diabetic arthropathy	Diabetes, unspecified
N08.3	Glomerular disorders in diabetes mellitus	Diabetes, unspecified
O24.0	Pre-existing diabetes mellitus, insulin-dependent	Type I diabetes
O24.1	Pre-existing diabetes mellitus, non-insulin-dependent	Type II diabetes
O24.3	Pre-existing diabetes mellitus, unspecified	Diabetes, unspecified

Antidiabetics

Prodcode	Product name	Subtype	Any metform	Mono-metformin
23	metformin tablets 500mg	oad	1	1
32	gliclazide tablets 80mg	oad		
93	metformin tablets 850mg	oad	1	1
321	insulin human actrapid (neutral) 40 i/u inj	insulin		
322	humalog injection 100 iu/ml [lilly]	insulin		
469	rosiglitazone tablets 4mg	oad		
479	acarbose tablets 50mg	oad		
547	glipizide tablets 2.5mg	oad		
548	pioglitazone tablets 15mg	oad		
735	metformin oral suspension 100mg/ml	oad	1	1
1253	chlorpropamide tablets 100mg	oad		
1254	glibenclamide tablets 5mg	oad		
1587	monotard injection 100 units/ml [novo]	insulin		
1588	actrapid injection 100 iu/ml [novo]	insulin		
1592	actrapid penfill 100 iu/ml [novo]	insulin		
1593	insulatard penfill 100 iu/ml [novo]	insulin		
1594	actrapid novolet 100 iu/ml [novo]	insulin		
1595	insulatard novolet 100 iu/ml [novo]	insulin		
1643	insulin novo monotard mc 100 i/u inj	insulin		
1645	insulin novo actrapid mc 100 i/u inj	insulin		
1649	human actraphane injection 100 iu/ml [novo]	insulin		
1805	mixtard 30/70 injection 100 units/ml [novo]	insulin		
1806	penmix 30/70 penfill injection 100 iu/ml [novo]	insulin		
1839	insulin humulin i (isophane) 100 i/u inj	insulin		
1840	humulin s injection 100 units/ml [lilly]	insulin		
1842	pork velosulin injection 100 units/ml [novo]	insulin		
1843	pork insulatard vial injection suspension 100 units/ml [novo]	insulin		
1844	ultratard injection 100 units/ml [novo]	insulin		
1847	chlorpropamide tablets 250mg	oad		
1886	insulatard ge injection 100 iu/ml [novo]	insulin		
1964	diamicron tablets 80mg [servier]	oad		
1965	tolbutamide tablets 500mg	oad		
2219	glibenclamide tablets 2.5mg	oad		
2220	penmix 20/80 pen [novo]	insulin		
2221	mixtard 30 novolet 100 iu/ml [novo]	insulin		
2373	insulin human velosulin 100 i/u inj	insulin		
2454	mixtard 30 penfill 100 iu/ml [novo]	insulin		
2455	mixtard 20 novolet 100 iu/ml [novo]	insulin		
2456	mixtard 10 novolet 100 iu/ml [novo]	insulin		
2459	pork mixtard 30 vial injection suspension 100 units/ml [novo]	insulin		

2808	insulin lentard inj	insulin		
2812	mixtard 40 novolet 100 iu/ml [novo]	insulin		
2928	metformin hcl 850 mg tab	oad	1	1
2929	mixtard 30 ge injection 100 iu/ml [novo]	insulin		
3252	metformin hcl 500 mg tab	oad	1	1
3396	penmix 10/90 penfill penfill [novo]	insulin		
3439	penmix 10/90 pen [novo]	insulin		
3550	mixtard 40 penfill 100 iu/ml [novo]	insulin		
3551	mixtard 20 penfill 100 iu/ml [novo]	insulin		
4093	humulin m2 injection 100 units/ml [lilly]	insulin		
4129	insulin soluble porcine injection 100 units/ml	insulin		
4163	rapitard mc injection 100 units/ml [novo]	insulin		
4198	humulin m3 injection 100 units/ml [lilly]	insulin		
4199	humulin m1 injection 100 units/ml [lilly]	insulin		
4247	insulin isophane porcine injection 100 units/ml	insulin		
4248	insulin novo ultratard mc 100 i/u inj	insulin		
4426	CHLORPROPAMIDE 500 MG TAB	oad		
4706	velosulin vial injection solution 100 units/ml [novo]	insulin		
4715	humalog mix 25 injection 25:75; 100 units/ml [lilly]	insulin		
4760	humulin i injection 100 units/ml [lilly]	insulin		
4784	lentard mc injection 100 units/ml [novo]	insulin		
4790	mixtard 50 penfill 100 iu/ml [novo]	insulin		
4862	diabetamide tablets 2.5mg [ashbourne]	oad		
5021	novorapid penfill injection solution 100 units/ml [novo]	insulin		
5174	acarbose tablets 100mg	oad		
5214	insulin lispro human prb injection 100 iu/ml	insulin		
5227	rosiglitazone tablets 8mg	oad		
5250	insulin biphasic lispro human prb injection 25:75; 100 units/ml	insulin		
5255	mixtard 10 penfill 100 iu/ml [novo]	insulin		
5276	glimepiride tablets 1mg	oad		
5316	glimepiride tablets 4mg	oad		
5353	glimepiride tablets 2mg	oad		
5501	insuman basal injection 100 iu/ml [aventis]	insulin		
5621	GLUCOBAY tablets 50mg [BAYER]	oad		
5627	gliclazide modified release tablet 30mg	oad		
5636	glipizide tablets 5mg	oad		
5678	nateglinide tablets 120mg	oad		
5845	mixtard 30 innolet injection suspension 30:70; 100 units/ml [novo]	insulin		
5891	insulatard flexpen injection 100 iu/ml [novo]	insulin		
5892	novorapid flexpen injection solution 100 units/ml [novo]	insulin		
5933	mixtard 50 novolet 100 iu/ml [novo]	insulin		
5953	insulin glargine injection 100 iu/ml	insulin		
5989	nateglinide tablets 180mg	oad		

6057	lantus injection 100 iu/ml [aventis]	insulin		
6061	novomix 30 injection 30:70; 100 units/ml [novo]	insulin		
6209	novorapid vial injection solution 100 units/ml [novo]	insulin		
6337	glimepiride tablets 3mg	oad		
6447	insulin aspart human pyr injection 100 iu/ml	insulin		
6855	avandamet tablets 2mg + 500mg [glaxsk pha]	oad	1	
6958	levemir flexpen injection solution 100 units/ml [novo]	insulin		
6965	levemir penfill injection solution 100 units/ml [novo]	insulin		
7048	metformin modified release tablet 500mg	oad	1	1
7166	glucophage tablets 500mg [merck ser]	oad	1	1
7228	novomix 30 flexpen injection suspension 100 units/ml [novo]	insulin		
7231	mixtard 30 penfill injection suspension 100 units/ml [novo]	insulin		
7237	lantus optiset injection solution 100 units/ml [aventis]	insulin		
7266	lantus cartridge injection solution 100 units/ml [aventis]	insulin		
7267	novomix 30 penfill injection suspension 100 units/ml [novo]	insulin		
7284	amaryl tablets 2mg [aventis]	oad		
7300	mixtard 30 vial injection suspension 100 units/ml [novo]	insulin		
7318	humalog cartridge injection solution 100 units/ml [lilly]	insulin		
7319	mixtard 20 penfill injection suspension 100 units/ml [novo]	insulin		
7325	avandamet tablets 4mg + 1000mg [glaxsk pha]	oad	1	
7332	amaryl tablets 1mg [aventis]	oad		
7349	actrapid vial injection solution 100 units/ml [novo]	insulin		
7350	insulin isophane porcine vial injection suspension 100 units/ml	insulin		
7375	rosiglitazone with metformin tablets 4mg + 1000mg	oad	1	
7393	insulin glargine cartridge injection solution 100 units/ml	insulin		
7400	insulin glargine disposable pen injection solution 100 units/ml	insulin		
7402	lantus vial injection solution 100 units/ml [aventis]	insulin		
7409	amaryl tablets 3mg [aventis]	oad		
7537	humulin zn injection 100 units/ml [lilly]	insulin		
7610	glucophage tablets 850mg [merck ser]	oad	1	1
7744	daonil tablets 5mg [aventis]	oad		
7757	insulin neulente (zinc susp)(purified) 100 i/u inj	insulin		
7763	insulin neuphane (isophane)(purified) 100 i/u inj	insulin		
7764	insulin neusulin (neutral)(purified) 100 i/u inj	insulin		
7765	insulin neutral (human) 100 i/u inj	insulin		
7771	human protaphane penfill 100 units/ml [novo]	insulin		
7772	human protaphane injection 100 units/ml [novo]	insulin		
7783	insulin isophane (human) 100 i/u inj	insulin		
7793	humaject m3 pen 100 iu/ml [lilly]	insulin		
7815	metformin 800 mg tab	oad	1	1
7861	insulin humulin s (neutral) cartridge 100 i/u	insulin		
7912	semi-daonil tablets 2.5mg [aventis]	oad		
7959	insulin mixtard 30/70 40 i/u inj	insulin		

8034	diabinese tablets 100mg [pfizer]	oad		
8118	humaject i pen 100 iu/ml [lilly]	insulin		
8168	diabinese tablets 250mg [pfizer]	oad		
8203	penmix 50/50 penfill injection 100 iu/ml [novo]	insulin		
8322	insulin zinc suspension mixed human pyr injection 100 units/ml	insulin		
8354	insulin isophane 70%/neutral 30% 100 i/u inj	insulin		
8376	insulin isophane 100 i/u	insulin		
8390	gliquidone tablets 30mg	oad		
8483	MONOJECT INSULIN NEEDLES	insulin		
8646	insulin zinc crystalline susp 100 i/u inj	insulin		
8838	insulin semitard 40 i/u inj	insulin		
8839	insulin semitard 100 i/u inj	insulin		
8841	humulin m5 injection 100 units/ml [lilly]	insulin		
8895	initard 50/50 injection 100 units/ml [novo]	insulin		
8976	euglucon tablets 2.5mg [aventis]	oad		
9079	insulin soluble 100 i/u inj	insulin		
9105	GLUCOBAY tablets 100mg [BAYER]	oad		
9108	tolbutamide 250 mg tab	oad		
9341	insulin biphasic isophane human prb injection 30:70; 100 units/ml	insulin		
9376	insulin zinc suspension crystalline human pyr - long acting injection 100 units/ml	insulin		
9503	hypurin bovine protamine zinc vial injection suspension 100 units/ml [wockhardt]	insulin		
9521	pork actrapid vial injection solution 100 units/ml [novo]	insulin		
9565	humaject s disposable pen injection solution 100 units/ml [lilly]	insulin		
9618	hypurin porcine 30/70 mix injection 100 iu/ml [wockhardt]	insulin		
9662	avandia tablets 4mg [glaxsk pha]	oad		
9699	pioglitazone tablets 30mg	oad		
9707	repaglinide tablets 1mg	oad		
9737	insulatard innolet injection 100 iu/ml [novo]	insulin		
9748	repaglinide tablets 2mg	oad		
9865	repaglinide tablets 500 micrograms	oad		
10001	humalog mix 50 disposable pen injection suspension 100 units/ml [lilly]	insulin		
10051	pioglitazone tablets 45mg	oad		
10067	insulin biphasic aspart human pyr injection 30:70; 100 units/ml	insulin		
10145	Humapen luxura insulin pen 3ml/1-60 units Insulin pen 3ml/1-60 units (Eli Lilly and Company Ltd)	insulin		
10175	insulin isophane human pyr injection 100 iu/ml	insulin		
10184	insulin detemir injection solution 100 iu/ml	insulin		
10207	insulin isophane human cartridge injection suspension 100 units/ml	insulin		
10208	insulatard innolet injection suspension 100 units/ml [novo]	insulin		
10225	lantus opticlik injection solution 100 units/ml [aventis]	insulin		
10229	humulin i disposable pen injection suspension 100 units/ml [lilly]	insulin		

10243	humalog mix 25 cartridge injection suspension 100 units/ml [lilly]	insulin		
10244	mixtard 40 penfill injection suspension 100 units/ml [novo]	insulin		
10245	mixtard 10 penfill injection suspension 100 units/ml [novo]	insulin		
10259	insulin glargine vial injection solution 100 units/ml	insulin		
10264	humalog disposable pen injection solution 100 units/ml [lilly]	insulin		
10277	humulin m3 cartridge injection suspension 100 units/ml [lilly]	insulin		
10427	tolazamide tablets 250mg	oad		
10484	penmix 20/80 penfill penfill [novo]	insulin		
10545	insulin humulin m4 cartridge 100 i/u	insulin		
10546	insulin humulin m4 100 i/u inj	insulin		
10547	humulin lente injection 100 units/ml [lilly]	insulin		
10566	insulin humulin m cartridge 100 i/u	insulin		
10572	insulin soluble bovine injection 100 units/ml	insulin		
10691	insulin isophane (nph) 100 i/u inj	insulin		
10887	penmix 40/60 penfill injection 100 iu/ml [novo]	insulin		
10910	humaject m2 pen 100 iu/ml [lilly]	insulin		
10915	humaject m1 pen 100 iu/ml [lilly]	insulin		
11055	insulin biphasic isophane human pyr injection 20:80; 100 units/ml	insulin		
11056	insulin biphasic isophane human pyr injection 30:70; 100 units/ml	insulin		
11080	insulin isophane human prb injection 100 iu/ml	insulin		
11107	humulin m4 injection 100 units/ml [lilly]	insulin		
11284	amaryl tablets 4mg [aventis]	oad		
11316	novonorm tablets 500 micrograms [novo]	oad		
11321	novonorm tablets 1mg [novo]	oad		
11337	novorapid novolet injection 100 iu/ml [novo]	insulin		
11366	novonorm tablets 2mg [novo]	oad		
11483	nateglinide tablets 60mg	oad		
11601	rosiglitazone with metformin tablets 2mg + 500mg	oad	1	
11604	rosiglitazone with metformin tablets 1mg + 500mg	oad	1	
11609	metformin with rosiglitazone tablets 500mg + 1mg	oad	1	
11610	metformin with rosiglitazone tablets 500mg + 2mg	oad	1	
11695	diamicron mr tablets 30mg [servier]	oad		
11717	rosiglitazone with metformin tablets 2mg + 1000mg	oad	1	
11737	metformin with rosiglitazone tablets 1000mg + 4mg	oad	1	
11760	metformin with rosiglitazone tablets 1000mg + 2mg	oad	1	
11946	tolbutamide injection 50mg/ml	oad		
11990	metformin oral solution 500mg/5ml	oad	1	1
12035	insulin zinc lente bovine vial injection suspension 100 units/ml	insulin		
12060	insulin quicksol (soluble neutral) 100 i/u inj	insulin		
12244	insulin zinc bovine susp 100 i/u inj	insulin		
12245	glutril tablets 25mg [roche]	oad		
12259	glibornuride tablets 25mg	oad		
12297	hypurin bovine neutral injection 100 units/ml [cp pharm]	insulin		

12299	semitard mc injection 100 units/ml [novo]	insulin		
12300	SYRINGE INSULIN (BS1619/1) 2ML	insulin		
12455	rastinon tablets 500mg [hoechstmar]	oad		
12513	glibenese tablets 5mg [pfizer]	oad		
12638	insulin soluble human pyr injection 100 units/ml	insulin		
12654	insulin soluble human prb injection 100 units/ml	insulin		
12818	mixtard 50 injection 50:50; 100 units/ml [novo]	insulin		
13277	mixtard 50 penfill injection suspension 100 units/ml [novo]	insulin		
13331	euglucon tablets 5mg [aventis]	oad		
13416	insulin biphasic injection 100 units/ml	insulin		
13516	hypurin bovine isophane injection 100 units/ml [cp pharm]	insulin		
13550	insulin bp 100 i/u	insulin		
13622	hypurin porcine neutral injection 100 units/ml [cp pharm]	insulin		
13628	romozin tablets 400mg [glaxo]	oad		
13729	insulin isophane human emp injection 100 units/ml	insulin		
13819	hypurin porcine isophane injection 100 units/ml [wockhardt]	insulin		
13837	insulin biphasic isophane human prb injection 10:90; 100 units/ml	insulin		
14164	avandamet tablets 2mg + 1000mg [glaxsk pha]	oad	1	
14270	humalog mix 25 disposable pen injection suspension 100 units/ml [lilly]	insulin		
14290	insulatard penfill injection suspension 100 units/ml [novo]	insulin		
14299	insulin glulisine cartridge injection solution 100 units/ml	insulin		
14301	insulin detemir cartridge injection solution 100 units/ml	insulin		
14313	insulin lispro cartridge injection solution 100 units/ml	insulin		
14330	insulin detemir disposable pen injection solution 100 units/ml	insulin		
14339	hypurin bovine neutral vial injection solution 100 units/ml [wockhardt]	insulin		
14340	hypurin bovine isophane vial injection suspension 100 units/ml [wockhardt]	insulin		
14345	apidra cartridge injection solution 100 units/ml [sanofi/ave]	insulin		
14357	humulin i cartridge injection suspension 100 units/ml [lilly]	insulin		
14362	insulin lispro disposable pen injection solution 100 units/ml	insulin		
14504	insulin hypurin protamine zinc 100 i/u inj	insulin		
14505	insulin protamine zinc bovine vial injection suspension 100 units/ml	insulin		
14506	insulin bovine protamine zinc 100 i/u inj	insulin		
14619	insulin biphasic isophane porcine injection 30:70; 100 units/ml	insulin		
14644	insulin biphasic isophane human prb injection 20:80; 100 units/ml	insulin		
14649	insulin biphasic isophane human pyr injection 10:90; 100 units/ml	insulin		
14918	humulin i vial injection suspension 100 units/ml [lilly]	insulin		
14925	insulin isophane human vial injection suspension 100 units/ml	insulin		
14928	insulatard vial injection suspension 100 units/ml [novo]	insulin		
14930	hypurin porcine neutral cartridge injection solution 100 units/ml [wockhardt]	insulin		

14933	hypurin porcine isophane cartridge injection suspension 100 units/ml [wockhardt]	insulin		
14938	insulin soluble bovine cartridge injection solution 100 units/ml	insulin		
14944	humulin s cartridge injection solution 100 units/ml [lilly]	insulin		
15040	insulin monophane (isophane) 100 i/u inj	insulin		
15199	insuman comb 25 injection 100 iu/ml [aventis]	insulin		
15232	avandia tablets 8mg [glaxsk pha]	oad		
15374	gliclazide oral suspension 40mg/5ml	oad		
15484	insulin isophane bovine injection 100 units/ml	insulin		
15624	insulin isophane (highly purified) 100 i/u inj	insulin		
15710	insulin soluble human emp injection 100 units/ml	insulin		
15955	starlix tablets 120mg [novartis]	oad		
15961	insulin isophane human crb injection 100 iu/ml	insulin		
16044	glucophage sr tablets 500mg [merck ser]	oad		
16129	insulin soluble human cartridge injection solution 100 units/ml	insulin		
16142	insulin aspart cartridge injection solution 100 units/ml	insulin		
16152	insulin biphasic isophane human cartridge injection suspension 30:70; 100 units/ml	insulin		
16160	humulin m3 disposable pen injection suspension 100 units/ml [lilly]	insulin		
16209	insulin hypurin soluble 100 i/u inj	insulin		
16211	tolbutamide 100 mg tab	oad		
16213	metformin 250 mg tab	oad	1	1
16602	calabren tablets 2.5mg [berk]	oad		
16682	tempulin injection 100 units/ml [knoll]	insulin		
16700	insulin zinc mixed bovine vial injection suspension 100 units/ml	insulin		
17336	novopen injection device 100 units/ml [novo]	insulin		
17343	gliclazide tablets 80mg [hillcross]	oad		
17580	avandamet tablets 1mg + 500mg [glaxsk pha]	oad	1	
17698	minodiab tablets 5mg [pharmacia]	oad		
17706	minodiab tablets 2.5mg [pharmacia]	oad		
17712	hypurin bovine lente vial injection suspension 100 units/ml [wockhardt]	insulin		
17731	penmix 50/50 injection 100 iu/ml [novo]	insulin		
17809	humaject m4 pen 100 iu/ml [lilly]	insulin		
18220	pioglitazone with metformin tablets 15mg + 850mg	oad	1	
18224	humalog vial injection solution 100 units/ml [lilly]	insulin		
18301	insulin soluble inj i/u^2	insulin		
18461	insulin zinc suspension mixed human prb injection 100 units/ml	insulin		
18590	insulin isophane bovine vial injection suspension 100 units/ml	insulin		
18592	insulin soluble bovine vial injection solution 100 units/ml	insulin		
18593	humalog mix 50 cartridge injection suspension 100 units/ml [lilly]	insulin		
18645	insulin neutral (purified) 100 i/u inj	insulin		
18931	insulin zinc suspension crystalline human prb - intermediate acting injection 100 units/ml	insulin		

19029	SYRINGE PRE-SET INSULIN FOR BLIND 2ML	insulin		
19336	tolazamide tablets 100mg	oad		
19472	actos tablets 45mg [takeda]	oad		
19491	apidra vial injection solution 100 units/ml [sanofi/ave]	insulin		
19513	humulin m3 vial injection suspension 100 units/ml [lilly]	insulin		
19658	glurenorm tablets 30mg [sanofi s]	oad		
19707	insulin humulin s (neutral soluble)	insulin		
19829	insulin novo monotard mc	insulin		
19877	insulin aspart disposable pen injection solution 100 units/ml	insulin		
19878	insulin biphasic isophane human disposable pen injection suspension 30:70; 100 units/ml	insulin		
20195	insulin bovine protamine zinc 40 i/u inj	insulin		
20196	insulin soluble 40 i/u inj	insulin		
20287	actos tablets 15mg [takeda]	oad		
20422	insuman comb 15 injection 100 iu/ml [aventis]	insulin		
20671	insulin hum/actraphane	insulin		
20672	insulin hum/actrapid	insulin		
20810	metformin	oad	1	1
20889	actos tablets 30mg [takeda]	oad		
20995	hypurin porcine 30/70 mix cartridge injection suspension 100 units/ml [wockhardt]	insulin		
21110	insulin biphasic isophane human prb injection 50:50; 100 units/ml	insulin		
21232	insulin biphasic isophane human vial injection suspension 30:70; 100 units/ml	insulin		
21235	humulin s vial injection solution 100 units/ml [lilly]	insulin		
21347	penmix 40/60 injection 100 iu/ml [novo]	insulin		
21374	insulin biphasic isophane human prb injection 40:60; 100 units/ml	insulin		
21395	insulin biphasic isophane human pyr injection 40:60; 100 units/ml	insulin		
21422	insulin biphasic isophane human cartridge injection suspension 40:60; 100 units/ml	insulin		
21424	glibenclamide oral suspension 5mg/5ml	oad		
21459	SYRINGE INSULIN U100 S/U+8MM NEEDLE	insulin		
21489	tolanase tablets 250mg [pharmacia]	oad		
21554	insuman comb 50 injection 100 iu/ml [aventis]	insulin		
21564	gliclazide tablets 80mg [wockhardt]	oad		
21583	apidra optiset injection solution 100 units/ml [sanofi/ave]	insulin		
21590	insulin glulisine disposable pen injection solution 100 units/ml	insulin		
21832	diabetamide tablets 5mg [ashbourne]	oad		
21870	RASTINON	oad		
21892	diaglyk tablets 80mg [ashbourne]	oad		
21945	insulin pork insulatard	insulin		
22058	pur-in mix 15/85 injection [cp pharm]	insulin		
22094	insulin humulin m2 vial	insulin		
22145	tolanase tablets 100mg [pharmacia]	oad		

22155	humaject m5 pen 100 iu/ml [lilly]	insulin		
22161	insulin humulin m1 vial	insulin		
22496	insulin zinc lente purified suspension	insulin		
22614	DAONIL 10 MG TAB	oad		
22636	tolbutamide 1 gm tab	oad		
22697	insulin biphasic isophane human pyr injection 50:50; 100 units/ml	insulin		
22806	insulin pork actrapid	insulin		
22823	insulin isophane (purified) 100 i/u inj	insulin		
22858	acetohexamide tablets 500mg	oad		
22945	insuman rapid injection 100 iu/ml [aventis]	insulin		
22983	insuman rapid cartridge injection solution 100 units/ml [aventis]	insulin		
23003	insulin isophane (nph) 40 i/u	insulin		
23099	insulin biphasic aspart disposable pen injection suspension 30:70; 100 units/ml	insulin		
23231	hypurin bovine neutral cartridge injection solution 100 units/ml [wockhardt]	insulin		
23945	starlix tablets 60mg [novartis]	oad		
23992	insuman basal optiset injection suspension 100 units/ml [aventis]	insulin		
23993	insuman rapid optiset injection solution 100 units/ml [aventis]	insulin		
24002	insuman comb 25 vial injection suspension 100 units/ml [aventis]	insulin		
24485	insulin zinc animal suspension	insulin		
24593	neutral insulin bovine injection 100 iu/ml	insulin		
24722	insulin isophane 50%/neutral 50% 100 i/u inj	insulin		
24795	insulin biphasic aspart cartridge injection suspension 30:70; 100 units/ml	insulin		
24800	hypurin porcine 30/70 mix vial injection suspension 100 units/ml [wockhardt]	insulin		
24845	insulin pur-in isophane 100 i/u inj	insulin		
24846	pur-in neutral injection 100 units/ml [cp pharm]	insulin		
24848	glymidine sodium tablets 500mg	oad		
24866	insulin insulatard (leo retard) 40 i/u inj	insulin		
24993	insuman comb 25 cartridge injection suspension 100 units/ml [aventis]	insulin		
25006	insulin human actrapid (neutral)	insulin		
25133	insuman comb 25 optiset injection suspension 100 units/ml [aventis]	insulin		
25479	insulin soluble porcine cartridge injection solution 100 units/ml	insulin		
25636	libanil tablets 2.5mg [aps]	oad		
25678	glucamet tablets 500mg [opus]	oad	1	1
25735	insulin biphasic isophane human cartridge injection suspension 20:80; 100 units/ml	insulin		
25736	insulin biphasic isophane human cartridge injection suspension 10:90; 100 units/ml	insulin		
25812	insulin isophane human disposable pen injection suspension 100 units/ml	insulin		
26060	insulin lispro vial injection solution 100 units/ml	insulin		

26098	hypurin porcine neutral vial injection solution 100 units/ml [wockhardt]	insulin		
26118	dimelor tablets 500mg [lilly]	oad		
26218	calabren tablets 5mg [berk]	oad		
26258	glucamet tablets 850mg [opus]	oad	1	1
26403	pur-in mix 25/75 injection [cp pharm]	insulin		
26498	insulin zinc suspension mixed bovine and porcine injection 100 units/ml	insulin		
26621	insulin soluble human crb injection 100 iu/ml	insulin		
26784	insulin zinc semilente susp bp 100 i/u inj	insulin		
26795	SYRINGE INSULIN DISPOSABLE	insulin		
27125	starlix tablets 180mg [novartis]	oad		
27151	SYRINGE INSULIN U100 S/U+8MM NEEDLE	insulin		
27177	insulin biphasic lispro human prb injection 50:50; 100 units/ml	insulin		
27280	insulin biphasic isophane porcine vial injection suspension 30:70; 100 units/ml	insulin		
27396	insulin soluble porcine vial injection solution 100 units/ml	insulin		
27402	insulin soluble human vial injection solution 100 units/ml	insulin		
27461	insuman basal cartridge injection suspension 100 units/ml [aventis]	insulin		
27501	orabet tablets 500mg [lagap]	oad		
27614	penmix 30/70 injection 100 iu/ml [novo]	insulin		
27911	insulin human actrapid penfill	insulin		
27969	glymese tablets 250mg [ddsa]	oad		
28096	insulin biphasic isophane human cartridge injection suspension 50:50; 100 units/ml	insulin		
28101	insulin glulisine vial injection solution 100 units/ml	insulin		
28183	hypurin porcine isophane vial injection suspension 100 units/ml [wockhardt]	insulin		
28185	insulin biphasic lispro cartridge injection suspension 25:75; 100 units/ml	insulin		
28442	insulin glulisine injection solution 100 units/ml	insulin		
28588	hypurin bovine isophane cartridge injection suspension 100 units/ml [wockhardt]	insulin		
28708	malix tablets 2.5mg [lagap]	oad		
28723	insulin zinc bovine suspension	insulin		
28978	insulin pur-in mix 15/85 100 i/u inj	insulin		
29326	glipizide tablets 5mg [gen (uk)]	oad		
29567	insulin aspart vial injection solution 100 units/ml	insulin		
29837	insulin biphasic isophane human prb injection 25:75; 100 units/ml	insulin		
29939	gliclazide tablets 80mg [gen (uk)]	oad		
29953	apidra opticlik injection solution 100 units/ml [sanofi/ave]	insulin		
30209	actrapid mc injection 100 units/ml [arun]	insulin		
30236	isophane insulin injection 100 iu/ml	insulin		
30316	metformin with pioglitazone tablets 850mg + 15mg	oad	1	
30460	malix tablets 5mg [lagap]	oad		

30686	insulin isophane porcine cartridge injection suspension 100 units/ml	insulin		
30819	insuman comb 15 optiset injection suspension 100 units/ml [aventis]	insulin		
30861	insulin zinc human suspension	insulin		
31077	competact film coated tablets [takeda]	oad	1	
31146	metsol oral solution 500mg/5ml [orbis]	oad	1	1
31205	insuman comb 50 optiset injection suspension 100 units/ml [aventis]	insulin		
31212	gliclazide tablets 80mg [actavis]	oad		
31258	insulin biphasic lispro disposable pen injection suspension 25:75; 100 units/ml	insulin		
31267	insulin pur-in mix 50/50 100 i/u inj	insulin		
31464	Exubera kit (Pfizer Ltd)	insulin		
31465	exubera powder for inhalation 1mg [pfizer]	insulin		
31467	exubera powder for inhalation 3mg [pfizer]	insulin		
31474	libanil tablets 5mg [aps]	oad		
32053	insulin humalog mix 25	insulin		
33087	metformin tablets 500mg [actavis]	oad	1	1
33167	insulin biphasic isophane human crb injection 25:75; 100 units/ml	insulin		
33232	insulin biphasic isophane human crb injection 50:50; 100 units/ml	insulin		
33562	duclazide tablets 80mg [dumex]	oad		
33673	tolbutamide tablets 500mg [actavis]	oad		
33674	metformin tablets 850mg [hillcross]	oad	1	1
33966	insulatard injection 100 units/ml [novo]	insulin		
34004	metformin tablets 500mg [ivax]	oad	1	1
34020	metformin tablets 850mg [ivax]	oad	1	1
34031	monotard mc injection 100 units/ml [novo]	insulin		
34097	human initard 50/50 injection 100 units/ml [novo]	insulin		
34135	metformin tablets 500mg [m&a pharm]	oad	1	1
34323	metformin tablets 500mg [hillcross]	oad	1	1
34399	gliclazide tablets 80mg [ivax]	oad		
34504	metformin tablets 500mg [wockhardt]	oad	1	1
34507	glibenclamide tablets 2.5mg [wockhardt]	oad		
34563	glibenclamide tablets 5mg [wockhardt]	oad		
34598	metformin tablets 500mg [gen (uk)]	oad	1	1
34676	glibenclamide tablets 2.5mg [hillcross]	oad		
34697	metformin tablets 850mg [wockhardt]	oad	1	1
34706	glibenclamide tablets 2.5mg [ivax]	oad		
34742	metformin tablets 850mg [teva]	oad	1	1
34802	glipizide tablets 5mg [ivax]	oad		
34836	metformin tablets 850mg [actavis]	oad	1	1
34917	metformin tablets 500mg [teva]	oad	1	1
34932	gliclazide tablets 80mg [genus]	oad		

34957	tolbutamide tablets 500mg [hillcross]	oad		
35022	sitagliptin tablets 100mg	oad		
35144	byetta injection 5 micrograms [lilly]	oad		
35149	exenatide injection 10micrograms	oad		
35150	byetta injection 10micrograms [lilly]	oad		
35251	exenatide injection 5 micrograms	oad		
35253	insuman comb 50 cartridge injection suspension 100 units/ml [aventis]	insulin		
35260	levemir innolet injection solution 100 units/ml [novo]	insulin		
35462	januvia tablets 100mg [m s d]	oad		
35468	insuman basal vial injection suspension 100 units/ml [aventis]	insulin		
35561	prandin tablets 2mg [novo]	oad		
35701	insulin biphasic lispro disposable pen injection suspension 50:50; 100 units/ml	insulin		
36031	insulin biphasic isophane porcine cartridge injection suspension 30:70; 100 units/ml	insulin		
36043	Exubera chamber (Pfizer Ltd)	insulin		
36066	insulin isophane bovine cartridge injection suspension 100 units/ml	insulin		
36146	insulin biphasic lispro cartridge injection suspension 50:50; 100 units/ml	insulin		
36194	insulin biphasic isophane human cartridge injection suspension 25:75; 100 units/ml	insulin		
36355	insulin human powder for inhalation 1mg	insulin		
36356	insulin human powder for inhalation 3mg	insulin		
36430	insulin soluble human disposable pen injection solution 100 units/ml	insulin		
36513	velosulin cartridge injection 100 units/ml [novo]	insulin		
36774	prandin tablets 1mg [novo]	oad		
36853	lantus solostar injection solution 100 units/ml [sanofi/ave]	insulin		
36856	gliclazide tablets 80mg [sandoz]	oad		
36920	apidra solostar injection solution 100 units/ml [sanofi/ave]	insulin		
36948	prandin tablets 500 micrograms [novo]	oad		
37617	rosiglitazone tablets 2mg	oad		
37874	vildagliptin with metformin tablets 50mg + 850mg	oad	1	
37875	vildagliptin tablets 50mg	oad		
37902	vildagliptin with metformin tablets 50mg + 1000mg	oad	1	
38355	metformin modified release tablet 750mg	oad	1	1
38400	glucophage sr tablets 750mg [merck ser]	oad	1	1
38422	isophane injection 100 iu/ml [celltech]	insulin		
38551	eucreas tablets 50mg + 1000mg [novartis]	oad	1	
38986	humalog kwikpen injection solution 100 units/ml [lilly]	insulin		
39006	humalog mix 25 kwikpen injection suspension 100 units/ml [lilly]	insulin		
39086	humalog mix 50 kwikpen injection suspension 100 units/ml [lilly]	insulin		
39149	galvus tablets 50mg [novartis]	oad		
39203	eucreas tablets 50mg + 850mg [novartis]	oad	1	

39560	bolamyn sr tablets 500mg [teva]	oad	1	1
39598	metformin modified release tablet 1000mg	oad	1	1
39729	glucophage sr tablets 1000mg [merck ser]	oad	1	1
39988	metformin oral powder 500mg	oad	1	1
40007	glucophage sachets 1000mg [merck ser]	oad	1	1
40110	glucophage sachets 500mg [merck ser]	oad	1	1
40233	metformin oral powder 1000mg	oad	1	1
40365	glimepiride tablets 1mg [actavis]	oad		
40425	nazdol mr tablets 30mg [teva]	oad		
40642	victoza injection 18mg/3ml [novo]	oad		
40693	liraglutide injection 18mg/3ml	oad		
41120	insulin biphasic isophane human disposable pen injection suspension 50:50; 100 units/ml	insulin		
41204	saxagliptin tablets 5mg	oad		
41431	onglyza tablets 5mg [bms]	oad		
41558	glibenclamide tablets 5mg [teva]	oad		
41559	glibenclamide tablets 5mg [hillcross]	oad		
41593	glibenclamide tablets 2.5mg [teva]	oad		
41834	insulin zinc suspension lente injection 100 iu/ml [celltech]	insulin		
41898	glibenclamide	oad		
41959	penject injection device 100 units/ml [hypoguard]	insulin		
42161	orabet tablets 500mg [sandoz]	oad		
42395	humalog mix 25 vial injection suspension 100 units/ml [lilly]	insulin		
42790	gliclazide tablets 80mg [merck-gen]	oad		
42954	insulin biphasic isophane human vial injection suspension 25:75; 100 units/ml	insulin		
43065	gliclazide tablets 40mg	oad		
43270	metformin sugar free oral solution 500mg/5ml [rosemont]	oad	1	1
43465	zicron tablets 40mg [bristol lb]	oad		
43619	sitagliptin with metformin tablets 50mg + 1000mg	oad	1	1
43684	janumet tablets 50mg + 1000mg [m s d]	oad		
43950	humulin i kwikpen injection suspension 100 units/ml [lilly]	insulin		
43953	insulin biphasic lispro vial injection suspension 25:75; 100 units/ml	insulin		
43991	humulin m3 kwikpen injection suspension 100 units/ml [lilly]	insulin		
44250	metformin oral solution 500mg/5ml [hillcross]	oad	1	1
44251	insulin zinc suspension mixed porcine injection 100 units/ml	insulin		
44304	glyconon tablets 500mg [ddsa]	oad		
44378	insulin biphasic isophane human disposable pen injection suspension 25:75; 100 units/ml	insulin		
44473	edicil mr tablets 30mg [ratiopharm]	oad		
44480	insuman comb 25 solostar injection suspension 100 units/ml [aventis]	insulin		
44738	niddaryl tablets 1mg [dee]	oad		
45158	insuman comb 15 cartridge injection suspension 100 units/ml [aventis]	insulin		

45215	gliclazide tablets 80mg [neolab]	oad		
45581	metabet sr tablets 500mg [morningsid]	oad	1	1
45775	saxagliptin tablets 2.5mg	oad		
45821	onglyza tablets 2.5mg [bms]	oad		
45831	dacadis modified release tablet 30mg [gen (uk)]	oad		
46001	insuman basal solostar injection suspension 100 units/ml [aventis]	insulin		
46458	exenatide powder for prolonged release injection suspension 2mg	oad		
46469	bydureon powder for prolonged release injection suspension 2mg [lilly]	oad		
46665	linagliptin tablets 5mg	oad		
46666	novorapid flextouch injection solution 100 units/ml [novo]	insulin		
46716	trajenta tablets 5mg [boeh ingl]	oad		
46927	tolbutamide tablets 500mg [teva]	oad		
46989	metabet sr tablets 1000mg [morningsid]	oad	1	1
47074	gliclazide oral suspension 80mg/5ml	oad		
47360	neutral insulin injection 100 units/ml [celltech]	insulin		
47856	Neuphane 100unit/ml Injection (Wellcome Medical Division)	insulin		
47894	Nazdol MR 30mg tablets (Consilient Health Ltd)	oad		
47939	Glucient SR 500mg tablets (Consilient Health Ltd)	oad	1	1
48056	Gliclazide 80mg tablets (Sovereign Medical Ltd)	oad		
48120	Avandia 2mg Tablet (GlaxoSmithKline UK Ltd)	oad		
48139	Pioglitazone 30mg tablets (A A H Pharmaceuticals Ltd)	oad		
48149	Metformin 500mg tablets (Almus Pharmaceuticals Ltd)	oad	1	1
48401	Sitagliptin 50mg tablets	oad		
48533	Sitagliptin 25mg tablets	oad		
49108	NovoRapid Penfill 100units/ml solution for injection 3ml cartridges (Necessity Supplies Ltd)	insulin		
49502	Glucophage SR 500mg tablets (Mawdsley-Brooks & Company Ltd)	oad	1	1
49738	Metformin 1g modified-release tablets (A A H Pharmaceuticals Ltd)	oad	1	1
49831	Lantus 100units/ml solution for injection 3ml pre-filled SoloStar pen (Necessity Supplies Ltd)	insulin		
50087	Januvia 50mg tablets (Merck Sharp & Dohme Ltd)	oad		
50124	Januvia 25mg tablets (Merck Sharp & Dohme Ltd)	oad		
50570	Glucophage SR 500mg tablets (Lexon (UK) Ltd)	oad	1	1
50633	Lantus 100units/ml solution for injection 3ml cartridges (Necessity Supplies Ltd)	insulin		
50682	Jentadueto 2.5mg/1000mg tablets (Boehringer Ingelheim Ltd)	oad		
50821	Metformin 850mg tablets (Pfizer Ltd)	oad	1	1
50970	Metformin 500mg tablets (Bristol Laboratories Ltd)	oad	1	1
51080	Metabet SR 1000mg tablets (Actavis UK Ltd)	oad	1	1
51135	Metformin 500mg modified-release tablets (A A H Pharmaceuticals Ltd)	oad	1	1
51527	Metformin 500mg tablets (Boston Healthcare Ltd)	oad	1	1
51743	NovoRapid Penfill 100units/ml solution for injection 3ml cartridges (Sigma Pharmaceuticals Plc)	insulin		

51955	Gliclazide 80mg tablets (Accord Healthcare Ltd)	oad		
52203	Enyglid 0.5mg tablets (Consilient Health Ltd)	oad		
52221	Diagemet XL 500mg tablets (Genus Pharmaceuticals Ltd)	oad		
52442	Metformin 500mg tablets (Pfizer Ltd)	oad	1	1
52445	Linagliptin 2.5mg / Metformin 1g tablets	oad		
52449	Linagliptin 2.5mg / Metformin 850mg tablets	oad		
52522	Humalog Mix50 KwikPen 100units/ml suspension for injection 3ml pre-filled pen (DE Pharmaceuticals)	insulin		
52634	Glucophage SR 500mg tablets (DE Pharmaceuticals)	oad	1	1
52748	Insulatard Penfill 100units/ml suspension for injection 3ml cartridges (Waymade Healthcare Plc)	insulin		
53118	NovoRapid FlexPen 100units/ml solution for injection 3ml pre-filled pen (Mawdsley-Brooks & Company Ltd)	insulin		
53251	NovoRapid Penfill 100units/ml solution for injection 3ml cartridges (DE Pharmaceuticals)	insulin		
53288	Gliclazide 30mg modified-release tablets (A A H Pharmaceuticals Ltd)	oad		
53478	Metformin 500mg modified-release tablets (Kent Pharmaceuticals Ltd)	oad	1	1
53710	Insulin human 500units/ml solution for injection 20ml vials	insulin		
53774	Metabet SR 500mg tablets (Actavis UK Ltd)	oad	1	1
53867	Metformin 500mg tablets (Zentiva)	oad	1	1
54150	Jentadueto 2.5mg/850mg tablets (Boehringer Ingelheim Ltd)	oad		
54182	Dapagliflozin 10mg tablets	oad		
54203	Forxiga 10mg tablets (AstraZeneca UK Ltd)	oad		
54265	Dapagliflozin 5mg tablets	oad		
54442	Metformin (roi) 1000mg Tablet	oad	1	1
54462	Insulin biphasic isophane human emp 25:75; 100 units/ml Injection	insulin		
54480	Forxiga 5mg tablets (AstraZeneca UK Ltd)	oad		
54764	Gliclazide 80mg tablets (Arrow Generics Ltd)	oad		
54891	Saxagliptin 2.5mg / Metformin 1g tablets	oad	1	
54898	Metformin 850mg tablets (Almus Pharmaceuticals Ltd)	oad	1	1
54973	Saxagliptin 2.5mg / Metformin 850mg tablets	oad		
55234	Tresiba FlexTouch 200units/ml solution for injection 3ml pre-filled pen (Novo Nordisk Ltd)	insulin		
55270	Duformin 500mg Tablet (Dumex Ltd)	oad		
55413	Lixisenatide 20micrograms/0.2ml solution for injection 3ml pre-filled disposable devices	oad		
55459	Lixisenatide 10micrograms/0.2ml solution for injection 3ml pre-filled disposable devices	oad		
55462	Tresiba FlexTouch 100units/ml solution for injection 3ml pre-filled pen (Novo Nordisk Ltd)	insulin		
55517	Insulin isophane human 100units/ml suspension for injection 10ml vials	insulin		
55603	Humalog KwikPen 100units/ml solution for injection 3ml pre-filled pen (DE Pharmaceuticals)	insulin		
55618	Levemir FlexPen 100units/ml solution for injection 3ml pre-filled pen (Waymade Healthcare Plc)	insulin		

55687	Insulin degludec 100units/ml solution for injection 3ml pre-filled disposable devices	insulin		
55711	Metformin 500mg tablets (Alliance Healthcare (Distribution) Ltd)	oad	1	1
55723	Lixisenatide 10micrograms/0.2ml solution for injection 3ml pre-filled disposable devices and Lixisenatide 20micrograms/0.2ml solution for injection 3ml pre-filled disposable devices	oad		
55728	Lyxumia 10micrograms/0.2ml solution for injection 3ml pre-filled pen (Sanofi)	oad		
55729	Lyxumia 20micrograms/0.2ml solution for injection 3ml pre-filled pen (Sanofi)	oad		
55739	Metformin 500mg tablets (Tillomed Laboratories Ltd)	oad	1	1
55767	Lyxumia 10micrograms/0.2ml solution for injection 3ml pre-filled pen and Lyxumia 20micrograms/0.2ml solution for injection 3ml pre-filled pen (Sanofi)	oad		
55862	Gliclazide Oral solution	oad		
55907	Insulin degludec 100units/ml solution for injection 3ml cartridges	insulin		
55910	Tresiba Penfill 100units/ml solution for injection 3ml cartridges (Novo Nordisk Ltd)	insulin		
56008	Gliclazide 80mg tablets (Almus Pharmaceuticals Ltd)	oad		
56208	Pioglitazone 15mg tablets (A A H Pharmaceuticals Ltd)	oad		
56376	Rosiglitazone 4mg with glimepiride 4mg tablet	oad		
56437	Gliclazide 60mg modified-release tablets	oad		
56489	NovoMix 30 Penfill 100units/ml suspension for injection 3ml cartridges (Waymade Healthcare Plc)	insulin		
56495	Lantus 100units/ml solution for injection 3ml pre-filled OptiSet pen (Waymade Healthcare Plc)	insulin		
56691	Insulin degludec 200units/ml solution for injection 3ml pre-filled disposable devices	insulin		
56831	Troglitazone 200mg Tablet	oad		
56857	Insulin isophane biphasic human 15/85 100units/ml suspension for injection 3ml cartridges	insulin		
56965	Komboglyze 2.5mg/1000mg tablets (AstraZeneca UK Ltd)	oad		
57147	Bolamyn SR 1000mg tablets (Teva UK Ltd)	oad	1	1
57457	Metformin 500mg tablets (Aurobindo Pharma Ltd)	oad	1	1
57529	Humalog 100units/ml solution for injection 10ml vials (Dowelhurst Ltd)	insulin		
57564	Humalog KwikPen 100units/ml solution for injection 3ml pre-filled pen (Waymade Healthcare Plc)	insulin		
57601	Daonil 5mg tablets (Dowelhurst Ltd)	oad		
57622	Humalog Mix50 KwikPen 100units/ml suspension for injection 3ml pre-filled pen (Waymade Healthcare Plc)	insulin		
57659	Pioglitazone 30mg tablets (Actavis UK Ltd)	oad		
57830	Gliclazide 30mg modified-release tablets (Alliance Healthcare (Distribution) Ltd)	oad		
58051	Metformin 500mg/5ml oral solution	oad	1	1
58607	Metformin 500mg/5ml oral solution sugar free (Zentiva)	oad	1	1
58865	Komboglyze 2.5mg/850mg tablets (AstraZeneca UK Ltd)	oad		
58882	Gliclazide 120mg/5ml oral suspension	oad		
59177	Alogliptin 25mg tablets	oad		

59385	Vipdomet 12.5mg/1000mg tablets (Takeda UK Ltd)	oad		
59500	Insulin isophane human 100units/ml suspension for injection 5ml vials	insulin		
59533	NovoRapid FlexPen 100units/ml solution for injection 3ml pre-filled pen (Sigma Pharmaceuticals Plc)	insulin		
59620	Glucophage SR 500mg tablets (Waymade Healthcare Plc)	oad	1	1
59809	Alogliptin 6.25mg tablets	oad		
60012	Dapagliflozin 5mg / Metformin 1g tablets	oad	1	
60073	Canagliflozin 100mg tablets	oad		
60074	Metformin 1g modified-release tablets (Waymade Healthcare Plc)	oad	1	1
60211	Canagliflozin 100mg tablets	oad		
60286	Metformin 500mg/5ml oral suspension	oad	1	1
60328	Alogliptin 12.5mg tablets	oad		
60379	Invokana 300mg tablets (Janssen-Cilag Ltd)	oad		
60386	Canagliflozin 300mg tablets	oad		
60430	Invokana 100mg tablets (Janssen-Cilag Ltd)	oad		
60495	Gliclazide 80mg tablets (Teva UK Ltd)	oad		
60497	Alogliptin 12.5mg / Metformin 1g tablets	oad		
60643	Xigduo 5mg/1000mg tablets (AstraZeneca UK Ltd)	oad		
60681	Vipidia 12.5mg tablets (Takeda UK Ltd)	oad		
60682	Vipidia 25mg tablets (Takeda UK Ltd)	oad		
60933	Humulin M3 100units/ml suspension for injection 10ml vials (Sigma Pharmaceuticals Plc)	insulin		
60951	Insulin human 100units/ml solution for injection 10ml vials	insulin		
60968	Metformin 500mg modified-release tablets (Actavis UK Ltd)	oad	1	1
61043	Sukkarto SR 1000mg tablets (Morningside Healthcare Ltd)	oad	1	1
61311	Glimepiride 4mg tablets (Sigma Pharmaceuticals Plc)	oad		
61559	Sukkarto SR 500mg tablets (Morningside Healthcare Ltd)	oad	1	1
61756	Empagliflozin 10mg tablets	oad		
61845	NovoRapid PumpCart 100units/ml solution for injection 1.6ml cartridges (Novo Nordisk Ltd)	insulin		
61925	NovoNorm 500microgram tablets (Waymade Healthcare Plc)	oad		
61957	Gliclazide 40mg tablets (A A H Pharmaceuticals Ltd)	oad		
62014	Glimepiride 2mg tablets (Accord Healthcare Ltd)	oad		
62034	Laaglyda MR 60mg tablets (Consilient Health Ltd)	oad		
62144	Metformin 500mg modified-release tablets (DE Pharmaceuticals)	oad	1	1
62172	Empagliflozin 25mg tablets	oad		
62180	Insulin aspart 100units/ml solution for injection 1.6ml cartridges	insulin		
62265	Metformin 500mg modified-release tablets (Mawdsley-Brooks & Company Ltd)	oad	1	1
62276	Humulin R 500units/ml solution for injection 20ml vials (Imported (United States))	insulin		
62326	Vipidia 6.25mg tablets (Takeda UK Ltd)	oad		
62426	Pioglitazone 30mg tablets (Accord Healthcare Ltd)	oad		
62605	Metformin 850mg tablets (Kent Pharmaceuticals Ltd)	oad	1	1

62661	Bydureon 2mg powder and solvent for suspension for injection pre-filled pen (AstraZeneca UK Ltd)	oad		
62760	Jardiance 10mg tablets (Boehringer Ingelheim Ltd)	oad		
62824	Metformin 1g modified-release tablets (Actavis UK Ltd)	oad	1	1
62899	Xultophy 100units/ml / 3.6mg/ml solution for injection 3ml pre-filled pen (Novo Nordisk Ltd)	insulin		
62904	Exenatide 2mg powder and solvent for suspension for injection pre-filled disposable devices	oad		
63031	Dapagliflozin 5mg / Metformin 850mg tablets	oad		
63045	Metformin 850mg tablets (Relonchem Ltd)	oad	1	1
63046	Pioglitazone 45mg tablets (A A H Pharmaceuticals Ltd)	oad		
63048	Gliclazide 80mg tablets (Alliance Healthcare (Distribution) Ltd)	oad		
63107	Pioglitazone 45mg tablets (Waymade Healthcare Plc)	oad		
63131	Ziclaseg 30mg modified-release tablets (Lupin (Europe) Ltd)	insulin		
63307	Metformin 1g/5ml oral solution	oad	1	1
63336	Trulicity 1.5mg/0.5ml solution for injection pre-filled pen (Eli Lilly and Company Ltd)	oad		
63401	Trulicity 0.75mg/0.5ml solution for injection pre-filled pen (Eli Lilly and Company Ltd)	oad		
63421	Pioglitazone 30mg tablets (Teva UK Ltd)	oad		
63464	Humalog KwikPen 200units/ml solution for injection 3ml pre-filled pen (Eli Lilly and Company Ltd)	insulin		
63516	Forxiga 10mg tablets (Waymade Healthcare Plc)	oad		
63562	Insulin degludec 100units/ml / Liraglutide 3.6mg/ml solution for injection 3ml pre-filled disposable devices	insulin		
63679	Hypurin soluble 100iu/ml Injection (C P Pharmaceuticals Ltd)	insulin		
63785	Dulaglutide 0.75mg/0.5ml solution for injection pre-filled disposable devices	oad		
63823	Dulaglutide 1.5mg/0.5ml solution for injection pre-filled disposable devices	oad		

Human immunodeficiency virus infection

Medcode	Readterm
2835	HIV positive
8281	HIV disease resulting in wasting syndrome
9130	Human immunodeficiency virus infection
23763	AIDS carrier
23770	Acquired immune deficiency syndrome
23951	HIV disease resulting in candidiasis
24872	[V]Asymptomatic human immunodeficency virus infection status
27641	HIV disease resulting in Pneumocystis carinii pneumonia
27853	HIV disease resulting in Kaposi's sarcoma
33943	Notification of AIDS
36294	Acquired human immunodeficiency virus infection syndrome NOS
37006	HIV disease resulting in mycobacterial infection
41185	[X]Dementia in human immunodef virus [HIV] disease
44288	[D]Laboratory evidence of human immunodeficiency virus [HIV]
44303	Human immunodef virus resulting in other disease
44617	HIV disease resulting in Burkitt's lymphoma
47632	HIV disease result/haematological+immunologic abnorms,NEC
50076	HIV disease resulting in multiple infections
51708	HIV dis reslt/oth mal neopl/lymph,h'matopoetc+reltd tissu
53636	Human immunodeficiency virus with neurological disease
58857	Acute human immunodeficiency virus infection
58859	Asymptomatic human immunodeficiency virus infection
62854	[X]Human immunodeficiency virus disease
62891	Human immunodeficiency virus with other clinical findings
65117	HIV disease resulting in lymphoid interstitial pneumonitis
66367	HIV dis resulting oth types of non-Hodgkin's lymphoma
66368	HIV disease resulting in cytomegaloviral disease
67575	HIV disease resulting in unspecified malignant neoplasm
69766	HIV infection with persistent generalised lymphadenopathy
69767	[X]HIV disease resulting in other non-Hodgkin's lymphoma
70528	Human immunodeficiency virus with secondary infection
70869	Human immunodeficiency virus with constitutional disease
71450	HIV disease resulting/unspcf infectious+parasitic disease
96751	[X]HIV disease result/haematological+immunologic abnorms,NEC
100769	[X]Unspecified human immunodeficiency virus [HIV] disease
101836	Human immunodeficiency virus with secondary cancers
102117	[X]HIV disease resulting in multiple infections
102252	[X]HIV disease resulting in other specified conditions
104134	[X]HIV disease resulting/other infectious+parasitic diseases
104466	HIV disease complicating pregnancy childbirth puerperium

104717	HIV disease resulting in Pneumocystis jirovecii pneumonia
105040	HIV pos gen health check serv declind - enhanc service admin
105324	HIV disease resulting in multiple malignant neoplasms
107807	[X]HIV disease resulting in other viral infections
108054	HIV disease resulting in Kaposi sarcoma
27519	Pneumonia with pneumocystis carinii
101191	Human immunodeficiency virus annual review
540	HTLV-3 antibody positive
43537	HIV 1 nucleic acid detection

icd	icd_description
B20	Human immunodef virus dis result infectious parasitic dis
B20.0	HIV disease resulting in mycobacterial infection
B20.1	HIV disease resulting in other bacterial infections
B20.2	HIV disease resulting in cytomegaloviral disease
B20.3	HIV disease resulting in other viral infections
B20.4	HIV disease resulting in candidiasis
B20.5	HIV disease resulting in other mycoses
B20.6	HIV disease resulting in Pneumocystis carinii pneumonia
B20.7	HIV disease resulting in multiple infections
B20.8	HIV dis resulting in oth infectious and parasitic dis
B20.9	HIV disease resulting in unspec infectious or parasitic dis
B21	Human immunodef virus dis resulting malignant neopl
B21.0	HIV disease resulting in Kaposi's sarcoma
B21.1	HIV disease resulting in Burkitt's lymphoma
B21.2	HIV dis resulting oth types of non-Hodgkin's lymphoma
B21.3	HIV dis result oth mal neo lymphoid haematopoietic rel tis
B21.7	HIV disease resulting in multiple malignant neoplasms
B21.8	HIV disease resulting in other malignant neoplasms
B21.9	HIV disease resulting in unspecified malignant neoplasm
B22	Human immunodef virus dis resulting in other spec dis
B22.0	HIV disease resulting in encephalopathy
B22.1	HIV disease resulting in lymphoid interstitial pneumonitis
B22.2	HIV disease resulting in wasting syndrome
B22.7	HIV dis resulting in multiple diseases classif elsewhere
B23	Human immunodef virus dis resulting in other conditions
B23.0	Acute HIV infection syndrome
B23.1	HIV dis result (persistent) generalized lymphadenopathy
B23.2	HIV dis result haematologic / immunologic abnorm NEC
B23.8	HIV disease resulting in other specified conditions
B24	Unspecified human immunodefiency virus [HIV] disease

F02.4	Dementia in human immunodef virus [HIV] disease
Z21	Asymptomatic human immunodef virus [HIV] infect status

Lymphoma

Medcode	Readterm
1481	Reticulosarcoma
1483	[M]Lymphoma NOS
2462	Hodgkin's disease
3371	[M]Non Hodgkins lymphoma
3604	Non - Hodgkin's lymphoma
3845	Eosinophilic granuloma
4870	Histiocytosis X (acute, progressive)
5179	Nodular lymphoma (Brill - Symmers disease)
5572	Myelofibrosis
7940	[X]Non-Hodgkin's lymphoma NOS
8649	[X]Non-Hodgkin's lymphoma, unspecified type
9172	[M]Waldenstrom's macroglobulinaemia
10411	Waldenstrom's macroglobulinaemia
12006	Mycosis fungoides
12323	Malignant neoplasm of lymphatic and haemopoietic tissue
12335	Malignant lymphoma NOS
12464	Peripheral T-cell lymphoma
15027	Malignant lymphoma NOS
15036	Malignant mast cell tumours
15504	Malignant lymphoma NOS of lymph nodes of multiple sites
16460	[M]Malignant lymphoma, non Hodgkin's type
16527	Macroglobulinaemia
16774	[M] Cutaneous lymphoma
17178	[M]Lymphomas, NOS or diffuse
17182	Follicular lymphoma NOS
17460	Diffuse non-Hodgkin's lymphoblastic (diffuse) lymphoma
17887	Malignant lymphoma otherwise specified
18383	[M] Large cell lymphoma
19140	Hodgkin's nodular sclerosis of lymph nodes of multiple sites
20437	[M]Lymphomas, nodular or follicular
20710	[M]Hodgkin's disease
21402	Burkitt's lymphoma
21463	[M]Lymphocytic lymphoma NOS
21549	Follicular non-Hodgkin's lymphoma
23711	[M]Malignant lymphoma, diffuse NOS
26111	Lymphomatoid papulosis
26135	[M] Alpha heavy chain disease
27416	Lymphosarcoma
27562	[M]Follicular lymphosarcoma NOS
27965	[M]AngiocentricT-cell lymphoma

28639	Follicular non-Hodgkin's small cleaved cell lymphoma
29178	Hodgkin's disease, nodular sclerosis
29876	Hodgkin's, lymphocytic-histiocytic predominance NOS
30646	Malignant neoplasm lymphatic or haematopoietic tissue OS
31324	Mast cell malignancy of lymph nodes of multiple sites
31492	[M] Monocytoid B-cell lymphoma
31537	[M]Hodgkin,s disease, lymphocytic predominance, nodular
31576	Other types of follicular non-Hodgkin's lymphoma
31726	[M]Malignant lymphoma, small cleaved cell, diffuse
31741	[M]Hodgkin,s disease, nodular sclerosis, lymphocytic deplet
31749	[M]Monocytoid B-cell lymphoma
31794	Unspecified B-cell non-Hodgkin's lymphoma
32240	Lymphoma stage III
33333	Other malignant neoplasm of lymphoid and histiocytic tissue
33869	[M]Malignant lymphoma, large cell, diffuse NOS
34089	Malignant lymphoma NOS of lymph nodes of axilla and arm
34352	[M]Lymphoblastic lymphoma NOS
34926	Letterer-Siwe disease
35014	Sezary's disease
36114	[M]Malignant lymphoma NOS
36736	Histiocytosis X , unspecified
37112	Malignant neoplasm of histiocytic tissue
38005	Mycosis fungoides NOS
38939	Hodgkin's disease, lymphocytic-histiocytic predominance
39798	Diffuse non-Hodgkin's lymphoma, unspecified
39883	[M]Malig lymp, follicular centre cell, cleaved, follicular
39906	[M]Malignant lymphoma, centrocytic
40000	Langerhans' cell histiocytosis
40508	[M]Hodgkin,s disease, nodular sclerosis, lymphocytic predom
40513	[M]Lymphoma, nodular or follicular NOS
40740	[X]Malignant neoplasms of lymphoid, haematopoietic and rela
40766	[M] Peripheral T-cell lymphoma NOS
40991	Lymphoma staging system
41369	Lymphosarcoma and reticulosarcoma
41754	[M]Malignant lymphoma, lymphoplasmacytoid type
41841	[M]Malignant lymphoma, follicular centre cell NOS
42198	[M]Hodgkin's disease, nodular sclerosis NOS
42461	Hodgkin's disease NOS
42579	Malignant lymphoma NOS of intra-abdominal lymph nodes
42769	[M]Hodgkin's disease NOS
43415	[X]Other Hodgkin's disease
44196	Hodgkin's granuloma
44267	Malignant histiocytosis

44318	Oth and unspecif peripheral & cutaneous T-cell lymphomas
44617	HIV disease resulting in Burkitt's lymphoma
45264	Nodular lymphoma of lymph nodes of head, face and neck
45768	[M]Acute progressive histiocytosis X
46877	[M]Malignant lymphoma, small lymphocytic NOS
46931	[M]Malignant lymphoma, stem cell type
46967	[M]Mycosis fungoides
47204	Letterer-Siwe disease NOS
47330	[M]HISTIOCYTIC MEDULLARY RETICULOSIS
48253	[M]Malignant lymphoma, immunoblastic type
49253	[M]Giant follicular lymphoma
49262	Follicular non-Hodgkin's large cell lymphoma
49301	Malignant neoplasm lymphatic or haematopoietic tissue NOS
49605	Hodgkin's disease, mixed cellularity
49825	[M]Reticulum cell sarcoma NOS
50668	Diffuse non-Hodgkin's small cell (diffuse) lymphoma
50695	Diffuse non-Hodgkin mixed sml & lge cell (diffuse) lymphoma
50696	Malignant lymphoma NOS of lymph nodes of head, face and neck
51285	[M]Hodgkin's disease, mixed cellularity
51680	[M]Malignant lymphoma, small cell, noncleaved, diffuse
51718	Histiocytosis X , chronic
51852	[M]Malig lymphoma, lymphocytic, intermediate different NOS
51895	[M]Lymphoma, diffuse or NOS
52591	[M]LYMPHOBLASTOMA NOS
52593	[M] Gamma heavy chain disease
53397	Hodgkin's disease NOS
53551	Diffuse non-Hodgkin's immunoblastic (diffuse) lymphoma
54083	Letterer-Siwe disease of lymph nodes of multiple sites
54190	[M] Angioimmunoblastic lymphadenopathy
54411	Hand - Schuller - Christian disease
55303	Hodgkin's nodular sclerosis of head, face and neck
56041	[M]Hodgkin's disease, lymphocytic predominance
57225	Hodgkin's disease, nodular sclerosis of unspecified site
57427	Malignant lymphoma NOS of unspecified site
57509	Waldenstrom's hypergammaglobulinaemic purpura
57544	[M]True histiocytic lymphoma
57737	Lymphoepithelioid lymphoma
58015	[M]Malignant lymphomatous polyposis
58082	Nodular lymphoma of lymph nodes of multiple sites
58684	Hodgkin's mixed cellularity of intrathoracic lymph nodes
58871	Malignant histiocytosis NOS
58953	[M]Malig lymp,follicular centre cell,noncleaved,follicular
58962	Malignant immunoproliferative small intestinal disease

59115	Burkitt's lymphoma of lymph nodes of head, face and neck
59593	[M]Letterer - Siwe disease
59755	Hodgkin's disease NOS of intrathoracic lymph nodes
59778	Hodgkin's disease NOS of lymph nodes of head, face and neck
60092	Malignant lymphoma NOS of spleen
60242	Reticulosarcoma of unspecified site
60275	[M]Malignant lymphoma, centroblastic type NOS
60504	[M]Lymphocytic lymphosarcoma NOS
60918	Lymphoma stage I
61149	Hodgkin's nodular sclerosis of intra-abdominal lymph nodes
61251	[M]Malign lymphoma,lymphocytic,intermediate differn, diffuse
61662	Hodgkin's disease NOS, unspecified site
61997	[M]Hodgkin's disease NOS
62380	Lymphosarcoma of intrathoracic lymph nodes
62437	Malignant reticulosis
63054	Hodgkin's disease, nodular sclerosis NOS
63105	Malignant lymphoma NOS of lymph node inguinal region and leg
63239	[M]Malignant histiocytosis
63375	[X]Unspecified B-cell non-Hodgkin's lymphoma
63625	Hodgkin's lymphocytic depletion lymph nodes axilla and arm
63699	[M]Malignant lymphoma, nodular NOS
63723	Lymphosarcoma NOS
63973	[M]Microglioma
63994	[M]Malignant lymphoma, large cell, cleaved, diffuse
64036	Hodgkin's sarcoma
64336	[X]Other specified types of non-Hodgkin's lymphoma
64343	[M]Hodgkin,s disease, nodular sclerosis, mixed cellularity
64427	Unspec malig neop lymphoid/histiocytic lymph node head/neck
64515	[X]Diffuse non-Hodgkin's lymphoma, unspecified
64670	Lymphosarcoma of intra-abdominal lymph nodes
64947	[M]Brill - Symmers' disease
65180	Diffuse non-Hodgkin's lymphoma undifferentiated (diffuse)
65434	Malignant neoplasms of lymphoid and histiocytic tissue NOS
65483	Hodgkin's nodular sclerosis of lymph nodes of axilla and arm
65489	Hodgkin's paragranuloma
65584	[M]Hodgkin,s disease, lymphocytic predominance, diffuse
65642	Malignant histiocytosis of intra-abdominal lymph nodes
65701	Nodular lymphoma NOS
66327	Nodular lymphoma of unspecified site
66367	HIV dis resulting oth types of non-Hodgkin's lymphoma
66603	[M]Malig lymphoma, follicular centre cell, non-cleaved NOS
67203	[M]Lymphoblastic lymphosarcoma NOS
67339	[M]MALIGNANT MASTOCYTOSIS

67506	Hodgkin's nodular sclerosis of intrathoracic lymph nodes
67518	[X]Other types of follicular non-Hodgkin's lymphoma
67703	Hodgkin's disease, lymphocytic depletion
68039	Hodgkin's sarcoma of lymph nodes of axilla and upper limb
68330	Hodgkin's, lymphocytic-histiocytic pred of head, face, neck
68964	[M]Malignant lymphoma, centroblastic-centrocytic, diffuse
69301	[M]Malignant lymphoma, convoluted cell type NOS
69497	Malignant histiocytosis of unspecified site
69767	[X]HIV disease resulting in other non-Hodgkin's lymphoma
69980	[M]Malignant lymphoma, lymphocytic, well differentiated NOS
70374	Reticulosarcoma of intra-abdominal lymph nodes
70509	Diffuse non-Hodgkin's centroblastic lymphoma
70716	Immunoproliferative neoplasm
70740	[M]Malignant reticulosis
70842	Follicular non-Hodg mixed sml cleavd & lge cell lymphoma
71031	Reticulosarcoma of lymph nodes of head, face and neck
71117	[M]Malignant lymphoma, undifferentiated cell type NOS
71142	Hodgkin's, lymphocytic-histiocytic predominance unspec site
71238	Lymphosarcoma of lymph nodes of head, face and neck
71262	Malignant lymphoma NOS of intrapelvic lymph nodes
71304	Burkitt's lymphoma NOS
71609	Unspec malig neop lymphoid/histiocytic nodes inguinal/leg
71619	[M]Malignant lymphoma, large cell, noncleaved, diffuse
71625	Lymphosarcoma of unspecified site
71652	[M]Malignant lymphoma, mixed small and large cell, diffuse
71672	Lymphoma stage IV
71994	Macroglobulinaemia NOS
72196	[M]Malignant lymphoma, lymphocytic, poorly different NOS
72241	[M]Prolymphocytic lymphosarcoma
72433	[M]Reticulosarcoma NOS
72500	[X]Mal neoplasm/lymphoid,haematopoietic+related tissu,unspcf
72714	Mycosis fungoides of lymph nodes of inguinal region and leg
72725	Malignant lymphoma NOS of intrathoracic lymph nodes
73532	Hodgkin's, lymphocytic-histiocytic pred intra-abdominal node
89230	[M]Hodgkin's granuloma
89657	Malignant mast cell tumour NOS
90201	T-zone lymphoma
91674	Mycosis fungoides of intra-abdominal lymph nodes
91900	Hodgkin's disease NOS of lymph nodes of axilla and arm
92068	Nodular lymphoma of intra-abdominal lymph nodes
92245	Hodgkin's, lymphocytic-histiocytic pred intrathoracic nodes
92380	Burkitt's lymphoma of lymph nodes of inguinal region and leg
93384	Unspec malig neop lymphoid/histiocytic of intrathoracic node

93951	Hodgkin's, lymphocytic-histiocytic pred inguinal and leg
94005	Hodgkin's disease, mixed cellularity NOS
94239	[M]Mast cell sarcoma
94279	Hodgkin's disease NOS of spleen
94407	Hodgkin's mixed cellularity of lymph nodes head, face, neck
94415	Malignant histiocytosis of lymph nodes head, face and neck
94935	Lymphoma stage II
94995	Nodular lymphoma of lymph nodes of inguinal region and leg
95012	Mycosis fungoides of lymph nodes of multiple sites
95049	Hodgkin's lymphocytic depletion of unspecified site
95058	Reticulosarcoma of spleen
95338	Hodgkin's, lymphocytic-histiocytic pred intrapelvic nodes
95464	[M]Mycosis fungoides
95545	Maltoma
95630	True histiocytic lymphoma
95715	Mucosa-associated lymphoma
95792	Lymphoid and histiocytic malignancy NOS
95949	Mycosis fungoides of unspecified site
96183	[M]Hodgkin's disease,lymphocytic depletion,diffuse fibrosis
96379	Mycosis fungoides of lymph nodes of axilla and upper limb
97577	Burkitt's lymphoma of intra-abdominal lymph nodes
97746	Hodgkin's disease NOS of lymph nodes of multiple sites
97756	[M]Sezary's disease
97852	[M]Malignant lymphoma, centroblastic type, follicular
97863	Hodgkin's disease, mixed cellularity of unspecified site
98596	[X]Other types of diffuse non-Hodgkin's lymphoma
98840	Hodgkin's paragranuloma of intra-abdominal lymph nodes
98909	Hodgkin's granuloma of lymph nodes of head, face and neck
98961	[M]Malignant lymphoma, centroblastic-centrocytic, follicular
99012	Hodgkin's disease NOS of lymph nodes inguinal region and leg
99067	Gamma heavy chain disease
99200	[M]Hodgkin's disease, nodular sclerosis, cellular phase
99240	Reticulosarcoma NOS
99655	[M]Lymphosarcoma NOS
99695	[M]Mycosis fungoides NOS
99887	Other specified reticulosarcoma or lymphosarcoma
99951	Reticulosarcoma or lymphosarcoma NOS
100006	Burkitt's lymphoma of intrathoracic lymph nodes
100352	Lymphosarcoma of lymph nodes of inguinal region and leg
100423	Hodgkin's paragranuloma of lymph nodes of head, face, neck
100532	Sezary's disease NOS
100544	[M]Reticulosarcoma, nodular
100615	Mast cell malignancy of lymph nodes inguinal region and leg

101114	Diffuse non-Hodgkin's large cell lymphoma
101350	Alpha heavy chain disease
101429	[M]Lymphogranuloma, malignant
101465	Unspec malig neop lymphoid/histiocytic of multiple sites
101530	Hodgkin's disease, lymphocytic depletion NOS
101715	Hodgkin's disease, lymphocytic depletion of spleen
102158	Letterer-Siwe disease of intrathoracic lymph nodes
102594	Diffuse large B-cell lymphoma
102688	[X]Other malignant immunoproliferative diseases
102715	LETTERER-SIWE DISEASE OF UNSPECIFIED SITES
103245	Lymphosarcoma of spleen
103353	Unspec malig neop lymphoid/histiocytic intra-abdominal nodes
103900	Mast cell malignancy of unspecified site
104152	Follicular lymphoma
104291	Hodgkin lymphoma
104386	Small cell B-cell lymphoma
104391	Non-Hodgkin lymphoma
104412	Lymphoblastic (diffuse) lymphoma
104484	Other classical Hodgkin lymphoma
104620	Mantle cell lymphoma
104743	Hodgkin's, lymphocytic-histiocytic pred of multiple sites
104790	Lymphosarcoma of lymph nodes of multiple sites
104862	Cutaneous T-cell lymphoma
104895	Nodular lymphocyte predominant Hodgkin lymphoma
104934	Other mature T/NK-cell lymphoma
105020	Follicular lymphoma grade 3a
105025	[X]OTH SPCF MAL NEOPLSM/LYMPHOID,HAEMATOPOIETIC+RLTD TISSUE
105038	Mediastinal (thymic) large B-cell lymphoma
105085	T/NK-cell lymphoma
105095	Follicular lymphoma grade 2
105203	Nodular lymphoma of intrathoracic lymph nodes
105375	Blastic NK-cell lymphoma
105472	Hodgkin's disease, nodular sclerosis of spleen
105559	Anaplastic large cell lymphoma, ALK-positive
105636	Angioimmunoblastic T-cell lymphoma
105709	Enteropathy-associated T-cell lymphoma
105762	UNIFOCAL LANGERHANS-CELL HISTIOCYTOSIS
105841	Nodular sclerosis classical Hodgkin lymphoma
105889	Follicular lymphoma grade 1
105925	Subcutaneous panniculitic T-cell lymphoma
105955	Anaplastic large cell lymphoma, ALK-negative
105966	EXTRANOD MARG ZONE B-CELL LYMPHOM MUCOSA-ASSOC LYMPHOID TISS
106063	Other types of follicular lymphoma

106137	[M]RETICULOSARCOMAS
106349	Hodgkin lymphoma NOS
106597	Lymphocyte-rich classical Hodgkin lymphoma
106867	Non-follicular lymphoma
106884	Nonfollicular lymphoma
106911	Hodgkin's, lymphocytic-histiocytic predominance of spleen
106969	Diffuse follicle centre lymphoma
106970	[M]Malig lymphoma, lymphocytic, well differentiated,nodular
107032	Hodgkin's lymphocytic depletion lymph nodes multiple sites
107166	Follicular lymphoma grade 3
107804	Hodgkin's disease NOS of intra-abdominal lymph nodes
107949	Hepatosplenic T-cell lymphoma
107973	Follicular lymphoma grade 3b
108037	Unspec malig neop lymphoid/histiocytic of unspecified site
108102	Heavy chain disease
108182	Diffuse non-Hodgkin's small cleaved cell (diffuse) lymphoma
108235	Waldenstrom macroglobulinaemia
108719	Cutaneous follicle centre lymphoma
108775	Mixed cellularity classical Hodgkin lymphoma
108886	Hodgkin's mixed cellularity of lymph nodes inguinal and leg
109342	Unspec malig neop lymphoid/histiocytic of intrapelvic nodes
109714	[X]Oth and unspecif peripheral & cutaneous T-cell lymphomas
109780	Extranodal NK/T-cell lymphoma, nasal type

icd	icd_description
C81	Hodgkin's disease
C81.0	Hodgkin's disease, lymphocytic predominance
C81.1	Hodgkin's disease, nodular sclerosis
C81.2	Hodgkin's disease, mixed cellularity
C81.3	Hodgkin's disease, lymphocytic depletion
C81.7	Hodgkin's disease, other Hodgkin's disease
C81.9	Hodgkin's disease, Hodgkin's disease, unspecified
C82	Follicular [nodular] non-Hodgkin's lymphoma
C82.0	Follicular non-Hodgkin's small cleaved cell lymphoma
C82.1	Follicular non-Hodg mixed sml cleavd & lge cell lymphoma
C82.2	Follicular non-Hodgkin's large cell lymphoma
C82.7	Follicular non-Hodgkin's other types of lymphoma
C82.9	Follicular non-Hodgkin's unspecified lymphoma
C83	Diffuse non-Hodgkin's lymphoma
C83.0	Diffuse non-Hodgkin's small cell (diffuse)lymphoma
C83.1	Diffuse non-Hodgkin's small cleaved cell (diffuse) lymphoma

C83.2	Diffuse non-Hodgkin mixed sml & lge cell (diffuse) lymphoma
C83.3	Diffuse non-Hodgkin's large cell (diffuse) lymphoma
C83.4	Diffuse non-Hodgkin's immunoblastic (diffuse) lymphoma
C83.5	Diffuse non-Hodgkin's lymphoblastic (diffuse) lymphoma
C83.6	Diffuse non-Hodgkin's lymphoma undifferentiated (diffuse)
C83.7	Diffuse non-Hodgkin's lymphoma, Burkitt's tumour
C83.8	Other types of diffuse non-Hodgkin's lymphoma
C83.9	Diffuse non-Hodgkin's lymphoma, unspecified
C84	Peripheral and cutaneous T-cell lymphomas
C84.0	Peripheral and cutaneous T-cell lymphomas, mycosis fungoides
C84.1	Peripheral and cutaneous T-cell lymphomas, Sezary's disease
C84.2	Peripheral and cutaneous T-cell lymphomas, T-zone lymphoma
C84.3	Periph & cutan T-cell lymphomas, lymphoepithelioid lymphoma
C84.4	Periph & cutan T-cell lymphomas, peripheral T-cell lymphoma
C84.5	Periph & cutan T-cell lymphomas, oth & unsp T-cell lymphomas
C85	Other and unspecified types of non-Hodgkin's lymphoma
C85.0	Oth & unspec types of non-Hodgkin's lymphoma, lymphosarcoma
C85.1	Oth & unsp types non-Hodgkin's B-cell lymphoma, unsp
C85.7	Oth specified types of non-Hodgkin's lymphoma
C85.9	Non-Hodgkin's lymphoma, unspecified type
C88	Malignant immunoproliferative diseases
C88.0	Waldenstrom's macroglobulinaemia
C88.1	Alpha heavy chain disease
C88.2	Gamma heavy chain disease
C88.3	Malignant immunoproliferative small intestinal disease
C88.7	Other malignant immunoproliferative diseases
C88.9	Malignant immunoproliferative disease, unspecified
C96	Oth & unspec malig neop lymphoid, haematapoietic & rel tiss
C96.0	Letterer-Siwe disease
C96.1	Malignant histiocytosis
C96.2	Malignant mast cell tumour
C96.3	True histiocyt lymphoma
C96.7	Oth spec malig neop lymphoid h'poietic & related tissue
C96.9	Malig neop lymphoid haematopoietic and related tissue unspec

Leukaemia

Medcode	Readterm
4072	Acute leukaemia NOS
4222	Lymphatic leukaemia
4250	Leukaemia NOS
4251	Acute lymphoid leukaemia
4413	Acute myeloid leukaemia
4637	[M]Leukaemias
5137	Leukaemic reticuloendotheliosis
5915	[M]Hairy cell leukaemia
6316	[M]Acute leukaemia NOS
7176	Myeloid leukaemia
8625	Chronic lymphoid leukaemia
10726	Chronic myeloid leukaemia
12146	[M]Lymphoid leukaemia NOS
16416	Chronic leukaemia NOS
19372	Lymphoid leukaemia
19974	Acute monocytic leukaemia
20440	Myelomonocytic leukaemia
20635	[M]Lymphatic leukaemia
22050	Chronic myelomonocytic leukaemia
22071	[M]Blast cell leukaemia
25191	Leukaemia of unspecified cell type
27330	Leukaemic reticuloendotheliosis
27340	Di Guglielmo's disease
27458	Chronic monocytic leukaemia
27520	Chronic myeloid leukaemia NOS
27664	Acute promyelocytic leukaemia
27790	Chronic lymphatic leukaemia
28276	Acute myelofibrosis
29335	[M]Adult T-cell leukaemia/lymphoma
30632	Other specified leukaemia NOS
31586	Prolymphocytic leukaemia
31701	Chronic granulocytic leukaemia
31750	[M]Chronic leukaemia NOS
33344	Myeloid leukaemia NOS
34692	Other leukaemia of unspecified cell type
35697	[M]Myeloid leukaemias
35875	Monocytic leukaemia
37272	Other specified leukaemia
37410	[M]Acute lymphoid leukaemia
37461	Adult T-cell leukaemia

37487	[M]Acute myelofibrosis
37723	[M]Granulocytic leukaemia NOS
38331	Other lymphoid leukaemia NOS
38914	Lymphoid leukaemia NOS
39629	Granulocytic sarcoma
40420	[M]Leukaemias unspecified
41500	[M]Chronic lymphoid leukaemia
41734	[M]Leukaemia NOS
42297	[M]Leukaemia NOS
42539	Acute erythraemia and erythroleukaemia
44420	Refractory anaemia with excess of blasts with transformation
46048	[M]Prolymphocytic leukaemia
46263	[M]Acute myelomonocytic leukaemia
46444	[M]Erythroleukaemias
48049	[M]Chronic myelomonocytic leukaemia
48155	[M]Lymphoid leukaemias
49327	[M]Acute megakaryoblastic leukaemia
49725	Other lymphoid leukaemia
50858	Acute panmyelosis
50928	[M]Burkitt's cell leukaemia
52327	Chloroma
52942	[M]Chronic myeloid leukaemia
54585	[M]Acute myeloid leukaemia
54793	Subacute leukaemia NOS
57316	[M]Acute promyelocytic leukaemia
57671	Megakaryocytic leukaemia
57713	[M]Eosinophilic leukaemias
59929	[M]Leukaemia unspecified, NOS
61500	Acute myelomonocytic leukaemia
61693	[X]Other myeloid leukaemia
62330	[M]Other myeloid leukaemia NOS
63475	Subacute myeloid leukaemia
63570	[M]Stem cell leukaemia
64963	[M]Blastic leukaemia
65122	Leukaemic reticuloendotheliosis of unspecified sites
65123	Leukaemic reticuloend of intra-abdominal lymph nodes
65165	[X]Other leukaemia of unspecified cell type
65721	Mast cell leukaemia
65777	Thrombocytic leukaemia
66089	Other myeloid leukaemia NOS
66694	[M]Naegeli-type monocytic leukaemia
67029	[X]Other lymphoid leukaemia
67700	Monoblastic leukaemia

69299	[M]Thrombocytic leukaemia
70724	Myeloid sarcoma
70935	[M]Erythroleukaemia
71377	[M]Eosinophilic leukaemia
71850	[M]Myeloid leukaemia NOS
72179	[M]Subacute leukaemia NOS
72197	Lymphosarcoma cell leukaemia
72222	[M]Megakaryocytic leukaemia
72310	[M]Aleukaemic leukaemia NOS
72774	Subacute lymphoid leukaemia
73066	[M]Miscellaneous leukaemias
73088	[M]Monocytic leukaemia NOS
73777	Leukaemic reticuloendotheliosis NOS
87335	Hairy cell leukaemia
89329	[X]Other specified leukaemias
89762	[X]Other monocytic leukaemia
93342	Monocytic leukaemia NOS
93944	[M]Chloroma
94174	Other and unspecified leukaemia
96893	[M]Myeloid sarcoma
98009	[M]Granulocytic sarcoma
99015	Other monocytic leukaemia
99413	Other and unspecified leukaemia NOS
100786	Chronic eosinophilic leukaemia
100927	[M]Erythroleukaemia NOS
101271	[M]Acute panmyelosis
101606	Subacute monocytic leukaemia
102764	[M]Acute panmyelosis
102783	Chronic neutrophilic leukaemia
104325	B-cell acute lymphoblastic leukaemia
104328	B-cell chronic lymphocytic leukaemia
104475	Subacute myelomonocytic leukaemia
104788	Acute myeloblastic leukaemia
104939	Adult T-cell lymphoma/leukaemia (HTLV-1-associated)
105069	Juvenile myelomonocytic leukaemia
105957	Chronic myeloid leukaemia, BCR/ABL positive
106197	[M]Basophilic leukaemia
106483	[M]Subacute myeloid leukaemia
106924	Clinical stage B chronic lymphocytic leukaemia
107017	Chronic lymphocytic leukaemia of B-cell type
107052	Clinical stage A chronic lymphocytic leukaemia
107163	Clinical stage C chronic lymphocytic leukaemia
107236	Atypical chronic myeloid leukaemia, BCR/ABL negative

107643	T-cell prolymphocytic leukaemia
107773	[M]Eosinophilic leukaemia NOS
108316	[M]Miscellaneous leukaemia NOS
108424	Acute monoblastic leukaemia
108656	B-cell prolymphocytic leukaemia
108715	Histiocytic leukaemia
108964	[M]Juvenile myelomonocytic leukaemia

icd	icd_description
C91	Lymphoid leukaemia
C91.0	Acute lymphoblastic leukaemia
C91.1	Chronic lymphocytic leukaemia
C91.2	Subacute lymphocytic leukaemia
C91.3	Prolymphocytic leukaemia
C91.4	Hairy-cell leukaemia
C91.5	Adult T-cell leukaemia
C91.7	Other lymphoid leukaemia
C91.9	Lymphoid leukaemia, unspecified
C92	Myeloid leukaemia
C92.0	Acute myeloid leukaemia
C92.1	Chronic myeloid leukaemia
C92.2	Subacute myeloid leukaemia
C92.3	Myeloid sarcoma
C92.4	Acute promyelocytic leukaemia
C92.5	Acute myelomonocytic leukaemia
C92.7	Other myeloid leukaemia
C92.9	Myeloid leukaemia, unspecified
C93	Monocytic leukaemia
C93.0	Acute monocytic leukaemia
C93.1	Chronic monocytic leukaemia
C93.2	Subacute monocytic leukaemia
C93.7	Other monocytic leukaemia
C93.9	Monocytic leukaemia, unspecified
C94	Other leukaemias of specified cell type
C94.0	Acute erythraemia & erythroleukaemia
C94.1	Chronic erythraemia
C94.2	Acute megakaryoblastic leukaemia
C94.3	Mast cell leukaemia
C94.4	Acute panmyelosis
C94.5	Acute myelofibrosis
C94.7	Other specified leukaemias

C95	Leukaemia of unspecified cell type
C95.0	Acute leukaemia of unsp cell type
C95.1	Chronic leukaemia unsp cell type
C95.2	Subacute leukaemia unsp cell type
C95.7	Other leukaemia unspecified cell type
C95.9	Leukaemia, unspecified

Myeloma

Medcode	Readterm
3672	[M]Myeloma NOS
4944	Multiple myeloma
15211	Myelomatosis
18744	[M]Multiple myeloma
19028	Solitary myeloma
21329	Plasmacytoma NOS
22158	Malignant plasma cell neoplasm, extramedullary plasmacytoma
31671	[M]Plasma cell myeloma
39187	Plasma cell leukaemia
39490	[M]Plasmacytic myeloma
43312	Myeloma - solitary
43552	Kahler's disease
46042	Lambda light chain myeloma
52946	Bone marrow: myeloma cells
53647	[M]Myelomatosis
59663	Neoplasm of uncertain behaviour of plasma cells
60433	Osteoporosis in multiple myelomatosis
63864	[M]Plasmacytoma NOS
64618	[M]Plasma cell leukaemias
73135	[M]Solitary myeloma
99702	[M]Plasma cell tumour, malignant
102164	[M]Monostotic myeloma
104418	Solitary plasmacytoma
38321	Plasmacytoma NOS
37182	Multiple myeloma and immunoproliferative neoplasms
43450	Immunoproliferative neoplasm or myeloma NOS

icd	icd_description
C90	Multiple myeloma and malignant plasma cell neoplasms
C90.0	Multiple myeloma
C90.1	Plasma cell leukaemia
C90.2	Malignant plasma cell neoplasm, extramedullary plasmacytoma

Haematopoietic stem cell transplantation

Medcode	Readterm
28232	Peripheral blood stem cell graft
86063	Autologous peripheral blood stem cell transplant
89920	Allogeneic peripheral blood stem cell transplant
18628	Transfusion of stem cells
54420	Second stage peripheral stem cell infusion
63236	First stage peripheral stem cell infusion
1392	Bone marrow transplant
15406	Allograft of bone marrow NEC
21021	Graft of bone marrow
34414	Autograft of bone marrow
52943	Bone-marrow transplant rejection
70870	Graft of bone marrow NOS
72436	Other specified graft of bone marrow
85492	Allograft of bone marrow from sibling donor
95840	Allograft of bone marrow from matched unrelated donor
98608	Allograft of bone marrow from unmatched unrelated donor
100912	Allograft of bone marrow from haploidentical donor
22726	Transfusion of bone marrow
73013	Inj.bone marrow - local action
93362	Allograft of cord blood to bone marrow
100078	Graft of cord blood to bone marrow

icd	icd_description
T86.0	Bone-marrow transplant rejection

opcs	description
X334	Autologous peripheral blood stem cell transplant
X335	Syngeneic peripheral blood stem cell transplant
X336	Allogeneic peripheral blood stem cell transplant
W34	Graft of bone marrow
W341	Autograft of bone marrow
W342	Allograft of bone marrow NEC
W343	Allograft of bone marrow from sibling donor
W344	Allograft of bone marrow from matched unrelated donor
W345	Allograft of bone marrow from haploidentical donor
W346	Allograft of bone marrow from unmatched unrelated donor
W348	Other specified graft of bone marrow

W349	Unspecified graft of bone marrow
W99	Graft of cord blood stem cells to bone marrow
W991	Allograft of cord blood stem cells to bone marrow
W998	Other specified graft of cord blood stem cells to bone marrow
W999	Unspecified graft of cord blood stem cells to bone marrow

Other immunosuppressive disease

Medcode	Readterm
938	Pancytopenia NOS
5823	Pancytopenia - acquired
10955	Di George syndrome
21975	Common variable immunodeficiency
31275	Pancytopenia with malformation
31322	Wiskott - Aldrich syndrome
31491	Pancytopenia-dysmelia
42394	Job's syndrome
42439	Thrombocytopenic eczema with immunodeficiency
48293	Severe combined immunodeficiency
57322	Common variable immunodeficiency
61326	Pancytopenia with pancreatitis
62236	Combined immunity deficiency
62328	Combined immunity deficiency NOS
73583	Ataxia-telangiectasia
30628	Bone marrow depression
22307	Patient immunosuppressed
47106	Patient immunocompromised
31541	Severe combined immunodefiency with reticular dysgenesis
39800	Immunodeficiency associated+major defect, unspecified
49542	Severe combined immunodef with low or normal B-cell numbers
50526	Major histocompatibility complex class I deficiency
65617	Immunodeficiency with short-limbed stature
66073	Severe combined immunodef with low T- and B-cell numbers
66857	Common variable immunodef wth autoantibod to B- or T-cells
72804	Adenosine deaminase deficiency
93936	Purine nucleoside phosphorylase deficiency
103977	Major histocompatibility complex class II deficiency
104651	Immune reconstitution syndrome
15422	Aplastic anaemia
15658	Acquired aplastic anaemia
16108	Aplastic anaemia due to chronic disease
21723	Acquired aplastic anaemia NOS
41142	Idiopathic aplastic anaemia
43166	Aplastic anaemia due to drugs
57859	Aplastic anaemia due to infection
66239	Aplastic anaemia due to toxic cause
68087	Aplastic anaemia NOS
70128	Radiation aplastic anaemia
73131	[X]Other common variable immunodeficiency

icd	icd_description
D61.1	Drug-induced aplastic anaemia
D61.2	Aplastic anaemia due to other external agents
D61.3	Idiopathic aplastic anaemia
D61.8	Other specified aplastic anaemias
D61.9	Aplastic anaemia, unspecified
D81	Combined immunodeficiencies
D81.0	Severe combined immunodefiency with reticular dysgenesis
D81.1	Severe combined immunodef with low T- and B-cell numbers
D81.2	Severe combined immunodef with low or normal B-cell numbers
D81.3	Adenosine deaminase [ADA] deficiency
D81.4	Nezelof's syndrome
D81.5	Purine nucleoside phosphorylase [PNP] deficiency
D81.6	Major histocompatibility complex class I deficiency
D81.7	Major histocompatibility complex class II deficiency
D81.8	Other combined immunodefiencies
D81.9	Combined immunodefiency, unspecified
D82.0	Wiskott-Aldrich syndrome
D82.1	Di George's syndrome
D82.2	immunodefiency with short-limbed stature
D83	Common variable immunodeficiency
D83.0	Com var immunodef with predom abn B-cell numb and funct
D83.1	Common var immunodef predom immunoregulatory T-cell disord
D83.2	Common variable immunodef autoantibodies to B- or T-cells
D83.8	Other common variable immunodefiencies
D83.9	Common variable immunodefiency, unspecified
D89.3	Immune reconstitution syndrome

Oral glucocorticoids

Prodcode	Product name
44	prednisolone enteric coated tablets 5mg
95	prednisolone tablets 5mg
186	dexamethasone elixir 0.5mg/5ml
229	cortisone acetate tablets 25mg
557	prednisolone enteric coated tablets 2.5mg
578	prednisolone tablets 1mg
955	prednisolone sodium phosphate soluble tablet 5mg
1063	PREDNESOL tablets 5mg [SOVEREIGN]
1280	dexamethasone tablets 2mg
1380	ENTOCORT CR modified release capsules 3mg [ASTRAZENEC]
1709	hydrocortisone pellets 2.5 mg loz
1971	BETNESOL tablets 0.5mg [FOCUS]
2044	prednisone 2.5 mg tab
2130	methylprednisolone tablets 4mg
2368	prednisolone tablets 2.5mg
2390	prednisolone e/c 1 mg tab
2704	prednisolone tablets 25mg
2799	prednisolone 10 mg tab
2949	prednisone tablets 5mg
3059	prednisolone 50 mg tab
3345	SINTISONE tablets [PHARMACIA]
3418	hydrocortisone tablets 10mg
3557	prednisone tablets 1mg
3898	budesonide modified release capsules 3mg
3969	dexamethasone 8 mg tab
3992	deflazacort tablets 6mg
4535	hydrocortisone tablets 20mg
4779	dexamethasone tablets 0.5mg
4943	dexamethasone sugar free oral solution 2mg/5ml
5157	dexamethasone oral solution 2mg/5ml
5490	DELTACORTRIL ENTERIC tablets 5mg [ALLIANCE]
5913	DELTACORTRIL ENTERIC tablets 2.5mg [ALLIANCE]
6095	budesonide capsules 3mg
6098	HYDROCORTONE tablets 10mg [M S D]
7286	betamethasone sodium phosphate soluble tablet 500micrograms
7548	cortisone acetate capsules 5mg
7584	prednisolone 4 mg tab
7710	prednisolone 15 mg tab
7934	prednisone 30 mg tab
8261	MEDRONE tablets 16mg [PHARMACIA]

9375	deflazacort tablets 1mg
9727	prednisolone tablets 50mg
9994	DECADRON tablets 0.5mg [M S D]
10431	DELTACORTRIL 1 MG TAB
10552	methylprednisolone tablets 16mg
10574	cortisone acetate tablets 5mg
10683	MEDRONE tablets 2mg [PHARMACIA]
10684	methylprednisolone tablets 2mg
10754	HYDROCORTISTAB tablets 20mg [WAYMADE]
10864	betamethasone tablets 500micrograms
11149	BETNELAN tablets 0.5mg [FOCUS]
12398	CORTELAN tablets 25mg [GLAXO]
12400	CORTISYL tablets 25mg [AVENTIS]
13043	HYDROCORTONE tablets 20mg [M S D]
13512	ORADEXON .5 MG TAB
13522	prednisolone 2 mg tab
13615	prednisone 10 mg tab
14076	hydrocortisone sugar free oral suspension 5mg/5ml
14172	methylprednisolone tablets 100mg
15471	hydrocortisone 25 mg tab
15555	MEDRONE tablets 4mg [PHARMACIA]
15617	LEDERCORT tablets 4mg [WYETH PHAR]
16525	BUDENOFALK capsules 3mg [DR FALK]
16724	prednisone 50 mg tab
17101	dexamethasone 750 mcg tab
17410	deflazacort tablets 30mg
18042	MEDRONE tablets 100mg [PHARMACIA]
18637	CORTISTAB tablets 25mg [WAYMADE]
18955	hydrocortisone 4.5 mg loz
19141	PREDNISOLONE soluble tablet 5mg [SOVEREIGN]
19908	triamcinolone tablets 2mg
20095	PRECORTISYL FORTE tablets 25mg [AVENTIS]
20577	CALCORT tablets 6mg [SHIRE]
20670	prednisolone e/c
20731	hydrocortisone pellets
21218	DEXSOL oral solution 2mg/5ml [ROSEMONT]
21417	PREDNISOLONE tablets 5mg [HILLCROSS]
21465	betamethasone .1 mg tab
21833	DECORTISYL tablets 5mg [ROUSSEL]
21903	ORADEXON-ORGANON tablets 2mg [ORGANON]
22555	CALCORT tablets 1mg [SHIRE]
22827	betamethasone .1 mg pel
23111	triamcinolone tablets 4mg

23210	CORTISTAB tablets 5mg [WAYMADE]
23512	PRECORTISYL tablets 5mg [HOECHSTMAR]
23788	cortisone acetate 2.5 mg tab
24014	LEDERCORT tablets 2mg [WYETH PHAR]
24716	prednisolone e/c
25272	PRECORTISYL tablets 1mg [HOECHSTMAR]
26806	DECADRON 2 MG TAB
27083	betamethasone valerate .1 mg tab
27720	hydrocortisone
27889	prednisolone
27959	prednisolone
27962	DELTASTAB tablets 1mg [WAYMADE]
28375	PREDNISOLONE enteric coated tablets 2.5mg [HILLCROSS]
28376	PREDNISOLONE enteric coated tablets 2.5mg [BIOREX]
28615	methylprednisolone l/a 4 mg cap
28859	DELTASTAB tablets 5mg [WAYMADE]
29112	CALCORT tablets 30mg [SHIRE]
29322	betamethasone loz
29333	PREDNISOLONE tablets 5mg [ACTAVIS]
30390	DELTASTAB 2 MG TAB
30971	DECORTISYL 25 MG TAB
31327	prednisolone steaglate tablets 6.65mg
31532	PREDNISOLONE enteric coated tablets 5mg [HILLCROSS]
32803	PREDNISOLONE enteric coated tablets 5mg [ACTAVIS]
32835	PREDNISOLONE tablets 5mg [WOCKHARDT]
33639	cortisone acetate msd 25 mg tab
33691	PREDNISOLONE enteric coated tablets 5mg [BIOREX]
33988	PREDNISOLONE tablets 5mg [CO-PHARMA]
33990	PREDNISOLONE tablets 5mg [IVAX]
34109	prednisolone enteric coated tablets 5mg
34393	PREDNISOLONE enteric coated tablets 5mg [TEVA]
34404	PREDNISOLONE tablets 1mg [ACTAVIS]
34452	PREDNISOLONE tablets 1mg [HILLCROSS]
34461	PREDNISOLONE enteric coated tablets 2.5mg [ACTAVIS]
34631	PREDNISOLONE tablets 1mg [CO-PHARMA]
34660	PREDNISOLONE tablets 1mg [KENT]
34748	PREDNISOLONE tablets 1mg [TEVA]
34781	PREDNISOLONE tablets 5mg [KENT]
34801	DEXAMETHASONE elixir 0.5mg/5ml [ROSEMONT]
34880	DEXAMETHASONE tablets 2mg [ORGANON]
34914	PREDNISOLONE tablets 1mg [CELLTECH]
34915	DEXAMETHASONE tablets 0.5mg [ORGANON]
34978	PREDNISOLONE tablets 1mg [WOCKHARDT]

36055	DEXAMETHASONE tablets 2mg [HILLCROSS]
36686	cortisone acetate msd 5 mg tab
37203	beclometasone gastro-resistant modified release tablets 5mg
38022	hydrocortisone oral suspension 10mg/5ml
38054	hydrocortisone tablets
38407	prednisolone (roi) tablets 20mg
39067	CLIPPER gastro-resistant modified release tablets 5mg [CHIESI]
41335	CALCORT tablets 6mg [SANOFI/AVE]
41515	PREDNISOLONE tablets 5mg [TEVA]
41745	PREDNISOLONE tablets 25mg [WINTHROP]
43544	PREDNISONE tablets 5mg [KNOLL]
44380	prednisone modified release tablet 1mg
44723	prednisone modified release tablet 5mg
44802	LODOTRA modified release tablet 5mg [NAPPPHARM]
44803	LODOTRA modified release tablet 2mg [NAPPPHARM]
45234	dexamethasone capsules
45302	PREDNISOLONE tablets 5mg [BIOREX]
46711	prednisone modified release tablet 2mg
47142	Prednisolone 5mg Soluble tablet (Amdipharm Plc)
50225	Betnesol 500microgram soluble tablets (Waymade Healthcare Plc)
51722	Hydrocortisone 5mg/5ml oral suspension
51753	Prednisolone 1mg tablets (Co-Pharma Ltd)
51824	Hydrocortisone 5mg/5ml oral suspension sugar free
51849	Hydrocortisone 1mg/5ml oral suspension
51871	Hydrocortisone 2mg capsules
51872	Hydrocortisone 2.5mg capsules
52053	Hydrocortisone 3mg/5ml oral suspension
52396	Dexamethasone 1mg/5ml oral solution
53143	Cortisone 25mg tablets (A A H Pharmaceuticals Ltd)
53207	Dexamethasone tablets
53313	Prednisolone 20mg/5ml oral suspension
53336	Prednisolone 25mg tablets (A A H Pharmaceuticals Ltd)
53705	Cortisone acetate 5mg Capsule (Martindale Pharmaceuticals Ltd)
53953	Hydrocortisone 5mg modified-release tablets
54118	Prednisolone 25mg/5ml oral suspension
54432	Lodotra 1mg modified-release tablets (Napp Pharmaceuticals Ltd)
54434	Prednisolone 2.5mg/5ml oral suspension
54793	Dexamethasone 2mg/5ml oral suspension
54794	Hydrocortisone 20mg modified-release tablets
55024	Prednisolone 5mg/5ml oral solution
55401	Dexamethasone 500microgram tablets (A A H Pharmaceuticals Ltd)
55480	Prednisolone 2.5mg gastro-resistant tablets (Alliance Pharmaceuticals Ltd)
56347	Dexamethasone 5mg/5ml oral solution

56891	Prednisolone 1mg tablets (Waymade Healthcare Plc)
57931	Hydrocortisone 20mg tablets (Teva UK Ltd)
58000	Prednisolone 5mg tablets (Almus Pharmaceuticals Ltd)
58061	Prednisone 50mg tablets
58234	Prednisolone 10mg/5ml oral solution
58369	Prednisolone 5mg tablets (Boston Healthcare Ltd)
58384	Prednisolone 1mg tablets (Almus Pharmaceuticals Ltd)
58474	Dexamethasone 2mg/5ml oral solution sugar free (A A H Pharmaceuticals Ltd)
58987	Prednisolone 5mg gastro-resistant tablets (Phoenix Healthcare Distribution Ltd)
59338	Prednisolone 1mg/5ml oral solution
59912	Prednisolone 5mg gastro-resistant tablets (Waymade Healthcare Plc)
60120	Dexamethasone 2mg tablets (Alliance Healthcare (Distribution) Ltd)
60421	Prednisolone 5mg tablets (Co-Pharma Ltd)
60946	Entocort CR 3mg capsules (Waymade Healthcare Plc)
61132	Prednisolone 1mg tablets (Boston Healthcare Ltd)
61162	Prednisolone 5mg tablets (Waymade Healthcare Plc)
61689	Prednisolone 5mg soluble tablets (A A H Pharmaceuticals Ltd)
62656	Prednisone 5mg Tablet (Hillcross Pharmaceuticals Ltd)
62909	Dexamethasone 2mg tablets (A A H Pharmaceuticals Ltd)
63066	Prednisolone 2.5mg tablets
63082	Prednisolone 20mg tablets
63138	Hydrocortisone 5mg/5ml oral solution
63172	Prednisolone 10mg tablets
63214	Prednisolone 5mg soluble tablets (Alliance Healthcare (Distribution) Ltd)
63549	Prednisolone 1mg/ml oral solution (Logixx Pharma Solutions Ltd)

Other immunosuppressants

Prodcode	Product name
58194	Abatacept 125mg/1ml solution for injection pre-filled syringes
60030	Abatacept 250mg powder for solution for infusion vials
61366	Abraxane 100mg powder for suspension for infusion vials (Celgene Ltd)
47695	Actinomycin D 500micrograms/vial sterile powder
48660	Adalimumab 40mg/0.8ml solution for injection pre-filled disposable devices
50996	Adalimumab 40mg/0.8ml solution for injection pre-filled syringes
56972	Adalimumab 40mg/0.8ml solution for injection vials
6882	adalimumab injection 40mg
63866	Adoport 2mg capsules (Sandoz Ltd)
44804	ADOPORT twice daily capsules 1mg [SANDOZ]
44640	ADOPORT twice daily capsules 500 micrograms [SANDOZ]
44641	ADOPORT twice daily capsules 5mg [SANDOZ]
26680	ADRIAMYCIN injection 10mg/vial [PHARMACIA]
27469	ADRIAMYCIN injection 50mg/vial [PHARMACIA]
37506	ADVAGRAF once daily modified release capsules 1mg [ASTELLAS]
40765	ADVAGRAF once daily modified release capsules 3mg [ASTELLAS]
39633	ADVAGRAF once daily modified release capsules 500 micrograms [ASTELLAS]
38919	ADVAGRAF once daily modified release capsules 5mg [ASTELLAS]
61854	Afatinib 20mg tablets
63638	Afinitor 2.5mg tablets (Novartis Pharmaceuticals UK Ltd)
43781	AFINITOR tablets 10mg [NOVARTIS]
42273	alemtuzumab concentrate for solution for infusion 30mg/1ml
26343	ALKERAN tablets 2mg [WELLCOME]
23270	ALKERAN tablets 5mg [WELLCOME]
36726	anakinra injection 100mg/0.67ml
18460	ARAVA tablets 100mg [AVENTIS]
16522	ARAVA tablets 10mg [AVENTIS]
17642	ARAVA tablets 20mg [AVENTIS]
25904	Arsenic 10mg/10ml solution for infusion ampoules
47789	Arzip 250mg capsules (Zentiva)
45393	ARZIP tablets 500mg [WINTHROP]
47194	Asparaginase 5000 unit/vial Powder for solution for injection (Imported)
25740	AVASTIN concentrate for solution for infusion 100mg/4ml [ROCHE]
58025	Axitinib 1mg tablets
62572	Axitinib 7mg tablets
59842	Azacitidine 100mg powder for suspension for injection vials
12339	AZAMUNE tablets 50mg [PENN]
3918	azathioprine 10 mg tab
8776	azathioprine 100 mg tab
55773	Azathioprine 10mg/5ml oral suspension

15556	azathioprine 125 mg tab
52921	Azathioprine 125mg/5ml oral suspension
53869	Azathioprine 20mg/5ml oral solution
54982	Azathioprine 20mg/5ml oral suspension
63121	Azathioprine 25mg tablets (Alliance Healthcare (Distribution) Ltd)
59006	Azathioprine 25mg tablets (Kent Pharmaceuticals Ltd)
17206	azathioprine 50 mg sus
53956	Azathioprine 50mg tablets (Almus Pharmaceuticals Ltd)
53797	Azathioprine 50mg tablets (Arrow Generics Ltd)
58654	Azathioprine 50mg tablets (Sandoz Ltd)
61160	Azathioprine 50mg tablets (Tillomed Laboratories Ltd)
51181	Azathioprine 60mg/5ml oral solution
770	azathioprine capsules
39115	azathioprine capsules 10mg
270	azathioprine injection 50mg/vial
55858	Azathioprine oral solution
22982	azathioprine oral solution 50mg/5ml
36792	azathioprine oral solution 50mg/ml
35518	azathioprine oral suspension 50mg/5ml
13320	azathioprine tablets 10mg
451	azathioprine tablets 25mg
34816	AZATHIOPRINE tablets 25mg [GEN (UK)]
32101	AZATHIOPRINE tablets 25mg [HILLCROSS]
571	azathioprine tablets 50mg
43562	AZATHIOPRINE tablets 50mg [ACTAVIS]
41670	AZATHIOPRINE tablets 50mg [CP PHARM]
34451	AZATHIOPRINE tablets 50mg [GEN (UK)]
34687	AZATHIOPRINE tablets 50mg [HILLCROSS]
29340	AZATHIOPRINE tablets 50mg [IVAX]
31215	AZATHIOPRINE tablets 50mg [KENT]
41620	AZATHIOPRINE tablets 50mg [TEVA]
37915	basiliximab powder for solution for infusion 10mg
40632	basiliximab powder for solution for infusion 20mg
57939	Bendamustine 100mg powder for solution for infusion vials
47767	Bendamustine 25mg powder for solution for infusion vials
26261	BERKAPRINE tablets 50mg [RORER]
38145	bevacizumab concentrate for solution for infusion 100mg/4ml
25851	Bleo-Kyowa 15,000unit powder for solution for injection vials (ProStrakan Ltd)
32261	Bleomycin 15,000unit powder for solution for injection vials
44740	bortezomib powder for solution for injection 3.5mg
58142	Brentuximab vedotin 50mg powder for solution for infusion vials
3874	busulfan tablets 2mg
22204	busulfan tablets 500micrograms

60444	Cabazitaxel 60mg/1.5ml solution for infusion vials
56410	Caelyx 50mg/25ml concentrate for solution for infusion vials (Janssen-Cilag Ltd)
37784	CAELYX concentrate for solution for infusion 20mg/10ml [JANSSEN]
24997	CAELYX concentrate for solution for infusion 2mg/ml [SCHERING-P]
40250	CAELYX concentrate for solution for infusion 50mg/25ml [JANSSEN]
45647	canakinumab powder for solution for injection 150mg
7340	capecitabine tablets 150mg
7341	capecitabine tablets 500mg
47852	Capexion 0.5mg capsules (Generics (UK) Ltd)
47512	Capexion 1mg capsules (Generics (UK) Ltd)
47984	Capexion 5mg capsules (Generics (UK) Ltd)
47102	Capimune 50mg capsules (Generics (UK) Ltd)
46395	CAPIMUNE capsules 100mg [GEN (UK)]
46637	CAPIMUNE capsules 25mg [GEN (UK)]
47192	Capsorin 100mg capsules (Morningside Healthcare Ltd)
47471	Capsorin 25mg capsules (Morningside Healthcare Ltd)
47047	Capsorin 50mg capsules (Morningside Healthcare Ltd)
61065	Carboplatin 450mg/45ml solution for infusion vials
10328	carboplatin concentrate for solution for infusion 10mg/ml
40781	carboplatin concentrate for solution for infusion 150mg/15ml
35855	carboplatin concentrate for solution for infusion 50mg/5ml
39450	carboplatin concentrate for solution for infusion 600mg/60ml
24096	carboplatin injection 150mg
18236	carboplatin injection 50mg/vial
41281	carmustine implant 7.7mg
20721	CCNU
25848	CCNU capsules 10mg [LUNDBECK]
8404	CCNU capsules 40mg [LUNDBECK]
52488	CellCept 250mg capsules (Lexon (UK) Ltd)
57593	CellCept 500mg tablets (Waymade Healthcare Plc)
16919	CELLCEPT capsules 250mg [ROCHE]
21732	CELLCEPT oral suspension 1g/5ml [ROCHE]
30581	CELLCEPT powder for concentrate for solution for infusion 500mg [ROCHE]
18804	CELLCEPT tablets 500mg [ROCHE]
45035	Ceplene 0.5mg/0.5ml solution for injection vials (Meda Pharmaceuticals Ltd)
44100	certolizumab pegol injection solution 200mg/1ml
11003	CERUBIDIN powder for concentrate for solution for injection 20mg/vial [RHONE]
38158	Cetuximab 100mg/20ml solution for infusion vials
5600	chlorambucil tablets 2mg
8665	chlorambucil tablets 5mg
28709	chlormethine injection 10mg
26119	CHLORMETHINE injection 10mg/ml [SOVEREIGN]
47042	Ciclosporin 100mg Capsule (Hillcross Pharmaceuticals Ltd)

63798	Ciclosporin 100mg capsules (A A H Pharmaceuticals Ltd)
59250	Ciclosporin 100mg capsules (Colorama Pharmaceuticals Ltd)
54975	Ciclosporin 100mg capsules (Cubic Pharmaceuticals Ltd)
48556	Ciclosporin 100mg capsules (Phoenix Healthcare Distribution Ltd)
54134	Ciclosporin 100mg capsules (Sigma Pharmaceuticals Plc)
47377	Ciclosporin 25mg Capsule (Hillcross Pharmaceuticals Ltd)
55116	Ciclosporin 25mg capsules (Cubic Pharmaceuticals Ltd)
62051	Ciclosporin 25mg capsules (Niche Pharma Ltd)
48763	Ciclosporin 25mg capsules (Phoenix Healthcare Distribution Ltd)
52743	Ciclosporin 25mg capsules (Sigma Pharmaceuticals Plc)
59249	Ciclosporin 50mg capsules (Colorama Pharmaceuticals Ltd)
54974	Ciclosporin 50mg capsules (Cubic Pharmaceuticals Ltd)
48798	Ciclosporin 50mg capsules (Phoenix Healthcare Distribution Ltd)
54867	Ciclosporin 50mg capsules (Sigma Pharmaceuticals Plc)
3896	ciclosporin capsules 100mg
16035	ciclosporin capsules 10mg
2838	ciclosporin capsules 25mg
2837	ciclosporin capsules 50mg
42924	ciclosporin concentrate for solution for infusion 250mg/5ml
38056	ciclosporin concentrate for solution for infusion 50mg/1ml
19370	ciclosporin concentrate for solution for infusion 50mg/ml
1626	ciclosporin oral solution 100mg/ml
43703	CIMZIA injection solution 200mg/1ml [UCB]
48068	Cisplatin 100mg/100ml solution for infusion vials
38453	cisplatin concentrate for solution for infusion 10mg/10ml
32824	cisplatin concentrate for solution for infusion 1mg/ml
44388	cisplatin concentrate for solution for infusion 50mg/50ml
28324	cisplatin powder 25mg/vial
38185	cisplatin powder for concentrate for solution for infusion 50mg
40454	cladribine injection 10mg/5ml
7480	cortisone acetate 25 mg inj
58496	Cosmegen Lyovac 500microgram powder for solution for injection vials (Orphan Europe (UK) Ltd)
58758	Crizotinib 200mg capsules
47752	Cyclophosphamide 25mg tablets
26322	cyclophosphamide injection 100mg
26066	cyclophosphamide injection 200mg
29840	cyclophosphamide powder for solution for injection 1000mg
16105	cyclophosphamide powder for solution for injection 500mg
3984	cyclophosphamide tablets 10mg
3985	cyclophosphamide tablets 50mg
34728	CYCLOPHOSPHAMIDE tablets 50mg [PHARMACIA]
27656	CYCLOSPORIN
29518	CYCLOSPORIN 250MG/5ML

19329	CYCLOSPORIN 250MG/5ML INJ
47522	Cytarabine 100mg/5ml solution for injection vials
41266	cytarabine injection solution 1g/10ml
47450	Cytosar 100mg Injection (Pharmacia Ltd)
19335	CYTOSAR injection 500mg [PHARMACIA]
18238	dacarbazine powder for solution for injection 100mg
28682	dacarbazine powder for solution for injection 200mg
42817	daclizumab concentrate for solution for infusion 25mg/5ml
27071	dactinomycin powder for solution for injection 500micrograms
42390	dasatinib tablets 100mg
37238	dasatinib tablets 20mg
36062	dasatinib tablets 50mg
36957	dasatinib tablets 70mg
43805	daunorubicin powder for concentrate for solution for injection 20mg/vial
13952	DECADRON injection 4mg/ml [MSD MORSON]
26454	DECADRON injection 4mg/ml [MSD MORSON]
21668	DECADRON SHOCK PAK 20mg/ml [M S D]
42449	DEXIMUNE capsules 100mg [DEXCEL]
42637	DEXIMUNE capsules 25mg [DEXCEL]
42448	DEXIMUNE capsules 50mg [DEXCEL]
47755	Docetaxel 160mg/16ml solution for infusion vials
55155	Docetaxel 20mg/2ml solution for infusion vials
36831	docetaxel concentrate for dilution for infusion solution 20mg/0.5ml
38999	docetaxel concentrate for dilution for infusion solution 80mg/2ml
33560	docetaxel concentrate for intravenous infusion 40mg/ml
44425	docetaxel concentrate for solution for infusion 20mg/1ml
37942	doxorubicin citrate liposomal complex powder for concentrate for solution for infusion 50mg
55271	Doxorubicin encapsulated in liposomes 2mg/ml concentrate solution for infusion
39307	doxorubicin injection 10mg/5ml
45026	doxorubicin injection 200mg/100ml
26947	doxorubicin injection 2mg/ml
30836	doxorubicin powder for solution for injection 10mg
32204	DTIC-DOME injection 100mg/vial [BAYER]
23832	ELOXATIN powder for concentrate for solution for infusion 100mg [SANOFI S]
61373	Enbrel 50mg/1ml solution for injection 1ml pre-filled MyClic pen (Pfizer Ltd)
49856	Enbrel 50mg/1ml solution for injection pre-filled syringes (Pfizer Ltd)
35419	ENBREL injection solution 25mg [PFIZER]
36556	ENBREL injection solution 50mg [PFIZER]
14886	ENBREL powder for solution for injection 25mg [PFIZER]
19257	ENBREL powder for solution for injection 50mg [WYETH PHAR]
44309	ENDOXANA injection 1000mg [BAXTER ONC]
44273	ENDOXANA injection 200mg [BAXTER ONC]
31193	ENDOXANA tablets 10mg [BAXTER ONC]

10729	ENDOXANA tablets 50mg [BAXTER ONC]
31539	epirubicin hydrochloride injection (powder) 20mg
39387	epirubicin hydrochloride injection 100mg/50ml
40251	epirubicin hydrochloride injection 200mg/100ml
29652	epirubicin hydrochloride injection 2mg/ml
40816	epirubicin hydrochloride injection 50mg/25ml
28325	epirubicin hydrochloride powder for solution for injection 50mg
36263	EPOSIN concentrate for solution for infusion 20mg/ml [MEDAC UK]
59276	Erbitux 100mg/20ml solution for infusion vials (Merck Serono Ltd)
62759	Eribulin 880micrograms/2ml solution for injection vials
60112	Erivedge 150mg capsules (Roche Products Ltd)
40094	Erlotinib 100mg tablets
33827	Erlotinib 150mg tablets
45992	Erlotinib 25mg tablets
38081	ERWINASE powder for solution for injection 10000 units/vial [OPI]
13604	ESTRACYT capsules 140mg [PHARMACIA]
13735	estramustine phosphate capsules 140mg
47843	Etanercept 10mg powder and solvent for solution for injection vials
56580	Etanercept 10mg powder and solvent for solution for injection vials
50494	Etanercept 50mg/1ml solution for injection 1ml pre-filled disposable devices
50998	Etanercept 50mg/1ml solution for injection pre-filled syringes
36008	etanercept injection solution 25mg
35126	etanercept injection solution 50mg
15921	etanercept powder for solution for injection 25mg
26387	etanercept powder for solution for injection 50mg
48177	Etoposide 100mg/5ml solution for infusion vials
63011	Etoposide 500mg/25ml solution for infusion vials
18751	etoposide capsules 100mg
8756	etoposide capsules 50mg
31115	etoposide concentrate for solution for infusion 20mg/ml
44387	etoposide phosphate lyophilised powder for injection 100mg
55683	Everolimus 10mg tablets
46070	everolimus tablets 5mg
29743	FLUDARA ORAL tablets 10mg [GENZYME]
24681	fludarabine powder for solution for injection 50mg
18476	fludarabine tablets 10mg
58240	Fluorouracil 2.5g/100ml solution for infusion vials
55091	Fluorouracil 500mg/10ml solution for injection vials
18070	fluorouracil capsules 250mg
19556	FLUORO-URACIL capsules 250mg [CAMBRIDGE]
39388	fluorouracil injection 1g/20ml
20229	FLUORO-URACIL injection 25mg/ml [CAMBRIDGE]
36575	fluorouracil injection 50mg/ml

41077	Gefitinib 250mg tablets
40780	gemcitabine powder for solution for infusion 1g/vial
33418	gemcitabine powder for solution for infusion 200mg/vial
21295	GLIVEC capsules 100mg [NOVARTIS]
33823	GLIVEC tablets 100mg [NOVARTIS]
28800	GLIVEC tablets 400mg [NOVARTIS]
60284	Golimumab 100mg/1ml solution for injection pre-filled disposable devices
47398	Golimumab 50mg/0.5ml solution for injection pre-filled syringes
46370	golimumab pre-filled pen injection solution 50mg
27292	Herceptin 150mg powder for solution for infusion vials (Roche Products Ltd)
58819	Herceptin 600mg/5ml solution for injection vials (Roche Products Ltd)
50121	Humira 40mg/0.8ml solution for injection pre-filled disposable devices (AbbVie Ltd)
52833	Humira 40mg/0.8ml solution for injection pre-filled syringes (AbbVie Ltd)
56593	Humira 40mg/0.8ml solution for injection vials (AbbVie Ltd)
23850	HUMIRA injection 40mg [ABBOTT]
33227	HYCAMTIN powder for concentrate for solution for infusion 4mg [GLAXSK PHA]
6884	HYDREA capsules 500mg [SQUIBB]
4257	hydrocortisone 100 mg inj
3788	hydrocortisone i/v 100 mg inj
47127	Hydroxycarbamide 100mg tablets
50565	Hydroxycarbamide 300mg capsules
59007	Hydroxycarbamide 500mg capsules (A A H Pharmaceuticals Ltd)
62157	Hydroxycarbamide 500mg/5ml oral suspension
6333	hydroxycarbamide capsules 500mg
33330	HYDROXYCARBAMIDE capsules 500mg [MEDAC UK]
39548	hydroxycarbamide film coated tablets 1000mg
38319	hydroxycarbamide oral solution 500mg/5ml
3873	hydroxyurea capsules 500mg
31339	idarubicin hydrochloride capsules 10mg
31984	idarubicin hydrochloride capsules 5mg
43168	ifosfamide injection 2g/vial
21286	imatinib capsules 100mg
29229	imatinib tablets 100mg
21318	imatinib tablets 400mg
21899	IMMUNOPRIN tablets 50mg [ASHBOURNE]
1901	IMURAN 10 MG TAB
14395	IMURAN injection 50mg/vial [ASPEN EURO]
30495	IMURAN tablets 10mg [WELLCOME]
43077	IMURAN tablets 25mg [ASPEN EURO]
671	IMURAN tablets 25mg [WELLCOME]
42988	IMURAN tablets 50mg [ASPEN EURO]
1899	IMURAN tablets 50mg [WELLCOME]
16822	infliximab powder for concentrate for solution for infusion 100mg

58862	Ipilimumab 200mg/40ml solution for infusion vials
56041	Ipilimumab 50mg/10ml solution for infusion vials
46563	Iressa 250mg tablets (AstraZeneca UK Ltd)
59339	Irinotecan 40mg/2ml concentrate for solution for infusion vials (Hospira UK Ltd)
40983	irinotecan hydrochloride concentrate for solution for infusion 40mg/2ml
32418	KINERET injection 100mg/0.67ml [AMGEN]
26502	LANVIS tablets 40mg [ALKOPHARMA]
46837	Lapatinib 250mg tablets
48217	Leflunomide 10mg tablets (medac UK)
62007	Leflunomide 15mg tablets
62993	Leflunomide 20mg tablets (Sandoz Ltd)
4970	leflunomide tablets 100mg
4971	leflunomide tablets 10mg
6934	leflunomide tablets 20mg
60630	Lenalidomide 2.5mg capsules
44529	lenalidomide capsules 10mg
46205	lenalidomide capsules 15mg
40626	lenalidomide capsules 25mg
42056	lenalidomide capsules 5mg
16838	LEUKERAN tablets 2mg [WELLCOME]
26315	LEUKERAN tablets 5mg [WELLCOME]
12067	lomustine capsules 40mg
44478	LYSODREN tablets 500mg [LAB HRA]
55815	MabCampath 30mg/1ml concentrate for solution for infusion vials (Genzyme Therapeutics Ltd)
31598	MABCAMPATH concentrate for solution for infusion 10mg/ml [SCHERING]
47502	MabThera 100mg/10ml concentrate for solution for infusion vials (Roche Products Ltd)
27342	MAXTREX injection 2.5mg/ml [PHARMACIA]
21753	MAXTREX tablets 10mg [PHARMACIA]
13428	MAXTREX tablets 2.5mg [PHARMACIA]
26580	melphalan injection 100mg/vial
37099	melphalan powder for solution for injection 50mg
16929	melphalan tablets 2mg
12150	melphalan tablets 5mg
57239	Mercaptopurine 20mg/ml oral suspension
56753	Mercaptopurine 25mg tablets
55772	Mercaptopurine 25mg/5ml oral suspension
61545	Mercaptopurine 50mg tablets (Aspen Pharma Trading Ltd)
52333	Mercaptopurine 75mg/5ml oral suspension
19982	mercaptopurine capsules 10mg
47369	Mercaptopurine Oral solution
32972	mercaptopurine tablets 10mg
3450	mercaptopurine tablets 50mg
27579	methotrexate

57441	Methotrexate 10mg tablets (A A H Pharmaceuticals Ltd)
58885	Methotrexate 10mg tablets (Sigma Pharmaceuticals Plc)
59538	Methotrexate 10mg tablets (Teva UK Ltd)
57174	Methotrexate 10mg tablets (Waymade Healthcare Plc)
61151	Methotrexate 10mg/0.2ml solution for injection pre-filled disposable devices
61081	Methotrexate 12.5mg/0.25ml solution for injection pre-filled disposable devices
61273	Methotrexate 15mg/0.3ml solution for injection pre-filled disposable devices
61137	Methotrexate 17.5mg/0.35ml solution for injection pre-filled disposable devices
56037	Methotrexate 2.5mg tablets (A A H Pharmaceuticals Ltd)
51120	Methotrexate 2.5mg tablets (Alliance Healthcare (Distribution) Ltd)
62833	Methotrexate 2.5mg tablets (DE Pharmaceuticals)
60979	Methotrexate 2.5mg tablets (Morningside Healthcare Ltd)
58303	Methotrexate 2.5mg tablets (Orion Pharma (UK) Ltd)
49951	Methotrexate 2.5mg tablets (Sandoz Ltd)
52606	Methotrexate 2.5mg tablets (Sigma Pharmaceuticals Plc)
59685	Methotrexate 2.5mg tablets (Teva UK Ltd)
53385	Methotrexate 2.5mg tablets (Waymade Healthcare Plc)
61085	Methotrexate 2.5mg tablets (Waymade Healthcare Plc)
51667	Methotrexate 200mg/8ml solution for injection vials
61140	Methotrexate 20mg/0.4ml solution for injection pre-filled disposable devices
61082	Methotrexate 22.5mg/0.45ml solution for injection pre-filled disposable devices
61122	Methotrexate 25mg/0.5ml solution for injection pre-filled disposable devices
21889	methotrexate 25mg/1ml
62753	Methotrexate 27.5mg/0.55ml solution for injection pre-filled syringes
61796	Methotrexate 30mg/0.6ml solution for injection pre-filled disposable devices
51321	Methotrexate 50mg/2ml solution for injection vials
53696	Methotrexate 50mg/2ml solution for injection vials (A A H Pharmaceuticals Ltd)
49547	Methotrexate 5g/200ml solution for infusion vials
61172	Methotrexate 7.5mg/0.15ml solution for injection pre-filled disposable devices
59723	Methotrexate 7.5mg/5ml oral solution
36167	methotrexate injection 1000mg/10ml
46407	methotrexate injection 1000mg/40ml
12816	methotrexate injection 100mg/ml
40371	methotrexate injection 10mg/0.2ml
7337	methotrexate injection 10mg/0.4ml
32865	methotrexate injection 10mg/1ml
46152	methotrexate injection 12.5mg/0.25ml
7336	methotrexate injection 12.5mg/0.5ml
40281	methotrexate injection 15mg/0.3ml
16540	methotrexate injection 15mg/0.6ml
27404	methotrexate injection 15mg/1.5ml
46156	methotrexate injection 17.5mg/0.35ml
18890	methotrexate injection 17.5mg/0.7ml

40273	methotrexate injection 20mg/0.4ml
14347	methotrexate injection 20mg/0.8ml
34258	METHOTREXATE injection 20mg/0.8ml [CENT HOME]
45165	methotrexate injection 20mg/1ml
26064	methotrexate injection 20mg/2ml
46129	methotrexate injection 22.5mg/0.45ml
17672	methotrexate injection 22.5mg/0.9ml
40328	methotrexate injection 25mg/0.5ml
45558	methotrexate injection 25mg/1.25ml
16519	methotrexate injection 25mg/1ml
24634	methotrexate injection 25mg/2.5ml
8583	methotrexate injection 25mg/ml
27642	methotrexate injection 27.5mg/1.1ml
44908	methotrexate injection 30mg/0.6ml
30703	methotrexate injection 30mg/1.2ml
46039	methotrexate injection 30mg/1.5ml
41086	methotrexate injection 5000mg/50ml
32229	methotrexate injection 500mg/20ml
24783	methotrexate injection 50mg/2ml
8327	methotrexate injection 50mg/3ml
30932	methotrexate injection 5mg/0.2ml
9528	methotrexate injection 5mg/2ml
40301	methotrexate injection 7.5mg/0.15ml
16570	methotrexate injection 7.5mg/0.3ml
35402	methotrexate injection 7.5mg/0.75ml
36800	methotrexate oral solution 10mg/5ml
36849	methotrexate oral suspension 10mg/5ml
28041	methotrexate oral suspension 12.5mg/5ml
35752	methotrexate oral suspension 7.5mg/5ml
14748	methotrexate sodium injection 25mg/ml
18424	methotrexate sodium tablets 2.5mg
41585	METHOTREXATE SODIUM tablets 2.5mg [WYETH PHAR]
29069	methotrexate sterile powder 500mg/vial
17035	methotrexate suspension 2.5mg/5ml
877	methotrexate tablets 10mg
34929	METHOTREXATE tablets 10mg [HOSPIRA]
823	methotrexate tablets 2.5mg
41104	METHOTREXATE tablets 2.5mg [CP PHARM]
20951	METHOTREXATE tablets 2.5mg [GOLDSHIELD]
32111	METHOTREXATE tablets 2.5mg [HOSPIRA]
30780	METHOTREXATE tablets 2.5mg [PHARMACIA]
9117	methylprednisolone 40 mg/ml inj
2181	methylprednisolone/lignocaine hcl (1ml) 40 mg/ml inj

57103	Metoject 27.5mg/0.55ml solution for injection pre-filled syringes (medac UK)
50950	Metoject 30mg/0.6ml solution for injection pre-filled syringes (medac UK)
40356	METOJECT injection 10mg/0.2ml [MEDAC UK]
37117	METOJECT injection 10mg/1ml [MEDAC UK]
46098	METOJECT injection 12.5mg/0.25ml [MEDAC UK]
40284	METOJECT injection 15mg/0.3ml [MEDAC UK]
27400	METOJECT injection 15mg/1.5ml [MEDAC UK]
46265	METOJECT injection 17.5mg/0.35ml [MEDAC UK]
40292	METOJECT injection 20mg/0.4ml [MEDAC UK]
14348	METOJECT injection 20mg/2ml [MEDAC UK]
46197	METOJECT injection 22.5mg/0.45ml [MEDAC UK]
40293	METOJECT injection 25mg/0.5ml [MEDAC UK]
33601	METOJECT injection 25mg/2.5ml [MEDAC UK]
40280	METOJECT injection 7.5mg/0.15ml [MEDAC UK]
35865	METOJECT injection 7.5mg/0.75ml [MEDAC UK]
61050	Metoject PEN 10mg/0.2ml solution for injection pre-filled pen (medac UK)
61211	Metoject PEN 12.5mg/0.25ml solution for injection pre-filled pen (medac UK)
61171	Metoject PEN 15mg/0.3ml solution for injection pre-filled pen (medac UK)
61181	Metoject PEN 17.5mg/0.35ml solution for injection pre-filled pen (medac UK)
61180	Metoject PEN 20mg/0.4ml solution for injection pre-filled pen (medac UK)
61419	Metoject PEN 22.5mg/0.45ml solution for injection pre-filled pen (medac UK)
61169	Metoject PEN 25mg/0.5ml solution for injection pre-filled pen (medac UK)
62421	Metoject PEN 27.5mg/0.55ml solution for injection pre-filled pen (medac UK)
61488	Metoject PEN 30mg/0.6ml solution for injection pre-filled pen (medac UK)
61178	Metoject PEN 7.5mg/0.15ml solution for injection pre-filled pen (medac UK)
30014	MITHRACIN injection 2.5mg/vial [PFIZER]
31494	mithramycin injection 2.5mg/vial
30757	mitobronitol (named patient only) 125 mg tab
113	Mitomycin 10mg powder for solution for injection vials
61458	Mitomycin 20mg powder for solution for injection vials
19287	Mitomycin 2mg powder for solution for injection vials
32593	Mitomycin 40mg powder for solution for injection vials
44594	Mitomycin-C Kyowa 10mg powder for solution for injection vials (ProStrakan Ltd)
30051	Mitomycin-C Kyowa 2mg powder for solution for injection vials (ProStrakan Ltd)
48007	Mitomycin-C Kyowa 40mg powder for solution for injection vials (ProStrakan Ltd)
35826	mitotane tablets 500mg
39366	mitoxantrone concentrate for solution for infusion 10mg/5ml
41267	mitoxantrone concentrate for solution for infusion 20mg/10ml
33174	mitoxantrone concentrate for solution for infusion 2mg/ml
46325	MODIGRAF granules for oral suspension 1mg [ASTELLAS]
46324	MODIGRAF granules for oral suspension 200micrograms [ASTELLAS]
57272	Mycophenolate mofetil 125mg/5ml oral suspension
60231	Mycophenolate mofetil 250mg capsules (A A H Pharmaceuticals Ltd)

54317	Mycophenolate mofetil 250mg capsules (Sandoz Ltd)
53255	Mycophenolate mofetil 250mg capsules (Sigma Pharmaceuticals Plc)
58530	Mycophenolate mofetil 500mg tablets (Sigma Pharmaceuticals Plc)
4438	mycophenolate mofetil capsules 250mg
16879	mycophenolate mofetil oral suspension 1g/5ml
7077	mycophenolate mofetil powder for concentrate for solution for infusion 500mg
4230	mycophenolate mofetil tablets 500mg
50669	Mycophenolate motefil 500mg tablets (Sandoz Ltd)
47746	Mycophenolate motefil 500mg tablets (Wockhardt UK Ltd)
35301	mycophenolic acid gastro-resistant tablets 180mg
26097	mycophenolic acid gastro-resistant tablets 360mg
37395	MYELOBROMOL (NAMED PATIENT ONLY) 125 MG TAB
37396	MYELOBROMOL tablets 125mg [DURBIN]
45489	MYFENAX capsules 250mg [TEVA]
45043	MYFENAX tablets 500mg [TEVA]
27290	MYFORTIC tablets 180mg [NOVARTIS]
27289	MYFORTIC tablets 360mg [NOVARTIS]
26301	MYLERAN tablets 2mg [WELLCOME]
32412	MYLERAN tablets 500micrograms [WELLCOME]
33878	MYOCET powder for concentrate for solution for infusion 50mg [CEPHALON]
41191	natalizumab concentrate for solution for infusion 300mg/15ml
28605	NATULAN capsules 50mg [CAMBRIDGE]
52615	Neoral 100mg capsules (Sigma Pharmaceuticals Plc)
49958	Neoral 25mg capsules (DE Pharmaceuticals)
53175	Neoral 25mg capsules (Mawdsley-Brooks & Company Ltd)
53176	Neoral 50mg capsules (DE Pharmaceuticals)
973	NEORAL capsules 100mg [NOVARTIS]
16137	NEORAL capsules 10mg [NOVARTIS]
972	NEORAL capsules 25mg [NOVARTIS]
4231	NEORAL capsules 50mg [NOVARTIS]
1905	NEORAL oral solution 100mg/ml [NOVARTIS]
42362	Nexavar 200mg tablets (Bayer Plc)
45820	nilotinib capsules 150mg
38317	nilotinib capsules 200mg
15405	NOVANTRONE concentrate for solution for infusion 2mg/ml [WYETH PHAR]
60707	Ofatumumab 100mg/5ml solution for infusion vials
27224	Oncovin 2mg Injection (Eli Lilly and Company Ltd)
19072	OPRISINE tablets 50mg [OPUS]
50277	Oxaliplatin 100mg/20ml concentrate for solution for infusion vials (A A H Pharmaceuticals Ltd)
58942	Oxaliplatin 200mg/40ml concentrate for solution for infusion vials (Accord Healthcare Ltd)
39895	oxaliplatin concentrate for solution for infusion 100mg/20ml
39553	oxaliplatin concentrate for solution for infusion 50mg/10ml
36714	oxaliplatin powder for concentrate for solution for infusion 100mg

27293	oxaliplatin powder for concentrate for solution for infusion 50mg
47754	Paclitaxel 100mg/16.7ml solution for infusion vials
39919	paclitaxel albumin bound powder for suspension for infusion 100mg
45147	paclitaxel concentrate for solution for infusion 150mg/25ml
35854	paclitaxel concentrate for solution for infusion 30mg/5ml
14381	paclitaxel concentrate for solution for infusion 6mg/ml
45122	Panitumumab 100mg/5ml solution for infusion vials
41963	PARAPLATIN concentrate for solution for infusion 10mg/ml [BRISTOL]
37542	PARAPLATIN injection 150mg [BRISTOL]
47552	Pazopanib 200mg tablets
46823	Pazopanib 400mg tablets
37272	pemetrexed powder for concentrate for solution for infusion 500mg
43888	pentostatin powder for solution for injection 10mg
61908	Pertuzumab 420mg/14ml solution for infusion vials
28889	PHARMORUBICIN injection solution 2mg/ml [PHARMACIA]
43639	PHARMORUBICIN powder for solution for injection 50mg [PHARMACIA]
59048	Pomalidomide 2mg capsules
8306	Prednisolone 25mg/1ml suspension for injection ampoules
24657	prednisolone pivalate 50 mg inj
27428	prednisolone sodium phosphate 16 mg inj
17186	procarbazine capsules 50mg
52993	Prograf 1mg capsules (DE Pharmaceuticals)
54198	Prograf 1mg capsules (Lexon (UK) Ltd)
51184	Prograf 1mg capsules (Necessity Supplies Ltd)
51185	Prograf 500microgram capsules (Necessity Supplies Ltd)
3683	PROGRAF twice daily capsules 1mg [ASTELLAS]
5870	PROGRAF twice daily capsules 500 micrograms [ASTELLAS]
13271	PROGRAF twice daily capsules 5mg [ASTELLAS]
29675	PURI-NETHOL tablets 50mg [ALKOPHARMA]
44222	raltitrexed powder for concentrate for solution for infusion 2mg
63210	Rapamune 0.5mg tablets (Pfizer Ltd)
33728	RAPAMUNE oral solution 1mg/ml [PFIZER]
23289	RAPAMUNE tablets 1mg [PFIZER]
28999	RAPAMUNE tablets 2mg [PFIZER]
22640	razoxane tablets 125mg
12066	RAZOXIN tablets 125mg [CAMBRIDGE]
22392	REMICADE powder for concentrate for solution for infusion 100mg [SCHERING-P]
58227	Revlimid 10mg capsules (Celgene Ltd)
56818	Revlimid 15mg capsules (Celgene Ltd)
41139	REVLIMID capsules 25mg [CELGENE]
39111	rituximab concentrate for intravenous infusion 10mg/ml
28490	rituximab concentrate for solution for infusion 100mg/10ml
36294	rituximab concentrate for solution for infusion 500mg/50ml

56962	RoActemra 200mg/10ml concentrate for solution for infusion vials (Roche Products Ltd)
59434	Ruxolitinib 15mg tablets
60008	Ruxolitinib 20mg tablets
13556	SANDIMMUN capsules 100mg [NOVARTIS]
3920	SANDIMMUN capsules 25mg [NOVARTIS]
15596	SANDIMMUN capsules 50mg [NOVARTIS]
26790	SANDIMMUN concentrate for solution for infusion 50mg/ml [NOVARTIS]
13494	SANDIMMUN sugar free solution 100mg/ml [NOVARTIS]
62618	Simponi 100mg/1ml solution for injection pre-filled pen (Merck Sharp & Dohme Ltd)
47740	Simponi 50mg/0.5ml solution for injection pre-filled disposable devices (Merck Sharp & Dohme Ltd)
61351	Simulect 10mg powder and solvent for solution for injection vials (Novartis Pharmaceuticals UK Ltd)
32614	SIMULECT powder for solution for infusion 20mg [NOVARTIS]
20097	sirolimus oral solution 1mg/ml
6600	sirolimus tablets 1mg
6484	sirolimus tablets 2mg
44783	sirolimus tablets 500 micrograms
35685	Sorafenib 200mg tablets
58174	Stelara 45mg/0.5ml solution for injection pre-filled syringes (Janssen-Cilag Ltd)
38871	Sunitinib 12.5mg capsules
44063	Sunitinib 25mg capsules
40580	Sunitinib 50mg capsules
39795	Sutent 12.5mg capsules (Pfizer Ltd)
42993	Sutent 25mg capsules (Pfizer Ltd)
45450	Sutent 50mg capsules (Pfizer Ltd)
47416	Tacni 0.5mg capsules (Teva UK Ltd)
47276	Tacni 1mg capsules (Teva UK Ltd)
47506	Tacni 5mg capsules (Teva UK Ltd)
48339	Tacrolimus 1mg capsules (A A H Pharmaceuticals Ltd)
55066	Tacrolimus 2.5mg/5ml oral solution
51790	Tacrolimus 500microgram capsules (A A H Pharmaceuticals Ltd)
54048	Tacrolimus 500micrograms/5ml oral suspension
47432	Tacrolimus 5mg/5ml oral suspension
63720	Tacrolimus 750microgram capsules
33123	tacrolimus concentrate for solution for infusion 5mg/1ml
43081	tacrolimus granules for oral suspension 1mg
43082	tacrolimus granules for oral suspension 200micrograms
37985	tacrolimus once daily modified release capsules 1mg
40964	tacrolimus once daily modified release capsules 3mg
38113	tacrolimus once daily modified release capsules 500 micrograms
38989	tacrolimus once daily modified release capsules 5mg
44926	tacrolimus oral suspension 2.5mg/5ml
37155	tacrolimus suspension 1mg/ml
2839	tacrolimus twice daily capsules 1mg

6495	tacrolimus twice daily capsules 500 micrograms
5089	tacrolimus twice daily capsules 5mg
63202	Tafinlar 75mg capsules (GlaxoSmithKline UK Ltd)
47556	Tarceva 100mg tablets (Roche Products Ltd)
25262	Tarceva 150mg tablets (Roche Products Ltd)
37441	Tarceva 25mg tablets (Roche Products Ltd)
40379	Targretin 75mg capsules (Eisai Ltd)
35384	TAXOL concentrate for solution for infusion 30mg/5ml [BMS]
16173	TAXOL concentrate for solution for infusion 6mg/ml [BMS]
47571	Taxotere 160mg/8ml concentrate for solution for infusion vials (Sanofi)
23849	TAXOTERE concentrate for intravenous infusion 40mg/ml [AVENTIS]
36552	TAXOTERE concentrate for solution for infusion 20mg/0.5ml [AVENTIS]
44087	TAXOTERE concentrate for solution for infusion 20mg/1ml [AVENTIS]
55077	Temodal 100mg capsules (Merck Sharp & Dohme Ltd)
35226	TEMODAL capsules 20mg [SCHERING-P]
29700	TEMODAL capsules 250mg [SCHERING-P]
33803	TEMODAL capsules 5mg [SCHERING-P]
21249	temozolomide capsules 100mg
42372	temozolomide capsules 140mg
32490	temozolomide capsules 20mg
21250	temozolomide capsules 250mg
27922	temozolomide capsules 5mg
62880	Teriflunomide 14mg tablets
26332	thiotepa 15 mg inj
33385	thiotepa powder for solution for injection 15mg
42696	thiotepa powder for solution for injection 15mg
20094	tioguanine tablets 40mg
62873	Tocilizumab 162mg/0.9ml solution for injection pre-filled syringes
62957	Tocilizumab 400mg/20ml solution for infusion vials
46348	tocilizumab concentrate for solution for infusion 200mg/10ml
41502	tocilizumab concentrate for solution for infusion 80mg/4ml
47396	Topotecan 1mg capsules
41960	topotecan powder for concentrate for solution for infusion 1mg
40453	TORISEL concentrate for solution for infusion 30mg/1.2ml [PFIZER]
59362	Trabectedin 1mg powder for solution for infusion vials
62009	Trabectedin 250microgram powder for solution for infusion vials
31076	Trastuzumab 150mg powder for solution for infusion vials
24448	treosulfan capsules 250mg
23871	TREOSULFAN capsules 250mg [FARILLON]
28726	treosulfan leo
43120	Trisenox 10mg/10ml concentrate for solution for infusion ampoules (Teva UK Ltd)
38254	TYSABRI concentrate for solution for infusion 300mg/15ml [BIOGEN]
33519	UFTORAL capsules 224mg + 100mg [MERCK SER]

33520	uracil with tegafur capsules 224mg + 100mg
46309	Ustekinumab 45mg/0.5ml injection solution
54485	Ustekinumab 45mg/0.5ml solution for injection pre-filled syringes
57172	Vandetanib 100mg tablets
40732	VELCADE powder for solution for injection 3.5mg [ORTHO BIO]
58932	Vemurafenib 240mg tablets
37375	VEPESID capsules 100mg [BRISTOL]
29761	VEPESID capsules 50mg [BRISTOL]
32604	vinblastine sulphate injection 10mg/10ml
31223	vinblastine sulphate injection 10mg/vial
40649	Vincristine 1mg/1ml solution for injection vials
55823	Vincristine 5mg/5ml solution for injection vials
18914	Vincristine sulphate 1mg injection
25589	Vincristine sulphate 1mg/ml injection
26316	Vincristine sulphate 2mg injection
40659	Vincristine sulphate 5mg injection
46838	Vinorelbine 80mg capsules
32774	vinorelbine capsules 20mg
42684	vinorelbine concentrate for solution for infusion 10mg/1ml
33171	vinorelbine injection solution 10mg/ml
47240	Vivadex 0.5mg capsules (Dexcel-Pharma Ltd)
47239	Vivadex 1mg capsules (Dexcel-Pharma Ltd)
55010	Vivadex 5mg capsules (Dexcel-Pharma Ltd)
56652	Votrient 200mg tablets (GlaxoSmithKline UK Ltd)
33127	XELODA tablets 150mg [ROCHE]
18063	XELODA tablets 500mg [ROCHE]
41058	ENBREL FOR PAEDIATRIC USE powder for solution for injection 25mg [PFIZER]

Medcode	Readterm
69387	Ext.beam-surgery+chemotherapy
51787	Ext.beam + chemotherapy
70290	Radiomimetic chemotherapy
73172	Radio-chemotherapy NOS
98882	Radio-chemo.: I-V route
62864	Radio-chemo.: oral route
107130	Radio-chem.: into cavity
40490	Intrathecal chemotherapy
15386	Intravenous chemotherapy
64997	Delivery of chemotherapy for neoplasm
28071	Continuous infusion of chemotherapy
18461	Intravenous injection of Methotrexate

37123	Intramuscular chemotherapy
17181	Intramuscular injection of Methotrexate
94912	Subcutaneous injection of methotrexate
30264	Subcutaneous chemotherapy
19467	Pre-operative chemotherapy
22490	Combined pre-operative chemotherapy and radiotherapy
95424	Delivery of chemotherapy for neoplasm NOS
783	Chemotherapy
101693	Cancer chemotherapy management plan
104142	Neoadjuvant chemotherapy
14887	Oral chemotherapy
18675	Post-operartive chemotherapy
94431	Delivery of oral chemotherapy for neoplasm NOS
107734	Deliver simple parenteral chemother neoplas first attendance
51959	Ambulatory chemotherapy
28809	Combined post-operative chemotherapy and radiotherapy
46028	Date chemotherapy stopped
5019	Cancer chemotherapy
35609	[V]Convalescence after chemotherapy
91778	Del comp chemo neo inc prolong infusion treat first attend
51781	[X]Other chemotherapy
90743	Delivery of oral chemotherapy for neoplasm
96310	Other specified delivery of chemotherapy for neoplasm
31489	[V]Chemotherapy session for neoplasm
89452	Procurement drugs for chemotherapy for neoplasm in bands 1-5
26149	Chemotherapy started
18832	Date chemotherapy completed
20381	[V]Maintenance chemotherapy
48547	High cost immunosuppressant drugs
105256	Immunosuppressive therapy
102559	Immunosuppressant drug therapy
103417	High cost immunosuppressant drugs NOS
102571	Anti-tumour necrosis factor drug monitoring

opcs	description
X352	Intravenous chemotherapy
X373	Intramuscular chemotherapy
X384	Subcutaneous chemotherapy
X70	Procurement of drugs for chemotherapy for neoplasm in Bands 1-5
X701	Procurement of drugs for chemotherapy for neoplasm for regimens in Band 1
X702	Procurement of drugs for chemotherapy for neoplasm for regimens in Band 2

X703	Procurement of drugs for chemotherapy for neoplasm for regimens in Band 3
X704	Procurement of drugs for chemotherapy for neoplasm for regimens in Band 4
X705	Procurement of drugs for chemotherapy for neoplasm for regimens in Band 5
X708	Other specified procurement of drugs for chemotherapy for neoplasm in Bands 1-5
X709	Unspecified procurement of drugs for chemotherapy for neoplasm in Bands 1-5
X71	Procurement of drugs for chemotherapy for neoplasm in Bands 6-10
X711	Procurement of drugs for chemotherapy for neoplasm for regimens in Band 6
X712	Procurement of drugs for chemotherapy for neoplasm for regimens in Band 7
X713	Procurement of drugs for chemotherapy for neoplasm for regimens in Band 8
X714	Procurement of drugs for chemotherapy for neoplasm for regimens in Band 9
X715	Procurement of drugs for chemotherapy for neoplasm for regimens in Band 10
X718	Other specified procurement of drugs for chemotherapy for neoplasm in Bands 6-10
X719	Unspecified procurement of drugs for chemotherapy for neoplasm in Bands 6-10
X72	Delivery of chemotherapy for neoplasm
X721	Delivery of complex chemotherapy for neoplasm including prolonged infusional treatment at first attendance
X722	Delivery of complex parenteral chemotherapy for neoplasm at first attendance
X723	Delivery of simple parenteral chemotherapy for neoplasm at first attendance
X724	Delivery of subsequent element of cycle of chemotherapy for neoplasm
X728	Other specified delivery of chemotherapy for neoplasm
X729	Unspecified delivery of chemotherapy for neoplasm
X73	Delivery of oral chemotherapy for neoplasm
X731	Delivery of exclusively oral chemotherapy for neoplasm
X738	Other specified delivery of oral chemotherapy for neoplasm
X739	Unspecified delivery of oral chemotherapy for neoplasm

Solid organ transplant

Medcode	Readterm
242	Allotransplantation of heart NEC
250	Transplantation of heart and lung
2124	Transplant follow-up
4405	Transplantation of liver
4438	Other transplantation of heart
6692	Liver transplant failure and rejection
9026	[V]Liver transplanted
9384	[V]Heart transplanted
10394	[V]Lung transplanted
10461	Transplantation of lung
11113	Transplanted organ rejection
25896	Transplanted organ complication NOS
27319	Transplantation of liver NOS
27679	Heart-lung transplant failure and rejection
29831	Transplanted organ failure
31997	Liver transplant with complication, without blame
32025	Orthotopic transplantation of liver
35368	Transplantation of pancreas
36960	[V]Heart and lungs transplant status
38011	Transplantation of lung NOS
41495	Other transplantation of heart NOS
44077	[V]Pancreas transplanted
44893	Transplanted organ complication
47484	Heart transplant failure and rejection
47861	Exploration of liver transplant
53626	Allotransplantation of heart and lung
56993	Transplantation of whole pancreas
59610	Transplant immunosuppression
61073	Transplantation of heart and lung NOS
64438	Heart transplant with complication, without blame
67499	Transplantation of pancreas NOS
69194	Replacement of previous liver transplant
69734	Other specified other transplantation of heart
71422	Heterotopic transplantation of liver
72939	Xenotransplantation of heart
73743	Other specified transplantation of lung
89445	Auxillary liver transplant
93713	Single lung transplant
93751	[V]Intestine transplanted
93844	Revision of transplantation of heart NEC

96129	Excision of transplanted pancreas
96423	[V]Transplanted organ
96578	Double lung transplant
97157	Orthotopic transplantation of liver NEC
99250	Other specified transplantation of liver
100073	Piggy back liver transplant
100621	Transplantation of ileum
101231	Transplantation of tail of pancreas
102998	Transplantation of thymus gland
105506	Orthotopic transplantation of whole liver
106015	Transplantation of spleen
107416	Piggyback transplantation of heart
108330	Complication of transplanted lung
109304	Cardiothoracic transplantation

icd	icd_description
T86.2	Heart transplant failure and rejection
T86.3	Heart-lung transplant failure and rejection
T86.4	Liver transplant failure and rejection
Y83.0	Surgical operation with transplant of whole organ
Z94.0	Kidney transplant status
Z94.1	Heart transplant status
Z94.2	Lung transplant status
Z94.3	Heart and lungs transplant status
Z94.4	Liver transplant status

opcs	description
B17	Transplantation of thymus gland
B171	Allotransplantation of thymus gland
B178	Other specified transplantation of thymus gland
B179	Unspecified transplantation of thymus gland
E53	Transplantation of lung
E531	Double lung transplant
E532	Single lung transplant
E533	Single lobe lung transplant
E538	Other specified transplantation of lung
E539	Unspecified transplantation of lung
G68	Transplantation of ileum
G681	Allotransplantation of ileum
G688	Other specified transplantation of ileum

G689	Unspecified transplantation of ileum
J01	Transplantation of liver
J011	Orthotopic transplantation of liver NEC
J012	Heterotopic transplantation of liver
J013	Replacement of previous liver transplant
J014	Transplantation of liver cells
J015	Orthotopic transplantation of whole liver
J018	Other specified transplantation of liver
J019	Unspecified transplantation of liver
J54	Transplantation of pancreas
J541	Transplantation of pancreas and duodenum
J542	Transplantation of whole pancreas
J543	Transplantation of tail of pancreas
J544	Transplantation of islet of Langerhans
J545	Renewal of transplanted pancreatic tissue
J548	Other specified transplantation of pancreas
J549	Unspecified transplantation of pancreas
J553	Excision of transplanted pancreas
J721	Transplantation of spleen
K01	Transplantation of heart and lung
K011	Allotransplantation of heart and lung
K012	Revision of transplantation of heart and lung
K018	Other specified transplantation of heart and lung
K019	Unspecified transplantation of heart and lung
K02	Other transplantation of heart
K021	Allotransplantation of heart NEC
K022	Xenotransplantation of heart
K023	Implantation of prosthetic heart
K024	Piggyback transplantation of heart
K025	Revision of implantation of prosthetic heart
K026	Revision of transplantation of heart NEC
K028	Other specified other transplantation of heart
K029	Unspecified other transplantation of heart

Alcohol use

Medcode	Readterm	Status
322	Moderate drinker - 3-6u/day	Current
385	Drinks rarely	Current
669	Nondependent alcohol abuse, unspecified	Current
749	Drinks occasionally	Current
956	Social drinker	Current
967	Stopped drinking alcohol	Ex
1399	Alcohol problem drinking	Current
1476	Delirium tremens	Current
1618	Heavy drinker - 7-9u/day	Current
2081	Alcoholism	Current
2082	Alcohol withdrawal syndrome	Current
2083	Alcohol detoxification	Current
2084	Alcohol dependence syndrome	Current
2689	Beer drinker	Current
2925	Alcoholic polyneuropathy	Current
3216	Acute alcoholic hepatitis	Current
3782	Intoxication - alcohol	Current
4447	Non-drinker alcohol	Non
4500	Korsakov's alcoholic psychosis	Current
4506	Alcoholic gastritis	Current
4743	Alcoholic cirrhosis of liver	Current
4915	Alcoholic cardiomyopathy	Current
5611	[X]Mental and behavioural disorders due to use of alcohol	Current
5740	Acute alcoholic intoxication in alcoholism	Current
5758	[X]Chronic alcoholism	Current
6169	Alcohol dependence syndrome NOS	Current
6467	[X]Alcoholic hallucinosis	Current
7123	[V]Personal history of alcoholism	Ex
7545	[V] Alcohol use	Current
7602	Chronic alcoholic hepatitis	Current
7692	Patient advised about alcohol	Current
7746	Nondependent alcohol abuse	Current
7885	Alcoholic liver damage unspecified	Current
7943	Alcoholic hepatitis	Current
8030	[V]Alcohol abuse counselling and surveillance	Current
8363	Oesophageal varices in alcoholic cirrhosis of the liver	Current
8388	[V]Alcohol rehabilitation	Current
8999	Heavy drinker	Current
9169	[D]Alcohol blood level excessive	Current
9489	Under care of community alcohol team	Current

9508	[X]Acute alcoholic drunkenness	Current
9849	Referral to community alcohol team	Current
10161	O/E - alcoholic breath	Current
10691	Alcoholic fatty liver	Current
11106	Korsakov's alcoholic psychosis with peripheral neuritis	Current
11491	Health ed. - alcohol	Current
11670	[X]Korsakov's psychosis, alcohol induced	Current
11740	Alcohol misuse - enhanced services administration	Current
12353	[X]Mental & behav dis due to use alcohol: psychotic disorder	Current
12554	Referral to community drug and alcohol team	Current
12949	Teetotaller	Non
12968	Drinks beer and spirits	Current
12969	Drinks wine	Current
12970	Non drinker alcohol	Non
12971	Spirit drinker	Current
12972	Light drinker - 1-2u/day	Current
12974	Nondependent alcohol abuse, episodic	Current
12975	Trivial drinker - <1u/day	Current
12976	Suspect alcohol abuse - denied	Current
12977	Very heavy drinker - >9u/day	Current
12979	Current non drinker	Non
12980	Light drinker	Current
12982	Alcohol intake above recommended sensible limits	Current
12983	Ex-very heavy drinker-(>9u/d)	Ex
12984	Very heavy drinker	Current
12985	Moderate drinker	Current
16225	Alcohol withdrawal delirium	Current
16237	Alcoholic psychoses	Current
16587	[V]Problems related to lifestyle alcohol use	Current
17259	[X]Delirium tremens, alcohol induced	Current
17330	Alcoholic hepatic failure	Current
17607	[X]Alcoholic psychosis NOS	Current
18156	Alcoholics anonymous	Current
18636	Wernicke-Korsakov syndrome	Current
18711	Lifestyle advice regarding alcohol	Current
19217	Alcohol causing toxic effect	Current
19401	Binge drinker	Current
19493	Ex-heavy drinker - (7-9u/day)	Ex
19494	Hazardous alcohol use	Current
19495	Ex-moderate drinker - (3-6u/d)	Ex
20407	Drunkenness - pathological	Current
20514	[X]Mental and behav dis due to use alcohol: withdrawal state	Current
20762	Alcohol amnestic syndrome	Current

21624	Episodic acute alcoholic intoxication in alcoholism	Current
21650	Admitted to alcohol detoxification centre	Current
21713	Alcoholic fibrosis and sclerosis of liver	Current
21879	[X]Mental and behav dis due to use of alcohol: harmful use	Current
22277	DTs - delirium tremens	Current
22707	Drinking problem scale	Current
22933	Ex-trivial drinker (<1u/day)	Ex
23610	Nondependent alcohol abuse, continuous	Current
23978	[X]Evid of alcohol involv determind by level of intoxication	Current
24064	Continuous chronic alcoholism	Current
24485	Chronic alcoholism in remission	Ex
24735	O/E - breath - alcohol smell	Current
24984	Alcohol-induced chronic pancreatitis	Current
25110	Alcohol withdrawal hallucinosis	Current
26106	Episodic chronic alcoholism	Current
26323	[X]Alcoholic dementia NOS	Current
26471	Ex-light drinker - (1-2u/day)	Ex
26472	Alcohol intake within recommended sensible limits	Current
27342	Alcoholic dementia NOS	Current
27518	Hangover (alcohol)	Current
27670	Maternal care for (suspected) damage to fetus from alcohol	Current
28150	Nondependent alcohol abuse NOS	Current
28780	[X]Alcohol addiction	Current
29691	Aversion therapy - alcoholism	Current
30162	[X]Alcoholic paranoia	Current
30404	Alcoholic paranoia	Current
30460	Alcoholism counselling	Current
30604	Alcohol-induced epilepsy	Current
30695	Harmful alcohol use	Current
31443	Chronic alcoholism	Current
31569	Nondependent alcohol abuse in remission	Current
31742	Alcoholic myopathy	Current
32927	[X]Alcohol withdrawal-induced seizure	Current
33635	Chronic alcoholism NOS	Current
33670	Other alcoholic psychosis	Current
33839	Cerebellar ataxia due to alcoholism	Current
35330	Alcohol consumption counselling	Current
36296	Acute alcoholic intoxication in alcoholism NOS	Current
36748	Alcoholic encephalopathy	Current
37264	Alcohol leaflet given	Current
37691	[X]Chronic alcoholic brain syndrome	Current
37946	Chronic alcoholic brain syndrome	Current
38061	Alcohol induced hallucinations	Current

39327	[X]Mental and behav dis due to use alcohol: dependence syndr	Current
39799	[X]Mental and behav dis due to use alcohol: amnesic syndrome	Current
40530	Acute alcoholic intoxication, unspecified, in alcoholism	Current
41920	Alcohol amnestic syndrome NOS	Current
41983	Alcohol detoxification	Current
43193	Unspecified chronic alcoholism	Current
44299	[X]Mental & behav dis due to use alcohol: acute intoxication	Current
44783	Pain in lymph nodes after alcohol consumption	Current
45169	[X]Men & behav dis due to use alcohol: oth men & behav dis	Current
46677	Alcohol withdrawal regime	Current
46848	DPS - Drinking problem scale	Current
47123	Alcohol counselling by other agencies	Current
47555	Cerebral degeneration due to alcoholism	Current
54209	Advice to change alcohol intake	Current
54505	Other alcoholic dementia	Current
56410	Delivery of rehabilitation for alcohol addiction	Current
56947	Continuous acute alcoholic intoxication in alcoholism	Current
57714	Alcohol dependence with acute alcoholic intoxication	Current
57939	Pathological alcohol intoxication	Current
59574	Acute alcoholic intoxication in remission, in alcoholism	Current
61383	Planned reduction of alcohol consumption	Current
63529	Alcohol misuse - enhanced service completed	Current
64389	[X]Ment & behav dis due use alcohol: unsp ment & behav dis	Current
64409	Self-monitoring of alcohol intake	Current
65754	Alcohol-induced pseudo-Cushing's syndrome	Current
65932	[X]Alcoholic jealousy	Current
67651	Alcoholic psychosis NOS	Current
68111	Other alcoholic psychosis NOS	Current
84218	Disqualified from driving due to excess alcohol	Current
94553	Referral to specialist alcohol treatment service	Current
94670	Alcohol misuse	Current
95181	Alcohol reduction programme	Current
95650	Advice to change drink intake	Current
96054	Extended intervention for excessive alcohol consumptn complt	Current
96993	Referral to alcohol brief intervention service	Current
97163	Advice to change alcoholic drink intake	Current
97261	Brief intervention for excessive alcohol consumptn declined	Current
97309	Advised to contact primary care alcohol worker	Current
97680	Declined referral to specialist alcohol treatment service	Current
12271	Drunkenness NOS	Current
8430	H/O: alcoholism	Ex
17777	Inebriety NOS	Current
99877	Feels should cut down drinking	Current

101718	Drinks in morning to get rid of hangover	Current
102448	Higher risk drinking	Current
102665	Increasing risk drinking	Current
104611	Alcohol-induced acute pancreatitis	Current
12442	Alcohol disorder monitoring	Current
32964	Alcohol abuse monitoring	Current

Prodcode	Product name	Status
2598	Acamprosate 333mg gastro-resistant tablets	Current
871	Disulfiram 200mg tablets	Current
56720	Nalmefene 18mg tablets	Current
6759	Campral EC 333mg tablets (Merck Serono Ltd)	Current
60240	Disulfiram 500mg tablets	Current
2269	Antabuse 200mg tablets (Actavis UK Ltd)	Current
57225	Selincro 18mg tablets (Lundbeck Ltd)	Current
60514	Esperal 500mg tablets (IDIS)	Current
5628	Campral ec 333mg Gastro-resistant tablet (Lipha Pharmaceuticals Ltd)	Current

icd	icd_description	alcstatus
E24.4	Alcohol-induced pseudo-Cushing's syndrome	Current
F10	Mental and behavioural disorders due to use of alcohol	Current
F10.0	Mental & behav dis due to use of alcohol: acute intoxication	Current
F10.1	Mental and behav dis due to use of alcohol: harmful use	Current
F10.2	Mental and behav dis due to use of alcohol: dependence synd	Current
F10.3	Mental and behav dis due to use of alcohol: withdrawal state	Current
F10.4	Men & behav dis due alcohol: withdrawl state with delirium	Current
F10.5	Mental & behav dis due to use of alcohol: psychotic disorder	Current
F10.6	Mental and behav dis due to use of alcohol: amnesic syndrome	Current
F10.7	Men & behav dis due use alc: resid & late-onset psychot dis	Current
F10.8	Men & behav dis due to use of alcohol: oth men & behav dis	Current
F10.9	Ment & behav dis due use alcohol: unsp ment & behav dis	Current
G31.2	Degeneration of nervous system due to alcohol	Current
G62.1	Alcoholic polyneuropathy	Current
G72.1	Alcoholic myopathy	Current
I42.6	Alcoholic cardiomyopathy	Current
K29.2	Alcoholic gastritis	Current
K70	Alcoholic liver disease	Current
K70.0	Alcoholic fatty liver	Current
K70.1	Alcoholic hepatitis	Current
K70.2	Alcoholic fibrosis and sclerosis of liver	Current

K70.3	Alcoholic cirrhosis of liver	Current
K70.4	Alcoholic hepatic failure	Current
K70.9	Alcoholic liver disease, unspecified	Current
K86.0	Alcohol-induced chronic pancreatitis	Current
T51.0	Toxic effect of ethanol	Current
Y90.1	Blood alcohol level of 20-39 mg/100 ml	Current
Y90.2	Blood alcohol level of 40-59 mg/100 ml	Current
Y90.3	Blood alcohol level of 60-79 mg/100 ml	Current
Y90.4	Blood alcohol level of 80-99 mg/100 ml	Current
Y90.5	Blood alcohol level of 100-119 mg/100 ml	Current
Y90.6	Blood alcohol level of 120-199 mg/100 ml	Current
Y90.7	Blood alcohol level of 200-239 mg/100 ml	Current
Y90.8	Blood alcohol level of 240 mg/100 ml or more	Current
Y91.0	Mild alcohol intoxication	Current
Y91.1	Moderate alcohol intoxication	Current
Y91.2	Severe alcohol intoxication	Current
Y91.3	Very severe alcohol intoxication	Current
Z50.2	Alcohol rehabilitation	Current
Z71.4	Alcohol abuse counselling and surveillance	Current
Z72.1	Alcohol use	Current
R78.0	Finding of alcohol in blood	Current

Smoking

Medcode	Readterm	Status
33	Never smoked tobacco	Non-smoker
54	Tobacco consumption	Current smoker
60	Current non-smoker	Non-smoker
90	Ex smoker	Ex-smoker
93	Cigarette smoker	Current smoker
776	Stopped smoking	Ex-smoker
1822	Very heavy smoker - 40+cigs/d	Current smoker
1823	Smoker	Current smoker
1878	Moderate smoker - 10-19 cigs/d	Current smoker
2111	Health ed. - smoking	Current smoker
3568	Heavy smoker - 20-39 cigs/day	Current smoker
9045	Advice on smoking	Current smoker
10184	Pregnancy smoking advice	Current smoker
10211	Smoking cessation milestones	Ex-smoker
10558	Current smoker	Current smoker
10898	Smoking free weeks	Ex-smoker
11527	DNA - Did not attend smoking cessation clinic	Current smoker
11713	Pack years	Current or ex-smoker
11788	Non-smoker	Non-smoker
12878	Date ceased smoking	Ex-smoker
12941	Occasional smoker	Current smoker
12942	Smoker - amount smoked	Current smoker
12943	Cigar smoker	Current smoker
12944	Light smoker - 1-9 cigs/day	Current smoker
12945	Rolls own cigarettes	Current smoker
12946	Ex-smoker - amount unknown	Ex-smoker
12947	Pipe smoker	Current smoker
12951	Smoking restarted	Current smoker
12952	Smoking started	Current smoker
12954	[V]Tobacco use	Current smoker
12955	Ex-moderate smoker (10-19/day)	Ex-smoker
12956	Ex-heavy smoker (20-39/day)	Ex-smoker
12957	Ex-light smoker (1-9/day)	Ex-smoker
12958	Trivial smoker - < 1 cig/day	Current smoker
12959	Ex-very heavy smoker (40+/day)	Ex-smoker
12960	Tobacco consumption NOS	Current smoker
12961	Ex-trivial smoker (<1/day)	Ex-smoker
12963	Cigar consumption	Current smoker
12964	Keeps trying to stop smoking	Current smoker
12965	Cigarette consumption	Current smoker

12966	Smoking reduced	Current smoker
12967	Pipe tobacco consumption	Current smoker
13351	Passive smoker	Non-smoker
18926	Lifestyle advice regarding smoking	Current smoker
19485	Stop smoking monitor.chck done	Current or ex-smoker
19488	Ex cigar smoker	Ex-smoker
21637	Stop smoking monitor admin.NOS	Current or ex-smoker
24529	Nicotine replacement therapy refused	Current smoker
25106	Nicotine replacement therapy provided free	Current or ex-smoker
26470	Ex pipe smoker	Ex-smoker
28834	Anti-smoking monitoring admin.	Current or ex-smoker
30423	Thinking about stopping smoking	Current smoker
30762	Not interested in stopping smoking	Current smoker
31114	Ready to stop smoking	Current smoker
32083	Stop smoking clinic admin.	Current or ex-smoker
32687	Tobacco dependence	Current smoker
34126	Negotiated date for cessation of smoking	Current smoker
35055	[V]Tobacco abuse counselling	Current smoker
38112	Smoking cessation programme start date	Current smoker
40417	Stop smoking monitor default	Current or ex-smoker
40418	Refuses stop smoking monitor	Current smoker
41979	Smoking restarted	Current smoker
42288	Pack years	Current or ex-smoker
43433	Toxic effect of tobacco and nicotine	Current smoker
46300	Cigarette pack-years	Current or ex-smoker
46321	Reason for restarting smoking	Current smoker
47273	Motives for smoking scale	Current smoker
49418	RFS - Reasons for smoking scale	Current smoker
52503	No smokers in the household	Non-smoker
56144	[X]Mental and behav dis due to use of tobacco: harmful use	Current smoker
57639	Bupropion refused	Current smoker
59866	Reasons for smoking scale	Current smoker
60720	Stop smoking monitor 2nd lettr	Current or ex-smoker
61905	[X]Mental and behavioural disorder due to use of tobacco	Current smoker
62686	Minutes from waking to first tobacco consumption	Current smoker
63016	[X]Bupropion causing adverse effects in therapeutic use	Current or ex-smoker
63299	FTND - Fagerstrom test for nicotine dependence	Current smoker
63666	Fagerstrom test for nicotine dependence	Current smoker
63901	Stop smoking monitoring delete	Current or ex-smoker
66387	Stop smoking monitor 3rd lettr	Current or ex-smoker
67178	Nicotine replacement therapy provided by community pharmacis	Current or ex-smoker
68658	Tobacco dependence NOS	Current smoker
70746	Tobacco dependence, continuous	Current smoker

72706	Tobacco dependence in remission	Ex-smoker
90522	Smoking cessation therapy NOS	Current or ex-smoker
91513	Occasions for smoking scale	Current smoker
91708	Other specified smoking cessation therapy	Current or ex-smoker
95610	Tobacco dependence, unspecified	Current smoker
96992	Smoking cessation - enhanced services administration	Current or ex-smoker
97210	Ex-cigarette smoker	Ex-smoker
97643	Fagerstrom test for nicotine dependence	Current smoker
98177	Non-smoker annual review - enhanced services administration	Non-smoker
98283	COPD structured smoking assessment declined - enh serv admin	Current or ex-smoker
98284	Refer COPD structured smoking assessment - enhanc serv admin	Current or ex-smoker
98347	Current smoker annual review - enhanced services admin	Current smoker
98447	Ex-smoker annual review - enhanced services administration	Ex-smoker
98493	Smoking cessatn monitor template complet - enhanc serv admin	Current or ex-smoker
99838	Recently stopped smoking	Ex-smoker
100099	Smoking cessation advice declined	Current smoker
100495	Ex roll-up cigarette smoker	Ex-smoker
100963	Ex-smoker annual review	Ex-smoker
101210	Consent given for smoking cessation data sharing	Current or ex-smoker
101325	Declin cons follow-up evaluation after smoking cess interven	Current or ex-smoker
101338	Failed attempt to stop smoking	Current smoker
101385	Consent given for follow-up by smoking cessation team	Current or ex-smoker
101634	Consent given follow-up after smoking cessation intervention	Current or ex-smoker
101851	Declined consent for follow-up by smoking cessation team	Current or ex-smoker
101854	Declined consent for smoking cessation data sharing	Current or ex-smoker
101878	Non-smoker annual review	Non-smoker
102361	Referral for smoking cessation service offered	Current smoker
102951	Lost to smoking cessation follow-up	Current or ex-smoker
103760	COPD structured smoking assessment declined	Current or ex-smoker
104310	Current smoker annual review	Current smoker
108835	Tobacco dependence, episodic	Current smoker
107792	[X]Mental and behav dis due to use tobacco: dependence syndr	Current smoker
103208	Smoking status at 12 weeks	Current or ex-smoker
108966	Smoking cessation ESA monitoring template completed	Current or ex-smoker
16717	Smokers' cough	Current or ex-smoker
10742	Referral to stop-smoking clinic	Current smoker
11356	Seen by smoking cessation advisor	Current smoker
7622	Smoking cessation advice	Current smoker
72700	[V]Personal history of tobacco abuse	Ex-smoker
63717	Bupropion contraindicated	Current or ex-smoker
66409	Nicotine replacement therapy contraindicated	Current or ex-smoker
104086	Stop smoking invitation first SMS text message	Current or ex-smoker
104185	Smoking cessation drug therapy declined	Current or ex-smoker

104230	Smoking cessation programme declined	Current or ex-smoker
105572	Stop smoking invitation short message service text message	Current or ex-smoker
106359	Referral to smoking cessation service	Current or ex-smoker
106384	Stop smoking invitation second SMS text message	Current or ex-smoker
106385	Stop smoking invitation third SMS text message	Current or ex-smoker
106391	Referral to smoking cessation service declined	Current or ex-smoker
107504	MFS - Motives for smoking scale	Current or ex-smoker
34127	Smoking status at 4 weeks	Current or ex-smoker
34374	Smoking status between 4 and 52 weeks	Current or ex-smoker
41405	Smoking status at 52 weeks	Current or ex-smoker
105710	Smoking cessation 12 week follow-up	Current or ex-smoker
103400	Referred for COPD structured smoking assessment	Current or ex-smoker
6359	Nicotine withdrawal	Current or ex-smoker
7130	Stop smoking monitoring admin.	Current or ex-smoker
9833	Nicotine replacement therapy	Current or ex-smoker
12240	Trying to give up smoking	Current or ex-smoker
12953	Attends stop smoking monitor.	Current or ex-smoker
18573	Referral to smoking cessation advisor	Current or ex-smoker
32572	Over the counter nicotine replacement therapy	Current or ex-smoker
41042	Smoking cessation advice provided by community pharmacist	Current or ex-smoker
42722	Stop smoking monitor 1st lettr	Current or ex-smoker
53101	Stop smoking monitor verb.inv.	Current or ex-smoker
58597	Stop smoking monitor phone inv	Current or ex-smoker
74907	Smoking cessation therapy	Current or ex-smoker
81440	Nicotine replacement therapy using nicotine patches	Current or ex-smoker
85247	Nicotine replacement therapy using nicotine inhalator	Current or ex-smoker
85975	Nicotine replacement therapy using nicotine gum	Current or ex-smoker
89464	Nicotine replacement therapy using nicotine lozenges	Current or ex-smoker
94958	Smoking cessation drug therapy	Current or ex-smoker
98137	Brief intervention for smoking cessation	Current or ex-smoker
98154	Referral to NHS stop smoking service	Current or ex-smoker
98245	Stop smoking face to face follow-up	Current or ex-smoker
101519	[X]Mental and behav dis due to use tobacco: withdrawal state	Current or ex-smoker
101764	Practice based smoking cessation programme start date	Current or ex-smoker

Prodcode	Product name	Status
1703	Nicorette 15mg Transdermal patch (Pharmacia Ltd)	Current or ex-smoker
42221	Nicotine 4mg lozenges sugar free (Teva UK Ltd)	Current or ex-smoker
38958	Nicotinell 1mg lozenges (Novartis Consumer Health UK Ltd)	Current or ex-smoker
41765	Nicotinell Mint 2mg medicated chewing gum (Novartis Consumer Health UK Ltd)	Current or ex-smoker
5502	Nicotine 15mg/16hours transdermal patches	Current or ex-smoker

39123	Nicotine 25mg/16hours transdermal patches	Current or ex-smoker
58034	Nicotine 2.5mg orodispersible films sugar free	Current or ex-smoker
48620	Boots NicAssist 10mg Inhalator (The Boots Company Plc)	Current or ex-smoker
41809	Nicorette Mint 4mg medicated chewing gum (McNeil Products Ltd)	Current or ex-smoker
5320	Nicorette 10mg Inhalator (McNeil Products Ltd)	Current or ex-smoker
5606	Nicotinell tts 20 sq cm Transdermal patch (Novartis Consumer Health UK Ltd)	Current or ex-smoker
41040	Nicorette lemon 2mg microtab (McNeil Products Ltd)	Current or ex-smoker
54574	Boots NicAssist Minty Fresh 4mg medicated chewing gum (The Boots Company Plc)	Current or ex-smoker
5784	Nicotine 4mg lozenges sugar free	Current or ex-smoker
42016	Nicorette Mint 2mg medicated chewing gum (McNeil Products Ltd)	Current or ex-smoker
57731	NicoBloc liquid (NicoBloc Plc)	Current or ex-smoker
42047	Nicotinell Liquorice 4mg medicated chewing gum (Novartis Consumer Health UK Ltd)	Current or ex-smoker
27411	Champix 1mg tablets (Pfizer Ltd)	Current or ex-smoker
5457	Nicotine 5mg/16hours transdermal patches	Current or ex-smoker
4717	Niquitin 14mg Transdermal patch (GlaxoSmithKline Consumer Healthcare)	Current or ex-smoker
41860	Nicotine bitartrate 2mg Sublingual tablet	Current or ex-smoker
61777	Nicotinell Support Icemint 4mg medicated chewing gum (Novartis Consumer Health UK Ltd)	Current or ex-smoker
62246	Nicotinell Support Icemint 2mg medicated chewing gum (Novartis Consumer Health UK Ltd)	Current or ex-smoker
41376	Nicorette 15mg patches (McNeil Products Ltd)	Current or ex-smoker
55417	Zyban 150mg modified-release tablets (Lexon (UK) Ltd)	Current or ex-smoker
27414	Varenicline 1mg tablets	Current or ex-smoker
2876	Nicorette Citrus 2mg medicated chewing gum (Pfizer Ltd)	Current or ex-smoker
1248	Nicorette 10mg/ml Nasal spray (Pharmacia Ltd)	Current or ex-smoker
35035	Champix 0.5mg tablets (Pfizer Ltd)	Current or ex-smoker
5515	Nicotine 1mg Lozenge	Current or ex-smoker
25510	Nicotine 2mg mint flavour chewing-gum	Current or ex-smoker
54102	NiQuitin Pre-Quit Clear 21mg patches (GlaxoSmithKline Consumer Healthcare)	Current or ex-smoker
4704	Niquitin 7mg Transdermal patch (GlaxoSmithKline Consumer Healthcare)	Current or ex-smoker
41425	Nicorette Freshmint 4mg medicated chewing gum (McNeil Products Ltd)	Current or ex-smoker
27412	Varenicline 1mg tablets and Varenicline 500microgram tablets	Current or ex-smoker
46717	Nicotine 15mg inhalation cartridges with device	Current or ex-smoker
40617	Nicotinell TTS 20 patches (Novartis Consumer Health UK Ltd)	Current or ex-smoker
41778	Nicorette Freshfruit 4mg medicated chewing gum (McNeil Products Ltd)	Current or ex-smoker
3818	Nicotinell tts 30 sq cm Transdermal patch (Novartis Consumer Health UK Ltd)	Current or ex-smoker
27410	Champix titration pack (Pfizer Ltd)	Current or ex-smoker
58675	Boots NicAssist 5mg patches (The Boots Company Plc)	Current or ex-smoker
25516	Nicotine 4mg mint flavour chewing-gum	Current or ex-smoker
37646	Nicotine 1.5mg lozenges sugar free	Current or ex-smoker
41879	Nicotinell Liquorice 2mg medicated chewing gum (Novartis Consumer Health UK Ltd)	Current or ex-smoker

5946	Nicotinell 2mg Medicated chewing-gum (Novartis Consumer Health UK Ltd)	Current or ex-smoker
60236	Boots NicAssist 10mg patches (The Boots Company Plc)	Current or ex-smoker
49204	Champix titration pack (Mawdsley-Brooks & Company Ltd)	Current or ex-smoker
3404	Niquitin 21mg Transdermal patch (GlaxoSmithKline Consumer Healthcare)	Current or ex-smoker
41372	NiQuitin Clear 21mg patches (GlaxoSmithKline Consumer Healthcare)	Current or ex-smoker
46592	Nicorette 15mg Inhalator (McNeil Products Ltd)	Current or ex-smoker
45504	Nicotine 1mg/dose oromucosal spray sugar free	Current or ex-smoker
10623	Nicabate 7mg Transdermal patch (Marion Merrell Dow Ltd)	Current or ex-smoker
41507	NiQuitin Clear 7mg patches (GlaxoSmithKline Consumer Healthcare)	Current or ex-smoker
42048	Nicotine bitartrate 1mg lozenges sugar free	Current or ex-smoker
27311	Niconil 22mg/24 hr Transdermal patch (Elan Pharma)	Current or ex-smoker
6448	Nicotine 21mg/24hours transdermal patches	Current or ex-smoker
41426	NiQuitin 7mg patches (GlaxoSmithKline Consumer Healthcare)	Current or ex-smoker
41801	Nicorette Freshmint 2mg medicated chewing gum (McNeil Products Ltd)	Current or ex-smoker
49901	Champix 1mg tablets (Waymade Healthcare Plc)	Current or ex-smoker
5440	Nicorette 10mg Transdermal patch (Pharmacia Ltd)	Current or ex-smoker
4166	Nicorette Citrus 4mg medicated chewing gum (Pfizer Ltd)	Current or ex-smoker
40683	Nicotinell TTS 10 patches (Novartis Consumer Health UK Ltd)	Current or ex-smoker
49607	Boots NicAssist 15mg patches (The Boots Company Plc)	Current or ex-smoker
6018	Nicorette 5mg Transdermal patch (Pharmacia Ltd)	Current or ex-smoker
40865	NiQuitin Minis Mint 4mg lozenges (GlaxoSmithKline Consumer Healthcare)	Current or ex-smoker
33392	Nicotine 22mg/24 hr Transdermal patch	Current or ex-smoker
41909	Nicotinell Mint 4mg medicated chewing gum (Novartis Consumer Health UK Ltd)	Current or ex-smoker
5877	Nicorette 2mg microtab (Pharmacia Ltd)	Current or ex-smoker
41505	NiQuitin Clear 14mg patches (GlaxoSmithKline Consumer Healthcare)	Current or ex-smoker
45429	Nicorette QuickMist 1mg/dose mouthspray (McNeil Products Ltd)	Current or ex-smoker
41485	NiQuitin 14mg patches (GlaxoSmithKline Consumer Healthcare)	Current or ex-smoker
49319	Nicorette Cools 4mg lozenges (McNeil Products Ltd)	Current or ex-smoker
31939	Nicorette 4mg mint flavour chewing-gum (Pharmacia Ltd)	Current or ex-smoker
56552	Boots NicAssist Minty Fresh 2mg medicated chewing gum (The Boots Company Plc)	Current or ex-smoker
5944	Nicotine 10mg inhalation cartridges with device	Current or ex-smoker
41368	NiQuitin 21mg patches (GlaxoSmithKline Consumer Healthcare)	Current or ex-smoker
50541	Champix titration pack (Sigma Pharmaceuticals Plc)	Current or ex-smoker
39572	Nicorette invisi 10mg/16hours patches (McNeil Products Ltd)	Current or ex-smoker
44106	NiQuitin Minis Cherry 1.5mg lozenges (GlaxoSmithKline Consumer Healthcare)	Current or ex-smoker
6698	Nicotinell 2mg lozenges (Novartis Consumer Health UK Ltd)	Current or ex-smoker
41881	Nicotinell Classic 2mg medicated chewing gum (Novartis Consumer Health UK Ltd)	Current or ex-smoker
9591	Nicotine 14mg/24hours transdermal patches	Current or ex-smoker
41864	Nicorette Freshfruit 2mg medicated chewing gum (McNeil Products Ltd)	Current or ex-smoker
39166	Nicorette invisi 15mg/16hours patches (McNeil Products Ltd)	Current or ex-smoker

29680	Niconil 11mg/24 hr Transdermal patch (Elan Pharma)	Current or ex-smoker
5758	Nicotine 4mg medicated chewing gum sugar free	Current or ex-smoker
41802	Nicorette 5mg patches (McNeil Products Ltd)	Current or ex-smoker
36457	Nicopatch 21mg/24hours transdermal patches (Pierre Fabre Ltd)	Current or ex-smoker
6630	NiQuitin Mint 2mg lozenges (GlaxoSmithKline Consumer Healthcare)	Current or ex-smoker
10527	Nicabate 14mg Transdermal patch (Marion Merrell Dow Ltd)	Current or ex-smoker
40730	NiQuitin Minis Mint 1.5mg lozenges (GlaxoSmithKline Consumer Healthcare)	Current or ex-smoker
57829	NiQuitin Strips Mint 2.5mg oral films (GlaxoSmithKline Consumer Healthcare)	Current or ex-smoker
36618	Nicopatch 7mg/24hours transdermal patches (Pierre Fabre Ltd)	Current or ex-smoker
5700	NiQuitin 2mg lozenges original menthol mint (GlaxoSmithKline Consumer Healthcare)	Current or ex-smoker
57417	Nicorette 5mg patches (Waymade Healthcare Plc)	Current or ex-smoker
45603	Nicorette Freshmint 2mg lozenges (McNeil Products Ltd)	Current or ex-smoker
41753	Nicorette Original 4mg medicated chewing gum (McNeil Products Ltd)	Current or ex-smoker
6642	NiQuitin Mint 4mg medicated chewing gum (GlaxoSmithKline Consumer Healthcare)	Current or ex-smoker
11718	Nicotine 2mg sublingual tablets sugar free	Current or ex-smoker
13048	Nicotinell 4mg Medicated chewing-gum (Novartis Consumer Health UK Ltd)	Current or ex-smoker
36635	Nicopatch 14mg/24hours transdermal patches (Pierre Fabre Ltd)	Current or ex-smoker
49305	Nicorette Cools 2mg lozenges (McNeil Products Ltd)	Current or ex-smoker
5531	Nicotinell 1mg Lozenge (Novartis Consumer Health UK Ltd)	Current or ex-smoker
6593	NiQuitin Mint 4mg lozenges (GlaxoSmithKline Consumer Healthcare)	Current or ex-smoker
39046	Nicorette invisi 25mg/16hours patches (McNeil Products Ltd)	Current or ex-smoker
41474	Nicorette 10mg patches (McNeil Products Ltd)	Current or ex-smoker
467	Zyban 150mg modified-release tablets (GlaxoSmithKline UK Ltd)	Current or ex-smoker
41808	Nicotinell Fruit 4mg medicated chewing gum (Novartis Consumer Health UK Ltd)	Current or ex-smoker
50487	NiQuitin Clear 14mg patches (Waymade Healthcare Plc)	Current or ex-smoker
37716	Nicopass 1.5mg Lozenge (Wockhardt UK Ltd)	Current or ex-smoker
5659	NiQuitin 4mg lozenges original menthol mint (GlaxoSmithKline Consumer Healthcare)	Current or ex-smoker
41356	Nicorette Microtab 2mg sublingual tablets (McNeil Products Ltd)	Current or ex-smoker
5115	Bupropion 150mg modified-release tablets	Current or ex-smoker
7303	Nicotinell tts 10 sq cm Transdermal patch (Novartis Consumer Health UK Ltd)	Current or ex-smoker
41377	Nicorette Original 2mg medicated chewing gum (McNeil Products Ltd)	Current or ex-smoker
42011	Nicotinell Classic 4mg medicated chewing gum (Novartis Consumer Health UK Ltd)	Current or ex-smoker
7644	Nicabate 21mg Transdermal patch (Marion Merrell Dow Ltd)	Current or ex-smoker
46588	Nicotinell Icemint 2mg medicated chewing gum (Novartis Consumer Health UK Ltd)	Current or ex-smoker
58410	NiQuitin Minis Orange 1.5mg lozenges (GlaxoSmithKline Consumer Healthcare)	Current or ex-smoker
46701	Nicotinell Icemint 4mg medicated chewing gum (Novartis Consumer Health UK Ltd)	Current or ex-smoker
41931	Nicotinell Fruit 2mg medicated chewing gum (Novartis Consumer Health UK Ltd)	Current or ex-smoker

8571	Nicotine 500micrograms/dose nasal spray	Current or ex-smoker
49088	NiQuitin Clear 21mg patches (Waymade Healthcare Plc)	Current or ex-smoker
25523	Nicorette 2mg mint flavour chewing-gum (Pharmacia Ltd)	Current or ex-smoker
41496	Nicorette 500micrograms/dose nasal spray (McNeil Products Ltd)	Current or ex-smoker
9806	Nicotine 2mg lozenges sugar free	Current or ex-smoker
6565	NiQuitin Mint 2mg medicated chewing gum (GlaxoSmithKline Consumer Healthcare)	Current or ex-smoker
35089	Varenicline 500microgram tablets	Current or ex-smoker
39521	NiQuitin Pre-Quit Mint 4mg lozenges (GlaxoSmithKline Consumer Healthcare)	Current or ex-smoker
9804	Nicotine 7mg/24hours transdermal patches	Current or ex-smoker
42286	Nicotine bitartrate 2mg lozenges sugar free	Current or ex-smoker
6323	Nicotine 2mg medicated chewing gum sugar free	Current or ex-smoker
40620	Nicotinell TTS 30 patches (Novartis Consumer Health UK Ltd)	Current or ex-smoker
55590	Nicotine 11mg/24 hr Transdermal patch	Current or ex-smoker
5479	Nicotine 10mg/16hours transdermal patches	Current or ex-smoker
41493	Nicorette Icy White 4mg medicated chewing gum (McNeil Products Ltd)	Current or ex-smoker
41779	Nicorette Icy White 2mg medicated chewing gum (McNeil Products Ltd)	Current or ex-smoker

icd	icd_description	Smokstatus
F17	Mental and behavioural disorders due to use of tobacco	Current smoker
F17.0	Men & behav dis due use tobacco: acute intoxication	Current smoker
F17.1	Men & behav dis due use tobacco: harmful use	Current smoker
F17.2	Men & behav dis due use tobacco: dependence syndrome	Current smoker
F17.3	Men & behav dis due use tobacco: withdrawl state	Current or ex-smoker
F17.4	Men & behav dis due use tobacco: withdrawl state + delirium	Current or ex-smoker
F17.5	Men & behav dis due use tobacco: psychotic disorder	Current smoker
F17.6	Men & behav dis due use tobacco: amnesic syndrome	Current smoker
F17.7	Men & behav dis due use tobacco: res & late-ons psych dis	Current smoker
F17.8	Men & behav dis due use tobacco: oth men & behav dis	Current smoker
F17.9	Men & behav dis due use tobacco: unsp men & behav dis	Current smoker
T65.2	Toxic effect of tobacco and nicotine	Current smoker
Z71.6	Tobacco abuse counselling	Current smoker
Z72.0	Tobacco use	Current smoker

Myocardial infarction

Medcode	Readterm
241	Acute myocardial infarction
1204	Heart attack
1677	MI - acute myocardial infarction
1678	Inferior myocardial infarction NOS
2491	Coronary thrombosis
3704	Acute subendocardial infarction
5387	Other specified anterior myocardial infarction
7783	ECG: myocardial infarction
8935	Acute inferolateral infarction
9507	Acute non-Q wave infarction
10562	Acute non-Q wave infarction
12139	Acute anterolateral infarction
12229	Acute ST segment elevation myocardial infarction
13566	Attack - heart
13571	Thrombosis - coronary
14658	Acute myocardial infarction NOS
14897	Anterior myocardial infarction NOS
14898	Lateral myocardial infarction NOS
15661	Dressler's syndrome
17133	Mural thrombosis
17872	Acute anteroseptal infarction
18842	Subsequent myocardial infarction
21854	Left ventricular thrombosis
23708	Atrial septal defect/curr comp folow acut myocardal infarct
23892	Posterior myocardial infarction NOS
24126	Haemopericardium/current comp folow acut myocard infarct
26966	ECG: S-T elevation
26972	ECG:posterior/inferior infarct
26975	ECG: antero-septal infarct.
28736	Acute atrial infarction
29553	Thrombosis atrium,auric append&vent/curr comp foll acute MI
29643	Acute inferoposterior infarction
29758	Acute transmural myocardial infarction of unspecif site
30330	Acute Q-wave infarct
30421	Cardiac rupture following myocardial infarction (MI)
32272	Postoperative myocardial infarction
32854	Acute posterolateral myocardial infarction
34803	Other acute myocardial infarction
35119	Post infarction pericarditis
36423	Certain current complication follow acute myocardial infarct

37657	Ventric septal defect/curr comp fol acut myocardal infarctn
38609	Subsequent myocardial infarction of inferior wall
39449	Coronary thrombosis not resulting in myocardial infarction
40429	Acute anteroapical infarction
40996	Percut translum coronary thrombolytic therapy- streptokinase
41221	Acute septal infarction
41835	Postoperative subendocardial myocardial infarction
45809	Subsequent myocardial infarction of anterior wall
46017	Other acute myocardial infarction NOS
46112	Postoperative transmural myocardial infarction anterior wall
46166	Subsequent myocardial infarction of unspecified site
46276	Postoperative transmural myocardial infarction inferior wall
52705	ECG: lateral infarction
55401	ECG: subendocardial infarct
59032	ECG: myocardial infarct NOS
59189	Ruptur cardiac wall w'out haemopericard/cur comp fol ac MI
59940	Ruptur chordae tendinae/curr comp fol acute myocard infarct
61670	Diab mellit insulin-glucose infus acute myocardial infarct
62626	Acute papillary muscle infarction
63467	True posterior myocardial infarction
68357	Microinfarction of heart
68748	Postoperative myocardial infarction, unspecified
69474	Rupture papillary muscle/curr comp fol acute myocard infarct
72562	Subsequent myocardial infarction of other sites
96838	[X]Acute transmural myocardial infarction of unspecif site
99991	[X]Subsequent myocardial infarction of unspecified site
106812	Postoperative transmural myocardial infarction unspec site
109035	[X]Subsequent myocardial infarction of other sites

icd	icd_description
I21	Acute myocardial infarction
I21.0	Acute transmural myocardial infarction of anterior wall
I21.1	Acute transmural myocardial infarction of inferior wall
I21.2	Acute transmural myocardial infarction of other sites
I21.3	Acute transmural myocardial infarction of unspecified site
I21.4	Acute subendocardial myocardial infarction
I21.9	Acute myocardial infarction, unspecified
I22	Subsequent myocardial infarction
I22.0	Subsequent myocardial infarction of anterior wall
I22.1	Subsequent myocardial infarction of inferior wall
I22.8	Subsequent myocardial infarction of other sites

I22.9	Subsequent myocardial infarction of unspecified site
I23	Certain current complication follow acute myocardial infarct
I23.0	Haemopericardium as curr comp folow acut myocard infarct
I23.1	Atral sept defect as curr comp folow acut myocardal infarct
I23.2	Ventric sep defect as curr comp fol acut myocardal infarc
I23.3	Rup cardac wal withou haemopercard as cur comp fol ac MI
I23.4	Rup chordae tendinae as curr comp fol acut myocard infarct
I23.5	Rup papilary muscle as curr comp fol acute myocard infarct
I23.6	Thromb atrium/auric append/vent as curr comp foll acute MI
I23.8	Oth current comp following acute myocardial infarction

Other ischaemic heart disease

Medcode	Readterm
240	Ischaemic heart disease
732	Transluminal balloon angioplasty of coronary artery NOS
733	Percutaneous transluminal angioplasty of artery NEC
737	Coronary artery bypass graft operations
1344	Coronary artery disease
1414	Angina on effort
1430	Angina pectoris
1431	Unstable angina
1655	Triple vessel disease of the heart
1676	Ischaemic heart disease NOS
1792	IHD - Ischaemic heart disease
2155	Ventricular cardiac aneurysm
2901	Transluminal balloon angioplasty of coronary artery
3159	Other specified other bypass of coronary artery
3999	Single coronary vessel disease
4656	Crescendo angina
5030	[V]Presence of coronary artery bypass graft
5254	Double coronary vessel disease
5413	Coronary atherosclerosis
5674	[V]Presence of coronary artery bypass graft - CABG
5703	Percutaneous balloon coronary angioplasty
5744	Open angioplasty of coronary artery
6182	Other therapeutic transluminal op on coronary artery OS
6331	Aneurysm of heart
6336	H/O: angina pectoris
6980	[V]Status following coronary angioplasty NOS
7134	Other autograft bypass of coronary artery
7137	Saphenous vein graft replacement of coronary artery OS
7320	Ischaemic cardiomyopathy
7347	Unstable angina
7442	Saphenous vein graft replacement of three coronary arteries
7609	Other autograft replacement of coronary artery NOS
7634	Saphenous vein graft replacement of two coronary arteries
7696	Syncope anginosa
8312	Saphenous vein graft bypass of coronary artery
8568	Cardiac syndrome X
8679	Saphenous vein graft replacement of one coronary artery
8942	Insertion of coronary artery stent
9276	Acute coronary insufficiency
9413	Other acute and subacute ischaemic heart disease

9414	Other autograft replacement of coronary artery
9555	Post infarct angina
10209	Autograft replacement of three coronary arteries NEC
11048	Variant angina pectoris
11610	Saphenous vein graft replacement of four+ coronary arteries
11983	Acute coronary syndrome
12734	Coronary artery bypass graft occlusion
12804	Stable angina
12986	Prinzmetal's angina
13185	Angina control
14782	Angina control - improving
15349	Angina control NOS
15373	Angina control - poor
15754	Other chronic ischaemic heart disease NOS
17307	Angina at rest
18118	Worsening angina
18125	Nocturnal angina
18249	Saphenous vein graft replacement of coronary artery
18643	[V]Presence of coronary angioplasty implant and graft
18670	Percut transluminal balloon angioplasty one coronary artery
18889	Asymptomatic coronary heart disease
18913	[V]Presence of aortocoronary bypass graft
19046	Rotary blade coronary angioplasty
19193	Prosthetic replacement of coronary artery NOS
19402	Prosthetic replacement of coronary artery
19413	Autograft replacement of two coronary arteries NEC
19542	Angina control - good
19655	Angina at rest
20095	Angina decubitus
20416	Atherosclerotic heart disease
20903	Peroperative angioplasty
21844	Transient myocardial ischaemia
22020	Endarterectomy of coronary artery NEC
22383	Other specified ischaemic heart disease
22647	LIMA single anastomosis
22828	Percutaneous transluminal laser coronary angioplasty
23078	Chronic myocardial ischaemia
24540	Chronic coronary insufficiency
24783	Arteriosclerotic heart disease
24888	Other therapeutic transluminal operations on coronary artery
25842	Angina pectoris NOS
26863	New onset angina
27484	Cardiac aneurysm

27951	Other acute and subacute ischaemic heart disease
27977	Other acute and subacute ischaemic heart disease NOS
28138	Other chronic ischaemic heart disease
28554	Angina pectoris NOS
28837	Creation of bypass from mammary artery to coronary artery
29300	Angina control - worsening
29421	Silent myocardial ischaemia
29902	Angina decubitus NOS
31519	Double implant of mammary arteries into coronary arteries
31540	Revision of bypass for three coronary arteries
31556	Allograft replacement of coronary artery
31679	Other therapeutic transluminal op on coronary artery NOS
32450	Ischaemic chest pain
32651	Allograft bypass of coronary artery
33461	Revision of bypass for coronary artery
33471	Other bypass of coronary artery NOS
33620	Repair of coronary artery NEC
33650	Percut transluminal coronary thrombolysis with streptokinase
33718	Double anastomosis of mammary arteries to coronary arteries
33735	Percut translum balloon angioplasty mult coronary arteries
34328	Refractory angina
34633	Other specified chronic ischaemic heart disease
34963	Other bypass of coronary artery
35713	Other specified chronic ischaemic heart disease NOS
36011	Prosthetic bypass of coronary artery
36523	Preinfarction syndrome
36609	Atherosclerotic cardiovascular disease
36854	Coronary artery spasm
37682	Connection of mammary artery to coronary artery
37719	Connection of mammary artery to coronary artery OS
38813	Rotary blade angioplasty
39449	Coronary thrombosis not resulting in myocardial infarction
39546	[X]Other forms of angina pectoris
39655	Impending infarction
39693	Subendocardial ischaemia
40996	Percut translum coronary thrombolytic therapy- streptokinase
41547	Transluminal balloon angioplasty of coronary artery OS
41677	Aneurysm of heart NOS
42304	Insertion of drug-eluting coronary artery stent
42462	Percut translum balloon angioplasty bypass graft coronary a
42708	Autograft replacement of four of more coronary arteries NEC
43939	Perc translumin balloon angioplasty stenting coronary artery
44561	Autograft replacement of one coronary artery NEC

44723	Single anast mammary art to left ant descend coronary art
45370	Allograft replacement of four or more coronary arteries
45886	Allograft replacement of three coronary arteries
45960	Antianginal therapy
47637	[X]Other forms of chronic ischaemic heart disease
48767	Allograft replacement of coronary artery NOS
48822	LIMA sequential anastomosis
51507	Single anastomosis of mammary artery to coronary artery NEC
51515	Saphenous vein graft replacement coronary artery NOS
52517	[X]Ischaemic heart diseases
52938	Revision of bypass for one coronary artery
54251	Preinfarction syndrome NOS
54535	Stenocardia
55092	Replacement of coronary arteries using multiple methods
55137	MI - myocardial infarction aborted
55598	Other replacement of coronary artery
56990	Connection of mammary artery to coronary artery NOS
57062	H/O: Angina in last year
57241	Allograft replacement of two coronary arteries
57634	Revision of bypass for coronary artery NOS
59193	Aneurysm of coronary vessels
59423	Other specified allograft replacement of coronary artery
60067	Perc translum ball angio insert 1-2 drug elut stents cor art
60753	Single implantation thoracic artery into coronary artery NEC
61072	Myocardial infarction aborted
61208	Perc translum balloon angioplasty stenting coronary art NOS
61310	Other autograft replacement of coronary artery OS
62608	Double anastom thoracic arteries to coronary arteries NEC
63153	Revision of implantation of thoracic artery into heart
64923	Prosthetic graft patch angioplasty
66236	Prosthetic replacement of three coronary arteries
66388	Status anginosus
66583	Percut translum inject therap subst to coronary artery NEC
66664	Prosthetic replacement of two coronary arteries
66921	Percutaneous transluminal angioplasty of vascular graft
67087	Other cardiac wall aneurysm
67554	Revision of bypass for two coronary arteries
67591	Single anastomosis of thoracic artery to coronary artery NEC
67761	Prosthetic replacement of four or more coronary arteries
68123	RIMA single anastomosis
68139	Single implantation of mammary artery into coronary artery
68401	[X]Other forms of acute ischaemic heart disease
69776	Mechanical complication of coronary bypass

70111	Allograft replacement of one coronary artery
70185	Percutaneous transluminal atherectomy
70755	Replacement of coronary artery NOS
72780	Connection of other thoracic artery to coronary artery NOS
85947	Perc translum balloon angioplasty insert 1-2 stents cor art
86071	Percut translum cutting balloon angioplasty coronary artery
86773	Percutaneous transluminal balloon angioplasty of artery
87849	Perc tran ball angio ins 3 or more drug elut stents cor art
91774	Acquired atrioventricular fistula of heart
92233	RIMA sequential anastomosis
92419	Prosthetic replacement of one coronary artery
92927	Percutaneous cor balloon angiop 3 more stents cor art NEC
93618	Percutaneous transluminal atherectomy of coronary artery
93828	Other specified replacement of coronary artery
96537	OS perc translumina balloon angioplast stenting coronary art
96804	Connection of other thoracic artery to coronary artery
97953	Other specified revision of bypass for coronary artery
101121	Coronary artery bypass graft operation planned
101569	Revision of bypass for four or more coronary arteries
103655	Frequency of angina
105184	Percutaneous coronary intervention
105216	H/O acute coronary syndrome
105250	Mural cardiac aneurysm
105479	Coronary microvascular disease
107406	Emergency percutaneous coronary intervention
107574	Referral to Angina Plan self-management programme
107967	Angina self-management plan agreed
108056	Referral to Angina Plan self-management programme declined
109391	Angina self-management plan review

icd	icd_description
I20	Angina pectoris
I20.0	Unstable angina
I20.1	Angina pectoris with documented spasm
I20.8	Other forms of angina pectoris
I20.9	Angina pectoris, unspecified
I24	Other acute ischaemic heart diseases
I24.0	Coronary thrombosis not resulting in myocardial infarction
I24.8	Other forms of acute ischaemic heart disease
I24.9	Acute ischaemic heart disease, unspecified
I25	Chronic ischaemic heart disease

I25.0	Atherosclerotic cardiovascular disease, so described
I25.1	Atherosclerotic heart disease
I25.2	Old myocardial infarction
I25.3	Aneurysm of heart
I25.4	Coronary artery aneurysm
I25.5	Ischaemic cardiomyopathy
I25.6	Silent myocardial ischaemia
I25.8	Other forms of chronic ischaemic heart disease
I25.9	Chronic ischaemic heart disease, unspecified
Z95.5	Presence of coronary angioplasty implant and graft

opcs	description
K40	Saphenous vein graft replacement of coronary artery
K401	Saphenous vein graft replacement of one coronary artery
K402	Saphenous vein graft replacement of two coronary arteries
K403	Saphenous vein graft replacement of three coronary arteries
K404	Saphenous vein graft replacement of four or more coronary arteries
K408	Other specified saphenous vein graft replacement of coronary artery
K409	Unspecified saphenous vein graft replacement of coronary artery
K41	Other autograft replacement of coronary artery
K411	Autograft replacement of one coronary artery NEC
K412	Autograft replacement of two coronary arteries NEC
K413	Autograft replacement of three coronary arteries NEC
K414	Autograft replacement of four or more coronary arteries NEC
K418	Other specified other autograft replacement of coronary artery
K419	Unspecified other autograft replacement of coronary artery
K42	Allograft replacement of coronary artery
K421	Allograft replacement of one coronary artery
K422	Allograft replacement of two coronary arteries
K423	Allograft replacement of three coronary arteries
K424	Allograft replacement of four or more coronary arteries
K428	Other specified allograft replacement of coronary artery
K429	Unspecified allograft replacement of coronary artery
K43	Prosthetic replacement of coronary artery
K431	Prosthetic replacement of one coronary artery
K432	Prosthetic replacement of two coronary arteries
K433	Prosthetic replacement of three coronary arteries
K434	Prosthetic replacement of four or more coronary arteries
K438	Other specified prosthetic replacement of coronary artery
K439	Unspecified prosthetic replacement of coronary artery
K44	Other replacement of coronary artery

K441	Replacement of coronary arteries using multiple methods
K442	Revision of replacement of coronary artery
K448	Other specified other replacement of coronary artery
K449	Unspecified other replacement of coronary artery
K45	Connection of thoracic artery to coronary artery
K451	Double anastomosis of mammary arteries to coronary arteries
K452	Double anastomosis of thoracic arteries to coronary arteries NEC
K453	Anastomosis of mammary artery to left anterior descending coronary artery
K454	Anastomosis of mammary artery to coronary artery NEC
K455	Anastomosis of thoracic artery to coronary artery NEC
K456	Revision of connection of thoracic artery to coronary artery
K458	Other specified connection of thoracic artery to coronary artery
K459	Unspecified connection of thoracic artery to coronary artery
K46	Other bypass of coronary artery
K461	Double implantation of mammary arteries into heart
K462	Double implantation of thoracic arteries into heart NEC
K463	Implantation of mammary artery into heart NEC
K464	Implantation of thoracic artery into heart NEC
K465	Revision of implantation of thoracic artery into heart
K468	Other specified other bypass of coronary artery
K469	Unspecified other bypass of coronary artery
K471	Endarterectomy of coronary artery
K49	Transluminal balloon angioplasty of coronary artery
K491	Percutaneous transluminal balloon angioplasty of one coronary artery
K492	Percutaneous transluminal balloon angioplasty of multiple coronary arteries
K493	Percutaneous transluminal balloon angioplasty of bypass graft of coronary artery
K494	Percutaneous transluminal cutting balloon angioplasty of coronary artery
K498	Other specified transluminal balloon angioplasty of coronary artery
K499	Unspecified transluminal balloon angioplasty of coronary artery
K50	Other therapeutic transluminal operations on coronary artery
K501	Percutaneous transluminal laser coronary angioplasty
K502	Percutaneous transluminal coronary thrombolysis using streptokinase
K503	Percutaneous transluminal injection of therapeutic substance into coronary artery NEC
K504	Percutaneous transluminal atherectomy of coronary artery
K508	Other specified other therapeutic transluminal operations on coronary artery
K509	Unspecified other therapeutic transluminal operations on coronary artery
K75	Percutaneous transluminal balloon angioplasty and insertion of stent into coronary artery
K751	Percutaneous transluminal balloon angioplasty and insertion of 1-2 drug-eluting stents into coronary artery
K752	Percutaneous transluminal balloon angioplasty and insertion of 3 or more drug-eluting stents into coronary artery
K753	Percutaneous transluminal balloon angioplasty and insertion of 1-2 stents into coronary artery
K754	Percutaneous transluminal balloon angioplasty and insertion of 3 or more stents into coronary artery NEC

K758	Other specified percutaneous transluminal balloon angioplasty and insertion of stent into coronary artery
K759	Unspecified percutaneous transluminal balloon angioplasty and insertion of stent into coronary artery

Hypertension

Medcode	Readterm
204	Hypertensive disease
351	High blood pressure
799	Essential hypertension
1894	Benign essential hypertension
3712	Hypertension NOS
3979	Hypertensive encephalopathy
4372	Systolic hypertension
4668	Hypertensive renal disease
6702	Hypertensive retinopathy
7057	Hypertensive disease NOS
7329	Secondary hypertension
8732	BP - hypertensive disease
8857	Cardiomegaly - hypertensive
10818	Essential hypertension NOS
15106	Hypertensive renal disease NOS
15377	Malignant essential hypertension
16059	Secondary hypertension NOS
16173	Hypertensive heart disease NOS
16292	Hypertensive heart disease
16565	Good hypertension control
17434	Nephrosclerosis
18590	Moderate hypertension control
18765	Other specified hypertensive disease
21837	Hypertensive heart&renal dis wth (congestive) heart failure
25371	Secondary benign renovascular hypertension
27511	Poor hypertension control
28684	Hypertensive heart and renal disease with renal failure
29310	Renal hypertension
31341	Hypertension secondary to drug
31387	Secondary renovascular hypertension NOS
31464	Hypertensive heart disease NOS
31755	Secondary malignant hypertension
31816	Hypertensive crisis
32423	Hypertensive renal disease with renal failure
32976	Hypertension induced by oral contraceptive pill
34744	Hypertension secondary to endocrine disorders
37086	Blind hypertensive eye
39649	Malignant hypertensive renal disease
42229	Secondary hypertension NOS
43935	Benign hypertensive renal disease

50157	Malignant hypertensive heart disease
51635	Secondary benign hypertension NOS
52127	Benign hypertensive heart disease with CCF
52427	Benign hypertensive heart disease
57288	Secondary benign hypertension
57987	Hyperten heart&renal dis+both(congestv)heart and renal fail
59383	Secondary malignant renovascular hypertension
61166	Hypertensive heart disease NOS without CCF
61660	Benign hypertensive heart disease without CCF
62718	Hypertensive heart disease NOS with CCF
63000	Benign hypertensive heart and renal disease
63466	Hypertensive heart and renal disease
67232	Malignant hypertensive heart and renal disease
68659	Hypertensive heart and renal disease NOS
69753	[X]Hypertensive diseases
72668	Malignant hypertensive heart disease with CCF
73293	Secondary malignant hypertension NOS
83473	Diastolic hypertension
95334	Malignant hypertensive heart disease without CCF
97533	[X]Hypertension secondary to other renal disorders
102458	[X]Other secondary hypertension
103046	Malignant hypertensive heart disease NOS
105274	Stage 2 hypertension (NICE - Nat Ins for Hth Clin Excl 2011)
105316	Stage 1 hypertension
105371	Stage 1 hypertension (NICE - Nat Ins for Hth Clin Excl 2011)
105480	Hypertension resistant to drug therapy
105487	Severe hypertension
105938	Benign hypertensive heart disease NOS
105989	Severe hypertension (Nat Inst for Health Clinical Ex 2011)
107704	Primary hypertension
19342	Hypertension resolved
3269	Hypertension resolved
2666	H/O: hypertension
98230	Lifestyle advice regarding hypertension
18057	Antihypertensive therapy
12948	Hypertension treatm.stopped
22333	Hypertension treatment refused
101649	Other specified high cost hypertension drugs
85944	High cost hypertension drugs
13188	Hypertensive treatm.changed
3425	On treatment for hypertension
21826	Hypertension treatm. started
12680	Hypertension clinical management plan

109611	Hypertension self-management plan agreed
102406	Hypertension 9 month review
30776	Hypertension:follow-up default
19070	Hypertension annual review
18482	Hypertension six month review

icd	icd_description
I10	Essential (primary) hypertension
I11	Hypertensive heart disease
I11.0	Hypertensive heart disease with (congestive) heart failure
I11.9	Hypertensive heart disease without (conges) heart failure
I12	Hypertensive renal disease
I12.0	Hypertensive renal disease with renal failure
I12.9	Hypertensive renal disease without renal failure
I13	Hypertensive heart and renal disease
I13.0	Hypertens heart and renal dis with (conges) heart failure
I13.1	Hypertensive heart and renal disease with renal failure
I13.2	Hyper heart and renal dis both (cong) heart and renal fail
I13.9	Hypertensive heart and renal disease, unspecified
I15	Secondary hypertension
I15.0	Renovascular hypertension
I15.1	Hypertension secondary to other renal disorders
I15.2	Hypertension secondary to endocrine disorders
I15.8	Other secondary hypertension
I15.9	Secondary hypertension, unspecified
I67.4	Hypertensive encephalopathy

Alcoholic liver disease

Medcode	Readterm
1638	Cirrhosis of liver NOS
3216	Acute alcoholic hepatitis
3450	Diffuse nodular cirrhosis
4743	Alcoholic cirrhosis of liver
5129	Portal hypertension
6015	[X]Other and unspecified cirrhosis of liver
6863	Cirrhosis and chronic liver disease
7602	Chronic alcoholic hepatitis
7885	Alcoholic liver damage unspecified
7943	Alcoholic hepatitis
8363	Oesophageal varices in alcoholic cirrhosis of the liver
10691	Alcoholic fatty liver
17330	Alcoholic hepatic failure
21713	Alcoholic fibrosis and sclerosis of liver
22841	Macronodular cirrhosis of liver
26319	Oesophageal varices in cirrhosis of the liver
40567	Capsular portal cirrhosis
44120	Toxic liver disease with fibrosis and cirrhosis of liver
44676	Fatty portal cirrhosis
47257	Portal cirrhosis
55454	Portal cirrhosis unspecified
68376	Florid cirrhosis
69204	Multilobular portal cirrhosis
92909	Hypertrophic portal cirrhosis
100474	Laennec's cirrhosis
100592	Hepatic fibrosis with hepatic sclerosis
71240	Transjugular intrahepatic creation of portosystemic shunt
87861	Oth transjugular intrahepatic operations blood vessel liver
89356	Percutaneous transluminal insertion of stent portal vein
90925	Transjugular intrahepatic operations blood vessel of liver
93737	Transjugular intrahepatic insertion stent into portal vein
95118	Percutan transluminal insertion of stent graft portal vein
106179	Transjugular intrahepatic angioplasty of portal vein
106828	Transjugular intrahepatic operations blood vessel liver NOS
107093	Transjugular intrahepatic ins stent graft into portal vein

icd	icd_description
K70	Alcoholic liver disease
K70.0	Alcoholic fatty liver

K70.1	Alcoholic hepatitis
K70.2	Alcoholic fibrosis and sclerosis of liver
K70.3	Alcoholic cirrhosis of liver
K70.4	Alcoholic hepatic failure
K70.9	Alcoholic liver disease, unspecified
K76.6	Portal hypertension

opcs	description
J111	Transjugular intrahepatic angioplasty of portal vein
J114	Transjugular intrahepatic creation of portosystemic shunt
J118	Other specified transjugular intrahepatic operations on blood vessel of liver
J119	Unspecified transjugular intrahepatic operations on blood vessel of liver

Pancreatitis

Medcode	Readterm
1419	Chronic pancreatitis
24984	Alcohol-induced chronic pancreatitis
49353	[X]Other chronic pancreatitis
71633	History of chronic pancreatitis
104611	Alcohol-induced acute pancreatitis

icd	icd_description
K85	Acute pancreatitis
K86.0	Alcohol-induced chronic pancreatitis
K86.1	Other chronic pancreatitis

Peripheral artery disease

Medcode	Readterm
44835	Embolism and thrombosis of a leg artery NOS
30495	Embolism and thrombosis of an arm artery NOS
44085	Embolism and thrombosis of an arm or leg artery
41597	Embolism and thrombosis of other arteries NOS
32235	Embolism and thrombosis of other specified artery
69232	Embolism and thrombosis of the anterior tibial artery
31460	Embolism and thrombosis of the axillary artery
34159	Embolism and thrombosis of the brachial artery
47665	Embolism and thrombosis of the coeliac artery
71860	Embolism and thrombosis of the dorsalis pedis artery
2065	Embolism and thrombosis of the femoral artery
27494	Embolism and thrombosis of the iliac artery unspecified
4539	Embolism and thrombosis of the popliteal artery
99532	Embolism and thrombosis of the posterior tibial artery
29372	Embolism and thrombosis of the radial artery
6900	Embolism and thrombosis of the subclavian artery
62368	Embolism and thrombosis of the ulnar artery
54865	Embolism and/or thrombosis of the common iliac artery
56919	Embolism and/or thrombosis of the external iliac artery
32634	Embolism and/or thrombosis of the internal iliac artery
15302	Peripheral arterial embolism and thrombosis NOS
109425	[X]Embolism and thrombosis of other arteries
28004	Arterial embolic and thrombotic occlusion
4289	Arterial embolism and thrombosis
3714	Arterial embolism and thrombosis NOS
8998	Arterial embolus and thrombosis
107071	ARAS - Atherosclerotic renal artery stenosis
16284	Renal artery atherosclerosis
23871	Peripheral angiopathic disease EC NOS
100579	[X]Atherosclerosis of other arteries
996	Arteriosclerosis
3995	Arteriosclerotic vascular disease NOS
5640	Atherosclerosis
36609	Atherosclerotic cardiovascular disease
56621	Cardiovascular arteriosclerosis unspecified
5168	Other specified artery atheroma
51804	Monckeberg's medial sclerosis
4970	[D]Gangrene
37750	[D]Gangrene NOS
73961	[X]Other specified peripheral vascular diseases

23497	Buerger's disease
6853	Claudication
7975	Claudication distance
12735	Gangrene of foot
5414	Gangrene of toe
1517	Intermittent claudication
1826	Ischaemia of legs
98174	Ischaemic foot
6308	Ischaemic leg ulcer
9561	Ischaemic toe
40068	Presenile gangrene
34638	Thromboangiitis obliterans
67401	Thromboangiitis obliterans NOS
101866	Vascular claudication
9204	Peripheral gangrene
14797	Extremity artery atheroma
16260	Extremity artery atheroma NOS
18423	Arterial insufficiency
38907	Other specified peripheral vascular disease
4325	Other specified peripheral vascular disease NOS
6827	Peripheral ischaemia
5702	Peripheral ischaemic vascular disease
105317	Peripheral arterial disease
3530	Peripheral vascular disease NOS
2760	Peripheral vascular disease NOS
39949	Gangrene of finger
23672	Gangrene of hand
5943	Other peripheral vascular disease
2066	Other bypass of femoral artery or popliteal artery NOS
9099	Other emergency bypass of femoral artery or popliteal artery
11766	Other emergency bypass of femoral artery
12331	Other bypass of popliteal artery
18060	Other bypass of femoral artery
21927	Other bypass of iliac artery
22016	Femoro-femoral prosthetic cross over graft
24097	Bypass popliteal artery by pop/pop a anast c vein graft NEC
24692	Other bypass of femoral artery or popliteal artery
27580	Bypass femoral artery by fem/pop art anast c prosthesis NEC
28030	Bypass femoral artery by fem/pop art anast c vein graft NEC
28616	Bypass iliac artery by iliac/femoral artery anastomosis NEC
30989	Femoro-femoral prosthetic cross over graft
32492	Bypass common iliac artery by aorta/com iliac art anast NEC
36065	Endarterectomy and patch repair of common iliac artery

36443	Bypass iliac artery by femoral/femoral art anastomosis NEC
37787	Other bypass of common femoral artery
38921	Other bypass of iliac artery NOS
39776	Other emergency bypass of popliteal artery
39877	Bypass femoral artery by fem/tib art anast c prosthesis NEC
40732	Other bypass of superficial femoral artery
41768	Ilio-femoral prosthetic cross over graft
41823	Bypass femoral artery by fem/tib art anast c vein graft NEC
42115	Bypass popliteal artery by pop/fem artery anastomosis NEC
42640	Other bypass of femoral artery or popliteal artery OS
43648	Emergency femoro-femoral prosthetic cross over graft
43651	Emerg bypass femoral art by fem/pop art anast c prosth NEC
44250	Emerg bypass iliac art by iliac/femoral art anastomosis NEC
45428	Bypass femoral artery by femoral/femoral art anastomosis NEC
48700	Bypass popliteal artery by pop/tib a anast c vein graft NEC
48755	Emerg bypass bifurc aorta by anast aorta to femoral artery
48939	Emerg bypass femoral artery by fem/fem art anastomosis NEC
52342	Emerg bypass femoral art by fem/pop a anast c vein graft NEC
52357	Other specified other bypass of iliac artery
53675	Bypass femoral artery by fem/peron a anast c vein graft NEC
55554	Bypass leg artery by aorta/com femoral art anastomosis NEC
60465	Bypass popliteal artery by pop/tib a anast c prosthesis NEC
60693	Emerg bypass pop art by pop/pop art anast c vein graft NEC
61974	Other bypass of femoral or popliteal artery by anastomosis
62775	Emerg bypass pop art by pop/peron art anast c vein graft NEC
63238	Other emergency bypass of deep femoral artery
64555	Bypass popliteal artery by pop/pop a anast c prosthesis NEC
65692	Other emergency bypass of femoral or popliteal artery OS
66804	Bypass leg artery by aorta/deep femoral art anastomosis NEC
66820	Emerg bypass femoral art by fem/tib art anast c prosth NEC
66879	Emerg bypass pop art by pop/tib art anast c vein graft NEC
66917	Emerg bypass leg artery by aorta/com fem art anastomosis NEC
67818	Emerg bypass popliteal art by pop/pop art anast c prosth NEC
67982	Bypass femoral artery by fem/peron a anast c prosthesis NEC
68141	Emerg bypass comm iliac art by aorta/com iliac art anast NEC
68320	Other emergency bypass of femoral or popliteal artery NOS
68412	Bypass popliteal art by pop/peron art anast c vein graft NEC
70922	Emerg bypass popliteal artery by pop/fem art anastomosis NEC
72448	Emerg bypass iliac artery by femoral/femoral art anast NEC
72491	Other emerg bypass femoral or popliteal art by anastomosis
96255	Emerg bypass femoral art by fem/tib a anast c vein graft NEC
97606	Other emergency bypass of superficial femoral artery
99676	Emerg bypass femoral art by fem/peron art anast c prosth NEC

100036	Bypass iliac artery by iliac/iliac artery anastomosis NEC
100113	Other emergency bypass of common femoral artery
101910	Other bypass of iliac artery by anastomosis
102031	Emerg bypass abdominal aorta by anastom aorta to aorta NEC
107158	Bypass popliteal artery by pop/peron art anast c prosth NEC

icd	icd_description
I70.1	Atherosclerosis of renal artery
I70.2	Atherosclerosis of arteries of extremities
I70.8	Atherosclerosis of other arteries
I70.9	Generalized and unspecified atherosclerosis
I73.1	Thromboangiitis obliterans [Buerger]
I73.8	Other specified peripheral vascular diseases
I73.9	Peripheral vascular disease, unspecified
I74.2	Embolism and thrombosis of arteries of upper extremities
I74.3	Embolism and thrombosis of arteries of lower extremities
I74.4	Embolism and thrombosis of arteries of extremities, unspec
I74.5	Embolism and thrombosis of iliac artery
I74.8	Embolism and thrombosis of other arteries
I74.9	Embolism and thrombosis of unspecified artery
I79.2	Peripheral angiopathy in diseases classified elsewhere
I79.8	Oth disord arteries, arterioles & capillaries in diseases CE

opcs	description
L50	Other emergency bypass of iliac artery
L501	Emergency bypass of common iliac artery by anastomosis of aorta to common iliac artery NEC
L502	Emergency bypass of iliac artery by anastomosis of aorta to external iliac artery NEC
L503	Emergency bypass of artery of leg by anastomosis of aorta to common femoral artery NEC
L504	Emergency bypass of artery of leg by anastomosis of aorta to deep femoral artery NEC
L505	Emergency bypass of iliac artery by anastomosis of iliac artery to iliac artery NEC
L506	Emergency bypass of artery of leg by anastomosis of iliac artery to femoral artery NEC
L508	Other specified other emergency bypass of iliac artery
L509	Unspecified other emergency bypass of iliac artery
L51	Other bypass of iliac artery
L511	Bypass of common iliac artery by anastomosis of aorta to common iliac artery NEC
L512	Bypass of iliac artery by anastomosis of aorta to external iliac artery NEC
L513	Bypass of artery of leg by anastomosis of aorta to common femoral artery NEC
L514	Bypass of artery of leg by anastomosis of aorta to deep femoral artery NEC

L515	Bypass of iliac artery by anastomosis of iliac artery to iliac artery NEC
L516	Bypass of artery of leg by anastomosis of iliac artery to femoral artery NEC
L518	Other specified other bypass of iliac artery
L519	Unspecified other bypass of iliac artery
L52	Reconstruction of iliac artery
L521	Endarterectomy of iliac artery and patch repair of iliac artery
L522	Endarterectomy of iliac artery NEC
L528	Other specified reconstruction of iliac artery
L529	Unspecified reconstruction of iliac artery
L531	Repair of iliac artery NEC
L532	Open embolectomy of iliac artery
L538	Other specified other open operations on iliac artery
L539	Unspecified other open operations on iliac artery
L54	Transluminal operations on iliac artery
L541	Percutaneous transluminal angioplasty of iliac artery
L542	Percutaneous transluminal embolectomy of iliac artery
L544	Percutaneous transluminal insertion of stent into iliac artery
L548	Other specified transluminal operations on iliac artery
L549	Unspecified transluminal operations on iliac artery
L58	Other emergency bypass of femoral artery
L581	Emergency bypass of femoral artery by anastomosis of femoral artery to femoral artery NEC
L582	Emergency bypass of femoral artery by anastomosis of femoral artery to popliteal artery using prosthesis NEC
L583	Emergency bypass of femoral artery by anastomosis of femoral artery to popliteal artery using vein graft NEC
L584	Emergency bypass of femoral artery by anastomosis of femoral artery to tibial artery using prosthesis NEC
L585	Emergency bypass of femoral artery by anastomosis of femoral artery to tibial artery using vein graft NEC
L586	Emergency bypass of femoral artery by anastomosis of femoral artery to peroneal artery using prosthesis NEC
L587	Emergency bypass of femoral artery by anastomosis of femoral artery to peroneal artery using vein graft NEC
L588	Other specified other emergency bypass of femoral artery
L589	Unspecified other emergency bypass of femoral artery
L59	Other bypass of femoral artery
L591	Bypass of femoral artery by anastomosis of femoral artery to femoral artery NEC
L592	Bypass of femoral artery by anastomosis of femoral artery to popliteal artery using prosthesis NEC
L593	Bypass of femoral artery by anastomosis of femoral artery to popliteal artery using vein graft NEC
L594	Bypass of femoral artery by anastomosis of femoral artery to tibial artery using prosthesis NEC
L595	Bypass of femoral artery by anastomosis of femoral artery to tibial artery using vein graft NEC
L596	Bypass of femoral artery by anastomosis of femoral artery to peroneal artery using prosthesis NEC
L597	Bypass of femoral artery by anastomosis of femoral artery to peroneal artery using vein graft NEC
L598	Other specified other bypass of femoral artery
L599	Unspecified other bypass of femoral artery

L60	Reconstruction of femoral artery
L601	Endarterectomy of femoral artery and patch repair of femoral artery
L602	Endarterectomy of femoral artery NEC
L603	Profundoplasty of femoral artery and patch repair of deep femoral artery
L604	Profundoplasty of femoral artery NEC
L608	Other specified reconstruction of femoral artery
L609	Unspecified reconstruction of femoral artery
L621	Repair of femoral artery NEC
L622	Open embolectomy of femoral artery
L628	Other specified other open operations on femoral artery
L629	Unspecified other open operations on femoral artery
L631	Percutaneous transluminal angioplasty of femoral artery
L632	Percutaneous transluminal embolectomy of femoral artery
L633	Percutaneous transluminal embolisation of femoral artery
L635	Percutaneous transluminal insertion of stent into femoral artery
L638	Other specified transluminal operations on femoral artery
L639	Unspecified transluminal operations on femoral artery
L651	Revision of reconstruction involving aorta
L652	Revision of reconstruction involving iliac artery
L653	Revision of reconstruction involving femoral artery

Heart failure

Medcode	Readterm
23707	Acute congestive heart failure
398	Congestive heart failure
884	Left ventricular failure
1223	Cardiac failure
2062	Heart failure
2906	Congestive cardiac failure
4024	Heart failure NOS
5255	Acute left ventricular failure
5942	Impaired left ventricular function
7251	Impaired left ventricular function
8966	Left ventricular systolic dysfunction
9524	Biventricular failure
9913	Heart failure confirmed
10079	Right heart failure
10154	Right ventricular failure
11424	Compensated cardiac failure
12550	Left ventricular diastolic dysfunction
13189	New York Heart Association classification - class II
17278	Cardiac failure NOS
19066	New York Heart Association classification - class III
19380	Heart failure review completed
21837	Hypertensive heart&renal dis wth (congestive) heart failure
23481	Asthma - cardiac
24503	Cardiac failure therapy
26242	New York Heart Assoc classification heart failure symptoms
27884	Decompensated cardiac failure
27964	Acute heart failure
32671	Chronic congestive heart failure
32898	Admit heart failure emergency
46672	New York Heart Assoc classification heart failure symptoms
46912	H/O: Heart failure in last year
51214	New York Heart Association classification - class IV
52127	Benign hypertensive heart disease with CCF
57987	Hyperten heart&renal dis+both(congestv)heart and renal fail
62718	Hypertensive heart disease NOS with CCF
66306	Heart failure as a complication of care
68682	Cardiac insufficiency as a complication of care
72668	Malignant hypertensive heart disease with CCF
94870	Congestive heart failure due to valvular disease
96799	Post cardiac operation heart failure NOS

103732	Has heart failure management plan
104275	Right ventricular failure
105542	Preferred place of care for next exacerbation heart failure
106897	Heart failure with preserved ejection fraction
104333	Diastolic dysfunction
104876	Right ventricular diastolic dysfunction
6434	Paroxysmal nocturnal dyspnoea
107397	Left ventricular cardiac dysfunction
15058	H/O: heart failure
100784	Heart failure resolved
101137	HFNEF - heart failure with normal ejection fraction
101138	Heart failure with normal ejection fraction
18853	New York Heart Association classification - class I
17851	Heart failure follow-up
30779	Heart failure annual review
83502	Heart failure 6 month review
106198	Heart failure self-management plan agreed
32945	Heart failure care plan discussed with patient
7321	Pulmonary oedema NOS
26082	Chronic pulmonary oedema
39706	Postoperative pulmonary oedema
43618	Pulmonary oedema - acute
48466	Acute oedema of lung, unspecified
102627	Worsening pulmonary oedema
558	Acute pulmonary oedema unspecified
1585	Pulmonary congestion
5155	O/E - pulmonary oedema
5293	Acute pulmonary oedema NOS
22262	Rheumatic left ventricular failure
20324	[D]Cardiorespiratory failure
8464	Acute cor pulmonale
5695	Chronic cor pulmonale

icd	icd_description
I11.0	Hypertensive heart disease with (congestive) heart failure
I13.0	Hypertens heart and renal dis with (conges) heart failure
I13.2	Hyper heart and renal dis both (cong) heart and renal fail
I50	Heart failure
I50.0	Congestive heart failure
I50.1	Left ventricular failure
I50.9	Heart failure, unspecified

Cerebrovascular disease

Medcode	Readterm
504	Transient cerebral ischaemia
569	Infarction - cerebral
1195	Amaurosis fugax
1298	CVA unspecified
1433	Transient ischaemic attack
1469	Stroke and cerebrovascular accident unspecified
1786	Subarachnoid haemorrhage
1895	Transient cerebral ischaemia NOS
2156	Stenosis, carotid artery
2418	Cerebrovascular disease
2652	Carotid artery stenosis
3149	Cerebral infarction NOS
3535	Intracerebral haemorrhage NOS
3585	Thrombosis lateral sinus
3979	Hypertensive encephalopathy
4152	Thrombosis, carotid artery
4240	Carotid artery occlusion
4273	Subdural haemorrhage - nontraumatic
4635	Cerebral aneurysm, nonruptured
5051	Intracerebral haemorrhage
5095	Binswanger's disease
5184	Precerebral atherosclerosis
5185	Lateral medullary syndrome
5268	Insufficiency - basilar artery
5363	CVA - cerebral artery occlusion
5365	Cerebral artery aneurysm operations
5602	Cerebellar infarction
5871	H/O: stroke
6116	CVA - Cerebrovascular accident unspecified
6155	Stroke due to cerebral arterial occlusion
6228	Sequelae of stroke
6253	Stroke unspecified
6305	H/O: CVA
6489	Transient global amnesia
6960	CVA - cerebrovascular accid due to intracerebral haemorrhage
7138	[V]Personal history of cerebrovascular accident (CVA)
7780	Left sided CVA
7912	Pontine haemorrhage
8443	Brain stem stroke syndrome
8837	Cerebral arterial occlusion

9696	Subarachnoid haemorrhage from posterior communicating artery
9943	Cereb autosom dominant arteriop subcort infarcts leukoenceph
9985	Left sided cerebral infarction
10062	Cerebrovascular disease NOS
10189	Cerebral amyloid angiopathy
10504	Right sided cerebral infarction
10625	Clipping of aneurysm of cerebral artery
11171	Cerebral atherosclerosis
12555	Generalised ischaemic cerebrovascular disease NOS
12833	Right sided CVA
13564	Cerebellar haemorrhage
13567	H/O: TIA
13577	Other cerebrovascular disease
13707	Stroke / transient ischaemic attack referral
15019	Cerebral embolism
15252	Brainstem infarction NOS
15788	Transient cerebral ischaemia NOS
16507	Intermittent cerebral ischaemia
16517	Cerebral thrombosis
16554	H/O sub-arachnoid haemorrhage
17322	Cerebellar stroke syndrome
17326	Subarachnoid haemorrh from intracranial artery
17734	Subdural haematoma - nontraumatic
18604	Stroke due to intracerebral haemorrhage
18689	Middle cerebral artery syndrome
18996	Transient global amnesia
19004	TGA - Transient global amnesia
19201	Right sided intracerebral haemorrhage, unspecified
19260	Posterior cerebral artery syndrome
19280	Anterior cerebral artery syndrome
19348	[V]Personal history of stroke
19354	Other transient cerebral ischaemia
19412	Subarachnoid haemorrhage from middle cerebral artery
20161	Thrombosis of superior longitudinal sinus
20284	Intracranial haemorrhage NOS
21118	Vertebro-basilar artery syndrome
22006	Thrombosis cavernous sinus
22018	Dissection of cerebral arteries, nonruptured
22400	Cerebral arteritis
23361	Late effects of cerebrovascular disease
23580	Subarachnoid haemorrhage NOS
23671	Cerebral infarct due to thrombosis of precerebral arteries
23942	Basilar artery syndrome

24385	Chronic cerebral ischaemia
24424	Congenital cerebral arteriovenous aneurysm
24446	Cerebral infarction due to embolism of precerebral arteries
25615	Brainstem infarction
26424	Infarction of basal ganglia
27975	Cerebral infarction due to embolism of cerebral arteries
28278	Transient global amnesia
28309	Thrombosis transverse sinus
28314	Left sided intracerebral haemorrhage, unspecified
29939	Ruptured berry aneurysm
30045	External capsule haemorrhage
30202	Intracerebral haemorrhage
30502	CVA prevention
31060	Intracerebral haemorrhage in hemisphere, unspecified
31390	Thrombosis of central nervous system venous sinuses
31595	Cortical haemorrhage
31704	Occlusion/stenosis cerebral arts not result cerebral infarct
31805	Other and unspecified intracranial haemorrhage
31941	Rupture of syphilitic cerebral aneurysm
32310	Moyamoya disease
32447	Basilar artery occlusion
33377	Vertebral artery syndrome
33499	Pure motor lacunar syndrome
33543	Cerebrl infarctn due/unspcf occlusn or sten/cerebrl artrs
34117	Other cerebrovascular disease OS
34135	H/O: CVA/stroke
34758	Cerebral embolus
36178	Extradural haemorrhage - nontraumatic
36717	Cerebral infarction due to thrombosis of cerebral arteries
37493	Other cerebrovascular disease NOS
37947	Nonpyogenic venous sinus thrombosis
39344	Cereb infarct due cerebral venous thrombosis, nonpyogenic
39403	Sequelae of cerebral infarction
40053	Generalised ischaemic cerebrovascular disease NOS
40338	Internal capsule haemorrhage
40758	Cereb infarct due unsp occlus/stenos precerebr arteries
40847	Vertebral artery occlusion
41180	Thrombophlebitis of central nervous system venous sinuses
41577	H/O: cerebrovascular disease
41910	Subarachnoid haemorrhage from basilar artery
42331	Subarachnoid haemorrhage from anterior communicating artery
43451	Sequelae of other nontraumatic intracranial haemorrhage
44740	Sequelae of subarachnoid haemorrhage

44765	Carotid artery syndrome hemispheric
45781	Precerebral arterial occlusion
46316	Basal nucleus haemorrhage
47607	CVA - cerebrovascular accident in the puerperium
47642	Wallenberg syndrome
48149	Sequelae of intracerebral haemorrhage
49715	Phlebitis and thrombophlebitis of intracranial sinuses
50594	Multiple and bilateral precerebral artery syndromes
50725	Listerial cerebral arteritis
51138	Sequelae/other + unspecified cerebrovascular diseases
51311	Other specified cerebrovascular disease
51326	Other precerebral artery occlusion
51759	Occlusion and stenosis of middle cerebral artery
51767	Pure sensory lacunar syndrome
53745	[X]Other cerebral infarction
53810	[X]Other intracerebral haemorrhage
54744	Cerebral degeneration due to cerebrovascular disease
55602	Occlusion and stenosis of cerebellar arteries
55883	Thrombophlebitis of cavernous sinus
55885	Embolism cavernous sinus
56007	Subarachnoid haemorrhage from carotid siphon and bifurcation
57315	Intracerebral haemorrhage
57495	Infarction - precerebral
57527	Occlusion and stenosis of anterior cerebral artery
60692	Subarachnoid haemorrhage from vertebral artery
61366	Thrombosis of central nervous system venous sinus NOS
63746	[X]Other transnt cerebral ischaemic attacks+related syndroms
63830	Stenosis of precerebral arteries
65745	[X]Other subarachnoid haemorrhage
65770	Occlusion and stenosis of posterior cerebral artery
65855	Phlebitis or thrombophlebitis of CNS venous sinus NOS
66873	H/O: Stroke in last year
68194	Binswanger's encephalopathy
70455	Thrombophlebitis of superior longitudinal venous sinus
70536	Acute cerebrovascular insufficiency NOS
71274	Occlusion+stenosis of multiple and bilat cerebral arteries
71585	Precerebral artery occlusion NOS
73901	[X]Cerebrovascular diseases
84404	Embolism transverse sinus
90572	[X]Occlusion and stenosis of other precerebral arteries
91627	[X]Cerebrl infarctn due/unspcf occlusn or sten/cerebrl artrs
92036	[X]Occlusion and stenosis of other cerebral arteries
93459	[X]Other lacunar syndromes

94482	[X]Cereb infarct due unsp occlus/stenos precerebr arteries
95347	[X]Other vascular syndroms/brain in cerebrovasculr diseases
96630	[X]Intracerebral haemorrhage in hemisphere, unspecified
98188	Small vessel cerebrovascular disease
98642	Multiple and bilateral precerebral arterial occlusion
99367	[X]Other cerebrovascular disorders in diseases CE
100639	Central post-stroke pain
101251	[V]Personal history of transient ischaemic attack
101733	Cerebral vein thrombosis
101824	Adrenocortical haemorrhage
105202	H/O amaurosis fugax
107440	Lobar cerebral haemorrhage
107725	Reversible cerebral vasoconstriction syndrome
108630	[X]Subarachnoid haemorrh from intracranial artery, unspecif
108668	[X]Subarachnoid haemorrhage from other intracranial arteries
36568	Embolism of central nervous system venous sinus
100015	Transient ischaemic attack clinical management plan
18686	Stroke/CVA annual review
55351	Delivery of rehabilitation for stroke
104505	Stroke initial post discharge review
105100	Stroke 6 month review
107195	Stroke self-management plan agreed
107886	Stroke annual review
109743	Stroke self-management plan review

icd	icd_description
G45	Transient cerebral ischaemic attacks and related syndromes
G45.0	Vertebro-basilar artery syndrome
G45.1	Carotid artery syndrome (hemispheric)
G45.2	Multiple and bilateral precerebral artery syndromes
G45.3	Amaurosis fugax
G45.4	Transient global amnesia
G45.8	Other transient cerebral ischaemic attacks and related synd
G45.9	Transient cerebral ischaemic attack, unspecified
G46	Vascular syndromes of brain in cerebrovascular diseases
G46.0	Middle cerebral artery syndrome
G46.1	Anterior cerebral artery syndrome
G46.2	Posterior cerebral artery syndrome
G46.3	Brain stem stroke syndrome
G46.4	Cerebellar stroke syndrome
G46.5	Pure motor lacunar syndrome
G46.6	Pure sensory lacunar syndrome

G46.7	Other lacunar syndromes
G46.8	Oth vascular syndromes of brain in cerebrovascular dis
I60	Subarachnoid haemorrhage
I60.0	Subarachnoid haemorrhage from carotid siphon and bifurcation
I60.1	Subarachnoid haemorrhage from middle cerebral artery
I60.2	Subarachnoid haemorrhage from anterior communicating artery
I60.3	Subarachnoid haemorrhage from posterior communicating artery
I60.4	Subarachnoid haemorrhage from basilar artery
I60.5	Subarachnoid haemorrhage from vertebral artery
I60.6	Subarachnoid haemorrhage from other intracranial arteries
I60.7	Subarachnoid haemorrhage from intracranial artery, unspec
I60.8	Other subarachnoid haemorrhage
I60.9	Subarachnoid haemorrhage, unspecified
I61	Intracerebral haemorrhage
I61.0	Intracerebral haemorrhage in hemisphere, subcortical
I61.1	Intracerebral haemorrhage in hemisphere, cortical
I61.2	Intracerebral haemorrhage in hemisphere, unspecified
I61.3	Intracerebral haemorrhage in brain stem
I61.4	Intracerebral haemorrhage in cerebellum
I61.5	Intracerebral haemorrhage, intraventricular
I61.6	Intracerebral haemorrhage, multiple localized
I61.8	Other intracerebral haemorrhage
I61.9	Intracerebral haemorrhage, unspecified
I62	Other nontraumatic intracranial haemorrhage
I62.0	Subdural haemorrhage (acute)(nontraumatic)
I62.1	Nontraumatic extradural haemorrhage
I62.9	Intracranial haemorrhage (nontraumatic), unspecified
I63	Cerebral infarction
I63.0	Cerebral infarct due to thrombosis of precerebral arteries
I63.1	Cerebral infarction due to embolism of precerebral arteries
I63.2	Cereb infarct due unsp occlusion or stenos precerebrl arts
I63.3	Cerebral infarction due to thrombosis of cerebral arteries
I63.4	Cerebral infarction due to embolism of cerebral arteries
I63.5	Cerebrl infarct due unspec occlusion or stenos cerebrl arts
I63.6	Cereb infarct due cerebral venous thrombosis, nonpyogenic
I63.8	Other cerebral infarction
I63.9	Cerebral infarction, unspecified
I64	Stroke, not specified as haemorrhage or infarction
I65	Occlusion/stenos precerebral arts not result cerebrl infarct
I65.0	Occlusion and stenosis of vertebral artery
I65.1	Occlusion and stenosis of basilar artery
I65.2	Occlusion and stenosis of carotid artery
I65.3	Occlusion and stenosis of multip and bilat precerebrl arts

I65.8	Occlusion and stenosis of other precerebral artery
I65.9	Occlusion and stenosis of unspecified precerebral artery
I66	Occlusion/stenosis cerebral arts not result cerebral infarct
I66.0	Occlusion and stenosis of middle cerebral artery
I66.1	Occlusion and stenosis of anterior cerebral artery
I66.2	Occlusion and stenosis of posterior cerebral artery
I66.3	Occlusion and stenosis of cerebellar arteries
I66.4	Occlusion and stenosis of multiple and bilat cerebrl arts
I66.8	Occlusion and stenosis of other cerebral artery
I66.9	Occlusion and stenosis of unspecified cerebral artery
I67	Other cerebrovascular diseases
I67.0	Dissection of cerebral arteries, nonruptured
I67.1	Cerebral aneurysm, nonruptured
I67.2	Cerebral atherosclerosis
I67.3	Progressive vascular leukoencephalopathy
I67.4	Hypertensive encephalopathy
I67.5	Moyamoya disease
I67.6	Nonpyogenic thrombosis of intracranial venous system
I67.7	Cerebral arteritis, not elsewhere classified
I67.8	Other specified cerebrovascular diseases
I67.9	Cerebrovascular disease, unspecified
I68	Cerebrovascular disorders in diseases classified elsewhere
I68.0	Cerebral amyloid angiopathy
I68.1	Cerebral arteritis in infect & parasit dis classif elsewh
I68.2	Cerebral arteritis in other diseases classified elsewhere
I68.8	Other cerebrovascular disorders in diseases EC
I69	Sequelae of cerebrovascular disease
I69.0	Sequelae of subarachnoid haemorrhage
I69.1	Sequelae of intracerebral haemorrhage
I69.2	Sequelae of other nontraumatic intracranial haemorrhage
I69.3	Sequelae of cerebral infarction
I69.4	Sequelae of stroke, not spec as haemorrhage or infarction
I69.8	Sequelae of other and unspecified cerebrovascular diseases

Terminal disease

medcode	readterm
26353	Terminal illness - late stage
100660	Last days of life
108509	Palliative Care Outcomes Collaboration Assessment Toolkit
108547	Palliative Care Problem Severity Score
106667	Prescription of palliative care anticipatory medication
6664	Terminal care
106773	End of life care
49651	Specialist palliative care treatment - daycare
26354	Specialist palliative care treatment - outpatient
18551	Palliative treatment
12739	On gold standards palliative care framework
100607	GSF supportive care stage 1 - advancing disease
100525	GSF supportive care stage 2 - increasing decline
101636	GSF supportv care stge 3 - last days: cat C - wks prognosis
100466	GSF supportv care stge 3 - last days: cat D - days prognosis
102415	GSF supportv care stge 3 - last days: cat B - mth prognosis
105306	GSF prognostic indicator stage B (green) - months prognosis
105314	GSF prognostic indicator stage C (yellow) - weeks prognosis
105447	GSF prognostic indicator stage D (red) - days prognosis
74909	Liverpool care pathway for the dying
106662	Integrated care priorities for end of life
99766	Has end of life advance care plan
109281	End of life advance care plan
104282	On Liverpool care pathway for the dying
105222	End of life care pathway
9755	Referral to palliative care service
34531	Refer for terminal care
22288	Referred to community specialist palliative care team
103941	Referral to community palliative care team declined
105961	Patient held palliative care record
105908	Planned palliative oncology treatment
106204	Planned supportive care for terminal illness
107583	Current supportive care for terminal illness
105975	Current palliative oncology treatment
6924	DS 1500 Disability living allowance completed
105757	Ambulance service notified of patient on EoL care register
105391	Palliative care handover form completed
100126	On end of life care register
104463	Under care of palliative care service
105214	Under care of palliative care specialist nurse

105877	Under care of physician
106582	Under care of palliative care physician
7060	[V]Palliative care
8976	Terminal illness
29285	Radiotherapy-tumour palliation
103607	Counselling for end of life issues
98251	Anticipatory palliative care
11318	Community specialist palliative care
10019	Specialist palliative care
10784	Specialist palliative care treatment - inpatient
97066	Issue of palliative care anticipatory medication box
97280	Issue of palliative care just in case box
98521	Management of multiple sclerosis in palliative phase
19458	Palliative care plan review
103591	GSF advance care plan discussion statement
26352	Refer to terminal care consult
50291	Discharged from community specialist palliative care team
106695	Discharge from palliative care service
73313	Palliative medicine
98441	Notif to primary care OOHS of palliative care plan in place
97051	Palliative care - enhanced services administration
105508	Healthcare prof would not be surprised (GSF surprise qu)
48775	Under the care of community palliative care team
96936	Seen by palliative care service
103569	Has end of life care pathway key general practitioner
105849	Has end of life care pathway key nurse
102536	Has end of life care pathway key worker
107660	Consnt apoint persn LPA persn welfar (MCA 05) shar EoLCC rec
9996	Palliative care
28899	Under care of palliative care physician
13628	Referral to palliative care physician
11978	Seen by palliative care physician

icd	icd_description
Z51.5	Palliative care

Charlson Comorbidity Index

medcode	Readterm	cci_1mi	cci_1ccf	cci_1pad	cci_1stroke	cci_1dement	cci_1resp	cci_1ctd	cci_1peptic	cci_1liver	cci_2hemi	cci_2renal	cci_2diabcomp	cci_2cancer	cci_2leuk	cci_2lymph	cci_3liver	cci_6mets	cci_6hiv
241	Acute myocardial infarction	1	0	0	0	0	0	0	0	0	0	0	0	0	0	0	0	0	0
1204	Heart attack	1	0	0	0	0	0	0	0	0	0	0	0	0	0	0	0	0	0
1677	MI - acute myocardial infarction	1	0	0	0	0	0	0	0	0	0	0	0	0	0	0	0	0	0
1678	Inferior myocardial infarction NOS	1	0	0	0	0	0	0	0	0	0	0	0	0	0	0	0	0	0
2491	Coronary thrombosis	1	0	0	0	0	0	0	0	0	0	0	0	0	0	0	0	0	0
3704	Acute subendocardial infarction	1	0	0	0	0	0	0	0	0	0	0	0	0	0	0	0	0	0
5387	Other specified anterior myocardial infarction	1	0	0	0	0	0	0	0	0	0	0	0	0	0	0	0	0	0
7783	ECG: myocardial infarction	1	0	0	0	0	0	0	0	0	0	0	0	0	0	0	0	0	0
8935	Acute inferolateral infarction	1	0	0	0	0	0	0	0	0	0	0	0	0	0	0	0	0	0
9507	Acute non-Q wave infarction	1	0	0	0	0	0	0	0	0	0	0	0	0	0	0	0	0	0
10562	Acute non-Q wave infarction	1	0	0	0	0	0	0	0	0	0	0	0	0	0	0	0	0	0
12139	Acute anterolateral infarction	1	0	0	0	0	0	0	0	0	0	0	0	0	0	0	0	0	0
12229	Acute ST segment elevation myocardial infarction	1	0	0	0	0	0	0	0	0	0	0	0	0	0	0	0	0	0
13566	Attack - heart	1	0	0	0	0	0	0	0	0	0	0	0	0	0	0	0	0	0
13571	Thrombosis - coronary	1	0	0	0	0	0	0	0	0	0	0	0	0	0	0	0	0	0
14658	Acute myocardial infarction NOS	1	0	0	0	0	0	0	0	0	0	0	0	0	0	0	0	0	0
14897	Anterior myocardial infarction NOS	1	0	0	0	0	0	0	0	0	0	0	0	0	0	0	0	0	0
14898	Lateral myocardial infarction NOS	1	0	0	0	0	0	0	0	0	0	0	0	0	0	0	0	0	0
15661	Dressler's syndrome	1	0	0	0	0	0	0	0	0	0	0	0	0	0	0	0	0	0
17133	Mural thrombosis	1	0	0	0	0	0	0	0	0	0	0	0	0	0	0	0	0	0
17872	Acute anteroseptal infarction	1	0	0	0	0	0	0	0	0	0	0	0	0	0	0	0	0	0
18842	Subsequent myocardial infarction	1	0	0	0	0	0	0	0	0	0	0	0	0	0	0	0	0	0
21854	Left ventricular thrombosis	1	0	0	0	0	0	0	0	0	0	0	0	0	0	0	0	0	0

23708	Atrial septal defect/curr comp folow acut myocardal infarct	1	0	0	0	0	0	0	0	0	0	0	0	0	0	0	0	0	0
23892	Posterior myocardial infarction NOS	1	0	0	0	0	0	0	0	0	0	0	0	0	0	0	0	0	0
24126	Haemopericardium/current comp folow acut myocard infarct	1	0	0	0	0	0	0	0	0	0	0	0	0	0	0	0	0	0
26966	ECG: S-T elevation	1	0	0	0	0	0	0	0	0	0	0	0	0	0	0	0	0	0
26972	ECG:posterior/inferior infarct	1	0	0	0	0	0	0	0	0	0	0	0	0	0	0	0	0	0
26975	ECG: antero-septal infarct.	1	0	0	0	0	0	0	0	0	0	0	0	0	0	0	0	0	0
28736	Acute atrial infarction	1	0	0	0	0	0	0	0	0	0	0	0	0	0	0	0	0	0
29553	Thrombosis atrium,auric append&vent/curr comp foll acute MI	1	0	0	0	0	0	0	0	0	0	0	0	0	0	0	0	0	0
29643	Acute inferoposterior infarction	1	0	0	0	0	0	0	0	0	0	0	0	0	0	0	0	0	0
29758	Acute transmural myocardial infarction of unspecif site	1	0	0	0	0	0	0	0	0	0	0	0	0	0	0	0	0	0
30330	Acute Q-wave infarct	1	0	0	0	0	0	0	0	0	0	0	0	0	0	0	0	0	0
30421	Cardiac rupture following myocardial infarction (MI)	1	0	0	0	0	0	0	0	0	0	0	0	0	0	0	0	0	0
32272	Postoperative myocardial infarction	1	0	0	0	0	0	0	0	0	0	0	0	0	0	0	0	0	0
32854	Acute posterolateral myocardial infarction	1	0	0	0	0	0	0	0	0	0	0	0	0	0	0	0	0	0
34803	Other acute myocardial infarction	1	0	0	0	0	0	0	0	0	0	0	0	0	0	0	0	0	0
35119	Post infarction pericarditis	1	0	0	0	0	0	0	0	0	0	0	0	0	0	0	0	0	0
36423	Certain current complication follow acute myocardial infarct	1	0	0	0	0	0	0	0	0	0	0	0	0	0	0	0	0	0
37657	Ventric septal defect/curr comp fol acut myocardal infarctn	1	0	0	0	0	0	0	0	0	0	0	0	0	0	0	0	0	0
38609	Subsequent myocardial infarction of inferior wall	1	0	0	0	0	0	0	0	0	0	0	0	0	0	0	0	0	0
39449	Coronary thrombosis not resulting in myocardial infarction	1	0	0	0	0	0	0	0	0	0	0	0	0	0	0	0	0	0
40429	Acute anteroapical infarction	1	0	0	0	0	0	0	0	0	0	0	0	0	0	0	0	0	0
40996	Percut translum coronary thrombolytic therapy- streptokinase	1	0	0	0	0	0	0	0	0	0	0	0	0	0	0	0	0	0
41221	Acute septal infarction	1	0	0	0	0	0	0	0	0	0	0	0	0	0	0	0	0	0
41835	Postoperative subendocardial myocardial infarction	1	0	0	0	0	0	0	0	0	0	0	0	0	0	0	0	0	0

45809	Subsequent myocardial infarction of anterior wall	1	0	0	0	0	0	0	0	0	0	0	0	0	0	0	0	0	0
46017	Other acute myocardial infarction NOS	1	0	0	0	0	0	0	0	0	0	0	0	0	0	0	0	0	0
46112	Postoperative transmural myocardial infarction anterior wall	1	0	0	0	0	0	0	0	0	0	0	0	0	0	0	0	0	0
46166	Subsequent myocardial infarction of unspecified site	1	0	0	0	0	0	0	0	0	0	0	0	0	0	0	0	0	0
46276	Postoperative transmural myocardial infarction inferior wall	1	0	0	0	0	0	0	0	0	0	0	0	0	0	0	0	0	0
52705	ECG: lateral infarction	1	0	0	0	0	0	0	0	0	0	0	0	0	0	0	0	0	0
55401	ECG: subendocardial infarct	1	0	0	0	0	0	0	0	0	0	0	0	0	0	0	0	0	0
59032	ECG: myocardial infarct NOS	1	0	0	0	0	0	0	0	0	0	0	0	0	0	0	0	0	0
59189	Ruptur cardiac wall w'out haemopericard/cur comp fol ac MI	1	0	0	0	0	0	0	0	0	0	0	0	0	0	0	0	0	0
59940	Ruptur chordae tendinae/curr comp fol acute myocard infarct	1	0	0	0	0	0	0	0	0	0	0	0	0	0	0	0	0	0
61670	Diab mellit insulin-glucose infus acute myocardial infarct	1	0	0	0	0	0	0	0	0	0	0	0	0	0	0	0	0	0
62626	Acute papillary muscle infarction	1	0	0	0	0	0	0	0	0	0	0	0	0	0	0	0	0	0
63467	True posterior myocardial infarction	1	0	0	0	0	0	0	0	0	0	0	0	0	0	0	0	0	0
68357	Microinfarction of heart	1	0	0	0	0	0	0	0	0	0	0	0	0	0	0	0	0	0
68748	Postoperative myocardial infarction, unspecified	1	0	0	0	0	0	0	0	0	0	0	0	0	0	0	0	0	0
69474	Rupture papillary muscle/curr comp fol acute myocard infarct	1	0	0	0	0	0	0	0	0	0	0	0	0	0	0	0	0	0
72562	Subsequent myocardial infarction of other sites	1	0	0	0	0	0	0	0	0	0	0	0	0	0	0	0	0	0
96838	[X]Acute transmural myocardial infarction of unspecif site	1	0	0	0	0	0	0	0	0	0	0	0	0	0	0	0	0	0
99991	[X]Subsequent myocardial infarction of unspecified site	1	0	0	0	0	0	0	0	0	0	0	0	0	0	0	0	0	0
106812	Postoperative transmural myocardial infarction unspec site	1	0	0	0	0	0	0	0	0	0	0	0	0	0	0	0	0	0
109035	[X]Subsequent myocardial infarction of other sites	1	0	0	0	0	0	0	0	0	0	0	0	0	0	0	0	0	0
23707	Acute congestive heart failure	0	1	0	0	0	0	0	0	0	0	0	0	0	0	0	0	0	0
398	Congestive heart failure	0	1	0	0	0	0	0	0	0	0	0	0	0	0	0	0	0	0
884	Left ventricular failure	0	1	0	0	0	0	0	0	0	0	0	0	0	0	0	0	0	0

1223	Cardiac failure	0	1	0	0	0	0	0	0	0	0	0	0	0	0	0	0	0	0
2062	Heart failure	0	1	0	0	0	0	0	0	0	0	0	0	0	0	0	0	0	0
2906	Congestive cardiac failure	0	1	0	0	0	0	0	0	0	0	0	0	0	0	0	0	0	0
4024	Heart failure NOS	0	1	0	0	0	0	0	0	0	0	0	0	0	0	0	0	0	0
5255	Acute left ventricular failure	0	1	0	0	0	0	0	0	0	0	0	0	0	0	0	0	0	0
5942	Impaired left ventricular function	0	1	0	0	0	0	0	0	0	0	0	0	0	0	0	0	0	0
7251	Impaired left ventricular function	0	1	0	0	0	0	0	0	0	0	0	0	0	0	0	0	0	0
8966	Left ventricular systolic dysfunction	0	1	0	0	0	0	0	0	0	0	0	0	0	0	0	0	0	0
9524	Biventricular failure	0	1	0	0	0	0	0	0	0	0	0	0	0	0	0	0	0	0
9913	Heart failure confirmed	0	1	0	0	0	0	0	0	0	0	0	0	0	0	0	0	0	0
10079	Right heart failure	0	1	0	0	0	0	0	0	0	0	0	0	0	0	0	0	0	0
10154	Right ventricular failure	0	1	0	0	0	0	0	0	0	0	0	0	0	0	0	0	0	0
11424	Compensated cardiac failure	0	1	0	0	0	0	0	0	0	0	0	0	0	0	0	0	0	0
12550	Left ventricular diastolic dysfunction	0	1	0	0	0	0	0	0	0	0	0	0	0	0	0	0	0	0
13189	New York Heart Association classification - class II	0	1	0	0	0	0	0	0	0	0	0	0	0	0	0	0	0	0
17278	Cardiac failure NOS	0	1	0	0	0	0	0	0	0	0	0	0	0	0	0	0	0	0
19066	New York Heart Association classification - class III	0	1	0	0	0	0	0	0	0	0	0	0	0	0	0	0	0	0
19380	Heart failure review completed	0	1	0	0	0	0	0	0	0	0	0	0	0	0	0	0	0	0
21837	Hypertensive heart&renal dis wth (congestive) heart failure	0	1	0	0	0	0	0	0	0	0	0	0	0	0	0	0	0	0
23481	Asthma - cardiac	0	1	0	0	0	0	0	0	0	0	0	0	0	0	0	0	0	0
24503	Cardiac failure therapy	0	1	0	0	0	0	0	0	0	0	0	0	0	0	0	0	0	0
26242	New York Heart Assoc classification heart failure symptoms	0	1	0	0	0	0	0	0	0	0	0	0	0	0	0	0	0	0
27884	Decompensated cardiac failure	0	1	0	0	0	0	0	0	0	0	0	0	0	0	0	0	0	0
27964	Acute heart failure	0	1	0	0	0	0	0	0	0	0	0	0	0	0	0	0	0	0
32671	Chronic congestive heart failure	0	1	0	0	0	0	0	0	0	0	0	0	0	0	0	0	0	0
32898	Admit heart failure emergency	0	1	0	0	0	0	0	0	0	0	0	0	0	0	0	0	0	0

46672	New York Heart Assoc classification heart failure symptoms	0	1	0	0	0	0	0	0	0	0	0	0	0	0	0	0	0	0
46912	H/O: Heart failure in last year	0	1	0	0	0	0	0	0	0	0	0	0	0	0	0	0	0	0
51214	New York Heart Association classification - class IV	0	1	0	0	0	0	0	0	0	0	0	0	0	0	0	0	0	0
52127	Benign hypertensive heart disease with CCF	0	1	0	0	0	0	0	0	0	0	0	0	0	0	0	0	0	0
57987	Hyperten heart&renal dis+both(congestv)heart and renal fail	0	1	0	0	0	0	0	0	0	0	0	0	0	0	0	0	0	0
62718	Hypertensive heart disease NOS with CCF	0	1	0	0	0	0	0	0	0	0	0	0	0	0	0	0	0	0
66306	Heart failure as a complication of care	0	1	0	0	0	0	0	0	0	0	0	0	0	0	0	0	0	0
68682	Cardiac insufficiency as a complication of care	0	1	0	0	0	0	0	0	0	0	0	0	0	0	0	0	0	0
72668	Malignant hypertensive heart disease with CCF	0	1	0	0	0	0	0	0	0	0	0	0	0	0	0	0	0	0
94870	Congestive heart failure due to valvular disease	0	1	0	0	0	0	0	0	0	0	0	0	0	0	0	0	0	0
96799	Post cardiac operation heart failure NOS	0	1	0	0	0	0	0	0	0	0	0	0	0	0	0	0	0	0
103732	Has heart failure management plan	0	1	0	0	0	0	0	0	0	0	0	0	0	0	0	0	0	0
104275	Right ventricular failure	0	1	0	0	0	0	0	0	0	0	0	0	0	0	0	0	0	0
105542	Preferred place of care for next exacerbation heart failure	0	1	0	0	0	0	0	0	0	0	0	0	0	0	0	0	0	0
106897	Heart failure with preserved ejection fraction	0	1	0	0	0	0	0	0	0	0	0	0	0	0	0	0	0	0
104333	Diastolic dysfunction	0	1	0	0	0	0	0	0	0	0	0	0	0	0	0	0	0	0
104876	Right ventricular diastolic dysfunction	0	1	0	0	0	0	0	0	0	0	0	0	0	0	0	0	0	0
6434	Paroxysmal nocturnal dyspnoea	0	1	0	0	0	0	0	0	0	0	0	0	0	0	0	0	0	0
107397	Left ventricular cardiac dysfunction	0	1	0	0	0	0	0	0	0	0	0	0	0	0	0	0	0	0
15058	H/O: heart failure	0	1	0	0	0	0	0	0	0	0	0	0	0	0	0	0	0	0
100784	Heart failure resolved	0	1	0	0	0	0	0	0	0	0	0	0	0	0	0	0	0	0
101137	HFNEF - heart failure with normal ejection fraction	0	1	0	0	0	0	0	0	0	0	0	0	0	0	0	0	0	0
101138	Heart failure with normal ejection fraction	0	1	0	0	0	0	0	0	0	0	0	0	0	0	0	0	0	0
18853	New York Heart Association classification - class I	0	1	0	0	0	0	0	0	0	0	0	0	0	0	0	0	0	0

17851	Heart failure follow-up	0	1	0	0	0	0	0	0	0	0	0	0	0	0	0	0	0	0
30779	Heart failure annual review	0	1	0	0	0	0	0	0	0	0	0	0	0	0	0	0	0	0
83502	Heart failure 6 month review	0	1	0	0	0	0	0	0	0	0	0	0	0	0	0	0	0	0
106198	Heart failure self-management plan agreed	0	1	0	0	0	0	0	0	0	0	0	0	0	0	0	0	0	0
32945	Heart failure care plan discussed with patient	0	1	0	0	0	0	0	0	0	0	0	0	0	0	0	0	0	0
7321	Pulmonary oedema NOS	0	1	0	0	0	0	0	0	0	0	0	0	0	0	0	0	0	0
26082	Chronic pulmonary oedema	0	1	0	0	0	0	0	0	0	0	0	0	0	0	0	0	0	0
39706	Postoperative pulmonary oedema	0	1	0	0	0	0	0	0	0	0	0	0	0	0	0	0	0	0
43618	Pulmonary oedema - acute	0	1	0	0	0	0	0	0	0	0	0	0	0	0	0	0	0	0
48466	Acute oedema of lung, unspecified	0	1	0	0	0	0	0	0	0	0	0	0	0	0	0	0	0	0
102627	Worsening pulmonary oedema	0	1	0	0	0	0	0	0	0	0	0	0	0	0	0	0	0	0
558	Acute pulmonary oedema unspecified	0	1	0	0	0	0	0	0	0	0	0	0	0	0	0	0	0	0
1585	Pulmonary congestion	0	1	0	0	0	0	0	0	0	0	0	0	0	0	0	0	0	0
5155	O/E - pulmonary oedema	0	1	0	0	0	0	0	0	0	0	0	0	0	0	0	0	0	0
5293	Acute pulmonary oedema NOS	0	1	0	0	0	0	0	0	0	0	0	0	0	0	0	0	0	0
22262	Rheumatic left ventricular failure	0	1	0	0	0	0	0	0	0	0	0	0	0	0	0	0	0	0
20324	[D]Cardiorespiratory failure	0	1	0	0	0	0	0	0	0	0	0	0	0	0	0	0	0	0
8464	Acute cor pulmonale	0	1	0	0	0	0	0	0	0	0	0	0	0	0	0	0	0	0
5695	Chronic cor pulmonale	0	1	0	0	0	0	0	0	0	0	0	0	0	0	0	0	0	0
16521	Dissecting aortic aneurysm	0	0	1	0	0	0	0	0	0	0	0	0	0	0	0	0	0	0
102725	[X]Aortic aneurysm of unspecified site, ruptured	0	0	1	0	0	0	0	0	0	0	0	0	0	0	0	0	0	0
101195	Aneurysm of suprarenal aorta	0	0	1	0	0	0	0	0	0	0	0	0	0	0	0	0	0	0
1735	Aortic aneurysm	0	0	1	0	0	0	0	0	0	0	0	0	0	0	0	0	0	0
6872	Aortic aneurysm NOS	0	0	1	0	0	0	0	0	0	0	0	0	0	0	0	0	0	0
16034	Aortic aneurysm without mention of rupture NOS	0	0	1	0	0	0	0	0	0	0	0	0	0	0	0	0	0	0
102719	[X]Aortic aneurysm of unspecified site, nonruptured	0	0	1	0	0	0	0	0	0	0	0	0	0	0	0	0	0	0

17345	AAA - Abdominal aortic aneurysm without mention of rupture	0	0	1	0	0	0	0	0	0	0	0	0	0	0	0	0	0	0
17767	Abdominal aortic aneurysm which has ruptured	0	0	1	0	0	0	0	0	0	0	0	0	0	0	0	0	0	0
1867	Abdominal aortic aneurysm without mention of rupture	0	0	1	0	0	0	0	0	0	0	0	0	0	0	0	0	0	0
1318	Aortic atherosclerosis	0	0	1	0	0	0	0	0	0	0	0	0	0	0	0	0	0	0
31900	Aortic bifurcation syndrome	0	0	1	0	0	0	0	0	0	0	0	0	0	0	0	0	0	0
98277	Aortic root dilatation	0	0	1	0	0	0	0	0	0	0	0	0	0	0	0	0	0	0
16993	H/O: aortic aneurysm	0	0	1	0	0	0	0	0	0	0	0	0	0	0	0	0	0	0
28109	Inflammatory abdominal aortic aneurysm	0	0	1	0	0	0	0	0	0	0	0	0	0	0	0	0	0	0
101379	Infrarenal abdominal aortic aneurysm	0	0	1	0	0	0	0	0	0	0	0	0	0	0	0	0	0	0
45521	Juxtarenal aortic aneurysm	0	0	1	0	0	0	0	0	0	0	0	0	0	0	0	0	0	0
9759	Leaking abdominal aortic aneurysm	0	0	1	0	0	0	0	0	0	0	0	0	0	0	0	0	0	0
13572	Ruptured abdominal aortic aneurysm	0	0	1	0	0	0	0	0	0	0	0	0	0	0	0	0	0	0
15304	Ruptured aortic aneurysm NOS	0	0	1	0	0	0	0	0	0	0	0	0	0	0	0	0	0	0
63920	Ruptured suprarenal aortic aneurysm	0	0	1	0	0	0	0	0	0	0	0	0	0	0	0	0	0	0
16800	Ruptured thoracic aortic aneurysm	0	0	1	0	0	0	0	0	0	0	0	0	0	0	0	0	0	0
27563	Thoracic aortic aneurysm which has ruptured	0	0	1	0	0	0	0	0	0	0	0	0	0	0	0	0	0	0
23532	Thoracic aortic aneurysm without mention of rupture	0	0	1	0	0	0	0	0	0	0	0	0	0	0	0	0	0	0
11430	Thoracoabdominal aortic aneurysm, ruptured	0	0	1	0	0	0	0	0	0	0	0	0	0	0	0	0	0	0
40787	Thoracoabdominal aortic aneurysm, without mention of rupture	0	0	1	0	0	0	0	0	0	0	0	0	0	0	0	0	0	0
105621	[X]Aneurysm of aorta in diseases classified elsewhere	0	0	1	0	0	0	0	0	0	0	0	0	0	0	0	0	0	0
44835	Embolism and thrombosis of a leg artery NOS	0	0	1	0	0	0	0	0	0	0	0	0	0	0	0	0	0	0
30495	Embolism and thrombosis of an arm artery NOS	0	0	1	0	0	0	0	0	0	0	0	0	0	0	0	0	0	0
44085	Embolism and thrombosis of an arm or leg artery	0	0	1	0	0	0	0	0	0	0	0	0	0	0	0	0	0	0
41597	Embolism and thrombosis of other arteries NOS	0	0	1	0	0	0	0	0	0	0	0	0	0	0	0	0	0	0

32235	Embolism and thrombosis of other specified artery	0	0	1	0	0	0	0	0	0	0	0	0	0	0	0	0	0	0
69232	Embolism and thrombosis of the anterior tibial artery	0	0	1	0	0	0	0	0	0	0	0	0	0	0	0	0	0	0
31460	Embolism and thrombosis of the axillary artery	0	0	1	0	0	0	0	0	0	0	0	0	0	0	0	0	0	0
34159	Embolism and thrombosis of the brachial artery	0	0	1	0	0	0	0	0	0	0	0	0	0	0	0	0	0	0
47665	Embolism and thrombosis of the coeliac artery	0	0	1	0	0	0	0	0	0	0	0	0	0	0	0	0	0	0
71860	Embolism and thrombosis of the dorsalis pedis artery	0	0	1	0	0	0	0	0	0	0	0	0	0	0	0	0	0	0
2065	Embolism and thrombosis of the femoral artery	0	0	1	0	0	0	0	0	0	0	0	0	0	0	0	0	0	0
27494	Embolism and thrombosis of the iliac artery unspecified	0	0	1	0	0	0	0	0	0	0	0	0	0	0	0	0	0	0
4539	Embolism and thrombosis of the popliteal artery	0	0	1	0	0	0	0	0	0	0	0	0	0	0	0	0	0	0
99532	Embolism and thrombosis of the posterior tibial artery	0	0	1	0	0	0	0	0	0	0	0	0	0	0	0	0	0	0
29372	Embolism and thrombosis of the radial artery	0	0	1	0	0	0	0	0	0	0	0	0	0	0	0	0	0	0
6900	Embolism and thrombosis of the subclavian artery	0	0	1	0	0	0	0	0	0	0	0	0	0	0	0	0	0	0
62368	Embolism and thrombosis of the ulnar artery	0	0	1	0	0	0	0	0	0	0	0	0	0	0	0	0	0	0
54865	Embolism and/or thrombosis of the common iliac artery	0	0	1	0	0	0	0	0	0	0	0	0	0	0	0	0	0	0
56919	Embolism and/or thrombosis of the external iliac artery	0	0	1	0	0	0	0	0	0	0	0	0	0	0	0	0	0	0
32634	Embolism and/or thrombosis of the internal iliac artery	0	0	1	0	0	0	0	0	0	0	0	0	0	0	0	0	0	0
15302	Peripheral arterial embolism and thrombosis NOS	0	0	1	0	0	0	0	0	0	0	0	0	0	0	0	0	0	0
109425	[X]Embolism and thrombosis of other arteries	0	0	1	0	0	0	0	0	0	0	0	0	0	0	0	0	0	0
28004	Arterial embolic and thrombotic occlusion	0	0	1	0	0	0	0	0	0	0	0	0	0	0	0	0	0	0
4289	Arterial embolism and thrombosis	0	0	1	0	0	0	0	0	0	0	0	0	0	0	0	0	0	0
3714	Arterial embolism and thrombosis NOS	0	0	1	0	0	0	0	0	0	0	0	0	0	0	0	0	0	0
8998	Arterial embolus and thrombosis	0	0	1	0	0	0	0	0	0	0	0	0	0	0	0	0	0	0
107071	ARAS - Atherosclerotic renal artery stenosis	0	0	1	0	0	0	0	0	0	0	0	0	0	0	0	0	0	0
16284	Renal artery atherosclerosis	0	0	1	0	0	0	0	0	0	0	0	0	0	0	0	0	0	0

23871	Peripheral angiopathic disease EC NOS	0	0	1	0	0	0	0	0	0	0	0	0	0	0	0	0	0	0
100579	[X]Atherosclerosis of other arteries	0	0	1	0	0	0	0	0	0	0	0	0	0	0	0	0	0	0
996	Arteriosclerosis	0	0	1	0	0	0	0	0	0	0	0	0	0	0	0	0	0	0
3995	Arteriosclerotic vascular disease NOS	0	0	1	0	0	0	0	0	0	0	0	0	0	0	0	0	0	0
5640	Atherosclerosis	0	0	1	0	0	0	0	0	0	0	0	0	0	0	0	0	0	0
36609	Atherosclerotic cardiovascular disease	0	0	1	0	0	0	0	0	0	0	0	0	0	0	0	0	0	0
56621	Cardiovascular arteriosclerosis unspecified	0	0	1	0	0	0	0	0	0	0	0	0	0	0	0	0	0	0
5168	Other specified artery atheroma	0	0	1	0	0	0	0	0	0	0	0	0	0	0	0	0	0	0
37199	Carotid artery atherosclerosis	0	0	1	0	0	0	0	0	0	0	0	0	0	0	0	0	0	0
51804	Monckeberg's medial sclerosis	0	0	1	0	0	0	0	0	0	0	0	0	0	0	0	0	0	0
4970	[D]Gangrene	0	0	1	0	0	0	0	0	0	0	0	0	0	0	0	0	0	0
37750	[D]Gangrene NOS	0	0	1	0	0	0	0	0	0	0	0	0	0	0	0	0	0	0
73961	[X]Other specified peripheral vascular diseases	0	0	1	0	0	0	0	0	0	0	0	0	0	0	0	0	0	0
23497	Buerger's disease	0	0	1	0	0	0	0	0	0	0	0	0	0	0	0	0	0	0
6853	Claudication	0	0	1	0	0	0	0	0	0	0	0	0	0	0	0	0	0	0
7975	Claudication distance	0	0	1	0	0	0	0	0	0	0	0	0	0	0	0	0	0	0
12735	Gangrene of foot	0	0	1	0	0	0	0	0	0	0	0	0	0	0	0	0	0	0
5414	Gangrene of toe	0	0	1	0	0	0	0	0	0	0	0	0	0	0	0	0	0	0
1517	Intermittent claudication	0	0	1	0	0	0	0	0	0	0	0	0	0	0	0	0	0	0
1826	Ischaemia of legs	0	0	1	0	0	0	0	0	0	0	0	0	0	0	0	0	0	0
98174	Ischaemic foot	0	0	1	0	0	0	0	0	0	0	0	0	0	0	0	0	0	0
6308	Ischaemic leg ulcer	0	0	1	0	0	0	0	0	0	0	0	0	0	0	0	0	0	0
9561	Ischaemic toe	0	0	1	0	0	0	0	0	0	0	0	0	0	0	0	0	0	0
40068	Presenile gangrene	0	0	1	0	0	0	0	0	0	0	0	0	0	0	0	0	0	0
34638	Thromboangiitis obliterans	0	0	1	0	0	0	0	0	0	0	0	0	0	0	0	0	0	0
67401	Thromboangiitis obliterans NOS	0	0	1	0	0	0	0	0	0	0	0	0	0	0	0	0	0	0

101866	Vascular claudication	0	0	1	0	0	0	0	0	0	0	0	0	0	0	0	0	0	0
9204	Peripheral gangrene	0	0	1	0	0	0	0	0	0	0	0	0	0	0	0	0	0	0
14797	Extremity artery atheroma	0	0	1	0	0	0	0	0	0	0	0	0	0	0	0	0	0	0
16260	Extremity artery atheroma NOS	0	0	1	0	0	0	0	0	0	0	0	0	0	0	0	0	0	0
18423	Arterial insufficiency	0	0	1	0	0	0	0	0	0	0	0	0	0	0	0	0	0	0
38907	Other specified peripheral vascular disease	0	0	1	0	0	0	0	0	0	0	0	0	0	0	0	0	0	0
4325	Other specified peripheral vascular disease NOS	0	0	1	0	0	0	0	0	0	0	0	0	0	0	0	0	0	0
6827	Peripheral ischaemia	0	0	1	0	0	0	0	0	0	0	0	0	0	0	0	0	0	0
5702	Peripheral ischaemic vascular disease	0	0	1	0	0	0	0	0	0	0	0	0	0	0	0	0	0	0
105317	Peripheral arterial disease	0	0	1	0	0	0	0	0	0	0	0	0	0	0	0	0	0	0
3530	Peripheral vascular disease NOS	0	0	1	0	0	0	0	0	0	0	0	0	0	0	0	0	0	0
2760	Peripheral vascular disease NOS	0	0	1	0	0	0	0	0	0	0	0	0	0	0	0	0	0	0
39949	Gangrene of finger	0	0	1	0	0	0	0	0	0	0	0	0	0	0	0	0	0	0
23672	Gangrene of hand	0	0	1	0	0	0	0	0	0	0	0	0	0	0	0	0	0	0
5943	Other peripheral vascular disease	0	0	1	0	0	0	0	0	0	0	0	0	0	0	0	0	0	0
93060	Emerg replace aneurysm thor aorta by anastom aorta to aorta	0	0	1	0	0	0	0	0	0	0	0	0	0	0	0	0	0	0
43108	Other replacement of aneurysmal segment of aorta	0	0	1	0	0	0	0	0	0	0	0	0	0	0	0	0	0	0
36651	Other replacement of aneurysmal segment of aorta NOS	0	0	1	0	0	0	0	0	0	0	0	0	0	0	0	0	0	0
55445	Other replacement of aneurysmal segment of aorta OS	0	0	1	0	0	0	0	0	0	0	0	0	0	0	0	0	0	0
1736	Aortic aneurysm repair	0	0	1	0	0	0	0	0	0	0	0	0	0	0	0	0	0	0
34153	Axillo-bifemoral bypass graft	0	0	1	0	0	0	0	0	0	0	0	0	0	0	0	0	0	0
40302	Axillo-unifemoral PTFE bypass graft	0	0	1	0	0	0	0	0	0	0	0	0	0	0	0	0	0	0
102684	Bypass aorta anastomosis axillary artery bi femoral arteries	0	0	1	0	0	0	0	0	0	0	0	0	0	0	0	0	0	0
2761	Bypass bifurc aorta by anastom aorta to femoral artery NEC	0	0	1	0	0	0	0	0	0	0	0	0	0	0	0	0	0	0
32492	Bypass common iliac artery by aorta/com iliac art anast NEC	0	0	1	0	0	0	0	0	0	0	0	0	0	0	0	0	0	0

67982	Bypass femoral artery by fem/peron a anast c prosthesis NEC	0	0	1	0	0	0	0	0	0	0	0	0	0	0	0	0	0	0	0	0
53675	Bypass femoral artery by fem/peron a anast c vein graft NEC	0	0	1	0	0	0	0	0	0	0	0	0	0	0	0	0	0	0	0	0
27580	Bypass femoral artery by fem/pop art anast c prosthesis NEC	0	0	1	0	0	0	0	0	0	0	0	0	0	0	0	0	0	0	0	0
28030	Bypass femoral artery by fem/pop art anast c vein graft NEC	0	0	1	0	0	0	0	0	0	0	0	0	0	0	0	0	0	0	0	0
39877	Bypass femoral artery by fem/tib art anast c prosthesis NEC	0	0	1	0	0	0	0	0	0	0	0	0	0	0	0	0	0	0	0	0
41823	Bypass femoral artery by fem/tib art anast c vein graft NEC	0	0	1	0	0	0	0	0	0	0	0	0	0	0	0	0	0	0	0	0
45428	Bypass femoral artery by femoral/femoral art anastomosis NEC	0	0	1	0	0	0	0	0	0	0	0	0	0	0	0	0	0	0	0	0
36443	Bypass iliac artery by femoral/femoral art anastomosis NEC	0	0	1	0	0	0	0	0	0	0	0	0	0	0	0	0	0	0	0	0
28616	Bypass iliac artery by iliac/femoral artery anastomosis NEC	0	0	1	0	0	0	0	0	0	0	0	0	0	0	0	0	0	0	0	0
100036	Bypass iliac artery by iliac/iliac artery anastomosis NEC	0	0	1	0	0	0	0	0	0	0	0	0	0	0	0	0	0	0	0	0
55554	Bypass leg artery by aorta/com femoral art anastomosis NEC	0	0	1	0	0	0	0	0	0	0	0	0	0	0	0	0	0	0	0	0
66804	Bypass leg artery by aorta/deep femoral art anastomosis NEC	0	0	1	0	0	0	0	0	0	0	0	0	0	0	0	0	0	0	0	0
68412	Bypass popliteal art by pop/peron art anast c vein graft NEC	0	0	1	0	0	0	0	0	0	0	0	0	0	0	0	0	0	0	0	0
42115	Bypass popliteal artery by pop/fem artery anastomosis NEC	0	0	1	0	0	0	0	0	0	0	0	0	0	0	0	0	0	0	0	0
64555	Bypass popliteal artery by pop/pop a anast c prosthesis NEC	0	0	1	0	0	0	0	0	0	0	0	0	0	0	0	0	0	0	0	0
24097	Bypass popliteal artery by pop/pop a anast c vein graft NEC	0	0	1	0	0	0	0	0	0	0	0	0	0	0	0	0	0	0	0	0
60465	Bypass popliteal artery by pop/tib a anast c prosthesis NEC	0	0	1	0	0	0	0	0	0	0	0	0	0	0	0	0	0	0	0	0
48700	Bypass popliteal artery by pop/tib a anast c vein graft NEC	0	0	1	0	0	0	0	0	0	0	0	0	0	0	0	0	0	0	0	0
38546	Dacron graft operations on aorta	0	0	1	0	0	0	0	0	0	0	0	0	0	0	0	0	0	0	0	0
102031	Emerg bypass abdominal aorta by anastom aorta to aorta NEC	0	0	1	0	0	0	0	0	0	0	0	0	0	0	0	0	0	0	0	0
48755	Emerg bypass bifurc aorta by anast aorta to femoral artery	0	0	1	0	0	0	0	0	0	0	0	0	0	0	0	0	0	0	0	0
68141	Emerg bypass comm iliac art by aorta/com iliac art anast NEC	0	0	1	0	0	0	0	0	0	0	0	0	0	0	0	0	0	0	0	0
99676	Emerg bypass femoral art by fem/peron art anast c prosth NEC	0	0	1	0	0	0	0	0	0	0	0	0	0	0	0	0	0	0	0	0
52342	Emerg bypass femoral art by fem/pop a anast c vein graft NEC	0	0	1	0	0	0	0	0	0	0	0	0	0	0	0	0	0	0	0	0

43651	Emerg bypass femoral art by fem/pop art anast c prosth NEC	0	0	1	0	0	0	0	0	0	0	0	0	0	0	0	0	0	0
96255	Emerg bypass femoral art by fem/tib a anast c vein graft NEC	0	0	1	0	0	0	0	0	0	0	0	0	0	0	0	0	0	0
66820	Emerg bypass femoral art by fem/tib art anast c prosth NEC	0	0	1	0	0	0	0	0	0	0	0	0	0	0	0	0	0	0
48939	Emerg bypass femoral artery by fem/fem art anastomosis NEC	0	0	1	0	0	0	0	0	0	0	0	0	0	0	0	0	0	0
44250	Emerg bypass iliac art by iliac/femoral art anastomosis NEC	0	0	1	0	0	0	0	0	0	0	0	0	0	0	0	0	0	0
72448	Emerg bypass iliac artery by femoral/femoral art anast NEC	0	0	1	0	0	0	0	0	0	0	0	0	0	0	0	0	0	0
66917	Emerg bypass leg artery by aorta/com fem art anastomosis NEC	0	0	1	0	0	0	0	0	0	0	0	0	0	0	0	0	0	0
62775	Emerg bypass pop art by pop/peron art anast c vein graft NEC	0	0	1	0	0	0	0	0	0	0	0	0	0	0	0	0	0	0
60693	Emerg bypass pop art by pop/pop art anast c vein graft NEC	0	0	1	0	0	0	0	0	0	0	0	0	0	0	0	0	0	0
66879	Emerg bypass pop art by pop/tib art anast c vein graft NEC	0	0	1	0	0	0	0	0	0	0	0	0	0	0	0	0	0	0
67818	Emerg bypass popliteal art by pop/pop art anast c prosth NEC	0	0	1	0	0	0	0	0	0	0	0	0	0	0	0	0	0	0
70922	Emerg bypass popliteal artery by pop/fem art anastomosis NEC	0	0	1	0	0	0	0	0	0	0	0	0	0	0	0	0	0	0
96654	Emerg repl aneurysm bifurc aorta by anast aorta to fem art	0	0	1	0	0	0	0	0	0	0	0	0	0	0	0	0	0	0
69922	Emerg repl aneurysm bifurc aorta by anast aorta to iliac a	0	0	1	0	0	0	0	0	0	0	0	0	0	0	0	0	0	0
54192	Emerg replace aneurysm abdom aorta by anast aorta/aorta NEC	0	0	1	0	0	0	0	0	0	0	0	0	0	0	0	0	0	0
99722	Emerg replace aneurysm asc aorta by anastom aorta to aorta	0	0	1	0	0	0	0	0	0	0	0	0	0	0	0	0	0	0
66232	Emerg replace aneurysm infrarenal aorta by anast aorta/aorta	0	0	1	0	0	0	0	0	0	0	0	0	0	0	0	0	0	0
17220	Emergency repair of aortic aneurysm	0	0	1	0	0	0	0	0	0	0	0	0	0	0	0	0	0	0
31613	Emergency replacement of aneurysmal segment of aorta	0	0	1	0	0	0	0	0	0	0	0	0	0	0	0	0	0	0
45474	Emergency replacement of aneurysmal segment of aorta NOS	0	0	1	0	0	0	0	0	0	0	0	0	0	0	0	0	0	0
45477	Emergency replacement of aneurysmal segment of aorta OS	0	0	1	0	0	0	0	0	0	0	0	0	0	0	0	0	0	0
89714	Endov ins stent graft for aortic dissection in any position	0	0	1	0	0	0	0	0	0	0	0	0	0	0	0	0	0	0
91462	Endov insertion of stent graft for thoracic aortic aneurysm	0	0	1	0	0	0	0	0	0	0	0	0	0	0	0	0	0	0
83577	Endovas ins stent graft for infrarenal abdom aortic aneurysm	0	0	1	0	0	0	0	0	0	0	0	0	0	0	0	0	0	0

94682	Endovas insert of stent graft for suprarenal aortic aneurysm	0	0	1	0	0	0	0	0	0	0	0	0	0	0	0	0	0	0
99859	Endovas insert stent for aortic aneurysm of bifurcation NEC	0	0	1	0	0	0	0	0	0	0	0	0	0	0	0	0	0	0
94069	Endovas insertion of stent graft for aortic bifurcation NEC	0	0	1	0	0	0	0	0	0	0	0	0	0	0	0	0	0	0
103427	Endovas insertion of stent graft for aorto-uniiliac aneurysm	0	0	1	0	0	0	0	0	0	0	0	0	0	0	0	0	0	0
97217	Endovascul insert stent infrarenal abdominal aortic aneurysm	0	0	1	0	0	0	0	0	0	0	0	0	0	0	0	0	0	0
99787	Endovascular ins stent for aortic dissection in any position	0	0	1	0	0	0	0	0	0	0	0	0	0	0	0	0	0	0
98175	Endovascular insertion of stent for aorto-uniiliac aneurysm	0	0	1	0	0	0	0	0	0	0	0	0	0	0	0	0	0	0
98542	Endovascular insertion of stent for thoracic aortic aneurysm	0	0	1	0	0	0	0	0	0	0	0	0	0	0	0	0	0	0
106780	Endovascular insertion stent for suprarenal aortic aneurysm	0	0	1	0	0	0	0	0	0	0	0	0	0	0	0	0	0	0
100195	Endovascular stenting for aortic aneurysm of bifurcation NEC	0	0	1	0	0	0	0	0	0	0	0	0	0	0	0	0	0	0
97109	Endovascular stenting for aorto-uniiliac aneurysm	0	0	1	0	0	0	0	0	0	0	0	0	0	0	0	0	0	0
70446	Endovascular stenting infrarenal abdominal aortic aneurysm	0	0	1	0	0	0	0	0	0	0	0	0	0	0	0	0	0	0
90549	Endovascular stenting of aortic bifurcation NEC	0	0	1	0	0	0	0	0	0	0	0	0	0	0	0	0	0	0
83527	Endovascular stenting of aortic dissection in any position	0	0	1	0	0	0	0	0	0	0	0	0	0	0	0	0	0	0
95976	Endovascular stenting of aorto-uniiliac aneurysm	0	0	1	0	0	0	0	0	0	0	0	0	0	0	0	0	0	0
97030	Endovascular stenting of suprarenal aortic aneurysm	0	0	1	0	0	0	0	0	0	0	0	0	0	0	0	0	0	0
51061	Endovascular stenting of thoracic aortic aneurysm	0	0	1	0	0	0	0	0	0	0	0	0	0	0	0	0	0	0
33430	Operation on aneurysm of aorta NEC	0	0	1	0	0	0	0	0	0	0	0	0	0	0	0	0	0	0
98565	OS translum ins stent graft for aneurysmal segment of aorta	0	0	1	0	0	0	0	0	0	0	0	0	0	0	0	0	0	0
37787	Other bypass of common femoral artery	0	0	1	0	0	0	0	0	0	0	0	0	0	0	0	0	0	0
18060	Other bypass of femoral artery	0	0	1	0	0	0	0	0	0	0	0	0	0	0	0	0	0	0
24692	Other bypass of femoral artery or popliteal artery	0	0	1	0	0	0	0	0	0	0	0	0	0	0	0	0	0	0
2066	Other bypass of femoral artery or popliteal artery NOS	0	0	1	0	0	0	0	0	0	0	0	0	0	0	0	0	0	0
42640	Other bypass of femoral artery or popliteal artery OS	0	0	1	0	0	0	0	0	0	0	0	0	0	0	0	0	0	0

61974	Other bypass of femoral or popliteal artery by anastomosis	0	0	1	0	0	0	0	0	0	0	0	0	0	0	0	0	0	0
21927	Other bypass of iliac artery	0	0	1	0	0	0	0	0	0	0	0	0	0	0	0	0	0	0
101910	Other bypass of iliac artery by anastomosis	0	0	1	0	0	0	0	0	0	0	0	0	0	0	0	0	0	0
38921	Other bypass of iliac artery NOS	0	0	1	0	0	0	0	0	0	0	0	0	0	0	0	0	0	0
12331	Other bypass of popliteal artery	0	0	1	0	0	0	0	0	0	0	0	0	0	0	0	0	0	0
40732	Other bypass of superficial femoral artery	0	0	1	0	0	0	0	0	0	0	0	0	0	0	0	0	0	0
72491	Other emerg bypass femoral or popliteal art by anastomosis	0	0	1	0	0	0	0	0	0	0	0	0	0	0	0	0	0	0
100113	Other emergency bypass of common femoral artery	0	0	1	0	0	0	0	0	0	0	0	0	0	0	0	0	0	0
63238	Other emergency bypass of deep femoral artery	0	0	1	0	0	0	0	0	0	0	0	0	0	0	0	0	0	0
11766	Other emergency bypass of femoral artery	0	0	1	0	0	0	0	0	0	0	0	0	0	0	0	0	0	0
68320	Other emergency bypass of femoral or popliteal artery NOS	0	0	1	0	0	0	0	0	0	0	0	0	0	0	0	0	0	0
65692	Other emergency bypass of femoral or popliteal artery OS	0	0	1	0	0	0	0	0	0	0	0	0	0	0	0	0	0	0
39776	Other emergency bypass of popliteal artery	0	0	1	0	0	0	0	0	0	0	0	0	0	0	0	0	0	0
31822	Other emergency bypass of segment of aorta	0	0	1	0	0	0	0	0	0	0	0	0	0	0	0	0	0	0
97606	Other emergency bypass of superficial femoral artery	0	0	1	0	0	0	0	0	0	0	0	0	0	0	0	0	0	0
52357	Other specified other bypass of iliac artery	0	0	1	0	0	0	0	0	0	0	0	0	0	0	0	0	0	0
101835	Other specified other emergency bypass of segment of aorta	0	0	1	0	0	0	0	0	0	0	0	0	0	0	0	0	0	0
44553	Replace aneurys infrarenal aorta by anast aorta to aorta NEC	0	0	1	0	0	0	0	0	0	0	0	0	0	0	0	0	0	0
54379	Replace aneurys suprarenal aorta by anast aorta to aorta NEC	0	0	1	0	0	0	0	0	0	0	0	0	0	0	0	0	0	0
19996	Replace aneurysm abdominal aorta by anast aorta to aorta NEC	0	0	1	0	0	0	0	0	0	0	0	0	0	0	0	0	0	0
64961	Replace aneurysm ascend aorta by anast of aorta/aorta NEC	0	0	1	0	0	0	0	0	0	0	0	0	0	0	0	0	0	0
56495	Replace aneurysm bifurc aorta by anast aorta to femoral art	0	0	1	0	0	0	0	0	0	0	0	0	0	0	0	0	0	0
56510	Replace aneurysm bifurc aorta by anast aorta to iliac artery	0	0	1	0	0	0	0	0	0	0	0	0	0	0	0	0	0	0
42444	Replace aneurysm thoracic aorta by anast of aorta/aorta NEC	0	0	1	0	0	0	0	0	0	0	0	0	0	0	0	0	0	0

52358	Replacement of aneurysmal bifurcation of aorta	0	0	1	0	0	0	0	0	0	0	0	0	0	0	0	0	0	0
66761	Replacement of aneurysmal bifurcation of aorta NOS	0	0	1	0	0	0	0	0	0	0	0	0	0	0	0	0	0	0
62301	Replacement of aneurysmal bifurcation of aorta OS	0	0	1	0	0	0	0	0	0	0	0	0	0	0	0	0	0	0
93627	Translum ins stent graft for aneurysmal segment of aorta NOS	0	0	1	0	0	0	0	0	0	0	0	0	0	0	0	0	0	0
94331	Translum insert stent graft for aneurysmal segment of aorta	0	0	1	0	0	0	0	0	0	0	0	0	0	0	0	0	0	0
93959	Transluminal operations on aneurysmal segment of aorta	0	0	1	0	0	0	0	0	0	0	0	0	0	0	0	0	0	0
90861	Transluminal operations on aneurysmal segment of aorta NOS	0	0	1	0	0	0	0	0	0	0	0	0	0	0	0	0	0	0
63408	Tube graft abdominal Aortic aneurysm (emergency)	0	0	1	0	0	0	0	0	0	0	0	0	0	0	0	0	0	0
26232	Tube graft of Abdominal aortic aneurysm	0	0	1	0	0	0	0	0	0	0	0	0	0	0	0	0	0	0
51166	Y graft abdominal Aortic aneurysm	0	0	1	0	0	0	0	0	0	0	0	0	0	0	0	0	0	0
92925	Y graft of abdominal Aortic aneurysm (emergency)	0	0	1	0	0	0	0	0	0	0	0	0	0	0	0	0	0	0
504	Transient cerebral ischaemia	0	0	0	1	0	0	0	0	0	0	0	0	0	0	0	0	0	0
569	Infarction - cerebral	0	0	0	1	0	0	0	0	0	0	0	0	0	0	0	0	0	0
1195	Amaurosis fugax	0	0	0	1	0	0	0	0	0	0	0	0	0	0	0	0	0	0
1298	CVA unspecified	0	0	0	1	0	0	0	0	0	0	0	0	0	0	0	0	0	0
1433	Transient ischaemic attack	0	0	0	1	0	0	0	0	0	0	0	0	0	0	0	0	0	0
1469	Stroke and cerebrovascular accident unspecified	0	0	0	1	0	0	0	0	0	0	0	0	0	0	0	0	0	0
1786	Subarachnoid haemorrhage	0	0	0	1	0	0	0	0	0	0	0	0	0	0	0	0	0	0
1895	Transient cerebral ischaemia NOS	0	0	0	1	0	0	0	0	0	0	0	0	0	0	0	0	0	0
2156	Stenosis, carotid artery	0	0	0	1	0	0	0	0	0	0	0	0	0	0	0	0	0	0
2418	Cerebrovascular disease	0	0	0	1	0	0	0	0	0	0	0	0	0	0	0	0	0	0
2652	Carotid artery stenosis	0	0	0	1	0	0	0	0	0	0	0	0	0	0	0	0	0	0
3149	Cerebral infarction NOS	0	0	0	1	0	0	0	0	0	0	0	0	0	0	0	0	0	0
3535	Intracerebral haemorrhage NOS	0	0	0	1	0	0	0	0	0	0	0	0	0	0	0	0	0	0
3585	Thrombosis lateral sinus	0	0	0	1	0	0	0	0	0	0	0	0	0	0	0	0	0	0
3979	Hypertensive encephalopathy	0	0	0	1	0	0	0	0	0	0	0	0	0	0	0	0	0	0
4152	Thrombosis, carotid artery	0	0	0	1	0	0	0	0	0	0	0	0	0	0	0	0	0	0

4240	Carotid artery occlusion	0	0	0	1	0	0	0	0	0	0	0	0	0	0	0	0	0	0
4273	Subdural haemorrhage - nontraumatic	0	0	0	1	0	0	0	0	0	0	0	0	0	0	0	0	0	0
4635	Cerebral aneurysm, nonruptured	0	0	0	1	0	0	0	0	0	0	0	0	0	0	0	0	0	0
5051	Intracerebral haemorrhage	0	0	0	1	0	0	0	0	0	0	0	0	0	0	0	0	0	0
5095	Binswanger's disease	0	0	0	1	0	0	0	0	0	0	0	0	0	0	0	0	0	0
5184	Precerebral atherosclerosis	0	0	0	1	0	0	0	0	0	0	0	0	0	0	0	0	0	0
5185	Lateral medullary syndrome	0	0	0	1	0	0	0	0	0	0	0	0	0	0	0	0	0	0
5268	Insufficiency - basilar artery	0	0	0	1	0	0	0	0	0	0	0	0	0	0	0	0	0	0
5363	CVA - cerebral artery occlusion	0	0	0	1	0	0	0	0	0	0	0	0	0	0	0	0	0	0
5365	Cerebral artery aneurysm operations	0	0	0	1	0	0	0	0	0	0	0	0	0	0	0	0	0	0
5602	Cerebellar infarction	0	0	0	1	0	0	0	0	0	0	0	0	0	0	0	0	0	0
5871	H/O: stroke	0	0	0	1	0	0	0	0	0	0	0	0	0	0	0	0	0	0
6116	CVA - Cerebrovascular accident unspecified	0	0	0	1	0	0	0	0	0	0	0	0	0	0	0	0	0	0
6155	Stroke due to cerebral arterial occlusion	0	0	0	1	0	0	0	0	0	0	0	0	0	0	0	0	0	0
6228	Sequelae of stroke	0	0	0	1	0	0	0	0	0	0	0	0	0	0	0	0	0	0
6253	Stroke unspecified	0	0	0	1	0	0	0	0	0	0	0	0	0	0	0	0	0	0
6305	H/O: CVA	0	0	0	1	0	0	0	0	0	0	0	0	0	0	0	0	0	0
6489	Transient global amnesia	0	0	0	1	0	0	0	0	0	0	0	0	0	0	0	0	0	0
6960	CVA - cerebrovascular accid due to intracerebral haemorrhage	0	0	0	1	0	0	0	0	0	0	0	0	0	0	0	0	0	0
7138	[V]Personal history of cerebrovascular accident (CVA)	0	0	0	1	0	0	0	0	0	0	0	0	0	0	0	0	0	0
7780	Left sided CVA	0	0	0	1	0	0	0	0	0	0	0	0	0	0	0	0	0	0
7912	Pontine haemorrhage	0	0	0	1	0	0	0	0	0	0	0	0	0	0	0	0	0	0
8443	Brain stem stroke syndrome	0	0	0	1	0	0	0	0	0	0	0	0	0	0	0	0	0	0
8837	Cerebral arterial occlusion	0	0	0	1	0	0	0	0	0	0	0	0	0	0	0	0	0	0
9696	Subarachnoid haemorrhage from posterior communicating artery	0	0	0	1	0	0	0	0	0	0	0	0	0	0	0	0	0	0
9943	Cereb autosom dominant arteriop subcort infarcts leukoenceph	0	0	0	1	0	0	0	0	0	0	0	0	0	0	0	0	0	0
9985	Left sided cerebral infarction	0	0	0	1	0	0	0	0	0	0	0	0	0	0	0	0	0	0
10062	Cerebrovascular disease NOS	0	0	0	1	0	0	0	0	0	0	0	0	0	0	0	0	0	0
10189	Cerebral amyloid angiopathy	0	0	0	1	0	0	0	0	0	0	0	0	0	0	0	0	0	0

10504	Right sided cerebral infarction	0	0	0	1	0	0	0	0	0	0	0	0	0	0	0	0	0	0
10625	Clipping of aneurysm of cerebral artery	0	0	0	1	0	0	0	0	0	0	0	0	0	0	0	0	0	0
11171	Cerebral atherosclerosis	0	0	0	1	0	0	0	0	0	0	0	0	0	0	0	0	0	0
12555	Generalised ischaemic cerebrovascular disease NOS	0	0	0	1	0	0	0	0	0	0	0	0	0	0	0	0	0	0
12833	Right sided CVA	0	0	0	1	0	0	0	0	0	0	0	0	0	0	0	0	0	0
13564	Cerebellar haemorrhage	0	0	0	1	0	0	0	0	0	0	0	0	0	0	0	0	0	0
13567	H/O: TIA	0	0	0	1	0	0	0	0	0	0	0	0	0	0	0	0	0	0
13577	Other cerebrovascular disease	0	0	0	1	0	0	0	0	0	0	0	0	0	0	0	0	0	0
13707	Stroke / transient ischaemic attack referral	0	0	0	1	0	0	0	0	0	0	0	0	0	0	0	0	0	0
15019	Cerebral embolism	0	0	0	1	0	0	0	0	0	0	0	0	0	0	0	0	0	0
15252	Brainstem infarction NOS	0	0	0	1	0	0	0	0	0	0	0	0	0	0	0	0	0	0
15788	Transient cerebral ischaemia NOS	0	0	0	1	0	0	0	0	0	0	0	0	0	0	0	0	0	0
16507	Intermittent cerebral ischaemia	0	0	0	1	0	0	0	0	0	0	0	0	0	0	0	0	0	0
16517	Cerebral thrombosis	0	0	0	1	0	0	0	0	0	0	0	0	0	0	0	0	0	0
16554	H/O sub-arachnoid haemorrhage	0	0	0	1	0	0	0	0	0	0	0	0	0	0	0	0	0	0
17322	Cerebellar stroke syndrome	0	0	0	1	0	0	0	0	0	0	0	0	0	0	0	0	0	0
17326	Subarachnoid haemorrh from intracranial artery	0	0	0	1	0	0	0	0	0	0	0	0	0	0	0	0	0	0
17734	Subdural haematoma - nontraumatic	0	0	0	1	0	0	0	0	0	0	0	0	0	0	0	0	0	0
18604	Stroke due to intracerebral haemorrhage	0	0	0	1	0	0	0	0	0	0	0	0	0	0	0	0	0	0
18689	Middle cerebral artery syndrome	0	0	0	1	0	0	0	0	0	0	0	0	0	0	0	0	0	0
18996	Transient global amnesia	0	0	0	1	0	0	0	0	0	0	0	0	0	0	0	0	0	0
19004	TGA - Transient global amnesia	0	0	0	1	0	0	0	0	0	0	0	0	0	0	0	0	0	0
19201	Right sided intracerebral haemorrhage, unspecified	0	0	0	1	0	0	0	0	0	0	0	0	0	0	0	0	0	0
19260	Posterior cerebral artery syndrome	0	0	0	1	0	0	0	0	0	0	0	0	0	0	0	0	0	0

19280	Anterior cerebral artery syndrome	0	0	0	1	0	0	0	0	0	0	0	0	0	0	0	0	0	0
19348	[V]Personal history of stroke	0	0	0	1	0	0	0	0	0	0	0	0	0	0	0	0	0	0
19354	Other transient cerebral ischaemia	0	0	0	1	0	0	0	0	0	0	0	0	0	0	0	0	0	0
19412	Subarachnoid haemorrhage from middle cerebral artery	0	0	0	1	0	0	0	0	0	0	0	0	0	0	0	0	0	0
20161	Thrombosis of superior longitudinal sinus	0	0	0	1	0	0	0	0	0	0	0	0	0	0	0	0	0	0
20284	Intracranial haemorrhage NOS	0	0	0	1	0	0	0	0	0	0	0	0	0	0	0	0	0	0
21118	Vertebro-basilar artery syndrome	0	0	0	1	0	0	0	0	0	0	0	0	0	0	0	0	0	0
22006	Thrombosis cavernous sinus	0	0	0	1	0	0	0	0	0	0	0	0	0	0	0	0	0	0
22018	Dissection of cerebral arteries, nonruptured	0	0	0	1	0	0	0	0	0	0	0	0	0	0	0	0	0	0
22400	Cerebral arteritis	0	0	0	1	0	0	0	0	0	0	0	0	0	0	0	0	0	0
23361	Late effects of cerebrovascular disease	0	0	0	1	0	0	0	0	0	0	0	0	0	0	0	0	0	0
23580	Subarachnoid haemorrhage NOS	0	0	0	1	0	0	0	0	0	0	0	0	0	0	0	0	0	0
23671	Cerebral infarct due to thrombosis of precerebral arteries	0	0	0	1	0	0	0	0	0	0	0	0	0	0	0	0	0	0
23942	Basilar artery syndrome	0	0	0	1	0	0	0	0	0	0	0	0	0	0	0	0	0	0
24385	Chronic cerebral ischaemia	0	0	0	1	0	0	0	0	0	0	0	0	0	0	0	0	0	0
24424	Congenital cerebral arteriovenous aneurysm	0	0	0	1	0	0	0	0	0	0	0	0	0	0	0	0	0	0
24446	Cerebral infarction due to embolism of precerebral arteries	0	0	0	1	0	0	0	0	0	0	0	0	0	0	0	0	0	0
25615	Brainstem infarction	0	0	0	1	0	0	0	0	0	0	0	0	0	0	0	0	0	0
26424	Infarction of basal ganglia	0	0	0	1	0	0	0	0	0	0	0	0	0	0	0	0	0	0
27975	Cerebral infarction due to embolism of cerebral arteries	0	0	0	1	0	0	0	0	0	0	0	0	0	0	0	0	0	0
28278	Transient global amnesia	0	0	0	1	0	0	0	0	0	0	0	0	0	0	0	0	0	0
28309	Thrombosis transverse sinus	0	0	0	1	0	0	0	0	0	0	0	0	0	0	0	0	0	0
28314	Left sided intracerebral haemorrhage, unspecified	0	0	0	1	0	0	0	0	0	0	0	0	0	0	0	0	0	0
29939	Ruptured berry aneurysm	0	0	0	1	0	0	0	0	0	0	0	0	0	0	0	0	0	0

30045	External capsule haemorrhage	0	0	0	1	0	0	0	0	0	0	0	0	0	0	0	0	0	0
30202	Intracerebral haemorrhage	0	0	0	1	0	0	0	0	0	0	0	0	0	0	0	0	0	0
30502	CVA prevention	0	0	0	1	0	0	0	0	0	0	0	0	0	0	0	0	0	0
31060	Intracerebral haemorrhage in hemisphere, unspecified	0	0	0	1	0	0	0	0	0	0	0	0	0	0	0	0	0	0
31390	Thrombosis of central nervous system venous sinuses	0	0	0	1	0	0	0	0	0	0	0	0	0	0	0	0	0	0
31595	Cortical haemorrhage	0	0	0	1	0	0	0	0	0	0	0	0	0	0	0	0	0	0
31704	Occlusion/stenosis cerebral arts not result cerebral infarct	0	0	0	1	0	0	0	0	0	0	0	0	0	0	0	0	0	0
31805	Other and unspecified intracranial haemorrhage	0	0	0	1	0	0	0	0	0	0	0	0	0	0	0	0	0	0
31941	Rupture of syphilitic cerebral aneurysm	0	0	0	1	0	0	0	0	0	0	0	0	0	0	0	0	0	0
32310	Moyamoya disease	0	0	0	1	0	0	0	0	0	0	0	0	0	0	0	0	0	0
32447	Basilar artery occlusion	0	0	0	1	0	0	0	0	0	0	0	0	0	0	0	0	0	0
33377	Vertebral artery syndrome	0	0	0	1	0	0	0	0	0	0	0	0	0	0	0	0	0	0
33499	Pure motor lacunar syndrome	0	0	0	1	0	0	0	0	0	0	0	0	0	0	0	0	0	0
33543	Cerebrl infarctn due/unspcf occlusn or sten/cerebrl artrs	0	0	0	1	0	0	0	0	0	0	0	0	0	0	0	0	0	0
34117	Other cerebrovascular disease OS	0	0	0	1	0	0	0	0	0	0	0	0	0	0	0	0	0	0
34135	H/O: CVA/stroke	0	0	0	1	0	0	0	0	0	0	0	0	0	0	0	0	0	0
34758	Cerebral embolus	0	0	0	1	0	0	0	0	0	0	0	0	0	0	0	0	0	0
36178	Extradural haemorrhage - nontraumatic	0	0	0	1	0	0	0	0	0	0	0	0	0	0	0	0	0	0
36717	Cerebral infarction due to thrombosis of cerebral arteries	0	0	0	1	0	0	0	0	0	0	0	0	0	0	0	0	0	0
37493	Other cerebrovascular disease NOS	0	0	0	1	0	0	0	0	0	0	0	0	0	0	0	0	0	0
37947	Nonpyogenic venous sinus thrombosis	0	0	0	1	0	0	0	0	0	0	0	0	0	0	0	0	0	0
39344	Cereb infarct due cerebral venous thrombosis, nonpyogenic	0	0	0	1	0	0	0	0	0	0	0	0	0	0	0	0	0	0
39403	Sequelae of cerebral infarction	0	0	0	1	0	0	0	0	0	0	0	0	0	0	0	0	0	0
40053	Generalised ischaemic cerebrovascular disease NOS	0	0	0	1	0	0	0	0	0	0	0	0	0	0	0	0	0	0

40338	Internal capsule haemorrhage	0	0	0	1	0	0	0	0	0	0	0	0	0	0	0	0	0	0
40758	Cereb infarct due unsp occlus/stenos precerebr arteries	0	0	0	1	0	0	0	0	0	0	0	0	0	0	0	0	0	0
40847	Vertebral artery occlusion	0	0	0	1	0	0	0	0	0	0	0	0	0	0	0	0	0	0
41180	Thrombophlebitis of central nervous system venous sinuses	0	0	0	1	0	0	0	0	0	0	0	0	0	0	0	0	0	0
41577	H/O: cerebrovascular disease	0	0	0	1	0	0	0	0	0	0	0	0	0	0	0	0	0	0
41910	Subarachnoid haemorrhage from basilar artery	0	0	0	1	0	0	0	0	0	0	0	0	0	0	0	0	0	0
42331	Subarachnoid haemorrhage from anterior communicating artery	0	0	0	1	0	0	0	0	0	0	0	0	0	0	0	0	0	0
43451	Sequelae of other nontraumatic intracranial haemorrhage	0	0	0	1	0	0	0	0	0	0	0	0	0	0	0	0	0	0
44740	Sequelae of subarachnoid haemorrhage	0	0	0	1	0	0	0	0	0	0	0	0	0	0	0	0	0	0
44765	Carotid artery syndrome hemispheric	0	0	0	1	0	0	0	0	0	0	0	0	0	0	0	0	0	0
45781	Precerebral arterial occlusion	0	0	0	1	0	0	0	0	0	0	0	0	0	0	0	0	0	0
46316	Basal nucleus haemorrhage	0	0	0	1	0	0	0	0	0	0	0	0	0	0	0	0	0	0
47607	CVA - cerebrovascular accident in the puerperium	0	0	0	1	0	0	0	0	0	0	0	0	0	0	0	0	0	0
47642	Wallenberg syndrome	0	0	0	1	0	0	0	0	0	0	0	0	0	0	0	0	0	0
48149	Sequelae of intracerebral haemorrhage	0	0	0	1	0	0	0	0	0	0	0	0	0	0	0	0	0	0
49715	Phlebitis and thrombophlebitis of intracranial sinuses	0	0	0	1	0	0	0	0	0	0	0	0	0	0	0	0	0	0
50594	Multiple and bilateral precerebral artery syndromes	0	0	0	1	0	0	0	0	0	0	0	0	0	0	0	0	0	0
50725	Listerial cerebral arteritis	0	0	0	1	0	0	0	0	0	0	0	0	0	0	0	0	0	0
51138	Sequelae/other + unspecified cerebrovascular diseases	0	0	0	1	0	0	0	0	0	0	0	0	0	0	0	0	0	0
51311	Other specified cerebrovascular disease	0	0	0	1	0	0	0	0	0	0	0	0	0	0	0	0	0	0
51326	Other precerebral artery occlusion	0	0	0	1	0	0	0	0	0	0	0	0	0	0	0	0	0	0
51759	Occlusion and stenosis of middle cerebral artery	0	0	0	1	0	0	0	0	0	0	0	0	0	0	0	0	0	0
51767	Pure sensory lacunar syndrome	0	0	0	1	0	0	0	0	0	0	0	0	0	0	0	0	0	0
53745	[X]Other cerebral infarction	0	0	0	1	0	0	0	0	0	0	0	0	0	0	0	0	0	0

53810	[X]Other intracerebral haemorrhage	0	0	0	1	0	0	0	0	0	0	0	0	0	0	0	0	0	0
54744	Cerebral degeneration due to cerebrovascular disease	0	0	0	1	0	0	0	0	0	0	0	0	0	0	0	0	0	0
55602	Occlusion and stenosis of cerebellar arteries	0	0	0	1	0	0	0	0	0	0	0	0	0	0	0	0	0	0
55883	Thrombophlebitis of cavernous sinus	0	0	0	1	0	0	0	0	0	0	0	0	0	0	0	0	0	0
55885	Embolism cavernous sinus	0	0	0	1	0	0	0	0	0	0	0	0	0	0	0	0	0	0
56007	Subarachnoid haemorrhage from carotid siphon and bifurcation	0	0	0	1	0	0	0	0	0	0	0	0	0	0	0	0	0	0
57315	Intracerebral haemorrhage	0	0	0	1	0	0	0	0	0	0	0	0	0	0	0	0	0	0
57495	Infarction - precerebral	0	0	0	1	0	0	0	0	0	0	0	0	0	0	0	0	0	0
57527	Occlusion and stenosis of anterior cerebral artery	0	0	0	1	0	0	0	0	0	0	0	0	0	0	0	0	0	0
60692	Subarachnoid haemorrhage from vertebral artery	0	0	0	1	0	0	0	0	0	0	0	0	0	0	0	0	0	0
61366	Thrombosis of central nervous system venous sinus NOS	0	0	0	1	0	0	0	0	0	0	0	0	0	0	0	0	0	0
63746	[X]Other transnt cerebral ischaemic attacks+related syndroms	0	0	0	1	0	0	0	0	0	0	0	0	0	0	0	0	0	0
63830	Stenosis of precerebral arteries	0	0	0	1	0	0	0	0	0	0	0	0	0	0	0	0	0	0
65745	[X]Other subarachnoid haemorrhage	0	0	0	1	0	0	0	0	0	0	0	0	0	0	0	0	0	0
65770	Occlusion and stenosis of posterior cerebral artery	0	0	0	1	0	0	0	0	0	0	0	0	0	0	0	0	0	0
65855	Phlebitis or thrombophlebitis of CNS venous sinus NOS	0	0	0	1	0	0	0	0	0	0	0	0	0	0	0	0	0	0
66873	H/O: Stroke in last year	0	0	0	1	0	0	0	0	0	0	0	0	0	0	0	0	0	0
68194	Binswanger's encephalopathy	0	0	0	1	0	0	0	0	0	0	0	0	0	0	0	0	0	0
70455	Thrombophlebitis of superior longitudinal venous sinus	0	0	0	1	0	0	0	0	0	0	0	0	0	0	0	0	0	0
70536	Acute cerebrovascular insufficiency NOS	0	0	0	1	0	0	0	0	0	0	0	0	0	0	0	0	0	0
71274	Occlusion+stenosis of multiple and bilat cerebral arteries	0	0	0	1	0	0	0	0	0	0	0	0	0	0	0	0	0	0
71585	Precerebral artery occlusion NOS	0	0	0	1	0	0	0	0	0	0	0	0	0	0	0	0	0	0
73901	[X]Cerebrovascular diseases	0	0	0	1	0	0	0	0	0	0	0	0	0	0	0	0	0	0
84404	Embolism transverse sinus	0	0	0	1	0	0	0	0	0	0	0	0	0	0	0	0	0	0

90572	[X]Occlusion and stenosis of other precerebral arteries	0	0	0	1	0	0	0	0	0	0	0	0	0	0	0	0	0	0
91627	[X]Cerebrl infarctn due/unspcf occlusn or sten/cerebrl artrs	0	0	0	1	0	0	0	0	0	0	0	0	0	0	0	0	0	0
92036	[X]Occlusion and stenosis of other cerebral arteries	0	0	0	1	0	0	0	0	0	0	0	0	0	0	0	0	0	0
93459	[X]Other lacunar syndromes	0	0	0	1	0	0	0	0	0	0	0	0	0	0	0	0	0	0
94482	[X]Cereb infarct due unsp occlus/stenos precerebr arteries	0	0	0	1	0	0	0	0	0	0	0	0	0	0	0	0	0	0
95347	[X]Other vascular syndroms/brain in cerebrovasculr diseases	0	0	0	1	0	0	0	0	0	0	0	0	0	0	0	0	0	0
96630	[X]Intracerebral haemorrhage in hemisphere, unspecified	0	0	0	1	0	0	0	0	0	0	0	0	0	0	0	0	0	0
98188	Small vessel cerebrovascular disease	0	0	0	1	0	0	0	0	0	0	0	0	0	0	0	0	0	0
98642	Multiple and bilateral precerebral arterial occlusion	0	0	0	1	0	0	0	0	0	0	0	0	0	0	0	0	0	0
99367	[X]Other cerebrovascular disorders in diseases CE	0	0	0	1	0	0	0	0	0	0	0	0	0	0	0	0	0	0
100639	Central post-stroke pain	0	0	0	1	0	0	0	0	0	0	0	0	0	0	0	0	0	0
101251	[V]Personal history of transient ischaemic attack	0	0	0	1	0	0	0	0	0	0	0	0	0	0	0	0	0	0
101733	Cerebral vein thrombosis	0	0	0	1	0	0	0	0	0	0	0	0	0	0	0	0	0	0
101824	Adrenocortical haemorrhage	0	0	0	1	0	0	0	0	0	0	0	0	0	0	0	0	0	0
105202	H/O amaurosis fugax	0	0	0	1	0	0	0	0	0	0	0	0	0	0	0	0	0	0
107440	Lobar cerebral haemorrhage	0	0	0	1	0	0	0	0	0	0	0	0	0	0	0	0	0	0
107725	Reversible cerebral vasoconstriction syndrome	0	0	0	1	0	0	0	0	0	0	0	0	0	0	0	0	0	0
108630	[X]Subarachnoid haemorrh from intracranial artery, unspecif	0	0	0	1	0	0	0	0	0	0	0	0	0	0	0	0	0	0
108668	[X]Subarachnoid haemorrhage from other intracranial arteries	0	0	0	1	0	0	0	0	0	0	0	0	0	0	0	0	0	0
36568	Embolism of central nervous system venous sinus	0	0	0	1	0	0	0	0	0	0	0	0	0	0	0	0	0	0
100015	Transient ischaemic attack clinical management plan	0	0	0	1	0	0	0	0	0	0	0	0	0	0	0	0	0	0
18686	Stroke/CVA annual review	0	0	0	1	0	0	0	0	0	0	0	0	0	0	0	0	0	0
55351	Delivery of rehabilitation for stroke	0	0	0	1	0	0	0	0	0	0	0	0	0	0	0	0	0	0
104505	Stroke initial post discharge review	0	0	0	1	0	0	0	0	0	0	0	0	0	0	0	0	0	0

105100	Stroke 6 month review	0	0	0	1	0	0	0	0	0	0	0	0	0	0	0	0	0	0
107195	Stroke self-management plan agreed	0	0	0	1	0	0	0	0	0	0	0	0	0	0	0	0	0	0
107886	Stroke annual review	0	0	0	1	0	0	0	0	0	0	0	0	0	0	0	0	0	0
109743	Stroke self-management plan review	0	0	0	1	0	0	0	0	0	0	0	0	0	0	0	0	0	0
1350	Senile/presenile dementia	0	0	0	0	1	0	0	0	0	0	0	0	0	0	0	0	0	0
1916	Senile dementia	0	0	0	0	1	0	0	0	0	0	0	0	0	0	0	0	0	0
1917	Alzheimer's disease	0	0	0	0	1	0	0	0	0	0	0	0	0	0	0	0	0	0
2882	Senile or presenile psychoses NOS	0	0	0	0	1	0	0	0	0	0	0	0	0	0	0	0	0	0
3325	[D] Senile confusion	0	0	0	0	1	0	0	0	0	0	0	0	0	0	0	0	0	0
4357	[X] Senile dementia NOS	0	0	0	0	1	0	0	0	0	0	0	0	0	0	0	0	0	0
4693	[X] Unspecified dementia	0	0	0	0	1	0	0	0	0	0	0	0	0	0	0	0	0	0
5931	H/O: dementia	0	0	0	0	1	0	0	0	0	0	0	0	0	0	0	0	0	0
6578	[X]Vascular dementia	0	0	0	0	1	0	0	0	0	0	0	0	0	0	0	0	0	0
7323	Uncomplicated senile dementia	0	0	0	0	1	0	0	0	0	0	0	0	0	0	0	0	0	0
7664	[X]Dementia in Alzheimer's disease	0	0	0	0	1	0	0	0	0	0	0	0	0	0	0	0	0	0
8195	[X]Alzheimer's dementia unspec	0	0	0	0	1	0	0	0	0	0	0	0	0	0	0	0	0	0
8634	Multi infarct dementia	0	0	0	0	1	0	0	0	0	0	0	0	0	0	0	0	0	0
8934	[X]Subcortical vascular dementia	0	0	0	0	1	0	0	0	0	0	0	0	0	0	0	0	0	0
9509	[X]Dementia in Parkinson's disease	0	0	0	0	1	0	0	0	0	0	0	0	0	0	0	0	0	0
9565	[X]Arteriosclerotic dementia	0	0	0	0	1	0	0	0	0	0	0	0	0	0	0	0	0	0
11175	[X]Multi-infarct dementia	0	0	0	0	1	0	0	0	0	0	0	0	0	0	0	0	0	0
11379	[X]Senile dementia,Alzheimer's type	0	0	0	0	1	0	0	0	0	0	0	0	0	0	0	0	0	0
12621	[X]Dementia in other diseases classified elsewhere	0	0	0	0	1	0	0	0	0	0	0	0	0	0	0	0	0	0
15165	Presenile dementia	0	0	0	0	1	0	0	0	0	0	0	0	0	0	0	0	0	0
15249	Other senile and presenile organic psychoses	0	0	0	0	1	0	0	0	0	0	0	0	0	0	0	0	0	0
16797	Alzheimer's disease with early onset	0	0	0	0	1	0	0	0	0	0	0	0	0	0	0	0	0	0
18386	Senile dementia with paranoia	0	0	0	0	1	0	0	0	0	0	0	0	0	0	0	0	0	0

19393	[X]Vascular dementia, unspecified	0	0	0	0	1	0	0	0	0	0	0	0	0	0	0	0	0	0
19477	Arteriosclerotic dementia	0	0	0	0	1	0	0	0	0	0	0	0	0	0	0	0	0	0
21887	Senile dementia with depression	0	0	0	0	1	0	0	0	0	0	0	0	0	0	0	0	0	0
25386	Dementia in conditions EC	0	0	0	0	1	0	0	0	0	0	0	0	0	0	0	0	0	0
25704	[X]Presenile dementia	0	0	0	0	1	0	0	0	0	0	0	0	0	0	0	0	0	0
26141	Alzheimer's disease assessment scale	0	0	0	0	1	0	0	0	0	0	0	0	0	0	0	0	0	0
26270	[X]Lewy body dementia	0	0	0	0	1	0	0	0	0	0	0	0	0	0	0	0	0	0
26323	[X]Alcoholic dementia NOS	0	0	0	0	1	0	0	0	0	0	0	0	0	0	0	0	0	0
27342	Alcoholic dementia NOS	0	0	0	0	1	0	0	0	0	0	0	0	0	0	0	0	0	0
27677	Presenile dementia with depression	0	0	0	0	1	0	0	0	0	0	0	0	0	0	0	0	0	0
27759	[X] Senile dementia, depressed or paranoid type	0	0	0	0	1	0	0	0	0	0	0	0	0	0	0	0	0	0
27935	[X] Senile psychosis NOS	0	0	0	0	1	0	0	0	0	0	0	0	0	0	0	0	0	0
28402	[X]Dementia in Pick's disease	0	0	0	0	1	0	0	0	0	0	0	0	0	0	0	0	0	0
29386	[X]Dementia in Alzheimer's disease, unspecified	0	0	0	0	1	0	0	0	0	0	0	0	0	0	0	0	0	0
30032	Presenile dementia with paranoia	0	0	0	0	1	0	0	0	0	0	0	0	0	0	0	0	0	0
30706	[X]Dementia in Alzheimer's dis, atypical or mixed type	0	0	0	0	1	0	0	0	0	0	0	0	0	0	0	0	0	0
31016	[X]Mixed cortical and subcortical vascular dementia	0	0	0	0	1	0	0	0	0	0	0	0	0	0	0	0	0	0
32057	Alzheimer's disease with late onset	0	0	0	0	1	0	0	0	0	0	0	0	0	0	0	0	0	0
33707	Senile and presenile organic psychotic conditions	0	0	0	0	1	0	0	0	0	0	0	0	0	0	0	0	0	0
34944	[X] Primary degenerative dementia NOS	0	0	0	0	1	0	0	0	0	0	0	0	0	0	0	0	0	0
37014	[X]Dementia in Huntington's disease	0	0	0	0	1	0	0	0	0	0	0	0	0	0	0	0	0	0
37015	Senile dementia with delirium	0	0	0	0	1	0	0	0	0	0	0	0	0	0	0	0	0	0
38438	Presenile dementia NOS	0	0	0	0	1	0	0	0	0	0	0	0	0	0	0	0	0	0
38678	[X]Dementia in Alzheimer's disease with late onset	0	0	0	0	1	0	0	0	0	0	0	0	0	0	0	0	0	0

41089	Senile dementia with depressive or paranoid features NOS	0	0	0	0	1	0	0	0	0	0	0	0	0	0	0	0	0	0
42279	Arteriosclerotic dementia NOS	0	0	0	0	1	0	0	0	0	0	0	0	0	0	0	0	0	0
42602	Uncomplicated presenile dementia	0	0	0	0	1	0	0	0	0	0	0	0	0	0	0	0	0	0
43089	Uncomplicated arteriosclerotic dementia	0	0	0	0	1	0	0	0	0	0	0	0	0	0	0	0	0	0
43292	Arteriosclerotic dementia with depression	0	0	0	0	1	0	0	0	0	0	0	0	0	0	0	0	0	0
43346	[X]Primary degen dementia of Alzheimer's type	0	0	0	0	1	0	0	0	0	0	0	0	0	0	0	0	0	0
44674	Senile dementia with depressive or paranoid features	0	0	0	0	1	0	0	0	0	0	0	0	0	0	0	0	0	0
46488	[X]Vascular dementia of acute onset	0	0	0	0	1	0	0	0	0	0	0	0	0	0	0	0	0	0
46762	[X]Alzheimer's disease type 1	0	0	0	0	1	0	0	0	0	0	0	0	0	0	0	0	0	0
47619	[X] Presenile psychosis NOS	0	0	0	0	1	0	0	0	0	0	0	0	0	0	0	0	0	0
48501	[X] Presenile dementia NOS	0	0	0	0	1	0	0	0	0	0	0	0	0	0	0	0	0	0
49263	[X]Dementia in Alzheimer's disease with early onset	0	0	0	0	1	0	0	0	0	0	0	0	0	0	0	0	0	0
49513	Presenile dementia with delirium	0	0	0	0	1	0	0	0	0	0	0	0	0	0	0	0	0	0
53446	[X]Delirium superimposed on dementia	0	0	0	0	1	0	0	0	0	0	0	0	0	0	0	0	0	0
54106	[X]Dementia in Creutzfeldt-Jakob disease	0	0	0	0	1	0	0	0	0	0	0	0	0	0	0	0	0	0
54505	Other alcoholic dementia	0	0	0	0	1	0	0	0	0	0	0	0	0	0	0	0	0	0
55222	Language disorder of dementia	0	0	0	0	1	0	0	0	0	0	0	0	0	0	0	0	0	0
55313	[X]Other vascular dementia	0	0	0	0	1	0	0	0	0	0	0	0	0	0	0	0	0	0
55467	Arteriosclerotic dementia with paranoia	0	0	0	0	1	0	0	0	0	0	0	0	0	0	0	0	0	0
55838	[X]Predominantly cortical dementia	0	0	0	0	1	0	0	0	0	0	0	0	0	0	0	0	0	0
56912	Arteriosclerotic dementia with delirium	0	0	0	0	1	0	0	0	0	0	0	0	0	0	0	0	0	0
59122	[X]Other Alzheimer's disease	0	0	0	0	1	0	0	0	0	0	0	0	0	0	0	0	0	0
60059	[X]Primary degen dementia	0	0	0	0	1	0	0	0	0	0	0	0	0	0	0	0	0	0
61528	[X]Alzheimer's disease type 2	0	0	0	0	1	0	0	0	0	0	0	0	0	0	0	0	0	0

62132	Drug-induced dementia	0	0	0	0	1	0	0	0	0	0	0	0	0	0	0	0	0	0
64267	[X]Dementia in other specified diseases classif elsewhere	0	0	0	0	1	0	0	0	0	0	0	0	0	0	0	0	0	0
109047	Antipsychotic drug therapy for dementia	0	0	0	0	1	0	0	0	0	0	0	0	0	0	0	0	0	0
12710	Dementia annual review	0	0	0	0	1	0	0	0	0	0	0	0	0	0	0	0	0	0
106311	Dementia care plan	0	0	0	0	1	0	0	0	0	0	0	0	0	0	0	0	0	0
11440	Pulmonary aspergillus disease	0	0	0	0	0	1	0	0	0	0	0	0	0	0	0	0	0	0
22835	Bronchiolitis obliterans organising pneumonia	0	0	0	0	0	1	0	0	0	0	0	0	0	0	0	0	0	0
4084	Airways obstructn irreversible	0	0	0	0	0	1	0	0	0	0	0	0	0	0	0	0	0	0
45427	Pituitary snuff-takers' disease	0	0	0	0	0	1	0	0	0	0	0	0	0	0	0	0	0	0
25013	Pneumoconioses	0	0	0	0	0	1	0	0	0	0	0	0	0	0	0	0	0	0
32727	Hyperreactive airways disease	0	0	0	0	0	1	0	0	0	0	0	0	0	0	0	0	0	0
50374	Chronic pulmonary radiation disease NOS	0	0	0	0	0	1	0	0	0	0	0	0	0	0	0	0	0	0
21257	Occupational lung disease	0	0	0	0	0	1	0	0	0	0	0	0	0	0	0	0	0	0
73414	Pneumopathy due to inhalation of other dust NOS	0	0	0	0	0	1	0	0	0	0	0	0	0	0	0	0	0	0
43285	Progressive massive fibrosis	0	0	0	0	0	1	0	0	0	0	0	0	0	0	0	0	0	0
69914	Chronic pulmonary radiation disease	0	0	0	0	0	1	0	0	0	0	0	0	0	0	0	0	0	0
60313	Pneumopathy due to inhalation of other dust	0	0	0	0	0	1	0	0	0	0	0	0	0	0	0	0	0	0
53851	[X]Airway disease due to other specific organic dusts	0	0	0	0	0	1	0	0	0	0	0	0	0	0	0	0	0	0
7318	Chronic laryngotracheitis	0	0	0	0	0	1	0	0	0	0	0	0	0	0	0	0	0	0
105939	[X]Pneumoconiosis due to other dust containing silica	0	0	0	0	0	1	0	0	0	0	0	0	0	0	0	0	0	0
94486	Chronic type 2 respiratory failure	0	0	0	0	0	1	0	0	0	0	0	0	0	0	0	0	0	0
106515	[X]Hypersensitivity pneumonitis due to other organic dusts	0	0	0	0	0	1	0	0	0	0	0	0	0	0	0	0	0	0
24814	Chronic respiratory failure	0	0	0	0	0	1	0	0	0	0	0	0	0	0	0	0	0	0
103753	Idiopathic pulmonary fibrosis	0	0	0	0	0	1	0	0	0	0	0	0	0	0	0	0	0	0

103472	Pulmonary fibrosis	0	0	0	0	0	1	0	0	0	0	0	0	0	0	0	0	0	0
109815	Interstitial lung disease due to collagen vascular disease	0	0	0	0	0	1	0	0	0	0	0	0	0	0	0	0	0	0
104915	Interstitial lung disease due to connective tissue disease	0	0	0	0	0	1	0	0	0	0	0	0	0	0	0	0	0	0
100742	Allergic bronchopulmonary aspergillosis	0	0	0	0	0	1	0	0	0	0	0	0	0	0	0	0	0	0
103559	Usual interstitial pneumonitis	0	0	0	0	0	1	0	0	0	0	0	0	0	0	0	0	0	0
103785	Respiratory bronchiolitis associated interstitial lung dis	0	0	0	0	0	1	0	0	0	0	0	0	0	0	0	0	0	0
94946	Chronic type 1 respiratory failure	0	0	0	0	0	1	0	0	0	0	0	0	0	0	0	0	0	0
53205	Acute drug-induced interstitial lung disorders	0	0	0	0	0	1	0	0	0	0	0	0	0	0	0	0	0	0
7994	O/E - fibrosis of lung	0	0	0	0	0	1	0	0	0	0	0	0	0	0	0	0	0	0
2680	O/E - fibrosis of lung present	0	0	0	0	0	1	0	0	0	0	0	0	0	0	0	0	0	0
103637	Hypersensitivity pneumonitis	0	0	0	0	0	1	0	0	0	0	0	0	0	0	0	0	0	0
11942	Chronic laryngitis and laryngotracheitis	0	0	0	0	0	1	0	0	0	0	0	0	0	0	0	0	0	0
15693	Tuberculous bronchiectasis	0	0	0	0	0	1	0	0	0	0	0	0	0	0	0	0	0	0
18207	Allergic bronchitis NEC	0	0	0	0	0	1	0	0	0	0	0	0	0	0	0	0	0	0
91912	[X]Other specified interstitial pulmonary diseases	0	0	0	0	0	1	0	0	0	0	0	0	0	0	0	0	0	0
7791	Postinflammatory pulmonary fibrosis	0	0	0	0	0	1	0	0	0	0	0	0	0	0	0	0	0	0
63174	Hamman - Rich syndrome	0	0	0	0	0	1	0	0	0	0	0	0	0	0	0	0	0	0
8317	Interstitial lung disease NEC	0	0	0	0	0	1	0	0	0	0	0	0	0	0	0	0	0	0
11312	Extrinsic allergic alveolitis	0	0	0	0	0	1	0	0	0	0	0	0	0	0	0	0	0	0
16741	Tuberculous fibrosis of lung	0	0	0	0	0	1	0	0	0	0	0	0	0	0	0	0	0	0
63172	Pneumoconiosis associated with tuberculosis	0	0	0	0	0	1	0	0	0	0	0	0	0	0	0	0	0	0
56427	Congenital bronchiectasis	0	0	0	0	0	1	0	0	0	0	0	0	0	0	0	0	0	0
47142	Chronic respiratory conditions due to chemical fumes	0	0	0	0	0	1	0	0	0	0	0	0	0	0	0	0	0	0
100994	Asbestos-induced pleural plaque	0	0	0	0	0	1	0	0	0	0	0	0	0	0	0	0	0	0

43417	Chronic drug-induced interstitial lung disorders	0	0	0	0	0	1	0	0	0	0	0	0	0	0	0	0	0	0
70815	Chronic respiratory conditions due to chemical fumes NOS	0	0	0	0	0	1	0	0	0	0	0	0	0	0	0	0	0	0
44015	Drug-induced interstitial lung disorders	0	0	0	0	0	1	0	0	0	0	0	0	0	0	0	0	0	0
37365	Byssinosis	0	0	0	0	0	1	0	0	0	0	0	0	0	0	0	0	0	0
18914	Cystic fibrosis with pulmonary manifestations	0	0	0	0	0	1	0	0	0	0	0	0	0	0	0	0	0	0
92955	Acute vesicular emphysema	0	0	0	0	0	1	0	0	0	0	0	0	0	0	0	0	0	0
6837	Idiopathic fibrosing alveolitis	0	0	0	0	0	1	0	0	0	0	0	0	0	0	0	0	0	0
53943	Coffee-workers' lung	0	0	0	0	0	1	0	0	0	0	0	0	0	0	0	0	0	0
23446	Silica pneumoconiosis NOS	0	0	0	0	0	1	0	0	0	0	0	0	0	0	0	0	0	0
31447	Pigeon-fanciers' lung	0	0	0	0	0	1	0	0	0	0	0	0	0	0	0	0	0	0
28229	Idiopathic fibrosing alveolitis NOS	0	0	0	0	0	1	0	0	0	0	0	0	0	0	0	0	0	0
20364	Recurrent bronchiectasis	0	0	0	0	0	1	0	0	0	0	0	0	0	0	0	0	0	0
62442	Allergic extrinsic alveolitis NOS	0	0	0	0	0	1	0	0	0	0	0	0	0	0	0	0	0	0
30235	Siderosis	0	0	0	0	0	1	0	0	0	0	0	0	0	0	0	0	0	0
31423	Pneumoconiosis NOS	0	0	0	0	0	1	0	0	0	0	0	0	0	0	0	0	0	0
94894	Bauxite fibrosis of lung	0	0	0	0	0	1	0	0	0	0	0	0	0	0	0	0	0	0
55552	Other allergic alveolitis NOS	0	0	0	0	0	1	0	0	0	0	0	0	0	0	0	0	0	0
41491	Post-infective bronchiectasis	0	0	0	0	0	1	0	0	0	0	0	0	0	0	0	0	0	0
91989	Cheese-washers' lung	0	0	0	0	0	1	0	0	0	0	0	0	0	0	0	0	0	0
6051	Diffuse pulmonary fibrosis	0	0	0	0	0	1	0	0	0	0	0	0	0	0	0	0	0	0
60805	Talc pneumoconiosis	0	0	0	0	0	1	0	0	0	0	0	0	0	0	0	0	0	0
28853	Fibrosing alveolitis associated with rheumatoid arthritis	0	0	0	0	0	1	0	0	0	0	0	0	0	0	0	0	0	0
27345	Bird-fancier's lung	0	0	0	0	0	1	0	0	0	0	0	0	0	0	0	0	0	0
26442	Cannabinosis	0	0	0	0	0	1	0	0	0	0	0	0	0	0	0	0	0	0

62233	Simple silicosis	0	0	0	0	0	1	0	0	0	0	0	0	0	0	0	0	0	0
94575	Graphite fibrosis of lung	0	0	0	0	0	1	0	0	0	0	0	0	0	0	0	0	0	0
51858	Other allergic alveolitis	0	0	0	0	0	1	0	0	0	0	0	0	0	0	0	0	0	0
8303	Asbestosis	0	0	0	0	0	1	0	0	0	0	0	0	0	0	0	0	0	0
67709	Malt workers' lung	0	0	0	0	0	1	0	0	0	0	0	0	0	0	0	0	0	0
46460	Silica and silicate pneumoconiosis	0	0	0	0	0	1	0	0	0	0	0	0	0	0	0	0	0	0
23461	Pneumoconiosis due to inorganic dust NOS	0	0	0	0	0	1	0	0	0	0	0	0	0	0	0	0	0	0
71853	Complicated silicosis	0	0	0	0	0	1	0	0	0	0	0	0	0	0	0	0	0	0
2195	Bronchiectasis	0	0	0	0	0	1	0	0	0	0	0	0	0	0	0	0	0	0
51410	Asbestosis NOS	0	0	0	0	0	1	0	0	0	0	0	0	0	0	0	0	0	0
27348	Idiopathic pulmonary haemosiderosis	0	0	0	0	0	1	0	0	0	0	0	0	0	0	0	0	0	0
36240	Bird-fancier's lung NOS	0	0	0	0	0	1	0	0	0	0	0	0	0	0	0	0	0	0
49194	Berylliosis	0	0	0	0	0	1	0	0	0	0	0	0	0	0	0	0	0	0
65376	Pneumoconiosis due to other inorganic dust	0	0	0	0	0	1	0	0	0	0	0	0	0	0	0	0	0	0
5005	Pleural plaque disease due to asbestosis	0	0	0	0	0	1	0	0	0	0	0	0	0	0	0	0	0	0
93577	Stannosis	0	0	0	0	0	1	0	0	0	0	0	0	0	0	0	0	0	0
19492	Coal workers' pneumoconiosis	0	0	0	0	0	1	0	0	0	0	0	0	0	0	0	0	0	0
93206	Suberosis (cork-handlers' lung)	0	0	0	0	0	1	0	0	0	0	0	0	0	0	0	0	0	0
70286	Fish-meal workers' lung	0	0	0	0	0	1	0	0	0	0	0	0	0	0	0	0	0	0
47782	Chronic pulmonary fibrosis due to chemical fumes	0	0	0	0	0	1	0	0	0	0	0	0	0	0	0	0	0	0
32679	Bronchiectasis NOS	0	0	0	0	0	1	0	0	0	0	0	0	0	0	0	0	0	0
53095	Allergic alveolitis and pneumonitis NOS	0	0	0	0	0	1	0	0	0	0	0	0	0	0	0	0	0	0
56652	Maple bark strippers' lung	0	0	0	0	0	1	0	0	0	0	0	0	0	0	0	0	0	0
54893	Compensatory emphysema	0	0	0	0	0	1	0	0	0	0	0	0	0	0	0	0	0	0

11833	Hypersensitivity pneumonitis NOS	0	0	0	0	0	1	0	0	0	0	0	0	0	0	0	0	0	0
41694	Mushroom workers' lung	0	0	0	0	0	1	0	0	0	0	0	0	0	0	0	0	0	0
65060	[X]Other interstitial pulmonary diseases with fibrosis	0	0	0	0	0	1	0	0	0	0	0	0	0	0	0	0	0	0
69452	Air-conditioner and humidifier lung	0	0	0	0	0	1	0	0	0	0	0	0	0	0	0	0	0	0
22905	Interstitial emphysema	0	0	0	0	0	1	0	0	0	0	0	0	0	0	0	0	0	0
29966	Idiopathic pulmonary haemosiderosis	0	0	0	0	0	1	0	0	0	0	0	0	0	0	0	0	0	0
46977	Allergic alveolitis and pneumonitis NOS	0	0	0	0	0	1	0	0	0	0	0	0	0	0	0	0	0	0
22536	Chronic pulmonary fibrosis following radiation	0	0	0	0	0	1	0	0	0	0	0	0	0	0	0	0	0	0
89206	Massive silicotic fibrosis	0	0	0	0	0	1	0	0	0	0	0	0	0	0	0	0	0	0
62200	Bagassosis	0	0	0	0	0	1	0	0	0	0	0	0	0	0	0	0	0	0
15588	Farmers' lung	0	0	0	0	0	1	0	0	0	0	0	0	0	0	0	0	0	0
5909	Chronic wheezy bronchitis	0	0	0	0	0	1	0	0	0	0	0	0	0	0	0	0	0	0
70787	Atrophic (senile) emphysema	0	0	0	0	0	1	0	0	0	0	0	0	0	0	0	0	0	0
63216	Obliterative bronchiolitis due to chemical fumes	0	0	0	0	0	1	0	0	0	0	0	0	0	0	0	0	0	0
54822	Budgerigar-fanciers' lung	0	0	0	0	0	1	0	0	0	0	0	0	0	0	0	0	0	0
5519	Cryptogenic fibrosing alveolitis	0	0	0	0	0	1	0	0	0	0	0	0	0	0	0	0	0	0
98283	copd structured smoking assessment declined - enh serv admin	0	0	0	0	0	1	0	0	0	0	0	0	0	0	0	0	0	0
103955	Asthma trigger - tobacco smoke	0	0	0	0	0	1	0	0	0	0	0	0	0	0	0	0	0	0
105457	Chronic obstructive pulmonary disease care pathway	0	0	0	0	0	1	0	0	0	0	0	0	0	0	0	0	0	0
47993	Does not have asthma management plan	0	0	0	0	0	1	0	0	0	0	0	0	0	0	0	0	0	0
103813	Asthma trigger - cold air	0	0	0	0	0	1	0	0	0	0	0	0	0	0	0	0	0	0
103494	History of chronic obstructive pulmonary disease	0	0	0	0	0	1	0	0	0	0	0	0	0	0	0	0	0	0
4836	nocturnal cough / wheeze	0	0	0	0	0	1	0	0	0	0	0	0	0	0	0	0	0	0
51116	[X]Antiasthmats caus adverse effects in therapeut use, NEC	0	0	0	0	0	1	0	0	0	0	0	0	0	0	0	0	0	0

101133	[X] Adverse reaction to antiasthmatic NOS	0	0	0	0	0	1	0	0	0	0	0	0	0	0	0	0	0	0
104235	[X] Adverse reaction to antiasthmatics	0	0	0	0	0	1	0	0	0	0	0	0	0	0	0	0	0	0
103944	Asthma trigger - airborne dust	0	0	0	0	0	1	0	0	0	0	0	0	0	0	0	0	0	0
104608	End stage chronic obstructive airways disease	0	0	0	0	0	1	0	0	0	0	0	0	0	0	0	0	0	0
102871	asthma trigger - exercise	0	0	0	0	0	1	0	0	0	0	0	0	0	0	0	0	0	0
63233	Adverse reaction to antiasthmatic NOS	0	0	0	0	0	1	0	0	0	0	0	0	0	0	0	0	0	0
103612	Asthma never causes night symptoms	0	0	0	0	0	1	0	0	0	0	0	0	0	0	0	0	0	0
103945	Asthma trigger - damp	0	0	0	0	0	1	0	0	0	0	0	0	0	0	0	0	0	0
55816	Adverse reaction to antiasthmatics	0	0	0	0	0	1	0	0	0	0	0	0	0	0	0	0	0	0
106805	Chronic asthma with fixed airflow obstruction	0	0	0	0	0	1	0	0	0	0	0	0	0	0	0	0	0	0
103952	Asthma trigger - emotion	0	0	0	0	0	1	0	0	0	0	0	0	0	0	0	0	0	0
73408	Antiasthmatic poisoning NOS	0	0	0	0	0	1	0	0	0	0	0	0	0	0	0	0	0	0
103998	Asthma limits activities most days	0	0	0	0	0	1	0	0	0	0	0	0	0	0	0	0	0	0
23618	Chronic tracheitis	0	0	0	0	0	1	0	0	0	0	0	0	0	0	0	0	0	0
5867	Exercise induced asthma	0	0	0	0	0	1	0	0	0	0	0	0	0	0	0	0	0	0
19003	Emergency COPD admission since last appointment	0	0	0	0	0	1	0	0	0	0	0	0	0	0	0	0	0	0
29645	Asthma control step 0	0	0	0	0	0	1	0	0	0	0	0	0	0	0	0	0	0	0
102952	Asthma trigger - warm air	0	0	0	0	0	1	0	0	0	0	0	0	0	0	0	0	0	0
103321	Asthma trigger - animals	0	0	0	0	0	1	0	0	0	0	0	0	0	0	0	0	0	0
99536	Bullous emphysema with collapse	0	0	0	0	0	1	0	0	0	0	0	0	0	0	0	0	0	0
26501	Asthma never causes daytime symptoms	0	0	0	0	0	1	0	0	0	0	0	0	0	0	0	0	0	0
102301	asthma trigger - seasonal	0	0	0	0	0	1	0	0	0	0	0	0	0	0	0	0	0	0
102341	asthma trigger - pollen	0	0	0	0	0	1	0	0	0	0	0	0	0	0	0	0	0	0
102713	asthma limits activities 1 to 2 times per month	0	0	0	0	0	1	0	0	0	0	0	0	0	0	0	0	0	0

102449	asthma trigger - respiratory infection	0	0	0	0	0	1	0	0	0	0	0	0	0	0	0	0	0	0
38143	Asthma never disturbs sleep	0	0	0	0	0	1	0	0	0	0	0	0	0	0	0	0	0	0
20886	Asthma control step 4	0	0	0	0	0	1	0	0	0	0	0	0	0	0	0	0	0	0
24506	Further asthma - drug prevent.	0	0	0	0	0	1	0	0	0	0	0	0	0	0	0	0	0	0
102888	asthma limits activities 1 to 2 times per week	0	0	0	0	0	1	0	0	0	0	0	0	0	0	0	0	0	0
40864	[x] adverse reaction to theophylline - asthma	0	0	0	0	0	1	0	0	0	0	0	0	0	0	0	0	0	0
102395	Asthma causes symptoms most nights	0	0	0	0	0	1	0	0	0	0	0	0	0	0	0	0	0	0
103007	Chronic obstructive pulmonary disease 6 monthly review	0	0	0	0	0	1	0	0	0	0	0	0	0	0	0	0	0	0
24479	Emergency asthma admission since last appointment	0	0	0	0	0	1	0	0	0	0	0	0	0	0	0	0	0	0
45771	Chronic obstructive pulmonary disease does not disturb sleep	0	0	0	0	0	1	0	0	0	0	0	0	0	0	0	0	0	0
102400	asthma causes night time symptoms 1 to 2 times per week	0	0	0	0	0	1	0	0	0	0	0	0	0	0	0	0	0	0
18224	Asthma control step 3	0	0	0	0	0	1	0	0	0	0	0	0	0	0	0	0	0	0
13173	Asthma not disturbing sleep	0	0	0	0	0	1	0	0	0	0	0	0	0	0	0	0	0	0
26504	asthma never restricts exercise	0	0	0	0	0	1	0	0	0	0	0	0	0	0	0	0	0	0
26861	Asthma sometimes restricts exercise	0	0	0	0	0	1	0	0	0	0	0	0	0	0	0	0	0	0
13174	Asthma not limiting activities	0	0	0	0	0	1	0	0	0	0	0	0	0	0	0	0	0	0
48591	Adverse reaction to theophylline (asthma)	0	0	0	0	0	1	0	0	0	0	0	0	0	0	0	0	0	0
19106	COPD accident and emergency attendance since last visit	0	0	0	0	0	1	0	0	0	0	0	0	0	0	0	0	0	0
13065	moderate asthma	0	0	0	0	0	1	0	0	0	0	0	0	0	0	0	0	0	0
31225	Asthma causes daytime symptoms 1 to 2 times per month	0	0	0	0	0	1	0	0	0	0	0	0	0	0	0	0	0	0
11022	Asthma trigger	0	0	0	0	0	1	0	0	0	0	0	0	0	0	0	0	0	0
11019	Admit COPD emergency	0	0	0	0	0	1	0	0	0	0	0	0	0	0	0	0	0	0
11150	mucopurulent chronic bronchitis	0	0	0	0	0	1	0	0	0	0	0	0	0	0	0	0	0	0
9876	Severe chronic obstructive pulmonary disease	0	0	0	0	0	1	0	0	0	0	0	0	0	0	0	0	0	0

7146	Extrinsic (atopic) asthma	0	0	0	0	0	1	0	0	0	0	0	0	0	0	0	0	0	0
12166	Other specified chronic obstructive airways disease	0	0	0	0	0	1	0	0	0	0	0	0	0	0	0	0	0	0
29325	Intrinsic asthma without status asthmaticus	0	0	0	0	0	1	0	0	0	0	0	0	0	0	0	0	0	0
78	Asthma	0	0	0	0	0	1	0	0	0	0	0	0	0	0	0	0	0	0
13064	Asthma severity	0	0	0	0	0	1	0	0	0	0	0	0	0	0	0	0	0	0
185	acute exacerbation of asthma	0	0	0	0	0	1	0	0	0	0	0	0	0	0	0	0	0	0
232	Asthma attack	0	0	0	0	0	1	0	0	0	0	0	0	0	0	0	0	0	0
7416	asthma disturbing sleep	0	0	0	0	0	1	0	0	0	0	0	0	0	0	0	0	0	0
61513	Mucopurulent chronic bronchitis NOS	0	0	0	0	0	1	0	0	0	0	0	0	0	0	0	0	0	0
1208	childhood asthma	0	0	0	0	0	1	0	0	0	0	0	0	0	0	0	0	0	0
38146	Asthma disturbs sleep weekly	0	0	0	0	0	1	0	0	0	0	0	0	0	0	0	0	0	0
719	h/o: asthma	0	0	0	0	0	1	0	0	0	0	0	0	0	0	0	0	0	0
66058	[X]Other emphysema	0	0	0	0	0	1	0	0	0	0	0	0	0	0	0	0	0	0
10487	Asthma - currently active	0	0	0	0	0	1	0	0	0	0	0	0	0	0	0	0	0	0
27819	Obstructive chronic bronchitis	0	0	0	0	0	1	0	0	0	0	0	0	0	0	0	0	0	0
45782	Extrinsic asthma NOS	0	0	0	0	0	1	0	0	0	0	0	0	0	0	0	0	0	0
1446	acute exacerbation of chronic obstructive airways disease	0	0	0	0	0	1	0	0	0	0	0	0	0	0	0	0	0	0
68662	Zonal bullous emphysema	0	0	0	0	0	1	0	0	0	0	0	0	0	0	0	0	0	0
41020	Absent from work or school due to asthma	0	0	0	0	0	1	0	0	0	0	0	0	0	0	0	0	0	0
33450	Emphysema NOS	0	0	0	0	0	1	0	0	0	0	0	0	0	0	0	0	0	0
10802	moderate chronic obstructive pulmonary disease	0	0	0	0	0	1	0	0	0	0	0	0	0	0	0	0	0	0
7884	Chron obstruct pulmonary dis wth acute exacerbation, unspec	0	0	0	0	0	1	0	0	0	0	0	0	0	0	0	0	0	0
44525	Obstructive chronic bronchitis NOS	0	0	0	0	0	1	0	0	0	0	0	0	0	0	0	0	0	0
38145	Asthma limits walking on the flat	0	0	0	0	0	1	0	0	0	0	0	0	0	0	0	0	0	0
47684	detergent asthma	0	0	0	0	0	1	0	0	0	0	0	0	0	0	0	0	0	0

7229	Asthma prophylactic medication used	0	0	0	0	0	1	0	0	0	0	0	0	0	0	0	0	0	0
41017	aspirin induced asthma	0	0	0	0	0	1	0	0	0	0	0	0	0	0	0	0	0	0
7191	asthma limiting activities	0	0	0	0	0	1	0	0	0	0	0	0	0	0	0	0	0	0
3458	occasional asthma	0	0	0	0	0	1	0	0	0	0	0	0	0	0	0	0	0	0
56860	Segmental bullous emphysema	0	0	0	0	0	1	0	0	0	0	0	0	0	0	0	0	0	0
13175	asthma disturbs sleep frequently	0	0	0	0	0	1	0	0	0	0	0	0	0	0	0	0	0	0
998	Chronic obstructive airways disease	0	0	0	0	0	1	0	0	0	0	0	0	0	0	0	0	0	0
5798	Chronic asthmatic bronchitis	0	0	0	0	0	1	0	0	0	0	0	0	0	0	0	0	0	0
24884	Asthma causes daytime symptoms 1 to 2 times per week	0	0	0	0	0	1	0	0	0	0	0	0	0	0	0	0	0	0
10980	Centrilobular emphysema	0	0	0	0	0	1	0	0	0	0	0	0	0	0	0	0	0	0
3665	Late onset asthma	0	0	0	0	0	1	0	0	0	0	0	0	0	0	0	0	0	0
67040	Other specified chronic obstructive pulmonary disease	0	0	0	0	0	1	0	0	0	0	0	0	0	0	0	0	0	0
3366	Severe asthma	0	0	0	0	0	1	0	0	0	0	0	0	0	0	0	0	0	0
45073	Intrinsic asthma NOS	0	0	0	0	0	1	0	0	0	0	0	0	0	0	0	0	0	0
21232	Allergic asthma NEC	0	0	0	0	0	1	0	0	0	0	0	0	0	0	0	0	0	0
31167	Asthma night-time symptoms	0	0	0	0	0	1	0	0	0	0	0	0	0	0	0	0	0	0
63479	MacLeod's unilateral emphysema	0	0	0	0	0	1	0	0	0	0	0	0	0	0	0	0	0	0
794	Emphysema	0	0	0	0	0	1	0	0	0	0	0	0	0	0	0	0	0	0
15157	chronic bronchitis nos	0	0	0	0	0	1	0	0	0	0	0	0	0	0	0	0	0	0
7058	Emergency admission, asthma	0	0	0	0	0	1	0	0	0	0	0	0	0	0	0	0	0	0
40159	Purulent chronic bronchitis	0	0	0	0	0	1	0	0	0	0	0	0	0	0	0	0	0	0
66043	Other chronic bronchitis	0	0	0	0	0	1	0	0	0	0	0	0	0	0	0	0	0	0
3018	mild asthma	0	0	0	0	0	1	0	0	0	0	0	0	0	0	0	0	0	0
15248	Hay fever with asthma	0	0	0	0	0	1	0	0	0	0	0	0	0	0	0	0	0	0
24248	Mixed simple and mucopurulent chronic bronchitis	0	0	0	0	0	1	0	0	0	0	0	0	0	0	0	0	0	0
4892	status asthmaticus nos	0	0	0	0	0	1	0	0	0	0	0	0	0	0	0	0	0	0

233	Severe asthma attack	0	0	0	0	0	1	0	0	0	0	0	0	0	0	0	0	0	0
45770	Chronic obstructive pulmonary disease disturbs sleep	0	0	0	0	0	1	0	0	0	0	0	0	0	0	0	0	0	0
3243	Chronic bronchitis	0	0	0	0	0	1	0	0	0	0	0	0	0	0	0	0	0	0
93568	Very severe chronic obstructive pulmonary disease	0	0	0	0	0	1	0	0	0	0	0	0	0	0	0	0	0	0
21061	Chronic obstruct pulmonary dis with acute lower resp infectn	0	0	0	0	0	1	0	0	0	0	0	0	0	0	0	0	0	0
1001	Chronic obstructive pulmonary disease	0	0	0	0	0	1	0	0	0	0	0	0	0	0	0	0	0	0
22752	Occupational asthma	0	0	0	0	0	1	0	0	0	0	0	0	0	0	0	0	0	0
30815	Asthma causing night waking	0	0	0	0	0	1	0	0	0	0	0	0	0	0	0	0	0	0
10863	Mild chronic obstructive pulmonary disease	0	0	0	0	0	1	0	0	0	0	0	0	0	0	0	0	0	0
4442	Asthma unspecified	0	0	0	0	0	1	0	0	0	0	0	0	0	0	0	0	0	0
64721	Chronic emphysema due to chemical fumes	0	0	0	0	0	1	0	0	0	0	0	0	0	0	0	0	0	0
60188	giant bullous emphysema	0	0	0	0	0	1	0	0	0	0	0	0	0	0	0	0	0	0
6707	Extrinsic asthma with asthma attack	0	0	0	0	0	1	0	0	0	0	0	0	0	0	0	0	0	0
14777	Extrinsic asthma without status asthmaticus	0	0	0	0	0	1	0	0	0	0	0	0	0	0	0	0	0	0
26306	Chronic bullous emphysema	0	0	0	0	0	1	0	0	0	0	0	0	0	0	0	0	0	0
1555	bronchial asthma	0	0	0	0	0	1	0	0	0	0	0	0	0	0	0	0	0	0
16070	Asthma NOS	0	0	0	0	0	1	0	0	0	0	0	0	0	0	0	0	0	0
26506	Asthma severely restricts exercise	0	0	0	0	0	1	0	0	0	0	0	0	0	0	0	0	0	0
68066	Other chronic bronchitis NOS	0	0	0	0	0	1	0	0	0	0	0	0	0	0	0	0	0	0
4606	Exercise induced asthma	0	0	0	0	0	1	0	0	0	0	0	0	0	0	0	0	0	0
25181	asthma restricts exercise	0	0	0	0	0	1	0	0	0	0	0	0	0	0	0	0	0	0
18323	Intrinsic asthma with asthma attack	0	0	0	0	0	1	0	0	0	0	0	0	0	0	0	0	0	0
11370	Asthma confirmed	0	0	0	0	0	1	0	0	0	0	0	0	0	0	0	0	0	0
65733	[x]other specified chronic obstructive pulmonary disease	0	0	0	0	0	1	0	0	0	0	0	0	0	0	0	0	0	0
45089	Chronic tracheobronchitis	0	0	0	0	0	1	0	0	0	0	0	0	0	0	0	0	0	0

11839	Asthma resolved	0	0	0	0	0	1	0	0	0	0	0	0	0	0	0	0	0	0
10996	Asthma resolved	0	0	0	0	0	1	0	0	0	0	0	0	0	0	0	0	0	0
23492	Chronic bullous emphysema NOS	0	0	0	0	0	1	0	0	0	0	0	0	0	0	0	0	0	0
16410	Other emphysema NOS	0	0	0	0	0	1	0	0	0	0	0	0	0	0	0	0	0	0
7731	Pollen asthma	0	0	0	0	0	1	0	0	0	0	0	0	0	0	0	0	0	0
15626	Chronic catarrhal bronchitis	0	0	0	0	0	1	0	0	0	0	0	0	0	0	0	0	0	0
37247	chronic obstructive pulmonary disease nos	0	0	0	0	0	1	0	0	0	0	0	0	0	0	0	0	0	0
25796	mixed asthma	0	0	0	0	0	1	0	0	0	0	0	0	0	0	0	0	0	0
2290	Allergic asthma	0	0	0	0	0	1	0	0	0	0	0	0	0	0	0	0	0	0
5710	Chronic obstructive airways disease NOS	0	0	0	0	0	1	0	0	0	0	0	0	0	0	0	0	0	0
39478	Wood asthma	0	0	0	0	0	1	0	0	0	0	0	0	0	0	0	0	0	0
40823	Brittle asthma	0	0	0	0	0	1	0	0	0	0	0	0	0	0	0	0	0	0
37959	fetid chronic bronchitis	0	0	0	0	0	1	0	0	0	0	0	0	0	0	0	0	0	0
27926	Extrinsic asthma with status asthmaticus	0	0	0	0	0	1	0	0	0	0	0	0	0	0	0	0	0	0
40788	Other emphysema	0	0	0	0	0	1	0	0	0	0	0	0	0	0	0	0	0	0
46036	multiple copd emergency hospital admissions	0	0	0	0	0	1	0	0	0	0	0	0	0	0	0	0	0	0
46578	panlobular emphysema	0	0	0	0	0	1	0	0	0	0	0	0	0	0	0	0	0	0
58196	Intrinsic asthma with status asthmaticus	0	0	0	0	0	1	0	0	0	0	0	0	0	0	0	0	0	0
73522	work aggravated asthma	0	0	0	0	0	1	0	0	0	0	0	0	0	0	0	0	0	0
14798	emphysematous bronchitis	0	0	0	0	0	1	0	0	0	0	0	0	0	0	0	0	0	0
38144	Asthma limits walking up hills or stairs	0	0	0	0	0	1	0	0	0	0	0	0	0	0	0	0	0	0
5267	Intrinsic asthma	0	0	0	0	0	1	0	0	0	0	0	0	0	0	0	0	0	0
13066	Asthma - currently dormant	0	0	0	0	0	1	0	0	0	0	0	0	0	0	0	0	0	0
39570	Asthma causes night symptoms 1 to 2 times per month	0	0	0	0	0	1	0	0	0	0	0	0	0	0	0	0	0	0

93353	Sequoiosis (red-cedar asthma)	0	0	0	0	0	1	0	0	0	0	0	0	0	0	0	0	0	0
61118	Simple chronic bronchitis NOS	0	0	0	0	0	1	0	0	0	0	0	0	0	0	0	0	0	0
42824	Asthma daytime symptoms	0	0	0	0	0	1	0	0	0	0	0	0	0	0	0	0	0	0
5627	Hay fever with asthma	0	0	0	0	0	1	0	0	0	0	0	0	0	0	0	0	0	0
25603	simple chronic bronchitis	0	0	0	0	0	1	0	0	0	0	0	0	0	0	0	0	0	0
26503	Asthma causes daytime symptoms most days	0	0	0	0	0	1	0	0	0	0	0	0	0	0	0	0	0	0
8335	Asthma attack NOS	0	0	0	0	0	1	0	0	0	0	0	0	0	0	0	0	0	0
12987	Late-onset asthma	0	0	0	0	0	1	0	0	0	0	0	0	0	0	0	0	0	0
18476	COPD follow-up	0	0	0	0	0	1	0	0	0	0	0	0	0	0	0	0	0	0
102685	Chronic obstructive pulmonary disease 3 monthly review	0	0	0	0	0	1	0	0	0	0	0	0	0	0	0	0	0	0
10043	asthma annual review	0	0	0	0	0	1	0	0	0	0	0	0	0	0	0	0	0	0
47337	Asthma accident and emergency attendance since last visit	0	0	0	0	0	1	0	0	0	0	0	0	0	0	0	0	0	0
105420	Asthma self-management plan review	0	0	0	0	0	1	0	0	0	0	0	0	0	0	0	0	0	0
104169	COPD self-management plan review	0	0	0	0	0	1	0	0	0	0	0	0	0	0	0	0	0	0
105674	Asthma self-management plan agreed	0	0	0	0	0	1	0	0	0	0	0	0	0	0	0	0	0	0
104265	GP OOH service notified of COPD care plan	0	0	0	0	0	1	0	0	0	0	0	0	0	0	0	0	0	0
104481	Has chronic obstructive pulmonary disease care plan	0	0	0	0	0	1	0	0	0	0	0	0	0	0	0	0	0	0
103678	Chronic obstructiv pulmonary disease medication optimisation	0	0	0	0	0	1	0	0	0	0	0	0	0	0	0	0	0	0
103558	Preferred place of care for next exacerbation of COPD	0	0	0	0	0	1	0	0	0	0	0	0	0	0	0	0	0	0
11287	Chronic obstructive pulmonary disease annual review	0	0	0	0	0	1	0	0	0	0	0	0	0	0	0	0	0	0
10274	Asthma medication review	0	0	0	0	0	1	0	0	0	0	0	0	0	0	0	0	0	0
98284	Refer COPD structured smoking assessment - enhanc serv admin	0	0	0	0	0	1	0	0	0	0	0	0	0	0	0	0	0	0
45777	chronic obstructive pulmonary disease clini management plan	0	0	0	0	0	1	0	0	0	0	0	0	0	0	0	0	0	0
19520	asthma treatment compliance satisfactory	0	0	0	0	0	1	0	0	0	0	0	0	0	0	0	0	0	0

19519	asthma treatment compliance unsatisfactory	0	0	0	0	0	1	0	0	0	0	0	0	0	0	0	0	0	0
99948	copd patient unsuitable for pulmonary rehab - enh serv admin	0	0	0	0	0	1	0	0	0	0	0	0	0	0	0	0	0	0
99793	patient has a written asthma personal action plan	0	0	0	0	0	1	0	0	0	0	0	0	0	0	0	0	0	0
9663	step up change in asthma management plan	0	0	0	0	0	1	0	0	0	0	0	0	0	0	0	0	0	0
18223	Step down change in asthma management plan	0	0	0	0	0	1	0	0	0	0	0	0	0	0	0	0	0	0
103400	referred for copd structured smoking assessment	0	0	0	0	0	1	0	0	0	0	0	0	0	0	0	0	0	0
9018	number of asthma exacerbations in past year	0	0	0	0	0	1	0	0	0	0	0	0	0	0	0	0	0	0
16667	asthma control step 2	0	0	0	0	0	1	0	0	0	0	0	0	0	0	0	0	0	0
7378	asthma management plan given	0	0	0	0	0	1	0	0	0	0	0	0	0	0	0	0	0	0
20860	asthma control step 5	0	0	0	0	0	1	0	0	0	0	0	0	0	0	0	0	0	0
9552	Change in asthma management plan	0	0	0	0	0	1	0	0	0	0	0	0	0	0	0	0	0	0
101042	issue of chronic obstructive pulmonary disease rescue pack	0	0	0	0	0	1	0	0	0	0	0	0	0	0	0	0	0	0
16785	asthma control step 1	0	0	0	0	0	1	0	0	0	0	0	0	0	0	0	0	0	0
18621	chronic obstructive pulmonary disease follow-up	0	0	0	0	0	1	0	0	0	0	0	0	0	0	0	0	0	0
25791	Asthma clinical management plan	0	0	0	0	0	1	0	0	0	0	0	0	0	0	0	0	0	0
42624	coad follow-up	0	0	0	0	0	1	0	0	0	0	0	0	0	0	0	0	0	0
104117	COPD self-management plan agreed	0	0	0	0	0	1	0	0	0	0	0	0	0	0	0	0	0	0
103864	COPD patient unsuitable for pulmonary rehabilitation	0	0	0	0	0	1	0	0	0	0	0	0	0	0	0	0	0	0
106945	Chronic obstructive pulmonary disease rescue pack declined	0	0	0	0	0	1	0	0	0	0	0	0	0	0	0	0	0	0
104985	On chronic obstructive pulmonary disease supprtv cre pathway	0	0	0	0	0	1	0	0	0	0	0	0	0	0	0	0	0	0
104710	On COPD (chr obstruc pulmonary disease) supportv cre pathway	0	0	0	0	0	1	0	0	0	0	0	0	0	0	0	0	0	0
104998	Chronic obstructve pulmonry disease rescue pack not indicatd	0	0	0	0	0	1	0	0	0	0	0	0	0	0	0	0	0	0
18501	copd self-management plan given	0	0	0	0	0	1	0	0	0	0	0	0	0	0	0	0	0	0
28743	Number of COPD exacerbations in past year	0	0	0	0	0	1	0	0	0	0	0	0	0	0	0	0	0	0

107167	Number days absent from school due to asthma in past 6 month	0	0	0	0	0	1	0	0	0	0	0	0	0	0	0	0	0	0
13176	asthma follow-up	0	0	0	0	0	1	0	0	0	0	0	0	0	0	0	0	0	0
844	Rheumatoid arthritis	0	0	0	0	0	0	1	0	0	0	0	0	0	0	0	0	0	0
1408	Polymyalgia rheumatica	0	0	0	0	0	0	1	0	0	0	0	0	0	0	0	0	0	0
1471	Polyarteritis nodosa	0	0	0	0	0	0	1	0	0	0	0	0	0	0	0	0	0	0
2360	Sicca (Sjogren's) syndrome	0	0	0	0	0	0	1	0	0	0	0	0	0	0	0	0	0	0
3042	Polymyalgia	0	0	0	0	0	0	1	0	0	0	0	0	0	0	0	0	0	0
3388	Hypermobility syndrome	0	0	0	0	0	0	1	0	0	0	0	0	0	0	0	0	0	0
3670	Scleroderma	0	0	0	0	0	0	1	0	0	0	0	0	0	0	0	0	0	0
3859	Pulmonary sarcoidosis	0	0	0	0	0	0	1	0	0	0	0	0	0	0	0	0	0	0
3865	Sarcoidosis	0	0	0	0	0	0	1	0	0	0	0	0	0	0	0	0	0	0
3944	Chronic post-rheumatic arthropathy	0	0	0	0	0	0	1	0	0	0	0	0	0	0	0	0	0	0
4125	Lupus erythematosus	0	0	0	0	0	0	1	0	0	0	0	0	0	0	0	0	0	0
4186	Juvenile rheumatoid arthritis - Still's disease	0	0	0	0	0	0	1	0	0	0	0	0	0	0	0	0	0	0
4502	Rheumatoid factor positive	0	0	0	0	0	0	1	0	0	0	0	0	0	0	0	0	0	0
4810	Wegener's granulomatosis	0	0	0	0	0	0	1	0	0	0	0	0	0	0	0	0	0	0
4908	Dermatomyositis	0	0	0	0	0	0	1	0	0	0	0	0	0	0	0	0	0	0
5723	Rheumatoid nodule	0	0	0	0	0	0	1	0	0	0	0	0	0	0	0	0	0	0
6157	Kawasaki disease	0	0	0	0	0	0	1	0	0	0	0	0	0	0	0	0	0	0
6639	H/O: rheumatoid arthritis	0	0	0	0	0	0	1	0	0	0	0	0	0	0	0	0	0	0
6916	Seronegative rheumatoid arthritis	0	0	0	0	0	0	1	0	0	0	0	0	0	0	0	0	0	0
7196	Other juvenile arthritis	0	0	0	0	0	0	1	0	0	0	0	0	0	0	0	0	0	0
7522	Lupus erythematosus NOS	0	0	0	0	0	0	1	0	0	0	0	0	0	0	0	0	0	0
7871	Systemic lupus erythematosus	0	0	0	0	0	0	1	0	0	0	0	0	0	0	0	0	0	0
8350	Flare of rheumatoid arthritis	0	0	0	0	0	0	1	0	0	0	0	0	0	0	0	0	0	0
9707	Seropositive errosive rheumatoid arthritis	0	0	0	0	0	0	1	0	0	0	0	0	0	0	0	0	0	0
9954	Rheumatoid lung	0	0	0	0	0	0	1	0	0	0	0	0	0	0	0	0	0	0
10432	Giant cell arteritis	0	0	0	0	0	0	1	0	0	0	0	0	0	0	0	0	0	0
11920	Systemic lupus erythematosus with pericarditis	0	0	0	0	0	0	1	0	0	0	0	0	0	0	0	0	0	0

12019	Seropositive rheumatoid arthritis, unspecified	0	0	0	0	0	0	1	0	0	0	0	0	0	0	0	0	0	0
12575	Juvenile arthritis in Crohn's disease	0	0	0	0	0	0	1	0	0	0	0	0	0	0	0	0	0	0
15511	Polymyositis	0	0	0	0	0	0	1	0	0	0	0	0	0	0	0	0	0	0
16235	Behcet's syndrome	0	0	0	0	0	0	1	0	0	0	0	0	0	0	0	0	0	0
17675	CREST syndrome	0	0	0	0	0	0	1	0	0	0	0	0	0	0	0	0	0	0
109815	Interstitial lung disease due to collagen vascular disease	0	0	0	0	0	0	1	0	0	0	0	0	0	0	0	0	0	0
104915	Interstitial lung disease due to connective tissue disease	0	0	0	0	0	0	1	0	0	0	0	0	0	0	0	0	0	0
18155	Rheumatoid bursitis	0	0	0	0	0	0	1	0	0	0	0	0	0	0	0	0	0	0
18380	Juvenile polyarteritis	0	0	0	0	0	0	1	0	0	0	0	0	0	0	0	0	0	0
20007	Disseminated lupus erythematosus	0	0	0	0	0	0	1	0	0	0	0	0	0	0	0	0	0	0
20271	Other specified diffuse collagen diseases	0	0	0	0	0	0	1	0	0	0	0	0	0	0	0	0	0	0
20509	Hypersensitivity angiitis	0	0	0	0	0	0	1	0	0	0	0	0	0	0	0	0	0	0
20615	Inflammatory polyarthropathy	0	0	0	0	0	0	1	0	0	0	0	0	0	0	0	0	0	0
21022	Myopathy due to Sjogren's disease	0	0	0	0	0	0	1	0	0	0	0	0	0	0	0	0	0	0
21151	Collagen disease NOS	0	0	0	0	0	0	1	0	0	0	0	0	0	0	0	0	0	0
21358	Rheumatoid arthritis of shoulder	0	0	0	0	0	0	1	0	0	0	0	0	0	0	0	0	0	0
21533	Pauciarticular juvenile rheumatoid arthritis	0	0	0	0	0	0	1	0	0	0	0	0	0	0	0	0	0	0
21602	Churg-Strauss vasculitis	0	0	0	0	0	0	1	0	0	0	0	0	0	0	0	0	0	0
21697	Thrombotic thrombocytopenic purpura	0	0	0	0	0	0	1	0	0	0	0	0	0	0	0	0	0	0
22205	Lupus nephritis	0	0	0	0	0	0	1	0	0	0	0	0	0	0	0	0	0	0
22509	Collagen diseases	0	0	0	0	0	0	1	0	0	0	0	0	0	0	0	0	0	0
23552	Felty's syndrome	0	0	0	0	0	0	1	0	0	0	0	0	0	0	0	0	0	0
23569	Hypersensitivity angiitis NOS	0	0	0	0	0	0	1	0	0	0	0	0	0	0	0	0	0	0
23657	Keratoconjunctivitis sicca (excluding Sjogren's syndrome)	0	0	0	0	0	0	1	0	0	0	0	0	0	0	0	0	0	0

23833	Other specified inflammatory polyarthropathy NOS	0	0	0	0	0	0	1	0	0	0	0	0	0	0	0	0	0	0
23834	Adult Still's Disease	0	0	0	0	0	0	1	0	0	0	0	0	0	0	0	0	0	0
24747	Inflammatory polyarthropathy NOS	0	0	0	0	0	0	1	0	0	0	0	0	0	0	0	0	0	0
25390	Subacute cutaneous lupus erythematosus	0	0	0	0	0	0	1	0	0	0	0	0	0	0	0	0	0	0
26405	Hepatic granulomas in sarcoidosis	0	0	0	0	0	0	1	0	0	0	0	0	0	0	0	0	0	0
26410	Autoimmune disease NOS	0	0	0	0	0	0	1	0	0	0	0	0	0	0	0	0	0	0
26860	Goodpasture's syndrome	0	0	0	0	0	0	1	0	0	0	0	0	0	0	0	0	0	0
27557	Juvenile rheumatoid arthritis NOS	0	0	0	0	0	0	1	0	0	0	0	0	0	0	0	0	0	0
27603	Rheumatoid arthritis and other inflammatory polyarthropathy	0	0	0	0	0	0	1	0	0	0	0	0	0	0	0	0	0	0
27769	Sarcoidosis of skin	0	0	0	0	0	0	1	0	0	0	0	0	0	0	0	0	0	0
28316	Dermatopolymyositis, unspecified	0	0	0	0	0	0	1	0	0	0	0	0	0	0	0	0	0	0
28417	Systemic sclerosis	0	0	0	0	0	0	1	0	0	0	0	0	0	0	0	0	0	0
28456	Juvenile arthritis in psoriasis	0	0	0	0	0	0	1	0	0	0	0	0	0	0	0	0	0	0
28952	Lupus pernio	0	0	0	0	0	0	1	0	0	0	0	0	0	0	0	0	0	0
29472	Giant cell arteritis with polymyalgia rheumatica	0	0	0	0	0	0	1	0	0	0	0	0	0	0	0	0	0	0
29519	Systemic lupus erythematosus with organ or sys involv	0	0	0	0	0	0	1	0	0	0	0	0	0	0	0	0	0	0
30532	Necrotising vasculopathy, unspecified	0	0	0	0	0	0	1	0	0	0	0	0	0	0	0	0	0	0
30548	Rheumatoid vasculitis	0	0	0	0	0	0	1	0	0	0	0	0	0	0	0	0	0	0
31054	Rheumatoid arthritis - multiple joint	0	0	0	0	0	0	1	0	0	0	0	0	0	0	0	0	0	0
31181	Juvenile seronegative polyarthritis	0	0	0	0	0	0	1	0	0	0	0	0	0	0	0	0	0	0
31209	Myopathy due to rheumatoid arthritis	0	0	0	0	0	0	1	0	0	0	0	0	0	0	0	0	0	0
31360	Juvenile rheumatoid arthritis	0	0	0	0	0	0	1	0	0	0	0	0	0	0	0	0	0	0
31564	Lung disease with systemic lupus erythematosus	0	0	0	0	0	0	1	0	0	0	0	0	0	0	0	0	0	0
31724	Rheumatoid lung	0	0	0	0	0	0	1	0	0	0	0	0	0	0	0	0	0	0

32001	Adult-onset Still's disease	0	0	0	0	0	0	1	0	0	0	0	0	0	0	0	0	0	0
32649	Juvenile dermatomyositis	0	0	0	0	0	0	1	0	0	0	0	0	0	0	0	0	0	0
33264	O/E-hands-rheumatoid spindling	0	0	0	0	0	0	1	0	0	0	0	0	0	0	0	0	0	0
33449	Lupus erythematosus chronicus	0	0	0	0	0	0	1	0	0	0	0	0	0	0	0	0	0	0
33980	Sarcoidosis of lung	0	0	0	0	0	0	1	0	0	0	0	0	0	0	0	0	0	0
34437	Sarcoid myocarditis	0	0	0	0	0	0	1	0	0	0	0	0	0	0	0	0	0	0
34780	Ulceration of vulva in Behcet's disease	0	0	0	0	0	0	1	0	0	0	0	0	0	0	0	0	0	0
35315	Arthropathy due to haemophilia	0	0	0	0	0	0	1	0	0	0	0	0	0	0	0	0	0	0
36276	Monarticular juvenile rheumatoid arthritis	0	0	0	0	0	0	1	0	0	0	0	0	0	0	0	0	0	0
36597	Other specified inflammatory polyarthropathy	0	0	0	0	0	0	1	0	0	0	0	0	0	0	0	0	0	0
36942	Drug-induced systemic lupus erythematosus	0	0	0	0	0	0	1	0	0	0	0	0	0	0	0	0	0	0
37431	Rheumatoid arthropathy + visceral/systemic involvement NOS	0	0	0	0	0	0	1	0	0	0	0	0	0	0	0	0	0	0
37640	Takayasu's disease	0	0	0	0	0	0	1	0	0	0	0	0	0	0	0	0	0	0
37747	Thrombotic microangiopathy	0	0	0	0	0	0	1	0	0	0	0	0	0	0	0	0	0	0
40387	Poikilodermatomyositis	0	0	0	0	0	0	1	0	0	0	0	0	0	0	0	0	0	0
40613	Sarcoid arthropathy	0	0	0	0	0	0	1	0	0	0	0	0	0	0	0	0	0	0
40751	Polyneuropathy in sarcoidosis	0	0	0	0	0	0	1	0	0	0	0	0	0	0	0	0	0	0
40797	Lupus erythematosus migrans	0	0	0	0	0	0	1	0	0	0	0	0	0	0	0	0	0	0
41148	Renal tubulo-interstitial disorder in SLE	0	0	0	0	0	0	1	0	0	0	0	0	0	0	0	0	0	0
41941	Rheumatoid arthritis of PIP joint of finger	0	0	0	0	0	0	1	0	0	0	0	0	0	0	0	0	0	0
42299	Rheumatoid arthritis of MCP joint	0	0	0	0	0	0	1	0	0	0	0	0	0	0	0	0	0	0
42405	Juvenile ankylosing spondylitis	0	0	0	0	0	0	1	0	0	0	0	0	0	0	0	0	0	0
42719	Systemic lupus erythematosus NOS	0	0	0	0	0	0	1	0	0	0	0	0	0	0	0	0	0	0
28853	Fibrosing alveolitis associated with rheumatoid arthritis	0	0	0	0	0	0	1	0	0	0	0	0	0	0	0	0	0	0

42940	Lung disease with polymyositis	0	0	0	0	0	0	1	0	0	0	0	0	0	0	0	0	0	0
43816	Rheumatoid carditis	0	0	0	0	0	0	1	0	0	0	0	0	0	0	0	0	0	0
44095	Polyneuropathy in disseminated lupus erythematosus	0	0	0	0	0	0	1	0	0	0	0	0	0	0	0	0	0	0
44141	Progressive systemic sclerosis	0	0	0	0	0	0	1	0	0	0	0	0	0	0	0	0	0	0
44203	Other rheumatoid arthritis of spine	0	0	0	0	0	0	1	0	0	0	0	0	0	0	0	0	0	0
44535	Haemophilic arthropathy	0	0	0	0	0	0	1	0	0	0	0	0	0	0	0	0	0	0
44743	Rheumatoid arthritis of cervical spine	0	0	0	0	0	0	1	0	0	0	0	0	0	0	0	0	0	0
46436	Rheumatoid lung disease	0	0	0	0	0	0	1	0	0	0	0	0	0	0	0	0	0	0
46622	Pauciarticular onset juvenile chronic arthritis	0	0	0	0	0	0	1	0	0	0	0	0	0	0	0	0	0	0
47037	Sarcoid heart disease	0	0	0	0	0	0	1	0	0	0	0	0	0	0	0	0	0	0
47364	Lung disease with Sjogren's disease	0	0	0	0	0	0	1	0	0	0	0	0	0	0	0	0	0	0
47465	Polyneuropathy in polyarteritis nodosa	0	0	0	0	0	0	1	0	0	0	0	0	0	0	0	0	0	0
47672	Nephrotic syndrome in systemic lupus erythematosus	0	0	0	0	0	0	1	0	0	0	0	0	0	0	0	0	0	0
47718	Myositis in sarcoidosis	0	0	0	0	0	0	1	0	0	0	0	0	0	0	0	0	0	0
47831	Acute polyarticular juvenile rheumatoid arthritis	0	0	0	0	0	0	1	0	0	0	0	0	0	0	0	0	0	0
48832	Rheumatoid arthritis of wrist	0	0	0	0	0	0	1	0	0	0	0	0	0	0	0	0	0	0
49067	Rheumatoid arthritis of hip	0	0	0	0	0	0	1	0	0	0	0	0	0	0	0	0	0	0
49075	Sarcoidosis of lymph nodes	0	0	0	0	0	0	1	0	0	0	0	0	0	0	0	0	0	0
49227	Other rheumatoid arthropathy + visceral/systemic involvement	0	0	0	0	0	0	1	0	0	0	0	0	0	0	0	0	0	0
49454	Meningitis due to sarcoidosis	0	0	0	0	0	0	1	0	0	0	0	0	0	0	0	0	0	0
49787	Rheumatoid myocarditis	0	0	0	0	0	0	1	0	0	0	0	0	0	0	0	0	0	0
50644	Juvenile rheumatoid arthropathy unspecified	0	0	0	0	0	0	1	0	0	0	0	0	0	0	0	0	0	0
50863	Rheumatoid arthritis of knee	0	0	0	0	0	0	1	0	0	0	0	0	0	0	0	0	0	0
51238	Rheumatoid arthritis of 1st MTP joint	0	0	0	0	0	0	1	0	0	0	0	0	0	0	0	0	0	0

51239	Rheumatoid arthritis of ankle	0	0	0	0	0	0	1	0	0	0	0	0	0	0	0	0	0	0
52210	Weber - Christian disease	0	0	0	0	0	0	1	0	0	0	0	0	0	0	0	0	0	0
52519	Myopathy due to sarcoidosis	0	0	0	0	0	0	1	0	0	0	0	0	0	0	0	0	0	0
52604	Lethal midline granuloma	0	0	0	0	0	0	1	0	0	0	0	0	0	0	0	0	0	0
52860	[X]Systemic connective tissue disorders	0	0	0	0	0	0	1	0	0	0	0	0	0	0	0	0	0	0
53621	Rheumatoid nodule	0	0	0	0	0	0	1	0	0	0	0	0	0	0	0	0	0	0
53789	[X]Other giant cell arteritis	0	0	0	0	0	0	1	0	0	0	0	0	0	0	0	0	0	0
53862	[X]Other dermatomyositis	0	0	0	0	0	0	1	0	0	0	0	0	0	0	0	0	0	0
54011	Weber - Christian disease	0	0	0	0	0	0	1	0	0	0	0	0	0	0	0	0	0	0
55601	Myopathy due to scleroderma	0	0	0	0	0	0	1	0	0	0	0	0	0	0	0	0	0	0
55612	Multiple cranial nerve palsies in sarcoidosis	0	0	0	0	0	0	1	0	0	0	0	0	0	0	0	0	0	0
56202	[X]Seropositive rheumatoid arthritis	0	0	0	0	0	0	1	0	0	0	0	0	0	0	0	0	0	0
56838	Caplan's syndrome	0	0	0	0	0	0	1	0	0	0	0	0	0	0	0	0	0	0
57313	Polyneuropathy in collagen vascular disease	0	0	0	0	0	0	1	0	0	0	0	0	0	0	0	0	0	0
57675	Libman-Sacks disease	0	0	0	0	0	0	1	0	0	0	0	0	0	0	0	0	0	0
57888	Myopathy due to polyarteritis nodosa	0	0	0	0	0	0	1	0	0	0	0	0	0	0	0	0	0	0
58706	[X]Other forms of systemic lupus erythematosus	0	0	0	0	0	0	1	0	0	0	0	0	0	0	0	0	0	0
58750	Nephrotic syndrome in polyarteritis nodosa	0	0	0	0	0	0	1	0	0	0	0	0	0	0	0	0	0	0
58818	Behcet's syndrome arthropathy	0	0	0	0	0	0	1	0	0	0	0	0	0	0	0	0	0	0
58841	Sarcoidosis of lung with sarcoidosis of lymph nodes	0	0	0	0	0	0	1	0	0	0	0	0	0	0	0	0	0	0
59738	Rheumatoid arthritis of elbow	0	0	0	0	0	0	1	0	0	0	0	0	0	0	0	0	0	0
62323	Polyarteritis nodosa and allied conditions	0	0	0	0	0	0	1	0	0	0	0	0	0	0	0	0	0	0
62401	Polyneuropathy in rheumatoid arthritis	0	0	0	0	0	0	1	0	0	0	0	0	0	0	0	0	0	0
62652	[X]Other specified necrotizing vasculopathies	0	0	0	0	0	0	1	0	0	0	0	0	0	0	0	0	0	0

62867	Arthropathy in Behcet's syndrome of multiple sites	0	0	0	0	0	0	1	0	0	0	0	0	0	0	0	0	0	0
63198	Rheumatoid arthritis of DIP joint of finger	0	0	0	0	0	0	1	0	0	0	0	0	0	0	0	0	0	0
63365	Rheumatoid arthritis of distal radio-ulnar joint	0	0	0	0	0	0	1	0	0	0	0	0	0	0	0	0	0	0
65391	Lupus erythematosus nodularis	0	0	0	0	0	0	1	0	0	0	0	0	0	0	0	0	0	0
67149	[X]Other overlap syndromes	0	0	0	0	0	0	1	0	0	0	0	0	0	0	0	0	0	0
68136	Polyarteritis nodosa and allied conditions NOS	0	0	0	0	0	0	1	0	0	0	0	0	0	0	0	0	0	0
68277	Acrosclerosis	0	0	0	0	0	0	1	0	0	0	0	0	0	0	0	0	0	0
68403	Giant cell arteritis NOS	0	0	0	0	0	0	1	0	0	0	0	0	0	0	0	0	0	0
68729	Arthropathy in Behcet's syndrome	0	0	0	0	0	0	1	0	0	0	0	0	0	0	0	0	0	0
68965	[X]Other systemic diseases of connective tissue	0	0	0	0	0	0	1	0	0	0	0	0	0	0	0	0	0	0
70221	[X]Other specified rheumatoid arthritis	0	0	0	0	0	0	1	0	0	0	0	0	0	0	0	0	0	0
70658	Rheumatoid arthritis of talonavicular joint	0	0	0	0	0	0	1	0	0	0	0	0	0	0	0	0	0	0
71083	Juvenile arthritis in ulcerative colitis	0	0	0	0	0	0	1	0	0	0	0	0	0	0	0	0	0	0
71206	Arthropathy in Behcet's syndrome of unspecified site	0	0	0	0	0	0	1	0	0	0	0	0	0	0	0	0	0	0
71258	Polyneuropathy in collagen vascular disease NOS	0	0	0	0	0	0	1	0	0	0	0	0	0	0	0	0	0	0
71763	[X]Other forms of systemic sclerosis	0	0	0	0	0	0	1	0	0	0	0	0	0	0	0	0	0	0
71784	Rheumatoid arthritis of other tarsal joint	0	0	0	0	0	0	1	0	0	0	0	0	0	0	0	0	0	0
72595	Sarcoidosis of inferior turbinates	0	0	0	0	0	0	1	0	0	0	0	0	0	0	0	0	0	0
73284	[X]Sarcoidosis of other and combined sites	0	0	0	0	0	0	1	0	0	0	0	0	0	0	0	0	0	0
73485	Unspecified circumscribed scleroderma	0	0	0	0	0	0	1	0	0	0	0	0	0	0	0	0	0	0
73619	Rheumatoid arthritis of subtalar joint	0	0	0	0	0	0	1	0	0	0	0	0	0	0	0	0	0	0
83529	[X]Mixed connective tissue disease	0	0	0	0	0	0	1	0	0	0	0	0	0	0	0	0	0	0
91559	Dermatopolymyositis in neoplastic disease	0	0	0	0	0	0	1	0	0	0	0	0	0	0	0	0	0	0
92421	[X]Systemic disorders/connective tissue in other diseases CE	0	0	0	0	0	0	1	0	0	0	0	0	0	0	0	0	0	0

93037	Acute febrile mucocutaneous lymph node syndrome NOS	0	0	0	0	0	0	1	0	0	0	0	0	0	0	0	0	0	0
93424	Thrombotic microangiopathy NOS	0	0	0	0	0	0	1	0	0	0	0	0	0	0	0	0	0	0
93715	[X]Other seropositive rheumatoid arthritis	0	0	0	0	0	0	1	0	0	0	0	0	0	0	0	0	0	0
93927	Polymyositis ossificans	0	0	0	0	0	0	1	0	0	0	0	0	0	0	0	0	0	0
94854	[X]Other juvenile arthritis	0	0	0	0	0	0	1	0	0	0	0	0	0	0	0	0	0	0
94996	Lung disease with systemic sclerosis	0	0	0	0	0	0	1	0	0	0	0	0	0	0	0	0	0	0
95437	[X]Dermatopolymyositis	0	0	0	0	0	0	1	0	0	0	0	0	0	0	0	0	0	0
95441	Arthropathy associated with haematological disorders	0	0	0	0	0	0	1	0	0	0	0	0	0	0	0	0	0	0
99414	Rheumatoid arthritis of lesser MTP joint	0	0	0	0	0	0	1	0	0	0	0	0	0	0	0	0	0	0
100365	Arthropathy in Behcet's syndrome of the ankle and foot	0	0	0	0	0	0	1	0	0	0	0	0	0	0	0	0	0	0
100776	Rheumatoid arthritis of sacro-iliac joint	0	0	0	0	0	0	1	0	0	0	0	0	0	0	0	0	0	0
100914	Rheumatoid arthritis of acromioclavicular joint	0	0	0	0	0	0	1	0	0	0	0	0	0	0	0	0	0	0
101433	Cerebral lupus	0	0	0	0	0	0	1	0	0	0	0	0	0	0	0	0	0	0
102193	Acute febrile mucocutaneous lymph node syndrome	0	0	0	0	0	0	1	0	0	0	0	0	0	0	0	0	0	0
103657	[X]Other specified localized connective tissue disorders	0	0	0	0	0	0	1	0	0	0	0	0	0	0	0	0	0	0
103858	Arthropathy in neoplastic disease	0	0	0	0	0	0	1	0	0	0	0	0	0	0	0	0	0	0
104312	Microscopic polyangiitis	0	0	0	0	0	0	1	0	0	0	0	0	0	0	0	0	0	0
105976	Acute scleroderma renal crisis	0	0	0	0	0	0	1	0	0	0	0	0	0	0	0	0	0	0
106440	[X]Rheumatoid arthritis+involvement/other organs or systems	0	0	0	0	0	0	1	0	0	0	0	0	0	0	0	0	0	0
107112	Rheumatoid arthritis of IP joint of toe	0	0	0	0	0	0	1	0	0	0	0	0	0	0	0	0	0	0
107382	Renal involvement in scleroderma	0	0	0	0	0	0	1	0	0	0	0	0	0	0	0	0	0	0
107791	Rheumatoid arthritis of tibio-fibular joint	0	0	0	0	0	0	1	0	0	0	0	0	0	0	0	0	0	0
107963	Rheumatoid arthritis of sternoclavicular joint	0	0	0	0	0	0	1	0	0	0	0	0	0	0	0	0	0	0
108072	Myopathy due to disseminated lupus erythematosus	0	0	0	0	0	0	1	0	0	0	0	0	0	0	0	0	0	0

109840	Goodpasture's disease	0	0	0	0	0	0	1	0	0	0	0	0	0	0	0	0	0	0
102088	Delivery of rehabilitation for rheumatoid arthritis	0	0	0	0	0	0	1	0	0	0	0	0	0	0	0	0	0	0
105507	Rheumatoid arthritis annual review	0	0	0	0	0	0	1	0	0	0	0	0	0	0	0	0	0	0
352	Duodenal ulcer - (DU)	0	0	0	0	0	0	0	1	0	0	0	0	0	0	0	0	0	0
657	Unspecified duodenal ulcer with perforation	0	0	0	0	0	0	0	1	0	0	0	0	0	0	0	0	0	0
670	Peptic ulcer - (PU) site unspecified	0	0	0	0	0	0	0	1	0	0	0	0	0	0	0	0	0	0
1262	Gastric ulcer - (GU)	0	0	0	0	0	0	0	1	0	0	0	0	0	0	0	0	0	0
1295	Stomal ulcer	0	0	0	0	0	0	0	1	0	0	0	0	0	0	0	0	0	0
2489	Ulcer of oesophagus	0	0	0	0	0	0	0	1	0	0	0	0	0	0	0	0	0	0
2812	H/O: peptic ulcer	0	0	0	0	0	0	0	1	0	0	0	0	0	0	0	0	0	0
2814	Unspecified duodenal ulcer with haemorrhage	0	0	0	0	0	0	0	1	0	0	0	0	0	0	0	0	0	0
2877	Gastric erosions	0	0	0	0	0	0	0	1	0	0	0	0	0	0	0	0	0	0
3101	Pyloric ulcer	0	0	0	0	0	0	0	1	0	0	0	0	0	0	0	0	0	0
3462	Duodenal erosion	0	0	0	0	0	0	0	1	0	0	0	0	0	0	0	0	0	0
4316	Marginal ulcer	0	0	0	0	0	0	0	1	0	0	0	0	0	0	0	0	0	0
4643	Peptic ulcer symptoms	0	0	0	0	0	0	0	1	0	0	0	0	0	0	0	0	0	0
4741	Closure of perforated duodenal ulcer	0	0	0	0	0	0	0	1	0	0	0	0	0	0	0	0	0	0
5283	Ulcerative oesophagitis	0	0	0	0	0	0	0	1	0	0	0	0	0	0	0	0	0	0
5521	Acute peptic ulcer with perforation	0	0	0	0	0	0	0	1	0	0	0	0	0	0	0	0	0	0
5596	Barrett's ulcer of oesophagus	0	0	0	0	0	0	0	1	0	0	0	0	0	0	0	0	0	0
5928	Operations on duodenal ulcer	0	0	0	0	0	0	0	1	0	0	0	0	0	0	0	0	0	0
6321	H/O: duodenal ulcer	0	0	0	0	0	0	0	1	0	0	0	0	0	0	0	0	0	0
6333	Prepyloric ulcer	0	0	0	0	0	0	0	1	0	0	0	0	0	0	0	0	0	0
6865	Stomach ulcer operations	0	0	0	0	0	0	0	1	0	0	0	0	0	0	0	0	0	0
7108	H/O: gastric ulcer	0	0	0	0	0	0	0	1	0	0	0	0	0	0	0	0	0	0
9853	Chronic duodenal ulcer	0	0	0	0	0	0	0	1	0	0	0	0	0	0	0	0	0	0
9981	Jejunal ulcer	0	0	0	0	0	0	0	1	0	0	0	0	0	0	0	0	0	0
10060	Oesophageal erosions	0	0	0	0	0	0	0	1	0	0	0	0	0	0	0	0	0	0
11104	Perforated chronic gastric ulcer	0	0	0	0	0	0	0	1	0	0	0	0	0	0	0	0	0	0

11124	Bleeding acute gastric ulcer	0	0	0	0	0	0	0	1	0	0	0	0	0	0	0	0	0	0
14671	Acute gastric ulcer with perforation	0	0	0	0	0	0	0	1	0	0	0	0	0	0	0	0	0	0
15175	Duodenal ulcer NOS	0	0	0	0	0	0	0	1	0	0	0	0	0	0	0	0	0	0
15403	Gastrojejunal ulcer NOS	0	0	0	0	0	0	0	1	0	0	0	0	0	0	0	0	0	0
15821	Stress ulcer NOS	0	0	0	0	0	0	0	1	0	0	0	0	0	0	0	0	0	0
15979	Unspecified gastrojejunal ulcer with perforation	0	0	0	0	0	0	0	1	0	0	0	0	0	0	0	0	0	0
18001	Acute duodenal ulcer with haemorrhage	0	0	0	0	0	0	0	1	0	0	0	0	0	0	0	0	0	0
18027	Acute duodenal ulcer	0	0	0	0	0	0	0	1	0	0	0	0	0	0	0	0	0	0
18319	Healed gastric ulcer leaving a scar	0	0	0	0	0	0	0	1	0	0	0	0	0	0	0	0	0	0
18324	Acute duodenal ulcer with perforation	0	0	0	0	0	0	0	1	0	0	0	0	0	0	0	0	0	0
18625	Bleeding chronic duodenal ulcer	0	0	0	0	0	0	0	1	0	0	0	0	0	0	0	0	0	0
18654	Chronic gastric ulcer	0	0	0	0	0	0	0	1	0	0	0	0	0	0	0	0	0	0
19928	Peptic ulcer NOS	0	0	0	0	0	0	0	1	0	0	0	0	0	0	0	0	0	0
20677	Closure of perforated gastric ulcer	0	0	0	0	0	0	0	1	0	0	0	0	0	0	0	0	0	0
22918	Duodenal ulcer disease	0	0	0	0	0	0	0	1	0	0	0	0	0	0	0	0	0	0
23082	Gastrojejunal ulcer (GJU)	0	0	0	0	0	0	0	1	0	0	0	0	0	0	0	0	0	0
23087	Perforated chronic duodenal ulcer	0	0	0	0	0	0	0	1	0	0	0	0	0	0	0	0	0	0
23487	Anastomotic ulcer	0	0	0	0	0	0	0	1	0	0	0	0	0	0	0	0	0	0
23688	Operations on gastric ulcer	0	0	0	0	0	0	0	1	0	0	0	0	0	0	0	0	0	0
24021	Peptic ulcer of oesophagus	0	0	0	0	0	0	0	1	0	0	0	0	0	0	0	0	0	0
24040	Acute gastric ulcer	0	0	0	0	0	0	0	1	0	0	0	0	0	0	0	0	0	0
24342	Acute gastric ulcer unspecified	0	0	0	0	0	0	0	1	0	0	0	0	0	0	0	0	0	0
26261	[V]Personal history of peptic ulcer	0	0	0	0	0	0	0	1	0	0	0	0	0	0	0	0	0	0
28366	Unspec duodenal ulcer	0	0	0	0	0	0	0	1	0	0	0	0	0	0	0	0	0	0

29317	Recurrent duodenal ulcer	0	0	0	0	0	0	0	1	0	0	0	0	0	0	0	0	0	0
29771	Gastric ulcer NOS	0	0	0	0	0	0	0	1	0	0	0	0	0	0	0	0	0	0
30054	Acute gastric ulcer with haemorrhage	0	0	0	0	0	0	0	1	0	0	0	0	0	0	0	0	0	0
32856	Acute peptic ulcer	0	0	0	0	0	0	0	1	0	0	0	0	0	0	0	0	0	0
33438	Chronic duodenal ulcer without mention of complication	0	0	0	0	0	0	0	1	0	0	0	0	0	0	0	0	0	0
33914	Operation on gastric ulcer NOS	0	0	0	0	0	0	0	1	0	0	0	0	0	0	0	0	0	0
36461	Unspecified gastric ulcer with perforation	0	0	0	0	0	0	0	1	0	0	0	0	0	0	0	0	0	0
36583	Bleeding chronic gastric ulcer	0	0	0	0	0	0	0	1	0	0	0	0	0	0	0	0	0	0
37268	Suture of duodenal ulcer not elsewhere classified	0	0	0	0	0	0	0	1	0	0	0	0	0	0	0	0	0	0
37620	Chronic peptic ulcer with perforation	0	0	0	0	0	0	0	1	0	0	0	0	0	0	0	0	0	0
37643	Chronic duodenal ulcer with perforation	0	0	0	0	0	0	0	1	0	0	0	0	0	0	0	0	0	0
40489	[V]Personal history of duodenal ulcer	0	0	0	0	0	0	0	1	0	0	0	0	0	0	0	0	0	0
40997	Chronic peptic ulcer	0	0	0	0	0	0	0	1	0	0	0	0	0	0	0	0	0	0
41271	[V] Personal history of gastric ulcer	0	0	0	0	0	0	0	1	0	0	0	0	0	0	0	0	0	0
42150	Ulcer of oesophagus NOS	0	0	0	0	0	0	0	1	0	0	0	0	0	0	0	0	0	0
42274	Acute gastrojejunal ulcer NOS	0	0	0	0	0	0	0	1	0	0	0	0	0	0	0	0	0	0
44073	Chronic duodenal ulcer with obstruction	0	0	0	0	0	0	0	1	0	0	0	0	0	0	0	0	0	0
44284	Unspecified gastric ulcer NOS	0	0	0	0	0	0	0	1	0	0	0	0	0	0	0	0	0	0
44309	Chronic gastric ulcer NOS	0	0	0	0	0	0	0	1	0	0	0	0	0	0	0	0	0	0
44324	Acute gastric ulcer NOS	0	0	0	0	0	0	0	1	0	0	0	0	0	0	0	0	0	0
44335	Acute duodenal ulcer without mention of complication	0	0	0	0	0	0	0	1	0	0	0	0	0	0	0	0	0	0
44637	Acute peptic ulcer with haemorrhage	0	0	0	0	0	0	0	1	0	0	0	0	0	0	0	0	0	0
45184	Acute gastrojejunal ulcer	0	0	0	0	0	0	0	1	0	0	0	0	0	0	0	0	0	0
45304	Acute peptic ulcer with haemorrhage and perforation	0	0	0	0	0	0	0	1	0	0	0	0	0	0	0	0	0	0

45981	Endoscopic injection haemostasis of duodenal ulcer	0	0	0	0	0	0	0	1	0	0	0	0	0	0	0	0	0	0
48730	Acute duodenal ulcer with haemorrhage and perforation	0	0	0	0	0	0	0	1	0	0	0	0	0	0	0	0	0	0
48946	Chronic gastric ulcer without mention of complication	0	0	0	0	0	0	0	1	0	0	0	0	0	0	0	0	0	0
48951	Chronic duodenal ulcer with haemorrhage	0	0	0	0	0	0	0	1	0	0	0	0	0	0	0	0	0	0
49926	Fungal ulcer of oesophagus	0	0	0	0	0	0	0	1	0	0	0	0	0	0	0	0	0	0
50048	Acute peptic ulcer NOS	0	0	0	0	0	0	0	1	0	0	0	0	0	0	0	0	0	0
50497	Unspecified peptic ulcer	0	0	0	0	0	0	0	1	0	0	0	0	0	0	0	0	0	0
51013	Oesophageal ulcer due to aspirin	0	0	0	0	0	0	0	1	0	0	0	0	0	0	0	0	0	0
51406	Chronic duodenal ulcer NOS	0	0	0	0	0	0	0	1	0	0	0	0	0	0	0	0	0	0
52138	Chronic duodenal ulcer unspecified	0	0	0	0	0	0	0	1	0	0	0	0	0	0	0	0	0	0
52313	Chronic peptic ulcer with obstruction	0	0	0	0	0	0	0	1	0	0	0	0	0	0	0	0	0	0
52323	Multiple gastric ulcers	0	0	0	0	0	0	0	1	0	0	0	0	0	0	0	0	0	0
53081	Unspecified gastric ulcer	0	0	0	0	0	0	0	1	0	0	0	0	0	0	0	0	0	0
53126	Chronic peptic ulcer with haemorrhage	0	0	0	0	0	0	0	1	0	0	0	0	0	0	0	0	0	0
53336	Chronic gastric ulcer with perforation	0	0	0	0	0	0	0	1	0	0	0	0	0	0	0	0	0	0
53669	Unspecified duodenal ulcer	0	0	0	0	0	0	0	1	0	0	0	0	0	0	0	0	0	0
53797	Acute duodenal ulcer NOS	0	0	0	0	0	0	0	1	0	0	0	0	0	0	0	0	0	0
53822	Acute duodenal ulcer unspecified	0	0	0	0	0	0	0	1	0	0	0	0	0	0	0	0	0	0
55350	Oversew of blood vessel of duodenal ulcer	0	0	0	0	0	0	0	1	0	0	0	0	0	0	0	0	0	0
55933	Operation on duodenal ulcer NOS	0	0	0	0	0	0	0	1	0	0	0	0	0	0	0	0	0	0
56005	Other specified operation on duodenal ulcer	0	0	0	0	0	0	0	1	0	0	0	0	0	0	0	0	0	0
57958	Unspecified gastric ulcer with haemorrhage	0	0	0	0	0	0	0	1	0	0	0	0	0	0	0	0	0	0
58603	Oesophageal ulcer due to medicines	0	0	0	0	0	0	0	1	0	0	0	0	0	0	0	0	0	0
60249	Unspecified peptic ulcer NOS	0	0	0	0	0	0	0	1	0	0	0	0	0	0	0	0	0	0

60346	Unspecified gastrojejunal ulcer with haemorrhage	0	0	0	0	0	0	0	1	0	0	0	0	0	0	0	0	0	0
63001	Anti-platelet induced gastric ulcer	0	0	0	0	0	0	0	1	0	0	0	0	0	0	0	0	0	0
63482	Unspecified gastrojejunal ulcer	0	0	0	0	0	0	0	1	0	0	0	0	0	0	0	0	0	0
63582	Chronic gastric ulcer with haemorrhage	0	0	0	0	0	0	0	1	0	0	0	0	0	0	0	0	0	0
63718	Endoscopic injection haemostasis of gastric ulcer	0	0	0	0	0	0	0	1	0	0	0	0	0	0	0	0	0	0
64014	Closure of gastric ulcer NEC	0	0	0	0	0	0	0	1	0	0	0	0	0	0	0	0	0	0
64111	Unspecified peptic ulcer with perforation	0	0	0	0	0	0	0	1	0	0	0	0	0	0	0	0	0	0
64165	Acute gastric ulcer without mention of complication	0	0	0	0	0	0	0	1	0	0	0	0	0	0	0	0	0	0
64556	Chronic gastric ulcer unspecified	0	0	0	0	0	0	0	1	0	0	0	0	0	0	0	0	0	0
64710	Resection of gastric ulcer by cautery	0	0	0	0	0	0	0	1	0	0	0	0	0	0	0	0	0	0
64913	Gastrocolic ulcer	0	0	0	0	0	0	0	1	0	0	0	0	0	0	0	0	0	0
65737	Unspecified duodenal ulcer NOS	0	0	0	0	0	0	0	1	0	0	0	0	0	0	0	0	0	0
66092	Unspecified gastrojejunal ulcer NOS	0	0	0	0	0	0	0	1	0	0	0	0	0	0	0	0	0	0
67082	Unspecified peptic ulcer without mention of complication	0	0	0	0	0	0	0	1	0	0	0	0	0	0	0	0	0	0
67356	Chronic gastric ulcer with obstruction	0	0	0	0	0	0	0	1	0	0	0	0	0	0	0	0	0	0
67711	Acute peptic ulcer unspecified	0	0	0	0	0	0	0	1	0	0	0	0	0	0	0	0	0	0
68661	Acute peptic ulcer without mention of complication	0	0	0	0	0	0	0	1	0	0	0	0	0	0	0	0	0	0
69663	Chronic peptic ulcer NOS	0	0	0	0	0	0	0	1	0	0	0	0	0	0	0	0	0	0
70005	Suture of ulcer of stomach NEC	0	0	0	0	0	0	0	1	0	0	0	0	0	0	0	0	0	0
70390	Chronic peptic ulcer unspecified	0	0	0	0	0	0	0	1	0	0	0	0	0	0	0	0	0	0
70456	Unspecified peptic ulcer with haemorrhage	0	0	0	0	0	0	0	1	0	0	0	0	0	0	0	0	0	0
71150	Unspecified duodenal ulcer without mention of complication	0	0	0	0	0	0	0	1	0	0	0	0	0	0	0	0	0	0
71403	Acute gastric ulcer with haemorrhage and perforation	0	0	0	0	0	0	0	1	0	0	0	0	0	0	0	0	0	0
71881	Chronic duodenal ulcer with haemorrhage and perforation	0	0	0	0	0	0	0	1	0	0	0	0	0	0	0	0	0	0

71897	Chronic gastric ulcer with haemorrhage and perforation	0	0	0	0	0	0	0	1	0	0	0	0	0	0	0	0	0	0
71904	Unspecified duodenal ulcer with obstruction	0	0	0	0	0	0	0	1	0	0	0	0	0	0	0	0	0	0
73338	Unspecified gastric ulcer without mention of complication	0	0	0	0	0	0	0	1	0	0	0	0	0	0	0	0	0	0
73417	Acute duodenal ulcer with obstruction	0	0	0	0	0	0	0	1	0	0	0	0	0	0	0	0	0	0
73697	Unspecified gastric ulcer with obstruction	0	0	0	0	0	0	0	1	0	0	0	0	0	0	0	0	0	0
85989	Non steroidal anti inflammatory drug induced duodenal ulcer	0	0	0	0	0	0	0	1	0	0	0	0	0	0	0	0	0	0
89023	Non steroidal anti inflammatory drug induced gastric ulcer	0	0	0	0	0	0	0	1	0	0	0	0	0	0	0	0	0	0
89227	Anti-platelet induced gastric ulcer NOS	0	0	0	0	0	0	0	1	0	0	0	0	0	0	0	0	0	0
89234	Anti-platelet induced duodenal ulcer	0	0	0	0	0	0	0	1	0	0	0	0	0	0	0	0	0	0
92695	Balfour excision of gastric ulcer	0	0	0	0	0	0	0	1	0	0	0	0	0	0	0	0	0	0
93436	Unspecified duodenal ulcer with haemorrhage and perforation	0	0	0	0	0	0	0	1	0	0	0	0	0	0	0	0	0	0
94104	Other specified operation on gastric ulcer	0	0	0	0	0	0	0	1	0	0	0	0	0	0	0	0	0	0
94397	Unspec gastric ulcer	0	0	0	0	0	0	0	1	0	0	0	0	0	0	0	0	0	0
94752	Oesophageal ulcer due to chemicals	0	0	0	0	0	0	0	1	0	0	0	0	0	0	0	0	0	0
96090	Chronic gastrojejunal ulcer	0	0	0	0	0	0	0	1	0	0	0	0	0	0	0	0	0	0
96622	Unspecified peptic ulcer with haemorrhage and perforation	0	0	0	0	0	0	0	1	0	0	0	0	0	0	0	0	0	0
96628	Acute gastrojejunal ulcer with haemorrhage	0	0	0	0	0	0	0	1	0	0	0	0	0	0	0	0	0	0
99430	Chronic peptic ulcer without mention of complication	0	0	0	0	0	0	0	1	0	0	0	0	0	0	0	0	0	0
99670	Unspecified peptic ulcer with obstruction	0	0	0	0	0	0	0	1	0	0	0	0	0	0	0	0	0	0
102177	Acute gastrojejunal ulcer with perforation	0	0	0	0	0	0	0	1	0	0	0	0	0	0	0	0	0	0
106330	Acute gastrojejunal ulcer with haemorrhage and perforation	0	0	0	0	0	0	0	1	0	0	0	0	0	0	0	0	0	0
109546	Acute gastric ulcer with obstruction	0	0	0	0	0	0	0	1	0	0	0	0	0	0	0	0	0	0
1638	Cirrhosis of liver NOS	0	0	0	0	0	0	0	0	1	0	0	0	0	0	0	0	0	0
1754	Chronic hepatitis	0	0	0	0	0	0	0	0	1	0	0	0	0	0	0	0	0	0

1755	Chronic aggressive hepatitis	0	0	0	0	0	0	0	0	1	0	0	0	0	0	0	0	0	0
3216	Acute alcoholic hepatitis	0	0	0	0	0	0	0	0	1	0	0	0	0	0	0	0	0	0
3450	Diffuse nodular cirrhosis	0	0	0	0	0	0	0	0	1	0	0	0	0	0	0	0	0	0
4743	Alcoholic cirrhosis of liver	0	0	0	0	0	0	0	0	1	0	0	0	0	0	0	0	0	0
5638	Primary biliary cirrhosis	0	0	0	0	0	0	0	0	1	0	0	0	0	0	0	0	0	0
6015	[X]Other and unspecified cirrhosis of liver	0	0	0	0	0	0	0	0	1	0	0	0	0	0	0	0	0	0
6863	Cirrhosis and chronic liver disease	0	0	0	0	0	0	0	0	1	0	0	0	0	0	0	0	0	0
7602	Chronic alcoholic hepatitis	0	0	0	0	0	0	0	0	1	0	0	0	0	0	0	0	0	0
7885	Alcoholic liver damage unspecified	0	0	0	0	0	0	0	0	1	0	0	0	0	0	0	0	0	0
7943	Alcoholic hepatitis	0	0	0	0	0	0	0	0	1	0	0	0	0	0	0	0	0	0
8206	Pigmentary cirrhosis of liver	0	0	0	0	0	0	0	0	1	0	0	0	0	0	0	0	0	0
9029	Chronic active hepatitis	0	0	0	0	0	0	0	0	1	0	0	0	0	0	0	0	0	0
9494	Biliary cirrhosis	0	0	0	0	0	0	0	0	1	0	0	0	0	0	0	0	0	0
10234	Non-alcoholic fatty liver	0	0	0	0	0	0	0	0	1	0	0	0	0	0	0	0	0	0
10539	Chronic liver disease NOS	0	0	0	0	0	0	0	0	1	0	0	0	0	0	0	0	0	0
10691	Alcoholic fatty liver	0	0	0	0	0	0	0	0	1	0	0	0	0	0	0	0	0	0
15424	Secondary biliary cirrhosis	0	0	0	0	0	0	0	0	1	0	0	0	0	0	0	0	0	0
15489	Chronic hepatitis NOS	0	0	0	0	0	0	0	0	1	0	0	0	0	0	0	0	0	0
16455	Non-alcoholic cirrhosis NOS	0	0	0	0	0	0	0	0	1	0	0	0	0	0	0	0	0	0
16725	Cirrhosis - non alcoholic	0	0	0	0	0	0	0	0	1	0	0	0	0	0	0	0	0	0
17219	Toxic liver disease with chronic persistent hepatitis	0	0	0	0	0	0	0	0	1	0	0	0	0	0	0	0	0	0
18739	Cryptogenic cirrhosis of liver	0	0	0	0	0	0	0	0	1	0	0	0	0	0	0	0	0	0
21713	Alcoholic fibrosis and sclerosis of liver	0	0	0	0	0	0	0	0	1	0	0	0	0	0	0	0	0	0
22766	Toxic liver disease with cholestasis	0	0	0	0	0	0	0	0	1	0	0	0	0	0	0	0	0	0
22841	Macronodular cirrhosis of liver	0	0	0	0	0	0	0	0	1	0	0	0	0	0	0	0	0	0
23578	Chronic persistent hepatitis	0	0	0	0	0	0	0	0	1	0	0	0	0	0	0	0	0	0

24813	Chronic viral hepatitis B with delta-agent	0	0	0	0	0	0	0	0	1	0	0	0	0	0	0	0	0	0
25383	Hepatic fibrosis	0	0	0	0	0	0	0	0	1	0	0	0	0	0	0	0	0	0
26367	Chronic viral hepatitis	0	0	0	0	0	0	0	0	1	0	0	0	0	0	0	0	0	0
27438	Cardiac portal cirrhosis	0	0	0	0	0	0	0	0	1	0	0	0	0	0	0	0	0	0
30586	Chronic viral hepatitis C	0	0	0	0	0	0	0	0	1	0	0	0	0	0	0	0	0	0
31897	Liver abscess and sequelae of chronic liver disease	0	0	0	0	0	0	0	0	1	0	0	0	0	0	0	0	0	0
32277	Chronic viral hepatitis, unspecified	0	0	0	0	0	0	0	0	1	0	0	0	0	0	0	0	0	0
33597	Other non-alcoholic chronic liver disease NOS	0	0	0	0	0	0	0	0	1	0	0	0	0	0	0	0	0	0
34642	Congenital hepatic fibrosis	0	0	0	0	0	0	0	0	1	0	0	0	0	0	0	0	0	0
36107	Toxic liver disease with hepatic necrosis	0	0	0	0	0	0	0	0	1	0	0	0	0	0	0	0	0	0
36727	Toxic liver disease	0	0	0	0	0	0	0	0	1	0	0	0	0	0	0	0	0	0
39351	Toxic liver disease with chronic active hepatitis	0	0	0	0	0	0	0	0	1	0	0	0	0	0	0	0	0	0
40567	Capsular portal cirrhosis	0	0	0	0	0	0	0	0	1	0	0	0	0	0	0	0	0	0
40963	Portal fibrosis without cirrhosis	0	0	0	0	0	0	0	0	1	0	0	0	0	0	0	0	0	0
41096	Chronic viral hepatitis B without delta-agent	0	0	0	0	0	0	0	0	1	0	0	0	0	0	0	0	0	0
41104	Toxic liver disease with acute hepatitis	0	0	0	0	0	0	0	0	1	0	0	0	0	0	0	0	0	0
41673	Toxic liver disease, unspecified	0	0	0	0	0	0	0	0	1	0	0	0	0	0	0	0	0	0
42843	Other non-alcoholic chronic liver disease	0	0	0	0	0	0	0	0	1	0	0	0	0	0	0	0	0	0
44120	Toxic liver disease with fibrosis and cirrhosis of liver	0	0	0	0	0	0	0	0	1	0	0	0	0	0	0	0	0	0
44676	Fatty portal cirrhosis	0	0	0	0	0	0	0	0	1	0	0	0	0	0	0	0	0	0
46723	Atrophy of left lobe of liver	0	0	0	0	0	0	0	0	1	0	0	0	0	0	0	0	0	0
47257	Portal cirrhosis	0	0	0	0	0	0	0	0	1	0	0	0	0	0	0	0	0	0
48102	Other sequelae of chronic liver disease	0	0	0	0	0	0	0	0	1	0	0	0	0	0	0	0	0	0
48928	Infectious cirrhosis NOS	0	0	0	0	0	0	0	0	1	0	0	0	0	0	0	0	0	0

53877	Chronic hepatitis unspecified	0	0	0	0	0	0	0	0	1	0	0	0	0	0	0	0	0	0
55454	Portal cirrhosis unspecified	0	0	0	0	0	0	0	0	1	0	0	0	0	0	0	0	0	0
58184	Indian childhood cirrhosis	0	0	0	0	0	0	0	0	1	0	0	0	0	0	0	0	0	0
58630	Biliary cirrhosis NOS	0	0	0	0	0	0	0	0	1	0	0	0	0	0	0	0	0	0
60104	Hepatic sclerosis	0	0	0	0	0	0	0	0	1	0	0	0	0	0	0	0	0	0
64750	Toxic liver disease with chronic lobular hepatitis	0	0	0	0	0	0	0	0	1	0	0	0	0	0	0	0	0	0
66534	Chronic lobular hepatitis	0	0	0	0	0	0	0	0	1	0	0	0	0	0	0	0	0	0
68376	Florid cirrhosis	0	0	0	0	0	0	0	0	1	0	0	0	0	0	0	0	0	0
69204	Multilobular portal cirrhosis	0	0	0	0	0	0	0	0	1	0	0	0	0	0	0	0	0	0
71453	Hepatic fibrosis	0	0	0	0	0	0	0	0	1	0	0	0	0	0	0	0	0	0
73482	Bacterial portal cirrhosis	0	0	0	0	0	0	0	0	1	0	0	0	0	0	0	0	0	0
91591	Biliary cirrhosis of children	0	0	0	0	0	0	0	0	1	0	0	0	0	0	0	0	0	0
92909	Hypertrophic portal cirrhosis	0	0	0	0	0	0	0	0	1	0	0	0	0	0	0	0	0	0
96664	Juvenile portal cirrhosis	0	0	0	0	0	0	0	0	1	0	0	0	0	0	0	0	0	0
99898	Chronic hepatitis annual review - enhanced services admin	0	0	0	0	0	0	0	0	1	0	0	0	0	0	0	0	0	0
100253	Xanthomatous portal cirrhosis	0	0	0	0	0	0	0	0	1	0	0	0	0	0	0	0	0	0
100474	Laennec's cirrhosis	0	0	0	0	0	0	0	0	1	0	0	0	0	0	0	0	0	0
100592	Hepatic fibrosis with hepatic sclerosis	0	0	0	0	0	0	0	0	1	0	0	0	0	0	0	0	0	0
102922	Cystic fibrosis related cirrhosis	0	0	0	0	0	0	0	0	1	0	0	0	0	0	0	0	0	0
103466	Fatty change of liver	0	0	0	0	0	0	0	0	1	0	0	0	0	0	0	0	0	0
103706	Fatty liver	0	0	0	0	0	0	0	0	1	0	0	0	0	0	0	0	0	0
106025	[X]Toxic liver disease, unspecified	0	0	0	0	0	0	0	0	1	0	0	0	0	0	0	0	0	0
106642	Chronic viral hepatitis B	0	0	0	0	0	0	0	0	1	0	0	0	0	0	0	0	0	0
107896	[X]Chronic viral hepatitis, unspecified	0	0	0	0	0	0	0	0	1	0	0	0	0	0	0	0	0	0

108343	[X]Other chronic viral hepatitis	0	0	0	0	0	0	0	0	1	0	0	0	0	0	0	0	0	0
108800	Liver abscess and chronic liver disease causing sequelae NOS	0	0	0	0	0	0	0	0	1	0	0	0	0	0	0	0	0	0
108819	Congestive cirrhosis	0	0	0	0	0	0	0	0	1	0	0	0	0	0	0	0	0	0
109540	Zooparasitic portal cirrhosis	0	0	0	0	0	0	0	0	1	0	0	0	0	0	0	0	0	0
3621	O/E – diplegia	0	0	0	0	0	0	0	0	0	2	0	0	0	0	0	0	0	0
102734	O/E - paraplegic in extension	0	0	0	0	0	0	0	0	0	2	0	0	0	0	0	0	0	0
8282	O/E - quadriplegia	0	0	0	0	0	0	0	0	0	2	0	0	0	0	0	0	0	0
1749	Hemiplegia	0	0	0	0	0	0	0	0	0	2	0	0	0	0	0	0	0	0
46128	Flaccid tetraplegia	0	0	0	0	0	0	0	0	0	2	0	0	0	0	0	0	0	0
55371	O/E - paraplegic in flexion	0	0	0	0	0	0	0	0	0	2	0	0	0	0	0	0	0	0
8933	Left hemiplegia	0	0	0	0	0	0	0	0	0	2	0	0	0	0	0	0	0	0
46175	Flaccid paraplegia	0	0	0	0	0	0	0	0	0	2	0	0	0	0	0	0	0	0
16117	Tetraplegia	0	0	0	0	0	0	0	0	0	2	0	0	0	0	0	0	0	0
8492	Hemiplegia NOS	0	0	0	0	0	0	0	0	0	2	0	0	0	0	0	0	0	0
3293	Right hemiplegia	0	0	0	0	0	0	0	0	0	2	0	0	0	0	0	0	0	0
36133	O/E - paraplegia	0	0	0	0	0	0	0	0	0	2	0	0	0	0	0	0	0	0
39085	Flaccid hemiplegia	0	0	0	0	0	0	0	0	0	2	0	0	0	0	0	0	0	0
40407	O/E - hemiplegic posture	0	0	0	0	0	0	0	0	0	2	0	0	0	0	0	0	0	0
22135	O/E - hemiplegia	0	0	0	0	0	0	0	0	0	2	0	0	0	0	0	0	0	0
3063	Paraplegia	0	0	0	0	0	0	0	0	0	2	0	0	0	0	0	0	0	0
9271	Quadriplegia	0	0	0	0	0	0	0	0	0	2	0	0	0	0	0	0	0	0
59494	Massive muscular calcification associated with paraplegia	0	0	0	0	0	0	0	0	0	2	0	0	0	0	0	0	0	0
512	Chronic renal failure	0	0	0	0	0	0	0	0	0	0	2	0	0	0	0	0	0	0
6712	End stage renal failure	0	0	0	0	0	0	0	0	0	0	2	0	0	0	0	0	0	0
8330	End-stage renal disease	0	0	0	0	0	0	0	0	0	0	2	0	0	0	0	0	0	0
12479	Chronic kidney disease stage 4	0	0	0	0	0	0	0	0	0	0	2	0	0	0	0	0	0	0

12566	Chronic kidney disease stage 3	0	0	0	0	0	0	0	0	0	0	2	0	0	0	0	0	0	0
12585	Chronic kidney disease stage 5	0	0	0	0	0	0	0	0	0	0	2	0	0	0	0	0	0	0
12720	Chronic renal impairment	0	0	0	0	0	0	0	0	0	0	2	0	0	0	0	0	0	0
53852	End stage renal failure	0	0	0	0	0	0	0	0	0	0	2	0	0	0	0	0	0	0
89332	predicted stage chronic kidney disease	0	0	0	0	0	0	0	0	0	0	2	0	0	0	0	0	0	0
94793	Chronic kidney disease stage 3 with proteinuria	0	0	0	0	0	0	0	0	0	0	2	0	0	0	0	0	0	0
94965	Chronic kidney disease stage 3A	0	0	0	0	0	0	0	0	0	0	2	0	0	0	0	0	0	0
95121	Chronic kidney disease stage 2 without proteinuria	0	0	0	0	0	0	0	0	0	0	2	0	0	0	0	0	0	0
95122	Chronic kidney disease stage 4 with proteinuria	0	0	0	0	0	0	0	0	0	0	2	0	0	0	0	0	0	0
95123	Chronic kidney disease stage 3 without proteinuria	0	0	0	0	0	0	0	0	0	0	2	0	0	0	0	0	0	0
95145	CKD stage 3 with proteinuria	0	0	0	0	0	0	0	0	0	0	2	0	0	0	0	0	0	0
95175	Chronic kidney disease stage 3A without proteinuria	0	0	0	0	0	0	0	0	0	0	2	0	0	0	0	0	0	0
95176	CKD stage 3A without proteinuria	0	0	0	0	0	0	0	0	0	0	2	0	0	0	0	0	0	0
95177	Chronic kidney disease stage 3B without proteinuria	0	0	0	0	0	0	0	0	0	0	2	0	0	0	0	0	0	0
95178	Chronic kidney disease stage 3B with proteinuria	0	0	0	0	0	0	0	0	0	0	2	0	0	0	0	0	0	0
95179	Chronic kidney disease stage 3B	0	0	0	0	0	0	0	0	0	0	2	0	0	0	0	0	0	0
95180	CKD stage 3B with proteinuria	0	0	0	0	0	0	0	0	0	0	2	0	0	0	0	0	0	0
95188	CKD stage 3 without proteinuria	0	0	0	0	0	0	0	0	0	0	2	0	0	0	0	0	0	0
95405	Chronic kidney disease stage 5 without proteinuria	0	0	0	0	0	0	0	0	0	0	2	0	0	0	0	0	0	0
95406	Chronic kidney disease stage 4 without proteinuria	0	0	0	0	0	0	0	0	0	0	2	0	0	0	0	0	0	0
95408	Chronic kidney disease stage 3A with proteinuria	0	0	0	0	0	0	0	0	0	0	2	0	0	0	0	0	0	0
95508	Chronic kidney disease stage 5 with proteinuria	0	0	0	0	0	0	0	0	0	0	2	0	0	0	0	0	0	0
95571	CKD stage 3A with proteinuria	0	0	0	0	0	0	0	0	0	0	2	0	0	0	0	0	0	0
97587	CKD stage 4 without proteinuria	0	0	0	0	0	0	0	0	0	0	2	0	0	0	0	0	0	0

97683	CKD stage 5 without proteinuria	0	0	0	0	0	0	0	0	0	0	2	0	0	0	0	0	0	0
99160	CKD stage 5 with proteinuria	0	0	0	0	0	0	0	0	0	0	2	0	0	0	0	0	0	0
99312	CKD stage 4 with proteinuria	0	0	0	0	0	0	0	0	0	0	2	0	0	0	0	0	0	0
100633	CKD stage 3B without proteinuria	0	0	0	0	0	0	0	0	0	0	2	0	0	0	0	0	0	0
105151	chronic kidney disease stage 5	0	0	0	0	0	0	0	0	0	0	2	0	0	0	0	0	0	0
2994	Peritoneal dialysis	0	0	0	0	0	0	0	0	0	0	2	0	0	0	0	0	0	0
2995	Acquired arteriovenous fistula	0	0	0	0	0	0	0	0	0	0	2	0	0	0	0	0	0	0
2996	Haemodialysis NEC	0	0	0	0	0	0	0	0	0	0	2	0	0	0	0	0	0	0
3205	Creation of arteriovenous fistula NEC	0	0	0	0	0	0	0	0	0	0	2	0	0	0	0	0	0	0
8037	Insertion of ambulatory peritoneal dialysis catheter	0	0	0	0	0	0	0	0	0	0	2	0	0	0	0	0	0	0
9765	Ligation of acquired arteriovenous fistula	0	0	0	0	0	0	0	0	0	0	2	0	0	0	0	0	0	0
11773	Dialysis for renal failure	0	0	0	0	0	0	0	0	0	0	2	0	0	0	0	0	0	0
18779	Repair of acquired arteriovenous fistula	0	0	0	0	0	0	0	0	0	0	2	0	0	0	0	0	0	0
20073	Renal dialysis	0	0	0	0	0	0	0	0	0	0	2	0	0	0	0	0	0	0
20196	H/O: renal dialysis	0	0	0	0	0	0	0	0	0	0	2	0	0	0	0	0	0	0
22252	[V]Renal dialysis status	0	0	0	0	0	0	0	0	0	0	2	0	0	0	0	0	0	0
23773	Removal of ambulatory peritoneal dialysis catheter	0	0	0	0	0	0	0	0	0	0	2	0	0	0	0	0	0	0
24151	Arteriovenous shunt	0	0	0	0	0	0	0	0	0	0	2	0	0	0	0	0	0	0
25521	Creation of brachial-cephalic fistula	0	0	0	0	0	0	0	0	0	0	2	0	0	0	0	0	0	0
28158	Kidney dialysis with complication without blame	0	0	0	0	0	0	0	0	0	0	2	0	0	0	0	0	0	0
28269	Creation of radial-cephalic fistula	0	0	0	0	0	0	0	0	0	0	2	0	0	0	0	0	0	0
30709	Insertion of temporary peritoneal dialysis catheter	0	0	0	0	0	0	0	0	0	0	2	0	0	0	0	0	0	0
30756	Continuous ambulatory peritoneal dialysis	0	0	0	0	0	0	0	0	0	0	2	0	0	0	0	0	0	0
31478	Removal of infected arteriovenous shunt	0	0	0	0	0	0	0	0	0	0	2	0	0	0	0	0	0	0
31549	Compensation for renal failure	0	0	0	0	0	0	0	0	0	0	2	0	0	0	0	0	0	0

35921	Failure of sterile precautions during perfusion	0	0	0	0	0	0	0	0	0	0	2	0	0	0	0	0	0	0
36442	Placement ambulatory dialysis apparatus - compens renal fail	0	0	0	0	0	0	0	0	0	0	2	0	0	0	0	0	0	0
44422	H/O: kidney dialysis	0	0	0	0	0	0	0	0	0	0	2	0	0	0	0	0	0	0
45160	[V]Aftercare involving peritoneal dialysis	0	0	0	0	0	0	0	0	0	0	2	0	0	0	0	0	0	0
46145	[V]Aftercare involving renal dialysis NOS	0	0	0	0	0	0	0	0	0	0	2	0	0	0	0	0	0	0
46438	[X] Peritoneal dialysis associated peritonitis	0	0	0	0	0	0	0	0	0	0	2	0	0	0	0	0	0	0
48022	Other specified compensation for renal failure	0	0	0	0	0	0	0	0	0	0	2	0	0	0	0	0	0	0
48639	Mechanical complication of dialysis catheter	0	0	0	0	0	0	0	0	0	0	2	0	0	0	0	0	0	0
48713	mechanical complication of arterio-venous surgical fistula	0	0	0	0	0	0	0	0	0	0	2	0	0	0	0	0	0	0
49028	h/o: kidney recipient	0	0	0	0	0	0	0	0	0	0	2	0	0	0	0	0	0	0
52088	[V]Preparatory care for dialysis	0	0	0	0	0	0	0	0	0	0	2	0	0	0	0	0	0	0
54844	[X]Failure sterile precautions dur kidney dialys/other perf	0	0	0	0	0	0	0	0	0	0	2	0	0	0	0	0	0	0
56760	Placement ambulatory apparatus compensation renal failure	0	0	0	0	0	0	0	0	0	0	2	0	0	0	0	0	0	0
58618	Arteriovenous shunt NOS	0	0	0	0	0	0	0	0	0	0	2	0	0	0	0	0	0	0
59194	Placement ambulatory apparatus-compensate renal failure OS	0	0	0	0	0	0	0	0	0	0	2	0	0	0	0	0	0	0
59315	Stenosis of arteriovenous dialysis fistula	0	0	0	0	0	0	0	0	0	0	2	0	0	0	0	0	0	0
60302	Creation of graft fistula for dialysis	0	0	0	0	0	0	0	0	0	0	2	0	0	0	0	0	0	0
60446	Care of haemodialysis equipment	0	0	0	0	0	0	0	0	0	0	2	0	0	0	0	0	0	0
60498	Reversing haemodialysis lines	0	0	0	0	0	0	0	0	0	0	2	0	0	0	0	0	0	0
60743	[V]Aftercare involving intermittent dialysis	0	0	0	0	0	0	0	0	0	0	2	0	0	0	0	0	0	0
63038	[V]Unspecified aftercare involving intermittent dialysis	0	0	0	0	0	0	0	0	0	0	2	0	0	0	0	0	0	0
63063	Insertion of arteriovenous prosthesis	0	0	0	0	0	0	0	0	0	0	2	0	0	0	0	0	0	0
63190	Attention to arteriovenous shunt	0	0	0	0	0	0	0	0	0	0	2	0	0	0	0	0	0	0
63305	Thrombectomy of arteriovenous fistula	0	0	0	0	0	0	0	0	0	0	2	0	0	0	0	0	0	0

63488	[V]Other specified aftercare involving intermittent dialysis	0	0	0	0	0	0	0	0	0	0	2	0	0	0	0	0	0	0
63502	Peritoneal dialysis bag procedure	0	0	0	0	0	0	0	0	0	0	2	0	0	0	0	0	0	0
64636	Compensation for renal failure NOS	0	0	0	0	0	0	0	0	0	0	2	0	0	0	0	0	0	0
64828	Peritoneal dialysis NEC	0	0	0	0	0	0	0	0	0	0	2	0	0	0	0	0	0	0
65089	Placement other apparatus- compensate for renal failure NOS	0	0	0	0	0	0	0	0	0	0	2	0	0	0	0	0	0	0
65398	Other specified arteriovenous shunt	0	0	0	0	0	0	0	0	0	0	2	0	0	0	0	0	0	0
66714	Renal dialysis with complication without blame	0	0	0	0	0	0	0	0	0	0	2	0	0	0	0	0	0	0
69266	Failure of sterile precautions during kidney dialysis	0	0	0	0	0	0	0	0	0	0	2	0	0	0	0	0	0	0
69427	Accid cut puncture perf h'ge - perfusion NOS	0	0	0	0	0	0	0	0	0	0	2	0	0	0	0	0	0	0
69760	[X]Other dialysis	0	0	0	0	0	0	0	0	0	0	2	0	0	0	0	0	0	0
71124	Haemofiltration	0	0	0	0	0	0	0	0	0	0	2	0	0	0	0	0	0	0
72336	Priming haemodialysis lines	0	0	0	0	0	0	0	0	0	0	2	0	0	0	0	0	0	0
74905	Haemodialysis training	0	0	0	0	0	0	0	0	0	0	2	0	0	0	0	0	0	0
83513	Placement other apparatus for compensation for renal failure	0	0	0	0	0	0	0	0	0	0	2	0	0	0	0	0	0	0
86419	dialysis fluid glucose level	0	0	0	0	0	0	0	0	0	0	2	0	0	0	0	0	0	0
88597	Automated peritoneal dialysis	0	0	0	0	0	0	0	0	0	0	2	0	0	0	0	0	0	0
96131	Banding of arteriovenous fistula	0	0	0	0	0	0	0	0	0	0	2	0	0	0	0	0	0	0
96184	Accid cut puncture perf h'ge - kidney dialysis	0	0	0	0	0	0	0	0	0	0	2	0	0	0	0	0	0	0
96347	Ligation of arteriovenous dialysis fistula	0	0	0	0	0	0	0	0	0	0	2	0	0	0	0	0	0	0
98888	peritoneal dialysis sample	0	0	0	0	0	0	0	0	0	0	2	0	0	0	0	0	0	0
99692	[v]aftercare involving extracorporeal dialysis	0	0	0	0	0	0	0	0	0	0	2	0	0	0	0	0	0	0
100235	Ligation of acquired arteriovenous fistula	0	0	0	0	0	0	0	0	0	0	2	0	0	0	0	0	0	0
101124	continuous ambulatory peritoneal dialysis associated perit	0	0	0	0	0	0	0	0	0	0	2	0	0	0	0	0	0	0
101736	dialysis fluid urea level	0	0	0	0	0	0	0	0	0	0	2	0	0	0	0	0	0	0

101756	Thomas intravascular shunt for dialysis	0	0	0	0	0	0	0	0	0	0	2	0	0	0	0	0	0	0
101912	dialysis training	0	0	0	0	0	0	0	0	0	0	2	0	0	0	0	0	0	0
104586	flushing of peritoneal dialysis catheter	0	0	0	0	0	0	0	0	0	0	2	0	0	0	0	0	0	0
104719	peritoneal dialysis training	0	0	0	0	0	0	0	0	0	0	2	0	0	0	0	0	0	0
105436	anaphylactoid reaction due to haemodialysis	0	0	0	0	0	0	0	0	0	0	2	0	0	0	0	0	0	0
105742	aneurysm of anastomotic site of dialysis av fistula	0	0	0	0	0	0	0	0	0	0	2	0	0	0	0	0	0	0
105760	ruptured aneurysm of dialysis vascular access	0	0	0	0	0	0	0	0	0	0	2	0	0	0	0	0	0	0
106720	thrombosis of dialysis arteriovenous fistula	0	0	0	0	0	0	0	0	0	0	2	0	0	0	0	0	0	0
106975	haemorrhage of dialysis arteriovenous fistula	0	0	0	0	0	0	0	0	0	0	2	0	0	0	0	0	0	0
107082	occlusion of dialysis arteriovenous fistula	0	0	0	0	0	0	0	0	0	0	2	0	0	0	0	0	0	0
107188	aneurysm of dialysis arteriovenous fistula	0	0	0	0	0	0	0	0	0	0	2	0	0	0	0	0	0	0
107217	arteriovenous fistula thrombosis	0	0	0	0	0	0	0	0	0	0	2	0	0	0	0	0	0	0
107220	aneurysm of needle site of dialysis arteriovenous fistula	0	0	0	0	0	0	0	0	0	0	2	0	0	0	0	0	0	0
107260	infection of dialysis arteriovenous fistula	0	0	0	0	0	0	0	0	0	0	2	0	0	0	0	0	0	0
107719	ligation of arteriovenous dialysis graft	0	0	0	0	0	0	0	0	0	0	2	0	0	0	0	0	0	0
107746	stenosis of dialysis vascular access	0	0	0	0	0	0	0	0	0	0	2	0	0	0	0	0	0	0
107900	disorders associated with peritoneal dialysis	0	0	0	0	0	0	0	0	0	0	2	0	0	0	0	0	0	0
108116	occlusion of dialysis vascular access	0	0	0	0	0	0	0	0	0	0	2	0	0	0	0	0	0	0
108213	infection of dialysis arteriovenous graft	0	0	0	0	0	0	0	0	0	0	2	0	0	0	0	0	0	0
108423	rupture of dialysis arteriovenous graft	0	0	0	0	0	0	0	0	0	0	2	0	0	0	0	0	0	0
16929	Anaemia secondary to renal failure	0	0	0	0	0	0	0	0	0	0	2	0	0	0	0	0	0	0
25394	Anaemia secondary to chronic renal failure	0	0	0	0	0	0	0	0	0	0	2	0	0	0	0	0	0	0
28684	Hypertensive heart and renal disease with renal failure	0	0	0	0	0	0	0	0	0	0	2	0	0	0	0	0	0	0
32423	Hypertensive renal disease with renal failure	0	0	0	0	0	0	0	0	0	0	2	0	0	0	0	0	0	0

53940	[X]Other chronic renal failure	0	0	0	0	0	0	0	0	0	0	2	0	0	0	0	0	0	0
107901	placement other apparatus- compensate for renal failure os	0	0	0	0	0	0	0	0	0	0	2	0	0	0	0	0	0	0
70712	det.ren.func.after ren.transpl	0	0	0	0	0	0	0	0	0	0	2	0	0	0	0	0	0	0
72004	Excision of rejected transplanted kidney	0	0	0	0	0	0	0	0	0	0	2	0	0	0	0	0	0	0
93366	Interventions associated with transplantation of kidney	0	0	0	0	0	0	0	0	0	0	2	0	0	0	0	0	0	0
103429	post-transplantation of kidney examination, recipient	0	0	0	0	0	0	0	0	0	0	2	0	0	0	0	0	0	0
103649	transplantation surgery	0	0	0	0	0	0	0	0	0	0	2	0	0	0	0	0	0	0
104049	interventions associated with transplantation of kidney nos	0	0	0	0	0	0	0	0	0	0	2	0	0	0	0	0	0	0
104050	os interventions associated with transplantation of kidney	0	0	0	0	0	0	0	0	0	0	2	0	0	0	0	0	0	0
105328	Cadaveric renal transplant	0	0	0	0	0	0	0	0	0	0	2	0	0	0	0	0	0	0
105724	unexplained episode of renal transplant dysfunction	0	0	0	0	0	0	0	0	0	0	2	0	0	0	0	0	0	0
105787	Xenograft renal transplant	0	0	0	0	0	0	0	0	0	0	2	0	0	0	0	0	0	0
106620	chronic rejection of renal transplant	0	0	0	0	0	0	0	0	0	0	2	0	0	0	0	0	0	0
106866	vascular complication of renal transplant	0	0	0	0	0	0	0	0	0	0	2	0	0	0	0	0	0	0
107183	discussion about kidney transplantation	0	0	0	0	0	0	0	0	0	0	2	0	0	0	0	0	0	0
107752	urological complication of renal transplant	0	0	0	0	0	0	0	0	0	0	2	0	0	0	0	0	0	0
109455	Allotransplantation of kidney from cadaver NEC	0	0	0	0	0	0	0	0	0	0	2	0	0	0	0	0	0	0
2997	Transplantation of kidney	0	0	0	0	0	0	0	0	0	0	2	0	0	0	0	0	0	0
5504	Transplantation of kidney NOS	0	0	0	0	0	0	0	0	0	0	2	0	0	0	0	0	0	0
5911	[V]Kidney transplanted	0	0	0	0	0	0	0	0	0	0	2	0	0	0	0	0	0	0
11553	Kidney transplant failure and rejection	0	0	0	0	0	0	0	0	0	0	2	0	0	0	0	0	0	0
11745	Transplantation of kidney from live donor	0	0	0	0	0	0	0	0	0	0	2	0	0	0	0	0	0	0
17253	Renal transplant planned	0	0	0	0	0	0	0	0	0	0	2	0	0	0	0	0	0	0
18774	Renal transplant with complication, without blame	0	0	0	0	0	0	0	0	0	0	2	0	0	0	0	0	0	0

24361	Transplantation of kidney from cadaver	0	0	0	0	0	0	0	0	0	0	2	0	0	0	0	0	0	0
26862	Exploration of renal transplant	0	0	0	0	0	0	0	0	0	0	2	0	0	0	0	0	0	0
48057	Renal tubulo-interstitial disordrs in transplant rejectn	0	0	0	0	0	0	0	0	0	0	2	0	0	0	0	0	0	0
48121	Transplant nephrectomy	0	0	0	0	0	0	0	0	0	0	2	0	0	0	0	0	0	0
54990	Kidney transplant with complication, without blame	0	0	0	0	0	0	0	0	0	0	2	0	0	0	0	0	0	0
55151	Autotransplant of kidney	0	0	0	0	0	0	0	0	0	0	2	0	0	0	0	0	0	0
66705	Allotransplantation of kidney from live donor	0	0	0	0	0	0	0	0	0	0	2	0	0	0	0	0	0	0
70874	Other specified transplantation of kidney	0	0	0	0	0	0	0	0	0	0	2	0	0	0	0	0	0	0
89924	Allotransplantation of kidney from cadaver, heart-beating	0	0	0	0	0	0	0	0	0	0	2	0	0	0	0	0	0	0
90952	Pre-transplantation of kidney work-up, recipient	0	0	0	0	0	0	0	0	0	0	2	0	0	0	0	0	0	0
96133	Allotransplantation kidney from cadaver, heart non-beating	0	0	0	0	0	0	0	0	0	0	2	0	0	0	0	0	0	0
98364	Allotransplantation of kidney from cadaver	0	0	0	0	0	0	0	0	0	0	2	0	0	0	0	0	0	0
100693	[X]Renal tubulo-interstitial disorders/transplant rejection	0	0	0	0	0	0	0	0	0	0	2	0	0	0	0	0	0	0
105811	renal transplant rejection	0	0	0	0	0	0	0	0	0	0	2	0	0	0	0	0	0	0
10081	Chronic uraemia	0	0	0	0	0	0	0	0	0	0	2	0	0	0	0	0	0	0
57987	Hyperten heart&renal dis+both(congestv)heart and renal fail	0	0	0	0	0	0	0	0	0	0	2	0	0	0	0	0	0	0
7045	H/O: Admission in last year for diabetes foot problem	0	0	0	0	0	0	0	0	0	0	0	2	0	0	0	0	0	0
18056	Foot abnormality - diabetes related	0	0	0	0	0	0	0	0	0	0	0	2	0	0	0	0	0	0
46963	Insulin-dependent diabetes mellitus with renal complications	0	0	0	0	0	0	0	0	0	0	0	2	0	0	0	0	0	0
106360	Erectile dysfunction due to diabetes mellitus	0	0	0	0	0	0	0	0	0	0	0	2	0	0	0	0	0	0
103902	Type II diabetes mellitus with arthropathy	0	0	0	0	0	0	0	0	0	0	0	2	0	0	0	0	0	0
108007	Type I diabetes mellitus with multiple complications	0	0	0	0	0	0	0	0	0	0	0	2	0	0	0	0	0	0
109837	Type I diabetes mellitus with renal complications	0	0	0	0	0	0	0	0	0	0	0	2	0	0	0	0	0	0
108005	Type 2 diabetes mellitus with multiple complications	0	0	0	0	0	0	0	0	0	0	0	2	0	0	0	0	0	0

56803	NIDDM with peripheral circulatory disorder	0	0	0	0	0	0	0	0	0	0	0	2	0	0	0	0	0	0
104323	Type II diabetes mellitus with gangrene	0	0	0	0	0	0	0	0	0	0	0	2	0	0	0	0	0	0
109197	Type II diabetes mellitus with neuropathic arthropathy	0	0	0	0	0	0	0	0	0	0	0	2	0	0	0	0	0	0
109051	Insulin dependent diabetes mellitus with gangrene	0	0	0	0	0	0	0	0	0	0	0	2	0	0	0	0	0	0
109865	Type 2 diabetes mellitus with polyneuropathy	0	0	0	0	0	0	0	0	0	0	0	2	0	0	0	0	0	0
104639	Type II diabetes mellitus with peripheral angiopathy	0	0	0	0	0	0	0	0	0	0	0	2	0	0	0	0	0	0
69124	IDDM with peripheral circulatory disorder	0	0	0	0	0	0	0	0	0	0	0	2	0	0	0	0	0	0
107881	Clinical diabetic nephropathy	0	0	0	0	0	0	0	0	0	0	0	2	0	0	0	0	0	0
108724	Type I diabetes mellitus with gastroparesis	0	0	0	0	0	0	0	0	0	0	0	2	0	0	0	0	0	0
18142	Diabetic cheiroarthropathy	0	0	0	0	0	0	0	0	0	0	0	2	0	0	0	0	0	0
64283	Other specified diabetes mellitus with unspecified comps	0	0	0	0	0	0	0	0	0	0	0	2	0	0	0	0	0	0
99231	Type I diabetes mellitus with mononeuropathy	0	0	0	0	0	0	0	0	0	0	0	2	0	0	0	0	0	0
91942	Type I diabetes mellitus with multiple complications	0	0	0	0	0	0	0	0	0	0	0	2	0	0	0	0	0	0
40401	Non-insulin dependent diabetes mellitus with gangrene	0	0	0	0	0	0	0	0	0	0	0	2	0	0	0	0	0	0
45491	Diabetes mellitus with unspecified complication	0	0	0	0	0	0	0	0	0	0	0	2	0	0	0	0	0	0
54212	Non-insulin-dependent d m with peripheral angiopath	0	0	0	0	0	0	0	0	0	0	0	2	0	0	0	0	0	0
62146	Non-insulin-dependent diabetes mellitus with multiple comps	0	0	0	0	0	0	0	0	0	0	0	2	0	0	0	0	0	0
93468	Type 1 diabetes mellitus with peripheral angiopathy	0	0	0	0	0	0	0	0	0	0	0	2	0	0	0	0	0	0
54856	Diabetes mellitus, adult onset, with ketoacidosis	0	0	0	0	0	0	0	0	0	0	0	2	0	0	0	0	0	0
49640	O/E - left chronic diabetic foot ulcer	0	0	0	0	0	0	0	0	0	0	0	2	0	0	0	0	0	0
31053	[D]Widespread diabetic foot gangrene	0	0	0	0	0	0	0	0	0	0	0	2	0	0	0	0	0	0
68390	Type 1 diabetes mellitus with ulcer	0	0	0	0	0	0	0	0	0	0	0	2	0	0	0	0	0	0
35399	Diabetes mellitus with peripheral circulatory disorder	0	0	0	0	0	0	0	0	0	0	0	2	0	0	0	0	0	0
98704	Insulin dependent diabetes mellitus with ulcer	0	0	0	0	0	0	0	0	0	0	0	2	0	0	0	0	0	0

53634	[D]Gangrene of toe in diabetic	0	0	0	0	0	0	0	0	0	0	0	2	0	0	0	0	0	0
12736	Type 2 diabetes mellitus with gangrene	0	0	0	0	0	0	0	0	0	0	0	2	0	0	0	0	0	0
102946	Insulin-dependent diabetes mellitus with renal complications	0	0	0	0	0	0	0	0	0	0	0	2	0	0	0	0	0	0
64449	Unspecified diabetes mellitus with multiple complications	0	0	0	0	0	0	0	0	0	0	0	2	0	0	0	0	0	0
64357	Diabetes mellitus NOS with unspecified complication	0	0	0	0	0	0	0	0	0	0	0	2	0	0	0	0	0	0
65704	Type 2 diabetes mellitus with ulcer	0	0	0	0	0	0	0	0	0	0	0	2	0	0	0	0	0	0
55075	Type II diabetes mellitus with ulcer	0	0	0	0	0	0	0	0	0	0	0	2	0	0	0	0	0	0
65267	Type 2 diabetes mellitus with multiple complications	0	0	0	0	0	0	0	0	0	0	0	2	0	0	0	0	0	0
62352	Type I diabetes mellitus with arthropathy	0	0	0	0	0	0	0	0	0	0	0	2	0	0	0	0	0	0
59253	Type 2 diabetes mellitus with arthropathy	0	0	0	0	0	0	0	0	0	0	0	2	0	0	0	0	0	0
7328	Cellulitis in diabetic foot	0	0	0	0	0	0	0	0	0	0	0	2	0	0	0	0	0	0
102112	Type I diabetes mellitus with gangrene	0	0	0	0	0	0	0	0	0	0	0	2	0	0	0	0	0	0
57333	Diabetic cheiropathy	0	0	0	0	0	0	0	0	0	0	0	2	0	0	0	0	0	0
49074	Type 2 diabetes mellitus with ulcer	0	0	0	0	0	0	0	0	0	0	0	2	0	0	0	0	0	0
18642	Type 1 diabetes mellitus with arthropathy	0	0	0	0	0	0	0	0	0	0	0	2	0	0	0	0	0	0
18143	Type II diabetes mellitus with arthropathy	0	0	0	0	0	0	0	0	0	0	0	2	0	0	0	0	0	0
54899	Type II diabetes mellitus with peripheral angiopathy	0	0	0	0	0	0	0	0	0	0	0	2	0	0	0	0	0	0
18683	Type 1 diabetes mellitus with ulcer	0	0	0	0	0	0	0	0	0	0	0	2	0	0	0	0	0	0
60499	Insulin dependent diabetes mellitus with gangrene	0	0	0	0	0	0	0	0	0	0	0	2	0	0	0	0	0	0
60699	Type 2 diabetes mellitus with peripheral angiopathy	0	0	0	0	0	0	0	0	0	0	0	2	0	0	0	0	0	0
9881	Mixed diabetic ulcer - foot	0	0	0	0	0	0	0	0	0	0	0	2	0	0	0	0	0	0
10098	Other specified diabetes mellitus with other spec comps	0	0	0	0	0	0	0	0	0	0	0	2	0	0	0	0	0	0
34450	Hyperosmolar non-ketotic state in type 2 diabetes mellitus	0	0	0	0	0	0	0	0	0	0	0	2	0	0	0	0	0	0
99311	Type I diabetes mellitus with ophthalmic complications	0	0	0	0	0	0	0	0	0	0	0	2	0	0	0	0	0	0

91646	Type II diabetes mellitus with ulcer	0	0	0	0	0	0	0	0	0	0	0	2	0	0	0	0	0	0
43227	Type II diabetes mellitus with multiple complications	0	0	0	0	0	0	0	0	0	0	0	2	0	0	0	0	0	0
100292	[X]Unspecified diabetes mellitus with renal complications	0	0	0	0	0	0	0	0	0	0	0	2	0	0	0	0	0	0
33807	Diabetes mellitus, adult with gangrene	0	0	0	0	0	0	0	0	0	0	0	2	0	0	0	0	0	0
63762	Diabetes mellitus, adult onset, + unspecified complication	0	0	0	0	0	0	0	0	0	0	0	2	0	0	0	0	0	0
49869	Type 2 diabetes mellitus with arthropathy	0	0	0	0	0	0	0	0	0	0	0	2	0	0	0	0	0	0
101881	Impaired vision due to diabetic retinopathy	0	0	0	0	0	0	0	0	0	0	0	2	0	0	0	0	0	0
44443	Insulin dependent diabetes mellitus with ulcer	0	0	0	0	0	0	0	0	0	0	0	2	0	0	0	0	0	0
101311	Insulin dependent diabetes mellitus with polyneuropathy	0	0	0	0	0	0	0	0	0	0	0	2	0	0	0	0	0	0
102163	Insulin dependent diabetes mellitus with nephropathy	0	0	0	0	0	0	0	0	0	0	0	2	0	0	0	0	0	0
69993	Type 1 diabetes mellitus with gangrene	0	0	0	0	0	0	0	0	0	0	0	2	0	0	0	0	0	0
51957	Type I diabetes mellitus with ulcer	0	0	0	0	0	0	0	0	0	0	0	2	0	0	0	0	0	0
93878	Type I diabetes mellitus with ulcer	0	0	0	0	0	0	0	0	0	0	0	2	0	0	0	0	0	0
32556	Diabetes with gangrene	0	0	0	0	0	0	0	0	0	0	0	2	0	0	0	0	0	0
70448	Diabetes mellitus, juvenile +peripheral circulatory disorder	0	0	0	0	0	0	0	0	0	0	0	2	0	0	0	0	0	0
62384	O/E - right chronic diabetic foot ulcer	0	0	0	0	0	0	0	0	0	0	0	2	0	0	0	0	0	0
34912	Non-insulin dependent diabetes mellitus with ulcer	0	0	0	0	0	0	0	0	0	0	0	2	0	0	0	0	0	0
34152	Diabetic peripheral angiopathy	0	0	0	0	0	0	0	0	0	0	0	2	0	0	0	0	0	0
35116	O/E - Left diabetic foot - ulcerated	0	0	0	0	0	0	0	0	0	0	0	2	0	0	0	0	0	0
42729	Type I diabetes mellitus with hypoglycaemic coma	0	0	0	0	0	0	0	0	0	0	0	2	0	0	0	0	0	0
62107	Type II diabetes mellitus with gangrene	0	0	0	0	0	0	0	0	0	0	0	2	0	0	0	0	0	0
102740	Type 1 diabetes mellitus with ophthalmic complications	0	0	0	0	0	0	0	0	0	0	0	2	0	0	0	0	0	0
32403	Diabetes mellitus with gangrene	0	0	0	0	0	0	0	0	0	0	0	2	0	0	0	0	0	0
37806	Type 2 diabetes mellitus with peripheral angiopathy	0	0	0	0	0	0	0	0	0	0	0	2	0	0	0	0	0	0

68792	Diabetes mellitus, juvenile type, + unspecified complication	0	0	0	0	0	0	0	0	0	0	0	2	0	0	0	0	0	0
101735	Insulin-dependent diabetes mellitus with neurological comps	0	0	0	0	0	0	0	0	0	0	0	2	0	0	0	0	0	0
100964	Type II diabetes mellitus with ophthalmic complications	0	0	0	0	0	0	0	0	0	0	0	2	0	0	0	0	0	0
33343	Diabetes mellitus with other specified manifestation	0	0	0	0	0	0	0	0	0	0	0	2	0	0	0	0	0	0
100770	Insulin dependent diabetes mellitus with diabetic cataract	0	0	0	0	0	0	0	0	0	0	0	2	0	0	0	0	0	0
65025	Diabetes mellitus NOS with peripheral circulatory disorder	0	0	0	0	0	0	0	0	0	0	0	2	0	0	0	0	0	0
102201	Type II diabetes mellitus with nephropathy	0	0	0	0	0	0	0	0	0	0	0	2	0	0	0	0	0	0
46150	Type 2 diabetes mellitus with gangrene	0	0	0	0	0	0	0	0	0	0	0	2	0	0	0	0	0	0
27921	Foot abnormality - diabetes related	0	0	0	0	0	0	0	0	0	0	0	2	0	0	0	0	0	0
35316	O/E - Right diabetic foot - ulcerated	0	0	0	0	0	0	0	0	0	0	0	2	0	0	0	0	0	0
24327	Ischaemic ulcer diabetic foot	0	0	0	0	0	0	0	0	0	0	0	2	0	0	0	0	0	0
65616	Insulin dependent diabetes mellitus with arthropathy	0	0	0	0	0	0	0	0	0	0	0	2	0	0	0	0	0	0
17313	Diabetic iritis	0	0	0	0	0	0	0	0	0	0	0	2	0	0	0	0	0	0
45276	Insulin dependent diabetes mellitus with multiple complicat	0	0	0	0	0	0	0	0	0	0	0	2	0	0	0	0	0	0
63371	Diabetes mellitus, adult, + other specified manifestation	0	0	0	0	0	0	0	0	0	0	0	2	0	0	0	0	0	0
64446	Insulin dependent diab mell with peripheral angiopathy	0	0	0	0	0	0	0	0	0	0	0	2	0	0	0	0	0	0
24693	Non-insulin dependent diabetes mellitus with arthropathy	0	0	0	0	0	0	0	0	0	0	0	2	0	0	0	0	0	0
47650	Type 1 diabetes mellitus with multiple complications	0	0	0	0	0	0	0	0	0	0	0	2	0	0	0	0	0	0
63357	Diabetes mellitus, adult, + peripheral circulatory disorder	0	0	0	0	0	0	0	0	0	0	0	2	0	0	0	0	0	0
46290	Other specified diabetes mellitus with multiple comps	0	0	0	0	0	0	0	0	0	0	0	2	0	0	0	0	0	0
70821	Diabetes mellitus NOS with other specified manifestation	0	0	0	0	0	0	0	0	0	0	0	2	0	0	0	0	0	0
52104	Insulin dependent diabetes mellitus with multiple complicatn	0	0	0	0	0	0	0	0	0	0	0	2	0	0	0	0	0	0
99628	[X]Glomerular disorders in diabetes mellitus	0	0	0	0	0	0	0	0	0	0	0	2	0	0	0	0	0	0
2986	Preproliferative diabetic retinopathy	0	0	0	0	0	0	0	0	0	0	0	2	0	0	0	0	0	0

13103	O/E - left eye preproliferative diabetic retinopathy	0	0	0	0	0	0	0	0	0	0	0	2	0	0	0	0	0	0
30477	High risk proliferative diabetic retinopathy	0	0	0	0	0	0	0	0	0	0	0	2	0	0	0	0	0	0
18209	Type 2 diabetes mellitus with renal complications	0	0	0	0	0	0	0	0	0	0	0	2	0	0	0	0	0	0
41049	Type 1 diabetes mellitus with retinopathy	0	0	0	0	0	0	0	0	0	0	0	2	0	0	0	0	0	0
47816	Type II diabetes mellitus with neuropathic arthropathy	0	0	0	0	0	0	0	0	0	0	0	2	0	0	0	0	0	0
17067	Autonomic neuropathy due to diabetes	0	0	0	0	0	0	0	0	0	0	0	2	0	0	0	0	0	0
95343	Type I diabetes mellitus with retinopathy	0	0	0	0	0	0	0	0	0	0	0	2	0	0	0	0	0	0
55842	Non-insulin-dependent diabetes mellitus with neuro comps	0	0	0	0	0	0	0	0	0	0	0	2	0	0	0	0	0	0
49276	Insulin-dependent diabetes mellitus with ophthalmic comps	0	0	0	0	0	0	0	0	0	0	0	2	0	0	0	0	0	0
30294	Type 1 diabetes mellitus with persistent microalbuminuria	0	0	0	0	0	0	0	0	0	0	0	2	0	0	0	0	0	0
93727	Type II diabetes mellitus with diabetic cataract	0	0	0	0	0	0	0	0	0	0	0	2	0	0	0	0	0	0
16502	Diabetes mellitus with renal manifestation	0	0	0	0	0	0	0	0	0	0	0	2	0	0	0	0	0	0
45467	Non-insulin dependent diabetes mellitus with polyneuropathy	0	0	0	0	0	0	0	0	0	0	0	2	0	0	0	0	0	0
6509	Insulin dependent diabetes mellitus with retinopathy	0	0	0	0	0	0	0	0	0	0	0	2	0	0	0	0	0	0
17247	Diabetic mononeuritis NOS	0	0	0	0	0	0	0	0	0	0	0	2	0	0	0	0	0	0
38161	Type I diabetes mellitus with retinopathy	0	0	0	0	0	0	0	0	0	0	0	2	0	0	0	0	0	0
69278	Non-insulin depend diabetes mellitus with diabetic cataract	0	0	0	0	0	0	0	0	0	0	0	2	0	0	0	0	0	0
2340	Diabetic amyotrophy	0	0	0	0	0	0	0	0	0	0	0	2	0	0	0	0	0	0
9835	O/E - diabetic maculopathy present both eyes	0	0	0	0	0	0	0	0	0	0	0	2	0	0	0	0	0	0
50813	Type II diabetes mellitus with mononeuropathy	0	0	0	0	0	0	0	0	0	0	0	2	0	0	0	0	0	0
39809	Insulin dependent diab mell with neuropathic arthropathy	0	0	0	0	0	0	0	0	0	0	0	2	0	0	0	0	0	0
18496	Type 2 diabetes mellitus with retinopathy	0	0	0	0	0	0	0	0	0	0	0	2	0	0	0	0	0	0
42762	Type 2 diabetes mellitus with retinopathy	0	0	0	0	0	0	0	0	0	0	0	2	0	0	0	0	0	0
61344	Type I diabetes mellitus with renal complications	0	0	0	0	0	0	0	0	0	0	0	2	0	0	0	0	0	0

39420	Myasthenic syndrome due to diabetic amyotrophy	0	0	0	0	0	0	0	0	0	0	0	2	0	0	0	0	0	0
49146	Type I diabetes mellitus with neurological complications	0	0	0	0	0	0	0	0	0	0	0	2	0	0	0	0	0	0
13102	O/E - right eye diabetic maculopathy	0	0	0	0	0	0	0	0	0	0	0	2	0	0	0	0	0	0
45499	Kimmelstiel - Wilson disease	0	0	0	0	0	0	0	0	0	0	0	2	0	0	0	0	0	0
11663	Neuropathic diabetic ulcer - foot	0	0	0	0	0	0	0	0	0	0	0	2	0	0	0	0	0	0
7069	Background diabetic retinopathy	0	0	0	0	0	0	0	0	0	0	0	2	0	0	0	0	0	0
13108	O/E - left eye diabetic maculopathy	0	0	0	0	0	0	0	0	0	0	0	2	0	0	0	0	0	0
44779	Type 2 diabetes mellitus with diabetic cataract	0	0	0	0	0	0	0	0	0	0	0	2	0	0	0	0	0	0
48192	Type II diabetes mellitus with diabetic cataract	0	0	0	0	0	0	0	0	0	0	0	2	0	0	0	0	0	0
16491	Diabetes mellitus with polyneuropathy	0	0	0	0	0	0	0	0	0	0	0	2	0	0	0	0	0	0
2342	Diabetic neuropathy	0	0	0	0	0	0	0	0	0	0	0	2	0	0	0	0	0	0
49655	Type II diabetes mellitus with retinopathy	0	0	0	0	0	0	0	0	0	0	0	2	0	0	0	0	0	0
13101	O/E - left eye proliferative diabetic retinopathy	0	0	0	0	0	0	0	0	0	0	0	2	0	0	0	0	0	0
11129	O/E - left eye background diabetic retinopathy	0	0	0	0	0	0	0	0	0	0	0	2	0	0	0	0	0	0
72320	Non-insulin dependent diabetes mellitus with mononeuropathy	0	0	0	0	0	0	0	0	0	0	0	2	0	0	0	0	0	0
97894	Type I diabetes mellitus with exudative maculopathy	0	0	0	0	0	0	0	0	0	0	0	2	0	0	0	0	0	0
33254	Diabetes mellitus with ophthalmic manifestation	0	0	0	0	0	0	0	0	0	0	0	2	0	0	0	0	0	0
91943	Type I diabetes mellitus with polyneuropathy	0	0	0	0	0	0	0	0	0	0	0	2	0	0	0	0	0	0
1323	Diabetic retinopathy	0	0	0	0	0	0	0	0	0	0	0	2	0	0	0	0	0	0
13099	O/E - right eye preproliferative diabetic retinopathy	0	0	0	0	0	0	0	0	0	0	0	2	0	0	0	0	0	0
52041	O/E - left eye stable treated prolif diabetic retinopathy	0	0	0	0	0	0	0	0	0	0	0	2	0	0	0	0	0	0
64571	Type II diabetes mellitus with nephropathy	0	0	0	0	0	0	0	0	0	0	0	2	0	0	0	0	0	0
10755	Non proliferative diabetic retinopathy	0	0	0	0	0	0	0	0	0	0	0	2	0	0	0	0	0	0
7795	Diabetes mellitus with neuropathy	0	0	0	0	0	0	0	0	0	0	0	2	0	0	0	0	0	0

35385	Type 2 diabetes mellitus with neuropathic arthropathy	0	0	0	0	0	0	0	0	0	0	0	2	0	0	0	0	0	0
34283	Diabetes mellitus NOS with ophthalmic manifestation	0	0	0	0	0	0	0	0	0	0	0	2	0	0	0	0	0	0
93922	Diabetes mellitus, juvenile type, with renal manifestation	0	0	0	0	0	0	0	0	0	0	0	2	0	0	0	0	0	0
50225	Type II diabetes mellitus with renal complications	0	0	0	0	0	0	0	0	0	0	0	2	0	0	0	0	0	0
52283	Insulin-dependent diabetes mellitus with neurological comps	0	0	0	0	0	0	0	0	0	0	0	2	0	0	0	0	0	0
47582	Type 1 diabetes mellitus with renal complications	0	0	0	0	0	0	0	0	0	0	0	2	0	0	0	0	0	0
66965	Type 2 diabetes mellitus with neuropathic arthropathy	0	0	0	0	0	0	0	0	0	0	0	2	0	0	0	0	0	0
31790	Polyneuropathy in diabetes	0	0	0	0	0	0	0	0	0	0	0	2	0	0	0	0	0	0
60208	Type I diabetes mellitus with neuropathic arthropathy	0	0	0	0	0	0	0	0	0	0	0	2	0	0	0	0	0	0
2471	Nephrotic syndrome in diabetes mellitus	0	0	0	0	0	0	0	0	0	0	0	2	0	0	0	0	0	0
98071	Insulin-dependent diabetes mellitus with ophthalmic comps	0	0	0	0	0	0	0	0	0	0	0	2	0	0	0	0	0	0
63690	Type 2 diabetes mellitus with gastroparesis	0	0	0	0	0	0	0	0	0	0	0	2	0	0	0	0	0	0
57621	Insulin dependent diabetes mellitus with nephropathy	0	0	0	0	0	0	0	0	0	0	0	2	0	0	0	0	0	0
18387	Type 1 diabetes mellitus with retinopathy	0	0	0	0	0	0	0	0	0	0	0	2	0	0	0	0	0	0
18425	Type 2 diabetes mellitus with polyneuropathy	0	0	0	0	0	0	0	0	0	0	0	2	0	0	0	0	0	0
40962	Non-insulin dependent d m with neuropathic arthropathy	0	0	0	0	0	0	0	0	0	0	0	2	0	0	0	0	0	0
48078	Acute painful diabetic neuropathy	0	0	0	0	0	0	0	0	0	0	0	2	0	0	0	0	0	0
5002	Diabetic polyneuropathy	0	0	0	0	0	0	0	0	0	0	0	2	0	0	0	0	0	0
37315	Diabetic mononeuropathy	0	0	0	0	0	0	0	0	0	0	0	2	0	0	0	0	0	0
18230	Type 1 diabetes mellitus with neuropathic arthropathy	0	0	0	0	0	0	0	0	0	0	0	2	0	0	0	0	0	0
55239	Type 1 diabetes mellitus with gastroparesis	0	0	0	0	0	0	0	0	0	0	0	2	0	0	0	0	0	0
24694	Insulin dependent diabetes mellitus with mononeuropathy	0	0	0	0	0	0	0	0	0	0	0	2	0	0	0	0	0	0
34268	Type 2 diabetes mellitus with neurological complications	0	0	0	0	0	0	0	0	0	0	0	2	0	0	0	0	0	0
66872	Type I diabetes mellitus with nephropathy	0	0	0	0	0	0	0	0	0	0	0	2	0	0	0	0	0	0

35785	Chronic painful diabetic neuropathy	0	0	0	0	0	0	0	0	0	0	0	2	0	0	0	0	0	0
59365	Non-insulin dependent diabetes mellitus with nephropathy	0	0	0	0	0	0	0	0	0	0	0	2	0	0	0	0	0	0
27891	Diabetic Charcot arthropathy	0	0	0	0	0	0	0	0	0	0	0	2	0	0	0	0	0	0
62674	Type 2 diabetes mellitus with mononeuropathy	0	0	0	0	0	0	0	0	0	0	0	2	0	0	0	0	0	0
69748	Diabetes mellitus, juvenile type, + ophthalmic manifestation	0	0	0	0	0	0	0	0	0	0	0	2	0	0	0	0	0	0
10659	Diabetic cataract	0	0	0	0	0	0	0	0	0	0	0	2	0	0	0	0	0	0
11599	Pan retinal photocoagulation for diabetes	0	0	0	0	0	0	0	0	0	0	0	2	0	0	0	0	0	0
50429	Non-insulin-dependent diabetes mellitus with ophthalm comps	0	0	0	0	0	0	0	0	0	0	0	2	0	0	0	0	0	0
67853	Diabetes mellitus	0	0	0	0	0	0	0	0	0	0	0	2	0	0	0	0	0	0
52630	O/E - sight threatening diabetic retinopathy	0	0	0	0	0	0	0	0	0	0	0	2	0	0	0	0	0	0
21983	Type 1 diabetes mellitus with renal complications	0	0	0	0	0	0	0	0	0	0	0	2	0	0	0	0	0	0
68105	Type 1 diabetes mellitus with mononeuropathy	0	0	0	0	0	0	0	0	0	0	0	2	0	0	0	0	0	0
24836	Type 2 diabetes mellitus with nephropathy	0	0	0	0	0	0	0	0	0	0	0	2	0	0	0	0	0	0
65463	High risk non proliferative diabetic retinopathy	0	0	0	0	0	0	0	0	0	0	0	2	0	0	0	0	0	0
47321	Type 2 diabetes mellitus with ophthalmic complications	0	0	0	0	0	0	0	0	0	0	0	2	0	0	0	0	0	0
35107	Diabetes mellitis with nephropathy NOS	0	0	0	0	0	0	0	0	0	0	0	2	0	0	0	0	0	0
22967	Retinal abnormality - diabetes related	0	0	0	0	0	0	0	0	0	0	0	2	0	0	0	0	0	0
22871	Type 1 diabetes mellitus with exudative maculopathy	0	0	0	0	0	0	0	0	0	0	0	2	0	0	0	0	0	0
25591	Type 2 diabetes mellitus with exudative maculopathy	0	0	0	0	0	0	0	0	0	0	0	2	0	0	0	0	0	0
47377	Other specified diabetes mellitus with ophthalmic complicatn	0	0	0	0	0	0	0	0	0	0	0	2	0	0	0	0	0	0
85991	Type II diabetes mellitus with persistent microalbuminuria	0	0	0	0	0	0	0	0	0	0	0	2	0	0	0	0	0	0
12640	Type 2 diabetes mellitus with nephropathy	0	0	0	0	0	0	0	0	0	0	0	2	0	0	0	0	0	0
61829	Type 1 diabetes mellitus with neurological complications	0	0	0	0	0	0	0	0	0	0	0	2	0	0	0	0	0	0
98616	Type II diabetes mellitus with neurological complications	0	0	0	0	0	0	0	0	0	0	0	2	0	0	0	0	0	0

41716	Insulin dependent diabetes mellitus with polyneuropathy	0	0	0	0	0	0	0	0	0	0	0	2	0	0	0	0	0	0
50527	Type II diabetes mellitus with polyneuropathy	0	0	0	0	0	0	0	0	0	0	0	2	0	0	0	0	0	0
41389	Diabetes mellitus, adult onset, + ophthalmic manifestation	0	0	0	0	0	0	0	0	0	0	0	2	0	0	0	0	0	0
10099	Advanced diabetic maculopathy	0	0	0	0	0	0	0	0	0	0	0	2	0	0	0	0	0	0
59725	Type II diabetes mellitus with ophthalmic complications	0	0	0	0	0	0	0	0	0	0	0	2	0	0	0	0	0	0
54008	Type 1 diabetes mellitus with neuropathic arthropathy	0	0	0	0	0	0	0	0	0	0	0	2	0	0	0	0	0	0
16230	Diabetes mellitus with neurological manifestation	0	0	0	0	0	0	0	0	0	0	0	2	0	0	0	0	0	0
44260	Insulin dependent diabetes mellitus with diabetic cataract	0	0	0	0	0	0	0	0	0	0	0	2	0	0	0	0	0	0
22573	Diabetes mellitus NOS with neurological manifestation	0	0	0	0	0	0	0	0	0	0	0	2	0	0	0	0	0	0
10418	Type 1 diabetes mellitus with nephropathy	0	0	0	0	0	0	0	0	0	0	0	2	0	0	0	0	0	0
18777	Type 2 diabetes mellitus with renal complications	0	0	0	0	0	0	0	0	0	0	0	2	0	0	0	0	0	0
61523	Other specified diabetes mellitus with neurological comps	0	0	0	0	0	0	0	0	0	0	0	2	0	0	0	0	0	0
67905	Type II diabetes mellitus with neurological complications	0	0	0	0	0	0	0	0	0	0	0	2	0	0	0	0	0	0
49554	Type 1 diabetes mellitus with diabetic cataract	0	0	0	0	0	0	0	0	0	0	0	2	0	0	0	0	0	0
46301	Type 1 diabetes mellitus with polyneuropathy	0	0	0	0	0	0	0	0	0	0	0	2	0	0	0	0	0	0
13279	Other specified diabetes mellitus with renal complications	0	0	0	0	0	0	0	0	0	0	0	2	0	0	0	0	0	0
70316	Type 2 diabetes mellitus with ophthalmic complications	0	0	0	0	0	0	0	0	0	0	0	2	0	0	0	0	0	0
57278	Type II diabetes mellitus with renal complications	0	0	0	0	0	0	0	0	0	0	0	2	0	0	0	0	0	0
42831	Type 1 diabetes mellitus with neurological complications	0	0	0	0	0	0	0	0	0	0	0	2	0	0	0	0	0	0
3286	Proliferative diabetic retinopathy	0	0	0	0	0	0	0	0	0	0	0	2	0	0	0	0	0	0
95351	Type II diabetes mellitus with mononeuropathy	0	0	0	0	0	0	0	0	0	0	0	2	0	0	0	0	0	0
47649	Type 1 diabetes mellitus with ophthalmic complications	0	0	0	0	0	0	0	0	0	0	0	2	0	0	0	0	0	0
24571	Asymptomatic diabetic neuropathy	0	0	0	0	0	0	0	0	0	0	0	2	0	0	0	0	0	0
44033	Diabetic mononeuritis multiplex	0	0	0	0	0	0	0	0	0	0	0	2	0	0	0	0	0	0

39317	Diabetes mellitus, adult onset, + neurological manifestation	0	0	0	0	0	0	0	0	0	0	0	2	0	0	0	0	0	0
47328	O/E - right eye stable treated prolif diabetic retinopathy	0	0	0	0	0	0	0	0	0	0	0	2	0	0	0	0	0	0
11433	O/E - right eye background diabetic retinopathy	0	0	0	0	0	0	0	0	0	0	0	2	0	0	0	0	0	0
3837	Diabetic maculopathy	0	0	0	0	0	0	0	0	0	0	0	2	0	0	0	0	0	0
35105	Diabetes mellitus	0	0	0	0	0	0	0	0	0	0	0	2	0	0	0	0	0	0
2475	Diabetic nephropathy	0	0	0	0	0	0	0	0	0	0	0	2	0	0	0	0	0	0
61670	Diab mellit insulin-glucose infus acute myocardial infarct	0	0	0	0	0	0	0	0	0	0	0	2	0	0	0	0	0	0
47409	Type II diabetes mellitus with polyneuropathy	0	0	0	0	0	0	0	0	0	0	0	2	0	0	0	0	0	0
93875	Insulin dependent diabetes mellitus with retinopathy	0	0	0	0	0	0	0	0	0	0	0	2	0	0	0	0	0	0
52303	Non-insulin-dependent diabetes mellitus with renal comps	0	0	0	0	0	0	0	0	0	0	0	2	0	0	0	0	0	0
44982	Type 2 diabetes mellitus with diabetic cataract	0	0	0	0	0	0	0	0	0	0	0	2	0	0	0	0	0	0
45919	Type 2 diabetes mellitus with neurological complications	0	0	0	0	0	0	0	0	0	0	0	2	0	0	0	0	0	0
13097	O/E - right eye proliferative diabetic retinopathy	0	0	0	0	0	0	0	0	0	0	0	2	0	0	0	0	0	0
11626	Diabetic retinopathy NOS	0	0	0	0	0	0	0	0	0	0	0	2	0	0	0	0	0	0
58604	Type II diabetes mellitus with retinopathy	0	0	0	0	0	0	0	0	0	0	0	2	0	0	0	0	0	0
17545	Type I diabetes mellitus with diabetic cataract	0	0	0	0	0	0	0	0	0	0	0	2	0	0	0	0	0	0
17262	Non-insulin-dependent diabetes mellitus with retinopathy	0	0	0	0	0	0	0	0	0	0	0	2	0	0	0	0	0	0
59903	Diabetic amyotrophy	0	0	0	0	0	0	0	0	0	0	0	2	0	0	0	0	0	0
47584	Advanced diabetic retinal disease	0	0	0	0	0	0	0	0	0	0	0	2	0	0	0	0	0	0
26054	Type 2 diabetes mellitus with persistent proteinuria	0	0	0	0	0	0	0	0	0	0	0	2	0	0	0	0	0	0
30323	Type 1 diabetes mellitus with persistent proteinuria	0	0	0	0	0	0	0	0	0	0	0	2	0	0	0	0	0	0
60796	Type II diabetes mellitus with persistent proteinuria	0	0	0	0	0	0	0	0	0	0	0	2	0	0	0	0	0	0
102620	Type I diabetes mellitus with persistent microalbuminuria	0	0	0	0	0	0	0	0	0	0	0	2	0	0	0	0	0	0
18390	Type 2 diabetes mellitus with persistent microalbuminuria	0	0	0	0	0	0	0	0	0	0	0	2	0	0	0	0	0	0

318	Malignant neoplasm of glottis	0	0	0	0	0	0	0	0	0	0	0	0	2	0	0	0	0	0
319	MALIGNANT NEOPLASM OF LARYNX	0	0	0	0	0	0	0	0	0	0	0	0	2	0	0	0	0	0
348	CA FEMALE BREAST	0	0	0	0	0	0	0	0	0	0	0	0	2	0	0	0	0	0
579	[M]Malignant melanoma NOS	0	0	0	0	0	0	0	0	0	0	0	0	2	0	0	0	0	0
779	MALIGNANT NEOPLASM OF URINARY BLADDER	0	0	0	0	0	0	0	0	0	0	0	0	2	0	0	0	0	0
780	Malignant neoplasm of prostate	0	0	0	0	0	0	0	0	0	0	0	0	2	0	0	0	0	0
865	Malignant melanoma of skin	0	0	0	0	0	0	0	0	0	0	0	0	2	0	0	0	0	0
876	Basal cell carcinoma	0	0	0	0	0	0	0	0	0	0	0	0	2	0	0	0	0	0
1056	MALIGNANT NEOPLASM OF OTHER AND UNSPECIFIED SITE NOS	0	0	0	0	0	0	0	0	0	0	0	0	2	0	0	0	0	0
1062	Malignant neoplasm of oesophagus	0	0	0	0	0	0	0	0	0	0	0	0	2	0	0	0	0	0
1220	MALIGNANT NEOPLASM OF COLON	0	0	0	0	0	0	0	0	0	0	0	0	2	0	0	0	0	0
1599	Malignant neoplasm of kidney parenchyma	0	0	0	0	0	0	0	0	0	0	0	0	2	0	0	0	0	0
1624	[M]SQUAMOUS CELL CARCINOMA NOS	0	0	0	0	0	0	0	0	0	0	0	0	2	0	0	0	0	0
1800	MALIGNANT NEOPLASM OF RECTUM	0	0	0	0	0	0	0	0	0	0	0	0	2	0	0	0	0	0
1940	RODENT ULCER	0	0	0	0	0	0	0	0	0	0	0	0	2	0	0	0	0	0
1986	CANCER OF OVARY	0	0	0	0	0	0	0	0	0	0	0	0	2	0	0	0	0	0
2123	[M]Neuroblastoma NOS	0	0	0	0	0	0	0	0	0	0	0	0	2	0	0	0	0	0
2272	[M]ADENOCARCINOMAS	0	0	0	0	0	0	0	0	0	0	0	0	2	0	0	0	0	0
2492	Malignant neoplasm of skin NOS	0	0	0	0	0	0	0	0	0	0	0	0	2	0	0	0	0	0
2587	LUNG CANCER	0	0	0	0	0	0	0	0	0	0	0	0	2	0	0	0	0	0
2744	MALIGNANT NEOPLASM OF UTERUS, PART UNSPECIFIED	0	0	0	0	0	0	0	0	0	0	0	0	2	0	0	0	0	0
2747	MALIGNANT NEOPLASM OF CERVIX UTERI	0	0	0	0	0	0	0	0	0	0	0	0	2	0	0	0	0	0
2755	CANCERS	0	0	0	0	0	0	0	0	0	0	0	0	2	0	0	0	0	0
2815	MALIGNANT NEOPLASM OF SIGMOID COLON	0	0	0	0	0	0	0	0	0	0	0	0	2	0	0	0	0	0
2890	MALIGNANT NEOPLASM OF ENDOMETRIUM OF CORPUS UTERI	0	0	0	0	0	0	0	0	0	0	0	0	2	0	0	0	0	0
2961	SEMINOMA OF TESTIS	0	0	0	0	0	0	0	0	0	0	0	0	2	0	0	0	0	0
3028	[M]Basal cell carcinoma NOS	0	0	0	0	0	0	0	0	0	0	0	0	2	0	0	0	0	0
3213	Malignant neoplasm of corpus uteri	0	0	0	0	0	0	0	0	0	0	0	0	2	0	0	0	0	0
3230	CERVICAL CARCINOMA (UTERUS)	0	0	0	0	0	0	0	0	0	0	0	0	2	0	0	0	0	0

3357	Carcinoma of digestive organs and peritoneum	0	0	0	0	0	0	0	0	0	0	0	0	2	0	0	0	0	0
3445	EPITHELIOMA BASAL CELL	0	0	0	0	0	0	0	0	0	0	0	0	2	0	0	0	0	0
3541	MALIGNANT NEOPLASM OF PENIS AND OTHER MALE GENITAL ORGANS	0	0	0	0	0	0	0	0	0	0	0	0	2	0	0	0	0	0
3811	MALIGNANT NEOPLASM OF CAECUM	0	0	0	0	0	0	0	0	0	0	0	0	2	0	0	0	0	0
3903	MALIGNANT NEOPLASM OF BRONCHUS OR LUNG NOS	0	0	0	0	0	0	0	0	0	0	0	0	2	0	0	0	0	0
3968	Malignant neoplasm of female breast	0	0	0	0	0	0	0	0	0	0	0	0	2	0	0	0	0	0
3969	[M]INTRACYSTIC CARCINOMA NOS	0	0	0	0	0	0	0	0	0	0	0	0	2	0	0	0	0	0
4118	[M]MYXOID CHONDROSARCOMA	0	0	0	0	0	0	0	0	0	0	0	0	2	0	0	0	0	0
4218	Malignant neoplasm of parathyroid gland	0	0	0	0	0	0	0	0	0	0	0	0	2	0	0	0	0	0
4388	Malignant neoplasm of parotid gland	0	0	0	0	0	0	0	0	0	0	0	0	2	0	0	0	0	0
4473	[M]Ewing's sarcoma	0	0	0	0	0	0	0	0	0	0	0	0	2	0	0	0	0	0
4554	Malignant neoplasm of vulva unspecified	0	0	0	0	0	0	0	0	0	0	0	0	2	0	0	0	0	0
4555	MALIG NEOP OF OTHER AND UNSPECIFIED FEMALE GENITAL ORGANS	0	0	0	0	0	0	0	0	0	0	0	0	2	0	0	0	0	0
4632	Other malignant neoplasm of skin	0	0	0	0	0	0	0	0	0	0	0	0	2	0	0	0	0	0
4852	[M]VERRUCOUS SQUAMOUS CELL CARCINOMA	0	0	0	0	0	0	0	0	0	0	0	0	2	0	0	0	0	0
4865	OESOPHAGEAL CANCER	0	0	0	0	0	0	0	0	0	0	0	0	2	0	0	0	0	0
5052	[M]Osteogenic sarcoma NOS	0	0	0	0	0	0	0	0	0	0	0	0	2	0	0	0	0	0
5069	MECKEL'S DIVERTICULUM	0	0	0	0	0	0	0	0	0	0	0	0	2	0	0	0	0	0
5136	CHORIOCARCINOMA	0	0	0	0	0	0	0	0	0	0	0	0	2	0	0	0	0	0
5637	MALIGNANT NEOPLASM OF THYROID GLAND	0	0	0	0	0	0	0	0	0	0	0	0	2	0	0	0	0	0
5901	RECTAL CARCINOMA	0	0	0	0	0	0	0	0	0	0	0	0	2	0	0	0	0	0
6203	[M]PAPILLARY SEROUS CYSTADENOMA, BORDERLINE MALIGNANCY	0	0	0	0	0	0	0	0	0	0	0	0	2	0	0	0	0	0
6436	[M]Transitional cell carcinoma NOS	0	0	0	0	0	0	0	0	0	0	0	0	2	0	0	0	0	0
6806	MALIGNANT NEOPLASM OF SMALL INTESTINE AND DUODENUM	0	0	0	0	0	0	0	0	0	0	0	0	2	0	0	0	0	0
6935	Malignant neoplasm of transverse colon	0	0	0	0	0	0	0	0	0	0	0	0	2	0	0	0	0	0
6966	[M]Spindle cell carcinoma	0	0	0	0	0	0	0	0	0	0	0	0	2	0	0	0	0	0
7046	Malignant neoplasm of body of uterus	0	0	0	0	0	0	0	0	0	0	0	0	2	0	0	0	0	0
7219	CARCINOMA OF RECTUM	0	0	0	0	0	0	0	0	0	0	0	0	2	0	0	0	0	0
7319	[M]INFILTRATING DUCTULAR CARCINOMA	0	0	0	0	0	0	0	0	0	0	0	0	2	0	0	0	0	0

7476	[M]SEMINOMAS	0	0	0	0	0	0	0	0	0	0	0	0	2	0	0	0	0	0
7484	Mesothelioma	0	0	0	0	0	0	0	0	0	0	0	0	2	0	0	0	0	0
7628	H/O: cancer	0	0	0	0	0	0	0	0	0	0	0	0	2	0	0	0	0	0
7740	Seminoma of undescended testis	0	0	0	0	0	0	0	0	0	0	0	0	2	0	0	0	0	0
7761	H/O Malignant melanoma	0	0	0	0	0	0	0	0	0	0	0	0	2	0	0	0	0	0
7805	MALIGNANT NEOPLASM OF OVARY	0	0	0	0	0	0	0	0	0	0	0	0	2	0	0	0	0	0
7856	[M]DEDIFFERENTIATED LIPOSARCOMA	0	0	0	0	0	0	0	0	0	0	0	0	2	0	0	0	0	0
7900	H/O: carcinoma	0	0	0	0	0	0	0	0	0	0	0	0	2	0	0	0	0	0
7941	[M]CHONDROSARCOMA NOS	0	0	0	0	0	0	0	0	0	0	0	0	2	0	0	0	0	0
7967	[M]Squamous cell neoplasms	0	0	0	0	0	0	0	0	0	0	0	0	2	0	0	0	0	0
7978	Hypernephroma	0	0	0	0	0	0	0	0	0	0	0	0	2	0	0	0	0	0
7982	Malignant neoplasm of common bile duct	0	0	0	0	0	0	0	0	0	0	0	0	2	0	0	0	0	0
8085	[M]SARCOMA NOS	0	0	0	0	0	0	0	0	0	0	0	0	2	0	0	0	0	0
8088	[M]Fibromyxosarcoma	0	0	0	0	0	0	0	0	0	0	0	0	2	0	0	0	0	0
8166	MALIGNANT NEOPLASM OF PANCREAS	0	0	0	0	0	0	0	0	0	0	0	0	2	0	0	0	0	0
8328	[M]ASTROCYTOMA, ANAPLASTIC TYPE	0	0	0	0	0	0	0	0	0	0	0	0	2	0	0	0	0	0
8351	[M]Infiltrating duct carcinoma	0	0	0	0	0	0	0	0	0	0	0	0	2	0	0	0	0	0
8386	Malignant neoplasm of stomach	0	0	0	0	0	0	0	0	0	0	0	0	2	0	0	0	0	0
8523	[M]Glioma NOS	0	0	0	0	0	0	0	0	0	0	0	0	2	0	0	0	0	0
8524	[M]ADENOACANTHOMA	0	0	0	0	0	0	0	0	0	0	0	0	2	0	0	0	0	0
8547	[M]Astrocytoma NOS	0	0	0	0	0	0	0	0	0	0	0	0	2	0	0	0	0	0
8550	Malignant neoplasm of pituitary gland	0	0	0	0	0	0	0	0	0	0	0	0	2	0	0	0	0	0
8627	[M]TUMOUR CELLS, MALIGNANT	0	0	0	0	0	0	0	0	0	0	0	0	2	0	0	0	0	0
8660	[M]OSTEOSARCOMA NOS	0	0	0	0	0	0	0	0	0	0	0	0	2	0	0	0	0	0
8693	Carcinoma of other and unspecified sites	0	0	0	0	0	0	0	0	0	0	0	0	2	0	0	0	0	0
8695	[M]Carcinoma NOS	0	0	0	0	0	0	0	0	0	0	0	0	2	0	0	0	0	0
8711	[M]CHOLANGIOCARCINOMA	0	0	0	0	0	0	0	0	0	0	0	0	2	0	0	0	0	0
8771	Malignant neoplasm of head of pancreas	0	0	0	0	0	0	0	0	0	0	0	0	2	0	0	0	0	0
8918	Malignant neoplasm of liver and intrahepatic bile ducts	0	0	0	0	0	0	0	0	0	0	0	0	2	0	0	0	0	0
8930	[M]Adenocarcinoma NOS	0	0	0	0	0	0	0	0	0	0	0	0	2	0	0	0	0	0

9030	MALIGNANT NEOPLASM OF OTHER AND ILL-DEFINED SITES	0	0	0	0	0	0	0	0	0	0	0	0	2	0	0	0	0	0
9088	MALIGNANT NEOPLASM OF HEPATIC FLEXURE OF COLON	0	0	0	0	0	0	0	0	0	0	0	0	2	0	0	0	0	0
9118	COLONIC CANCER	0	0	0	0	0	0	0	0	0	0	0	0	2	0	0	0	0	0
9156	[M]Oat cell carcinoma	0	0	0	0	0	0	0	0	0	0	0	0	2	0	0	0	0	0
9237	Malignant neoplasm of larynx NOS	0	0	0	0	0	0	0	0	0	0	0	0	2	0	0	0	0	0
9291	[M]Small cell carcinoma NOS	0	0	0	0	0	0	0	0	0	0	0	0	2	0	0	0	0	0
9444	[V]Personal history of malignant neoplasm of genital organ	0	0	0	0	0	0	0	0	0	0	0	0	2	0	0	0	0	0
9447	[M]ENDOMETRIOID CARCINOMA	0	0	0	0	0	0	0	0	0	0	0	0	2	0	0	0	0	0
9470	MALIGNANT NEOPLASM OF FEMALE BREAST NOS	0	0	0	0	0	0	0	0	0	0	0	0	2	0	0	0	0	0
9491	ANAL CARCINOMA	0	0	0	0	0	0	0	0	0	0	0	0	2	0	0	0	0	0
9575	[M]GLIOBLASTOMA MULTIFORME	0	0	0	0	0	0	0	0	0	0	0	0	2	0	0	0	0	0
9600	MESOTHELIOMA OF PLEURA	0	0	0	0	0	0	0	0	0	0	0	0	2	0	0	0	0	0
9622	Malignant neoplasm of cauda equina	0	0	0	0	0	0	0	0	0	0	0	0	2	0	0	0	0	0
9712	[M]Papillary transitional cell carcinoma	0	0	0	0	0	0	0	0	0	0	0	0	2	0	0	0	0	0
9815	[X]Personal history of other neoplasms	0	0	0	0	0	0	0	0	0	0	0	0	2	0	0	0	0	0
9859	[M]Seminoma NOS	0	0	0	0	0	0	0	0	0	0	0	0	2	0	0	0	0	0
9885	[M]Basal cell carcinoma	0	0	0	0	0	0	0	0	0	0	0	0	2	0	0	0	0	0
9902	CARCINOMA OF BONE, CONNECTIVE TISSUE, SKIN AND BREAST	0	0	0	0	0	0	0	0	0	0	0	0	2	0	0	0	0	0
9965	CANCER CARE PLAN	0	0	0	0	0	0	0	0	0	0	0	0	2	0	0	0	0	0
9984	Carcinoma of lip	0	0	0	0	0	0	0	0	0	0	0	0	2	0	0	0	0	0
10178	Gleason grading of prostate cancer	0	0	0	0	0	0	0	0	0	0	0	0	2	0	0	0	0	0
10283	Malignant neoplasm of tongue	0	0	0	0	0	0	0	0	0	0	0	0	2	0	0	0	0	0
10332	CANCER PRIMARY HEALTHCARE MULTIDISCIPLINARY TEAM	0	0	0	0	0	0	0	0	0	0	0	0	2	0	0	0	0	0
10335	CANCER CONFIRMED	0	0	0	0	0	0	0	0	0	0	0	0	2	0	0	0	0	0
10358	MALIGNANT NEOPLASM OF UPPER LOBE, BRONCHUS OR LUNG	0	0	0	0	0	0	0	0	0	0	0	0	2	0	0	0	0	0
10541	[M]PAPILLARY CARCINOMA NOS	0	0	0	0	0	0	0	0	0	0	0	0	2	0	0	0	0	0
10588	[M]LEIOMYOSARCOMA NOS	0	0	0	0	0	0	0	0	0	0	0	0	2	0	0	0	0	0

10668	[M]Renal cell carcinoma	0	0	0	0	0	0	0	0	0	0	0	0	2	0	0	0	0	0
10698	MALIGNANT NEOPLASM OF VAGINAL VAULT	0	0	0	0	0	0	0	0	0	0	0	0	2	0	0	0	0	0
10851	Cerebral tumour - malignant	0	0	0	0	0	0	0	0	0	0	0	0	2	0	0	0	0	0
10864	MALIGNANT NEOPLASM OF DESCENDING COLON	0	0	0	0	0	0	0	0	0	0	0	0	2	0	0	0	0	0
10946	MALIGNANT NEOPLASM OF ASCENDING COLON	0	0	0	0	0	0	0	0	0	0	0	0	2	0	0	0	0	0
10949	Malignant neoplasm of ampulla of Vater	0	0	0	0	0	0	0	0	0	0	0	0	2	0	0	0	0	0
10995	MALIGNANT NEOPLASM OF OTHER AND UNSPECIFIED SITES	0	0	0	0	0	0	0	0	0	0	0	0	2	0	0	0	0	0
11009	MALIG NEOP OTH/ILL-DEFINED SITES DIGESTIVE TRACT/PERITONEUM	0	0	0	0	0	0	0	0	0	0	0	0	2	0	0	0	0	0
11035	PRIMARY MALIGNANT NEOPLASM OF UNKNOWN SITE	0	0	0	0	0	0	0	0	0	0	0	0	2	0	0	0	0	0
11075	CANCER DIAGNOSIS DISCUSSED WITH SIGNIFICANT OTHER	0	0	0	0	0	0	0	0	0	0	0	0	2	0	0	0	0	0
11628	CANCER OF BOWEL	0	0	0	0	0	0	0	0	0	0	0	0	2	0	0	0	0	0
11834	EXCISION BIOPSY OF RODENT ULCER	0	0	0	0	0	0	0	0	0	0	0	0	2	0	0	0	0	0
11922	[M]LENTIGO MALIGNA MELANOMA	0	0	0	0	0	0	0	0	0	0	0	0	2	0	0	0	0	0
11991	Primary vulval cancer	0	0	0	0	0	0	0	0	0	0	0	0	2	0	0	0	0	0
12106	[V]Personal history of malignant neoplasm	0	0	0	0	0	0	0	0	0	0	0	0	2	0	0	0	0	0
12300	[M]Paget's disease, mammary	0	0	0	0	0	0	0	0	0	0	0	0	2	0	0	0	0	0
12309	[M]Gliomas	0	0	0	0	0	0	0	0	0	0	0	0	2	0	0	0	0	0
12388	[M]UROTHELIAL CARCINOMA	0	0	0	0	0	0	0	0	0	0	0	0	2	0	0	0	0	0
12389	MALIGNANT NEOPLASM OF RENAL PELVIS	0	0	0	0	0	0	0	0	0	0	0	0	2	0	0	0	0	0
12427	[M]LOBULAR CARCINOMA NOS	0	0	0	0	0	0	0	0	0	0	0	0	2	0	0	0	0	0
12480	[M]PAGET'S DISEASE AND INTRADUCTAL CARCINOMA OF BREAST	0	0	0	0	0	0	0	0	0	0	0	0	2	0	0	0	0	0
12490	Malignant neoplasm of nose NOS	0	0	0	0	0	0	0	0	0	0	0	0	2	0	0	0	0	0
12492	H/O: neoplasm	0	0	0	0	0	0	0	0	0	0	0	0	2	0	0	0	0	0
12497	[M]MUCINOUS ADENOCARCINOMA	0	0	0	0	0	0	0	0	0	0	0	0	2	0	0	0	0	0

12499	[X]Malignant neoplasm of breast	0	0	0	0	0	0	0	0	0	0	0	0	2	0	0	0	0	0
12539	SARCOMA OF BONE AND CONNECTIVE TISSUE	0	0	0	0	0	0	0	0	0	0	0	0	2	0	0	0	0	0
12580	[M]ADENOSQUAMOUS CARCINOMA	0	0	0	0	0	0	0	0	0	0	0	0	2	0	0	0	0	0
12582	MALIGNANT NEOPLASM OF LOWER LOBE OF LUNG	0	0	0	0	0	0	0	0	0	0	0	0	2	0	0	0	0	0
12609	[M]CARCINOMA, ANAPLASTIC TYPE, NOS	0	0	0	0	0	0	0	0	0	0	0	0	2	0	0	0	0	0
12870	MALIGNANT NEOPLASM OF MAIN BRONCHUS	0	0	0	0	0	0	0	0	0	0	0	0	2	0	0	0	0	0
13243	Malignant neoplasm of trachea, bronchus and lung	0	0	0	0	0	0	0	0	0	0	0	0	2	0	0	0	0	0
13252	Malignant neoplasm of genitourinary organ	0	0	0	0	0	0	0	0	0	0	0	0	2	0	0	0	0	0
13559	Malig neop of kidney and other unspecified urinary organs	0	0	0	0	0	0	0	0	0	0	0	0	2	0	0	0	0	0
13574	[M]METATYPICAL CARCINOMA	0	0	0	0	0	0	0	0	0	0	0	0	2	0	0	0	0	0
14712	MALIGNANT NEOPLASM OF LIP	0	0	0	0	0	0	0	0	0	0	0	0	2	0	0	0	0	0
14792	MALIGNANT NEOPLASM OF OTHER AND UNSPECIFIED PARTS OF MOUTH	0	0	0	0	0	0	0	0	0	0	0	0	2	0	0	0	0	0
14800	Malignant neoplasm of stomach NOS	0	0	0	0	0	0	0	0	0	0	0	0	2	0	0	0	0	0
15148	Malignant neoplasm of testis	0	0	0	0	0	0	0	0	0	0	0	0	2	0	0	0	0	0
15182	Malignant neoplasm of connective and soft tissue, site NOS	0	0	0	0	0	0	0	0	0	0	0	0	2	0	0	0	0	0
15221	MALIGNANT NEOPLASM OF TRACHEA	0	0	0	0	0	0	0	0	0	0	0	0	2	0	0	0	0	0
15223	MALIGNANT NEOPLASM OF URETER	0	0	0	0	0	0	0	0	0	0	0	0	2	0	0	0	0	0
15419	[M]Hypernephroma	0	0	0	0	0	0	0	0	0	0	0	0	2	0	0	0	0	0
15644	MALIGNANT NEOPLASM OF URETHRA	0	0	0	0	0	0	0	0	0	0	0	0	2	0	0	0	0	0
15684	Malignant neoplasm of frontal sinus	0	0	0	0	0	0	0	0	0	0	0	0	2	0	0	0	0	0
15709	Malignant neoplasm of digestive organs and peritoneum	0	0	0	0	0	0	0	0	0	0	0	0	2	0	0	0	0	0
15711	Malignant neoplasm cerebrum (excluding lobes and ventricles)	0	0	0	0	0	0	0	0	0	0	0	0	2	0	0	0	0	0
15868	MALIGNANT NEOPLASM OF SKIN OF TRUNK, EXCLUDING SCROTUM, NOS	0	0	0	0	0	0	0	0	0	0	0	0	2	0	0	0	0	0

15907	MALIGNANT NEOPLASM GALLBLADDER/EXTRAHEPATIC BILE DUCTS NOS	0	0	0	0	0	0	0	0	0	0	0	0	2	0	0	0	0	0
15976	MALIGNANT NEOPLASM OF ABDOMEN	0	0	0	0	0	0	0	0	0	0	0	0	2	0	0	0	0	0
15991	Malignant neoplasm of choroid	0	0	0	0	0	0	0	0	0	0	0	0	2	0	0	0	0	0
16075	MALIGNANT NEOPLASM OF BONE AND ARTICULAR CARTILAGE NOS	0	0	0	0	0	0	0	0	0	0	0	0	2	0	0	0	0	0
16105	MALIGNANT NEOPLASM OF GALLBLADDER	0	0	0	0	0	0	0	0	0	0	0	0	2	0	0	0	0	0
16126	PRIMARY CARCINOMA OF LIVER	0	0	0	0	0	0	0	0	0	0	0	0	2	0	0	0	0	0
16146	[M]Adenocarcinoma with squamous metaplasia	0	0	0	0	0	0	0	0	0	0	0	0	2	0	0	0	0	0
16202	MALIGNANT NEOPLASM OF SKIN OF NOSE (EXTERNAL)	0	0	0	0	0	0	0	0	0	0	0	0	2	0	0	0	0	0
16241	MALIGNANT NEOPLASM OF TONSIL	0	0	0	0	0	0	0	0	0	0	0	0	2	0	0	0	0	0
16280	MALIGNANT NEOPLASM OF NECK NOS	0	0	0	0	0	0	0	0	0	0	0	0	2	0	0	0	0	0
16297	Malignant neoplasm of pharynx unspecified	0	0	0	0	0	0	0	0	0	0	0	0	2	0	0	0	0	0
16298	Malignant neoplasm of retroperitoneum and peritoneum NOS	0	0	0	0	0	0	0	0	0	0	0	0	2	0	0	0	0	0
16639	[V]Personal history of malignant neoplasm of breast	0	0	0	0	0	0	0	0	0	0	0	0	2	0	0	0	0	0
16677	[M]MEDULLARY CARCINOMA NOS	0	0	0	0	0	0	0	0	0	0	0	0	2	0	0	0	0	0
16704	Malignant neoplasm of vertebral column	0	0	0	0	0	0	0	0	0	0	0	0	2	0	0	0	0	0
16723	[M]BRONCHIOLAR CARCINOMA	0	0	0	0	0	0	0	0	0	0	0	0	2	0	0	0	0	0
16874	CARCINOMA OF GENITOURINARY ORGAN	0	0	0	0	0	0	0	0	0	0	0	0	2	0	0	0	0	0
16902	[M]Basal cell adenocarcinoma	0	0	0	0	0	0	0	0	0	0	0	0	2	0	0	0	0	0
16915	Malignant neoplasm of intrahepatic bile ducts	0	0	0	0	0	0	0	0	0	0	0	0	2	0	0	0	0	0
16967	Malignant neoplasm of overlapping lesion of corpus uteri	0	0	0	0	0	0	0	0	0	0	0	0	2	0	0	0	0	0
17064	H/O: malignant neoplasm (*)	0	0	0	0	0	0	0	0	0	0	0	0	2	0	0	0	0	0
17212	[M]RHABDOID SARCOMA	0	0	0	0	0	0	0	0	0	0	0	0	2	0	0	0	0	0
17232	[M]Amelanotic melanoma	0	0	0	0	0	0	0	0	0	0	0	0	2	0	0	0	0	0

17314	[M]WILMS' TUMOUR	0	0	0	0	0	0	0	0	0	0	0	0	2	0	0	0	0	0
17391	Malignant neoplasm of carina of bronchus	0	0	0	0	0	0	0	0	0	0	0	0	2	0	0	0	0	0
17475	Malignant neoplasm of maxilla	0	0	0	0	0	0	0	0	0	0	0	0	2	0	0	0	0	0
17559	MALIGNANT NEOPLASM OF INTESTINAL TRACT, PART UNSPECIFIED	0	0	0	0	0	0	0	0	0	0	0	0	2	0	0	0	0	0
17841	MALIGNANT NEOPLASM OF GLANS PENIS	0	0	0	0	0	0	0	0	0	0	0	0	2	0	0	0	0	0
17874	MESOTHELIOMA OF PERITONEUM	0	0	0	0	0	0	0	0	0	0	0	0	2	0	0	0	0	0
17912	Malignant neoplasm, overlapping lesion of floor of mouth	0	0	0	0	0	0	0	0	0	0	0	0	2	0	0	0	0	0
18245	Malignant neoplasm of skin of lip	0	0	0	0	0	0	0	0	0	0	0	0	2	0	0	0	0	0
18270	EXCISION MALIGNANT SKIN TUMOUR	0	0	0	0	0	0	0	0	0	0	0	0	2	0	0	0	0	0
18314	Malignant neoplasm of bone and articular cartilage	0	0	0	0	0	0	0	0	0	0	0	0	2	0	0	0	0	0
18354	Malignant neoplasm of other specified skin sites	0	0	0	0	0	0	0	0	0	0	0	0	2	0	0	0	0	0
18503	GLEASON PROSTATE GRADE 2-4 (LOW)	0	0	0	0	0	0	0	0	0	0	0	0	2	0	0	0	0	0
18608	Malig neop of bone, connective tissue, skin and breast	0	0	0	0	0	0	0	0	0	0	0	0	2	0	0	0	0	0
18612	Gleason prostate grade 5-7 (medium)	0	0	0	0	0	0	0	0	0	0	0	0	2	0	0	0	0	0
18613	MALIGNANT NEOPLASM OF DUODENUM	0	0	0	0	0	0	0	0	0	0	0	0	2	0	0	0	0	0
18617	Malignant neoplasm of brain	0	0	0	0	0	0	0	0	0	0	0	0	2	0	0	0	0	0
18618	Malignant neoplasm of skin of abdominal wall	0	0	0	0	0	0	0	0	0	0	0	0	2	0	0	0	0	0
18619	MALIGNANT NEOPLASM OF SPLENIC FLEXURE OF COLON	0	0	0	0	0	0	0	0	0	0	0	0	2	0	0	0	0	0
18632	Malignant neoplasm of appendix	0	0	0	0	0	0	0	0	0	0	0	0	2	0	0	0	0	0
18678	Malignant neoplasm of lower lobe bronchus	0	0	0	0	0	0	0	0	0	0	0	0	2	0	0	0	0	0
18712	RENAL MALIGNANT NEOPLASM	0	0	0	0	0	0	0	0	0	0	0	0	2	0	0	0	0	0
18771	[M]Clear cell sarcoma of kidney	0	0	0	0	0	0	0	0	0	0	0	0	2	0	0	0	0	0
18866	H/O: malignancy	0	0	0	0	0	0	0	0	0	0	0	0	2	0	0	0	0	0
18882	MALIGNANT NEOPLASM OF OVERLAPPING LESION OF LIP	0	0	0	0	0	0	0	0	0	0	0	0	2	0	0	0	0	0

19092	Dukes stage B	0	0	0	0	0	0	0	0	0	0	0	0	2	0	0	0	0	0
19141	Malignant neoplasm of ovary and other uterine adnexa	0	0	0	0	0	0	0	0	0	0	0	0	2	0	0	0	0	0
19144	[X]Melanoma and other malignant neoplasms of skin	0	0	0	0	0	0	0	0	0	0	0	0	2	0	0	0	0	0
19162	Malignant neoplasm of anterior wall of urinary bladder	0	0	0	0	0	0	0	0	0	0	0	0	2	0	0	0	0	0
19226	Malignant neoplasm of parietal lobe	0	0	0	0	0	0	0	0	0	0	0	0	2	0	0	0	0	0
19318	Malignant neoplasm of pyloric antrum of stomach	0	0	0	0	0	0	0	0	0	0	0	0	2	0	0	0	0	0
19321	MALIGNANT NEOPLASM OF CONNECTIVE AND SOFT TISSUE OF HAND	0	0	0	0	0	0	0	0	0	0	0	0	2	0	0	0	0	0
19334	[M]Carcinosarcoma NOS	0	0	0	0	0	0	0	0	0	0	0	0	2	0	0	0	0	0
19389	MALIG NEOP OF BONE, CONNECTIVE TISSUE, SKIN AND BREAST OS	0	0	0	0	0	0	0	0	0	0	0	0	2	0	0	0	0	0
19415	MALIGNANT NEOPLASM OF LIP, ORAL CAVITY AND PHARYNX	0	0	0	0	0	0	0	0	0	0	0	0	2	0	0	0	0	0
19423	MALIGNANT NEOPLASM OF MALE BREAST	0	0	0	0	0	0	0	0	0	0	0	0	2	0	0	0	0	0
19437	Osteosarcoma	0	0	0	0	0	0	0	0	0	0	0	0	2	0	0	0	0	0
19444	[X]MALIGNANT MELANOMA OF SKIN, UNSPECIFIED	0	0	0	0	0	0	0	0	0	0	0	0	2	0	0	0	0	0
19475	Malignant neoplasm of descended testis	0	0	0	0	0	0	0	0	0	0	0	0	2	0	0	0	0	0
20084	[M]EPENDYMOMA NOS	0	0	0	0	0	0	0	0	0	0	0	0	2	0	0	0	0	0
20092	Malignant neoplasm of floor of mouth	0	0	0	0	0	0	0	0	0	0	0	0	2	0	0	0	0	0
20160	MALIGNANT NEOPLASM OF EYE	0	0	0	0	0	0	0	0	0	0	0	0	2	0	0	0	0	0
20166	Malignant neoplasm of female genital organ NOS	0	0	0	0	0	0	0	0	0	0	0	0	2	0	0	0	0	0
20170	Pancoast's syndrome	0	0	0	0	0	0	0	0	0	0	0	0	2	0	0	0	0	0
20292	Malignant neoplasm of major salivary glands	0	0	0	0	0	0	0	0	0	0	0	0	2	0	0	0	0	0
20350	[M]YOLK SAC TUMOUR	0	0	0	0	0	0	0	0	0	0	0	0	2	0	0	0	0	0
20685	Malignant neoplasm of axillary tail of female breast	0	0	0	0	0	0	0	0	0	0	0	0	2	0	0	0	0	0
20807	[M]PAPILLARY SQUAMOUS CELL CARCINOMA	0	0	0	0	0	0	0	0	0	0	0	0	2	0	0	0	0	0
20982	[M]Nodular melanoma	0	0	0	0	0	0	0	0	0	0	0	0	2	0	0	0	0	0

21131	[M]SEROUS CYSTADENOMA, BORDERLINE MALIGNANCY	0	0	0	0	0	0	0	0	0	0	0	0	2	0	0	0	0	0
21173	[M]Mullerian mixed tumour	0	0	0	0	0	0	0	0	0	0	0	0	2	0	0	0	0	0
21217	[M]Small cell-large cell carcinoma	0	0	0	0	0	0	0	0	0	0	0	0	2	0	0	0	0	0
21327	MALIGNANT NEOPLASM OF SKIN OF TEMPLE	0	0	0	0	0	0	0	0	0	0	0	0	2	0	0	0	0	0
21330	Malignant neoplasm of retroperitoneum	0	0	0	0	0	0	0	0	0	0	0	0	2	0	0	0	0	0
21447	[M]FIBROBLASTIC OSTEOSARCOMA	0	0	0	0	0	0	0	0	0	0	0	0	2	0	0	0	0	0
21609	[M]Carcinoma	0	0	0	0	0	0	0	0	0	0	0	0	2	0	0	0	0	0
21620	Malignant neoplasm of pylorus of stomach	0	0	0	0	0	0	0	0	0	0	0	0	2	0	0	0	0	0
21681	[M]Nephroblastoma NOS	0	0	0	0	0	0	0	0	0	0	0	0	2	0	0	0	0	0
21682	[M]MALIGNANT TERATOMA, INTERMEDIATE TYPE	0	0	0	0	0	0	0	0	0	0	0	0	2	0	0	0	0	0
21698	Malignant neoplasm of main bronchus NOS	0	0	0	0	0	0	0	0	0	0	0	0	2	0	0	0	0	0
21715	[X]MESOTHELIOMA OF LUNG	0	0	0	0	0	0	0	0	0	0	0	0	2	0	0	0	0	0
21732	[M]MYXOSARCOMA	0	0	0	0	0	0	0	0	0	0	0	0	2	0	0	0	0	0
21741	[M]Follicular adenocarcinoma NOS	0	0	0	0	0	0	0	0	0	0	0	0	2	0	0	0	0	0
21758	[M]Chordoma	0	0	0	0	0	0	0	0	0	0	0	0	2	0	0	0	0	0
21770	[M]MESOTHELIOMA, UNSPECIFIED	0	0	0	0	0	0	0	0	0	0	0	0	2	0	0	0	0	0
21786	Seminoma of descended testis	0	0	0	0	0	0	0	0	0	0	0	0	2	0	0	0	0	0
21833	[M]Duct carcinoma NOS	0	0	0	0	0	0	0	0	0	0	0	0	2	0	0	0	0	0
21847	[M]Follicular carcinoma	0	0	0	0	0	0	0	0	0	0	0	0	2	0	0	0	0	0
21868	[M]Neoplasm, malignant	0	0	0	0	0	0	0	0	0	0	0	0	2	0	0	0	0	0
21914	[M]Intraepithelial carcinoma NOS	0	0	0	0	0	0	0	0	0	0	0	0	2	0	0	0	0	0
22156	[M]MALIGNANT TUMOUR, SMALL CELL TYPE	0	0	0	0	0	0	0	0	0	0	0	0	2	0	0	0	0	0
22163	Carcinoma of caecum	0	0	0	0	0	0	0	0	0	0	0	0	2	0	0	0	0	0
22187	HEPATOCELLULAR CARCINOMA	0	0	0	0	0	0	0	0	0	0	0	0	2	0	0	0	0	0

22290	MALIGNANT NEOPLASM OF CONNECTIVE AND SOFT TISSUE OF THORAX	0	0	0	0	0	0	0	0	0	0	0	0	2	0	0	0	0	0
22382	CANCER DIAGNOSIS DISCUSSED WITH PATIENT	0	0	0	0	0	0	0	0	0	0	0	0	2	0	0	0	0	0
22441	MALIGNANT NEOPLASM OF SUBGLOTTIS	0	0	0	0	0	0	0	0	0	0	0	0	2	0	0	0	0	0
22561	[M]TELANGIECTATIC OSTEOSARCOMA	0	0	0	0	0	0	0	0	0	0	0	0	2	0	0	0	0	0
22650	[M]Angiosarcoma	0	0	0	0	0	0	0	0	0	0	0	0	2	0	0	0	0	0
22692	[M]Acral lentiginous melanoma	0	0	0	0	0	0	0	0	0	0	0	0	2	0	0	0	0	0
22893	Malignant neoplasm of oropharynx	0	0	0	0	0	0	0	0	0	0	0	0	2	0	0	0	0	0
22894	MALIGNANT NEOPLASM OF CARDIO-OESOPHAGEAL JUNCTION OF STOMACH	0	0	0	0	0	0	0	0	0	0	0	0	2	0	0	0	0	0
23081	[M]CARCINOID BRONCHIAL ADENOMA	0	0	0	0	0	0	0	0	0	0	0	0	2	0	0	0	0	0
23083	[M]GLIOBLASTOMA NOS	0	0	0	0	0	0	0	0	0	0	0	0	2	0	0	0	0	0
23085	[M]Epithelioid cell melanoma	0	0	0	0	0	0	0	0	0	0	0	0	2	0	0	0	0	0
23224	TUMOUR STAGING	0	0	0	0	0	0	0	0	0	0	0	0	2	0	0	0	0	0
23380	Malignant neoplasm of nipple of female breast	0	0	0	0	0	0	0	0	0	0	0	0	2	0	0	0	0	0
23389	MALIGNANT NEOPLASM OF NASAL CAVITIES	0	0	0	0	0	0	0	0	0	0	0	0	2	0	0	0	0	0
23399	Malignant neoplasm of upper-outer quadrant of female breast	0	0	0	0	0	0	0	0	0	0	0	0	2	0	0	0	0	0
23433	MALIGNANT NEOPLASM OF EXTRAHEPATIC BILE DUCTS	0	0	0	0	0	0	0	0	0	0	0	0	2	0	0	0	0	0
23480	Malignant neoplasm of perianal skin	0	0	0	0	0	0	0	0	0	0	0	0	2	0	0	0	0	0
23861	MALIGNANT NEOPLASM OF CHEST WALL NOS	0	0	0	0	0	0	0	0	0	0	0	0	2	0	0	0	0	0
23936	[V]Personal history of malignant neoplasm of cervix uteri	0	0	0	0	0	0	0	0	0	0	0	0	2	0	0	0	0	0
24048	MALIGNANT NEOPLASM OF RETROCAECAL TISSUE	0	0	0	0	0	0	0	0	0	0	0	0	2	0	0	0	0	0
24208	[M]SUPERFICIAL SPREADING MELANOMA	0	0	0	0	0	0	0	0	0	0	0	0	2	0	0	0	0	0
24235	Malig neopl peripheral nerves and autonomic nervous system	0	0	0	0	0	0	0	0	0	0	0	0	2	0	0	0	0	0
24370	Malignant neoplasm of anal canal	0	0	0	0	0	0	0	0	0	0	0	0	2	0	0	0	0	0
24374	Carcinoma of lip, oral cavity and pharynx	0	0	0	0	0	0	0	0	0	0	0	0	2	0	0	0	0	0

24375	Dermatofibrosarcoma protuberans	0	0	0	0	0	0	0	0	0	0	0	0	2	0	0	0	0	0
24397	MALIGNANT NEOPLASM OF TONSILLAR FOSSA	0	0	0	0	0	0	0	0	0	0	0	0	2	0	0	0	0	0
24456	MALIG NEOP AUDITORY TUBE, MIDDLE EAR AND MASTOID AIR CELLS	0	0	0	0	0	0	0	0	0	0	0	0	2	0	0	0	0	0
24511	[M]MALIGNANT TUMOUR, GIANT CELL TYPE	0	0	0	0	0	0	0	0	0	0	0	0	2	0	0	0	0	0
24523	[M]PAGET'S DISEASE, EXTRAMAMMARY, EXC PAGET'S DISEASE BONE	0	0	0	0	0	0	0	0	0	0	0	0	2	0	0	0	0	0
24539	[M]CHONDROBLASTIC OSTEOSARCOMA	0	0	0	0	0	0	0	0	0	0	0	0	2	0	0	0	0	0
24551	[M]Melanocarcinoma	0	0	0	0	0	0	0	0	0	0	0	0	2	0	0	0	0	0
24675	Malignant neoplasm of nasopharynx	0	0	0	0	0	0	0	0	0	0	0	0	2	0	0	0	0	0
24852	Malignant neoplasm of lingual tonsil	0	0	0	0	0	0	0	0	0	0	0	0	2	0	0	0	0	0
25245	MALIGNANT NEOPLASM OF SKIN OF FINGER	0	0	0	0	0	0	0	0	0	0	0	0	2	0	0	0	0	0
25535	Primary malignant neoplasm of liver	0	0	0	0	0	0	0	0	0	0	0	0	2	0	0	0	0	0
25602	MALIGNANT MELANOMA OF FINGER	0	0	0	0	0	0	0	0	0	0	0	0	2	0	0	0	0	0
25641	[M]LIVER CELL CARCINOMA	0	0	0	0	0	0	0	0	0	0	0	0	2	0	0	0	0	0
25886	MALIGNANT NEOPLASM OF UPPER LOBE OF LUNG	0	0	0	0	0	0	0	0	0	0	0	0	2	0	0	0	0	0
25961	[M]LARGE CELL CARCINOMA NOS	0	0	0	0	0	0	0	0	0	0	0	0	2	0	0	0	0	0
26034	OTHER MALIGNANT NEOPLASM NOS	0	0	0	0	0	0	0	0	0	0	0	0	2	0	0	0	0	0
26081	GLEASON PROSTATE GRADE 8-10 (HIGH)	0	0	0	0	0	0	0	0	0	0	0	0	2	0	0	0	0	0
26134	Malignant neoplasm of epiglottis	0	0	0	0	0	0	0	0	0	0	0	0	2	0	0	0	0	0
26165	Malignant neoplasm of supraglottis	0	0	0	0	0	0	0	0	0	0	0	0	2	0	0	0	0	0
26197	ADMINISTRATION OF CANCER TREATMENT	0	0	0	0	0	0	0	0	0	0	0	0	2	0	0	0	0	0
26253	[M]Neuroendocrine carcinoma	0	0	0	0	0	0	0	0	0	0	0	0	2	0	0	0	0	0
26393	MALIGNANT NEOPLASM OF LIVER UNSPECIFIED	0	0	0	0	0	0	0	0	0	0	0	0	2	0	0	0	0	0
26413	[M]Pleomorphic carcinoma	0	0	0	0	0	0	0	0	0	0	0	0	2	0	0	0	0	0
26448	Malignant neoplasm of faucial tonsil	0	0	0	0	0	0	0	0	0	0	0	0	2	0	0	0	0	0

26454	Malignant neoplasm/overlapping lesion/feml genital organs	0	0	0	0	0	0	0	0	0	0	0	0	2	0	0	0	0	0
26652	MALIG NEOP NASAL CAVITIES, MIDDLE EAR AND ACCESSORY SINUSES	0	0	0	0	0	0	0	0	0	0	0	0	2	0	0	0	0	0
26813	Malignant neoplasm of larynx, other specified site	0	0	0	0	0	0	0	0	0	0	0	0	2	0	0	0	0	0
26814	[M]HEPATOMA, MALIGNANT	0	0	0	0	0	0	0	0	0	0	0	0	2	0	0	0	0	0
26853	Malignant neoplasm of nipple and areola of female breast	0	0	0	0	0	0	0	0	0	0	0	0	2	0	0	0	0	0
26881	[M]DERMATOFIBROMA PROTUBERANS	0	0	0	0	0	0	0	0	0	0	0	0	2	0	0	0	0	0
27363	[M]Meningioma, malignant	0	0	0	0	0	0	0	0	0	0	0	0	2	0	0	0	0	0
27370	MALIGNANT NEOPLASM SKIN OF OTHER AND UNSPECIFIED PARTS FACE	0	0	0	0	0	0	0	0	0	0	0	0	2	0	0	0	0	0
27439	[M]Kaposi's sarcoma	0	0	0	0	0	0	0	0	0	0	0	0	2	0	0	0	0	0
27440	[M]Linitis plastica	0	0	0	0	0	0	0	0	0	0	0	0	2	0	0	0	0	0
27449	Malignant neoplasm of upper limb NOS	0	0	0	0	0	0	0	0	0	0	0	0	2	0	0	0	0	0
27483	MALIGNANT NEOPLASM OF THYMUS	0	0	0	0	0	0	0	0	0	0	0	0	2	0	0	0	0	0
27509	[M]MESOTHELIOMA, MALIGNANT	0	0	0	0	0	0	0	0	0	0	0	0	2	0	0	0	0	0
27528	Malignant neoplasm of ribs, sternum and clavicle	0	0	0	0	0	0	0	0	0	0	0	0	2	0	0	0	0	0
27540	MALIGNANT NEOPLASM OF RENAL CALYCES	0	0	0	0	0	0	0	0	0	0	0	0	2	0	0	0	0	0
27617	MALIGNANT NEOPLASM OF OVERLAPPING LESION OF VULVA	0	0	0	0	0	0	0	0	0	0	0	0	2	0	0	0	0	0
27653	[M]GLIOMA NOS	0	0	0	0	0	0	0	0	0	0	0	0	2	0	0	0	0	0
27715	Malignant neoplasm of anterior mediastinum	0	0	0	0	0	0	0	0	0	0	0	0	2	0	0	0	0	0
27744	[M]OLIGODENDROGLIOMA NOS	0	0	0	0	0	0	0	0	0	0	0	0	2	0	0	0	0	0
27748	[M]ASTROCYTIC GLIOMA	0	0	0	0	0	0	0	0	0	0	0	0	2	0	0	0	0	0
27846	[M]FIBRILLARY ASTROCYTOMA	0	0	0	0	0	0	0	0	0	0	0	0	2	0	0	0	0	0
27849	[M]Villous adenocarcinoma	0	0	0	0	0	0	0	0	0	0	0	0	2	0	0	0	0	0
27855	Malignant neoplasm of rectosigmoid junction	0	0	0	0	0	0	0	0	0	0	0	0	2	0	0	0	0	0
27897	Malignant neoplasm of anus unspecified	0	0	0	0	0	0	0	0	0	0	0	0	2	0	0	0	0	0

27931	Kaposi's sarcoma of skin	0	0	0	0	0	0	0	0	0	0	0	0	2	0	0	0	0	0
27971	[M]GERMINOMA	0	0	0	0	0	0	0	0	0	0	0	0	2	0	0	0	0	0
28003	CHORIOCARCINOMA	0	0	0	0	0	0	0	0	0	0	0	0	2	0	0	0	0	0
28069	MALIGNANT NEOPLASM OF RETINA	0	0	0	0	0	0	0	0	0	0	0	0	2	0	0	0	0	0
28148	MALIGNANT NEOPLASM OF ADRENAL GLAND	0	0	0	0	0	0	0	0	0	0	0	0	2	0	0	0	0	0
28163	MALIGNANT NEOPLASM OF COLON NOS	0	0	0	0	0	0	0	0	0	0	0	0	2	0	0	0	0	0
28241	MALIGNANT NEOPLASM OF URETERIC ORIFICE	0	0	0	0	0	0	0	0	0	0	0	0	2	0	0	0	0	0
28272	[M]ADENOCARCINOMA, INTESTINAL TYPE	0	0	0	0	0	0	0	0	0	0	0	0	2	0	0	0	0	0
28311	Malignant neoplasm of cervix uteri NOS	0	0	0	0	0	0	0	0	0	0	0	0	2	0	0	0	0	0
28396	[M]MUCINOUS CYSTADENOMA, BORDERLINE MALIGNANCY	0	0	0	0	0	0	0	0	0	0	0	0	2	0	0	0	0	0
28451	Malignant neoplasm of hypopharynx NOS	0	0	0	0	0	0	0	0	0	0	0	0	2	0	0	0	0	0
28556	Malignant melanoma of skin NOS	0	0	0	0	0	0	0	0	0	0	0	0	2	0	0	0	0	0
28559	MALIGNANT NEOPLASM OF PALATE NOS	0	0	0	0	0	0	0	0	0	0	0	0	2	0	0	0	0	0
28599	[M]Liposarcoma NOS	0	0	0	0	0	0	0	0	0	0	0	0	2	0	0	0	0	0
28625	[M]Mucoepidermoid carcinoma	0	0	0	0	0	0	0	0	0	0	0	0	2	0	0	0	0	0
28628	[M]LIPOSARCOMA, WELL DIFFERENTIATED TYPE	0	0	0	0	0	0	0	0	0	0	0	0	2	0	0	0	0	0
28665	MALIGNANT NEOPLASM OF NASOPHARYNX NOS	0	0	0	0	0	0	0	0	0	0	0	0	2	0	0	0	0	0
28836	[M]Retinoblastomas	0	0	0	0	0	0	0	0	0	0	0	0	2	0	0	0	0	0
28881	[V]Personal history of malignant neoplasm of kidney	0	0	0	0	0	0	0	0	0	0	0	0	2	0	0	0	0	0
28919	MALIGNANT NEOPLASM OF CEREBRAL MENINGES	0	0	0	0	0	0	0	0	0	0	0	0	2	0	0	0	0	0
28941	[M]Embryonal carcinoma NOS	0	0	0	0	0	0	0	0	0	0	0	0	2	0	0	0	0	0
29008	[M]HURTHLE CELL ADENOCARCINOMA	0	0	0	0	0	0	0	0	0	0	0	0	2	0	0	0	0	0
29160	Malignant neoplasm of connective and soft tissue of axilla	0	0	0	0	0	0	0	0	0	0	0	0	2	0	0	0	0	0
29283	Malignant neoplasm of other site of respiratory tract	0	0	0	0	0	0	0	0	0	0	0	0	2	0	0	0	0	0

29284	[V]Personal history of malignant neoplasm of lung	0	0	0	0	0	0	0	0	0	0	0	0	2	0	0	0	0	0
29337	[M] Small cell osteosarcoma	0	0	0	0	0	0	0	0	0	0	0	0	2	0	0	0	0	0
29462	Malignant neoplasm of kidney or urinary organs NOS	0	0	0	0	0	0	0	0	0	0	0	0	2	0	0	0	0	0
29524	[M]Basal cell carcinoma, fibroepithelial type	0	0	0	0	0	0	0	0	0	0	0	0	2	0	0	0	0	0
29580	[M]SERTOLI CELL CARCINOMA	0	0	0	0	0	0	0	0	0	0	0	0	2	0	0	0	0	0
29787	[M]Squamous cell carcinoma, keratinising type NOS	0	0	0	0	0	0	0	0	0	0	0	0	2	0	0	0	0	0
29826	MALIGNANT NEOPLASM OF UPPER-INNER QUADRANT OF FEMALE BREAST	0	0	0	0	0	0	0	0	0	0	0	0	2	0	0	0	0	0
29945	[M]Malignant teratoma	0	0	0	0	0	0	0	0	0	0	0	0	2	0	0	0	0	0
30165	MALIGNANT NEOPLASM OF MESORECTUM	0	0	0	0	0	0	0	0	0	0	0	0	2	0	0	0	0	0
30189	[M]Intraductal papillary adenocarcinoma with invasion	0	0	0	0	0	0	0	0	0	0	0	0	2	0	0	0	0	0
30273	[M]PILOCYTIC ASTROCYTOMA	0	0	0	0	0	0	0	0	0	0	0	0	2	0	0	0	0	0
30322	[V]Personal history of malignant neoplasm of urinary organ	0	0	0	0	0	0	0	0	0	0	0	0	2	0	0	0	0	0
30402	Malignant neoplasm of buccal mucosa	0	0	0	0	0	0	0	0	0	0	0	0	2	0	0	0	0	0
30416	[M]COLLOID ADENOCARCINOMA	0	0	0	0	0	0	0	0	0	0	0	0	2	0	0	0	0	0
30511	MALIG NEOP OF OTHER ENDOCRINE GLANDS AND RELATED STRUCTURES	0	0	0	0	0	0	0	0	0	0	0	0	2	0	0	0	0	0
30526	[X]Mesothelioma	0	0	0	0	0	0	0	0	0	0	0	0	2	0	0	0	0	0
30537	Polyneuropathy in malignant disease	0	0	0	0	0	0	0	0	0	0	0	0	2	0	0	0	0	0
30542	MALIG NEOP OF CONNECTIVE AND SOFT TISSUE OF LOWER LEG	0	0	0	0	0	0	0	0	0	0	0	0	2	0	0	0	0	0
30543	Malignant neoplasm of skin of breast	0	0	0	0	0	0	0	0	0	0	0	0	2	0	0	0	0	0
30547	[V]FOLLOW-UP EXAM AFTER RADIOTHERAPY FOR MALIGNANT NEOPLASM	0	0	0	0	0	0	0	0	0	0	0	0	2	0	0	0	0	0
30576	Malignant neoplasm of skin of forehead	0	0	0	0	0	0	0	0	0	0	0	0	2	0	0	0	0	0
30577	MALIGNANT NEOPLASM OF SKIN OF FORE-ARM	0	0	0	0	0	0	0	0	0	0	0	0	2	0	0	0	0	0
30645	Malignant neoplasm of skin of cheek	0	0	0	0	0	0	0	0	0	0	0	0	2	0	0	0	0	0
30700	Malignant neoplasm of oesophagus NOS	0	0	0	0	0	0	0	0	0	0	0	0	2	0	0	0	0	0

30747	MALIGNANT NEOPLASM OF SKIN OF UPPER LIMB AND SHOULDER	0	0	0	0	0	0	0	0	0	0	0	0	2	0	0	0	0	0
30988	[M]Small cell carcinoma	0	0	0	0	0	0	0	0	0	0	0	0	2	0	0	0	0	0
31004	[M]Adenoid squamous cell carcinoma	0	0	0	0	0	0	0	0	0	0	0	0	2	0	0	0	0	0
31026	[M]SPINDLE CELL SARCOMA	0	0	0	0	0	0	0	0	0	0	0	0	2	0	0	0	0	0
31090	[M]Pigmented dermatofibrosarcoma protuberans	0	0	0	0	0	0	0	0	0	0	0	0	2	0	0	0	0	0
31102	Malignant neoplasm of urinary bladder NOS	0	0	0	0	0	0	0	0	0	0	0	0	2	0	0	0	0	0
31188	Malignant neoplasm of lower lobe, bronchus or lung	0	0	0	0	0	0	0	0	0	0	0	0	2	0	0	0	0	0
31210	Hepatoblastoma of liver	0	0	0	0	0	0	0	0	0	0	0	0	2	0	0	0	0	0
31268	Malignant neoplasm of middle lobe	0	0	0	0	0	0	0	0	0	0	0	0	2	0	0	0	0	0
31323	[M]Fibrosarcoma NOS	0	0	0	0	0	0	0	0	0	0	0	0	2	0	0	0	0	0
31364	Malignant neoplasm of cheek mucosa	0	0	0	0	0	0	0	0	0	0	0	0	2	0	0	0	0	0
31393	CARCINOMA GALLBLADDER	0	0	0	0	0	0	0	0	0	0	0	0	2	0	0	0	0	0
31399	MALIGNANT NEOPLASM OF LOWER LIMB NOS	0	0	0	0	0	0	0	0	0	0	0	0	2	0	0	0	0	0
31421	[M]Rhabdomyosarcoma NOS	0	0	0	0	0	0	0	0	0	0	0	0	2	0	0	0	0	0
31546	Malignant neoplasm of central part of female breast	0	0	0	0	0	0	0	0	0	0	0	0	2	0	0	0	0	0
31561	[V]FOLLOW-UP EXAMINATION AFT SURGERY FOR MALIGNANT NEOPLASM	0	0	0	0	0	0	0	0	0	0	0	0	2	0	0	0	0	0
31573	Malignant neoplasm of pleura	0	0	0	0	0	0	0	0	0	0	0	0	2	0	0	0	0	0
31574	[M]GLIOMA, MALIGNANT	0	0	0	0	0	0	0	0	0	0	0	0	2	0	0	0	0	0
31608	Malignant neoplasm of other site of uterine body	0	0	0	0	0	0	0	0	0	0	0	0	2	0	0	0	0	0
31609	[M]Granulosa cell tumour, malignant	0	0	0	0	0	0	0	0	0	0	0	0	2	0	0	0	0	0
31673	[M]OSTEOCLASTOMA, MALIGNANT	0	0	0	0	0	0	0	0	0	0	0	0	2	0	0	0	0	0
31700	Malignant neoplasm of upper lobe bronchus	0	0	0	0	0	0	0	0	0	0	0	0	2	0	0	0	0	0
31767	[M]MEDULLOMYOBLASTOMA	0	0	0	0	0	0	0	0	0	0	0	0	2	0	0	0	0	0
31772	[M]Dermatofibrosarcoma NOS	0	0	0	0	0	0	0	0	0	0	0	0	2	0	0	0	0	0

32022	Malignant neoplasm of cardia of stomach	0	0	0	0	0	0	0	0	0	0	0	0	2	0	0	0	0	0
32024	Malignant neoplasm of upper gum	0	0	0	0	0	0	0	0	0	0	0	0	2	0	0	0	0	0
32174	MALIGNANT NEOPLASM OF MAXILLARY SINUS	0	0	0	0	0	0	0	0	0	0	0	0	2	0	0	0	0	0
32191	[M]DYSGERMINOMA	0	0	0	0	0	0	0	0	0	0	0	0	2	0	0	0	0	0
32213	[M]Malignant tumour, fusiform cell type	0	0	0	0	0	0	0	0	0	0	0	0	2	0	0	0	0	0
32246	[V]Personal history of malignant neoplasm of bronchus	0	0	0	0	0	0	0	0	0	0	0	0	2	0	0	0	0	0
32294	[M]GLUCAGONOMA, MALIGNANT	0	0	0	0	0	0	0	0	0	0	0	0	2	0	0	0	0	0
32351	Squamous cell carcinoma antigen level	0	0	0	0	0	0	0	0	0	0	0	0	2	0	0	0	0	0
32362	MALIGNANT NEOPLASM OF FUNDUS OF STOMACH	0	0	0	0	0	0	0	0	0	0	0	0	2	0	0	0	0	0
32372	Malignant neoplasm of thoracic vertebra	0	0	0	0	0	0	0	0	0	0	0	0	2	0	0	0	0	0
32411	Cancer treatment started	0	0	0	0	0	0	0	0	0	0	0	0	2	0	0	0	0	0
32472	[M]INFLAMMATORY CARCINOMA	0	0	0	0	0	0	0	0	0	0	0	0	2	0	0	0	0	0
32641	[M]Merkel cell carcinoma	0	0	0	0	0	0	0	0	0	0	0	0	2	0	0	0	0	0
32768	MALIGNANT MELANOMA OF BREAST	0	0	0	0	0	0	0	0	0	0	0	0	2	0	0	0	0	0
32955	Malignant neoplasm of other site of cervix	0	0	0	0	0	0	0	0	0	0	0	0	2	0	0	0	0	0
33271	Malignant neoplasm of pinna NEC	0	0	0	0	0	0	0	0	0	0	0	0	2	0	0	0	0	0
33388	Malignant neoplasm of adenoid	0	0	0	0	0	0	0	0	0	0	0	0	2	0	0	0	0	0
33444	MALIGNANT NEOPLASM OF HILUS OF LUNG	0	0	0	0	0	0	0	0	0	0	0	0	2	0	0	0	0	0
33497	[M]Squamous cell carcinoma, microinvasive	0	0	0	0	0	0	0	0	0	0	0	0	2	0	0	0	0	0
33617	MALIGNANT NEOPLASM OF BODY OF UTERUS NOS	0	0	0	0	0	0	0	0	0	0	0	0	2	0	0	0	0	0
33636	[M]Teratoma, malignant, NOS	0	0	0	0	0	0	0	0	0	0	0	0	2	0	0	0	0	0
33682	Malignant neoplasm of skin of lower leg	0	0	0	0	0	0	0	0	0	0	0	0	2	0	0	0	0	0
33775	[M]Adenoid cystic carcinoma	0	0	0	0	0	0	0	0	0	0	0	0	2	0	0	0	0	0
33833	MALIGNANT NEOPLASM OF MANDIBLE	0	0	0	0	0	0	0	0	0	0	0	0	2	0	0	0	0	0

33871	Malignant neoplasm of ileum	0	0	0	0	0	0	0	0	0	0	0	0	2	0	0	0	0	0
33997	MALIGNANT NEOPLASM OF SKIN OF AURICLE (EAR)	0	0	0	0	0	0	0	0	0	0	0	0	2	0	0	0	0	0
34000	[M]CYSTADENOCARCINOMA NOS	0	0	0	0	0	0	0	0	0	0	0	0	2	0	0	0	0	0
34012	Malignant neoplasm of hypopharynx	0	0	0	0	0	0	0	0	0	0	0	0	2	0	0	0	0	0
34015	[M]Bronchiolo-alveolar adenocarcinoma	0	0	0	0	0	0	0	0	0	0	0	0	2	0	0	0	0	0
34030	[M]ENDOMETRIAL STROMAL SARCOMA	0	0	0	0	0	0	0	0	0	0	0	0	2	0	0	0	0	0
34075	MALIG NEOP OF RESPIRATORY TRACT AND INTRATHORACIC ORGANS	0	0	0	0	0	0	0	0	0	0	0	0	2	0	0	0	0	0
34096	[M]GRANULAR CELL CARCINOMA	0	0	0	0	0	0	0	0	0	0	0	0	2	0	0	0	0	0
34110	[M]CARCINOID TUMOUR, MALIGNANT	0	0	0	0	0	0	0	0	0	0	0	0	2	0	0	0	0	0
34252	[M]GLIOSARCOMA	0	0	0	0	0	0	0	0	0	0	0	0	2	0	0	0	0	0
34259	MALIGNANT MELANOMA OF GROIN	0	0	0	0	0	0	0	0	0	0	0	0	2	0	0	0	0	0
34269	[M]Sebaceous adenocarcinoma	0	0	0	0	0	0	0	0	0	0	0	0	2	0	0	0	0	0
34388	MALIGNANT NEOPLASM OF PANCREAS NOS	0	0	0	0	0	0	0	0	0	0	0	0	2	0	0	0	0	0
34395	[M]VERRUCOUS CARCINOMA NOS	0	0	0	0	0	0	0	0	0	0	0	0	2	0	0	0	0	0
34409	Malignant neoplasm of base of tongue dorsal surface	0	0	0	0	0	0	0	0	0	0	0	0	2	0	0	0	0	0
34451	Malignant neoplasm of connective and other soft tissue	0	0	0	0	0	0	0	0	0	0	0	0	2	0	0	0	0	0
34742	MALIGNANT NEOPLASM OF PLEURA NOS	0	0	0	0	0	0	0	0	0	0	0	0	2	0	0	0	0	0
34763	[M]MEDULLOBLASTOMA NOS	0	0	0	0	0	0	0	0	0	0	0	0	2	0	0	0	0	0
34878	MALIGNANT NEOPLASM OF MEDIAL CUNEIFORM	0	0	0	0	0	0	0	0	0	0	0	0	2	0	0	0	0	0
34879	[M]Cylindroid adenocarcinoma	0	0	0	0	0	0	0	0	0	0	0	0	2	0	0	0	0	0
35034	[M]FIBROXANTHOSARCOMA	0	0	0	0	0	0	0	0	0	0	0	0	2	0	0	0	0	0
35039	MALIGNANT NEOPLASM, OVERLAPPING LESION OF BILIARY TRACT	0	0	0	0	0	0	0	0	0	0	0	0	2	0	0	0	0	0
35071	[M]Mixed germ cell tumour	0	0	0	0	0	0	0	0	0	0	0	0	2	0	0	0	0	0
35113	[X]Malignant neoplasm of urinary tract	0	0	0	0	0	0	0	0	0	0	0	0	2	0	0	0	0	0

35180	[X]Malignant neoplasm of digestive organs	0	0	0	0	0	0	0	0	0	0	0	0	2	0	0	0	0	0
35223	[M]Spermatocytic seminoma	0	0	0	0	0	0	0	0	0	0	0	0	2	0	0	0	0	0
35285	[X]MALIGNANT NEOPLASM OF EYE, BRAIN AND OTHER PARTS OF CENT	0	0	0	0	0	0	0	0	0	0	0	0	2	0	0	0	0	0
35325	[X]MALIGNANT NEOPLASM OF RESPIRATORY AND INTRATHORACIC ORGA	0	0	0	0	0	0	0	0	0	0	0	0	2	0	0	0	0	0
35348	[M]Papillary adenocarcinoma NOS	0	0	0	0	0	0	0	0	0	0	0	0	2	0	0	0	0	0
35357	MALIGNANT NEOPLASM OF RECTUM, RECTOSIGMOID JUNCTION AND ANUS	0	0	0	0	0	0	0	0	0	0	0	0	2	0	0	0	0	0
35457	[M]Basosquamous carcinoma	0	0	0	0	0	0	0	0	0	0	0	0	2	0	0	0	0	0
35458	Dukes stage C1	0	0	0	0	0	0	0	0	0	0	0	0	2	0	0	0	0	0
35474	[M]Giant cell carcinoma	0	0	0	0	0	0	0	0	0	0	0	0	2	0	0	0	0	0
35535	MALIGNANT NEOPLASM OF PANCREATIC DUCT	0	0	0	0	0	0	0	0	0	0	0	0	2	0	0	0	0	0
35771	[V]Personal history of malignant neoplasm of thyroid	0	0	0	0	0	0	0	0	0	0	0	0	2	0	0	0	0	0
35795	Malignant neoplasm of Islets of Langerhans	0	0	0	0	0	0	0	0	0	0	0	0	2	0	0	0	0	0
35816	[V]Personal history of malignant neoplasm of bladder	0	0	0	0	0	0	0	0	0	0	0	0	2	0	0	0	0	0
35963	Malignant neoplasm of lateral wall of urinary bladder	0	0	0	0	0	0	0	0	0	0	0	0	2	0	0	0	0	0
36161	Malignant neoplasm of tongue, tip and lateral border	0	0	0	0	0	0	0	0	0	0	0	0	2	0	0	0	0	0
36321	[V]Folow-up exam aft unspec treatment for malignant neoplasm	0	0	0	0	0	0	0	0	0	0	0	0	2	0	0	0	0	0
36371	MALIGNANT NEOPLASM OF OVERLAPPING LESION OF BRONCHUS & LUNG	0	0	0	0	0	0	0	0	0	0	0	0	2	0	0	0	0	0
36495	Carcinoma common bile duct	0	0	0	0	0	0	0	0	0	0	0	0	2	0	0	0	0	0
36530	[M]ALVEOLAR CELL CARCINOMA	0	0	0	0	0	0	0	0	0	0	0	0	2	0	0	0	0	0
36716	Malignant neoplasm of floor of mouth NOS	0	0	0	0	0	0	0	0	0	0	0	0	2	0	0	0	0	0
36731	MALIGNANT NEOPLASM OF CANTHUS	0	0	0	0	0	0	0	0	0	0	0	0	2	0	0	0	0	0
36870	[M]Adenosarcoma	0	0	0	0	0	0	0	0	0	0	0	0	2	0	0	0	0	0
36876	[M]Eosinophil carcinoma	0	0	0	0	0	0	0	0	0	0	0	0	2	0	0	0	0	0
36899	MALIGNANT MELANOMA OF TOE	0	0	0	0	0	0	0	0	0	0	0	0	2	0	0	0	0	0

36949	MALIGNANT NEOPLASM OF OTHER SITE OF URINARY BLADDER	0	0	0	0	0	0	0	0	0	0	0	0	2	0	0	0	0	0
37016	Malignant neoplasm of sebaceous gland	0	0	0	0	0	0	0	0	0	0	0	0	2	0	0	0	0	0
37096	MALIGNANT NEOPLASM OF TONGUE, JUNCTIONAL ZONE	0	0	0	0	0	0	0	0	0	0	0	0	2	0	0	0	0	0
37165	Malignant neoplasm of scalp	0	0	0	0	0	0	0	0	0	0	0	0	2	0	0	0	0	0
37306	[V]Personal history of malignant neoplasm of prostate	0	0	0	0	0	0	0	0	0	0	0	0	2	0	0	0	0	0
37328	Malignant neoplasm of vagina	0	0	0	0	0	0	0	0	0	0	0	0	2	0	0	0	0	0
37354	[M]CLEAR CELL ADENOCARCINOMA NOS	0	0	0	0	0	0	0	0	0	0	0	0	2	0	0	0	0	0
37473	[M]Cerebellar sarcoma NOS	0	0	0	0	0	0	0	0	0	0	0	0	2	0	0	0	0	0
37477	[M]Schwannoma, malignant	0	0	0	0	0	0	0	0	0	0	0	0	2	0	0	0	0	0
37510	[M]Carcinoma in pleomorphic adenoma	0	0	0	0	0	0	0	0	0	0	0	0	2	0	0	0	0	0
37516	Malignant neoplasm of uvula	0	0	0	0	0	0	0	0	0	0	0	0	2	0	0	0	0	0
37542	[M]TERATOCARCINOMA	0	0	0	0	0	0	0	0	0	0	0	0	2	0	0	0	0	0
37549	KAPOSI'S SARCOMA OF PALATE	0	0	0	0	0	0	0	0	0	0	0	0	2	0	0	0	0	0
37553	Malignant neoplasm of lip	0	0	0	0	0	0	0	0	0	0	0	0	2	0	0	0	0	0
37590	MALIGNANT NEOPLASM OF HARD PALATE	0	0	0	0	0	0	0	0	0	0	0	0	2	0	0	0	0	0
37618	Malignant neoplasm of axilla NOS	0	0	0	0	0	0	0	0	0	0	0	0	2	0	0	0	0	0
37621	[M]ENDODERMAL SINUS TUMOUR	0	0	0	0	0	0	0	0	0	0	0	0	2	0	0	0	0	0
37680	[M]FIBROUS HISTIOCYTOMA, MALIGNANT	0	0	0	0	0	0	0	0	0	0	0	0	2	0	0	0	0	0
37688	[M]ACINAR CELL CARCINOMA	0	0	0	0	0	0	0	0	0	0	0	0	2	0	0	0	0	0
37724	MALIGNANT NEOPLASM OF RETROMOLAR AREA	0	0	0	0	0	0	0	0	0	0	0	0	2	0	0	0	0	0
37793	FIGO STAGING OF GYNAECOLOGICAL MALIGNANCY	0	0	0	0	0	0	0	0	0	0	0	0	2	0	0	0	0	0
37805	MALIGNANT NEOPLASM OF CRICOID CARTILAGE	0	0	0	0	0	0	0	0	0	0	0	0	2	0	0	0	0	0
37810	Malignant neoplasm of trachea NOS	0	0	0	0	0	0	0	0	0	0	0	0	2	0	0	0	0	0
37842	Malignant neoplasm of rib	0	0	0	0	0	0	0	0	0	0	0	0	2	0	0	0	0	0

37859	MALIGNANT NEOPLASM OF CARDIA OF STOMACH NOS	0	0	0	0	0	0	0	0	0	0	0	0	2	0	0	0	0	0
37872	Malignant melanoma of lower leg	0	0	0	0	0	0	0	0	0	0	0	0	2	0	0	0	0	0
37916	Malignant neoplasm of other specified mouth parts	0	0	0	0	0	0	0	0	0	0	0	0	2	0	0	0	0	0
37940	MALIGNANT NEOPLASM OF PHARYNGEAL RECESS	0	0	0	0	0	0	0	0	0	0	0	0	2	0	0	0	0	0
37969	Malignant neoplasm of skin of chest, excluding breast	0	0	0	0	0	0	0	0	0	0	0	0	2	0	0	0	0	0
38442	[M]Serous cystadenocarcinoma, NOS	0	0	0	0	0	0	0	0	0	0	0	0	2	0	0	0	0	0
38454	[M]Basaloid carcinoma	0	0	0	0	0	0	0	0	0	0	0	0	2	0	0	0	0	0
38475	Malignant neoplasm of other site of female breast NOS	0	0	0	0	0	0	0	0	0	0	0	0	2	0	0	0	0	0
38481	[M]EPITHELIOID HAEMANGIOENDOTHELIOMA, MALIGNANT	0	0	0	0	0	0	0	0	0	0	0	0	2	0	0	0	0	0
38488	Malignant neoplasm of ventral tongue surface NOS	0	0	0	0	0	0	0	0	0	0	0	0	2	0	0	0	0	0
38510	MALIGNANT NEOPLASM OF TESTIS NOS	0	0	0	0	0	0	0	0	0	0	0	0	2	0	0	0	0	0
38551	[M]Gliomatosis cerebri	0	0	0	0	0	0	0	0	0	0	0	0	2	0	0	0	0	0
38575	[M]APOCRINE ADENOCARCINOMA	0	0	0	0	0	0	0	0	0	0	0	0	2	0	0	0	0	0
38689	Malignant melanoma of trunk (excluding scrotum)	0	0	0	0	0	0	0	0	0	0	0	0	2	0	0	0	0	0
38736	MALIGNANT NEOPLASM OF OTHER AND UNSPECIFIED SITE OS	0	0	0	0	0	0	0	0	0	0	0	0	2	0	0	0	0	0
38756	[M]Cystic mesothelioma	0	0	0	0	0	0	0	0	0	0	0	0	2	0	0	0	0	0
38770	[M]EPITHELIAL-MYOEPITHELIAL CARCINOMA	0	0	0	0	0	0	0	0	0	0	0	0	2	0	0	0	0	0
38862	Malignant neoplasm of trigone of urinary bladder	0	0	0	0	0	0	0	0	0	0	0	0	2	0	0	0	0	0
38931	Malignant neoplasm of genitourinary organ OS	0	0	0	0	0	0	0	0	0	0	0	0	2	0	0	0	0	0
38938	Malignant neoplasm of pelvis	0	0	0	0	0	0	0	0	0	0	0	0	2	0	0	0	0	0
38961	Malignant neoplasm of other sites of bronchus or lung	0	0	0	0	0	0	0	0	0	0	0	0	2	0	0	0	0	0
38978	MALIGNANT NEOPLASM OF LIVER AND INTRAHEPATIC BILE DUCTS NOS	0	0	0	0	0	0	0	0	0	0	0	0	2	0	0	0	0	0
39027	[X]MALIGNANT NEOPLASM OF OTHER SPECIFIED SITES	0	0	0	0	0	0	0	0	0	0	0	0	2	0	0	0	0	0
39038	[M]SIGNET RING CARCINOMA	0	0	0	0	0	0	0	0	0	0	0	0	2	0	0	0	0	0

39084	Malignant neoplasm of laryngopharynx	0	0	0	0	0	0	0	0	0	0	0	0	2	0	0	0	0	0
39088	Malignant neoplasm of occipital lobe	0	0	0	0	0	0	0	0	0	0	0	0	2	0	0	0	0	0
39121	[M]Ganglioneuroblastoma	0	0	0	0	0	0	0	0	0	0	0	0	2	0	0	0	0	0
39386	[M]MIXED GLIOMA	0	0	0	0	0	0	0	0	0	0	0	0	2	0	0	0	0	0
39388	[M]OLFACTORY NEUROBLASTOMA	0	0	0	0	0	0	0	0	0	0	0	0	2	0	0	0	0	0
39413	Malignant neoplasm of pelvic peritoneum	0	0	0	0	0	0	0	0	0	0	0	0	2	0	0	0	0	0
39430	MALIGNANT NEOPLASM OF LIP, ORAL CAVITY AND PHARYNX NOS	0	0	0	0	0	0	0	0	0	0	0	0	2	0	0	0	0	0
39531	MALIG NEO, OVERLAPPING LESION OF HEART, MEDIASTINUM & PLEURA	0	0	0	0	0	0	0	0	0	0	0	0	2	0	0	0	0	0
39554	MALIGNANT NEOPLASM OF VALLECULA	0	0	0	0	0	0	0	0	0	0	0	0	2	0	0	0	0	0
39590	MALIGNANT NEOPLASM, OVERLAPPING LESION OF ACCESSORY SINUSES	0	0	0	0	0	0	0	0	0	0	0	0	2	0	0	0	0	0
39760	[M]INFILTRATING DUCT AND LOBULAR CARCINOMA	0	0	0	0	0	0	0	0	0	0	0	0	2	0	0	0	0	0
39863	[V]Personal history of malignant neoplasm of nose	0	0	0	0	0	0	0	0	0	0	0	0	2	0	0	0	0	0
39870	Malignant neoplasm of tail of pancreas	0	0	0	0	0	0	0	0	0	0	0	0	2	0	0	0	0	0
39878	MALIGNANT MELANOMA OF POPLITEAL FOSSA AREA	0	0	0	0	0	0	0	0	0	0	0	0	2	0	0	0	0	0
39897	MALIGNANT NEOPLASM OF PYRIFORM SINUS	0	0	0	0	0	0	0	0	0	0	0	0	2	0	0	0	0	0
39899	MALIGNANT NEOPLASM OF CRANIOPHARYNGEAL DUCT	0	0	0	0	0	0	0	0	0	0	0	0	2	0	0	0	0	0
39923	Malignant neoplasm of middle lobe of lung	0	0	0	0	0	0	0	0	0	0	0	0	2	0	0	0	0	0
40014	Malignant neoplasm of soft tissue of face	0	0	0	0	0	0	0	0	0	0	0	0	2	0	0	0	0	0
40240	[M]HEPATOCELLULAR CARCINOMA NOS	0	0	0	0	0	0	0	0	0	0	0	0	2	0	0	0	0	0
40292	MALIGNANT NEOPLASM OF SOFT PALATE	0	0	0	0	0	0	0	0	0	0	0	0	2	0	0	0	0	0
40303	[M]MIXED EPITHELIOID AND SPINDLE MELANOMA	0	0	0	0	0	0	0	0	0	0	0	0	2	0	0	0	0	0
40359	[M]JUVENILE BREAST CARCINOMA	0	0	0	0	0	0	0	0	0	0	0	0	2	0	0	0	0	0
40437	MALIGNANT NEOPLASM OF OTHER SPECIFIED SITE OF EYE	0	0	0	0	0	0	0	0	0	0	0	0	2	0	0	0	0	0
40438	[M]Bile duct carcinoma	0	0	0	0	0	0	0	0	0	0	0	0	2	0	0	0	0	0

40443	MALIGNANT NEOPLASM OF SWEAT GLAND	0	0	0	0	0	0	0	0	0	0	0	0	2	0	0	0	0	0
40492	[M]Triton tumour, malignant	0	0	0	0	0	0	0	0	0	0	0	0	2	0	0	0	0	0
40557	MALIGNANT NEOPLASM OF TONGUE NOS	0	0	0	0	0	0	0	0	0	0	0	0	2	0	0	0	0	0
40592	[X]MALIGNANT NEOPLASM OF MESOTHELIAL AND SOFT TISSUE	0	0	0	0	0	0	0	0	0	0	0	0	2	0	0	0	0	0
40595	[X]Malignant neoplasm of bronchus or lung	0	0	0	0	0	0	0	0	0	0	0	0	2	0	0	0	0	0
40598	[X]MALIGNANT NEOPLASM OF FEMALE GENITAL ORGANS	0	0	0	0	0	0	0	0	0	0	0	0	2	0	0	0	0	0
40608	[X]Malignant neoplasm of thyroid and other endocrine glands	0	0	0	0	0	0	0	0	0	0	0	0	2	0	0	0	0	0
40622	[M]Mucoid cell carcinoma	0	0	0	0	0	0	0	0	0	0	0	0	2	0	0	0	0	0
40671	[X]Malignant neoplasm of male genital organs	0	0	0	0	0	0	0	0	0	0	0	0	2	0	0	0	0	0
40749	[X]Malignant neoplasm of bone and articular cartilage	0	0	0	0	0	0	0	0	0	0	0	0	2	0	0	0	0	0
40810	Malignant neoplasm of body of pancreas	0	0	0	0	0	0	0	0	0	0	0	0	2	0	0	0	0	0
40814	Malignant neoplasm of tibia	0	0	0	0	0	0	0	0	0	0	0	0	2	0	0	0	0	0
40966	MALIGNANT NEOPLASM OF SACRAL VERTEBRA	0	0	0	0	0	0	0	0	0	0	0	0	2	0	0	0	0	0
40986	Dukes stage A	0	0	0	0	0	0	0	0	0	0	0	0	2	0	0	0	0	0
41011	MALIG NEOP OF BONE, CONNECTIVE TISSUE, SKIN AND BREAST NOS	0	0	0	0	0	0	0	0	0	0	0	0	2	0	0	0	0	0
41215	Malignant neoplasm of pyloric canal of stomach	0	0	0	0	0	0	0	0	0	0	0	0	2	0	0	0	0	0
41278	MALIGNANT MELANOMA OF EXTERNAL SURFACE OF CHEEK	0	0	0	0	0	0	0	0	0	0	0	0	2	0	0	0	0	0
41313	[M]Bile duct cystadenocarcinoma	0	0	0	0	0	0	0	0	0	0	0	0	2	0	0	0	0	0
41362	Malignant neoplasm of thoracic oesophagus	0	0	0	0	0	0	0	0	0	0	0	0	2	0	0	0	0	0
41490	Malignant melanoma of foot	0	0	0	0	0	0	0	0	0	0	0	0	2	0	0	0	0	0
41515	[X]Malignant neoplasm/central nervous system, unspecified	0	0	0	0	0	0	0	0	0	0	0	0	2	0	0	0	0	0
41520	MALIGNANT NEOPLASM OF BRAIN NOS	0	0	0	0	0	0	0	0	0	0	0	0	2	0	0	0	0	0
41523	MALIGNANT NEOPLASM OF MIDDLE LOBE BRONCHUS	0	0	0	0	0	0	0	0	0	0	0	0	2	0	0	0	0	0
41530	Malignant neoplasm of other sites of tongue	0	0	0	0	0	0	0	0	0	0	0	0	2	0	0	0	0	0

41571	Malignant neoplasm of bladder neck	0	0	0	0	0	0	0	0	0	0	0	0	2	0	0	0	0	0
41695	[M]Primitive neuroectodermal tumour	0	0	0	0	0	0	0	0	0	0	0	0	2	0	0	0	0	0
41816	[M]SQUAMOUS CELL CARCINOMA, SMALL CELL, NON-KERATINISING	0	0	0	0	0	0	0	0	0	0	0	0	2	0	0	0	0	0
41931	MALIGNANT NEOPLASM OF CHEEK NOS	0	0	0	0	0	0	0	0	0	0	0	0	2	0	0	0	0	0
41958	MALIGNANT NEOPLASM OF LOWER EYELID	0	0	0	0	0	0	0	0	0	0	0	0	2	0	0	0	0	0
42012	MALIGNANT NEOPLASM OF POSTERIOR WALL OF URINARY BLADDER	0	0	0	0	0	0	0	0	0	0	0	0	2	0	0	0	0	0
42023	MALIGNANT NEOPLASM OF URACHUS	0	0	0	0	0	0	0	0	0	0	0	0	2	0	0	0	0	0
42070	MALIGNANT NEOPLASM OF LOWER-OUTER QUADRANT OF FEMALE BREAST	0	0	0	0	0	0	0	0	0	0	0	0	2	0	0	0	0	0
42082	[M]Alveolar rhabdomyosarcoma	0	0	0	0	0	0	0	0	0	0	0	0	2	0	0	0	0	0
42153	MALIGNANT MELANOMA OF OTHER SPECIFIED SKIN SITE	0	0	0	0	0	0	0	0	0	0	0	0	2	0	0	0	0	0
42193	MALIGNANT NEOPLASM OF LESSER CURVE OF STOMACH UNSPECIFIED	0	0	0	0	0	0	0	0	0	0	0	0	2	0	0	0	0	0
42218	Malignant neoplasm of other specified sites	0	0	0	0	0	0	0	0	0	0	0	0	2	0	0	0	0	0
42361	Dukes stage C2	0	0	0	0	0	0	0	0	0	0	0	0	2	0	0	0	0	0
42416	Malignant neoplasm of lower third of oesophagus	0	0	0	0	0	0	0	0	0	0	0	0	2	0	0	0	0	0
42426	Malignant neoplasm of frontal lobe	0	0	0	0	0	0	0	0	0	0	0	0	2	0	0	0	0	0
42429	Malignant neoplasm overlapping lesion of skin	0	0	0	0	0	0	0	0	0	0	0	0	2	0	0	0	0	0
42460	Malignant neoplasm of pineal gland	0	0	0	0	0	0	0	0	0	0	0	0	2	0	0	0	0	0
42509	[V]FOLLOW-UP EXAM AFT COMBINED TREATMENT FOR MALIG NEOPLASM	0	0	0	0	0	0	0	0	0	0	0	0	2	0	0	0	0	0
42542	[M]PAGET'S DISEASE AND INFILTRATING BREAST DUCT CARCINOMA	0	0	0	0	0	0	0	0	0	0	0	0	2	0	0	0	0	0
42553	[M]Adenocarcinoma with cartilaginous and osseous metaplasia	0	0	0	0	0	0	0	0	0	0	0	0	2	0	0	0	0	0
42566	MALIGNANT NEOPLASM OF LOWER LOBE, BRONCHUS OR LUNG NOS	0	0	0	0	0	0	0	0	0	0	0	0	2	0	0	0	0	0
42569	MALIGNANT NEOPLASM OF RESPIRATORY TRACT NOS	0	0	0	0	0	0	0	0	0	0	0	0	2	0	0	0	0	0
42707	MALIGNANT NEOPLASM OF SKIN OF UPPER ARM	0	0	0	0	0	0	0	0	0	0	0	0	2	0	0	0	0	0
42714	Malignant melanoma of ankle	0	0	0	0	0	0	0	0	0	0	0	0	2	0	0	0	0	0

42856	Malignant neoplasm of nasal cavities NOS	0	0	0	0	0	0	0	0	0	0	0	0	2	0	0	0	0	0
43087	Malignant neoplasm of eyelid including canthus	0	0	0	0	0	0	0	0	0	0	0	0	2	0	0	0	0	0
43111	Malignant neoplasm of laryngeal cartilage	0	0	0	0	0	0	0	0	0	0	0	0	2	0	0	0	0	0
43122	Malignant neoplasm of skin of shoulder	0	0	0	0	0	0	0	0	0	0	0	0	2	0	0	0	0	0
43151	[X]Malignant neoplasm/bone+articular cartilage, unspecified	0	0	0	0	0	0	0	0	0	0	0	0	2	0	0	0	0	0
43200	MALIGNANT NEOPLASM OF OROPHARYNX NOS	0	0	0	0	0	0	0	0	0	0	0	0	2	0	0	0	0	0
43311	[V]Personal history of malignant neoplasm of larynx	0	0	0	0	0	0	0	0	0	0	0	0	2	0	0	0	0	0
43390	MALIGNANT NEOPLASM OF SMALL INTESTINE NOS	0	0	0	0	0	0	0	0	0	0	0	0	2	0	0	0	0	0
43392	Malignant neoplasm of penis, part unspecified	0	0	0	0	0	0	0	0	0	0	0	0	2	0	0	0	0	0
43400	MALIGNANT NEOPLASM OF GUM	0	0	0	0	0	0	0	0	0	0	0	0	2	0	0	0	0	0
43431	Malignant neoplasm of base of tongue	0	0	0	0	0	0	0	0	0	0	0	0	2	0	0	0	0	0
43435	MALIGNANT NEOPLASM OF OTHER SITE OF CERVIX NOS	0	0	0	0	0	0	0	0	0	0	0	0	2	0	0	0	0	0
43463	MALIGNANT MELANOMA OF BACK	0	0	0	0	0	0	0	0	0	0	0	0	2	0	0	0	0	0
43475	MALIG NEOP OF CONNECTIVE AND SOFT TISSUE HEAD, FACE AND NECK	0	0	0	0	0	0	0	0	0	0	0	0	2	0	0	0	0	0
43479	Malignant neoplasm of jejunum	0	0	0	0	0	0	0	0	0	0	0	0	2	0	0	0	0	0
43490	[X]OTHER SPECIFIED CARCINOMAS OF LIVER	0	0	0	0	0	0	0	0	0	0	0	0	2	0	0	0	0	0
43548	MALIGNANT NEOPLASM OF POSTCRICOID REGION	0	0	0	0	0	0	0	0	0	0	0	0	2	0	0	0	0	0
43572	MALIGNANT NEOPLASM OF BODY OF STOMACH	0	0	0	0	0	0	0	0	0	0	0	0	2	0	0	0	0	0
43614	MALIGNANT NEOPLASM/BONES+ARTICULAR CARTILAGE/LIMB,UNSPFD	0	0	0	0	0	0	0	0	0	0	0	0	2	0	0	0	0	0
43619	MALIGNANT NEOPLASM OF SKIN OF NECK	0	0	0	0	0	0	0	0	0	0	0	0	2	0	0	0	0	0
43642	Malignant neoplasm of dorsal surface of tongue	0	0	0	0	0	0	0	0	0	0	0	0	2	0	0	0	0	0
43715	MALIGNANT MELANOMA OF UMBILICUS	0	0	0	0	0	0	0	0	0	0	0	0	2	0	0	0	0	0
43717	[M]Verrucous epidermoid carcinoma	0	0	0	0	0	0	0	0	0	0	0	0	2	0	0	0	0	0
43761	Malignant neoplasm of labia majora	0	0	0	0	0	0	0	0	0	0	0	0	2	0	0	0	0	0

43781	MALIGNANT NEOPLASM OF DORSUM OF TONGUE NOS	0	0	0	0	0	0	0	0	0	0	0	0	2	0	0	0	0	0
43865	[M]IMMATURE TERATOMA	0	0	0	0	0	0	0	0	0	0	0	0	2	0	0	0	0	0
43940	Malignant neoplasm of isthmus of uterine body	0	0	0	0	0	0	0	0	0	0	0	0	2	0	0	0	0	0
44061	[M]SPINDLE CELL MELANOMA NOS	0	0	0	0	0	0	0	0	0	0	0	0	2	0	0	0	0	0
44074	[M]Mucin-producing adenocarcinoma	0	0	0	0	0	0	0	0	0	0	0	0	2	0	0	0	0	0
44089	Malignant neoplasm of brain stem	0	0	0	0	0	0	0	0	0	0	0	0	2	0	0	0	0	0
44108	Malignant neoplasm of retroperitoneum and peritoneum	0	0	0	0	0	0	0	0	0	0	0	0	2	0	0	0	0	0
44139	MALIGNANT NEOPLASM OF ANTERIOR WALL OF NASOPHARYNX	0	0	0	0	0	0	0	0	0	0	0	0	2	0	0	0	0	0
44157	[M]Melanosarcoma NOS	0	0	0	0	0	0	0	0	0	0	0	0	2	0	0	0	0	0
44169	MALIGNANT NEOPLASM OF UPPER LOBE, BRONCHUS OR LUNG NOS	0	0	0	0	0	0	0	0	0	0	0	0	2	0	0	0	0	0
44356	Malig neop other/ill-defined sites resp/intrathoracic organs	0	0	0	0	0	0	0	0	0	0	0	0	2	0	0	0	0	0
44399	PRIMARY MALIGNANT NEOPLASM OF LIVER NOS	0	0	0	0	0	0	0	0	0	0	0	0	2	0	0	0	0	0
44421	[V]Folow-up exam aft other treatment for malignant neoplasm	0	0	0	0	0	0	0	0	0	0	0	0	2	0	0	0	0	0
44452	Malignant neoplasm of vomer	0	0	0	0	0	0	0	0	0	0	0	0	2	0	0	0	0	0
44609	Malignant neoplasm of ilium	0	0	0	0	0	0	0	0	0	0	0	0	2	0	0	0	0	0
44778	[M]Adenocarcinoma in tubulovillous adenoma	0	0	0	0	0	0	0	0	0	0	0	0	2	0	0	0	0	0
44805	MALIG NEOP OF CONNECTIVE AND SOFT TISSUE THIGH AND UPPER LEG	0	0	0	0	0	0	0	0	0	0	0	0	2	0	0	0	0	0
44884	Malignant neoplasm of other urinary organs	0	0	0	0	0	0	0	0	0	0	0	0	2	0	0	0	0	0
44930	[M]Papillary serous cystadenocarcinoma	0	0	0	0	0	0	0	0	0	0	0	0	2	0	0	0	0	0
44996	Malignant neoplasm of dome of urinary bladder	0	0	0	0	0	0	0	0	0	0	0	0	2	0	0	0	0	0
45071	MALIGNANT NEOPLASM OF CONNECTIVE AND SOFT TISSUE OF ABDOMEN	0	0	0	0	0	0	0	0	0	0	0	0	2	0	0	0	0	0
45077	Malignant neoplasm of skin of back	0	0	0	0	0	0	0	0	0	0	0	0	2	0	0	0	0	0
45139	MALIGNANT MELANOMA OF EXTERNAL SURFACE OF NOSE	0	0	0	0	0	0	0	0	0	0	0	0	2	0	0	0	0	0
45154	MALIGNANT NEOPLASM OF CEREBELLUM	0	0	0	0	0	0	0	0	0	0	0	0	2	0	0	0	0	0

45222	MALIGNANT NEOPLASM OF LOWER-INNER QUADRANT OF FEMALE BREAST	0	0	0	0	0	0	0	0	0	0	0	0	2	0	0	0	0	0
45260	[X]Malignant neoplasm of urinary organ	0	0	0	0	0	0	0	0	0	0	0	0	2	0	0	0	0	0
45262	[X]MALIGNANT NEOPLASM OF MALE GENITAL ORGAN, UNSPECIFIED	0	0	0	0	0	0	0	0	0	0	0	0	2	0	0	0	0	0
45267	Malignant neoplasm of other and ill defined site NOS	0	0	0	0	0	0	0	0	0	0	0	0	2	0	0	0	0	0
45306	MALIGNANT MELANOMA OF NECK	0	0	0	0	0	0	0	0	0	0	0	0	2	0	0	0	0	0
45307	CARCINOMA OF RESPIRATORY TRACT AND INTRATHORACIC ORGANS	0	0	0	0	0	0	0	0	0	0	0	0	2	0	0	0	0	0
45408	Malignant neoplasm of anterior portion of floor of mouth	0	0	0	0	0	0	0	0	0	0	0	0	2	0	0	0	0	0
45458	[M]SQUAMOUS CELL CARCINOMA, SPINDLE CELL TYPE	0	0	0	0	0	0	0	0	0	0	0	0	2	0	0	0	0	0
45490	Malignant neoplasm of corpus uteri NOS	0	0	0	0	0	0	0	0	0	0	0	0	2	0	0	0	0	0
45510	[M]LYMPHOEPITHELIAL CARCINOMA	0	0	0	0	0	0	0	0	0	0	0	0	2	0	0	0	0	0
45531	[M]GEMISTOCYTIC ASTROCYTOMA	0	0	0	0	0	0	0	0	0	0	0	0	2	0	0	0	0	0
45667	MALIGNANT NEOPLASM OF ORBIT	0	0	0	0	0	0	0	0	0	0	0	0	2	0	0	0	0	0
45755	Malignant melanoma of fore-arm	0	0	0	0	0	0	0	0	0	0	0	0	2	0	0	0	0	0
45760	Malignant melanoma of trunk, excluding scrotum, NOS	0	0	0	0	0	0	0	0	0	0	0	0	2	0	0	0	0	0
45766	[X]Malignant neoplasm of intestinal tract, part unspecified	0	0	0	0	0	0	0	0	0	0	0	0	2	0	0	0	0	0
45793	Malignant neoplasm of myometrium of corpus uteri	0	0	0	0	0	0	0	0	0	0	0	0	2	0	0	0	0	0
45803	[V]Personal history of malignant neoplasm of tongue	0	0	0	0	0	0	0	0	0	0	0	0	2	0	0	0	0	0
45922	Malignant neoplasm, overlapping lesion of eye and adnexa	0	0	0	0	0	0	0	0	0	0	0	0	2	0	0	0	0	0
45986	Malignant neoplasm of lateral portion of floor of mouth	0	0	0	0	0	0	0	0	0	0	0	0	2	0	0	0	0	0
46008	Malignant neoplasm skin other and unspec part of face NOS	0	0	0	0	0	0	0	0	0	0	0	0	2	0	0	0	0	0
46113	[M]PAPILLARY CYSTADENOMA, BORDERLINE MALIGNANCY	0	0	0	0	0	0	0	0	0	0	0	0	2	0	0	0	0	0
46114	MALIG NEOP OTHER/ILL-DEFINED SITES LIP, ORAL CAVITY, PHARYNX	0	0	0	0	0	0	0	0	0	0	0	0	2	0	0	0	0	0
46153	MALIGNANT NEOPLASM OF PARAMETRIUM	0	0	0	0	0	0	0	0	0	0	0	0	2	0	0	0	0	0
46159	MALIGNANT NEOPLASM OF CLOACOGENIC ZONE	0	0	0	0	0	0	0	0	0	0	0	0	2	0	0	0	0	0

46255	MALIGNANT MELANOMA OF LOWER LIMB AND HIP	0	0	0	0	0	0	0	0	0	0	0	0	2	0	0	0	0	0
46282	[V]Personal history of malignant neoplasm of bone	0	0	0	0	0	0	0	0	0	0	0	0	2	0	0	0	0	0
46404	[M]OLIGODENDROBLASTOMA	0	0	0	0	0	0	0	0	0	0	0	0	2	0	0	0	0	0
46458	Malignant neoplasm of skin of perineum	0	0	0	0	0	0	0	0	0	0	0	0	2	0	0	0	0	0
46548	Malignant neoplasm of pharyngeal tonsil	0	0	0	0	0	0	0	0	0	0	0	0	2	0	0	0	0	0
46581	[M]Pleomorphic cell sarcoma	0	0	0	0	0	0	0	0	0	0	0	0	2	0	0	0	0	0
46613	Malignant neoplasm of specified parts of peritoneum	0	0	0	0	0	0	0	0	0	0	0	0	2	0	0	0	0	0
46728	MALIGNANT NEOPLASM OF ANTERIOR EPIGLOTTIS	0	0	0	0	0	0	0	0	0	0	0	0	2	0	0	0	0	0
46741	[M]AMELOBLASTIC ODONTOSARCOMA	0	0	0	0	0	0	0	0	0	0	0	0	2	0	0	0	0	0
46761	[M]PAPILLARY AND FOLLICULAR ADENOCARCINOMA	0	0	0	0	0	0	0	0	0	0	0	0	2	0	0	0	0	0
46769	[M]EPENDYMOBLASTOMA	0	0	0	0	0	0	0	0	0	0	0	0	2	0	0	0	0	0
46771	[M]HEPATOCELLULAR CARCINOMA, FIBROLAMELLAR	0	0	0	0	0	0	0	0	0	0	0	0	2	0	0	0	0	0
46779	[V]Personal history of malignant neoplasm of uterine body	0	0	0	0	0	0	0	0	0	0	0	0	2	0	0	0	0	0
46789	MALIGNANT NEOPLASM OF CHOROID PLEXUS	0	0	0	0	0	0	0	0	0	0	0	0	2	0	0	0	0	0
46792	Malignant neoplasm of temporal lobe	0	0	0	0	0	0	0	0	0	0	0	0	2	0	0	0	0	0
46905	Malignant neoplasm of coccygeal body	0	0	0	0	0	0	0	0	0	0	0	0	2	0	0	0	0	0
46939	Malignant neoplasm of cervical vertebra	0	0	0	0	0	0	0	0	0	0	0	0	2	0	0	0	0	0
47094	Malignant melanoma of eyebrow	0	0	0	0	0	0	0	0	0	0	0	0	2	0	0	0	0	0
47205	MALIGNANT OVERLAPPING LESION OF TONGUE	0	0	0	0	0	0	0	0	0	0	0	0	2	0	0	0	0	0
47252	Malignant melanoma of other and unspecified parts of face	0	0	0	0	0	0	0	0	0	0	0	0	2	0	0	0	0	0
47286	Malignant neoplasm of thorax	0	0	0	0	0	0	0	0	0	0	0	0	2	0	0	0	0	0
47556	Malignant neoplasm of temporal lobe NOS	0	0	0	0	0	0	0	0	0	0	0	0	2	0	0	0	0	0
47633	[X]Malig neopl, overlap lesion brain & other part of CNS	0	0	0	0	0	0	0	0	0	0	0	0	2	0	0	0	0	0
47668	MALIGNANT NEOPLASM OF TUNICA VAGINALIS	0	0	0	0	0	0	0	0	0	0	0	0	2	0	0	0	0	0

47669	[V]Personal history of malignant neoplasm of skin	0	0	0	0	0	0	0	0	0	0	0	0	2	0	0	0	0	0
47683	[V]Personal history of malignant neoplasm of kidney	0	0	0	0	0	0	0	0	0	0	0	0	2	0	0	0	0	0
47734	[M]Epithelioid mesothelioma, malignant	0	0	0	0	0	0	0	0	0	0	0	0	2	0	0	0	0	0
47767	MALIGNANT NEOPLASM OF SCROTUM	0	0	0	0	0	0	0	0	0	0	0	0	2	0	0	0	0	0
47801	Malignant neoplasm, overlapping lesion of bladder	0	0	0	0	0	0	0	0	0	0	0	0	2	0	0	0	0	0
47810	MALIGNANT NEOPLASM OF UNSPECIFIED SITE	0	0	0	0	0	0	0	0	0	0	0	0	2	0	0	0	0	0
47840	Malignant neoplasm of aortic body	0	0	0	0	0	0	0	0	0	0	0	0	2	0	0	0	0	0
47862	MALIGNANT NEOPLASM OF THYROID CARTILAGE	0	0	0	0	0	0	0	0	0	0	0	0	2	0	0	0	0	0
47899	Malignant neoplasm of greater vestibular (Bartholin's) gland	0	0	0	0	0	0	0	0	0	0	0	0	2	0	0	0	0	0
47920	[M]C CELL CARCINOMA	0	0	0	0	0	0	0	0	0	0	0	0	2	0	0	0	0	0
48048	[M]GIANT CELL AND SPINDLE CELL CARCINOMA	0	0	0	0	0	0	0	0	0	0	0	0	2	0	0	0	0	0
48073	MALIGNANT NEOPLASM OF BASAL GANGLIA	0	0	0	0	0	0	0	0	0	0	0	0	2	0	0	0	0	0
48085	[V]Personal history of malignant neoplasm of brain	0	0	0	0	0	0	0	0	0	0	0	0	2	0	0	0	0	0
48145	ANAEMIA IN OVARIAN CARCINOMA	0	0	0	0	0	0	0	0	0	0	0	0	2	0	0	0	0	0
48223	[M]Scirrhous adenocarcinoma	0	0	0	0	0	0	0	0	0	0	0	0	2	0	0	0	0	0
48231	MALIGNANT NEOPLASM OF OTHER SPECIFIED SITES OF COLON	0	0	0	0	0	0	0	0	0	0	0	0	2	0	0	0	0	0
48237	MALIGNANT NEOPLASM OF PREPYLORUS OF STOMACH	0	0	0	0	0	0	0	0	0	0	0	0	2	0	0	0	0	0
48275	[M]EMBRYONAL RHABDOMYOSARCOMA	0	0	0	0	0	0	0	0	0	0	0	0	2	0	0	0	0	0
48348	[M]PULMONARY BLASTOMA	0	0	0	0	0	0	0	0	0	0	0	0	2	0	0	0	0	0
48517	MALIGNANT NEOPLASM OF SOFT TISSUE OF NECK	0	0	0	0	0	0	0	0	0	0	0	0	2	0	0	0	0	0
48519	MALIGNANT NEOPLASM OF JUNCTIONAL REGION OF EPIGLOTTIS	0	0	0	0	0	0	0	0	0	0	0	0	2	0	0	0	0	0
48537	MALIGNANT NEOPLASM OF OTHER SPECIFIED SITES OF PANCREAS	0	0	0	0	0	0	0	0	0	0	0	0	2	0	0	0	0	0
48743	Malignant neoplasm of body of penis	0	0	0	0	0	0	0	0	0	0	0	0	2	0	0	0	0	0
48808	[V]Personal history of malignant neoplasm of testis	0	0	0	0	0	0	0	0	0	0	0	0	2	0	0	0	0	0

48809	MALIGNANT NEOPLASM OF MALE BREAST NOS	0	0	0	0	0	0	0	0	0	0	0	0	2	0	0	0	0	0
48820	MALIGNANT NEOPLASM OF ENDOCERVIX	0	0	0	0	0	0	0	0	0	0	0	0	2	0	0	0	0	0
48952	[M]Retinoblastoma NOS	0	0	0	0	0	0	0	0	0	0	0	0	2	0	0	0	0	0
49023	[M]Endothelial bone sarcoma	0	0	0	0	0	0	0	0	0	0	0	0	2	0	0	0	0	0
49054	MALIGNANT NEOPLASM OF SCAPULA	0	0	0	0	0	0	0	0	0	0	0	0	2	0	0	0	0	0
49132	Malignant neoplasm of medulla oblongata	0	0	0	0	0	0	0	0	0	0	0	0	2	0	0	0	0	0
49148	Malignant neoplasm	0	0	0	0	0	0	0	0	0	0	0	0	2	0	0	0	0	0
49186	[M]Oligodendroglioma, anaplastic type	0	0	0	0	0	0	0	0	0	0	0	0	2	0	0	0	0	0
49289	[V]Personal history of malig neop of trachea/bronchus/lung	0	0	0	0	0	0	0	0	0	0	0	0	2	0	0	0	0	0
49292	[X]Malignant neoplsm/ill-defin sites within digestive system	0	0	0	0	0	0	0	0	0	0	0	0	2	0	0	0	0	0
49360	MALIGNANT NEOPLASM OF LOWER GUM	0	0	0	0	0	0	0	0	0	0	0	0	2	0	0	0	0	0
49400	MALIGNANT NEOPLASM OF ENDOMETRIUM	0	0	0	0	0	0	0	0	0	0	0	0	2	0	0	0	0	0
49403	MALIGNANT NEOPLASM OF SKIN OF CHIN	0	0	0	0	0	0	0	0	0	0	0	0	2	0	0	0	0	0
49447	[V]Personal history of malignant neoplasm of stomach	0	0	0	0	0	0	0	0	0	0	0	0	2	0	0	0	0	0
49463	MALIGNANT NEOPLASM OF TARSUS OF EYELID	0	0	0	0	0	0	0	0	0	0	0	0	2	0	0	0	0	0
49482	Myopathy due to malignant disease	0	0	0	0	0	0	0	0	0	0	0	0	2	0	0	0	0	0
49491	MALIGNANT NEOPLASM OF STERNUM	0	0	0	0	0	0	0	0	0	0	0	0	2	0	0	0	0	0
49525	Kaposi's sarcoma, unspecified	0	0	0	0	0	0	0	0	0	0	0	0	2	0	0	0	0	0
49629	[M]GASTRINOMA, MALIGNANT	0	0	0	0	0	0	0	0	0	0	0	0	2	0	0	0	0	0
49701	Malignant neoplasm of vertebral column NOS	0	0	0	0	0	0	0	0	0	0	0	0	2	0	0	0	0	0
49714	Malignant neoplasm of spinal meninges	0	0	0	0	0	0	0	0	0	0	0	0	2	0	0	0	0	0
49758	MALIGNANT NEOPLASM OF OTHER SITES LIP, ORAL CAVITY, PHARYNX	0	0	0	0	0	0	0	0	0	0	0	0	2	0	0	0	0	0
49811	[M]MESODERMAL MIXED TUMOUR	0	0	0	0	0	0	0	0	0	0	0	0	2	0	0	0	0	0
49814	Malignant melanoma of axilla	0	0	0	0	0	0	0	0	0	0	0	0	2	0	0	0	0	0

49828	Malignant neoplasm of fallopian tube	0	0	0	0	0	0	0	0	0	0	0	0	2	0	0	0	0	0
49862	[M]Osteoblastic sarcoma	0	0	0	0	0	0	0	0	0	0	0	0	2	0	0	0	0	0
49875	Malignant neoplasm of meninges, unspecified	0	0	0	0	0	0	0	0	0	0	0	0	2	0	0	0	0	0
49900	[M]Klatskin's tumour	0	0	0	0	0	0	0	0	0	0	0	0	2	0	0	0	0	0
50035	MALIGNANT NEOPLASM OF AORTIC BODY AND OTHER PARAGANGLIA	0	0	0	0	0	0	0	0	0	0	0	0	2	0	0	0	0	0
50140	[M]Cribriform carcinoma	0	0	0	0	0	0	0	0	0	0	0	0	2	0	0	0	0	0
50151	[M]Pineoblastoma	0	0	0	0	0	0	0	0	0	0	0	0	2	0	0	0	0	0
50152	MALIGNANT SACRAL TERATOMA	0	0	0	0	0	0	0	0	0	0	0	0	2	0	0	0	0	0
50222	Malignant neoplasm of connective and soft tissue of shoulder	0	0	0	0	0	0	0	0	0	0	0	0	2	0	0	0	0	0
50235	[M]ASTROBLASTOMA	0	0	0	0	0	0	0	0	0	0	0	0	2	0	0	0	0	0
50285	MALIGNANT NEOPLASM OF ENDOCERVIX NOS	0	0	0	0	0	0	0	0	0	0	0	0	2	0	0	0	0	0
50289	MALIGNANT NEOPLASM OF HEART NOS	0	0	0	0	0	0	0	0	0	0	0	0	2	0	0	0	0	0
50290	KAPOSI'S SARCOMA OF LYMPH NODES	0	0	0	0	0	0	0	0	0	0	0	0	2	0	0	0	0	0
50292	[X]Malignant neoplasm of mediastinum	0	0	0	0	0	0	0	0	0	0	0	0	2	0	0	0	0	0
50296	MALIGNANT NEOPLASM OF UPPER LIP, LIPSTICK AREA	0	0	0	0	0	0	0	0	0	0	0	0	2	0	0	0	0	0
50297	MALIGNANT NEOPLASM OF EXOCERVIX	0	0	0	0	0	0	0	0	0	0	0	0	2	0	0	0	0	0
50298	MALIGNANT NEOPLASM OF ORBITAL BONE	0	0	0	0	0	0	0	0	0	0	0	0	2	0	0	0	0	0
50299	MALIGNANT NEOPLASM OF ZYGOMATIC BONE	0	0	0	0	0	0	0	0	0	0	0	0	2	0	0	0	0	0
50379	[M]SYNOVIAL SARCOMA NOS	0	0	0	0	0	0	0	0	0	0	0	0	2	0	0	0	0	0
50402	Malignant neoplasm of fibula	0	0	0	0	0	0	0	0	0	0	0	0	2	0	0	0	0	0
50475	MALIGNANT NEOPLASM OF MAJOR SALIVARY GLAND NOS	0	0	0	0	0	0	0	0	0	0	0	0	2	0	0	0	0	0
50505	Malignant melanoma of shoulder	0	0	0	0	0	0	0	0	0	0	0	0	2	0	0	0	0	0
50579	Malignant neoplasm, overlapping lesion of larynx	0	0	0	0	0	0	0	0	0	0	0	0	2	0	0	0	0	0
50605	[M]GLOMANGIOSARCOMA	0	0	0	0	0	0	0	0	0	0	0	0	2	0	0	0	0	0

50681	MALIGNANT NEOPLASM OF PREPUCE (FORESKIN)	0	0	0	0	0	0	0	0	0	0	0	0	2	0	0	0	0	0
50777	MALIGNANT NEOPLASM,OVERLAP LESION PERIPH NERVE & AUTON NS	0	0	0	0	0	0	0	0	0	0	0	0	2	0	0	0	0	0
50789	MALIGNANT NEOPLASM OF UPPER THIRD OF OESOPHAGUS	0	0	0	0	0	0	0	0	0	0	0	0	2	0	0	0	0	0
50859	[M]Giant cell bone sarcoma	0	0	0	0	0	0	0	0	0	0	0	0	2	0	0	0	0	0
50898	Malignant neoplasm of omentum	0	0	0	0	0	0	0	0	0	0	0	0	2	0	0	0	0	0
50946	[M]MEDULLARY CARCINOMA WITH AMYLOID STROMA	0	0	0	0	0	0	0	0	0	0	0	0	2	0	0	0	0	0
50974	MALIGNANT NEOPLASM RECTUM,RECTOSIGMOID JUNCTION AND ANUS NOS	0	0	0	0	0	0	0	0	0	0	0	0	2	0	0	0	0	0
51001	[V]Personal history of malignant neoplasm of oesophagus	0	0	0	0	0	0	0	0	0	0	0	0	2	0	0	0	0	0
51115	Malignant neoplasm of spinal cord	0	0	0	0	0	0	0	0	0	0	0	0	2	0	0	0	0	0
51209	MALIGNANT MELANOMA OF CHEST WALL	0	0	0	0	0	0	0	0	0	0	0	0	2	0	0	0	0	0
51237	Malignant neoplasm of rib	0	0	0	0	0	0	0	0	0	0	0	0	2	0	0	0	0	0
51255	MALIGNANT NEOPLASM OF DIGESTIVE TRACT AND PERITONEUM NOS	0	0	0	0	0	0	0	0	0	0	0	0	2	0	0	0	0	0
51352	Malignant neoplasms of independent (primary) multiple sites	0	0	0	0	0	0	0	0	0	0	0	0	2	0	0	0	0	0
51353	[M]Malignant melanoma	0	0	0	0	0	0	0	0	0	0	0	0	2	0	0	0	0	0
51656	[M]Mucinous cystadenocarcinoma NOS	0	0	0	0	0	0	0	0	0	0	0	0	2	0	0	0	0	0
51690	MALIGNANT NEOPLASM, OVERLAPPING LESION OF STOMACH	0	0	0	0	0	0	0	0	0	0	0	0	2	0	0	0	0	0
51786	MALIGNANT NEOPLASM OF SUBMANDIBULAR GLAND	0	0	0	0	0	0	0	0	0	0	0	0	2	0	0	0	0	0
51795	Malignant neoplasm of glomus jugulare	0	0	0	0	0	0	0	0	0	0	0	0	2	0	0	0	0	0
51818	MALIGNANT NEOPLASM OF JAW NOS	0	0	0	0	0	0	0	0	0	0	0	0	2	0	0	0	0	0
51873	Malignant melanoma of thigh	0	0	0	0	0	0	0	0	0	0	0	0	2	0	0	0	0	0
51878	[M]AESTHESIONEUROBLASTOMA	0	0	0	0	0	0	0	0	0	0	0	0	2	0	0	0	0	0
51921	MALIGNANT NEOPLASM OF PUBIS	0	0	0	0	0	0	0	0	0	0	0	0	2	0	0	0	0	0
51926	MALIGNANT NEOPLASM OF FAUCIAL PILLAR	0	0	0	0	0	0	0	0	0	0	0	0	2	0	0	0	0	0

51965	Malignant neoplasm of connective and soft tissue of pelvis	0	0	0	0	0	0	0	0	0	0	0	0	2	0	0	0	0	0
52029	[X]Malignant neoplasm without specification of site	0	0	0	0	0	0	0	0	0	0	0	0	2	0	0	0	0	0
52141	[V]Personal history of malignant neoplasm of ovary	0	0	0	0	0	0	0	0	0	0	0	0	2	0	0	0	0	0
52263	[M]SEROUS CYSTADENOMA, BORDERLINE MALIGNANCY	0	0	0	0	0	0	0	0	0	0	0	0	2	0	0	0	0	0
52266	[M]GRAWITZ TUMOUR	0	0	0	0	0	0	0	0	0	0	0	0	2	0	0	0	0	0
52316	MALIGNANT NEOPLASM OF PELVIS	0	0	0	0	0	0	0	0	0	0	0	0	2	0	0	0	0	0
52326	[M]Adenocarcinoma in adenomatous polyp	0	0	0	0	0	0	0	0	0	0	0	0	2	0	0	0	0	0
52493	[M]TERATOBLASTOMA, MALIGNANT	0	0	0	0	0	0	0	0	0	0	0	0	2	0	0	0	0	0
52511	Malignant neoplasm of cerebral ventricles	0	0	0	0	0	0	0	0	0	0	0	0	2	0	0	0	0	0
52537	MALIGNANT NEOPLASM OF HEPATIC DUCT	0	0	0	0	0	0	0	0	0	0	0	0	2	0	0	0	0	0
52570	MALIGNANT NEOPLASM, OVERLAPPING LESION OF PENIS	0	0	0	0	0	0	0	0	0	0	0	0	2	0	0	0	0	0
52594	MALIGNANT NEOPLASM OF GENITOURINARY ORGAN NOS	0	0	0	0	0	0	0	0	0	0	0	0	2	0	0	0	0	0
52684	[M]Mesenchymal chondrosarcoma	0	0	0	0	0	0	0	0	0	0	0	0	2	0	0	0	0	0
52751	[M]EPENDYMOMA, ANAPLASTIC TYPE	0	0	0	0	0	0	0	0	0	0	0	0	2	0	0	0	0	0
53103	Malignant neoplasm of endocervical gland	0	0	0	0	0	0	0	0	0	0	0	0	2	0	0	0	0	0
53129	[M]Oncytic adenocarcinoma	0	0	0	0	0	0	0	0	0	0	0	0	2	0	0	0	0	0
53369	MALIGNANT MELANOMA OF GREAT TOE	0	0	0	0	0	0	0	0	0	0	0	0	2	0	0	0	0	0
53504	MALIG NEOPL, OVERLAP LESION BRAIN & OTHER PART OF CNS	0	0	0	0	0	0	0	0	0	0	0	0	2	0	0	0	0	0
53515	Malignant neoplasm skin of ear and external auricular canal	0	0	0	0	0	0	0	0	0	0	0	0	2	0	0	0	0	0
53591	MALIGNANT NEOPLASM OF OTHER SPECIFIED PART OF OESOPHAGUS	0	0	0	0	0	0	0	0	0	0	0	0	2	0	0	0	0	0
53594	MALIGNANT NEOPLASM OF ETHMOID BONE	0	0	0	0	0	0	0	0	0	0	0	0	2	0	0	0	0	0
53599	MALIGNANT NEOPLASM OF FRONTAL BONE	0	0	0	0	0	0	0	0	0	0	0	0	2	0	0	0	0	0
53629	Malignant melanoma of buttock	0	0	0	0	0	0	0	0	0	0	0	0	2	0	0	0	0	0
53884	MALIGNANT NEOPLASM TONSIL NOS	0	0	0	0	0	0	0	0	0	0	0	0	2	0	0	0	0	0

53910	Malignant neoplasm of clitoris	0	0	0	0	0	0	0	0	0	0	0	0	2	0	0	0	0	0
53989	Malig neop connective and soft tissue upper limb/shoulder	0	0	0	0	0	0	0	0	0	0	0	0	2	0	0	0	0	0
54103	Malignant neoplasm gallbladder and extrahepatic bile ducts	0	0	0	0	0	0	0	0	0	0	0	0	2	0	0	0	0	0
54133	Malignant neoplasm of cerebrum NOS	0	0	0	0	0	0	0	0	0	0	0	0	2	0	0	0	0	0
54134	Malignant neoplasm of middle lobe	0	0	0	0	0	0	0	0	0	0	0	0	2	0	0	0	0	0
54171	Malignant neoplasm of middle third of oesophagus	0	0	0	0	0	0	0	0	0	0	0	0	2	0	0	0	0	0
54184	MALIGNANT NEOPLASM OF RENAL PELVIS NOS	0	0	0	0	0	0	0	0	0	0	0	0	2	0	0	0	0	0
54186	MALIGNANT NEOPLASM OF DIAPHRAGM	0	0	0	0	0	0	0	0	0	0	0	0	2	0	0	0	0	0
54202	Malignant neoplasm of other site of male breast	0	0	0	0	0	0	0	0	0	0	0	0	2	0	0	0	0	0
54222	Malignant neoplasm of connective and soft tissue of foot	0	0	0	0	0	0	0	0	0	0	0	0	2	0	0	0	0	0
54234	Malignant neoplasm of scalp and skin of neck	0	0	0	0	0	0	0	0	0	0	0	0	2	0	0	0	0	0
54267	MALIGNANT NEOPLASM OF UNSPECIFIED SITE NOS	0	0	0	0	0	0	0	0	0	0	0	0	2	0	0	0	0	0
54276	[M]PSEUDOSARCOMATOUS CARCINOMA	0	0	0	0	0	0	0	0	0	0	0	0	2	0	0	0	0	0
54284	[M]NEUROEPITHELIOMATOUS NEOPLASMS	0	0	0	0	0	0	0	0	0	0	0	0	2	0	0	0	0	0
54305	MALIGNANT MELANOMA OF KNEE	0	0	0	0	0	0	0	0	0	0	0	0	2	0	0	0	0	0
54336	Cancer hospital treatment completed	0	0	0	0	0	0	0	0	0	0	0	0	2	0	0	0	0	0
54352	MALIGNANT NEOPLASM OF SKIN OF HAND	0	0	0	0	0	0	0	0	0	0	0	0	2	0	0	0	0	0
54493	MALIGNANT NEOPLASM OF XIPHOID PROCESS	0	0	0	0	0	0	0	0	0	0	0	0	2	0	0	0	0	0
54494	MALIGNANT NEOPLASM OF NIPPLE AND AREOLA OF MALE BREAST	0	0	0	0	0	0	0	0	0	0	0	0	2	0	0	0	0	0
54613	MALIGNANT NEOPLASM OF TYMPANIC ANTRUM	0	0	0	0	0	0	0	0	0	0	0	0	2	0	0	0	0	0
54627	[M]Choriocarcinoma combined with teratoma	0	0	0	0	0	0	0	0	0	0	0	0	2	0	0	0	0	0
54631	Malignant neoplasm of pelvic bones, sacrum and coccyx	0	0	0	0	0	0	0	0	0	0	0	0	2	0	0	0	0	0
54632	MALIGNANT MELANOMA OF EYELID INCLUDING CANTHUS	0	0	0	0	0	0	0	0	0	0	0	0	2	0	0	0	0	0
54636	MALIGNANT NEOPLASM OF ETHMOID SINUS	0	0	0	0	0	0	0	0	0	0	0	0	2	0	0	0	0	0

54685	MALIGNANT MELANOMA OF UPPER ARM	0	0	0	0	0	0	0	0	0	0	0	0	2	0	0	0	0	0
54691	MALIGNANT NEOPLASM OF LUMBAR VERTEBRA	0	0	0	0	0	0	0	0	0	0	0	0	2	0	0	0	0	0
54747	MALIGNANT NEOPLASM OF PARIETAL BONE	0	0	0	0	0	0	0	0	0	0	0	0	2	0	0	0	0	0
54749	[M]PAPILLARY MUCINOUS CYSTADENOCARCINOMA	0	0	0	0	0	0	0	0	0	0	0	0	2	0	0	0	0	0
54956	MALIGNANT NEOPLASM OF EYE NOS	0	0	0	0	0	0	0	0	0	0	0	0	2	0	0	0	0	0
54965	MALIG NEOP CONNECTIVE AND SOFT TISSUE OF POPLITEAL SPACE	0	0	0	0	0	0	0	0	0	0	0	0	2	0	0	0	0	0
55015	MALIGNANT NEOPLASM OF MOUTH NOS	0	0	0	0	0	0	0	0	0	0	0	0	2	0	0	0	0	0
55019	MALIGNANT NEOPLASM OF OTHER SPECIFIED SITE OF STOMACH	0	0	0	0	0	0	0	0	0	0	0	0	2	0	0	0	0	0
55066	Malignant neoplasm of tonsillar pillar	0	0	0	0	0	0	0	0	0	0	0	0	2	0	0	0	0	0
55098	Malignant neoplasm of head NOS	0	0	0	0	0	0	0	0	0	0	0	0	2	0	0	0	0	0
55101	Malignant neoplasm of pelvis NOS	0	0	0	0	0	0	0	0	0	0	0	0	2	0	0	0	0	0
55246	MALIGNANT NEOPLASM OF ACCESSORY SINUS NOS	0	0	0	0	0	0	0	0	0	0	0	0	2	0	0	0	0	0
55268	[M]Myosarcoma	0	0	0	0	0	0	0	0	0	0	0	0	2	0	0	0	0	0
55292	MALIGNANT MELANOMA OF UPPER LIMB OR SHOULDER NOS	0	0	0	0	0	0	0	0	0	0	0	0	2	0	0	0	0	0
55374	MALIGNANT NEOPLASM OF EPIGLOTTIS NOS	0	0	0	0	0	0	0	0	0	0	0	0	2	0	0	0	0	0
55429	[M]MUCOID ADENOCARCIONOMA	0	0	0	0	0	0	0	0	0	0	0	0	2	0	0	0	0	0
55434	Malignant neoplasm of greater curve of stomach unspecified	0	0	0	0	0	0	0	0	0	0	0	0	2	0	0	0	0	0
55468	[M]MUCOCARCINOID TUMOUR, MALIGNANT	0	0	0	0	0	0	0	0	0	0	0	0	2	0	0	0	0	0
55550	Malignant neoplasm of upper eyelid	0	0	0	0	0	0	0	0	0	0	0	0	2	0	0	0	0	0
55588	[X]MALIGNANT NEOPLASM OF FEMALE GENITAL ORGAN, UNSPECIFIED	0	0	0	0	0	0	0	0	0	0	0	0	2	0	0	0	0	0
55595	Malignant neoplasm of sphenoid bone	0	0	0	0	0	0	0	0	0	0	0	0	2	0	0	0	0	0
55630	Malignant neoplasm of other specified site of nasopharynx	0	0	0	0	0	0	0	0	0	0	0	0	2	0	0	0	0	0
55658	[M]ORCHIOBLASTOMA	0	0	0	0	0	0	0	0	0	0	0	0	2	0	0	0	0	0
55659	Malig neop other site rectum, rectosigmoid junction and anus	0	0	0	0	0	0	0	0	0	0	0	0	2	0	0	0	0	0

55670	MALIGNANT NEOPLASM OF SKIN OF EYEBROW	0	0	0	0	0	0	0	0	0	0	0	0	2	0	0	0	0	0
55881	Malignant melanoma of scalp	0	0	0	0	0	0	0	0	0	0	0	0	2	0	0	0	0	0
55947	[M]PLEOMORPHIC LIPOSARCOMA	0	0	0	0	0	0	0	0	0	0	0	0	2	0	0	0	0	0
55953	Malignant neoplasm of occipital bone	0	0	0	0	0	0	0	0	0	0	0	0	2	0	0	0	0	0
56121	[X]Malignant neoplasm of skin	0	0	0	0	0	0	0	0	0	0	0	0	2	0	0	0	0	0
56355	Malignant neoplasm of lateral wall of oropharynx	0	0	0	0	0	0	0	0	0	0	0	0	2	0	0	0	0	0
56490	Malignant neoplasm of nervous system NOS	0	0	0	0	0	0	0	0	0	0	0	0	2	0	0	0	0	0
56513	MALIGNANT NEOPLASM OF FEMUR	0	0	0	0	0	0	0	0	0	0	0	0	2	0	0	0	0	0
56600	[M]Epidermoid carcinoma NOS	0	0	0	0	0	0	0	0	0	0	0	0	2	0	0	0	0	0
56676	[M]Myxoid liposarcoma	0	0	0	0	0	0	0	0	0	0	0	0	2	0	0	0	0	0
56709	MALIGNANT NEOPLASM OF OTHER SITES OF FLOOR OF MOUTH	0	0	0	0	0	0	0	0	0	0	0	0	2	0	0	0	0	0
56715	Malignant neoplasm of other site of female breast	0	0	0	0	0	0	0	0	0	0	0	0	2	0	0	0	0	0
56718	Malignant neoplasm of eyeball NOS	0	0	0	0	0	0	0	0	0	0	0	0	2	0	0	0	0	0
56794	[M]Adenocarcinoid tumour	0	0	0	0	0	0	0	0	0	0	0	0	2	0	0	0	0	0
56918	MALIGNANT NEOPLASM OTHER SPEC DIGESTIVE TRACT AND PERITONEUM	0	0	0	0	0	0	0	0	0	0	0	0	2	0	0	0	0	0
56925	[X]Malignant melanoma of other+unspecified parts of face	0	0	0	0	0	0	0	0	0	0	0	0	2	0	0	0	0	0
56954	Malignant neoplasm of skin of knee	0	0	0	0	0	0	0	0	0	0	0	0	2	0	0	0	0	0
57047	Malignant neoplasm of carotid body	0	0	0	0	0	0	0	0	0	0	0	0	2	0	0	0	0	0
57084	[M]SEMINOMA, ANAPLASTIC TYPE	0	0	0	0	0	0	0	0	0	0	0	0	2	0	0	0	0	0
57087	[M]EMBRYONAL TERATOMA	0	0	0	0	0	0	0	0	0	0	0	0	2	0	0	0	0	0
57184	[X]Oth malignant neoplasm/skin of oth+unspecfd parts of face	0	0	0	0	0	0	0	0	0	0	0	0	2	0	0	0	0	0
57191	[X]Malignant neoplasm/other specified male genital organs	0	0	0	0	0	0	0	0	0	0	0	0	2	0	0	0	0	0
57235	Malignant neoplasm of endocervical canal	0	0	0	0	0	0	0	0	0	0	0	0	2	0	0	0	0	0
57248	Malignant neoplasm aryepiglottic fold, hypopharyngeal aspect	0	0	0	0	0	0	0	0	0	0	0	0	2	0	0	0	0	0

57260	MALIGNANT MELANOMA OF EAR AND EXTERNAL AURICULAR CANAL	0	0	0	0	0	0	0	0	0	0	0	0	2	0	0	0	0	0
57336	[M]Epithelioma, malignant	0	0	0	0	0	0	0	0	0	0	0	0	2	0	0	0	0	0
57442	Malignant neoplasm of skin of lower limb and hip	0	0	0	0	0	0	0	0	0	0	0	0	2	0	0	0	0	0
57446	MALIGNANT NEOPLASM OF SKIN OF TRUNK, EXCLUDING SCROTUM	0	0	0	0	0	0	0	0	0	0	0	0	2	0	0	0	0	0
57471	MALIG NEOP OF CONNECTIVE AND SOFT TISSUE TRUNK UNSPECIFIED	0	0	0	0	0	0	0	0	0	0	0	0	2	0	0	0	0	0
57482	Malignant neoplasm of connective and soft tissue of fore-arm	0	0	0	0	0	0	0	0	0	0	0	0	2	0	0	0	0	0
57505	[M]Pleomorphic rhabdomyosarcoma	0	0	0	0	0	0	0	0	0	0	0	0	2	0	0	0	0	0
57513	[M]Epidermoid carcinoma	0	0	0	0	0	0	0	0	0	0	0	0	2	0	0	0	0	0
57551	MYASTHENIC SYNDROME DUE TO OTHER MALIGNANCY	0	0	0	0	0	0	0	0	0	0	0	0	2	0	0	0	0	0
57677	[M]HEPATOBLASTOMA	0	0	0	0	0	0	0	0	0	0	0	0	2	0	0	0	0	0
57680	[M]Spinous cell carcinoma	0	0	0	0	0	0	0	0	0	0	0	0	2	0	0	0	0	0
57719	MALIGNANT NEOPLASM OF SQUAMOCOLUMNAR JUNCTION OF CERVIX	0	0	0	0	0	0	0	0	0	0	0	0	2	0	0	0	0	0
57727	[V]Personal history of malignant neoplasm of large intestine	0	0	0	0	0	0	0	0	0	0	0	0	2	0	0	0	0	0
57729	[M]Lymphangiosarcoma	0	0	0	0	0	0	0	0	0	0	0	0	2	0	0	0	0	0
57756	[X]Malignant neoplasm/other specified female genital organs	0	0	0	0	0	0	0	0	0	0	0	0	2	0	0	0	0	0
57796	[M]SYNOVIAL SARCOMA, BIPHASIC TYPE	0	0	0	0	0	0	0	0	0	0	0	0	2	0	0	0	0	0
57802	[M]ALVEOLAR ADENOCARCINOMA	0	0	0	0	0	0	0	0	0	0	0	0	2	0	0	0	0	0
57854	Malignant neoplasm of inguinal region NOS	0	0	0	0	0	0	0	0	0	0	0	0	2	0	0	0	0	0
57988	Malignant neoplasm of carpal bone - scaphoid	0	0	0	0	0	0	0	0	0	0	0	0	2	0	0	0	0	0
58061	MALIGNANT NEOPLASM OF LABIA MINORA	0	0	0	0	0	0	0	0	0	0	0	0	2	0	0	0	0	0
58088	MALIGNANT NEOPLASM OF INTRAHEPATIC GALL DUCT	0	0	0	0	0	0	0	0	0	0	0	0	2	0	0	0	0	0
58094	MALIGNANT NEOPLASM, OVERLAPPING LESION OF CERVIX UTERI	0	0	0	0	0	0	0	0	0	0	0	0	2	0	0	0	0	0
58121	Malignant neoplasm of anterior 2/3 of tongue unspecified	0	0	0	0	0	0	0	0	0	0	0	0	2	0	0	0	0	0
58131	[M]COMEDOCARCINOMA NOS	0	0	0	0	0	0	0	0	0	0	0	0	2	0	0	0	0	0

58167	UNDER CARE CANCER PRIMARY HEALTHCARE MULTIDISCIPLINARY TEAM	0	0	0	0	0	0	0	0	0	0	0	0	2	0	0	0	0	0
58177	[V]Personal history of malignant neoplasm of liver	0	0	0	0	0	0	0	0	0	0	0	0	2	0	0	0	0	0
58601	Malignant neoplasm of skin of thigh	0	0	0	0	0	0	0	0	0	0	0	0	2	0	0	0	0	0
58798	[M]TRANSITIONAL CELL CARCINOMA, SPINDLE CELL TYPE	0	0	0	0	0	0	0	0	0	0	0	0	2	0	0	0	0	0
58835	[M]Desmoplastic melanoma	0	0	0	0	0	0	0	0	0	0	0	0	2	0	0	0	0	0
58836	Malig neop of connective and soft tissue of pelvis NOS	0	0	0	0	0	0	0	0	0	0	0	0	2	0	0	0	0	0
58837	[M]SMALL CELL SARCOMA	0	0	0	0	0	0	0	0	0	0	0	0	2	0	0	0	0	0
58902	[M]Olfactory neurogenic tumour	0	0	0	0	0	0	0	0	0	0	0	0	2	0	0	0	0	0
58903	Malignant neoplasm of head	0	0	0	0	0	0	0	0	0	0	0	0	2	0	0	0	0	0
58949	MALIGNANT NEOPLASM OF PHALANGES OF FOOT	0	0	0	0	0	0	0	0	0	0	0	0	2	0	0	0	0	0
58958	Malignant melanoma of temple	0	0	0	0	0	0	0	0	0	0	0	0	2	0	0	0	0	0
58973	[X]MALIGNANT NEOPLASM OF LIP, ORAL CAVITY AND PHARYNX	0	0	0	0	0	0	0	0	0	0	0	0	2	0	0	0	0	0
59004	Malignant neoplasm of lateral wall of nasopharynx	0	0	0	0	0	0	0	0	0	0	0	0	2	0	0	0	0	0
59036	MALIGNANT NEOPLASM OF BONES OF SKULL AND FACE	0	0	0	0	0	0	0	0	0	0	0	0	2	0	0	0	0	0
59041	MALIGNANT NEOPLASM OF CILIARY BODY	0	0	0	0	0	0	0	0	0	0	0	0	2	0	0	0	0	0
59061	Malignant melanoma of auricle (ear)	0	0	0	0	0	0	0	0	0	0	0	0	2	0	0	0	0	0
59076	SPUTUM: MALIGNANT CELLS	0	0	0	0	0	0	0	0	0	0	0	0	2	0	0	0	0	0
59092	MALIGNANT NEOPLASM OF PYLORUS OF STOMACH NOS	0	0	0	0	0	0	0	0	0	0	0	0	2	0	0	0	0	0
59097	Malignant neoplasm of lower uterine segment	0	0	0	0	0	0	0	0	0	0	0	0	2	0	0	0	0	0
59143	[M]SQUAMOUS CELL CARCINOMA, LARGE CELL, NON-KERATINISING	0	0	0	0	0	0	0	0	0	0	0	0	2	0	0	0	0	0
59152	Malignant neoplasm of connective and soft tissue of perineum	0	0	0	0	0	0	0	0	0	0	0	0	2	0	0	0	0	0
59170	Malignant neoplasm of corpus callosum	0	0	0	0	0	0	0	0	0	0	0	0	2	0	0	0	0	0
59223	MALIGNANT NEOPLASM OF ISCHIUM	0	0	0	0	0	0	0	0	0	0	0	0	2	0	0	0	0	0
59240	[M]CARCINOMA, DIFFUSE TYPE	0	0	0	0	0	0	0	0	0	0	0	0	2	0	0	0	0	0

59251	[M]Cystosarcoma phyllodes, malignant	0	0	0	0	0	0	0	0	0	0	0	0	2	0	0	0	0	0
59284	[M]MUCOUS ADENOCARCINOMA	0	0	0	0	0	0	0	0	0	0	0	0	2	0	0	0	0	0
59286	Malignant neoplasm of overlapping lesion of urinary organs	0	0	0	0	0	0	0	0	0	0	0	0	2	0	0	0	0	0
59310	[M]Osteochondrosarcoma	0	0	0	0	0	0	0	0	0	0	0	0	2	0	0	0	0	0
59362	MALIGNANT NEOPLASM OF LABIA MAJORA NOS	0	0	0	0	0	0	0	0	0	0	0	0	2	0	0	0	0	0
59381	MALIGNANT NEOPLASM OF IRIS	0	0	0	0	0	0	0	0	0	0	0	0	2	0	0	0	0	0
59382	MALIGNANT NEOPLASM OF SOFT TISSUE OF HEAD	0	0	0	0	0	0	0	0	0	0	0	0	2	0	0	0	0	0
59388	Malignant neoplasm of mesocaecum	0	0	0	0	0	0	0	0	0	0	0	0	2	0	0	0	0	0
59415	[M]Thymoma, malignant	0	0	0	0	0	0	0	0	0	0	0	0	2	0	0	0	0	0
59520	MALIGNANT NEOPLASM OF MALAR BONE	0	0	0	0	0	0	0	0	0	0	0	0	2	0	0	0	0	0
59651	[M]MIXED TYPE LIPOSARCOMA	0	0	0	0	0	0	0	0	0	0	0	0	2	0	0	0	0	0
59718	MALIG NEOP PITUITARY GLAND OR CRANIOPHARYNGEAL DUCT NOS	0	0	0	0	0	0	0	0	0	0	0	0	2	0	0	0	0	0
59823	Malignant neoplasm pituitary gland and craniopharyngeal duct	0	0	0	0	0	0	0	0	0	0	0	0	2	0	0	0	0	0
59831	Malignant neoplasm of nipple or areola of female breast NOS	0	0	0	0	0	0	0	0	0	0	0	0	2	0	0	0	0	0
59918	[M]Follicular adenocarcinoma, well differentiated type	0	0	0	0	0	0	0	0	0	0	0	0	2	0	0	0	0	0
59919	[M]Multicentric basal cell carcinoma	0	0	0	0	0	0	0	0	0	0	0	0	2	0	0	0	0	0
60035	Malignant neoplasm of cartilage of ear	0	0	0	0	0	0	0	0	0	0	0	0	2	0	0	0	0	0
60045	[M]Tubular adenocarcinoma	0	0	0	0	0	0	0	0	0	0	0	0	2	0	0	0	0	0
60052	Malignant neoplasm of specified site NOS	0	0	0	0	0	0	0	0	0	0	0	0	2	0	0	0	0	0
60127	[M]MYXOLIPOSARCOMA	0	0	0	0	0	0	0	0	0	0	0	0	2	0	0	0	0	0
60162	[X]Malignant neoplasm overlapping lesion of skin	0	0	0	0	0	0	0	0	0	0	0	0	2	0	0	0	0	0
60247	MALIG NEOP OF CONNECTIVE AND SOFT TISSUE OF ABDOMEN NOS	0	0	0	0	0	0	0	0	0	0	0	0	2	0	0	0	0	0
60312	Malignant neoplasm other gallbladder/extrahepatic bile duct	0	0	0	0	0	0	0	0	0	0	0	0	2	0	0	0	0	0
60347	[M]LEPTOMENINGEAL SARCOMA	0	0	0	0	0	0	0	0	0	0	0	0	2	0	0	0	0	0

60403	MALIGNANT NEOPLASM OF COSTAL CARTILAGE	0	0	0	0	0	0	0	0	0	0	0	0	2	0	0	0	0	0
60526	MALIGNANT NEOPLASM OF SKIN OF UPPER LIMB OR SHOULDER NOS	0	0	0	0	0	0	0	0	0	0	0	0	2	0	0	0	0	0
60631	[M]Osteosarcoma in Paget's disease of bone	0	0	0	0	0	0	0	0	0	0	0	0	2	0	0	0	0	0
60756	[M]CYLINDROID BRONCHIAL ADENOMA	0	0	0	0	0	0	0	0	0	0	0	0	2	0	0	0	0	0
60772	Malignant neoplasm of vagina NOS	0	0	0	0	0	0	0	0	0	0	0	0	2	0	0	0	0	0
60775	[M]ADRENAL CORTICAL CARCINOMA	0	0	0	0	0	0	0	0	0	0	0	0	2	0	0	0	0	0
60803	[M]Paget's disease, breast	0	0	0	0	0	0	0	0	0	0	0	0	2	0	0	0	0	0
61064	MALIGNANT NEOPLASM OF MEDIASTINUM, PART UNSPECIFIED	0	0	0	0	0	0	0	0	0	0	0	0	2	0	0	0	0	0
61082	[M]Pneumoblastoma	0	0	0	0	0	0	0	0	0	0	0	0	2	0	0	0	0	0
61194	Malignant neoplasm of skin of lower limb or hip NOS	0	0	0	0	0	0	0	0	0	0	0	0	2	0	0	0	0	0
61246	Malignant melanoma of heel	0	0	0	0	0	0	0	0	0	0	0	0	2	0	0	0	0	0
61390	Malignant neoplasm of adrenal cortex	0	0	0	0	0	0	0	0	0	0	0	0	2	0	0	0	0	0
61399	MALIGNANT NEOPLASM OF CEREBRAL CORTEX	0	0	0	0	0	0	0	0	0	0	0	0	2	0	0	0	0	0
61467	[M]FOLLICULAR ADENOCARCINOMA, TRABECULAR TYPE	0	0	0	0	0	0	0	0	0	0	0	0	2	0	0	0	0	0
61510	Malignant neoplasm of palatoglossal arch	0	0	0	0	0	0	0	0	0	0	0	0	2	0	0	0	0	0
61542	[M]MALIGNANT TERATOMA, UNDIFFERENTIATED TYPE	0	0	0	0	0	0	0	0	0	0	0	0	2	0	0	0	0	0
61555	Malignant neoplasm of retroperitoneum NOS	0	0	0	0	0	0	0	0	0	0	0	0	2	0	0	0	0	0
61588	[M]SIGNET RING CELL CARCINOMA	0	0	0	0	0	0	0	0	0	0	0	0	2	0	0	0	0	0
61643	Malignant neoplasm of intrahepatic bile ducts NOS	0	0	0	0	0	0	0	0	0	0	0	0	2	0	0	0	0	0
61655	[V]Personal history of malignant neoplasm - accessory sinus	0	0	0	0	0	0	0	0	0	0	0	0	2	0	0	0	0	0
61692	Malignant neoplasm of lip unspecified	0	0	0	0	0	0	0	0	0	0	0	0	2	0	0	0	0	0
61695	MALIGNANT NEOPLASM OF CERVICAL OESOPHAGUS	0	0	0	0	0	0	0	0	0	0	0	0	2	0	0	0	0	0
61716	MALIGNANT NEOPLASM OF PERIPHERAL NERVE,UPP LIMB,INCL SHOULD	0	0	0	0	0	0	0	0	0	0	0	0	2	0	0	0	0	0
61741	Malignant neoplasm of humerus	0	0	0	0	0	0	0	0	0	0	0	0	2	0	0	0	0	0

61783	[M]JUVENILE ASTROCYTOMA	0	0	0	0	0	0	0	0	0	0	0	0	2	0	0	0	0	0
61984	[M]Spheroidal cell carcinoma	0	0	0	0	0	0	0	0	0	0	0	0	2	0	0	0	0	0
62080	MALIGNANT NEOPLASM OF SKIN OF EXTERNAL AUDITORY MEATUS	0	0	0	0	0	0	0	0	0	0	0	0	2	0	0	0	0	0
62088	[M]MALIGNANT MELANOMA IN HUTCHINSON'S MELANOTIC FRECKLE	0	0	0	0	0	0	0	0	0	0	0	0	2	0	0	0	0	0
62104	MALIGNANT NEOPLASM OF TEMPORAL BONE	0	0	0	0	0	0	0	0	0	0	0	0	2	0	0	0	0	0
62126	Malignant neoplasm of thalamus	0	0	0	0	0	0	0	0	0	0	0	0	2	0	0	0	0	0
62182	MALIGNANT NEOPLASM OF VESTIBULE OF NOSE	0	0	0	0	0	0	0	0	0	0	0	0	2	0	0	0	0	0
62305	MALIGNANT NEOPLASM OF SKIN OF BUTTOCK	0	0	0	0	0	0	0	0	0	0	0	0	2	0	0	0	0	0
62348	[M]HAEMANGIOSARCOMA	0	0	0	0	0	0	0	0	0	0	0	0	2	0	0	0	0	0
62396	[M]EPITHELIOID CELL SARCOMA	0	0	0	0	0	0	0	0	0	0	0	0	2	0	0	0	0	0
62399	MALIG NEOP SKIN OF EAR AND EXTERNAL AURICULAR CANAL NOS	0	0	0	0	0	0	0	0	0	0	0	0	2	0	0	0	0	0
62475	Malignant melanoma of hand	0	0	0	0	0	0	0	0	0	0	0	0	2	0	0	0	0	0
62556	Malignant neoplasm of thymus	0	0	0	0	0	0	0	0	0	0	0	0	2	0	0	0	0	0
62630	Malignant neoplasm of long bones of leg NOS	0	0	0	0	0	0	0	0	0	0	0	0	2	0	0	0	0	0
62761	Malignant neoplasm of septum of nose	0	0	0	0	0	0	0	0	0	0	0	0	2	0	0	0	0	0
62785	[V]Personal history of malignant neoplasm of rectum	0	0	0	0	0	0	0	0	0	0	0	0	2	0	0	0	0	0
62814	[V]Personal history of malig neop of gastrointestinal tract	0	0	0	0	0	0	0	0	0	0	0	0	2	0	0	0	0	0
62840	MALIGNANT NEOPLASM OF VENTRAL SURFACE OF TONGUE	0	0	0	0	0	0	0	0	0	0	0	0	2	0	0	0	0	0
62941	[M]Neurofibrosarcoma	0	0	0	0	0	0	0	0	0	0	0	0	2	0	0	0	0	0
63102	[M]Islet cell carcinoma	0	0	0	0	0	0	0	0	0	0	0	0	2	0	0	0	0	0
63104	Malignant neoplasm of orbit NOS	0	0	0	0	0	0	0	0	0	0	0	0	2	0	0	0	0	0
63224	Malignant neoplasm of penis and other male genital organ NOS	0	0	0	0	0	0	0	0	0	0	0	0	2	0	0	0	0	0
63247	[M]Sarcoma botryoides	0	0	0	0	0	0	0	0	0	0	0	0	2	0	0	0	0	0
63286	[M]CLEAR CELL SARCOMA OF TENDONS AND APONEUROSES	0	0	0	0	0	0	0	0	0	0	0	0	2	0	0	0	0	0

63300	[X]Malignant neoplasm/overlap lesion/bone+articulr cartilage	0	0	0	0	0	0	0	0	0	0	0	0	2	0	0	0	0	0
63331	Malignant neoplasm of spermatic cord	0	0	0	0	0	0	0	0	0	0	0	0	2	0	0	0	0	0
63430	MALIGNANT NEOPLASM OF ENDOCARDIUM	0	0	0	0	0	0	0	0	0	0	0	0	2	0	0	0	0	0
63460	Malignant neoplasm of arytenoid cartilage	0	0	0	0	0	0	0	0	0	0	0	0	2	0	0	0	0	0
63470	MALIGNANT NEOPLASM OF ABDOMINAL OESOPHAGUS	0	0	0	0	0	0	0	0	0	0	0	0	2	0	0	0	0	0
63518	[M]Adenosarcoma	0	0	0	0	0	0	0	0	0	0	0	0	2	0	0	0	0	0
63568	Malignant neoplasm of peripheral nerves of head	0	0	0	0	0	0	0	0	0	0	0	0	2	0	0	0	0	0
63571	[M]PAROSTEAL OSTEOSARCOMA	0	0	0	0	0	0	0	0	0	0	0	0	2	0	0	0	0	0
63574	[M]MALIGNANT MELANOMA IN JUNCTIONAL NAEVUS	0	0	0	0	0	0	0	0	0	0	0	0	2	0	0	0	0	0
63598	[X]Malignant neoplasms/independent (primary) multiple sites	0	0	0	0	0	0	0	0	0	0	0	0	2	0	0	0	0	0
63657	MALIGNANT NEOPLASM OF CONJUNCTIVA	0	0	0	0	0	0	0	0	0	0	0	0	2	0	0	0	0	0
63659	[M]Juxtacortical chondrosarcoma	0	0	0	0	0	0	0	0	0	0	0	0	2	0	0	0	0	0
63695	MALIGNANT NEOPLASM OF PERIPHERAL NERVE OF THORAX	0	0	0	0	0	0	0	0	0	0	0	0	2	0	0	0	0	0
63925	[X]MALIGNANT NEOPLASM OF MENINGES, UNSPECIFIED	0	0	0	0	0	0	0	0	0	0	0	0	2	0	0	0	0	0
63979	MALIGNANT NEOPLASM OF FRENULUM LINGUAE	0	0	0	0	0	0	0	0	0	0	0	0	2	0	0	0	0	0
63988	MALIGNANT NEOPLASM OF CONNECTIVE AND SOFT TISSUE OF THUMB	0	0	0	0	0	0	0	0	0	0	0	0	2	0	0	0	0	0
63995	MALIGNANT NEOPLASM OF MECKEL'S DIVERTICULUM	0	0	0	0	0	0	0	0	0	0	0	0	2	0	0	0	0	0
63997	MALIGNANT MELANOMA OF THUMB	0	0	0	0	0	0	0	0	0	0	0	0	2	0	0	0	0	0
64106	MALIGNANT NEOPLASM OF SPECIFIED PARTS OF PERITONEUM NOS	0	0	0	0	0	0	0	0	0	0	0	0	2	0	0	0	0	0
64195	MALIG NEOP OF ENDOCRINE GLAND OR RELATED STRUCTURE NOS	0	0	0	0	0	0	0	0	0	0	0	0	2	0	0	0	0	0
64270	Malignant neoplasm of skin of ankle	0	0	0	0	0	0	0	0	0	0	0	0	2	0	0	0	0	0
64309	[X]Malignant neoplasm of endocrine gland, unspecified	0	0	0	0	0	0	0	0	0	0	0	0	2	0	0	0	0	0
64327	MALIGNANT MELANOMA OF LOWER LIMB OR HIP NOS	0	0	0	0	0	0	0	0	0	0	0	0	2	0	0	0	0	0
64345	Malignant neoplasm of connective and soft tissue, upper arm	0	0	0	0	0	0	0	0	0	0	0	0	2	0	0	0	0	0

64406	Malignant neoplasm of skin of thumb	0	0	0	0	0	0	0	0	0	0	0	0	2	0	0	0	0	0
64462	Malignant neoplasm of posterior pharynx	0	0	0	0	0	0	0	0	0	0	0	0	2	0	0	0	0	0
64497	[X]MALIGNANT NEOPLASM OF UTERINE ADNEXA, UNSPECIFIED	0	0	0	0	0	0	0	0	0	0	0	0	2	0	0	0	0	0
64516	MALIGNANT NEOPLASM OF PARIETAL PERITONEUM	0	0	0	0	0	0	0	0	0	0	0	0	2	0	0	0	0	0
64557	MALIGNANT NEOPLASM OF CEREBRAL PEDUNCLE	0	0	0	0	0	0	0	0	0	0	0	0	2	0	0	0	0	0
64568	[V]Personal history of malig neop of gastrointestinal tract	0	0	0	0	0	0	0	0	0	0	0	0	2	0	0	0	0	0
64596	[M]MYXOID LEIOMYOSARCOMA	0	0	0	0	0	0	0	0	0	0	0	0	2	0	0	0	0	0
64602	Malignant neoplasm of undescended testis	0	0	0	0	0	0	0	0	0	0	0	0	2	0	0	0	0	0
64686	MALIGNANT NEOPLASM OF AREOLA OF FEMALE BREAST	0	0	0	0	0	0	0	0	0	0	0	0	2	0	0	0	0	0
64727	Dukes stage D	0	0	0	0	0	0	0	0	0	0	0	0	2	0	0	0	0	0
64810	Malignant neoplasm of thorax NOS	0	0	0	0	0	0	0	0	0	0	0	0	2	0	0	0	0	0
64817	MALIGNANT NEOPLASM OF LACRIMAL GLAND	0	0	0	0	0	0	0	0	0	0	0	0	2	0	0	0	0	0
64848	MALIGNANT NEOPLASM OF ULNA	0	0	0	0	0	0	0	0	0	0	0	0	2	0	0	0	0	0
64897	[X]Malignant neoplasms/independent(primary)multiple sites	0	0	0	0	0	0	0	0	0	0	0	0	2	0	0	0	0	0
64971	Malignant neoplasm of olfactory bulb	0	0	0	0	0	0	0	0	0	0	0	0	2	0	0	0	0	0
65047	[M]PHAEOCHROMOCYTOMA, MALIGNANT	0	0	0	0	0	0	0	0	0	0	0	0	2	0	0	0	0	0
65051	[M]Papillary cystadenocarcinoma, NOS	0	0	0	0	0	0	0	0	0	0	0	0	2	0	0	0	0	0
65106	MALIGNANT NEOPLASM OF UTERINE ADNEXA NOS	0	0	0	0	0	0	0	0	0	0	0	0	2	0	0	0	0	0
65124	MALIGNANT NEOPLASM OF INTERLOBULAR BILE DUCTS	0	0	0	0	0	0	0	0	0	0	0	0	2	0	0	0	0	0
65159	Malignant neoplasm of perinephric tissue	0	0	0	0	0	0	0	0	0	0	0	0	2	0	0	0	0	0
65164	MALIGNANT MELANOMA OF UPPER LIMB AND SHOULDER	0	0	0	0	0	0	0	0	0	0	0	0	2	0	0	0	0	0
65215	Malignant neoplasm of sphenoidal sinus	0	0	0	0	0	0	0	0	0	0	0	0	2	0	0	0	0	0
65216	[M]Cloacogenic carcinoma	0	0	0	0	0	0	0	0	0	0	0	0	2	0	0	0	0	0

65233	Malig neop connective and soft tissue other specified site	0	0	0	0	0	0	0	0	0	0	0	0	2	0	0	0	0	0
65241	MALIGNANT NEOPLASM, OVERLAPPING LESION OF BRAIN	0	0	0	0	0	0	0	0	0	0	0	0	2	0	0	0	0	0
65312	Malignant neoplasm of anterior wall of stomach NEC	0	0	0	0	0	0	0	0	0	0	0	0	2	0	0	0	0	0
65357	Malignant neoplasm of nasolacrimal duct	0	0	0	0	0	0	0	0	0	0	0	0	2	0	0	0	0	0
65372	Malignant neoplasm of other specified site of stomach NOS	0	0	0	0	0	0	0	0	0	0	0	0	2	0	0	0	0	0
65458	MALIG NEOP OF OTHER AND UNSPECIFIED PARTS OF NERVOUS SYSTEM	0	0	0	0	0	0	0	0	0	0	0	0	2	0	0	0	0	0
65460	Malignant neoplasm of spleen NEC	0	0	0	0	0	0	0	0	0	0	0	0	2	0	0	0	0	0
65466	Kaposi's sarcoma of multiple organs	0	0	0	0	0	0	0	0	0	0	0	0	2	0	0	0	0	0
65599	Malignant neoplasm of acoustic nerve	0	0	0	0	0	0	0	0	0	0	0	0	2	0	0	0	0	0
65605	MALIGNANT NEOPLASM OF MYOCARDIUM	0	0	0	0	0	0	0	0	0	0	0	0	2	0	0	0	0	0
65625	MALIGNANT MELANOMA OF SCALP AND NECK	0	0	0	0	0	0	0	0	0	0	0	0	2	0	0	0	0	0
65782	Malignant neoplasm of skin of toe	0	0	0	0	0	0	0	0	0	0	0	0	2	0	0	0	0	0
65793	Malig neop of upper respiratory tract, part unspecified	0	0	0	0	0	0	0	0	0	0	0	0	2	0	0	0	0	0
65861	[M]DERMOID CYST WITH MALIGNANT TRANSFORMATION	0	0	0	0	0	0	0	0	0	0	0	0	2	0	0	0	0	0
65880	MALIG NEOP OF SCAPULA AND LONG BONES OF UPPER ARM NOS	0	0	0	0	0	0	0	0	0	0	0	0	2	0	0	0	0	0
65952	[M]DESMOPLASTIC MEDULLOBLASTOMA	0	0	0	0	0	0	0	0	0	0	0	0	2	0	0	0	0	0
66000	[M]ADENOCARCINOMA WITH APOCRINE METAPLASIA	0	0	0	0	0	0	0	0	0	0	0	0	2	0	0	0	0	0
66064	[M]GIANT CELL GLIOBLASTOMA	0	0	0	0	0	0	0	0	0	0	0	0	2	0	0	0	0	0
66088	Malig neop of connective and soft tissue of hip and leg	0	0	0	0	0	0	0	0	0	0	0	0	2	0	0	0	0	0
66166	Malignant neoplasm	0	0	0	0	0	0	0	0	0	0	0	0	2	0	0	0	0	0
66270	MALIGNANT NEOPLASM OF UPPER LIP, EXTERNAL	0	0	0	0	0	0	0	0	0	0	0	0	2	0	0	0	0	0
66319	MALIGNANT NEOPLASM OF SKIN OF GROIN	0	0	0	0	0	0	0	0	0	0	0	0	2	0	0	0	0	0
66384	MALIGNANT NEOPLASM OF LOWER LIP, EXTERNAL	0	0	0	0	0	0	0	0	0	0	0	0	2	0	0	0	0	0
66422	MALIGNANT NEOPLASM, OVERLAPPING LESION OF NASOPHARYNX	0	0	0	0	0	0	0	0	0	0	0	0	2	0	0	0	0	0

66444	[X]Malignant neoplasm/overlap lesion/heart,mediastinm+pleura	0	0	0	0	0	0	0	0	0	0	0	0	2	0	0	0	0	0
66447	MALIGNANT NEOPLASM OF SKIN OF SCAPULAR REGION	0	0	0	0	0	0	0	0	0	0	0	0	2	0	0	0	0	0
66457	[V]Personal history of other specified malignant neoplasm	0	0	0	0	0	0	0	0	0	0	0	0	2	0	0	0	0	0
66488	MALIG NEOP OF CONNECTIVE AND SOFT TISSUE OF ABDOMINAL WALL	0	0	0	0	0	0	0	0	0	0	0	0	2	0	0	0	0	0
66541	[M]Round cell carcinoma	0	0	0	0	0	0	0	0	0	0	0	0	2	0	0	0	0	0
66607	[M]Mixed tumour	0	0	0	0	0	0	0	0	0	0	0	0	2	0	0	0	0	0
66639	Malignant neoplasm of clavicle	0	0	0	0	0	0	0	0	0	0	0	0	2	0	0	0	0	0
66646	MALIGNANT NEOPLASM, OVERLAP LESION OF RESP & INTRATHOR ORGS	0	0	0	0	0	0	0	0	0	0	0	0	2	0	0	0	0	0
66750	Malignant neoplasm of heart	0	0	0	0	0	0	0	0	0	0	0	0	2	0	0	0	0	0
66876	[M]PSEUDOMUCINOUS ADENOCARCINOMA	0	0	0	0	0	0	0	0	0	0	0	0	2	0	0	0	0	0
66908	MALIGNANT NEOPLASM OF COCCYGEAL VERTEBRA	0	0	0	0	0	0	0	0	0	0	0	0	2	0	0	0	0	0
67019	[M]Angiomyosarcoma	0	0	0	0	0	0	0	0	0	0	0	0	2	0	0	0	0	0
67034	[X]MESOTHELIOMA OF OTHER SITES	0	0	0	0	0	0	0	0	0	0	0	0	2	0	0	0	0	0
67107	MALIGNANT NEOPLASM OF PARIETAL PLEURA	0	0	0	0	0	0	0	0	0	0	0	0	2	0	0	0	0	0
67211	Malignant neoplasm of spinal meninges NOS	0	0	0	0	0	0	0	0	0	0	0	0	2	0	0	0	0	0
67217	MALIGNANT NEOPLASM OF TRUNK NOS	0	0	0	0	0	0	0	0	0	0	0	0	2	0	0	0	0	0
67236	MALIGNANT NEOPLASM OF HIPPOCAMPUS	0	0	0	0	0	0	0	0	0	0	0	0	2	0	0	0	0	0
67288	[M]Medulloepithelioma NOS	0	0	0	0	0	0	0	0	0	0	0	0	2	0	0	0	0	0
67323	MALIGNANT NEOPLASM OF OROPHARYNX, OTHER SPECIFIED SITES	0	0	0	0	0	0	0	0	0	0	0	0	2	0	0	0	0	0
67324	MALIG NEOP OF CONNECTIVE AND SOFT TISSUE OF INGUINAL REGION	0	0	0	0	0	0	0	0	0	0	0	0	2	0	0	0	0	0
67342	[M]Adenocarcinoma in villous adenoma	0	0	0	0	0	0	0	0	0	0	0	0	2	0	0	0	0	0
67430	[M]TIBIAL ADAMANTINOMA	0	0	0	0	0	0	0	0	0	0	0	0	2	0	0	0	0	0
67446	MALIGNANT NEOPLASM OF LOWER LIP, VERMILION BORDER	0	0	0	0	0	0	0	0	0	0	0	0	2	0	0	0	0	0
67451	MALIGNANT NEOPLASM/OVERLAP LESION/BONE+ARTICULR CARTILAGE	0	0	0	0	0	0	0	0	0	0	0	0	2	0	0	0	0	0

67497	Malignant neoplasm	0	0	0	0	0	0	0	0	0	0	0	0	2	0	0	0	0	0
67504	MALIGNANT NEOPLASM OF LOWER LIP, BUCCAL ASPECT	0	0	0	0	0	0	0	0	0	0	0	0	2	0	0	0	0	0
67587	[M]PLEOMORPHIC XANTHOASTROCYTOMA	0	0	0	0	0	0	0	0	0	0	0	0	2	0	0	0	0	0
67701	[M]Secretory breast carcinoma	0	0	0	0	0	0	0	0	0	0	0	0	2	0	0	0	0	0
67712	[M]Choriocarcinoma	0	0	0	0	0	0	0	0	0	0	0	0	2	0	0	0	0	0
67748	MALIGNANT NEOPLASM OF SKIN OF UMBILICUS	0	0	0	0	0	0	0	0	0	0	0	0	2	0	0	0	0	0
67763	MALIGNANT NEOPLASM OF COSTO-VERTEBRAL JOINT	0	0	0	0	0	0	0	0	0	0	0	0	2	0	0	0	0	0
67806	MALIGNANT MELANOMA OF FACE NOS	0	0	0	0	0	0	0	0	0	0	0	0	2	0	0	0	0	0
67884	MALIGNANT NEOPLASM OF AREOLA OF MALE BREAST	0	0	0	0	0	0	0	0	0	0	0	0	2	0	0	0	0	0
67912	[M]Papillary epidermoid carcinoma	0	0	0	0	0	0	0	0	0	0	0	0	2	0	0	0	0	0
67914	MALIGNANT NEOPLASM OF SKIN OF GREAT TOE	0	0	0	0	0	0	0	0	0	0	0	0	2	0	0	0	0	0
67934	[M]CARCINOSARCOMA, EMBRYONAL TYPE	0	0	0	0	0	0	0	0	0	0	0	0	2	0	0	0	0	0
67949	Malignant neoplasm of other male genital organ	0	0	0	0	0	0	0	0	0	0	0	0	2	0	0	0	0	0
67966	[M]NAEVOCARCINOMA	0	0	0	0	0	0	0	0	0	0	0	0	2	0	0	0	0	0
67970	[M]SMALL CELL CARCINOMA, FUSIFORM CELL TYPE	0	0	0	0	0	0	0	0	0	0	0	0	2	0	0	0	0	0
68018	[V]Personal history of malignant neoplasm of anus	0	0	0	0	0	0	0	0	0	0	0	0	2	0	0	0	0	0
68027	[X]Malignant neoplasm/other and unspecified cranial nerves	0	0	0	0	0	0	0	0	0	0	0	0	2	0	0	0	0	0
68055	MALIGNANT NEOPLASM OF LONG BONES OF LEG	0	0	0	0	0	0	0	0	0	0	0	0	2	0	0	0	0	0
68133	Malignant melanoma of forehead	0	0	0	0	0	0	0	0	0	0	0	0	2	0	0	0	0	0
68155	MALIGNANT NEOPLASM OF FUNDUS OF CORPUS UTERI	0	0	0	0	0	0	0	0	0	0	0	0	2	0	0	0	0	0
68161	MALIGNANT NEOPLASM OF SEMINAL VESICLE	0	0	0	0	0	0	0	0	0	0	0	0	2	0	0	0	0	0
68197	MALIGNANT NEOPLASM OF SKIN OF POPLITEAL FOSSA AREA	0	0	0	0	0	0	0	0	0	0	0	0	2	0	0	0	0	0
68220	[M]Fibrochondrosarcoma	0	0	0	0	0	0	0	0	0	0	0	0	2	0	0	0	0	0
68236	MALIGNANT NEOPLASM OF HEAD, NECK AND FACE	0	0	0	0	0	0	0	0	0	0	0	0	2	0	0	0	0	0

68399	Malignant neoplasm of lip unspecified, mucosa	0	0	0	0	0	0	0	0	0	0	0	0	2	0	0	0	0	0
68410	Primary angiosarcoma of liver	0	0	0	0	0	0	0	0	0	0	0	0	2	0	0	0	0	0
68447	[M]BLUE NAEVUS, MALIGNANT	0	0	0	0	0	0	0	0	0	0	0	0	2	0	0	0	0	0
68456	[M]CHROMOPHOBE CARCINOMA	0	0	0	0	0	0	0	0	0	0	0	0	2	0	0	0	0	0
68480	Malignant neoplasm of nipple of male breast	0	0	0	0	0	0	0	0	0	0	0	0	2	0	0	0	0	0
68612	[V]Personal history of unspecified malignant neoplasm	0	0	0	0	0	0	0	0	0	0	0	0	2	0	0	0	0	0
68641	Malignant neoplasm of brain stem NOS	0	0	0	0	0	0	0	0	0	0	0	0	2	0	0	0	0	0
68730	[M]AMELOBLASTIC FIBROSARCOMA	0	0	0	0	0	0	0	0	0	0	0	0	2	0	0	0	0	0
68757	[M]Nonencapsulated sclerosing carcinoma	0	0	0	0	0	0	0	0	0	0	0	0	2	0	0	0	0	0
68783	[M]SKIN APPENDAGE CARCINOMA	0	0	0	0	0	0	0	0	0	0	0	0	2	0	0	0	0	0
68787	Malignant neoplasm of back NOS	0	0	0	0	0	0	0	0	0	0	0	0	2	0	0	0	0	0
68824	MALIGNANT NEOPLASM, OVERLAPPING LESION MALE GENITAL ORGS	0	0	0	0	0	0	0	0	0	0	0	0	2	0	0	0	0	0
68889	[M]Balloon cell melanoma	0	0	0	0	0	0	0	0	0	0	0	0	2	0	0	0	0	0
68956	[M]GIANT CELL TUMOUR OF BONE, MALIGNANT	0	0	0	0	0	0	0	0	0	0	0	0	2	0	0	0	0	0
69104	MALIGNANT NEOPLASM OF CARPAL BONE - LUNATE	0	0	0	0	0	0	0	0	0	0	0	0	2	0	0	0	0	0
69146	Malignant neoplasm of bones of skull and face NOS	0	0	0	0	0	0	0	0	0	0	0	0	2	0	0	0	0	0
69210	[M]Goblet cell tumour	0	0	0	0	0	0	0	0	0	0	0	0	2	0	0	0	0	0
69300	[M]POLYGONAL CELL CARCINOMA	0	0	0	0	0	0	0	0	0	0	0	0	2	0	0	0	0	0
69671	MALIGNANT NEOPLASM OF POSTERIOR THIRD OF TONGUE	0	0	0	0	0	0	0	0	0	0	0	0	2	0	0	0	0	0
69761	MALIGNANT NEOPLASM OF LIP, VERMILION BORDER NOS	0	0	0	0	0	0	0	0	0	0	0	0	2	0	0	0	0	0
69821	Malignant neoplasm of the pouch of Douglas	0	0	0	0	0	0	0	0	0	0	0	0	2	0	0	0	0	0
69844	[M]Round cell sarcoma	0	0	0	0	0	0	0	0	0	0	0	0	2	0	0	0	0	0
69927	Malignant neoplasm of first metatarsal bone	0	0	0	0	0	0	0	0	0	0	0	0	2	0	0	0	0	0
69951	MALIGNANT NEOPLASM OF ROOF OF MOUTH	0	0	0	0	0	0	0	0	0	0	0	0	2	0	0	0	0	0

69978	[M]BORDERLINE MUCINOUS CYSTADENOMA OF THE OVARY	0	0	0	0	0	0	0	0	0	0	0	0	2	0	0	0	0	0
69981	[M]Neurilemmoma, malignant	0	0	0	0	0	0	0	0	0	0	0	0	2	0	0	0	0	0
70104	Malignant neoplasm of cerebral meninges NOS	0	0	0	0	0	0	0	0	0	0	0	0	2	0	0	0	0	0
70126	Malignant neoplasm of optic nerve	0	0	0	0	0	0	0	0	0	0	0	0	2	0	0	0	0	0
70380	MALIGNANT NEOPLASM OF SKIN OF AXILLARY FOLD	0	0	0	0	0	0	0	0	0	0	0	0	2	0	0	0	0	0
70383	[M]BRENNER TUMOUR, MALIGNANT	0	0	0	0	0	0	0	0	0	0	0	0	2	0	0	0	0	0
70463	MALIGNANT NEOPLASM OF CONNECTIVE AND SOFT TISSUE OF BUTTOCK	0	0	0	0	0	0	0	0	0	0	0	0	2	0	0	0	0	0
70516	[M]Biliary tract adenomas and adenocarcinomas	0	0	0	0	0	0	0	0	0	0	0	0	2	0	0	0	0	0
70587	MALIGNANT NEOPLASM OF SKIN OF FOOT	0	0	0	0	0	0	0	0	0	0	0	0	2	0	0	0	0	0
70637	Malignant melanoma of lip	0	0	0	0	0	0	0	0	0	0	0	0	2	0	0	0	0	0
70696	Malignant neoplasm of other major salivary glands	0	0	0	0	0	0	0	0	0	0	0	0	2	0	0	0	0	0
70729	Malignant neoplasm of isthmus of uterine body NOS	0	0	0	0	0	0	0	0	0	0	0	0	2	0	0	0	0	0
70819	Malignant neoplasm of palate unspecified	0	0	0	0	0	0	0	0	0	0	0	0	2	0	0	0	0	0
70824	MALIGNANT NEOPLASM OF ADRENAL GLAND NOS	0	0	0	0	0	0	0	0	0	0	0	0	2	0	0	0	0	0
70928	MALIGNANT NEOPLASM OF SUBLINGUAL GLAND	0	0	0	0	0	0	0	0	0	0	0	0	2	0	0	0	0	0
70942	MALIGNANT NEOPLASM OF HYPOTHALAMUS	0	0	0	0	0	0	0	0	0	0	0	0	2	0	0	0	0	0
70988	MALIGNANT NEOPLASM OF SKIN OF HIP	0	0	0	0	0	0	0	0	0	0	0	0	2	0	0	0	0	0
71136	Malignant melanoma of chin	0	0	0	0	0	0	0	0	0	0	0	0	2	0	0	0	0	0
71139	Malignant neoplasm of other parts of brain	0	0	0	0	0	0	0	0	0	0	0	0	2	0	0	0	0	0
71147	Malignant neoplasm of lower lip	0	0	0	0	0	0	0	0	0	0	0	0	2	0	0	0	0	0
71204	MALIGNANT NEOPLASM OF CARTILAGE OF NOSE	0	0	0	0	0	0	0	0	0	0	0	0	2	0	0	0	0	0
71301	[M]Struma ovarii, malignant	0	0	0	0	0	0	0	0	0	0	0	0	2	0	0	0	0	0
71497	[M]Oxyphilic adenocarcinoma	0	0	0	0	0	0	0	0	0	0	0	0	2	0	0	0	0	0
71584	Malignant neoplasm of lacrimal duct	0	0	0	0	0	0	0	0	0	0	0	0	2	0	0	0	0	0

71627	[M]SWEAT GLAND ADENOCARCINOMA	0	0	0	0	0	0	0	0	0	0	0	0	2	0	0	0	0	0
71810	MALIGNANT NEOPLASM OF SCAPULA AND LONG BONES OF UPPER ARM	0	0	0	0	0	0	0	0	0	0	0	0	2	0	0	0	0	0
71869	[M]ALVEOLAR SOFT PART SARCOMA	0	0	0	0	0	0	0	0	0	0	0	0	2	0	0	0	0	0
71895	[M]Superficial spreading adenocarcinoma	0	0	0	0	0	0	0	0	0	0	0	0	2	0	0	0	0	0
71946	MALIGNANT NEOPLASM OF MASTOID AIR CELLS	0	0	0	0	0	0	0	0	0	0	0	0	2	0	0	0	0	0
72127	MALIGNANT NEOPLASM OF EPIDIDYMIS	0	0	0	0	0	0	0	0	0	0	0	0	2	0	0	0	0	0
72174	Malignant neoplasm of paraurethral glands	0	0	0	0	0	0	0	0	0	0	0	0	2	0	0	0	0	0
72212	MALIGNANT NEOPLASM OF CALCANEUM	0	0	0	0	0	0	0	0	0	0	0	0	2	0	0	0	0	0
72224	FIBROSARCOMA OF SPLEEN	0	0	0	0	0	0	0	0	0	0	0	0	2	0	0	0	0	0
72262	[V]Personal history of malig neop other intrathoracic organ	0	0	0	0	0	0	0	0	0	0	0	0	2	0	0	0	0	0
72277	[M]Basophil carcinoma	0	0	0	0	0	0	0	0	0	0	0	0	2	0	0	0	0	0
72443	[M]ODONTOGENIC TUMOUR, MALIGNANT	0	0	0	0	0	0	0	0	0	0	0	0	2	0	0	0	0	0
72445	MALIGNANT NEOPLASM OF CYSTIC DUCT	0	0	0	0	0	0	0	0	0	0	0	0	2	0	0	0	0	0
72464	Malignant neoplasm of metacarpal bones	0	0	0	0	0	0	0	0	0	0	0	0	2	0	0	0	0	0
72522	Malignant neoplasm of great vessels	0	0	0	0	0	0	0	0	0	0	0	0	2	0	0	0	0	0
72723	Malignant neoplasm of cornu of corpus uteri	0	0	0	0	0	0	0	0	0	0	0	0	2	0	0	0	0	0
73251	[M]MALIGNANT MELANOMA IN GIANT PIGMENTED NAEVUS	0	0	0	0	0	0	0	0	0	0	0	0	2	0	0	0	0	0
73275	[M]ADENOCARCINOMA IN ADENOMATOUS POLPOSIS COLI	0	0	0	0	0	0	0	0	0	0	0	0	2	0	0	0	0	0
73296	[X]Malignant neoplasm/bones+articular cartilage/limb,unspfd	0	0	0	0	0	0	0	0	0	0	0	0	2	0	0	0	0	0
73434	[M]ADENOCARCINOMA IN MULTIPLE ADENOMATOUS POLYPS	0	0	0	0	0	0	0	0	0	0	0	0	2	0	0	0	0	0
73439	MALIGNANT NEOPLASM OF ANTERIOR EPIGLOTTIS NOS	0	0	0	0	0	0	0	0	0	0	0	0	2	0	0	0	0	0
73510	MALIGNANT NEOPLASM OF SUPRACLAVICULAR FOSSA NOS	0	0	0	0	0	0	0	0	0	0	0	0	2	0	0	0	0	0
73530	Malignant neoplasm of hand bones	0	0	0	0	0	0	0	0	0	0	0	0	2	0	0	0	0	0
73536	Malignant melanoma of hip	0	0	0	0	0	0	0	0	0	0	0	0	2	0	0	0	0	0

73537	MALIG NEOP AUDITORY TUBE, MIDDLE EAR, MASTOID AIR CELLS NOS	0	0	0	0	0	0	0	0	0	0	0	0	2	0	0	0	0	0
73556	MALIGNANT NEOPLASM OF HAND BONES NOS	0	0	0	0	0	0	0	0	0	0	0	0	2	0	0	0	0	0
73614	MALIGNANT NEOPLASM OF LIP UNSPECIFIED, BUCCAL ASPECT	0	0	0	0	0	0	0	0	0	0	0	0	2	0	0	0	0	0
73718	Malig neop connective and soft tissue head	0	0	0	0	0	0	0	0	0	0	0	0	2	0	0	0	0	0
73744	Malignant melanoma of ear and external auricular canal NOS	0	0	0	0	0	0	0	0	0	0	0	0	2	0	0	0	0	0
73760	Malignant neoplasm of scalp or skin of neck NOS	0	0	0	0	0	0	0	0	0	0	0	0	2	0	0	0	0	0
73916	[M]Epithelioid leiomyosarcoma	0	0	0	0	0	0	0	0	0	0	0	0	2	0	0	0	0	0
73962	Malignant neoplasm of upper lip, vermilion border	0	0	0	0	0	0	0	0	0	0	0	0	2	0	0	0	0	0
73988	MALIGNANT NEOPLASM OF PERIPHERAL NERVE OF PELVIS	0	0	0	0	0	0	0	0	0	0	0	0	2	0	0	0	0	0
73992	MALIGNANT NEOPLASM OF CORNEA	0	0	0	0	0	0	0	0	0	0	0	0	2	0	0	0	0	0
74896	MALIGNANT NEOPLASM OF EXTRAHEPATIC BILE DUCTS NOS	0	0	0	0	0	0	0	0	0	0	0	0	2	0	0	0	0	0
86046	Malignant neoplasm of peripheral nerve of abdomen	0	0	0	0	0	0	0	0	0	0	0	0	2	0	0	0	0	0
86812	Malignant neoplasm of phalanges of hand	0	0	0	0	0	0	0	0	0	0	0	0	2	0	0	0	0	0
86820	[M]MESOTHELIOMA, BIPHASIC TYPE, MALIGNANT	0	0	0	0	0	0	0	0	0	0	0	0	2	0	0	0	0	0
86996	MALIGNANT NEOPLASM OF CONNECTIVE TISSUE OF ORBIT	0	0	0	0	0	0	0	0	0	0	0	0	2	0	0	0	0	0
86997	[X]MALIGNANT NEOPLASM/ILL-DEFINED SITES WITHIN RESP SYSTEM	0	0	0	0	0	0	0	0	0	0	0	0	2	0	0	0	0	0
87003	[M]MESENCHYMOMA, MALIGNANT	0	0	0	0	0	0	0	0	0	0	0	0	2	0	0	0	0	0
87113	MALIGNANT NEOPLASM-PLURIGLANDULAR INVOLVEMENT,UNSPECIFIED	0	0	0	0	0	0	0	0	0	0	0	0	2	0	0	0	0	0
88144	Malignant neoplasm of other specified part of nervous system	0	0	0	0	0	0	0	0	0	0	0	0	2	0	0	0	0	0
88362	Malignant neoplasm of other specified hypopharyngeal site	0	0	0	0	0	0	0	0	0	0	0	0	2	0	0	0	0	0
89258	Malignant neoplasm of peripheral nerve of low limb, incl hip	0	0	0	0	0	0	0	0	0	0	0	0	2	0	0	0	0	0
89593	Malignant neoplasm of intrahepatic biliary passages	0	0	0	0	0	0	0	0	0	0	0	0	2	0	0	0	0	0
89909	MALIGNANT NEOPLASM OF LOWER LIP, MUCOSA	0	0	0	0	0	0	0	0	0	0	0	0	2	0	0	0	0	0
89916	Malignant neoplasm of presacral region	0	0	0	0	0	0	0	0	0	0	0	0	2	0	0	0	0	0

90124	MALIGNANT NEOPLASM OF POSTERIOR WALL OF OROPHARYNX	0	0	0	0	0	0	0	0	0	0	0	0	2	0	0	0	0	0
90290	Malignant neoplasm of mesentery	0	0	0	0	0	0	0	0	0	0	0	0	2	0	0	0	0	0
90546	MALIG NEOP CONNECTIVE AND SOFT TISSUE HIP AND LEG NOS	0	0	0	0	0	0	0	0	0	0	0	0	2	0	0	0	0	0
90610	MALIGNANT NEOPLASM OF UPPER LIP, ORAL ASPECT	0	0	0	0	0	0	0	0	0	0	0	0	2	0	0	0	0	0
90659	MALIGNANT NEOPLASM OF OTHER SPECIFIED ENDOCRINE GLAND	0	0	0	0	0	0	0	0	0	0	0	0	2	0	0	0	0	0
91035	Malignant neoplasm of fixed part of tongue NOS	0	0	0	0	0	0	0	0	0	0	0	0	2	0	0	0	0	0
91037	MALIGNANT NEOPLASM OF OTHER SPECIFIED SITE OF OROPHARYNX NOS	0	0	0	0	0	0	0	0	0	0	0	0	2	0	0	0	0	0
91240	Malignant neoplasm of pons	0	0	0	0	0	0	0	0	0	0	0	0	2	0	0	0	0	0
91457	[X]Malignant neoplasm/connective + soft tissue,unspecified	0	0	0	0	0	0	0	0	0	0	0	0	2	0	0	0	0	0
91509	Malignant neoplasm of descended testis NOS	0	0	0	0	0	0	0	0	0	0	0	0	2	0	0	0	0	0
91586	Malignant neoplasm of connective and soft tissue of finger	0	0	0	0	0	0	0	0	0	0	0	0	2	0	0	0	0	0
91843	MALIGNANT NEOPLASM OF LOWER LIP, FRENULUM	0	0	0	0	0	0	0	0	0	0	0	0	2	0	0	0	0	0
91895	MALIGNANT NEOPLASM OF GLOSSOEPIGLOTTIC FOLD	0	0	0	0	0	0	0	0	0	0	0	0	2	0	0	0	0	0
91896	[X]MAL NEOPLASM/CONNECTIVE+SOFT TISSUE OF TRUNK,UNSPECIFIED	0	0	0	0	0	0	0	0	0	0	0	0	2	0	0	0	0	0
92293	[M]SPINDLE CELL MELANOMA, TYPE B	0	0	0	0	0	0	0	0	0	0	0	0	2	0	0	0	0	0
92329	MALIGNANT NEOPLASM OF OTHER MALE GENITAL ORGAN NOS	0	0	0	0	0	0	0	0	0	0	0	0	2	0	0	0	0	0
92371	Malignant neoplasm of radius	0	0	0	0	0	0	0	0	0	0	0	0	2	0	0	0	0	0
92382	Malignant neoplasm of fourth metatarsal bone	0	0	0	0	0	0	0	0	0	0	0	0	2	0	0	0	0	0
92720	Malignant neoplasm of posterior mediastinum	0	0	0	0	0	0	0	0	0	0	0	0	2	0	0	0	0	0
93175	[M]INTRAOSSEOUS CARCINOMA	0	0	0	0	0	0	0	0	0	0	0	0	2	0	0	0	0	0
93218	MALIGNANT NEOPLASM OF GUM NOS	0	0	0	0	0	0	0	0	0	0	0	0	2	0	0	0	0	0
93352	Squamous cell carcinoma of skin	0	0	0	0	0	0	0	0	0	0	0	0	2	0	0	0	0	0
93402	Excision biopsy of basal cell carcinoma	0	0	0	0	0	0	0	0	0	0	0	0	2	0	0	0	0	0
93478	MALIGNANT NEOPLASM, OVERLAPPING LESION OF COLON	0	0	0	0	0	0	0	0	0	0	0	0	2	0	0	0	0	0

93490	Squamous cell carcinoma of skin NOS	0	0	0	0	0	0	0	0	0	0	0	0	2	0	0	0	0	0
93537	MALIGNANT NEOPLASM OF MIDBRAIN	0	0	0	0	0	0	0	0	0	0	0	0	2	0	0	0	0	0
93665	[X]Kaposi's sarcoma	0	0	0	0	0	0	0	0	0	0	0	0	2	0	0	0	0	0
93762	MALIGNANT NEOPLASM OF PLACENTA	0	0	0	0	0	0	0	0	0	0	0	0	2	0	0	0	0	0
93778	Malignant neoplasm of spleen NOS	0	0	0	0	0	0	0	0	0	0	0	0	2	0	0	0	0	0
93842	Malignant neoplasm of palatopharyngeal arch	0	0	0	0	0	0	0	0	0	0	0	0	2	0	0	0	0	0
94000	Bowel cancer detected by national screening programme	0	0	0	0	0	0	0	0	0	0	0	0	2	0	0	0	0	0
94083	[M]SOLID CARCINOMA NOS	0	0	0	0	0	0	0	0	0	0	0	0	2	0	0	0	0	0
94220	MALIGNANT NEOPLASM OF ADRENAL MEDULLA	0	0	0	0	0	0	0	0	0	0	0	0	2	0	0	0	0	0
94251	MALIGNANT NEOPLASM OF LIP, UNSPECIFIED, LIPSTICK AREA	0	0	0	0	0	0	0	0	0	0	0	0	2	0	0	0	0	0
94272	Malig neoplasm of connective and soft tissues of lumb spine	0	0	0	0	0	0	0	0	0	0	0	0	2	0	0	0	0	0
94278	Malignant neoplasm of gastro-oesophageal junction	0	0	0	0	0	0	0	0	0	0	0	0	2	0	0	0	0	0
94286	[M]Congenital fibrosarcoma	0	0	0	0	0	0	0	0	0	0	0	0	2	0	0	0	0	0
94355	MALIGNANT NEOPLASM OF FLANK NOS	0	0	0	0	0	0	0	0	0	0	0	0	2	0	0	0	0	0
94390	MALIGNANT NEOPLASM OF ROOF OF NASOPHARYNX	0	0	0	0	0	0	0	0	0	0	0	0	2	0	0	0	0	0
94427	MALIGNANT NEOPLASM OF FIFTH METACARPAL BONE	0	0	0	0	0	0	0	0	0	0	0	0	2	0	0	0	0	0
94438	[M]SIGNET RING CARCINOMA NOS	0	0	0	0	0	0	0	0	0	0	0	0	2	0	0	0	0	0
94441	MALIGNANT NEOPLASM OF LOWER LIP, ORAL ASPECT	0	0	0	0	0	0	0	0	0	0	0	0	2	0	0	0	0	0
94776	Malignant neoplasm	0	0	0	0	0	0	0	0	0	0	0	0	2	0	0	0	0	0
94810	[M]Adenocarcinoma with spindle cell metaplasia	0	0	0	0	0	0	0	0	0	0	0	0	2	0	0	0	0	0
94873	[M]SQUAMOUS CELL CARCINOMA OF SKIN NOS	0	0	0	0	0	0	0	0	0	0	0	0	2	0	0	0	0	0
94975	MALIGNANT NEOPLASM OF PERICARDIUM	0	0	0	0	0	0	0	0	0	0	0	0	2	0	0	0	0	0
95008	[M]GELATINOUS ADENOCARCINOMA	0	0	0	0	0	0	0	0	0	0	0	0	2	0	0	0	0	0
95016	Malignant neoplasm of Waldeyer's ring	0	0	0	0	0	0	0	0	0	0	0	0	2	0	0	0	0	0

95024	[M]Infantile fibrosarcoma	0	0	0	0	0	0	0	0	0	0	0	0	2	0	0	0	0	0
95057	Malignant neoplasm of ectopic site of female breast	0	0	0	0	0	0	0	0	0	0	0	0	2	0	0	0	0	0
95150	[M]Serous surface papillary carcinoma	0	0	0	0	0	0	0	0	0	0	0	0	2	0	0	0	0	0
95182	MALIGNANT NEOPLASM OF TALUS	0	0	0	0	0	0	0	0	0	0	0	0	2	0	0	0	0	0
95323	Malignant neoplasm of ectopic site of male breast	0	0	0	0	0	0	0	0	0	0	0	0	2	0	0	0	0	0
95373	[M]LEYDIG CELL TUMOUR, MALIGNANT	0	0	0	0	0	0	0	0	0	0	0	0	2	0	0	0	0	0
95421	Malignant neoplasm of other specified female genital organ	0	0	0	0	0	0	0	0	0	0	0	0	2	0	0	0	0	0
95429	Malignant neoplasm of posterior wall of nasopharynx	0	0	0	0	0	0	0	0	0	0	0	0	2	0	0	0	0	0
95458	MALIGNANT NEOPLASM OF NASAL BONE	0	0	0	0	0	0	0	0	0	0	0	0	2	0	0	0	0	0
95480	MALIGNANT NEOPLASM OF LOWER LIP, LIPSTICK AREA	0	0	0	0	0	0	0	0	0	0	0	0	2	0	0	0	0	0
95505	Malignant neoplasm of cervical stump	0	0	0	0	0	0	0	0	0	0	0	0	2	0	0	0	0	0
95609	[M]INSULINOMA, MALIGNANT	0	0	0	0	0	0	0	0	0	0	0	0	2	0	0	0	0	0
95629	MALIGNANT MELANOMA OF PERINEUM	0	0	0	0	0	0	0	0	0	0	0	0	2	0	0	0	0	0
95644	MALIGNANT NEOPLASM OF HEART	0	0	0	0	0	0	0	0	0	0	0	0	2	0	0	0	0	0
95671	[X]Malignant neoplasm of peritoneum, unspecified	0	0	0	0	0	0	0	0	0	0	0	0	2	0	0	0	0	0
95772	Malignant neoplasm of upper buccal sulcus	0	0	0	0	0	0	0	0	0	0	0	0	2	0	0	0	0	0
95783	Malignant neoplasm of specified site of pancreas NOS	0	0	0	0	0	0	0	0	0	0	0	0	2	0	0	0	0	0
95818	[M]PARAGANGLIOMA, MALIGNANT	0	0	0	0	0	0	0	0	0	0	0	0	2	0	0	0	0	0
96003	MALIGNANT NEOPLASM OF JUNCTION OF HARD AND SOFT PALATE	0	0	0	0	0	0	0	0	0	0	0	0	2	0	0	0	0	0
96094	Siewert type III adenocarcinoma	0	0	0	0	0	0	0	0	0	0	0	0	2	0	0	0	0	0
96226	[X]Malignant neoplasm/overlap lesion/other+ill-defined sites	0	0	0	0	0	0	0	0	0	0	0	0	2	0	0	0	0	0
96231	[M]FIBROXANTHOMA, MALIGNANT	0	0	0	0	0	0	0	0	0	0	0	0	2	0	0	0	0	0
96429	Malignant neoplasm of undescended testis NOS	0	0	0	0	0	0	0	0	0	0	0	0	2	0	0	0	0	0
96445	Malignant neoplasm of turbinate	0	0	0	0	0	0	0	0	0	0	0	0	2	0	0	0	0	0

96585	OVERLAPPING MALIGNANT MELANOMA OF SKIN	0	0	0	0	0	0	0	0	0	0	0	0	2	0	0	0	0	0
96635	Malignant neoplasm of ectopic pancreatic tissue	0	0	0	0	0	0	0	0	0	0	0	0	2	0	0	0	0	0
96782	MALIGNANT NEOPLASM OF LOWER LIP, INNER ASPECT NOS	0	0	0	0	0	0	0	0	0	0	0	0	2	0	0	0	0	0
96783	MALIGNANT NEOPLASM OF COMMISSURE OF LIP	0	0	0	0	0	0	0	0	0	0	0	0	2	0	0	0	0	0
96798	[M]Meningothelial sarcoma	0	0	0	0	0	0	0	0	0	0	0	0	2	0	0	0	0	0
96802	Malignant neoplasm of posterior wall of stomach NEC	0	0	0	0	0	0	0	0	0	0	0	0	2	0	0	0	0	0
96869	MALIGNANT NEOPLASM OF POSTERIOR WALL OF NASOPHARYNX NOS	0	0	0	0	0	0	0	0	0	0	0	0	2	0	0	0	0	0
96971	MALIG NEOP OTHER SITE NASAL CAVITY, MIDDLE EAR AND SINUSES	0	0	0	0	0	0	0	0	0	0	0	0	2	0	0	0	0	0
97332	MALIGNANT NEOPLASM OF LARYNGEAL CARTILAGE NOS	0	0	0	0	0	0	0	0	0	0	0	0	2	0	0	0	0	0
97463	[M]GIANT CELL SARCOMA (EXCEPT OF BONE)	0	0	0	0	0	0	0	0	0	0	0	0	2	0	0	0	0	0
97499	Siewert type II adenocarcinoma	0	0	0	0	0	0	0	0	0	0	0	0	2	0	0	0	0	0
97530	MALIGNANT NEOPLASM OF LOWER BUCCAL SULCUS	0	0	0	0	0	0	0	0	0	0	0	0	2	0	0	0	0	0
97547	MALIGNANT NEOPLASM OF INTRATHORACIC SITE NOS	0	0	0	0	0	0	0	0	0	0	0	0	2	0	0	0	0	0
97593	[M]AMELOBLASTOMA, MALIGNANT	0	0	0	0	0	0	0	0	0	0	0	0	2	0	0	0	0	0
97875	Malignant neoplasm	0	0	0	0	0	0	0	0	0	0	0	0	2	0	0	0	0	0
97961	[M]NEUROEPITHELIOMA NOS	0	0	0	0	0	0	0	0	0	0	0	0	2	0	0	0	0	0
97996	Malignant neoplasm of other site of uterine adnexa	0	0	0	0	0	0	0	0	0	0	0	0	2	0	0	0	0	0
98104	MALIGNANT NEOPLASM OF OTHER SPECIFIED PLEURA	0	0	0	0	0	0	0	0	0	0	0	0	2	0	0	0	0	0
98142	Siewert type I adenocarcinoma	0	0	0	0	0	0	0	0	0	0	0	0	2	0	0	0	0	0
98322	[M]HAEMANGIOENDOTHELIOMA, MALIGNANT	0	0	0	0	0	0	0	0	0	0	0	0	2	0	0	0	0	0
98361	[X]KAPOSI'S SARCOMA OF OTHER SITES	0	0	0	0	0	0	0	0	0	0	0	0	2	0	0	0	0	0
98408	MALIG NEOP OF CONNECTIVE AND SOFT TISSUE OF THORAX NOS	0	0	0	0	0	0	0	0	0	0	0	0	2	0	0	0	0	0
98483	[M]ODONTOGENIC FIBROSARCOMA	0	0	0	0	0	0	0	0	0	0	0	0	2	0	0	0	0	0
98500	Malignant neoplasm of upper lip	0	0	0	0	0	0	0	0	0	0	0	0	2	0	0	0	0	0

98537	MALIGNANT NEOPLASM OF TYMPANIC CAVITY	0	0	0	0	0	0	0	0	0	0	0	0	2	0	0	0	0	0
98559	[M]Chondroblastoma, malignant	0	0	0	0	0	0	0	0	0	0	0	0	2	0	0	0	0	0
98696	[M]Papillary cystadenoma, borderline malignancy	0	0	0	0	0	0	0	0	0	0	0	0	2	0	0	0	0	0
98740	MALIGNANT NEOPLASM OF UPPER LIP, VERMILION BORDER NOS	0	0	0	0	0	0	0	0	0	0	0	0	2	0	0	0	0	0
98781	[M]TRABECULAR ADENOCARCINOMA	0	0	0	0	0	0	0	0	0	0	0	0	2	0	0	0	0	0
98797	[M]EMBRYONAL SARCOMA	0	0	0	0	0	0	0	0	0	0	0	0	2	0	0	0	0	0
98800	[M]PILOID ASTROCYTOMA	0	0	0	0	0	0	0	0	0	0	0	0	2	0	0	0	0	0
98813	Malig neop eyeball excl conjunctiva	0	0	0	0	0	0	0	0	0	0	0	0	2	0	0	0	0	0
98825	[M]MIXED ISLET CELL AND EXOCRINE ADENOCARCINOMA	0	0	0	0	0	0	0	0	0	0	0	0	2	0	0	0	0	0
98883	[M]Medullary carcinoma with lymphoid stroma	0	0	0	0	0	0	0	0	0	0	0	0	2	0	0	0	0	0
98911	MALIGNANT NEOPLASM OF NASAL CONCHAE	0	0	0	0	0	0	0	0	0	0	0	0	2	0	0	0	0	0
99001	MALIGNANT NEOPLASM OF UPPER LIP, FRENULUM	0	0	0	0	0	0	0	0	0	0	0	0	2	0	0	0	0	0
99096	[X]Malignant neopl/overlapping les/resp+intrathoracic organs	0	0	0	0	0	0	0	0	0	0	0	0	2	0	0	0	0	0
99185	Malignant neoplasm of glossopalatine fold	0	0	0	0	0	0	0	0	0	0	0	0	2	0	0	0	0	0
99257	MALIGNANT MELANOMA OF SCALP AND NECK NOS	0	0	0	0	0	0	0	0	0	0	0	0	2	0	0	0	0	0
99386	Malignant neoplasm posterior margin nasal septum and choanae	0	0	0	0	0	0	0	0	0	0	0	0	2	0	0	0	0	0
99491	[M]Neuroepitheliomatous neoplasm NOS	0	0	0	0	0	0	0	0	0	0	0	0	2	0	0	0	0	0
99493	MALIGNANT NEOPLASM OF UPPER LIP, INNER ASPECT	0	0	0	0	0	0	0	0	0	0	0	0	2	0	0	0	0	0
99572	MALIGNANT NEOPLASM OF CONNECTIVE AND SOFT TISSUE OF TOE	0	0	0	0	0	0	0	0	0	0	0	0	2	0	0	0	0	0
99621	Malignant neoplasm of cranial nerves	0	0	0	0	0	0	0	0	0	0	0	0	2	0	0	0	0	0
99665	[M]Juxtacortical osteogenic sarcoma	0	0	0	0	0	0	0	0	0	0	0	0	2	0	0	0	0	0
99797	[M]MALIGNANT GIANT CELL TUMOUR OF SOFT PARTS	0	0	0	0	0	0	0	0	0	0	0	0	2	0	0	0	0	0
99896	MALIGNANT NEOPLASM OF OTHER SPECIFIED SITE SMALL INTESTINE	0	0	0	0	0	0	0	0	0	0	0	0	2	0	0	0	0	0
99913	MALIGNANT NEOPLASM OF GLOBUS PALLIDUS	0	0	0	0	0	0	0	0	0	0	0	0	2	0	0	0	0	0

99931	[V]Personal history of malignant neoplasm of tongue	0	0	0	0	0	0	0	0	0	0	0	0	2	0	0	0	0	0
100002	MALIGNANT NEOPLASM OF TONSILLAR FOSSA NOS	0	0	0	0	0	0	0	0	0	0	0	0	2	0	0	0	0	0
100083	NEUROBLASTOMA	0	0	0	0	0	0	0	0	0	0	0	0	2	0	0	0	0	0
100111	[M]SCHNEIDERIAN CARCINOMA	0	0	0	0	0	0	0	0	0	0	0	0	2	0	0	0	0	0
100144	Malignant neoplasm of lip, oral aspect	0	0	0	0	0	0	0	0	0	0	0	0	2	0	0	0	0	0
100232	Malig neop of other site of heart, thymus and mediastinum	0	0	0	0	0	0	0	0	0	0	0	0	2	0	0	0	0	0
100267	[M]Adamantinoma, malignant	0	0	0	0	0	0	0	0	0	0	0	0	2	0	0	0	0	0
100371	[M]Epithelial nephroblastoma	0	0	0	0	0	0	0	0	0	0	0	0	2	0	0	0	0	0
100584	Malignant neoplasm of cardiac orifice of stomach	0	0	0	0	0	0	0	0	0	0	0	0	2	0	0	0	0	0
100590	[M]Tumour embolus	0	0	0	0	0	0	0	0	0	0	0	0	2	0	0	0	0	0
100625	[M]Carcinoid tumour, nonargentaffin, malignant	0	0	0	0	0	0	0	0	0	0	0	0	2	0	0	0	0	0
100721	Malignant neoplasm of upper lip, inner aspect NOS	0	0	0	0	0	0	0	0	0	0	0	0	2	0	0	0	0	0
100733	Malignant neoplasm of other part of brain NOS	0	0	0	0	0	0	0	0	0	0	0	0	2	0	0	0	0	0
100906	Malignant neoplasm of lip, unspecified, external	0	0	0	0	0	0	0	0	0	0	0	0	2	0	0	0	0	0
100918	MALIGNANT NEOPLASM OF ANTERIOR WALL OF NASOPHARYNX NOS	0	0	0	0	0	0	0	0	0	0	0	0	2	0	0	0	0	0
101086	MALIGNANT NEOPLASM OF CRANIAL NERVES NOS	0	0	0	0	0	0	0	0	0	0	0	0	2	0	0	0	0	0
101095	[M]Grade 1 (Stage pTa) papillary urothelial/transit cell ca	0	0	0	0	0	0	0	0	0	0	0	0	2	0	0	0	0	0
101608	Malignant neoplasm of ureteropelvic junction	0	0	0	0	0	0	0	0	0	0	0	0	2	0	0	0	0	0
101668	[X]Malignant neoplasm/peripheral nerves of trunk,unspecified	0	0	0	0	0	0	0	0	0	0	0	0	2	0	0	0	0	0
101700	HEREDITARY NONPOLYPOSIS COLON CANCER	0	0	0	0	0	0	0	0	0	0	0	0	2	0	0	0	0	0
101707	MALIGNANT NEOPLASM OF LOWER LIP, VERMILION BORDER NOS	0	0	0	0	0	0	0	0	0	0	0	0	2	0	0	0	0	0
101753	MALIGNANT NEOPLASM OF OTHER SITES OF GUM	0	0	0	0	0	0	0	0	0	0	0	0	2	0	0	0	0	0
101778	Malignant neoplasm of broad ligament	0	0	0	0	0	0	0	0	0	0	0	0	2	0	0	0	0	0
101805	Malignant neoplasm of lacrimal sac	0	0	0	0	0	0	0	0	0	0	0	0	2	0	0	0	0	0

101885	Mesothelioma of pericardium	0	0	0	0	0	0	0	0	0	0	0	0	2	0	0	0	0	0
101907	Overlapping malign lesion of retroperitoneum and peritoneum	0	0	0	0	0	0	0	0	0	0	0	0	2	0	0	0	0	0
101923	[M]FIBROLIPOSARCOMA	0	0	0	0	0	0	0	0	0	0	0	0	2	0	0	0	0	0
101978	[M]Grade 3 (Stage pTa) papillary urothelial/transit cell ca	0	0	0	0	0	0	0	0	0	0	0	0	2	0	0	0	0	0
101988	Malignant neoplasm of palatine tonsil	0	0	0	0	0	0	0	0	0	0	0	0	2	0	0	0	0	0
102030	[M]Complex epithelial neoplasm NOS	0	0	0	0	0	0	0	0	0	0	0	0	2	0	0	0	0	0
102116	CLARK MELANOMA LEVEL 4	0	0	0	0	0	0	0	0	0	0	0	0	2	0	0	0	0	0
102142	MALIGNANT NEOPLASM OF ANTERIOR 2/3 OF TONGUE VENTRAL SURFACE	0	0	0	0	0	0	0	0	0	0	0	0	2	0	0	0	0	0
102145	Malignant melanoma of external auditory meatus	0	0	0	0	0	0	0	0	0	0	0	0	2	0	0	0	0	0
102151	Malignant neoplasm of overlapping lesion of tonsil	0	0	0	0	0	0	0	0	0	0	0	0	2	0	0	0	0	0
102205	Malignant neoplasm of lateral wall of nasopharynx NOS	0	0	0	0	0	0	0	0	0	0	0	0	2	0	0	0	0	0
102244	[M]Grade 2 (Stage pTa) papillary urothelial/transit cell ca	0	0	0	0	0	0	0	0	0	0	0	0	2	0	0	0	0	0
102314	H/O: prostate cancer	0	0	0	0	0	0	0	0	0	0	0	0	2	0	0	0	0	0
102356	[M]Polyembryoma	0	0	0	0	0	0	0	0	0	0	0	0	2	0	0	0	0	0
102417	[M]SUPERFICIAL BASAL CELL CARCINOMA	0	0	0	0	0	0	0	0	0	0	0	0	2	0	0	0	0	0
102547	[M]BASAL CELL CARCINOMA, NODULAR	0	0	0	0	0	0	0	0	0	0	0	0	2	0	0	0	0	0
102593	[M]Noninfiltrating intraductal papillary adenocarcinoma	0	0	0	0	0	0	0	0	0	0	0	0	2	0	0	0	0	0
102949	Malignant neoplasm of connective and soft tissue of hip	0	0	0	0	0	0	0	0	0	0	0	0	2	0	0	0	0	0
103034	[M]Endometrioid adenofibroma, malignant	0	0	0	0	0	0	0	0	0	0	0	0	2	0	0	0	0	0
103047	[M]Spongioblastoma NOS	0	0	0	0	0	0	0	0	0	0	0	0	2	0	0	0	0	0
103066	[M]PIGMENTED BASAL CELL CARCINOMA	0	0	0	0	0	0	0	0	0	0	0	0	2	0	0	0	0	0
103100	[V]Personal history of malignant neoplasm of eye	0	0	0	0	0	0	0	0	0	0	0	0	2	0	0	0	0	0
103178	[M]Basal cell carcinoma, infiltrative	0	0	0	0	0	0	0	0	0	0	0	0	2	0	0	0	0	0
103354	MALIGNANT NEOPLASM OF SHORT BONES OF LEG NOS	0	0	0	0	0	0	0	0	0	0	0	0	2	0	0	0	0	0

103440	[M]Basal cell carcinoma, micronodular	0	0	0	0	0	0	0	0	0	0	0	0	2	0	0	0	0	0
103796	Malignant neoplasm of vestibule of mouth	0	0	0	0	0	0	0	0	0	0	0	0	2	0	0	0	0	0
103883	[M]RETINOBLASTOMA, UNDIFFERENTIATED TYPE	0	0	0	0	0	0	0	0	0	0	0	0	2	0	0	0	0	0
103946	MALIGNANT NEOPLASM OF MUCOSA OF TRACHEA	0	0	0	0	0	0	0	0	0	0	0	0	2	0	0	0	0	0
103995	Malignant neoplasm of aortic body or paraganglia NOS	0	0	0	0	0	0	0	0	0	0	0	0	2	0	0	0	0	0
104025	MALIGNANT NEOPLASM OF SKIN OF HEEL	0	0	0	0	0	0	0	0	0	0	0	0	2	0	0	0	0	0
104128	Kaposi's sarcoma of soft tissue	0	0	0	0	0	0	0	0	0	0	0	0	2	0	0	0	0	0
104139	Malig neoplasm of connective and soft tissues of thor spine	0	0	0	0	0	0	0	0	0	0	0	0	2	0	0	0	0	0
104324	MALIGNANT TUMOUR OF UNKNOWN ORIGIN	0	0	0	0	0	0	0	0	0	0	0	0	2	0	0	0	0	0
104609	CLARK MELANOMA LEVEL 2	0	0	0	0	0	0	0	0	0	0	0	0	2	0	0	0	0	0
104684	MULTIPLE SELF-HEALING EPITHELIOMA OF FERGUSON-SMITH	0	0	0	0	0	0	0	0	0	0	0	0	2	0	0	0	0	0
104720	[M]SARCOMATOID MESOTHELIOMA	0	0	0	0	0	0	0	0	0	0	0	0	2	0	0	0	0	0
105072	[X]MAL NEOPLASM/OVERLAP LES/PERIPH NERV+AUTONOMIC NERV SYSTM	0	0	0	0	0	0	0	0	0	0	0	0	2	0	0	0	0	0
105083	Histiocytic sarcoma	0	0	0	0	0	0	0	0	0	0	0	0	2	0	0	0	0	0
105166	[M]Glomoid sarcoma	0	0	0	0	0	0	0	0	0	0	0	0	2	0	0	0	0	0
105275	[M]Periosteal osteogenic sarcoma	0	0	0	0	0	0	0	0	0	0	0	0	2	0	0	0	0	0
105388	LOCAL RECURRENCE OF MALIGNANT TUMOUR OF URINARY BLADDER	0	0	0	0	0	0	0	0	0	0	0	0	2	0	0	0	0	0
105475	MALIGNANT NEOPLASM OF SHORT BONES OF LEG	0	0	0	0	0	0	0	0	0	0	0	0	2	0	0	0	0	0
105488	LOCAL RECURRENCE OF MALIGNANT TUMOUR OF BREAST	0	0	0	0	0	0	0	0	0	0	0	0	2	0	0	0	0	0
105613	MALIGNANT NEOPLASM OF SPHINCTER OF ODDI	0	0	0	0	0	0	0	0	0	0	0	0	2	0	0	0	0	0
105797	MALIGNANT NEOPLASM OF ACROMION	0	0	0	0	0	0	0	0	0	0	0	0	2	0	0	0	0	0
105944	[M]MIXED CELL RHABDOMYOSARCOMA	0	0	0	0	0	0	0	0	0	0	0	0	2	0	0	0	0	0
106069	MALIGNANT NEOPLASM OF CARPAL BONES	0	0	0	0	0	0	0	0	0	0	0	0	2	0	0	0	0	0
106131	[M]OLFACTORY NEUROEPITHELIOMA	0	0	0	0	0	0	0	0	0	0	0	0	2	0	0	0	0	0

106194	MALIGNANT NEOPLASM OF VISCERAL PLEURA	0	0	0	0	0	0	0	0	0	0	0	0	2	0	0	0	0	0
106654	Mal neoplasm/periph nerves+autonomic nervous system,unspc	0	0	0	0	0	0	0	0	0	0	0	0	2	0	0	0	0	0
106889	[M]EMBRYONAL HEPATOMA	0	0	0	0	0	0	0	0	0	0	0	0	2	0	0	0	0	0
106915	MALIGNANT NEOPLASM OF NASOPHARYNGEAL SOFT PALATE SURFACE	0	0	0	0	0	0	0	0	0	0	0	0	2	0	0	0	0	0
107126	MALIGNANT NEOPLASM OF SACROCOCCYGEAL REGION	0	0	0	0	0	0	0	0	0	0	0	0	2	0	0	0	0	0
107258	Malignant neoplasm of midline of tongue	0	0	0	0	0	0	0	0	0	0	0	0	2	0	0	0	0	0
107299	[M]Combined hepatocellular carcinoma and cholangiocarcinoma	0	0	0	0	0	0	0	0	0	0	0	0	2	0	0	0	0	0
108389	Malignant neoplasm soft tissues of cervical spine	0	0	0	0	0	0	0	0	0	0	0	0	2	0	0	0	0	0
108638	Malignant neoplasm of third metacarpal bone	0	0	0	0	0	0	0	0	0	0	0	0	2	0	0	0	0	0
108667	Angiosarcoma of spleen	0	0	0	0	0	0	0	0	0	0	0	0	2	0	0	0	0	0
108682	[M]Germinoblastic sarcoma NOS	0	0	0	0	0	0	0	0	0	0	0	0	2	0	0	0	0	0
108866	Clark melanoma level 5	0	0	0	0	0	0	0	0	0	0	0	0	2	0	0	0	0	0
108922	Wilms' tumour + nephrotic syndrome + pseudohermaphroditism	0	0	0	0	0	0	0	0	0	0	0	0	2	0	0	0	0	0
109002	Malignant melanoma of perianal skin	0	0	0	0	0	0	0	0	0	0	0	0	2	0	0	0	0	0
109429	[V]Personal history of malignant neoplasm of genital organ	0	0	0	0	0	0	0	0	0	0	0	0	2	0	0	0	0	0
109473	Malignant neoplasm of cerebral pia mater	0	0	0	0	0	0	0	0	0	0	0	0	2	0	0	0	0	0
4072	Acute leukaemia NOS	0	0	0	0	0	0	0	0	0	0	0	0	0	2	0	0	0	0
4222	Lymphatic leukaemia	0	0	0	0	0	0	0	0	0	0	0	0	0	2	0	0	0	0
4250	Leukaemia NOS	0	0	0	0	0	0	0	0	0	0	0	0	0	2	0	0	0	0
4251	Acute lymphoid leukaemia	0	0	0	0	0	0	0	0	0	0	0	0	0	2	0	0	0	0
4413	Acute myeloid leukaemia	0	0	0	0	0	0	0	0	0	0	0	0	0	2	0	0	0	0
4637	[M]Leukaemias	0	0	0	0	0	0	0	0	0	0	0	0	0	2	0	0	0	0
5137	Leukaemic reticuloendotheliosis	0	0	0	0	0	0	0	0	0	0	0	0	0	2	0	0	0	0
5915	[M]Hairy cell leukaemia	0	0	0	0	0	0	0	0	0	0	0	0	0	2	0	0	0	0
6316	[M]Acute leukaemia NOS	0	0	0	0	0	0	0	0	0	0	0	0	0	2	0	0	0	0
7176	Myeloid leukaemia	0	0	0	0	0	0	0	0	0	0	0	0	0	2	0	0	0	0

8625	Chronic lymphoid leukaemia	0	0	0	0	0	0	0	0	0	0	0	0	0	2	0	0	0	0
10726	Chronic myeloid leukaemia	0	0	0	0	0	0	0	0	0	0	0	0	0	2	0	0	0	0
12146	[M]Lymphoid leukaemia NOS	0	0	0	0	0	0	0	0	0	0	0	0	0	2	0	0	0	0
16416	Chronic leukaemia NOS	0	0	0	0	0	0	0	0	0	0	0	0	0	2	0	0	0	0
19372	Lymphoid leukaemia	0	0	0	0	0	0	0	0	0	0	0	0	0	2	0	0	0	0
19974	Acute monocytic leukaemia	0	0	0	0	0	0	0	0	0	0	0	0	0	2	0	0	0	0
20440	Myelomonocytic leukaemia	0	0	0	0	0	0	0	0	0	0	0	0	0	2	0	0	0	0
20635	[M]Lymphatic leukaemia	0	0	0	0	0	0	0	0	0	0	0	0	0	2	0	0	0	0
22050	Chronic myelomonocytic leukaemia	0	0	0	0	0	0	0	0	0	0	0	0	0	2	0	0	0	0
22071	[M]Blast cell leukaemia	0	0	0	0	0	0	0	0	0	0	0	0	0	2	0	0	0	0
25191	Leukaemia of unspecified cell type	0	0	0	0	0	0	0	0	0	0	0	0	0	2	0	0	0	0
27330	Leukaemic reticuloendotheliosis	0	0	0	0	0	0	0	0	0	0	0	0	0	2	0	0	0	0
27340	Di Guglielmo's disease	0	0	0	0	0	0	0	0	0	0	0	0	0	2	0	0	0	0
27458	Chronic monocytic leukaemia	0	0	0	0	0	0	0	0	0	0	0	0	0	2	0	0	0	0
27520	Chronic myeloid leukaemia NOS	0	0	0	0	0	0	0	0	0	0	0	0	0	2	0	0	0	0
27664	Acute promyelocytic leukaemia	0	0	0	0	0	0	0	0	0	0	0	0	0	2	0	0	0	0
27790	Chronic lymphatic leukaemia	0	0	0	0	0	0	0	0	0	0	0	0	0	2	0	0	0	0
28276	Acute myelofibrosis	0	0	0	0	0	0	0	0	0	0	0	0	0	2	0	0	0	0
29335	[M]Adult T-cell leukaemia/lymphoma	0	0	0	0	0	0	0	0	0	0	0	0	0	2	0	0	0	0
30632	Other specified leukaemia NOS	0	0	0	0	0	0	0	0	0	0	0	0	0	2	0	0	0	0
31586	Prolymphocytic leukaemia	0	0	0	0	0	0	0	0	0	0	0	0	0	2	0	0	0	0
31701	Chronic granulocytic leukaemia	0	0	0	0	0	0	0	0	0	0	0	0	0	2	0	0	0	0
31750	[M]Chronic leukaemia NOS	0	0	0	0	0	0	0	0	0	0	0	0	0	2	0	0	0	0
33344	Myeloid leukaemia NOS	0	0	0	0	0	0	0	0	0	0	0	0	0	2	0	0	0	0

34692	Other leukaemia of unspecified cell type	0	0	0	0	0	0	0	0	0	0	0	0	0	2	0	0	0	0
35697	[M]Myeloid leukaemias	0	0	0	0	0	0	0	0	0	0	0	0	0	2	0	0	0	0
35875	Monocytic leukaemia	0	0	0	0	0	0	0	0	0	0	0	0	0	2	0	0	0	0
37272	Other specified leukaemia	0	0	0	0	0	0	0	0	0	0	0	0	0	2	0	0	0	0
37410	[M]Acute lymphoid leukaemia	0	0	0	0	0	0	0	0	0	0	0	0	0	2	0	0	0	0
37461	Adult T-cell leukaemia	0	0	0	0	0	0	0	0	0	0	0	0	0	2	0	0	0	0
37487	[M]Acute myelofibrosis	0	0	0	0	0	0	0	0	0	0	0	0	0	2	0	0	0	0
37723	[M]Granulocytic leukaemia NOS	0	0	0	0	0	0	0	0	0	0	0	0	0	2	0	0	0	0
38331	Other lymphoid leukaemia NOS	0	0	0	0	0	0	0	0	0	0	0	0	0	2	0	0	0	0
38914	Lymphoid leukaemia NOS	0	0	0	0	0	0	0	0	0	0	0	0	0	2	0	0	0	0
39629	Granulocytic sarcoma	0	0	0	0	0	0	0	0	0	0	0	0	0	2	0	0	0	0
40420	[M]Leukaemias unspecified	0	0	0	0	0	0	0	0	0	0	0	0	0	2	0	0	0	0
41500	[M]Chronic lymphoid leukaemia	0	0	0	0	0	0	0	0	0	0	0	0	0	2	0	0	0	0
41734	[M]Leukaemia NOS	0	0	0	0	0	0	0	0	0	0	0	0	0	2	0	0	0	0
42297	[M]Leukaemia NOS	0	0	0	0	0	0	0	0	0	0	0	0	0	2	0	0	0	0
42539	Acute erythraemia and erythroleukaemia	0	0	0	0	0	0	0	0	0	0	0	0	0	2	0	0	0	0
44420	Refractory anaemia with excess of blasts with transformation	0	0	0	0	0	0	0	0	0	0	0	0	0	2	0	0	0	0
46048	[M]Prolymphocytic leukaemia	0	0	0	0	0	0	0	0	0	0	0	0	0	2	0	0	0	0
46263	[M]Acute myelomonocytic leukaemia	0	0	0	0	0	0	0	0	0	0	0	0	0	2	0	0	0	0
46444	[M]Erythroleukaemias	0	0	0	0	0	0	0	0	0	0	0	0	0	2	0	0	0	0
48049	[M]Chronic myelomonocytic leukaemia	0	0	0	0	0	0	0	0	0	0	0	0	0	2	0	0	0	0
48155	[M]Lymphoid leukaemias	0	0	0	0	0	0	0	0	0	0	0	0	0	2	0	0	0	0
49327	[M]Acute megakaryoblastic leukaemia	0	0	0	0	0	0	0	0	0	0	0	0	0	2	0	0	0	0
49725	Other lymphoid leukaemia	0	0	0	0	0	0	0	0	0	0	0	0	0	2	0	0	0	0

50858	Acute panmyelosis	0	0	0	0	0	0	0	0	0	0	0	0	0	2	0	0	0	0
50928	[M]Burkitt's cell leukaemia	0	0	0	0	0	0	0	0	0	0	0	0	0	2	0	0	0	0
52327	Chloroma	0	0	0	0	0	0	0	0	0	0	0	0	0	2	0	0	0	0
52942	[M]Chronic myeloid leukaemia	0	0	0	0	0	0	0	0	0	0	0	0	0	2	0	0	0	0
54585	[M]Acute myeloid leukaemia	0	0	0	0	0	0	0	0	0	0	0	0	0	2	0	0	0	0
54793	Subacute leukaemia NOS	0	0	0	0	0	0	0	0	0	0	0	0	0	2	0	0	0	0
57316	[M]Acute promyelocytic leukaemia	0	0	0	0	0	0	0	0	0	0	0	0	0	2	0	0	0	0
57671	Megakaryocytic leukaemia	0	0	0	0	0	0	0	0	0	0	0	0	0	2	0	0	0	0
57713	[M]Eosinophilic leukaemias	0	0	0	0	0	0	0	0	0	0	0	0	0	2	0	0	0	0
59929	[M]Leukaemia unspecified, NOS	0	0	0	0	0	0	0	0	0	0	0	0	0	2	0	0	0	0
61500	Acute myelomonocytic leukaemia	0	0	0	0	0	0	0	0	0	0	0	0	0	2	0	0	0	0
61693	[X]Other myeloid leukaemia	0	0	0	0	0	0	0	0	0	0	0	0	0	2	0	0	0	0
62330	[M]Other myeloid leukaemia NOS	0	0	0	0	0	0	0	0	0	0	0	0	0	2	0	0	0	0
63475	Subacute myeloid leukaemia	0	0	0	0	0	0	0	0	0	0	0	0	0	2	0	0	0	0
63570	[M]Stem cell leukaemia	0	0	0	0	0	0	0	0	0	0	0	0	0	2	0	0	0	0
64963	[M]Blastic leukaemia	0	0	0	0	0	0	0	0	0	0	0	0	0	2	0	0	0	0
65122	Leukaemic reticuloendotheliosis of unspecified sites	0	0	0	0	0	0	0	0	0	0	0	0	0	2	0	0	0	0
65123	Leukaemic reticuloend of intra-abdominal lymph nodes	0	0	0	0	0	0	0	0	0	0	0	0	0	2	0	0	0	0
65165	[X]Other leukaemia of unspecified cell type	0	0	0	0	0	0	0	0	0	0	0	0	0	2	0	0	0	0
65721	Mast cell leukaemia	0	0	0	0	0	0	0	0	0	0	0	0	0	2	0	0	0	0
65777	Thrombocytic leukaemia	0	0	0	0	0	0	0	0	0	0	0	0	0	2	0	0	0	0
66089	Other myeloid leukaemia NOS	0	0	0	0	0	0	0	0	0	0	0	0	0	2	0	0	0	0
66694	[M]Naegeli-type monocytic leukaemia	0	0	0	0	0	0	0	0	0	0	0	0	0	2	0	0	0	0
67029	[X]Other lymphoid leukaemia	0	0	0	0	0	0	0	0	0	0	0	0	0	2	0	0	0	0

67700	Monoblastic leukaemia	0	0	0	0	0	0	0	0	0	0	0	0	0	2	0	0	0	0
69299	[M]Thrombocytic leukaemia	0	0	0	0	0	0	0	0	0	0	0	0	0	2	0	0	0	0
70724	Myeloid sarcoma	0	0	0	0	0	0	0	0	0	0	0	0	0	2	0	0	0	0
70935	[M]Erythroleukaemia	0	0	0	0	0	0	0	0	0	0	0	0	0	2	0	0	0	0
71377	[M]Eosinophilic leukaemia	0	0	0	0	0	0	0	0	0	0	0	0	0	2	0	0	0	0
71850	[M]Myeloid leukaemia NOS	0	0	0	0	0	0	0	0	0	0	0	0	0	2	0	0	0	0
72179	[M]Subacute leukaemia NOS	0	0	0	0	0	0	0	0	0	0	0	0	0	2	0	0	0	0
72197	Lymphosarcoma cell leukaemia	0	0	0	0	0	0	0	0	0	0	0	0	0	2	0	0	0	0
72222	[M]Megakaryocytic leukaemia	0	0	0	0	0	0	0	0	0	0	0	0	0	2	0	0	0	0
72310	[M]Aleukaemic leukaemia NOS	0	0	0	0	0	0	0	0	0	0	0	0	0	2	0	0	0	0
72774	Subacute lymphoid leukaemia	0	0	0	0	0	0	0	0	0	0	0	0	0	2	0	0	0	0
73066	[M]Miscellaneous leukaemias	0	0	0	0	0	0	0	0	0	0	0	0	0	2	0	0	0	0
73088	[M]Monocytic leukaemia NOS	0	0	0	0	0	0	0	0	0	0	0	0	0	2	0	0	0	0
73777	Leukaemic reticuloendotheliosis NOS	0	0	0	0	0	0	0	0	0	0	0	0	0	2	0	0	0	0
87335	Hairy cell leukaemia	0	0	0	0	0	0	0	0	0	0	0	0	0	2	0	0	0	0
89329	[X]Other specified leukaemias	0	0	0	0	0	0	0	0	0	0	0	0	0	2	0	0	0	0
89762	[X]Other monocytic leukaemia	0	0	0	0	0	0	0	0	0	0	0	0	0	2	0	0	0	0
93342	Monocytic leukaemia NOS	0	0	0	0	0	0	0	0	0	0	0	0	0	2	0	0	0	0
93944	[M]Chloroma	0	0	0	0	0	0	0	0	0	0	0	0	0	2	0	0	0	0
94174	Other and unspecified leukaemia	0	0	0	0	0	0	0	0	0	0	0	0	0	2	0	0	0	0
96893	[M]Myeloid sarcoma	0	0	0	0	0	0	0	0	0	0	0	0	0	2	0	0	0	0
98009	[M]Granulocytic sarcoma	0	0	0	0	0	0	0	0	0	0	0	0	0	2	0	0	0	0
99015	Other monocytic leukaemia	0	0	0	0	0	0	0	0	0	0	0	0	0	2	0	0	0	0
99413	Other and unspecified leukaemia NOS	0	0	0	0	0	0	0	0	0	0	0	0	0	2	0	0	0	0

100786	Chronic eosinophilic leukaemia	0	0	0	0	0	0	0	0	0	0	0	0	0	2	0	0	0	0
100927	[M]Erythroleukaemia NOS	0	0	0	0	0	0	0	0	0	0	0	0	0	2	0	0	0	0
101271	[M]Acute panmyelosis	0	0	0	0	0	0	0	0	0	0	0	0	0	2	0	0	0	0
101606	Subacute monocytic leukaemia	0	0	0	0	0	0	0	0	0	0	0	0	0	2	0	0	0	0
102764	[M]Acute panmyelosis	0	0	0	0	0	0	0	0	0	0	0	0	0	2	0	0	0	0
102783	Chronic neutrophilic leukaemia	0	0	0	0	0	0	0	0	0	0	0	0	0	2	0	0	0	0
104325	B-cell acute lymphoblastic leukaemia	0	0	0	0	0	0	0	0	0	0	0	0	0	2	0	0	0	0
104328	B-cell chronic lymphocytic leukaemia	0	0	0	0	0	0	0	0	0	0	0	0	0	2	0	0	0	0
104475	Subacute myelomonocytic leukaemia	0	0	0	0	0	0	0	0	0	0	0	0	0	2	0	0	0	0
104788	Acute myeloblastic leukaemia	0	0	0	0	0	0	0	0	0	0	0	0	0	2	0	0	0	0
104939	Adult T-cell lymphoma/leukaemia (HTLV-1-associated)	0	0	0	0	0	0	0	0	0	0	0	0	0	2	0	0	0	0
105069	Juvenile myelomonocytic leukaemia	0	0	0	0	0	0	0	0	0	0	0	0	0	2	0	0	0	0
105957	Chronic myeloid leukaemia, BCR/ABL positive	0	0	0	0	0	0	0	0	0	0	0	0	0	2	0	0	0	0
106197	[M]Basophilic leukaemia	0	0	0	0	0	0	0	0	0	0	0	0	0	2	0	0	0	0
106483	[M]Subacute myeloid leukaemia	0	0	0	0	0	0	0	0	0	0	0	0	0	2	0	0	0	0
106924	Clinical stage B chronic lymphocytic leukaemia	0	0	0	0	0	0	0	0	0	0	0	0	0	2	0	0	0	0
107017	Chronic lymphocytic leukaemia of B-cell type	0	0	0	0	0	0	0	0	0	0	0	0	0	2	0	0	0	0
107052	Clinical stage A chronic lymphocytic leukaemia	0	0	0	0	0	0	0	0	0	0	0	0	0	2	0	0	0	0
107163	Clinical stage C chronic lymphocytic leukaemia	0	0	0	0	0	0	0	0	0	0	0	0	0	2	0	0	0	0
107236	Atypical chronic myeloid leukaemia, BCR/ABL negative	0	0	0	0	0	0	0	0	0	0	0	0	0	2	0	0	0	0
107643	T-cell prolymphocytic leukaemia	0	0	0	0	0	0	0	0	0	0	0	0	0	2	0	0	0	0
107773	[M]Eosinophilic leukaemia NOS	0	0	0	0	0	0	0	0	0	0	0	0	0	2	0	0	0	0
108316	[M]Miscellaneous leukaemia NOS	0	0	0	0	0	0	0	0	0	0	0	0	0	2	0	0	0	0
108424	Acute monoblastic leukaemia	0	0	0	0	0	0	0	0	0	0	0	0	0	2	0	0	0	0

108656	B-cell prolymphocytic leukaemia	0	0	0	0	0	0	0	0	0	0	0	0	0	2	0	0	0	0
108715	Histiocytic leukaemia	0	0	0	0	0	0	0	0	0	0	0	0	0	2	0	0	0	0
108964	[M]Juvenile myelomonocytic leukaemia	0	0	0	0	0	0	0	0	0	0	0	0	0	2	0	0	0	0
17177	H/O: * leukaemia	0	0	0	0	0	0	0	0	0	0	0	0	0	2	0	0	0	0
36693	[V]Personal history of leukaemia	0	0	0	0	0	0	0	0	0	0	0	0	0	2	0	0	0	0
53477	[V]Follow-up examination after chemotherapy for leukaemia	0	0	0	0	0	0	0	0	0	0	0	0	0	2	0	0	0	0
94597	[V]Personal history of lymphoid leukaemia	0	0	0	0	0	0	0	0	0	0	0	0	0	2	0	0	0	0
103645	H/O: * other lymph/haematopoi.	0	0	0	0	0	0	0	0	0	0	0	0	0	2	0	0	0	0
3672	[M]Myeloma NOS	0	0	0	0	0	0	0	0	0	0	0	0	0	0	2	0	0	0
4944	Multiple myeloma	0	0	0	0	0	0	0	0	0	0	0	0	0	0	2	0	0	0
15211	Myelomatosis	0	0	0	0	0	0	0	0	0	0	0	0	0	0	2	0	0	0
18744	[M]Multiple myeloma	0	0	0	0	0	0	0	0	0	0	0	0	0	0	2	0	0	0
19028	Solitary myeloma	0	0	0	0	0	0	0	0	0	0	0	0	0	0	2	0	0	0
21329	Plasmacytoma NOS	0	0	0	0	0	0	0	0	0	0	0	0	0	0	2	0	0	0
22158	Malignant plasma cell neoplasm, extramedullary plasmacytoma	0	0	0	0	0	0	0	0	0	0	0	0	0	0	2	0	0	0
31671	[M]Plasma cell myeloma	0	0	0	0	0	0	0	0	0	0	0	0	0	0	2	0	0	0
39187	Plasma cell leukaemia	0	0	0	0	0	0	0	0	0	0	0	0	0	0	2	0	0	0
39490	[M]Plasmacytic myeloma	0	0	0	0	0	0	0	0	0	0	0	0	0	0	2	0	0	0
43312	Myeloma - solitary	0	0	0	0	0	0	0	0	0	0	0	0	0	0	2	0	0	0
43552	Kahler's disease	0	0	0	0	0	0	0	0	0	0	0	0	0	0	2	0	0	0
46042	Lambda light chain myeloma	0	0	0	0	0	0	0	0	0	0	0	0	0	0	2	0	0	0
52946	Bone marrow: myeloma cells	0	0	0	0	0	0	0	0	0	0	0	0	0	0	2	0	0	0
53647	[M]Myelomatosis	0	0	0	0	0	0	0	0	0	0	0	0	0	0	2	0	0	0
59663	Neoplasm of uncertain behaviour of plasma cells	0	0	0	0	0	0	0	0	0	0	0	0	0	0	2	0	0	0

60433	Osteoporosis in multiple myelomatosis	0	0	0	0	0	0	0	0	0	0	0	0	0	0	2	0	0	0
63864	[M]Plasmacytoma NOS	0	0	0	0	0	0	0	0	0	0	0	0	0	0	2	0	0	0
64618	[M]Plasma cell leukaemias	0	0	0	0	0	0	0	0	0	0	0	0	0	0	2	0	0	0
73135	[M]Solitary myeloma	0	0	0	0	0	0	0	0	0	0	0	0	0	0	2	0	0	0
99702	[M]Plasma cell tumour, malignant	0	0	0	0	0	0	0	0	0	0	0	0	0	0	2	0	0	0
102164	[M]Monostotic myeloma	0	0	0	0	0	0	0	0	0	0	0	0	0	0	2	0	0	0
104418	Solitary plasmacytoma	0	0	0	0	0	0	0	0	0	0	0	0	0	0	2	0	0	0
38321	Plasmacytoma NOS	0	0	0	0	0	0	0	0	0	0	0	0	0	0	2	0	0	0
37182	Multiple myeloma and immunoproliferative neoplasms	0	0	0	0	0	0	0	0	0	0	0	0	0	0	2	0	0	0
43450	Immunoproliferative neoplasm or myeloma NOS	0	0	0	0	0	0	0	0	0	0	0	0	0	0	2	0	0	0
40561	[V]Personal history of Hodgkin's disease	0	0	0	0	0	0	0	0	0	0	0	0	0	0	2	0	0	0
72204	[V]Personal history other lymphatic/haematopoietic neoplasm	0	0	0	0	0	0	0	0	0	0	0	0	0	0	2	0	0	0
51182	H/O: * other lymph/haematopoi.	0	0	0	0	0	0	0	0	0	0	0	0	0	0	2	0	0	0
1481	Reticulosarcoma	0	0	0	0	0	0	0	0	0	0	0	0	0	0	2	0	0	0
1483	[M]Lymphoma NOS	0	0	0	0	0	0	0	0	0	0	0	0	0	0	2	0	0	0
2462	Hodgkin's disease	0	0	0	0	0	0	0	0	0	0	0	0	0	0	2	0	0	0
3371	[M]Non Hodgkins lymphoma	0	0	0	0	0	0	0	0	0	0	0	0	0	0	2	0	0	0
3604	Non - Hodgkin's lymphoma	0	0	0	0	0	0	0	0	0	0	0	0	0	0	2	0	0	0
3845	Eosinophilic granuloma	0	0	0	0	0	0	0	0	0	0	0	0	0	0	2	0	0	0
4870	Histiocytosis X (acute, progressive)	0	0	0	0	0	0	0	0	0	0	0	0	0	0	2	0	0	0
5179	Nodular lymphoma (Brill - Symmers disease)	0	0	0	0	0	0	0	0	0	0	0	0	0	0	2	0	0	0
7940	[X]Non-Hodgkin's lymphoma NOS	0	0	0	0	0	0	0	0	0	0	0	0	0	0	2	0	0	0
8649	[X]Non-Hodgkin's lymphoma, unspecified type	0	0	0	0	0	0	0	0	0	0	0	0	0	0	2	0	0	0
9172	[M]Waldenstrom's macroglobulinaemia	0	0	0	0	0	0	0	0	0	0	0	0	0	0	2	0	0	0
10411	Waldenstrom's macroglobulinaemia	0	0	0	0	0	0	0	0	0	0	0	0	0	0	2	0	0	0
12006	Mycosis fungoides	0	0	0	0	0	0	0	0	0	0	0	0	0	0	2	0	0	0

12323	Malignant neoplasm of lymphatic and haemopoietic tissue	0	0	0	0	0	0	0	0	0	0	0	0	0	0	2	0	0	0
12335	Malignant lymphoma NOS	0	0	0	0	0	0	0	0	0	0	0	0	0	0	2	0	0	0
12464	Peripheral T-cell lymphoma	0	0	0	0	0	0	0	0	0	0	0	0	0	0	2	0	0	0
15027	Malignant lymphoma NOS	0	0	0	0	0	0	0	0	0	0	0	0	0	0	2	0	0	0
15036	Malignant mast cell tumours	0	0	0	0	0	0	0	0	0	0	0	0	0	0	2	0	0	0
15504	Malignant lymphoma NOS of lymph nodes of multiple sites	0	0	0	0	0	0	0	0	0	0	0	0	0	0	2	0	0	0
16460	[M]Malignant lymphoma, non Hodgkin's type	0	0	0	0	0	0	0	0	0	0	0	0	0	0	2	0	0	0
16527	Macroglobulinaemia	0	0	0	0	0	0	0	0	0	0	0	0	0	0	2	0	0	0
16774	[M] Cutaneous lymphoma	0	0	0	0	0	0	0	0	0	0	0	0	0	0	2	0	0	0
17178	[M]Lymphomas, NOS or diffuse	0	0	0	0	0	0	0	0	0	0	0	0	0	0	2	0	0	0
17182	Follicular lymphoma NOS	0	0	0	0	0	0	0	0	0	0	0	0	0	0	2	0	0	0
17460	Diffuse non-Hodgkin's lymphoblastic (diffuse) lymphoma	0	0	0	0	0	0	0	0	0	0	0	0	0	0	2	0	0	0
17887	Malignant lymphoma otherwise specified	0	0	0	0	0	0	0	0	0	0	0	0	0	0	2	0	0	0
18383	[M] Large cell lymphoma	0	0	0	0	0	0	0	0	0	0	0	0	0	0	2	0	0	0
19140	Hodgkin's nodular sclerosis of lymph nodes of multiple sites	0	0	0	0	0	0	0	0	0	0	0	0	0	0	2	0	0	0
20437	[M]Lymphomas, nodular or follicular	0	0	0	0	0	0	0	0	0	0	0	0	0	0	2	0	0	0
20710	[M]Hodgkin's disease	0	0	0	0	0	0	0	0	0	0	0	0	0	0	2	0	0	0
21402	Burkitt's lymphoma	0	0	0	0	0	0	0	0	0	0	0	0	0	0	2	0	0	0
21463	[M]Lymphocytic lymphoma NOS	0	0	0	0	0	0	0	0	0	0	0	0	0	0	2	0	0	0
21549	Follicular non-Hodgkin's lymphoma	0	0	0	0	0	0	0	0	0	0	0	0	0	0	2	0	0	0
23711	[M]Malignant lymphoma, diffuse NOS	0	0	0	0	0	0	0	0	0	0	0	0	0	0	2	0	0	0
26111	Lymphomatoid papulosis	0	0	0	0	0	0	0	0	0	0	0	0	0	0	2	0	0	0
26135	[M] Alpha heavy chain disease	0	0	0	0	0	0	0	0	0	0	0	0	0	0	2	0	0	0
27416	Lymphosarcoma	0	0	0	0	0	0	0	0	0	0	0	0	0	0	2	0	0	0

27562	[M]Follicular lymphosarcoma NOS	0	0	0	0	0	0	0	0	0	0	0	0	0	0	2	0	0	0
27965	[M]AngiocentricT-cell lymphoma	0	0	0	0	0	0	0	0	0	0	0	0	0	0	2	0	0	0
28639	Follicular non-Hodgkin's small cleaved cell lymphoma	0	0	0	0	0	0	0	0	0	0	0	0	0	0	2	0	0	0
29178	Hodgkin's disease, nodular sclerosis	0	0	0	0	0	0	0	0	0	0	0	0	0	0	2	0	0	0
29876	Hodgkin's, lymphocytic-histiocytic predominance NOS	0	0	0	0	0	0	0	0	0	0	0	0	0	0	2	0	0	0
30646	Malignant neoplasm lymphatic or haematopoietic tissue OS	0	0	0	0	0	0	0	0	0	0	0	0	0	0	2	0	0	0
31324	Mast cell malignancy of lymph nodes of multiple sites	0	0	0	0	0	0	0	0	0	0	0	0	0	0	2	0	0	0
31492	[M] Monocytoid B-cell lymphoma	0	0	0	0	0	0	0	0	0	0	0	0	0	0	2	0	0	0
31537	[M]Hodgkin,s disease, lymphocytic predominance, nodular	0	0	0	0	0	0	0	0	0	0	0	0	0	0	2	0	0	0
31576	Other types of follicular non-Hodgkin's lymphoma	0	0	0	0	0	0	0	0	0	0	0	0	0	0	2	0	0	0
31726	[M]Malignant lymphoma, small cleaved cell, diffuse	0	0	0	0	0	0	0	0	0	0	0	0	0	0	2	0	0	0
31741	[M]Hodgkin,s disease, nodular sclerosis, lymphocytic deplet	0	0	0	0	0	0	0	0	0	0	0	0	0	0	2	0	0	0
31749	[M]Monocytoid B-cell lymphoma	0	0	0	0	0	0	0	0	0	0	0	0	0	0	2	0	0	0
31794	Unspecified B-cell non-Hodgkin's lymphoma	0	0	0	0	0	0	0	0	0	0	0	0	0	0	2	0	0	0
32240	Lymphoma stage III	0	0	0	0	0	0	0	0	0	0	0	0	0	0	2	0	0	0
33333	Other malignant neoplasm of lymphoid and histiocytic tissue	0	0	0	0	0	0	0	0	0	0	0	0	0	0	2	0	0	0
33869	[M]Malignant lymphoma, large cell, diffuse NOS	0	0	0	0	0	0	0	0	0	0	0	0	0	0	2	0	0	0
34089	Malignant lymphoma NOS of lymph nodes of axilla and arm	0	0	0	0	0	0	0	0	0	0	0	0	0	0	2	0	0	0
34352	[M]Lymphoblastic lymphoma NOS	0	0	0	0	0	0	0	0	0	0	0	0	0	0	2	0	0	0
34926	Letterer-Siwe disease	0	0	0	0	0	0	0	0	0	0	0	0	0	0	2	0	0	0
35014	Sezary's disease	0	0	0	0	0	0	0	0	0	0	0	0	0	0	2	0	0	0
36114	[M]Malignant lymphoma NOS	0	0	0	0	0	0	0	0	0	0	0	0	0	0	2	0	0	0
36736	Histiocytosis X , unspecified	0	0	0	0	0	0	0	0	0	0	0	0	0	0	2	0	0	0
37112	Malignant neoplasm of histiocytic tissue	0	0	0	0	0	0	0	0	0	0	0	0	0	0	2	0	0	0

38005	Mycosis fungoides NOS	0	0	0	0	0	0	0	0	0	0	0	0	0	0	2	0	0	0
38939	Hodgkin's disease, lymphocytic-histiocytic predominance	0	0	0	0	0	0	0	0	0	0	0	0	0	0	2	0	0	0
39798	Diffuse non-Hodgkin's lymphoma, unspecified	0	0	0	0	0	0	0	0	0	0	0	0	0	0	2	0	0	0
39883	[M]Malig lymp, follicular centre cell, cleaved, follicular	0	0	0	0	0	0	0	0	0	0	0	0	0	0	2	0	0	0
39906	[M]Malignant lymphoma, centrocytic	0	0	0	0	0	0	0	0	0	0	0	0	0	0	2	0	0	0
40000	Langerhans' cell histiocytosis	0	0	0	0	0	0	0	0	0	0	0	0	0	0	2	0	0	0
40508	[M]Hodgkin,s disease, nodular sclerosis, lymphocytic predom	0	0	0	0	0	0	0	0	0	0	0	0	0	0	2	0	0	0
40513	[M]Lymphoma, nodular or follicular NOS	0	0	0	0	0	0	0	0	0	0	0	0	0	0	2	0	0	0
40740	[X]Malignant neoplasms of lymphoid, haematopoietic and rela	0	0	0	0	0	0	0	0	0	0	0	0	0	0	2	0	0	0
40766	[M] Peripheral T-cell lymphoma NOS	0	0	0	0	0	0	0	0	0	0	0	0	0	0	2	0	0	0
40991	Lymphoma staging system	0	0	0	0	0	0	0	0	0	0	0	0	0	0	2	0	0	0
41369	Lymphosarcoma and reticulosarcoma	0	0	0	0	0	0	0	0	0	0	0	0	0	0	2	0	0	0
41754	[M]Malignant lymphoma, lymphoplasmacytoid type	0	0	0	0	0	0	0	0	0	0	0	0	0	0	2	0	0	0
41841	[M]Malignant lymphoma, follicular centre cell NOS	0	0	0	0	0	0	0	0	0	0	0	0	0	0	2	0	0	0
42198	[M]Hodgkin's disease, nodular sclerosis NOS	0	0	0	0	0	0	0	0	0	0	0	0	0	0	2	0	0	0
42461	Hodgkin's disease NOS	0	0	0	0	0	0	0	0	0	0	0	0	0	0	2	0	0	0
42579	Malignant lymphoma NOS of intra-abdominal lymph nodes	0	0	0	0	0	0	0	0	0	0	0	0	0	0	2	0	0	0
42769	[M]Hodgkin's disease NOS	0	0	0	0	0	0	0	0	0	0	0	0	0	0	2	0	0	0
43415	[X]Other Hodgkin's disease	0	0	0	0	0	0	0	0	0	0	0	0	0	0	2	0	0	0
44196	Hodgkin's granuloma	0	0	0	0	0	0	0	0	0	0	0	0	0	0	2	0	0	0
44267	Malignant histiocytosis	0	0	0	0	0	0	0	0	0	0	0	0	0	0	2	0	0	0
44318	Oth and unspecif peripheral & cutaneous T-cell lymphomas	0	0	0	0	0	0	0	0	0	0	0	0	0	0	2	0	0	0
45264	Nodular lymphoma of lymph nodes of head, face and neck	0	0	0	0	0	0	0	0	0	0	0	0	0	0	2	0	0	0
45768	[M]Acute progressive histiocytosis X	0	0	0	0	0	0	0	0	0	0	0	0	0	0	2	0	0	0

46877	[M]Malignant lymphoma, small lymphocytic NOS	0	0	0	0	0	0	0	0	0	0	0	0	0	0	2	0	0	0
46931	[M]Malignant lymphoma, stem cell type	0	0	0	0	0	0	0	0	0	0	0	0	0	0	2	0	0	0
46967	[M]Mycosis fungoides	0	0	0	0	0	0	0	0	0	0	0	0	0	0	2	0	0	0
47204	Letterer-Siwe disease NOS	0	0	0	0	0	0	0	0	0	0	0	0	0	0	2	0	0	0
47330	[M]HISTIOCYTIC MEDULLARY RETICULOSIS	0	0	0	0	0	0	0	0	0	0	0	0	0	0	2	0	0	0
48253	[M]Malignant lymphoma, immunoblastic type	0	0	0	0	0	0	0	0	0	0	0	0	0	0	2	0	0	0
49253	[M]Giant follicular lymphoma	0	0	0	0	0	0	0	0	0	0	0	0	0	0	2	0	0	0
49262	Follicular non-Hodgkin's large cell lymphoma	0	0	0	0	0	0	0	0	0	0	0	0	0	0	2	0	0	0
49301	Malignant neoplasm lymphatic or haematopoietic tissue NOS	0	0	0	0	0	0	0	0	0	0	0	0	0	0	2	0	0	0
49605	Hodgkin's disease, mixed cellularity	0	0	0	0	0	0	0	0	0	0	0	0	0	0	2	0	0	0
49825	[M]Reticulum cell sarcoma NOS	0	0	0	0	0	0	0	0	0	0	0	0	0	0	2	0	0	0
50668	Diffuse non-Hodgkin's small cell (diffuse) lymphoma	0	0	0	0	0	0	0	0	0	0	0	0	0	0	2	0	0	0
50695	Diffuse non-Hodgkin mixed sml & lge cell (diffuse) lymphoma	0	0	0	0	0	0	0	0	0	0	0	0	0	0	2	0	0	0
50696	Malignant lymphoma NOS of lymph nodes of head, face and neck	0	0	0	0	0	0	0	0	0	0	0	0	0	0	2	0	0	0
51285	[M]Hodgkin's disease, mixed cellularity	0	0	0	0	0	0	0	0	0	0	0	0	0	0	2	0	0	0
51680	[M]Malignant lymphoma, small cell, noncleaved, diffuse	0	0	0	0	0	0	0	0	0	0	0	0	0	0	2	0	0	0
51718	Histiocytosis X , chronic	0	0	0	0	0	0	0	0	0	0	0	0	0	0	2	0	0	0
51852	[M]Malig lymphoma, lymphocytic, intermediate different NOS	0	0	0	0	0	0	0	0	0	0	0	0	0	0	2	0	0	0
51895	[M]Lymphoma, diffuse or NOS	0	0	0	0	0	0	0	0	0	0	0	0	0	0	2	0	0	0
52591	[M]LYMPHOBLASTOMA NOS	0	0	0	0	0	0	0	0	0	0	0	0	0	0	2	0	0	0
52593	[M] Gamma heavy chain disease	0	0	0	0	0	0	0	0	0	0	0	0	0	0	2	0	0	0
53397	Hodgkin's disease NOS	0	0	0	0	0	0	0	0	0	0	0	0	0	0	2	0	0	0
53551	Diffuse non-Hodgkin's immunoblastic (diffuse) lymphoma	0	0	0	0	0	0	0	0	0	0	0	0	0	0	2	0	0	0
54083	Letterer-Siwe disease of lymph nodes of multiple sites	0	0	0	0	0	0	0	0	0	0	0	0	0	0	2	0	0	0

54190	[M] Angioimmunoblastic lymphadenopathy	0	0	0	0	0	0	0	0	0	0	0	0	0	0	2	0	0	0
54411	Hand - Schuller - Christian disease	0	0	0	0	0	0	0	0	0	0	0	0	0	0	2	0	0	0
55303	Hodgkin's nodular sclerosis of head, face and neck	0	0	0	0	0	0	0	0	0	0	0	0	0	0	2	0	0	0
56041	[M]Hodgkin's disease, lymphocytic predominance	0	0	0	0	0	0	0	0	0	0	0	0	0	0	2	0	0	0
57225	Hodgkin's disease, nodular sclerosis of unspecified site	0	0	0	0	0	0	0	0	0	0	0	0	0	0	2	0	0	0
57427	Malignant lymphoma NOS of unspecified site	0	0	0	0	0	0	0	0	0	0	0	0	0	0	2	0	0	0
57509	Waldenstrom's hypergammaglobulinaemic purpura	0	0	0	0	0	0	0	0	0	0	0	0	0	0	2	0	0	0
57544	[M]True histiocytic lymphoma	0	0	0	0	0	0	0	0	0	0	0	0	0	0	2	0	0	0
57737	Lymphoepithelioid lymphoma	0	0	0	0	0	0	0	0	0	0	0	0	0	0	2	0	0	0
58015	[M]Malignant lymphomatous polyposis	0	0	0	0	0	0	0	0	0	0	0	0	0	0	2	0	0	0
58082	Nodular lymphoma of lymph nodes of multiple sites	0	0	0	0	0	0	0	0	0	0	0	0	0	0	2	0	0	0
58684	Hodgkin's mixed cellularity of intrathoracic lymph nodes	0	0	0	0	0	0	0	0	0	0	0	0	0	0	2	0	0	0
58871	Malignant histiocytosis NOS	0	0	0	0	0	0	0	0	0	0	0	0	0	0	2	0	0	0
58953	[M]Malig lymp,follicular centre cell,noncleaved,follicular	0	0	0	0	0	0	0	0	0	0	0	0	0	0	2	0	0	0
58962	Malignant immunoproliferative small intestinal disease	0	0	0	0	0	0	0	0	0	0	0	0	0	0	2	0	0	0
59115	Burkitt's lymphoma of lymph nodes of head, face and neck	0	0	0	0	0	0	0	0	0	0	0	0	0	0	2	0	0	0
59593	[M]Letterer - Siwe disease	0	0	0	0	0	0	0	0	0	0	0	0	0	0	2	0	0	0
59755	Hodgkin's disease NOS of intrathoracic lymph nodes	0	0	0	0	0	0	0	0	0	0	0	0	0	0	2	0	0	0
59778	Hodgkin's disease NOS of lymph nodes of head, face and neck	0	0	0	0	0	0	0	0	0	0	0	0	0	0	2	0	0	0
60092	Malignant lymphoma NOS of spleen	0	0	0	0	0	0	0	0	0	0	0	0	0	0	2	0	0	0
60242	Reticulosarcoma of unspecified site	0	0	0	0	0	0	0	0	0	0	0	0	0	0	2	0	0	0
60275	[M]Malignant lymphoma, centroblastic type NOS	0	0	0	0	0	0	0	0	0	0	0	0	0	0	2	0	0	0
60504	[M]Lymphocytic lymphosarcoma NOS	0	0	0	0	0	0	0	0	0	0	0	0	0	0	2	0	0	0
60918	Lymphoma stage I	0	0	0	0	0	0	0	0	0	0	0	0	0	0	2	0	0	0

61149	Hodgkin's nodular sclerosis of intra-abdominal lymph nodes	0	0	0	0	0	0	0	0	0	0	0	0	0	0	2	0	0	0
61251	[M]Malign lymphoma,lymphocytic,intermediate differn, diffuse	0	0	0	0	0	0	0	0	0	0	0	0	0	0	2	0	0	0
61662	Hodgkin's disease NOS, unspecified site	0	0	0	0	0	0	0	0	0	0	0	0	0	0	2	0	0	0
61997	[M]Hodgkin's disease NOS	0	0	0	0	0	0	0	0	0	0	0	0	0	0	2	0	0	0
62380	Lymphosarcoma of intrathoracic lymph nodes	0	0	0	0	0	0	0	0	0	0	0	0	0	0	2	0	0	0
62437	Malignant reticulosis	0	0	0	0	0	0	0	0	0	0	0	0	0	0	2	0	0	0
63054	Hodgkin's disease, nodular sclerosis NOS	0	0	0	0	0	0	0	0	0	0	0	0	0	0	2	0	0	0
63105	Malignant lymphoma NOS of lymph node inguinal region and leg	0	0	0	0	0	0	0	0	0	0	0	0	0	0	2	0	0	0
63239	[M]Malignant histiocytosis	0	0	0	0	0	0	0	0	0	0	0	0	0	0	2	0	0	0
63375	[X]Unspecified B-cell non-Hodgkin's lymphoma	0	0	0	0	0	0	0	0	0	0	0	0	0	0	2	0	0	0
63625	Hodgkin's lymphocytic depletion lymph nodes axilla and arm	0	0	0	0	0	0	0	0	0	0	0	0	0	0	2	0	0	0
63699	[M]Malignant lymphoma, nodular NOS	0	0	0	0	0	0	0	0	0	0	0	0	0	0	2	0	0	0
63723	Lymphosarcoma NOS	0	0	0	0	0	0	0	0	0	0	0	0	0	0	2	0	0	0
63973	[M]Microglioma	0	0	0	0	0	0	0	0	0	0	0	0	0	0	2	0	0	0
63994	[M]Malignant lymphoma, large cell, cleaved, diffuse	0	0	0	0	0	0	0	0	0	0	0	0	0	0	2	0	0	0
64036	Hodgkin's sarcoma	0	0	0	0	0	0	0	0	0	0	0	0	0	0	2	0	0	0
64336	[X]Other specified types of non-Hodgkin's lymphoma	0	0	0	0	0	0	0	0	0	0	0	0	0	0	2	0	0	0
64343	[M]Hodgkin,s disease, nodular sclerosis, mixed cellularity	0	0	0	0	0	0	0	0	0	0	0	0	0	0	2	0	0	0
64427	Unspec malig neop lymphoid/histiocytic lymph node head/neck	0	0	0	0	0	0	0	0	0	0	0	0	0	0	2	0	0	0
64515	[X]Diffuse non-Hodgkin's lymphoma, unspecified	0	0	0	0	0	0	0	0	0	0	0	0	0	0	2	0	0	0
64670	Lymphosarcoma of intra-abdominal lymph nodes	0	0	0	0	0	0	0	0	0	0	0	0	0	0	2	0	0	0
64947	[M]Brill - Symmers' disease	0	0	0	0	0	0	0	0	0	0	0	0	0	0	2	0	0	0
65180	Diffuse non-Hodgkin's lymphoma undifferentiated (diffuse)	0	0	0	0	0	0	0	0	0	0	0	0	0	0	2	0	0	0

65434	Malignant neoplasms of lymphoid and histiocytic tissue NOS	0	0	0	0	0	0	0	0	0	0	0	0	0	0	2	0	0	0
65483	Hodgkin's nodular sclerosis of lymph nodes of axilla and arm	0	0	0	0	0	0	0	0	0	0	0	0	0	0	2	0	0	0
65489	Hodgkin's paragranuloma	0	0	0	0	0	0	0	0	0	0	0	0	0	0	2	0	0	0
65584	[M]Hodgkin,s disease, lymphocytic predominance, diffuse	0	0	0	0	0	0	0	0	0	0	0	0	0	0	2	0	0	0
65642	Malignant histiocytosis of intra-abdominal lymph nodes	0	0	0	0	0	0	0	0	0	0	0	0	0	0	2	0	0	0
65701	Nodular lymphoma NOS	0	0	0	0	0	0	0	0	0	0	0	0	0	0	2	0	0	0
66327	Nodular lymphoma of unspecified site	0	0	0	0	0	0	0	0	0	0	0	0	0	0	2	0	0	0
66603	[M]Malig lymphoma, follicular centre cell, non-cleaved NOS	0	0	0	0	0	0	0	0	0	0	0	0	0	0	2	0	0	0
67203	[M]Lymphoblastic lymphosarcoma NOS	0	0	0	0	0	0	0	0	0	0	0	0	0	0	2	0	0	0
67339	[M]MALIGNANT MASTOCYTOSIS	0	0	0	0	0	0	0	0	0	0	0	0	0	0	2	0	0	0
67506	Hodgkin's nodular sclerosis of intrathoracic lymph nodes	0	0	0	0	0	0	0	0	0	0	0	0	0	0	2	0	0	0
67518	[X]Other types of follicular non-Hodgkin's lymphoma	0	0	0	0	0	0	0	0	0	0	0	0	0	0	2	0	0	0
67703	Hodgkin's disease, lymphocytic depletion	0	0	0	0	0	0	0	0	0	0	0	0	0	0	2	0	0	0
68039	Hodgkin's sarcoma of lymph nodes of axilla and upper limb	0	0	0	0	0	0	0	0	0	0	0	0	0	0	2	0	0	0
68330	Hodgkin's, lymphocytic-histiocytic pred of head, face, neck	0	0	0	0	0	0	0	0	0	0	0	0	0	0	2	0	0	0
68964	[M]Malignant lymphoma, centroblastic-centrocytic, diffuse	0	0	0	0	0	0	0	0	0	0	0	0	0	0	2	0	0	0
69301	[M]Malignant lymphoma, convoluted cell type NOS	0	0	0	0	0	0	0	0	0	0	0	0	0	0	2	0	0	0
69497	Malignant histiocytosis of unspecified site	0	0	0	0	0	0	0	0	0	0	0	0	0	0	2	0	0	0
69980	[M]Malignant lymphoma, lymphocytic, well differentiated NOS	0	0	0	0	0	0	0	0	0	0	0	0	0	0	2	0	0	0
70374	Reticulosarcoma of intra-abdominal lymph nodes	0	0	0	0	0	0	0	0	0	0	0	0	0	0	2	0	0	0
70509	Diffuse non-Hodgkin's centroblastic lymphoma	0	0	0	0	0	0	0	0	0	0	0	0	0	0	2	0	0	0
70716	Immunoproliferative neoplasm	0	0	0	0	0	0	0	0	0	0	0	0	0	0	2	0	0	0
70740	[M]Malignant reticulosis	0	0	0	0	0	0	0	0	0	0	0	0	0	0	2	0	0	0
70842	Follicular non-Hodg mixed sml cleavd & lge cell lymphoma	0	0	0	0	0	0	0	0	0	0	0	0	0	0	2	0	0	0

71031	Reticulosarcoma of lymph nodes of head, face and neck	0	0	0	0	0	0	0	0	0	0	0	0	0	0	2	0	0	0
71117	[M]Malignant lymphoma, undifferentiated cell type NOS	0	0	0	0	0	0	0	0	0	0	0	0	0	0	2	0	0	0
71142	Hodgkin's, lymphocytic-histiocytic predominance unspec site	0	0	0	0	0	0	0	0	0	0	0	0	0	0	2	0	0	0
71238	Lymphosarcoma of lymph nodes of head, face and neck	0	0	0	0	0	0	0	0	0	0	0	0	0	0	2	0	0	0
71262	Malignant lymphoma NOS of intrapelvic lymph nodes	0	0	0	0	0	0	0	0	0	0	0	0	0	0	2	0	0	0
71304	Burkitt's lymphoma NOS	0	0	0	0	0	0	0	0	0	0	0	0	0	0	2	0	0	0
71609	Unspec malig neop lymphoid/histiocytic nodes inguinal/leg	0	0	0	0	0	0	0	0	0	0	0	0	0	0	2	0	0	0
71619	[M]Malignant lymphoma, large cell, noncleaved, diffuse	0	0	0	0	0	0	0	0	0	0	0	0	0	0	2	0	0	0
71625	Lymphosarcoma of unspecified site	0	0	0	0	0	0	0	0	0	0	0	0	0	0	2	0	0	0
71652	[M]Malignant lymphoma, mixed small and large cell, diffuse	0	0	0	0	0	0	0	0	0	0	0	0	0	0	2	0	0	0
71672	Lymphoma stage IV	0	0	0	0	0	0	0	0	0	0	0	0	0	0	2	0	0	0
71994	Macroglobulinaemia NOS	0	0	0	0	0	0	0	0	0	0	0	0	0	0	2	0	0	0
72196	[M]Malignant lymphoma, lymphocytic, poorly different NOS	0	0	0	0	0	0	0	0	0	0	0	0	0	0	2	0	0	0
72241	[M]Prolymphocytic lymphosarcoma	0	0	0	0	0	0	0	0	0	0	0	0	0	0	2	0	0	0
72433	[M]Reticulosarcoma NOS	0	0	0	0	0	0	0	0	0	0	0	0	0	0	2	0	0	0
72500	[X]Mal neoplasm/lymphoid,haematopoietic+related tissu,unspcf	0	0	0	0	0	0	0	0	0	0	0	0	0	0	2	0	0	0
72714	Mycosis fungoides of lymph nodes of inguinal region and leg	0	0	0	0	0	0	0	0	0	0	0	0	0	0	2	0	0	0
72725	Malignant lymphoma NOS of intrathoracic lymph nodes	0	0	0	0	0	0	0	0	0	0	0	0	0	0	2	0	0	0
73532	Hodgkin's, lymphocytic-histiocytic pred intra-abdominal node	0	0	0	0	0	0	0	0	0	0	0	0	0	0	2	0	0	0
89230	[M]Hodgkin's granuloma	0	0	0	0	0	0	0	0	0	0	0	0	0	0	2	0	0	0
89657	Malignant mast cell tumour NOS	0	0	0	0	0	0	0	0	0	0	0	0	0	0	2	0	0	0
90201	T-zone lymphoma	0	0	0	0	0	0	0	0	0	0	0	0	0	0	2	0	0	0
91674	Mycosis fungoides of intra-abdominal lymph nodes	0	0	0	0	0	0	0	0	0	0	0	0	0	0	2	0	0	0

91900	Hodgkin's disease NOS of lymph nodes of axilla and arm	0	0	0	0	0	0	0	0	0	0	0	0	0	0	2	0	0	0
92068	Nodular lymphoma of intra-abdominal lymph nodes	0	0	0	0	0	0	0	0	0	0	0	0	0	0	2	0	0	0
92245	Hodgkin's, lymphocytic-histiocytic pred intrathoracic nodes	0	0	0	0	0	0	0	0	0	0	0	0	0	0	2	0	0	0
92380	Burkitt's lymphoma of lymph nodes of inguinal region and leg	0	0	0	0	0	0	0	0	0	0	0	0	0	0	2	0	0	0
93384	Unspec malig neop lymphoid/histiocytic of intrathoracic node	0	0	0	0	0	0	0	0	0	0	0	0	0	0	2	0	0	0
93951	Hodgkin's, lymphocytic-histiocytic pred inguinal and leg	0	0	0	0	0	0	0	0	0	0	0	0	0	0	2	0	0	0
94005	Hodgkin's disease, mixed cellularity NOS	0	0	0	0	0	0	0	0	0	0	0	0	0	0	2	0	0	0
94239	[M]Mast cell sarcoma	0	0	0	0	0	0	0	0	0	0	0	0	0	0	2	0	0	0
94279	Hodgkin's disease NOS of spleen	0	0	0	0	0	0	0	0	0	0	0	0	0	0	2	0	0	0
94407	Hodgkin's mixed cellularity of lymph nodes head, face, neck	0	0	0	0	0	0	0	0	0	0	0	0	0	0	2	0	0	0
94415	Malignant histiocytosis of lymph nodes head, face and neck	0	0	0	0	0	0	0	0	0	0	0	0	0	0	2	0	0	0
94935	Lymphoma stage II	0	0	0	0	0	0	0	0	0	0	0	0	0	0	2	0	0	0
94995	Nodular lymphoma of lymph nodes of inguinal region and leg	0	0	0	0	0	0	0	0	0	0	0	0	0	0	2	0	0	0
95012	Mycosis fungoides of lymph nodes of multiple sites	0	0	0	0	0	0	0	0	0	0	0	0	0	0	2	0	0	0
95049	Hodgkin's lymphocytic depletion of unspecified site	0	0	0	0	0	0	0	0	0	0	0	0	0	0	2	0	0	0
95058	Reticulosarcoma of spleen	0	0	0	0	0	0	0	0	0	0	0	0	0	0	2	0	0	0
95338	Hodgkin's, lymphocytic-histiocytic pred intrapelvic nodes	0	0	0	0	0	0	0	0	0	0	0	0	0	0	2	0	0	0
95464	[M]Mycosis fungoides	0	0	0	0	0	0	0	0	0	0	0	0	0	0	2	0	0	0
95545	Maltoma	0	0	0	0	0	0	0	0	0	0	0	0	0	0	2	0	0	0
95630	True histiocytic lymphoma	0	0	0	0	0	0	0	0	0	0	0	0	0	0	2	0	0	0
95715	Mucosa-associated lymphoma	0	0	0	0	0	0	0	0	0	0	0	0	0	0	2	0	0	0
95792	Lymphoid and histiocytic malignancy NOS	0	0	0	0	0	0	0	0	0	0	0	0	0	0	2	0	0	0
95949	Mycosis fungoides of unspecified site	0	0	0	0	0	0	0	0	0	0	0	0	0	0	2	0	0	0
96183	[M]Hodgkin's disease,lymphocytic depletion,diffuse fibrosis	0	0	0	0	0	0	0	0	0	0	0	0	0	0	2	0	0	0

96379	Mycosis fungoides of lymph nodes of axilla and upper limb	0	0	0	0	0	0	0	0	0	0	0	0	0	0	2	0	0	0
97577	Burkitt's lymphoma of intra-abdominal lymph nodes	0	0	0	0	0	0	0	0	0	0	0	0	0	0	2	0	0	0
97746	Hodgkin's disease NOS of lymph nodes of multiple sites	0	0	0	0	0	0	0	0	0	0	0	0	0	0	2	0	0	0
97756	[M]Sezary's disease	0	0	0	0	0	0	0	0	0	0	0	0	0	0	2	0	0	0
97852	[M]Malignant lymphoma, centroblastic type, follicular	0	0	0	0	0	0	0	0	0	0	0	0	0	0	2	0	0	0
97863	Hodgkin's disease, mixed cellularity of unspecified site	0	0	0	0	0	0	0	0	0	0	0	0	0	0	2	0	0	0
98596	[X]Other types of diffuse non-Hodgkin's lymphoma	0	0	0	0	0	0	0	0	0	0	0	0	0	0	2	0	0	0
98840	Hodgkin's paragranuloma of intra-abdominal lymph nodes	0	0	0	0	0	0	0	0	0	0	0	0	0	0	2	0	0	0
98909	Hodgkin's granuloma of lymph nodes of head, face and neck	0	0	0	0	0	0	0	0	0	0	0	0	0	0	2	0	0	0
98961	[M]Malignant lymphoma, centroblastic-centrocytic, follicular	0	0	0	0	0	0	0	0	0	0	0	0	0	0	2	0	0	0
99012	Hodgkin's disease NOS of lymph nodes inguinal region and leg	0	0	0	0	0	0	0	0	0	0	0	0	0	0	2	0	0	0
99067	Gamma heavy chain disease	0	0	0	0	0	0	0	0	0	0	0	0	0	0	2	0	0	0
99200	[M]Hodgkin's disease, nodular sclerosis, cellular phase	0	0	0	0	0	0	0	0	0	0	0	0	0	0	2	0	0	0
99240	Reticulosarcoma NOS	0	0	0	0	0	0	0	0	0	0	0	0	0	0	2	0	0	0
99655	[M]Lymphosarcoma NOS	0	0	0	0	0	0	0	0	0	0	0	0	0	0	2	0	0	0
99695	[M]Mycosis fungoides NOS	0	0	0	0	0	0	0	0	0	0	0	0	0	0	2	0	0	0
99887	Other specified reticulosarcoma or lymphosarcoma	0	0	0	0	0	0	0	0	0	0	0	0	0	0	2	0	0	0
99951	Reticulosarcoma or lymphosarcoma NOS	0	0	0	0	0	0	0	0	0	0	0	0	0	0	2	0	0	0
100006	Burkitt's lymphoma of intrathoracic lymph nodes	0	0	0	0	0	0	0	0	0	0	0	0	0	0	2	0	0	0
100352	Lymphosarcoma of lymph nodes of inguinal region and leg	0	0	0	0	0	0	0	0	0	0	0	0	0	0	2	0	0	0
100423	Hodgkin's paragranuloma of lymph nodes of head, face, neck	0	0	0	0	0	0	0	0	0	0	0	0	0	0	2	0	0	0
100532	Sezary's disease NOS	0	0	0	0	0	0	0	0	0	0	0	0	0	0	2	0	0	0
100544	[M]Reticulosarcoma, nodular	0	0	0	0	0	0	0	0	0	0	0	0	0	0	2	0	0	0
100615	Mast cell malignancy of lymph nodes inguinal region and leg	0	0	0	0	0	0	0	0	0	0	0	0	0	0	2	0	0	0

101114	Diffuse non-Hodgkin's large cell lymphoma	0	0	0	0	0	0	0	0	0	0	0	0	0	0	2	0	0	0
101350	Alpha heavy chain disease	0	0	0	0	0	0	0	0	0	0	0	0	0	0	2	0	0	0
101429	[M]Lymphogranuloma, malignant	0	0	0	0	0	0	0	0	0	0	0	0	0	0	2	0	0	0
101465	Unspec malig neop lymphoid/histiocytic of multiple sites	0	0	0	0	0	0	0	0	0	0	0	0	0	0	2	0	0	0
101530	Hodgkin's disease, lymphocytic depletion NOS	0	0	0	0	0	0	0	0	0	0	0	0	0	0	2	0	0	0
101715	Hodgkin's disease, lymphocytic depletion of spleen	0	0	0	0	0	0	0	0	0	0	0	0	0	0	2	0	0	0
102158	Letterer-Siwe disease of intrathoracic lymph nodes	0	0	0	0	0	0	0	0	0	0	0	0	0	0	2	0	0	0
102594	Diffuse large B-cell lymphoma	0	0	0	0	0	0	0	0	0	0	0	0	0	0	2	0	0	0
102688	[X]Other malignant immunoproliferative diseases	0	0	0	0	0	0	0	0	0	0	0	0	0	0	2	0	0	0
102715	LETTERER-SIWE DISEASE OF UNSPECIFIED SITES	0	0	0	0	0	0	0	0	0	0	0	0	0	0	2	0	0	0
103245	Lymphosarcoma of spleen	0	0	0	0	0	0	0	0	0	0	0	0	0	0	2	0	0	0
103353	Unspec malig neop lymphoid/histiocytic intra-abdominal nodes	0	0	0	0	0	0	0	0	0	0	0	0	0	0	2	0	0	0
103900	Mast cell malignancy of unspecified site	0	0	0	0	0	0	0	0	0	0	0	0	0	0	2	0	0	0
104152	Follicular lymphoma	0	0	0	0	0	0	0	0	0	0	0	0	0	0	2	0	0	0
104291	Hodgkin lymphoma	0	0	0	0	0	0	0	0	0	0	0	0	0	0	2	0	0	0
104386	Small cell B-cell lymphoma	0	0	0	0	0	0	0	0	0	0	0	0	0	0	2	0	0	0
104391	Non-Hodgkin lymphoma	0	0	0	0	0	0	0	0	0	0	0	0	0	0	2	0	0	0
104412	Lymphoblastic (diffuse) lymphoma	0	0	0	0	0	0	0	0	0	0	0	0	0	0	2	0	0	0
104484	Other classical Hodgkin lymphoma	0	0	0	0	0	0	0	0	0	0	0	0	0	0	2	0	0	0
104620	Mantle cell lymphoma	0	0	0	0	0	0	0	0	0	0	0	0	0	0	2	0	0	0
104743	Hodgkin's, lymphocytic-histiocytic pred of multiple sites	0	0	0	0	0	0	0	0	0	0	0	0	0	0	2	0	0	0
104790	Lymphosarcoma of lymph nodes of multiple sites	0	0	0	0	0	0	0	0	0	0	0	0	0	0	2	0	0	0
104862	Cutaneous T-cell lymphoma	0	0	0	0	0	0	0	0	0	0	0	0	0	0	2	0	0	0
104895	Nodular lymphocyte predominant Hodgkin lymphoma	0	0	0	0	0	0	0	0	0	0	0	0	0	0	2	0	0	0

104934	Other mature T/NK-cell lymphoma	0	0	0	0	0	0	0	0	0	0	0	0	0	0	2	0	0	0
105020	Follicular lymphoma grade 3a	0	0	0	0	0	0	0	0	0	0	0	0	0	0	2	0	0	0
105025	[X]OTH SPCF MAL NEOPLSM/LYMPHOID,HAEMATOPOIETIC+RLTD TISSUE	0	0	0	0	0	0	0	0	0	0	0	0	0	0	2	0	0	0
105038	Mediastinal (thymic) large B-cell lymphoma	0	0	0	0	0	0	0	0	0	0	0	0	0	0	2	0	0	0
105085	T/NK-cell lymphoma	0	0	0	0	0	0	0	0	0	0	0	0	0	0	2	0	0	0
105095	Follicular lymphoma grade 2	0	0	0	0	0	0	0	0	0	0	0	0	0	0	2	0	0	0
105203	Nodular lymphoma of intrathoracic lymph nodes	0	0	0	0	0	0	0	0	0	0	0	0	0	0	2	0	0	0
105375	Blastic NK-cell lymphoma	0	0	0	0	0	0	0	0	0	0	0	0	0	0	2	0	0	0
105472	Hodgkin's disease, nodular sclerosis of spleen	0	0	0	0	0	0	0	0	0	0	0	0	0	0	2	0	0	0
105559	Anaplastic large cell lymphoma, ALK-positive	0	0	0	0	0	0	0	0	0	0	0	0	0	0	2	0	0	0
105636	Angioimmunoblastic T-cell lymphoma	0	0	0	0	0	0	0	0	0	0	0	0	0	0	2	0	0	0
105709	Enteropathy-associated T-cell lymphoma	0	0	0	0	0	0	0	0	0	0	0	0	0	0	2	0	0	0
105762	UNIFOCAL LANGERHANS-CELL HISTIOCYTOSIS	0	0	0	0	0	0	0	0	0	0	0	0	0	0	2	0	0	0
105841	Nodular sclerosis classical Hodgkin lymphoma	0	0	0	0	0	0	0	0	0	0	0	0	0	0	2	0	0	0
105889	Follicular lymphoma grade 1	0	0	0	0	0	0	0	0	0	0	0	0	0	0	2	0	0	0
105925	Subcutaneous panniculitic T-cell lymphoma	0	0	0	0	0	0	0	0	0	0	0	0	0	0	2	0	0	0
105955	Anaplastic large cell lymphoma, ALK-negative	0	0	0	0	0	0	0	0	0	0	0	0	0	0	2	0	0	0
105966	EXTRANOD MARG ZONE B-CELL LYMPHOM MUCOSA-ASSOC LYMPHOID TISS	0	0	0	0	0	0	0	0	0	0	0	0	0	0	2	0	0	0
106063	Other types of follicular lymphoma	0	0	0	0	0	0	0	0	0	0	0	0	0	0	2	0	0	0
106137	[M]RETICULOSARCOMAS	0	0	0	0	0	0	0	0	0	0	0	0	0	0	2	0	0	0
106349	Hodgkin lymphoma NOS	0	0	0	0	0	0	0	0	0	0	0	0	0	0	2	0	0	0
106597	Lymphocyte-rich classical Hodgkin lymphoma	0	0	0	0	0	0	0	0	0	0	0	0	0	0	2	0	0	0
106867	Non-follicular lymphoma	0	0	0	0	0	0	0	0	0	0	0	0	0	0	2	0	0	0

106884	Nonfollicular lymphoma	0	0	0	0	0	0	0	0	0	0	0	0	0	0	2	0	0	0
106911	Hodgkin's, lymphocytic-histiocytic predominance of spleen	0	0	0	0	0	0	0	0	0	0	0	0	0	0	2	0	0	0
106969	Diffuse follicle centre lymphoma	0	0	0	0	0	0	0	0	0	0	0	0	0	0	2	0	0	0
106970	[M]Malig lymphoma, lymphocytic, well differentiated,nodular	0	0	0	0	0	0	0	0	0	0	0	0	0	0	2	0	0	0
107032	Hodgkin's lymphocytic depletion lymph nodes multiple sites	0	0	0	0	0	0	0	0	0	0	0	0	0	0	2	0	0	0
107166	Follicular lymphoma grade 3	0	0	0	0	0	0	0	0	0	0	0	0	0	0	2	0	0	0
107804	Hodgkin's disease NOS of intra-abdominal lymph nodes	0	0	0	0	0	0	0	0	0	0	0	0	0	0	2	0	0	0
107949	Hepatosplenic T-cell lymphoma	0	0	0	0	0	0	0	0	0	0	0	0	0	0	2	0	0	0
107973	Follicular lymphoma grade 3b	0	0	0	0	0	0	0	0	0	0	0	0	0	0	2	0	0	0
108037	Unspec malig neop lymphoid/histiocytic of unspecified site	0	0	0	0	0	0	0	0	0	0	0	0	0	0	2	0	0	0
108102	Heavy chain disease	0	0	0	0	0	0	0	0	0	0	0	0	0	0	2	0	0	0
108182	Diffuse non-Hodgkin's small cleaved cell (diffuse) lymphoma	0	0	0	0	0	0	0	0	0	0	0	0	0	0	2	0	0	0
108235	Waldenstrom macroglobulinaemia	0	0	0	0	0	0	0	0	0	0	0	0	0	0	2	0	0	0
108719	Cutaneous follicle centre lymphoma	0	0	0	0	0	0	0	0	0	0	0	0	0	0	2	0	0	0
108775	Mixed cellularity classical Hodgkin lymphoma	0	0	0	0	0	0	0	0	0	0	0	0	0	0	2	0	0	0
108886	Hodgkin's mixed cellularity of lymph nodes inguinal and leg	0	0	0	0	0	0	0	0	0	0	0	0	0	0	2	0	0	0
109342	Unspec malig neop lymphoid/histiocytic of intrapelvic nodes	0	0	0	0	0	0	0	0	0	0	0	0	0	0	2	0	0	0
109714	[X]Oth and unspecif peripheral & cutaneous T-cell lymphomas	0	0	0	0	0	0	0	0	0	0	0	0	0	0	2	0	0	0
109780	Extranodal NK/T-cell lymphoma, nasal type	0	0	0	0	0	0	0	0	0	0	0	0	0	0	2	0	0	0
1641	Oesophageal varices	0	0	0	0	0	0	0	0	0	0	0	0	0	0	0	3	0	0
5129	Portal hypertension	0	0	0	0	0	0	0	0	0	0	0	0	0	0	0	3	0	0
6690	Acute hepatic failure	0	0	0	0	0	0	0	0	0	0	0	0	0	0	0	3	0	0
6692	Liver transplant failure and rejection	0	0	0	0	0	0	0	0	0	0	0	0	0	0	0	3	0	0
8363	Oesophageal varices in alcoholic cirrhosis of the liver	0	0	0	0	0	0	0	0	0	0	0	0	0	0	0	3	0	0
9026	[V]Liver transplanted	0	0	0	0	0	0	0	0	0	0	0	0	0	0	0	3	0	0

10636	Hepatorenal syndrome	0	0	0	0	0	0	0	0	0	0	0	0	0	0	0	3	0	0
10721	Hepatorenal syndrome as a complication of care	0	0	0	0	0	0	0	0	0	0	0	0	0	0	0	3	0	0
10797	Oesophageal varices NOS	0	0	0	0	0	0	0	0	0	0	0	0	0	0	0	3	0	0
11960	Fibreoptic endoscopic injection sclerotherapy oesoph varices	0	0	0	0	0	0	0	0	0	0	0	0	0	0	0	3	0	0
16062	Hepatic failure	0	0	0	0	0	0	0	0	0	0	0	0	0	0	0	3	0	0
16759	Fibreoptic endoscopic banding of oesophageal varices	0	0	0	0	0	0	0	0	0	0	0	0	0	0	0	3	0	0
17222	Liver failure as a complication of care	0	0	0	0	0	0	0	0	0	0	0	0	0	0	0	3	0	0
17330	Alcoholic hepatic failure	0	0	0	0	0	0	0	0	0	0	0	0	0	0	0	3	0	0
20233	Open operation on oesophageal varices NOS	0	0	0	0	0	0	0	0	0	0	0	0	0	0	0	3	0	0
20912	Open injection sclerotherapy to oesophageal varices	0	0	0	0	0	0	0	0	0	0	0	0	0	0	0	3	0	0
21769	[X] Liver failure	0	0	0	0	0	0	0	0	0	0	0	0	0	0	0	3	0	0
22168	Subacute hepatitis - noninfective	0	0	0	0	0	0	0	0	0	0	0	0	0	0	0	3	0	0
22411	Encephalopathy - hepatic	0	0	0	0	0	0	0	0	0	0	0	0	0	0	0	3	0	0
23511	Hepatic coma	0	0	0	0	0	0	0	0	0	0	0	0	0	0	0	3	0	0
23775	Liver failure NOS	0	0	0	0	0	0	0	0	0	0	0	0	0	0	0	3	0	0
24220	Open operations on oesophageal varices	0	0	0	0	0	0	0	0	0	0	0	0	0	0	0	3	0	0
24901	[X] Hepatic failure	0	0	0	0	0	0	0	0	0	0	0	0	0	0	0	3	0	0
24989	Oesophageal varices with bleeding	0	0	0	0	0	0	0	0	0	0	0	0	0	0	0	3	0	0
25700	[V]Assessment for liver transplant	0	0	0	0	0	0	0	0	0	0	0	0	0	0	0	3	0	0
26319	Oesophageal varices in cirrhosis of the liver	0	0	0	0	0	0	0	0	0	0	0	0	0	0	0	3	0	0
26490	Subacute hepatic failure	0	0	0	0	0	0	0	0	0	0	0	0	0	0	0	3	0	0
28929	Gastric varices	0	0	0	0	0	0	0	0	0	0	0	0	0	0	0	3	0	0
31997	Liver transplant with complication, without blame	0	0	0	0	0	0	0	0	0	0	0	0	0	0	0	3	0	0
36194	Hepatic failure as a complication of care	0	0	0	0	0	0	0	0	0	0	0	0	0	0	0	3	0	0

39945	Acute liver failure	0	0	0	0	0	0	0	0	0	0	0	0	0	0	0	3	0	0
41386	Acute hepatic failure due to drugs	0	0	0	0	0	0	0	0	0	0	0	0	0	0	0	3	0	0
41480	Acute necrosis of liver	0	0	0	0	0	0	0	0	0	0	0	0	0	0	0	3	0	0
43404	Local ligation of oesophageal varices	0	0	0	0	0	0	0	0	0	0	0	0	0	0	0	3	0	0
44424	Oesophageal varices in diseases EC	0	0	0	0	0	0	0	0	0	0	0	0	0	0	0	3	0	0
46647	Rigid oesophagoscopic banding of oesophageal varices	0	0	0	0	0	0	0	0	0	0	0	0	0	0	0	3	0	0
47214	Rigid oesophagoscopic injection sclerotherapy oesoph varices	0	0	0	0	0	0	0	0	0	0	0	0	0	0	0	3	0	0
47861	Exploration of liver transplant	0	0	0	0	0	0	0	0	0	0	0	0	0	0	0	3	0	0
48488	Acute and subacute liver necrosis	0	0	0	0	0	0	0	0	0	0	0	0	0	0	0	3	0	0
55637	Acute necrosis of liver NOS	0	0	0	0	0	0	0	0	0	0	0	0	0	0	0	3	0	0
55962	Viral hepatitis A with coma	0	0	0	0	0	0	0	0	0	0	0	0	0	0	0	3	0	0
56070	Hepatic failure NOS	0	0	0	0	0	0	0	0	0	0	0	0	0	0	0	3	0	0
57324	Subacute necrosis of liver	0	0	0	0	0	0	0	0	0	0	0	0	0	0	0	3	0	0
62038	Tanner devascularisation for bleeding varices	0	0	0	0	0	0	0	0	0	0	0	0	0	0	0	3	0	0
62582	Oesophageal varices in diseases EC NOS	0	0	0	0	0	0	0	0	0	0	0	0	0	0	0	3	0	0
65050	Viral hepatitis C with coma	0	0	0	0	0	0	0	0	0	0	0	0	0	0	0	3	0	0
65067	Acute and subacute liver necrosis NOS	0	0	0	0	0	0	0	0	0	0	0	0	0	0	0	3	0	0
69053	Viral hepatitis B with coma	0	0	0	0	0	0	0	0	0	0	0	0	0	0	0	3	0	0
69194	Replacement of previous liver transplant	0	0	0	0	0	0	0	0	0	0	0	0	0	0	0	3	0	0
69367	Subacute necrosis of liver NOS	0	0	0	0	0	0	0	0	0	0	0	0	0	0	0	3	0	0
69552	Other specified viral hepatitis with coma	0	0	0	0	0	0	0	0	0	0	0	0	0	0	0	3	0	0
71240	Transjugular intrahepatic creation of portosystemic shunt	0	0	0	0	0	0	0	0	0	0	0	0	0	0	0	3	0	0
73139	Oesophageal varices without bleeding in diseases EC	0	0	0	0	0	0	0	0	0	0	0	0	0	0	0	3	0	0
87861	Oth transjugular intrahepatic operations blood vessel liver	0	0	0	0	0	0	0	0	0	0	0	0	0	0	0	3	0	0

88994	Compensation for liver failure	0	0	0	0	0	0	0	0	0	0	0	0	0	0	0	3	0	0
89356	Percutaneous transluminal insertion of stent portal vein	0	0	0	0	0	0	0	0	0	0	0	0	0	0	0	3	0	0
89445	Auxillary liver transplant	0	0	0	0	0	0	0	0	0	0	0	0	0	0	0	3	0	0
89587	Other specified viral hepatitis with hepatic coma NOS	0	0	0	0	0	0	0	0	0	0	0	0	0	0	0	3	0	0
89717	Fibreopt endoscop rubber band ligation of upper GIT varices	0	0	0	0	0	0	0	0	0	0	0	0	0	0	0	3	0	0
90925	Transjugular intrahepatic operations blood vessel of liver	0	0	0	0	0	0	0	0	0	0	0	0	0	0	0	3	0	0
93737	Transjugular intrahepatic insertion stent into portal vein	0	0	0	0	0	0	0	0	0	0	0	0	0	0	0	3	0	0
95118	Percutan transluminal insertion of stent graft portal vein	0	0	0	0	0	0	0	0	0	0	0	0	0	0	0	3	0	0
96756	Oesophageal varices with bleeding in diseases EC	0	0	0	0	0	0	0	0	0	0	0	0	0	0	0	3	0	0
97650	Compensation for liver failure NOS	0	0	0	0	0	0	0	0	0	0	0	0	0	0	0	3	0	0
100073	Piggy back liver transplant	0	0	0	0	0	0	0	0	0	0	0	0	0	0	0	3	0	0
105611	[X]Oesophageal varices in diseases classified elsewhere	0	0	0	0	0	0	0	0	0	0	0	0	0	0	0	3	0	0
105758	Liver transplant planned	0	0	0	0	0	0	0	0	0	0	0	0	0	0	0	3	0	0
106179	Transjugular intrahepatic angioplasty of portal vein	0	0	0	0	0	0	0	0	0	0	0	0	0	0	0	3	0	0
106575	Discussion about liver transplantation	0	0	0	0	0	0	0	0	0	0	0	0	0	0	0	3	0	0
106828	Transjugular intrahepatic operations blood vessel liver NOS	0	0	0	0	0	0	0	0	0	0	0	0	0	0	0	3	0	0
107093	Transjugular intrahepatic ins stent graft into portal vein	0	0	0	0	0	0	0	0	0	0	0	0	0	0	0	3	0	0
107975	Other specified open operation on oesophageal varices	0	0	0	0	0	0	0	0	0	0	0	0	0	0	0	3	0	0
1952	Secondary malignant neoplasm of kidney	0	0	0	0	0	0	0	0	0	0	0	0	0	0	0	0	6	0
3152	[M]Carcinoma, metastatic, NOS	0	0	0	0	0	0	0	0	0	0	0	0	0	0	0	0	6	0
3197	[M]Neoplasm, metastatic	0	0	0	0	0	0	0	0	0	0	0	0	0	0	0	0	6	0
4137	Secondary malignant neoplasm of lung	0	0	0	0	0	0	0	0	0	0	0	0	0	0	0	0	6	0
4403	Liver metastases	0	0	0	0	0	0	0	0	0	0	0	0	0	0	0	0	6	0
5198	Secondary malignant neoplasm of brain	0	0	0	0	0	0	0	0	0	0	0	0	0	0	0	0	6	0
5199	Cerebral metastasis	0	0	0	0	0	0	0	0	0	0	0	0	0	0	0	0	6	0

5455	[M]Adenocarcinoma, metastatic, NOS	0	0	0	0	0	0	0	0	0	0	0	0	0	0	0	0	6	0
5842	Secondary malignant neoplasm of other specified sites	0	0	0	0	0	0	0	0	0	0	0	0	0	0	0	0	6	0
6170	Carcinomatosis	0	0	0	0	0	0	0	0	0	0	0	0	0	0	0	0	6	0
6471	Metastases of respiratory and/or digestive systems	0	0	0	0	0	0	0	0	0	0	0	0	0	0	0	0	6	0
6701	Secondary and unspec malig neop intrapelvic lymph nodes	0	0	0	0	0	0	0	0	0	0	0	0	0	0	0	0	6	0
6985	[M]Secondary neoplasm	0	0	0	0	0	0	0	0	0	0	0	0	0	0	0	0	6	0
7593	Malignant pleural effusion	0	0	0	0	0	0	0	0	0	0	0	0	0	0	0	0	6	0
7654	Secondary malignant neoplasm of bone and bone marrow	0	0	0	0	0	0	0	0	0	0	0	0	0	0	0	0	6	0
7830	Lymph node metastases	0	0	0	0	0	0	0	0	0	0	0	0	0	0	0	0	6	0
8154	Malignant ascites	0	0	0	0	0	0	0	0	0	0	0	0	0	0	0	0	6	0
8600	Pain from metastases	0	0	0	0	0	0	0	0	0	0	0	0	0	0	0	0	6	0
9505	Secondary malignant neoplasm of skin of breast	0	0	0	0	0	0	0	0	0	0	0	0	0	0	0	0	6	0
9618	Secondary and unspecified malignant neoplasm of lymph nodes	0	0	0	0	0	0	0	0	0	0	0	0	0	0	0	0	6	0
13569	Disseminated malignancy NOS	0	0	0	0	0	0	0	0	0	0	0	0	0	0	0	0	6	0
15103	Secondary malignant neoplasm of liver	0	0	0	0	0	0	0	0	0	0	0	0	0	0	0	0	6	0
15507	Secondary and unspec malig neop lymph nodes NOS	0	0	0	0	0	0	0	0	0	0	0	0	0	0	0	0	6	0
16213	Secondary malignant neoplasm of pleura	0	0	0	0	0	0	0	0	0	0	0	0	0	0	0	0	6	0
16500	Secondary malignant neoplasm of other specified site NOS	0	0	0	0	0	0	0	0	0	0	0	0	0	0	0	0	6	0
16692	[M]Carcinomatosis	0	0	0	0	0	0	0	0	0	0	0	0	0	0	0	0	6	0
16760	Secondary malignant neoplasm of breast	0	0	0	0	0	0	0	0	0	0	0	0	0	0	0	0	6	0
17098	[M]PSEUDOMYXOMA PERITONEI	0	0	0	0	0	0	0	0	0	0	0	0	0	0	0	0	6	0
18616	Secondary malignant neoplasm of other specified sites	0	0	0	0	0	0	0	0	0	0	0	0	0	0	0	0	6	0
18658	Secondary and unspec malig neop common iliac lymph nodes	0	0	0	0	0	0	0	0	0	0	0	0	0	0	0	0	6	0
18676	Pathological fracture due to metastatic bone disease	0	0	0	0	0	0	0	0	0	0	0	0	0	0	0	0	6	0
19945	Secondary malignant neoplasm of skin	0	0	0	0	0	0	0	0	0	0	0	0	0	0	0	0	6	0

20159	Secondary and unspec malig neop lymph nodes multiple sites	0	0	0	0	0	0	0	0	0	0	0	0	0	0	0	0	6	0
21590	Secondary malignant neoplasm of prostate	0	0	0	0	0	0	0	0	0	0	0	0	0	0	0	0	6	0
22146	Secondary malignant neoplasm of bladder	0	0	0	0	0	0	0	0	0	0	0	0	0	0	0	0	6	0
22267	[M]Neoplasm, malig, uncertain whether primary or metastatic	0	0	0	0	0	0	0	0	0	0	0	0	0	0	0	0	6	0
22524	Secondary malignant neoplasm of other specified site NOS	0	0	0	0	0	0	0	0	0	0	0	0	0	0	0	0	6	0
24293	[M]Squamous cell carcinoma, metastatic NOS	0	0	0	0	0	0	0	0	0	0	0	0	0	0	0	0	6	0
24301	SECONDARY CARCINOMA OF RESPIRATORY AND/OR DIGESTIVE SYSTEMS	0	0	0	0	0	0	0	0	0	0	0	0	0	0	0	0	6	0
25366	Secondary and unspec malig neop ant mediastinal lymph nodes	0	0	0	0	0	0	0	0	0	0	0	0	0	0	0	0	6	0
27391	Secondary malignant neoplasm of peritoneum	0	0	0	0	0	0	0	0	0	0	0	0	0	0	0	0	6	0
27651	Secondary carcinoma of other specified sites	0	0	0	0	0	0	0	0	0	0	0	0	0	0	0	0	6	0
28059	Secondary and unspec malig neop of facial lymph nodes	0	0	0	0	0	0	0	0	0	0	0	0	0	0	0	0	6	0
28727	Secondary malignant neoplasm of colon	0	0	0	0	0	0	0	0	0	0	0	0	0	0	0	0	6	0
29285	Radiotherapy-tumour palliation	0	0	0	0	0	0	0	0	0	0	0	0	0	0	0	0	6	0
33395	Secondary and unspec malig neop superficial cervical LN	0	0	0	0	0	0	0	0	0	0	0	0	0	0	0	0	6	0
33843	Secondary malignant neoplasm of brain and spinal cord	0	0	0	0	0	0	0	0	0	0	0	0	0	0	0	0	6	0
34145	Secondary malignant neoplasm of testis	0	0	0	0	0	0	0	0	0	0	0	0	0	0	0	0	6	0
34891	[M]Sarcomatosis NOS	0	0	0	0	0	0	0	0	0	0	0	0	0	0	0	0	6	0
35053	Secondary malig neop of respiratory and digestive systems	0	0	0	0	0	0	0	0	0	0	0	0	0	0	0	0	6	0
35186	[X]Malignant neoplasm of ill-defined, secondary and unspeci	0	0	0	0	0	0	0	0	0	0	0	0	0	0	0	0	6	0
35364	Secondary malignant neoplasm of retroperitoneum	0	0	0	0	0	0	0	0	0	0	0	0	0	0	0	0	6	0
35999	Secondary malignant neoplasm of skin of neck	0	0	0	0	0	0	0	0	0	0	0	0	0	0	0	0	6	0
36147	Secondary malignant neoplasm of liver	0	0	0	0	0	0	0	0	0	0	0	0	0	0	0	0	6	0
36200	Secondary malig neop of large intestine or rectum NOS	0	0	0	0	0	0	0	0	0	0	0	0	0	0	0	0	6	0
36401	Secondary malignant neoplasm of adrenal gland	0	0	0	0	0	0	0	0	0	0	0	0	0	0	0	0	6	0

37540	Secondary and unspec malig neop axillary lymph nodes	0	0	0	0	0	0	0	0	0	0	0	0	0	0	0	0	6	0
37919	Secondary and unspec malig neop internal mammary lymph nodes	0	0	0	0	0	0	0	0	0	0	0	0	0	0	0	0	6	0
38343	Secondary and unspec malig neop submental lymph nodes	0	0	0	0	0	0	0	0	0	0	0	0	0	0	0	0	6	0
38918	Secondary malignant neoplasm of spinal cord	0	0	0	0	0	0	0	0	0	0	0	0	0	0	0	0	6	0
39433	Secondary and unspec malig neop submandibular lymph nodes	0	0	0	0	0	0	0	0	0	0	0	0	0	0	0	0	6	0
41144	Secondary malignant neoplasm of skin of trunk	0	0	0	0	0	0	0	0	0	0	0	0	0	0	0	0	6	0
41691	Secondary and unspec malig neop coeliac lymph nodes	0	0	0	0	0	0	0	0	0	0	0	0	0	0	0	0	6	0
43930	Secondary malignant neoplasm of skin of head	0	0	0	0	0	0	0	0	0	0	0	0	0	0	0	0	6	0
44529	Secondary malignant neoplasm of large intestine and rectum	0	0	0	0	0	0	0	0	0	0	0	0	0	0	0	0	6	0
44615	Secondary malignant neoplasm of ovary	0	0	0	0	0	0	0	0	0	0	0	0	0	0	0	0	6	0
44627	Secondary and unspec malig neop anterior cervical LN	0	0	0	0	0	0	0	0	0	0	0	0	0	0	0	0	6	0
44931	Secondary and unspec malig neop intra-abdominal LN NOS	0	0	0	0	0	0	0	0	0	0	0	0	0	0	0	0	6	0
45824	Secondary malignant neoplasm of tongue	0	0	0	0	0	0	0	0	0	0	0	0	0	0	0	0	6	0
46409	Secondary and unspec malig neop pectoral lymph nodes	0	0	0	0	0	0	0	0	0	0	0	0	0	0	0	0	6	0
47366	Secondary and unspec malig neop sacral lymph nodes	0	0	0	0	0	0	0	0	0	0	0	0	0	0	0	0	6	0
48828	Secondary malignant neoplasm of skin of hip and leg	0	0	0	0	0	0	0	0	0	0	0	0	0	0	0	0	6	0
49145	Secondary malignant neoplasm of penis	0	0	0	0	0	0	0	0	0	0	0	0	0	0	0	0	6	0
49214	Secondary and unspec malig neop lymph nodes head/face/neck	0	0	0	0	0	0	0	0	0	0	0	0	0	0	0	0	6	0
50199	Secondary and unspec malig neop axilla and upper limb LN	0	0	0	0	0	0	0	0	0	0	0	0	0	0	0	0	6	0
50904	Secondary and unspec malig neop infraclavicular lymph nodes	0	0	0	0	0	0	0	0	0	0	0	0	0	0	0	0	6	0
51551	Secondary malignant neoplasm of mediastinum	0	0	0	0	0	0	0	0	0	0	0	0	0	0	0	0	6	0
52190	Secondary and unspec malig neop pulmonary lymph nodes	0	0	0	0	0	0	0	0	0	0	0	0	0	0	0	0	6	0
52736	Secondary and unspec malig neop intra-abdominal lymph nodes	0	0	0	0	0	0	0	0	0	0	0	0	0	0	0	0	6	0
53528	Secondary malignant neoplasm of urethra	0	0	0	0	0	0	0	0	0	0	0	0	0	0	0	0	6	0

53694	[M]Krukenberg tumour	0	0	0	0	0	0	0	0	0	0	0	0	0	0	0	0	6	0
54120	Secondary malignant neoplasm of other part of nervous system	0	0	0	0	0	0	0	0	0	0	0	0	0	0	0	0	6	0
54253	[X]Secondary malignant neoplasm of other specified sites	0	0	0	0	0	0	0	0	0	0	0	0	0	0	0	0	6	0
54278	Secondary and unspec malig neop superficial inguinal LN	0	0	0	0	0	0	0	0	0	0	0	0	0	0	0	0	6	0
54679	Secondary malignant neoplasm of unknown site	0	0	0	0	0	0	0	0	0	0	0	0	0	0	0	0	6	0
54874	[M]Metastatic signet ring cell carcinoma	0	0	0	0	0	0	0	0	0	0	0	0	0	0	0	0	6	0
55090	Secondary malignant neoplasm of uterus	0	0	0	0	0	0	0	0	0	0	0	0	0	0	0	0	6	0
55096	Secondary malignant neoplasm of skin NOS	0	0	0	0	0	0	0	0	0	0	0	0	0	0	0	0	6	0
55463	Secondary and unspec malig neop post mediastinal lymph nodes	0	0	0	0	0	0	0	0	0	0	0	0	0	0	0	0	6	0
55946	Secondary malignant neoplasm of duodenum	0	0	0	0	0	0	0	0	0	0	0	0	0	0	0	0	6	0
56345	Secondary malignant neoplasm of other digestive organ	0	0	0	0	0	0	0	0	0	0	0	0	0	0	0	0	6	0
57481	[X]Secondary malignant neoplasm/oth+unspc respiratory organs	0	0	0	0	0	0	0	0	0	0	0	0	0	0	0	0	6	0
58692	Secondary and unspec malig neop paratracheal lymph nodes	0	0	0	0	0	0	0	0	0	0	0	0	0	0	0	0	6	0
59375	Secondary malignant neoplasm of brain or spinal cord NOS	0	0	0	0	0	0	0	0	0	0	0	0	0	0	0	0	6	0
60134	Secondary malignant neoplasm of ureter	0	0	0	0	0	0	0	0	0	0	0	0	0	0	0	0	6	0
60335	Secondary malignant neoplasm of vulva	0	0	0	0	0	0	0	0	0	0	0	0	0	0	0	0	6	0
61289	Secondary and unspec malig neop deep inguinal lymph nodes	0	0	0	0	0	0	0	0	0	0	0	0	0	0	0	0	6	0
61677	Secondary and unspec malig neop inferior mesenteric LN	0	0	0	0	0	0	0	0	0	0	0	0	0	0	0	0	6	0
62124	Secondary and unspec malig neop bronchopulmonary lymph nodes	0	0	0	0	0	0	0	0	0	0	0	0	0	0	0	0	6	0
62584	Secondary malignant neoplasm of other respiratory organs	0	0	0	0	0	0	0	0	0	0	0	0	0	0	0	0	6	0
62828	Secondary malignant neoplasm of other urinary organ NOS	0	0	0	0	0	0	0	0	0	0	0	0	0	0	0	0	6	0
62909	Secondary malignant neoplasm of rectum	0	0	0	0	0	0	0	0	0	0	0	0	0	0	0	0	6	0
62951	Bone tumour/metast.irradiat.	0	0	0	0	0	0	0	0	0	0	0	0	0	0	0	0	6	0
63896	Secondary malignant neoplasm of skin of shoulder and arm	0	0	0	0	0	0	0	0	0	0	0	0	0	0	0	0	6	0

63915	Secondary and unspec malig neop inguinal and lower limb LN	0	0	0	0	0	0	0	0	0	0	0	0	0	0	0	0	6	0
64116	Secondary and unspec malig neop intrathoracic lymph nodes	0	0	0	0	0	0	0	0	0	0	0	0	0	0	0	0	6	0
64680	Secondary malignant neoplasm of small intestine and duodenum	0	0	0	0	0	0	0	0	0	0	0	0	0	0	0	0	6	0
64918	Secondary and unspec malig neop of superficial parotid LN	0	0	0	0	0	0	0	0	0	0	0	0	0	0	0	0	6	0
65253	Secondary and unspec malignant neoplasm occipital lymph node	0	0	0	0	0	0	0	0	0	0	0	0	0	0	0	0	6	0
65490	Secondary cancer of the vulva	0	0	0	0	0	0	0	0	0	0	0	0	0	0	0	0	6	0
66083	Secondary malig neop of respiratory or digestive system NOS	0	0	0	0	0	0	0	0	0	0	0	0	0	0	0	0	6	0
66163	[X]2ndry+unspcf malignant neoplasm lymph nodes/multi regions	0	0	0	0	0	0	0	0	0	0	0	0	0	0	0	0	6	0
66775	Secondary and unspec malignant neoplasm mastoid lymph nodes	0	0	0	0	0	0	0	0	0	0	0	0	0	0	0	0	6	0
67129	Secondary unspec malig neop lymph nodes head/face/neck NOS	0	0	0	0	0	0	0	0	0	0	0	0	0	0	0	0	6	0
67248	Thyroid tumour/metast irradiat	0	0	0	0	0	0	0	0	0	0	0	0	0	0	0	0	6	0
67396	Secondary malig neop of retroperitoneum and peritoneum	0	0	0	0	0	0	0	0	0	0	0	0	0	0	0	0	6	0
67797	Secondary and unspec malig neop superfic tracheobronchial LN	0	0	0	0	0	0	0	0	0	0	0	0	0	0	0	0	6	0
68332	[X]2ndry malignant neoplasm/oth+unspec parts/nervous system	0	0	0	0	0	0	0	0	0	0	0	0	0	0	0	0	6	0
68611	Secondary and unspec malig neop deep cervical LN	0	0	0	0	0	0	0	0	0	0	0	0	0	0	0	0	6	0
69132	Secondary and unspec malig neop external iliac lymph nodes	0	0	0	0	0	0	0	0	0	0	0	0	0	0	0	0	6	0
69392	Secondary and unspec malig neop inferior tracheobronchial LN	0	0	0	0	0	0	0	0	0	0	0	0	0	0	0	0	6	0
70026	Secondary malig neop of small intestine or duodenum NOS	0	0	0	0	0	0	0	0	0	0	0	0	0	0	0	0	6	0
70736	Secondary malignant neoplasm of vagina	0	0	0	0	0	0	0	0	0	0	0	0	0	0	0	0	6	0
70747	Secondary and unspec malig neop of inguinal and leg LN NOS	0	0	0	0	0	0	0	0	0	0	0	0	0	0	0	0	6	0
72713	Secondary and unspec malig neop superficial mesenteric LN	0	0	0	0	0	0	0	0	0	0	0	0	0	0	0	0	6	0
72803	Secondary and unspec malig neop intrapelvic LN NOS	0	0	0	0	0	0	0	0	0	0	0	0	0	0	0	0	6	0
73213	Secondary malignant neoplasm of other urinary organs	0	0	0	0	0	0	0	0	0	0	0	0	0	0	0	0	6	0
73538	Secondary and unspec malig neop axilla and upper limb LN NOS	0	0	0	0	0	0	0	0	0	0	0	0	0	0	0	0	6	0

73616	Secondary malignant neoplasm of cervix uteri	0	0	0	0	0	0	0	0	0	0	0	0	0	0	0	0	6	0
84368	Secondary and unspec malig neop internal iliac lymph nodes	0	0	0	0	0	0	0	0	0	0	0	0	0	0	0	0	6	0
88022	[X]Secondary malignant neoplasm/oth+unspcfd digestive organs	0	0	0	0	0	0	0	0	0	0	0	0	0	0	0	0	6	0
92703	Secondary and unspec malig neop deep parotid lymph nodes	0	0	0	0	0	0	0	0	0	0	0	0	0	0	0	0	6	0
93716	Secondary and unspec malig neop intrathoracic LN NOS	0	0	0	0	0	0	0	0	0	0	0	0	0	0	0	0	6	0
95378	Secondary and unspec malig neop diaphragmatic lymph nodes	0	0	0	0	0	0	0	0	0	0	0	0	0	0	0	0	6	0
97091	[X]2ndry malignant neoplasm/bladder+oth+unsp urinary organs	0	0	0	0	0	0	0	0	0	0	0	0	0	0	0	0	6	0
97672	Secondary malig neop of retroperitoneum or peritoneum NOS	0	0	0	0	0	0	0	0	0	0	0	0	0	0	0	0	6	0
97832	Secondary cancer of the cervix	0	0	0	0	0	0	0	0	0	0	0	0	0	0	0	0	6	0
98626	Secondary and unspec malig neop supratrochlear lymph nodes	0	0	0	0	0	0	0	0	0	0	0	0	0	0	0	0	6	0
99511	Secondary malignant neoplasm of ileum	0	0	0	0	0	0	0	0	0	0	0	0	0	0	0	0	6	0
100296	Secondary malignant neoplasm of skin of face	0	0	0	0	0	0	0	0	0	0	0	0	0	0	0	0	6	0
101662	Secondary and unspec malig neop circumflex iliac LN	0	0	0	0	0	0	0	0	0	0	0	0	0	0	0	0	6	0
104480	Secondary malignant neoplasm of epididymis and vas deferens	0	0	0	0	0	0	0	0	0	0	0	0	0	0	0	0	6	0
104699	[M]No microscopic confirmation tumour, clinically metastatic	0	0	0	0	0	0	0	0	0	0	0	0	0	0	0	0	6	0
105953	Secondary and unspec malig neop intercostal lymph nodes	0	0	0	0	0	0	0	0	0	0	0	0	0	0	0	0	6	0
106134	[M]Meningeal sarcomatosis	0	0	0	0	0	0	0	0	0	0	0	0	0	0	0	0	6	0
2835	HIV positive	0	0	0	0	0	0	0	0	0	0	0	0	0	0	0	0	0	6
8281	HIV disease resulting in wasting syndrome	0	0	0	0	0	0	0	0	0	0	0	0	0	0	0	0	0	6
9130	Human immunodeficiency virus infection	0	0	0	0	0	0	0	0	0	0	0	0	0	0	0	0	0	6
23763	AIDS carrier	0	0	0	0	0	0	0	0	0	0	0	0	0	0	0	0	0	6
23770	Acquired immune deficiency syndrome	0	0	0	0	0	0	0	0	0	0	0	0	0	0	0	0	0	6
23951	HIV disease resulting in candidiasis	0	0	0	0	0	0	0	0	0	0	0	0	0	0	0	0	0	6
24872	[V]Asymptomatic human immunodeficency virus infection status	0	0	0	0	0	0	0	0	0	0	0	0	0	0	0	0	0	6

27641	HIV disease resulting in Pneumocystis carinii pneumonia	0	0	0	0	0	0	0	0	0	0	0	0	0	0	0	0	0	6
27853	HIV disease resulting in Kaposi's sarcoma	0	0	0	0	0	0	0	0	0	0	0	0	0	0	0	0	0	6
33943	Notification of AIDS	0	0	0	0	0	0	0	0	0	0	0	0	0	0	0	0	0	6
36294	Acquired human immunodeficiency virus infection syndrome NOS	0	0	0	0	0	0	0	0	0	0	0	0	0	0	0	0	0	6
37006	HIV disease resulting in mycobacterial infection	0	0	0	0	0	0	0	0	0	0	0	0	0	0	0	0	0	6
41185	[X]Dementia in human immunodef virus [HIV] disease	0	0	0	0	0	0	0	0	0	0	0	0	0	0	0	0	0	6
44288	[D]Laboratory evidence of human immunodeficiency virus [HIV]	0	0	0	0	0	0	0	0	0	0	0	0	0	0	0	0	0	6
44303	Human immunodef virus resulting in other disease	0	0	0	0	0	0	0	0	0	0	0	0	0	0	0	0	0	6
44617	HIV disease resulting in Burkitt's lymphoma	0	0	0	0	0	0	0	0	0	0	0	0	0	0	0	0	0	6
47632	HIV disease result/haematological+immunologic abnorms,NEC	0	0	0	0	0	0	0	0	0	0	0	0	0	0	0	0	0	6
50076	HIV disease resulting in multiple infections	0	0	0	0	0	0	0	0	0	0	0	0	0	0	0	0	0	6
51708	HIV dis reslt/oth mal neopl/lymph,h'matopoetc+reltd tissu	0	0	0	0	0	0	0	0	0	0	0	0	0	0	0	0	0	6
53636	Human immunodeficiency virus with neurological disease	0	0	0	0	0	0	0	0	0	0	0	0	0	0	0	0	0	6
58857	Acute human immunodeficiency virus infection	0	0	0	0	0	0	0	0	0	0	0	0	0	0	0	0	0	6
58859	Asymptomatic human immunodeficiency virus infection	0	0	0	0	0	0	0	0	0	0	0	0	0	0	0	0	0	6
62854	[X]Human immunodeficiency virus disease	0	0	0	0	0	0	0	0	0	0	0	0	0	0	0	0	0	6
62891	Human immunodeficiency virus with other clinical findings	0	0	0	0	0	0	0	0	0	0	0	0	0	0	0	0	0	6
65117	HIV disease resulting in lymphoid interstitial pneumonitis	0	0	0	0	0	0	0	0	0	0	0	0	0	0	0	0	0	6
66367	HIV dis resulting oth types of non-Hodgkin's lymphoma	0	0	0	0	0	0	0	0	0	0	0	0	0	0	0	0	0	6
66368	HIV disease resulting in cytomegaloviral disease	0	0	0	0	0	0	0	0	0	0	0	0	0	0	0	0	0	6
67575	HIV disease resulting in unspecified malignant neoplasm	0	0	0	0	0	0	0	0	0	0	0	0	0	0	0	0	0	6
69766	HIV infection with persistent generalised lymphadenopathy	0	0	0	0	0	0	0	0	0	0	0	0	0	0	0	0	0	6
69767	[X]HIV disease resulting in other non-Hodgkin's lymphoma	0	0	0	0	0	0	0	0	0	0	0	0	0	0	0	0	0	6

70528	Human immunodeficiency virus with secondary infection	0	0	0	0	0	0	0	0	0	0	0	0	0	0	0	0	0	6
70869	Human immunodeficiency virus with constitutional disease	0	0	0	0	0	0	0	0	0	0	0	0	0	0	0	0	0	6
71450	HIV disease resulting/unspcf infectious+parasitic disease	0	0	0	0	0	0	0	0	0	0	0	0	0	0	0	0	0	6
96751	[X]HIV disease result/haematological+immunologic abnorms,NEC	0	0	0	0	0	0	0	0	0	0	0	0	0	0	0	0	0	6
100769	[X]Unspecified human immunodeficiency virus [HIV] disease	0	0	0	0	0	0	0	0	0	0	0	0	0	0	0	0	0	6
101836	Human immunodeficiency virus with secondary cancers	0	0	0	0	0	0	0	0	0	0	0	0	0	0	0	0	0	6
102117	[X]HIV disease resulting in multiple infections	0	0	0	0	0	0	0	0	0	0	0	0	0	0	0	0	0	6
102252	[X]HIV disease resulting in other specified conditions	0	0	0	0	0	0	0	0	0	0	0	0	0	0	0	0	0	6
104134	[X]HIV disease resulting/other infectious+parasitic diseases	0	0	0	0	0	0	0	0	0	0	0	0	0	0	0	0	0	6
104466	HIV disease complicating pregnancy childbirth puerperium	0	0	0	0	0	0	0	0	0	0	0	0	0	0	0	0	0	6
104717	HIV disease resulting in Pneumocystis jirovecii pneumonia	0	0	0	0	0	0	0	0	0	0	0	0	0	0	0	0	0	6
105040	HIV pos gen health check serv declind - enhanc service admin	0	0	0	0	0	0	0	0	0	0	0	0	0	0	0	0	0	6
105324	HIV disease resulting in multiple malignant neoplasms	0	0	0	0	0	0	0	0	0	0	0	0	0	0	0	0	0	6
107807	[X]HIV disease resulting in other viral infections	0	0	0	0	0	0	0	0	0	0	0	0	0	0	0	0	0	6
108054	HIV disease resulting in Kaposi sarcoma	0	0	0	0	0	0	0	0	0	0	0	0	0	0	0	0	0	6
27519	Pneumonia with pneumocystis carinii	0	0	0	0	0	0	0	0	0	0	0	0	0	0	0	0	0	6
101191	Human immunodeficiency virus annual review	0	0	0	0	0	0	0	0	0	0	0	0	0	0	0	0	0	6
540	HTLV-3 antibody positive	0	0	0	0	0	0	0	0	0	0	0	0	0	0	0	0	0	6
43537	HIV 1 nucleic acid detection	0	0	0	0	0	0	0	0	0	0	0	0	0	0	0	0	0	6

icd	icd_description	cci_1mi	cci_1ccf	cci_1pad	cci_1stroke	cci_1dement	cci_1resp	cci_1ctd	cci_1peptic	cci_1liver	cci_2hemi	cci_2renal	cci_2diabcomp	cci_2cancer	cci_2leuk	cci_2lymph	cci_3liver	cci_6mets	cci_6hiv
I21	Acute myocardial infarction	1	0	0	0	0	0	0	0	0	0	0	0	0	0	0	0	0	0

I21.0	Acute transmural myocardial infarction of anterior wall	1	0	0	0	0	0	0	0	0	0	0	0	0	0	0	0	0	0
I21.1	Acute transmural myocardial infarction of inferior wall	1	0	0	0	0	0	0	0	0	0	0	0	0	0	0	0	0	0
I21.2	Acute transmural myocardial infarction of other sites	1	0	0	0	0	0	0	0	0	0	0	0	0	0	0	0	0	0
I21.3	Acute transmural myocardial infarction of unspecified site	1	0	0	0	0	0	0	0	0	0	0	0	0	0	0	0	0	0
I21.4	Acute subendocardial myocardial infarction	1	0	0	0	0	0	0	0	0	0	0	0	0	0	0	0	0	0
I21.9	Acute myocardial infarction, unspecified	1	0	0	0	0	0	0	0	0	0	0	0	0	0	0	0	0	0
I22	Subsequent myocardial infarction	1	0	0	0	0	0	0	0	0	0	0	0	0	0	0	0	0	0
I22.0	Subsequent myocardial infarction of anterior wall	1	0	0	0	0	0	0	0	0	0	0	0	0	0	0	0	0	0
I22.1	Subsequent myocardial infarction of inferior wall	1	0	0	0	0	0	0	0	0	0	0	0	0	0	0	0	0	0
I22.8	Subsequent myocardial infarction of other sites	1	0	0	0	0	0	0	0	0	0	0	0	0	0	0	0	0	0
I22.9	Subsequent myocardial infarction of unspecified site	1	0	0	0	0	0	0	0	0	0	0	0	0	0	0	0	0	0
I23	Certain current complication follow acute myocardial infarct	1	0	0	0	0	0	0	0	0	0	0	0	0	0	0	0	0	0
I23.0	Haemopericardium as curr comp folow acut myocard infarct	1	0	0	0	0	0	0	0	0	0	0	0	0	0	0	0	0	0
I23.1	Atral sept defect as curr comp folow acut myocardal infarct	1	0	0	0	0	0	0	0	0	0	0	0	0	0	0	0	0	0
I23.2	Ventric sep defect as curr comp fol acut myocardal infarc	1	0	0	0	0	0	0	0	0	0	0	0	0	0	0	0	0	0
I23.3	Rup cardac wal withou haemopercard as cur comp fol ac MI	1	0	0	0	0	0	0	0	0	0	0	0	0	0	0	0	0	0
I23.4	Rup chordae tendinae as curr comp fol acut myocard infarct	1	0	0	0	0	0	0	0	0	0	0	0	0	0	0	0	0	0
I23.5	Rup papilary muscle as curr comp fol acute myocard infarct	1	0	0	0	0	0	0	0	0	0	0	0	0	0	0	0	0	0
I23.6	Thromb atrium/auric append/vent as curr comp foll acute MI	1	0	0	0	0	0	0	0	0	0	0	0	0	0	0	0	0	0
I23.8	Oth current comp following acute myocardial infarction	1	0	0	0	0	0	0	0	0	0	0	0	0	0	0	0	0	0
I11.0	Hypertensive heart disease with (congestive) heart failure	0	1	0	0	0	0	0	0	0	0	0	0	0	0	0	0	0	0
I13.0	Hypertens heart and renal dis with (conges) heart failure	0	1	0	0	0	0	0	0	0	0	0	0	0	0	0	0	0	0
I13.2	Hyper heart and renal dis both (cong) heart and renal fail	0	1	0	0	0	0	0	0	0	0	0	0	0	0	0	0	0	0
I50	Heart failure	0	1	0	0	0	0	0	0	0	0	0	0	0	0	0	0	0	0

I50.0	Congestive heart failure	0	1	0	0	0	0	0	0	0	0	0	0	0	0	0	0	0	0
I50.1	Left ventricular failure	0	1	0	0	0	0	0	0	0	0	0	0	0	0	0	0	0	0
I50.9	Heart failure, unspecified	0	1	0	0	0	0	0	0	0	0	0	0	0	0	0	0	0	0
I70	Atherosclerosis	0	0	1	0	0	0	0	0	0	0	0	0	0	0	0	0	0	0
I70.0	Atherosclerosis of aorta	0	0	1	0	0	0	0	0	0	0	0	0	0	0	0	0	0	0
I70.1	Atherosclerosis of renal artery	0	0	1	0	0	0	0	0	0	0	0	0	0	0	0	0	0	0
I70.2	Atherosclerosis of arteries of extremities	0	0	1	0	0	0	0	0	0	0	0	0	0	0	0	0	0	0
I70.8	Atherosclerosis of other arteries	0	0	1	0	0	0	0	0	0	0	0	0	0	0	0	0	0	0
I70.9	Generalized and unspecified atherosclerosis	0	0	1	0	0	0	0	0	0	0	0	0	0	0	0	0	0	0
I71	Aortic aneurysm and dissection	0	0	1	0	0	0	0	0	0	0	0	0	0	0	0	0	0	0
I71.0	Dissection of aorta [any part]	0	0	1	0	0	0	0	0	0	0	0	0	0	0	0	0	0	0
I71.1	Thoracic aortic aneurysm, ruptured	0	0	1	0	0	0	0	0	0	0	0	0	0	0	0	0	0	0
I71.2	Thoracic aortic aneurysm, without mention of rupture	0	0	1	0	0	0	0	0	0	0	0	0	0	0	0	0	0	0
I71.3	Abdominal aortic aneurysm, ruptured	0	0	1	0	0	0	0	0	0	0	0	0	0	0	0	0	0	0
I71.4	Abdominal aortic aneurysm, without mention of rupture	0	0	1	0	0	0	0	0	0	0	0	0	0	0	0	0	0	0
I71.5	Thoracoabdominal aortic aneurysm, ruptured	0	0	1	0	0	0	0	0	0	0	0	0	0	0	0	0	0	0
I71.6	Thoracoabdominal aortic aneurysm, without mention of rupture	0	0	1	0	0	0	0	0	0	0	0	0	0	0	0	0	0	0
I71.8	Aortic aneurysm of unspecified site, ruptured	0	0	1	0	0	0	0	0	0	0	0	0	0	0	0	0	0	0
I71.9	Aortic aneurysm of unspec site, without mention of rupture	0	0	1	0	0	0	0	0	0	0	0	0	0	0	0	0	0	0
I73.1	Thromboangiitis obliterans [Buerger]	0	0	1	0	0	0	0	0	0	0	0	0	0	0	0	0	0	0
I73.8	Other specified peripheral vascular diseases	0	0	1	0	0	0	0	0	0	0	0	0	0	0	0	0	0	0
I73.9	Peripheral vascular disease, unspecified	0	0	1	0	0	0	0	0	0	0	0	0	0	0	0	0	0	0
I74.2	Embolism and thrombosis of arteries of upper extremities	0	0	1	0	0	0	0	0	0	0	0	0	0	0	0	0	0	0
I74.3	Embolism and thrombosis of arteries of lower extremities	0	0	1	0	0	0	0	0	0	0	0	0	0	0	0	0	0	0

I74.4	Embolism and thrombosis of arteries of extremities, unspec	0	0	1	0	0	0	0	0	0	0	0	0	0	0	0	0	0	0
I74.5	Embolism and thrombosis of iliac artery	0	0	1	0	0	0	0	0	0	0	0	0	0	0	0	0	0	0
I74.8	Embolism and thrombosis of other arteries	0	0	1	0	0	0	0	0	0	0	0	0	0	0	0	0	0	0
I74.9	Embolism and thrombosis of unspecified artery	0	0	1	0	0	0	0	0	0	0	0	0	0	0	0	0	0	0
I79.2	Peripheral angiopathy in diseases classified elsewhere	0	0	1	0	0	0	0	0	0	0	0	0	0	0	0	0	0	0
I79.8	Oth disord arteries, arterioles & capillaries in diseases CE	0	0	1	0	0	0	0	0	0	0	0	0	0	0	0	0	0	0
G45	Transient cerebral ischaemic attacks and related syndromes	0	0	0	1	0	0	0	0	0	0	0	0	0	0	0	0	0	0
G45.0	Vertebro-basilar artery syndrome	0	0	0	1	0	0	0	0	0	0	0	0	0	0	0	0	0	0
G45.1	Carotid artery syndrome (hemispheric)	0	0	0	1	0	0	0	0	0	0	0	0	0	0	0	0	0	0
G45.2	Multiple and bilateral precerebral artery syndromes	0	0	0	1	0	0	0	0	0	0	0	0	0	0	0	0	0	0
G45.3	Amaurosis fugax	0	0	0	1	0	0	0	0	0	0	0	0	0	0	0	0	0	0
G45.4	Transient global amnesia	0	0	0	1	0	0	0	0	0	0	0	0	0	0	0	0	0	0
G45.8	Other transient cerebral ischaemic attacks and related synd	0	0	0	1	0	0	0	0	0	0	0	0	0	0	0	0	0	0
G45.9	Transient cerebral ischaemic attack, unspecified	0	0	0	1	0	0	0	0	0	0	0	0	0	0	0	0	0	0
G46	Vascular syndromes of brain in cerebrovascular diseases	0	0	0	1	0	0	0	0	0	0	0	0	0	0	0	0	0	0
G46.0	Middle cerebral artery syndrome	0	0	0	1	0	0	0	0	0	0	0	0	0	0	0	0	0	0
G46.1	Anterior cerebral artery syndrome	0	0	0	1	0	0	0	0	0	0	0	0	0	0	0	0	0	0
G46.2	Posterior cerebral artery syndrome	0	0	0	1	0	0	0	0	0	0	0	0	0	0	0	0	0	0
G46.3	Brain stem stroke syndrome	0	0	0	1	0	0	0	0	0	0	0	0	0	0	0	0	0	0
G46.4	Cerebellar stroke syndrome	0	0	0	1	0	0	0	0	0	0	0	0	0	0	0	0	0	0
G46.5	Pure motor lacunar syndrome	0	0	0	1	0	0	0	0	0	0	0	0	0	0	0	0	0	0
G46.6	Pure sensory lacunar syndrome	0	0	0	1	0	0	0	0	0	0	0	0	0	0	0	0	0	0
G46.7	Other lacunar syndromes	0	0	0	1	0	0	0	0	0	0	0	0	0	0	0	0	0	0
G46.8	Oth vascular syndromes of brain in cerebrovascular dis	0	0	0	1	0	0	0	0	0	0	0	0	0	0	0	0	0	0

I60	Subarachnoid haemorrhage	0	0	0	1	0	0	0	0	0	0	0	0	0	0	0	0	0	0
I60.0	Subarachnoid haemorrhage from carotid siphon and bifurcation	0	0	0	1	0	0	0	0	0	0	0	0	0	0	0	0	0	0
I60.1	Subarachnoid haemorrhage from middle cerebral artery	0	0	0	1	0	0	0	0	0	0	0	0	0	0	0	0	0	0
I60.2	Subarachnoid haemorrhage from anterior communicating artery	0	0	0	1	0	0	0	0	0	0	0	0	0	0	0	0	0	0
I60.3	Subarachnoid haemorrhage from posterior communicating artery	0	0	0	1	0	0	0	0	0	0	0	0	0	0	0	0	0	0
I60.4	Subarachnoid haemorrhage from basilar artery	0	0	0	1	0	0	0	0	0	0	0	0	0	0	0	0	0	0
I60.5	Subarachnoid haemorrhage from vertebral artery	0	0	0	1	0	0	0	0	0	0	0	0	0	0	0	0	0	0
I60.6	Subarachnoid haemorrhage from other intracranial arteries	0	0	0	1	0	0	0	0	0	0	0	0	0	0	0	0	0	0
I60.7	Subarachnoid haemorrhage from intracranial artery, unspec	0	0	0	1	0	0	0	0	0	0	0	0	0	0	0	0	0	0
I60.8	Other subarachnoid haemorrhage	0	0	0	1	0	0	0	0	0	0	0	0	0	0	0	0	0	0
I60.9	Subarachnoid haemorrhage, unspecified	0	0	0	1	0	0	0	0	0	0	0	0	0	0	0	0	0	0
I61	Intracerebral haemorrhage	0	0	0	1	0	0	0	0	0	0	0	0	0	0	0	0	0	0
I61.0	Intracerebral haemorrhage in hemisphere, subcortical	0	0	0	1	0	0	0	0	0	0	0	0	0	0	0	0	0	0
I61.1	Intracerebral haemorrhage in hemisphere, cortical	0	0	0	1	0	0	0	0	0	0	0	0	0	0	0	0	0	0
I61.2	Intracerebral haemorrhage in hemisphere, unspecified	0	0	0	1	0	0	0	0	0	0	0	0	0	0	0	0	0	0
I61.3	Intracerebral haemorrhage in brain stem	0	0	0	1	0	0	0	0	0	0	0	0	0	0	0	0	0	0
I61.4	Intracerebral haemorrhage in cerebellum	0	0	0	1	0	0	0	0	0	0	0	0	0	0	0	0	0	0
I61.5	Intracerebral haemorrhage, intraventricular	0	0	0	1	0	0	0	0	0	0	0	0	0	0	0	0	0	0
I61.6	Intracerebral haemorrhage, multiple localized	0	0	0	1	0	0	0	0	0	0	0	0	0	0	0	0	0	0
I61.8	Other intracerebral haemorrhage	0	0	0	1	0	0	0	0	0	0	0	0	0	0	0	0	0	0
I61.9	Intracerebral haemorrhage, unspecified	0	0	0	1	0	0	0	0	0	0	0	0	0	0	0	0	0	0
I62	Other nontraumatic intracranial haemorrhage	0	0	0	1	0	0	0	0	0	0	0	0	0	0	0	0	0	0
I62.0	Subdural haemorrhage (acute)(nontraumatic)	0	0	0	1	0	0	0	0	0	0	0	0	0	0	0	0	0	0
I62.1	Nontraumatic extradural haemorrhage	0	0	0	1	0	0	0	0	0	0	0	0	0	0	0	0	0	0

I62.9	Intracranial haemorrhage (nontraumatic), unspecified	0	0	0	1	0	0	0	0	0	0	0	0	0	0	0	0	0	0
I63	Cerebral infarction	0	0	0	1	0	0	0	0	0	0	0	0	0	0	0	0	0	0
I63.0	Cerebral infarct due to thrombosis of precerebral arteries	0	0	0	1	0	0	0	0	0	0	0	0	0	0	0	0	0	0
I63.1	Cerebral infarction due to embolism of precerebral arteries	0	0	0	1	0	0	0	0	0	0	0	0	0	0	0	0	0	0
I63.2	Cereb infarct due unsp occlusion or stenos precerebrl arts	0	0	0	1	0	0	0	0	0	0	0	0	0	0	0	0	0	0
I63.3	Cerebral infarction due to thrombosis of cerebral arteries	0	0	0	1	0	0	0	0	0	0	0	0	0	0	0	0	0	0
I63.4	Cerebral infarction due to embolism of cerebral arteries	0	0	0	1	0	0	0	0	0	0	0	0	0	0	0	0	0	0
I63.5	Cerebrl infarct due unspec occlusion or stenos cerebrl arts	0	0	0	1	0	0	0	0	0	0	0	0	0	0	0	0	0	0
I63.6	Cereb infarct due cerebral venous thrombosis, nonpyogenic	0	0	0	1	0	0	0	0	0	0	0	0	0	0	0	0	0	0
I63.8	Other cerebral infarction	0	0	0	1	0	0	0	0	0	0	0	0	0	0	0	0	0	0
I63.9	Cerebral infarction, unspecified	0	0	0	1	0	0	0	0	0	0	0	0	0	0	0	0	0	0
I64	Stroke, not specified as haemorrhage or infarction	0	0	0	1	0	0	0	0	0	0	0	0	0	0	0	0	0	0
I65	Occlusion/stenos precerebral arts not result cerebrl infarct	0	0	0	1	0	0	0	0	0	0	0	0	0	0	0	0	0	0
I65.0	Occlusion and stenosis of vertebral artery	0	0	0	1	0	0	0	0	0	0	0	0	0	0	0	0	0	0
I65.1	Occlusion and stenosis of basilar artery	0	0	0	1	0	0	0	0	0	0	0	0	0	0	0	0	0	0
I65.2	Occlusion and stenosis of carotid artery	0	0	0	1	0	0	0	0	0	0	0	0	0	0	0	0	0	0
I65.3	Occlusion and stenosis of multip and bilat precerebrl arts	0	0	0	1	0	0	0	0	0	0	0	0	0	0	0	0	0	0
I65.8	Occlusion and stenosis of other precerebral artery	0	0	0	1	0	0	0	0	0	0	0	0	0	0	0	0	0	0
I65.9	Occlusion and stenosis of unspecified precerebral artery	0	0	0	1	0	0	0	0	0	0	0	0	0	0	0	0	0	0
I66	Occlusion/stenosis cerebral arts not result cerebral infarct	0	0	0	1	0	0	0	0	0	0	0	0	0	0	0	0	0	0
I66.0	Occlusion and stenosis of middle cerebral artery	0	0	0	1	0	0	0	0	0	0	0	0	0	0	0	0	0	0
I66.1	Occlusion and stenosis of anterior cerebral artery	0	0	0	1	0	0	0	0	0	0	0	0	0	0	0	0	0	0
I66.2	Occlusion and stenosis of posterior cerebral artery	0	0	0	1	0	0	0	0	0	0	0	0	0	0	0	0	0	0
I66.3	Occlusion and stenosis of cerebellar arteries	0	0	0	1	0	0	0	0	0	0	0	0	0	0	0	0	0	0

I66.4	Occlusion and stenosis of multiple and bilat cerebrl arts	0	0	0	1	0	0	0	0	0	0	0	0	0	0	0	0	0	0
I66.8	Occlusion and stenosis of other cerebral artery	0	0	0	1	0	0	0	0	0	0	0	0	0	0	0	0	0	0
I66.9	Occlusion and stenosis of unspecified cerebral artery	0	0	0	1	0	0	0	0	0	0	0	0	0	0	0	0	0	0
I67	Other cerebrovascular diseases	0	0	0	1	0	0	0	0	0	0	0	0	0	0	0	0	0	0
I67.0	Dissection of cerebral arteries, nonruptured	0	0	0	1	0	0	0	0	0	0	0	0	0	0	0	0	0	0
I67.1	Cerebral aneurysm, nonruptured	0	0	0	1	0	0	0	0	0	0	0	0	0	0	0	0	0	0
I67.2	Cerebral atherosclerosis	0	0	0	1	0	0	0	0	0	0	0	0	0	0	0	0	0	0
I67.3	Progressive vascular leukoencephalopathy	0	0	0	1	0	0	0	0	0	0	0	0	0	0	0	0	0	0
I67.4	Hypertensive encephalopathy	0	0	0	1	0	0	0	0	0	0	0	0	0	0	0	0	0	0
I67.5	Moyamoya disease	0	0	0	1	0	0	0	0	0	0	0	0	0	0	0	0	0	0
I67.6	Nonpyogenic thrombosis of intracranial venous system	0	0	0	1	0	0	0	0	0	0	0	0	0	0	0	0	0	0
I67.7	Cerebral arteritis, not elsewhere classified	0	0	0	1	0	0	0	0	0	0	0	0	0	0	0	0	0	0
I67.8	Other specified cerebrovascular diseases	0	0	0	1	0	0	0	0	0	0	0	0	0	0	0	0	0	0
I67.9	Cerebrovascular disease, unspecified	0	0	0	1	0	0	0	0	0	0	0	0	0	0	0	0	0	0
I68	Cerebrovascular disorders in diseases classified elsewhere	0	0	0	1	0	0	0	0	0	0	0	0	0	0	0	0	0	0
I68.0	Cerebral amyloid angiopathy	0	0	0	1	0	0	0	0	0	0	0	0	0	0	0	0	0	0
I68.1	Cerebral arteritis in infect & parasit dis classif elsewh	0	0	0	1	0	0	0	0	0	0	0	0	0	0	0	0	0	0
I68.2	Cerebral arteritis in other diseases classified elsewhere	0	0	0	1	0	0	0	0	0	0	0	0	0	0	0	0	0	0
I68.8	Other cerebrovascular disorders in diseases EC	0	0	0	1	0	0	0	0	0	0	0	0	0	0	0	0	0	0
I69	Sequelae of cerebrovascular disease	0	0	0	1	0	0	0	0	0	0	0	0	0	0	0	0	0	0
I69.0	Sequelae of subarachnoid haemorrhage	0	0	0	1	0	0	0	0	0	0	0	0	0	0	0	0	0	0
I69.1	Sequelae of intracerebral haemorrhage	0	0	0	1	0	0	0	0	0	0	0	0	0	0	0	0	0	0
I69.2	Sequelae of other nontraumatic intracranial haemorrhage	0	0	0	1	0	0	0	0	0	0	0	0	0	0	0	0	0	0
I69.3	Sequelae of cerebral infarction	0	0	0	1	0	0	0	0	0	0	0	0	0	0	0	0	0	0

I69.4	Sequelae of stroke, not spec as haemorrhage or infarction	0	0	0	1	0	0	0	0	0	0	0	0	0	0	0	0	0	0
I69.8	Sequelae of other and unspecified cerebrovascular diseases	0	0	0	1	0	0	0	0	0	0	0	0	0	0	0	0	0	0
G30	Alzheimer's disease	0	0	0	0	1	0	0	0	0	0	0	0	0	0	0	0	0	0
G30.0	Alzheimer's disease with early onset	0	0	0	0	1	0	0	0	0	0	0	0	0	0	0	0	0	0
G30.1	Alzheimer's disease with late onset	0	0	0	0	1	0	0	0	0	0	0	0	0	0	0	0	0	0
G30.8	Other Alzheimer's disease	0	0	0	0	1	0	0	0	0	0	0	0	0	0	0	0	0	0
G30.9	Alzheimer's disease, unspecified	0	0	0	0	1	0	0	0	0	0	0	0	0	0	0	0	0	0
F00	Dementia in Alzheimer's disease	0	0	0	0	1	0	0	0	0	0	0	0	0	0	0	0	0	0
F00.0	Dementia in Alzheimer's disease with early onset	0	0	0	0	1	0	0	0	0	0	0	0	0	0	0	0	0	0
F00.1	Dementia in Alzheimer's disease with late onset	0	0	0	0	1	0	0	0	0	0	0	0	0	0	0	0	0	0
F00.2	Dementia in Alzheimer's disease, atypical or mixed type	0	0	0	0	1	0	0	0	0	0	0	0	0	0	0	0	0	0
F00.9	Dementia in Alzheimer's disease, unspecified	0	0	0	0	1	0	0	0	0	0	0	0	0	0	0	0	0	0
F01	Vascular dementia	0	0	0	0	1	0	0	0	0	0	0	0	0	0	0	0	0	0
F01.0	Vascular dementia of acute onset	0	0	0	0	1	0	0	0	0	0	0	0	0	0	0	0	0	0
F01.1	Multi-infarct dementia	0	0	0	0	1	0	0	0	0	0	0	0	0	0	0	0	0	0
F01.2	Subcortical vascular dementia	0	0	0	0	1	0	0	0	0	0	0	0	0	0	0	0	0	0
F01.3	Mixed cortical and subcortical vascular dementia	0	0	0	0	1	0	0	0	0	0	0	0	0	0	0	0	0	0
F01.8	Other vascular dementia	0	0	0	0	1	0	0	0	0	0	0	0	0	0	0	0	0	0
F01.9	Vascular dementia, unspecified	0	0	0	0	1	0	0	0	0	0	0	0	0	0	0	0	0	0
F02	Dementia in other diseases classified elsewhere	0	0	0	0	1	0	0	0	0	0	0	0	0	0	0	0	0	0
F02.0	Dementia in Pick's disease	0	0	0	0	1	0	0	0	0	0	0	0	0	0	0	0	0	0
F02.1	Dementia in Creutzfeldt-Jakob disease	0	0	0	0	1	0	0	0	0	0	0	0	0	0	0	0	0	0
F02.2	Dementia in Huntington's disease	0	0	0	0	1	0	0	0	0	0	0	0	0	0	0	0	0	0
F02.3	Dementia in Parkinson's disease	0	0	0	0	1	0	0	0	0	0	0	0	0	0	0	0	0	0

F02.8	Dementia in other specified diseases classified elsewhere	0	0	0	0	1	0	0	0	0	0	0	0	0	0	0	0	0	0
F03	Unspecified dementia	0	0	0	0	1	0	0	0	0	0	0	0	0	0	0	0	0	0
F05.1	Delirium superimposed on dementia	0	0	0	0	1	0	0	0	0	0	0	0	0	0	0	0	0	0
J40	Bronchitis, not specified as acute or chronic	0	0	0	0	0	1	0	0	0	0	0	0	0	0	0	0	0	0
J41	Simple and mucopurulent chronic bronchitis	0	0	0	0	0	1	0	0	0	0	0	0	0	0	0	0	0	0
J41.0	Simple chronic bronchitis	0	0	0	0	0	1	0	0	0	0	0	0	0	0	0	0	0	0
J41.1	Mucopurulent chronic bronchitis	0	0	0	0	0	1	0	0	0	0	0	0	0	0	0	0	0	0
J41.8	Mixed simple and mucopurulent chronic bronchitis	0	0	0	0	0	1	0	0	0	0	0	0	0	0	0	0	0	0
J42	Unspecified chronic bronchitis	0	0	0	0	0	1	0	0	0	0	0	0	0	0	0	0	0	0
J43	Emphysema	0	0	0	0	0	1	0	0	0	0	0	0	0	0	0	0	0	0
J43.0	MacLeod's syndrome	0	0	0	0	0	1	0	0	0	0	0	0	0	0	0	0	0	0
J43.1	Panlobular emphysema	0	0	0	0	0	1	0	0	0	0	0	0	0	0	0	0	0	0
J43.2	Centrilobular emphysema	0	0	0	0	0	1	0	0	0	0	0	0	0	0	0	0	0	0
J43.8	Other emphysema	0	0	0	0	0	1	0	0	0	0	0	0	0	0	0	0	0	0
J43.9	Emphysema, unspecified	0	0	0	0	0	1	0	0	0	0	0	0	0	0	0	0	0	0
J44	Other chronic obstructive pulmonary disease	0	0	0	0	0	1	0	0	0	0	0	0	0	0	0	0	0	0
J44.0	Chronic obstruct pulmonary dis with acute lower resp infec	0	0	0	0	0	1	0	0	0	0	0	0	0	0	0	0	0	0
J44 1	Chron obstruct pulmonary dis wth acute exacerbation, unspec	0	0	0	0	0	1	0	0	0	0	0	0	0	0	0	0	0	0
J44.8	Other specified chronic obstructive pulmonary disease	0	0	0	0	0	1	0	0	0	0	0	0	0	0	0	0	0	0
J44.9	Chronic obstructive pulmonary disease, unspecified	0	0	0	0	0	1	0	0	0	0	0	0	0	0	0	0	0	0
J45	Asthma	0	0	0	0	0	1	0	0	0	0	0	0	0	0	0	0	0	0
J45.0	Predominantly allergic asthma	0	0	0	0	0	1	0	0	0	0	0	0	0	0	0	0	0	0
J45.1	Nonallergic asthma	0	0	0	0	0	1	0	0	0	0	0	0	0	0	0	0	0	0
J45.8	Mixed asthma	0	0	0	0	0	1	0	0	0	0	0	0	0	0	0	0	0	0

J45.9	Asthma, unspecified	0	0	0	0	0	1	0	0	0	0	0	0	0	0	0	0	0	0
J46	Status asthmaticus	0	0	0	0	0	1	0	0	0	0	0	0	0	0	0	0	0	0
J47	Bronchiectasis	0	0	0	0	0	1	0	0	0	0	0	0	0	0	0	0	0	0
J60	Coalworker's pneumoconiosis	0	0	0	0	0	1	0	0	0	0	0	0	0	0	0	0	0	0
J61	Pneumoconiosis due to asbestos and other mineral fibres	0	0	0	0	0	1	0	0	0	0	0	0	0	0	0	0	0	0
J62	Pneumoconiosis due to dust containing silica	0	0	0	0	0	1	0	0	0	0	0	0	0	0	0	0	0	0
J62.0	Pneumoconiosis due to talc dust	0	0	0	0	0	1	0	0	0	0	0	0	0	0	0	0	0	0
J62.8	Pneumoconiosis due to other dust containing silica	0	0	0	0	0	1	0	0	0	0	0	0	0	0	0	0	0	0
J63	Pneumoconiosis due to other inorganic dusts	0	0	0	0	0	1	0	0	0	0	0	0	0	0	0	0	0	0
J63.0	Aluminosis (of lung)	0	0	0	0	0	1	0	0	0	0	0	0	0	0	0	0	0	0
J63.1	Bauxite fibrosis (of lung)	0	0	0	0	0	1	0	0	0	0	0	0	0	0	0	0	0	0
J63.2	Berylliosis	0	0	0	0	0	1	0	0	0	0	0	0	0	0	0	0	0	0
J63.3	Graphite fibrosis (of lung)	0	0	0	0	0	1	0	0	0	0	0	0	0	0	0	0	0	0
J63.4	Siderosis	0	0	0	0	0	1	0	0	0	0	0	0	0	0	0	0	0	0
J63.5	Stannosis	0	0	0	0	0	1	0	0	0	0	0	0	0	0	0	0	0	0
J63.8	Pneumoconiosis due to other specified inorganic dusts	0	0	0	0	0	1	0	0	0	0	0	0	0	0	0	0	0	0
J64	Unspecified pneumoconiosis	0	0	0	0	0	1	0	0	0	0	0	0	0	0	0	0	0	0
J65	Pneumoconiosis associated with tuberculosis	0	0	0	0	0	1	0	0	0	0	0	0	0	0	0	0	0	0
J66	Airway disease due to specific organic dust	0	0	0	0	0	1	0	0	0	0	0	0	0	0	0	0	0	0
J66.0	Byssinosis	0	0	0	0	0	1	0	0	0	0	0	0	0	0	0	0	0	0
J66.1	Flax-dresser's disease	0	0	0	0	0	1	0	0	0	0	0	0	0	0	0	0	0	0
J66.2	Cannabinosis	0	0	0	0	0	1	0	0	0	0	0	0	0	0	0	0	0	0
J66.8	Airway disease due to other specific organic dusts	0	0	0	0	0	1	0	0	0	0	0	0	0	0	0	0	0	0
J67	Hypersensitivity pneumonitis due to organic dust	0	0	0	0	0	1	0	0	0	0	0	0	0	0	0	0	0	0

J67.0	Farmer's lung	0	0	0	0	0	1	0	0	0	0	0	0	0	0	0	0	0	0
J67.1	Bagassosis	0	0	0	0	0	1	0	0	0	0	0	0	0	0	0	0	0	0
J67.2	Bird fancier's lung	0	0	0	0	0	1	0	0	0	0	0	0	0	0	0	0	0	0
J67.3	Suberosis	0	0	0	0	0	1	0	0	0	0	0	0	0	0	0	0	0	0
J67.4	Maltworker's lung	0	0	0	0	0	1	0	0	0	0	0	0	0	0	0	0	0	0
J67.5	Mushroom-worker's lung	0	0	0	0	0	1	0	0	0	0	0	0	0	0	0	0	0	0
J67.6	Maple-bark-stripper's lung	0	0	0	0	0	1	0	0	0	0	0	0	0	0	0	0	0	0
J67.7	Air-conditioner and humidifier lung	0	0	0	0	0	1	0	0	0	0	0	0	0	0	0	0	0	0
J67.8	Hypersensitivity pneumonitis due to other organic dusts	0	0	0	0	0	1	0	0	0	0	0	0	0	0	0	0	0	0
J67.9	Hypersensitivity pneumonitis due to unspecified organic dust	0	0	0	0	0	1	0	0	0	0	0	0	0	0	0	0	0	0
J68.4	Chron resp condition due chemicals gas fume vapor	0	0	0	0	0	1	0	0	0	0	0	0	0	0	0	0	0	0
J70.1	Chronic and other pulmonary manifestations due to radiation	0	0	0	0	0	1	0	0	0	0	0	0	0	0	0	0	0	0
J70.3	Chronic drug-induced interstitial lung disorders	0	0	0	0	0	1	0	0	0	0	0	0	0	0	0	0	0	0
J84.1	Other interstitial pulmonary diseases with fibrosis	0	0	0	0	0	1	0	0	0	0	0	0	0	0	0	0	0	0
J92.0	Pleural plaque with presence of asbestos	0	0	0	0	0	1	0	0	0	0	0	0	0	0	0	0	0	0
J96.1	Chronic respiratory failure	0	0	0	0	0	1	0	0	0	0	0	0	0	0	0	0	0	0
J98.2	Interstitial emphysema	0	0	0	0	0	1	0	0	0	0	0	0	0	0	0	0	0	0
J98.3	Compensatory emphysema	0	0	0	0	0	1	0	0	0	0	0	0	0	0	0	0	0	0
M05	Seropositive rheumatoid arthritis	0	0	0	0	0	0	1	0	0	0	0	0	0	0	0	0	0	0
M05.0	Felty's syndrome	0	0	0	0	0	0	1	0	0	0	0	0	0	0	0	0	0	0
M05.1	Rheumatoid lung disease	0	0	0	0	0	0	1	0	0	0	0	0	0	0	0	0	0	0
M05.2	Rheumatoid vasculitis	0	0	0	0	0	0	1	0	0	0	0	0	0	0	0	0	0	0
M05.3	Rheumatoid arthritis with involvement of oth organs and sys	0	0	0	0	0	0	1	0	0	0	0	0	0	0	0	0	0	0
M05.8	Other seropositive rheumatoid arthritis	0	0	0	0	0	0	1	0	0	0	0	0	0	0	0	0	0	0

M05.9	Seropositive rheumatoid arthritis, unspecified	0	0	0	0	0	0	1	0	0	0	0	0	0	0	0	0	0	0
M06	Other rheumatoid arthritis	0	0	0	0	0	0	1	0	0	0	0	0	0	0	0	0	0	0
M06.0	Seronegative rheumatoid arthritis	0	0	0	0	0	0	1	0	0	0	0	0	0	0	0	0	0	0
M06.1	Adult-onset Still's disease	0	0	0	0	0	0	1	0	0	0	0	0	0	0	0	0	0	0
M06.2	Rheumatoid bursitis	0	0	0	0	0	0	1	0	0	0	0	0	0	0	0	0	0	0
M06.3	Rheumatoid nodule	0	0	0	0	0	0	1	0	0	0	0	0	0	0	0	0	0	0
M06.4	Inflammatory polyarthropathy	0	0	0	0	0	0	1	0	0	0	0	0	0	0	0	0	0	0
M06.8	Other specified rheumatoid arthritis	0	0	0	0	0	0	1	0	0	0	0	0	0	0	0	0	0	0
M06.9	Rheumatoid arthritis, unspecified	0	0	0	0	0	0	1	0	0	0	0	0	0	0	0	0	0	0
M08	Juvenile arthritis	0	0	0	0	0	0	1	0	0	0	0	0	0	0	0	0	0	0
M08.0	Juvenile rheumatoid arthritis	0	0	0	0	0	0	1	0	0	0	0	0	0	0	0	0	0	0
M08.1	Juvenile ankylosing spondylitis	0	0	0	0	0	0	1	0	0	0	0	0	0	0	0	0	0	0
M08.2	Juvenile arthritis with systemic onset	0	0	0	0	0	0	1	0	0	0	0	0	0	0	0	0	0	0
M08.3	Juvenile polyarthritis (seronegative)	0	0	0	0	0	0	1	0	0	0	0	0	0	0	0	0	0	0
M08.4	Pauciarticular juvenile arthritis	0	0	0	0	0	0	1	0	0	0	0	0	0	0	0	0	0	0
M08.8	Other juvenile arthritis	0	0	0	0	0	0	1	0	0	0	0	0	0	0	0	0	0	0
M08.9	Juvenile arthritis, unspecified	0	0	0	0	0	0	1	0	0	0	0	0	0	0	0	0	0	0
M09	Juvenile arthritis in diseases classified elsewhere	0	0	0	0	0	0	1	0	0	0	0	0	0	0	0	0	0	0
M09.0	Juvenile arthritis in psoriasis	0	0	0	0	0	0	1	0	0	0	0	0	0	0	0	0	0	0
M09.1	Juvenile arthritis in Crohn's disease [reg enteritis]	0	0	0	0	0	0	1	0	0	0	0	0	0	0	0	0	0	0
M09.2	Juvenile arthritis in ulcerative colitis	0	0	0	0	0	0	1	0	0	0	0	0	0	0	0	0	0	0
M09.8	Juvenile arthritis in other diseases classified elsewhere	0	0	0	0	0	0	1	0	0	0	0	0	0	0	0	0	0	0
M30	Polyarteritis nodosa and related conditions	0	0	0	0	0	0	1	0	0	0	0	0	0	0	0	0	0	0
M30.0	Polyarteritis nodosa	0	0	0	0	0	0	1	0	0	0	0	0	0	0	0	0	0	0

M3 0.1	Polyarteritis with lung involvement [Churg-Strauss]	0	0	0	0	0	0	1	0	0	0	0	0	0	0	0	0	0	0
M3 0.2	Juvenile polyarteritis	0	0	0	0	0	0	1	0	0	0	0	0	0	0	0	0	0	0
M3 0.3	Mucocutaneous lymph node syndrome [Kawasaki]	0	0	0	0	0	0	1	0	0	0	0	0	0	0	0	0	0	0
M3 0.8	Other conditions related to polyarteritis nodosa	0	0	0	0	0	0	1	0	0	0	0	0	0	0	0	0	0	0
M3 1	Other necrotizing vasculopathies	0	0	0	0	0	0	1	0	0	0	0	0	0	0	0	0	0	0
M3 1.0	Hypersensitivity angiitis	0	0	0	0	0	0	1	0	0	0	0	0	0	0	0	0	0	0
M3 1.1	Thrombotic microangiopathy	0	0	0	0	0	0	1	0	0	0	0	0	0	0	0	0	0	0
M3 1.2	Lethal midline granuloma	0	0	0	0	0	0	1	0	0	0	0	0	0	0	0	0	0	0
M3 1.3	Wegener's granulomatosis	0	0	0	0	0	0	1	0	0	0	0	0	0	0	0	0	0	0
M3 1.4	Aortic arch syndrome [Takayasu]	0	0	0	0	0	0	1	0	0	0	0	0	0	0	0	0	0	0
M3 1.5	Giant cell arteritis with polymyalgia rheumatica	0	0	0	0	0	0	1	0	0	0	0	0	0	0	0	0	0	0
M3 1.6	Other giant cell arteritis	0	0	0	0	0	0	1	0	0	0	0	0	0	0	0	0	0	0
M3 1.8	Other specified necrotizing vasculopathies	0	0	0	0	0	0	1	0	0	0	0	0	0	0	0	0	0	0
M3 1.9	Necrotizing vasculopathy, unspecified	0	0	0	0	0	0	1	0	0	0	0	0	0	0	0	0	0	0
M3 2	Systemic lupus erythematosus	0	0	0	0	0	0	1	0	0	0	0	0	0	0	0	0	0	0
M3 2.0	Drug-induced systemic lupus erythematosus	0	0	0	0	0	0	1	0	0	0	0	0	0	0	0	0	0	0
M3 2.1	Systemic lupus erythematosus with organ or sys involv	0	0	0	0	0	0	1	0	0	0	0	0	0	0	0	0	0	0
M3 2.8	Other forms of systemic lupus erythematosus	0	0	0	0	0	0	1	0	0	0	0	0	0	0	0	0	0	0
M3 2.9	Systemic lupus erythematosus, unspecified	0	0	0	0	0	0	1	0	0	0	0	0	0	0	0	0	0	0
M3 3	Dermatopolymyositis	0	0	0	0	0	0	1	0	0	0	0	0	0	0	0	0	0	0
M3 3.0	Juvenile dermatomyositis	0	0	0	0	0	0	1	0	0	0	0	0	0	0	0	0	0	0
M3 3.1	Other dermatomyositis	0	0	0	0	0	0	1	0	0	0	0	0	0	0	0	0	0	0
M3 3.2	Polymyositis	0	0	0	0	0	0	1	0	0	0	0	0	0	0	0	0	0	0
M3 3.9	Dermatopolymyositis, unspecified	0	0	0	0	0	0	1	0	0	0	0	0	0	0	0	0	0	0

M34	Systemic sclerosis	0	0	0	0	0	0	1	0	0	0	0	0	0	0	0	0	0	0
M34.0	Progressive systemic sclerosis	0	0	0	0	0	0	1	0	0	0	0	0	0	0	0	0	0	0
M34.1	CR(E)ST syndrome	0	0	0	0	0	0	1	0	0	0	0	0	0	0	0	0	0	0
M34.2	Systemic sclerosis induced by drugs and chemicals	0	0	0	0	0	0	1	0	0	0	0	0	0	0	0	0	0	0
M34.8	Other forms of systemic sclerosis	0	0	0	0	0	0	1	0	0	0	0	0	0	0	0	0	0	0
M34.9	Systemic sclerosis, unspecified	0	0	0	0	0	0	1	0	0	0	0	0	0	0	0	0	0	0
M35	Other systemic involvement of connective tissue	0	0	0	0	0	0	1	0	0	0	0	0	0	0	0	0	0	0
M35.0	Sicca syndrome [Sjogren]	0	0	0	0	0	0	1	0	0	0	0	0	0	0	0	0	0	0
M35.1	Other overlap syndromes	0	0	0	0	0	0	1	0	0	0	0	0	0	0	0	0	0	0
M35.2	Behcet's disease	0	0	0	0	0	0	1	0	0	0	0	0	0	0	0	0	0	0
M35.3	Polymyalgia rheumatica	0	0	0	0	0	0	1	0	0	0	0	0	0	0	0	0	0	0
M35.4	Diffuse (eosinophilic) fasciitis	0	0	0	0	0	0	1	0	0	0	0	0	0	0	0	0	0	0
M35.5	Multifocal fibrosclerosis	0	0	0	0	0	0	1	0	0	0	0	0	0	0	0	0	0	0
M35.6	Relapsing panniculitis [Weber-Christian]	0	0	0	0	0	0	1	0	0	0	0	0	0	0	0	0	0	0
M35.7	Hypermobility syndrome	0	0	0	0	0	0	1	0	0	0	0	0	0	0	0	0	0	0
M35.8	Other specified systemic involvement of connective tissue	0	0	0	0	0	0	1	0	0	0	0	0	0	0	0	0	0	0
M35.9	Systemic involvement of connective tissue, unspecified	0	0	0	0	0	0	1	0	0	0	0	0	0	0	0	0	0	0
M36	Systemic disorders of connective tissue in diseases EC	0	0	0	0	0	0	1	0	0	0	0	0	0	0	0	0	0	0
M36.0	Dermato(poly)myositis in neopastic disease	0	0	0	0	0	0	1	0	0	0	0	0	0	0	0	0	0	0
M36.1	Arthropathy in neopastic disease	0	0	0	0	0	0	1	0	0	0	0	0	0	0	0	0	0	0
M36.2	Haemophilic arthropathy	0	0	0	0	0	0	1	0	0	0	0	0	0	0	0	0	0	0
M36.3	Arthropathy in other blood disorders	0	0	0	0	0	0	1	0	0	0	0	0	0	0	0	0	0	0
M36.4	Arthropathy in hypersensitivity reactions EC	0	0	0	0	0	0	1	0	0	0	0	0	0	0	0	0	0	0
M36.8	Systemic disord of connective tissue in other diseases EC	0	0	0	0	0	0	1	0	0	0	0	0	0	0	0	0	0	0

D86	Sarcoidosis	0	0	0	0	0	0	1	0	0	0	0	0	0	0	0	0	0	0
D86 .0	Sarcoidosis of lung	0	0	0	0	0	0	1	0	0	0	0	0	0	0	0	0	0	0
D86 .1	Sarcoidosis of lymph nodes	0	0	0	0	0	0	1	0	0	0	0	0	0	0	0	0	0	0
D86 .2	Sarcoidosis of lung with sarcoidosis of lymph nodes	0	0	0	0	0	0	1	0	0	0	0	0	0	0	0	0	0	0
D86 .3	Sarcoidosis of skin	0	0	0	0	0	0	1	0	0	0	0	0	0	0	0	0	0	0
D86 .8	Sarcoidosis of other and combined sites	0	0	0	0	0	0	1	0	0	0	0	0	0	0	0	0	0	0
D86 .9	Sarcoidosis, unspecified	0	0	0	0	0	0	1	0	0	0	0	0	0	0	0	0	0	0
K22 .1	Ulcer of oesophagus	0	0	0	0	0	0	0	1	0	0	0	0	0	0	0	0	0	0
K25	Gastric ulcer	0	0	0	0	0	0	0	1	0	0	0	0	0	0	0	0	0	0
K25 .0	Gastric ulcer, acute with haemorrhage	0	0	0	0	0	0	0	1	0	0	0	0	0	0	0	0	0	0
K25 .1	Gastric ulcer, acute with perforation	0	0	0	0	0	0	0	1	0	0	0	0	0	0	0	0	0	0
K25 .2	Gastric ulcer, acute with both haemorrhage and perforation	0	0	0	0	0	0	0	1	0	0	0	0	0	0	0	0	0	0
K25 .3	Gastric ulcer, acute without haemorrhage or perforation	0	0	0	0	0	0	0	1	0	0	0	0	0	0	0	0	0	0
K25 .4	Gastric ulcer, chronic or unspecified with haemorrhage	0	0	0	0	0	0	0	1	0	0	0	0	0	0	0	0	0	0
K25 .5	Gastric ulcer, chronic or unspecified with perforation	0	0	0	0	0	0	0	1	0	0	0	0	0	0	0	0	0	0
K25 .6	Chronic or unspecified with both haemorrhage and perforation	0	0	0	0	0	0	0	1	0	0	0	0	0	0	0	0	0	0
K25 .7	Gastric ulcer, chronic without haemorrhage or perforation	0	0	0	0	0	0	0	1	0	0	0	0	0	0	0	0	0	0
K25 .9	Unspec as acute or chronic w'out haemorrhage or perforation	0	0	0	0	0	0	0	1	0	0	0	0	0	0	0	0	0	0
K26	Duodenal ulcer	0	0	0	0	0	0	0	1	0	0	0	0	0	0	0	0	0	0
K26 .0	Duodenal ulcer, acute with haemorrhage	0	0	0	0	0	0	0	1	0	0	0	0	0	0	0	0	0	0
K26 .1	Duodenal ulcer, acute with perforation	0	0	0	0	0	0	0	1	0	0	0	0	0	0	0	0	0	0
K26 .2	Duodenal ulcer, acute with both haemorrhage and perforation	0	0	0	0	0	0	0	1	0	0	0	0	0	0	0	0	0	0
K26 .3	Duodenal ulcer, acute without haemorrhage or perforation	0	0	0	0	0	0	0	1	0	0	0	0	0	0	0	0	0	0
K26 .4	Duodenal ulcer, chronic or unspecified with haemorrhage	0	0	0	0	0	0	0	1	0	0	0	0	0	0	0	0	0	0

K26.5	Duodenal ulcer, chronic or unspecified with perforation	0	0	0	0	0	0	0	1	0	0	0	0	0	0	0	0	0	0
K26.6	Chronic or unspecified with both haemorrhage and perforation	0	0	0	0	0	0	0	1	0	0	0	0	0	0	0	0	0	0
K26.7	Duodenal ulcer, chronic without haemorrhage or perforation	0	0	0	0	0	0	0	1	0	0	0	0	0	0	0	0	0	0
K26.9	Unspec as acute or chronic w'out haemorrhage or perforation	0	0	0	0	0	0	0	1	0	0	0	0	0	0	0	0	0	0
K27	Peptic ulcer, site unspecified	0	0	0	0	0	0	0	1	0	0	0	0	0	0	0	0	0	0
K27.0	Peptic ulcer, acute with haemorrhage	0	0	0	0	0	0	0	1	0	0	0	0	0	0	0	0	0	0
K27.1	Peptic ulcer, acute with perforation	0	0	0	0	0	0	0	1	0	0	0	0	0	0	0	0	0	0
K27.2	Peptic ulcer, acute with both haemorrhage and perforation	0	0	0	0	0	0	0	1	0	0	0	0	0	0	0	0	0	0
K27.3	Peptic ulcer, acute without haemorrhage or perforation	0	0	0	0	0	0	0	1	0	0	0	0	0	0	0	0	0	0
K27.4	Peptic ulcer, chronic or unspecified with haemorrhage	0	0	0	0	0	0	0	1	0	0	0	0	0	0	0	0	0	0
K27.5	Peptic ulcer, chronic or unspecified with perforation	0	0	0	0	0	0	0	1	0	0	0	0	0	0	0	0	0	0
K27.6	Chronic or unspecified with both haemorrhage and perforation	0	0	0	0	0	0	0	1	0	0	0	0	0	0	0	0	0	0
K27.7	Peptic ulcer, chronic without haemorrhage or perforation	0	0	0	0	0	0	0	1	0	0	0	0	0	0	0	0	0	0
K27.9	Unspec as acute or chronic w'out haemorrhage or perforation	0	0	0	0	0	0	0	1	0	0	0	0	0	0	0	0	0	0
K28	Gastrojejunal ulcer	0	0	0	0	0	0	0	1	0	0	0	0	0	0	0	0	0	0
K28.0	Gastrojejunal ulcer, acute with haemorrhage	0	0	0	0	0	0	0	1	0	0	0	0	0	0	0	0	0	0
K28.1	Gastrojejunal ulcer, acute with perforation	0	0	0	0	0	0	0	1	0	0	0	0	0	0	0	0	0	0
K28.2	Acute with both haemorrhage and perforation	0	0	0	0	0	0	0	1	0	0	0	0	0	0	0	0	0	0
K28.3	Acute without haemorrhage or perforation	0	0	0	0	0	0	0	1	0	0	0	0	0	0	0	0	0	0
K28.4	Gastrojejunal ulcer, chronic or unspecified with haemorrhage	0	0	0	0	0	0	0	1	0	0	0	0	0	0	0	0	0	0
K28.5	Gastrojejunal ulcer, chronic or unspecified with perforation	0	0	0	0	0	0	0	1	0	0	0	0	0	0	0	0	0	0
K28.6	Chronic or unspecified with both haemorrhage and perforation	0	0	0	0	0	0	0	1	0	0	0	0	0	0	0	0	0	0
K28.7	Chronic without haemorrhage or perforation	0	0	0	0	0	0	0	1	0	0	0	0	0	0	0	0	0	0
K28.9	Unspec as acute or chronic w'out haemorrhage or perforation	0	0	0	0	0	0	0	1	0	0	0	0	0	0	0	0	0	0

B18	Chronic viral hepatitis	0	0	0	0	0	0	0	0	1	0	0	0	0	0	0	0	0	0
B18.0	Chronic viral hepatitis B with delta-agent	0	0	0	0	0	0	0	0	1	0	0	0	0	0	0	0	0	0
B18.1	Chronic viral hepatitis B without delta-agent	0	0	0	0	0	0	0	0	1	0	0	0	0	0	0	0	0	0
B18.2	Chronic viral hepatitis C	0	0	0	0	0	0	0	0	1	0	0	0	0	0	0	0	0	0
B18.8	Other chronic viral hepatitis	0	0	0	0	0	0	0	0	1	0	0	0	0	0	0	0	0	0
B18.9	Chronic viral hepatitis, unspecified	0	0	0	0	0	0	0	0	1	0	0	0	0	0	0	0	0	0
K70.0	Alcoholic fatty liver	0	0	0	0	0	0	0	0	1	0	0	0	0	0	0	0	0	0
K70.1	Alcoholic hepatitis	0	0	0	0	0	0	0	0	1	0	0	0	0	0	0	0	0	0
K70.2	Alcoholic fibrosis and sclerosis of liver	0	0	0	0	0	0	0	0	1	0	0	0	0	0	0	0	0	0
K70.3	Alcoholic cirrhosis of liver	0	0	0	0	0	0	0	0	1	0	0	0	0	0	0	0	0	0
K70.9	Alcoholic liver disease, unspecified	0	0	0	0	0	0	0	0	1	0	0	0	0	0	0	0	0	0
K71	Toxic liver disease	0	0	0	0	0	0	0	0	1	0	0	0	0	0	0	0	0	0
K71.0	Toxic liver disease with cholestasis	0	0	0	0	0	0	0	0	1	0	0	0	0	0	0	0	0	0
K71.1	Toxic liver disease with hepatic necrosis	0	0	0	0	0	0	0	0	1	0	0	0	0	0	0	0	0	0
K71.2	Toxic liver disease with acute hepatitis	0	0	0	0	0	0	0	0	1	0	0	0	0	0	0	0	0	0
K71.3	Toxic liver disease with chronic persistent hepatitis	0	0	0	0	0	0	0	0	1	0	0	0	0	0	0	0	0	0
K71.4	Toxic liver disease with chronic lobular hepatitis	0	0	0	0	0	0	0	0	1	0	0	0	0	0	0	0	0	0
K71.5	Toxic liver disease with chronic active hepatitis	0	0	0	0	0	0	0	0	1	0	0	0	0	0	0	0	0	0
K71.6	Toxic liver disease with hepatitis, not elsewhere classified	0	0	0	0	0	0	0	0	1	0	0	0	0	0	0	0	0	0
K71.7	Toxic liver disease with fibrosis and cirrhosis of liver	0	0	0	0	0	0	0	0	1	0	0	0	0	0	0	0	0	0
K71.8	Toxic liver disease with other disorders of liver	0	0	0	0	0	0	0	0	1	0	0	0	0	0	0	0	0	0
K71.9	Toxic liver disease, unspecified	0	0	0	0	0	0	0	0	1	0	0	0	0	0	0	0	0	0
K73	Chronic hepatitis, not elsewhere classified	0	0	0	0	0	0	0	0	1	0	0	0	0	0	0	0	0	0
K73.0	Chronic persistent hepatitis, not elsewhere classified	0	0	0	0	0	0	0	0	1	0	0	0	0	0	0	0	0	0

K73 .1	Chronic lobular hepatitis, not elsewhere classified	0	0	0	0	0	0	0	0	1	0	0	0	0	0	0	0	0	0
K73 .2	Chronic active hepatitis, not elsewhere classified	0	0	0	0	0	0	0	0	1	0	0	0	0	0	0	0	0	0
K73 .8	Other chronic hepatitis, not elsewhere classified	0	0	0	0	0	0	0	0	1	0	0	0	0	0	0	0	0	0
K73 .9	Chronic hepatitis, unspecified	0	0	0	0	0	0	0	0	1	0	0	0	0	0	0	0	0	0
K74	Fibrosis and cirrhosis of liver	0	0	0	0	0	0	0	0	1	0	0	0	0	0	0	0	0	0
K74 .0	Hepatic fibrosis	0	0	0	0	0	0	0	0	1	0	0	0	0	0	0	0	0	0
K74 .1	Hepatic sclerosis	0	0	0	0	0	0	0	0	1	0	0	0	0	0	0	0	0	0
K74 .2	Hepatic fibrosis with hepatic sclerosis	0	0	0	0	0	0	0	0	1	0	0	0	0	0	0	0	0	0
K74 .3	Primary biliary cirrhosis	0	0	0	0	0	0	0	0	1	0	0	0	0	0	0	0	0	0
K74 .4	Secondary biliary cirrhosis	0	0	0	0	0	0	0	0	1	0	0	0	0	0	0	0	0	0
K74 .5	Biliary cirrhosis, unspecified	0	0	0	0	0	0	0	0	1	0	0	0	0	0	0	0	0	0
K74 .6	Other and unspecified cirrhosis of liver	0	0	0	0	0	0	0	0	1	0	0	0	0	0	0	0	0	0
K76 .0	Fatty (change of) liver, not elsewhere classified	0	0	0	0	0	0	0	0	1	0	0	0	0	0	0	0	0	0
G81	Hemiplegia	0	0	0	0	0	0	0	0	0	2	0	0	0	0	0	0	0	0
G81 .0	Flaccid hemiplegia	0	0	0	0	0	0	0	0	0	2	0	0	0	0	0	0	0	0
G81 .1	Spastic hemiplegia	0	0	0	0	0	0	0	0	0	2	0	0	0	0	0	0	0	0
G81 .9	Hemiplegia, unspecified	0	0	0	0	0	0	0	0	0	2	0	0	0	0	0	0	0	0
G82	Paraplegia and tetraplegia	0	0	0	0	0	0	0	0	0	2	0	0	0	0	0	0	0	0
G82 .0	Flaccid paraplegia	0	0	0	0	0	0	0	0	0	2	0	0	0	0	0	0	0	0
G82 .1	Spastic paraplegia	0	0	0	0	0	0	0	0	0	2	0	0	0	0	0	0	0	0
G82 .2	Paraplegia, unspecified	0	0	0	0	0	0	0	0	0	2	0	0	0	0	0	0	0	0
G82 .3	Flaccid tetraplegia	0	0	0	0	0	0	0	0	0	2	0	0	0	0	0	0	0	0
G82 .4	Spastic tetraplegia	0	0	0	0	0	0	0	0	0	2	0	0	0	0	0	0	0	0
G82 .5	Tetraplegia, unspecified	0	0	0	0	0	0	0	0	0	2	0	0	0	0	0	0	0	0

I12.0	Hypertensive renal disease with renal failure	0	0	0	0	0	0	0	0	0	0	2	0	0	0	0	0	0	0
I13.1	Hypertensive heart and renal disease with renal failure	0	0	0	0	0	0	0	0	0	0	2	0	0	0	0	0	0	0
I77.0	arteriovenous fistula, acquired	0	0	0	0	0	0	0	0	0	0	2	0	0	0	0	0	0	0
N16.5	renal tubulo-interstitial disorders in transplant rejection	0	0	0	0	0	0	0	0	0	0	2	0	0	0	0	0	0	0
N18.0	End-stage renal disease	0	0	0	0	0	0	0	0	0	0	2	0	0	0	0	0	0	0
N18.3	Chronic kidney disease, stage 3	0	0	0	0	0	0	0	0	0	0	2	0	0	0	0	0	0	0
N18.4	Chronic kidney disease, stage 4	0	0	0	0	0	0	0	0	0	0	2	0	0	0	0	0	0	0
N18.5	Chronic kidney disease, stage 5	0	0	0	0	0	0	0	0	0	0	2	0	0	0	0	0	0	0
N18.8	Other chronic renal failure	0	0	0	0	0	0	0	0	0	0	2	0	0	0	0	0	0	0
N18.9	Chronic renal failure, unspecified	0	0	0	0	0	0	0	0	0	0	2	0	0	0	0	0	0	0
N19	Unspecified kidney failure	0	0	0	0	0	0	0	0	0	0	2	0	0	0	0	0	0	0
T82.4	mechanical complication of vascular dialysis catheter	0	0	0	0	0	0	0	0	0	0	2	0	0	0	0	0	0	0
T86.1	kidney transplant failure and rejection	0	0	0	0	0	0	0	0	0	0	2	0	0	0	0	0	0	0
Y60.2	during kidney dialysis or other perfusion	0	0	0	0	0	0	0	0	0	0	2	0	0	0	0	0	0	0
Y61.2	during kidney dialysis or other perfusion	0	0	0	0	0	0	0	0	0	0	2	0	0	0	0	0	0	0
Y62.2	during kidney dialysis or other perfusion	0	0	0	0	0	0	0	0	0	0	2	0	0	0	0	0	0	0
Y84.1	kidney dialysis	0	0	0	0	0	0	0	0	0	0	2	0	0	0	0	0	0	0
Z49	care involving dialysis	0	0	0	0	0	0	0	0	0	0	2	0	0	0	0	0	0	0
Z49.0	preparatory care for dialysis	0	0	0	0	0	0	0	0	0	0	2	0	0	0	0	0	0	0
Z49.1	extracorporeal dialysis	0	0	0	0	0	0	0	0	0	0	2	0	0	0	0	0	0	0
Z49.2	other dialysis	0	0	0	0	0	0	0	0	0	0	2	0	0	0	0	0	0	0
Z94.0	kidney transplant status	0	0	0	0	0	0	0	0	0	0	2	0	0	0	0	0	0	0
Z99.2	dependence on renal dialysis	0	0	0	0	0	0	0	0	0	0	2	0	0	0	0	0	0	0
I13.2	Hyper heart and renal dis both (cong) heart and renal fail	0	0	0	0	0	0	0	0	0	0	2	0	0	0	0	0	0	0

M14.2	Diabetic arthropathy	0	0	0	0	0	0	0	0	0	0	0	2	0	0	0	0	0	0
H28.0	Diabetic cataract	0	0	0	0	0	0	0	0	0	0	0	2	0	0	0	0	0	0
G59.0	Diabetic mononeuropathy	0	0	0	0	0	0	0	0	0	0	0	2	0	0	0	0	0	0
G63.2	Diabetic polyneuropathy	0	0	0	0	0	0	0	0	0	0	0	2	0	0	0	0	0	0
H36.0	Diabetic retinopathy	0	0	0	0	0	0	0	0	0	0	0	2	0	0	0	0	0	0
N08.3	Glomerular disorders in diabetes mellitus	0	0	0	0	0	0	0	0	0	0	0	2	0	0	0	0	0	0
E10.7	Insulin-dependent diabetes mellitus with multiple comps	0	0	0	0	0	0	0	0	0	0	0	2	0	0	0	0	0	0
E10.4	Insulin-dependent diabetes mellitus with neurological comps	0	0	0	0	0	0	0	0	0	0	0	2	0	0	0	0	0	0
E10.3	Insulin-dependent diabetes mellitus with ophthalmic comps	0	0	0	0	0	0	0	0	0	0	0	2	0	0	0	0	0	0
E10.6	Insulin-dependent diabetes mellitus with other spec comps	0	0	0	0	0	0	0	0	0	0	0	2	0	0	0	0	0	0
E10.5	Insulin-dependent diabetes mellitus with periph circ comps	0	0	0	0	0	0	0	0	0	0	0	2	0	0	0	0	0	0
E10.2	Insulin-dependent diabetes mellitus with renal complications	0	0	0	0	0	0	0	0	0	0	0	2	0	0	0	0	0	0
E10.8	Insulin-dependent diabetes mellitus with unspec comps	0	0	0	0	0	0	0	0	0	0	0	2	0	0	0	0	0	0
E11.6	Non-insulin-depend diabetes mellitus with other spec comp	0	0	0	0	0	0	0	0	0	0	0	2	0	0	0	0	0	0
E11.5	Non-insulin-depend diabetes mellitus with periph circ comp	0	0	0	0	0	0	0	0	0	0	0	2	0	0	0	0	0	0
E11.7	Non-insulin-dependent diabetes mellitus with multiple comps	0	0	0	0	0	0	0	0	0	0	0	2	0	0	0	0	0	0
E11.4	Non-insulin-dependent diabetes mellitus with neuro comps	0	0	0	0	0	0	0	0	0	0	0	2	0	0	0	0	0	0
E11.3	Non-insulin-dependent diabetes mellitus with ophthalm comps	0	0	0	0	0	0	0	0	0	0	0	2	0	0	0	0	0	0
E11.2	Non-insulin-dependent diabetes mellitus with renal comps	0	0	0	0	0	0	0	0	0	0	0	2	0	0	0	0	0	0
E11.8	Non-insulin-dependent diabetes mellitus with unspec comps	0	0	0	0	0	0	0	0	0	0	0	2	0	0	0	0	0	0
E14.7	Unspecified diabetes mellitus with multiple complications	0	0	0	0	0	0	0	0	0	0	0	2	0	0	0	0	0	0
E14.4	Unspecified diabetes mellitus with neurological comps	0	0	0	0	0	0	0	0	0	0	0	2	0	0	0	0	0	0
E14.3	Unspecified diabetes mellitus with ophthalmic complications	0	0	0	0	0	0	0	0	0	0	0	2	0	0	0	0	0	0
E14.6	Unspecified diabetes mellitus with other specified comps	0	0	0	0	0	0	0	0	0	0	0	2	0	0	0	0	0	0

E14.5	Unspecified diabetes mellitus with periph circulatory comps	0	0	0	0	0	0	0	0	0	0	0	2	0	0	0	0	0	0
E14.2	Unspecified diabetes mellitus with renal complications	0	0	0	0	0	0	0	0	0	0	0	2	0	0	0	0	0	0
E14.8	Unspecified diabetes mellitus with unspecified complications	0	0	0	0	0	0	0	0	0	0	0	2	0	0	0	0	0	0
C00	Malignant neoplasm of lip	0	0	0	0	0	0	0	0	0	0	0	0	2	0	0	0	0	0
C00.0	Malignant neoplasm of external upper lip	0	0	0	0	0	0	0	0	0	0	0	0	2	0	0	0	0	0
C00.1	Malignant neoplasm of external lower lip	0	0	0	0	0	0	0	0	0	0	0	0	2	0	0	0	0	0
C00.2	Malignant neoplasm of external lip, unspecified	0	0	0	0	0	0	0	0	0	0	0	0	2	0	0	0	0	0
C00.3	Malignant neoplasm of upper lip, inner aspect	0	0	0	0	0	0	0	0	0	0	0	0	2	0	0	0	0	0
C00.4	Malignant neoplasm of lower lip, inner aspect	0	0	0	0	0	0	0	0	0	0	0	0	2	0	0	0	0	0
C00.5	Malignant neoplasm of lip, unspecified, inner aspect	0	0	0	0	0	0	0	0	0	0	0	0	2	0	0	0	0	0
C00.6	Malignant neoplasm of commissure of lip	0	0	0	0	0	0	0	0	0	0	0	0	2	0	0	0	0	0
C00.8	Malignant neoplasm of overlapping lesion of lip	0	0	0	0	0	0	0	0	0	0	0	0	2	0	0	0	0	0
C00.9	Malignant neoplasm of lip, unspecified	0	0	0	0	0	0	0	0	0	0	0	0	2	0	0	0	0	0
C01	Malignant neoplasm of base of tongue	0	0	0	0	0	0	0	0	0	0	0	0	2	0	0	0	0	0
C02	Malignant neoplasm of other and unspecified parts of tongue	0	0	0	0	0	0	0	0	0	0	0	0	2	0	0	0	0	0
C02.0	Malignant neoplasm of dorsal surface tongue	0	0	0	0	0	0	0	0	0	0	0	0	2	0	0	0	0	0
C02.1	Malignant neoplasm of border of tongue	0	0	0	0	0	0	0	0	0	0	0	0	2	0	0	0	0	0
C02.2	Malignant neoplasm of ventral surface of tongue	0	0	0	0	0	0	0	0	0	0	0	0	2	0	0	0	0	0
C02.3	Malignant neo of anterior two-thirds of tongue, part uns	0	0	0	0	0	0	0	0	0	0	0	0	2	0	0	0	0	0
C02.4	Malignant neoplasm of lingual tonsil	0	0	0	0	0	0	0	0	0	0	0	0	2	0	0	0	0	0
C02.8	Malignant neoplasm of overlapping lesion of tongue	0	0	0	0	0	0	0	0	0	0	0	0	2	0	0	0	0	0
C02.9	Malignant neoplasm of tongue, unspecified	0	0	0	0	0	0	0	0	0	0	0	0	2	0	0	0	0	0
C03	Malignant neoplasm of gum	0	0	0	0	0	0	0	0	0	0	0	0	2	0	0	0	0	0
C03.0	Malignant neoplasm of upper gum	0	0	0	0	0	0	0	0	0	0	0	0	2	0	0	0	0	0

C03.1	Malignant neoplasm of lower gum	0	0	0	0	0	0	0	0	0	0	0	0	2	0	0	0	0	0
C03.9	Malignant neoplasm of gum unspecified	0	0	0	0	0	0	0	0	0	0	0	0	2	0	0	0	0	0
C04	Malignant neoplasm of floor of mouth	0	0	0	0	0	0	0	0	0	0	0	0	2	0	0	0	0	0
C04.0	Malignant neoplasm of floor of anterior floor of mouth	0	0	0	0	0	0	0	0	0	0	0	0	2	0	0	0	0	0
C04.1	Malignant neoplasm of lateral floor of mouth	0	0	0	0	0	0	0	0	0	0	0	0	2	0	0	0	0	0
C04.8	Malignant neoplasm, overlapping lesion of floor of mouth	0	0	0	0	0	0	0	0	0	0	0	0	2	0	0	0	0	0
C04.9	Malignant neoplasm of floor of mouth, floor of mouth, unspec	0	0	0	0	0	0	0	0	0	0	0	0	2	0	0	0	0	0
C05	Malignant neoplasm of palate	0	0	0	0	0	0	0	0	0	0	0	0	2	0	0	0	0	0
C05.0	Malignant neoplasm of hard palate	0	0	0	0	0	0	0	0	0	0	0	0	2	0	0	0	0	0
C05.1	Malignant neoplasm of soft palate	0	0	0	0	0	0	0	0	0	0	0	0	2	0	0	0	0	0
C05.2	Malignant neoplasm of uvula	0	0	0	0	0	0	0	0	0	0	0	0	2	0	0	0	0	0
C05.8	Malignant neoplasm, overlapping lesion of palate	0	0	0	0	0	0	0	0	0	0	0	0	2	0	0	0	0	0
C05.9	Malignant neoplasm of palate, unspecified	0	0	0	0	0	0	0	0	0	0	0	0	2	0	0	0	0	0
C06	Malignant neoplasm of other and unspecified parts of mouth	0	0	0	0	0	0	0	0	0	0	0	0	2	0	0	0	0	0
C06.0	Malignant neoplasm cheek mucosa	0	0	0	0	0	0	0	0	0	0	0	0	2	0	0	0	0	0
C06.1	Malignant neoplasm of vestibule of mouth	0	0	0	0	0	0	0	0	0	0	0	0	2	0	0	0	0	0
C06.2	Malignant neoplasm of retromolar area	0	0	0	0	0	0	0	0	0	0	0	0	2	0	0	0	0	0
C06.8	Malignant neoplasm, overlap les of oth & unsp part of mouth	0	0	0	0	0	0	0	0	0	0	0	0	2	0	0	0	0	0
C06.9	Malignant neoplasm of part of mouth, unspecified	0	0	0	0	0	0	0	0	0	0	0	0	2	0	0	0	0	0
C07	Malignant neoplasm of parotid gland	0	0	0	0	0	0	0	0	0	0	0	0	2	0	0	0	0	0
C08	Maligt neoplasm of oth and unspec major saliv glands	0	0	0	0	0	0	0	0	0	0	0	0	2	0	0	0	0	0
C08.0	Malignant neoplasm of submandibular gland	0	0	0	0	0	0	0	0	0	0	0	0	2	0	0	0	0	0
C08.1	Malignant neoplasm of sublingual gland	0	0	0	0	0	0	0	0	0	0	0	0	2	0	0	0	0	0
C08.8	Malignant neoplasm, overlapping lesion of major saliv gland	0	0	0	0	0	0	0	0	0	0	0	0	2	0	0	0	0	0

C08 .9	Malignant neoplasm of major salivary gland, unspecified	0	0	0	0	0	0	0	0	0	0	0	0	2	0	0	0	0	0
C09	Malignant neoplasm of tonsil	0	0	0	0	0	0	0	0	0	0	0	0	2	0	0	0	0	0
C09 .0	Malignant neoplasm tonsillar fossa	0	0	0	0	0	0	0	0	0	0	0	0	2	0	0	0	0	0
C09 .1	Malig neo of tonsillar pillar (anterior)(posterior)	0	0	0	0	0	0	0	0	0	0	0	0	2	0	0	0	0	0
C09 .8	Malignant neoplasm of overlapping lesion of tonsil	0	0	0	0	0	0	0	0	0	0	0	0	2	0	0	0	0	0
C09 .9	Malignant neoplasm of tonsil unspecified	0	0	0	0	0	0	0	0	0	0	0	0	2	0	0	0	0	0
C10	Malignant neoplasm of oropharynx	0	0	0	0	0	0	0	0	0	0	0	0	2	0	0	0	0	0
C10 .0	Malignant neoplasm of vallecula	0	0	0	0	0	0	0	0	0	0	0	0	2	0	0	0	0	0
C10 .1	Malignant neoplasm of anterior surface of epiglottis	0	0	0	0	0	0	0	0	0	0	0	0	2	0	0	0	0	0
C10 .2	Malignant neoplasm of lateral wall of oropharynx	0	0	0	0	0	0	0	0	0	0	0	0	2	0	0	0	0	0
C10 .3	Malignant neoplasm of posterior wall of oropharynx	0	0	0	0	0	0	0	0	0	0	0	0	2	0	0	0	0	0
C10 .4	Malignant neoplasm of branchial cleft	0	0	0	0	0	0	0	0	0	0	0	0	2	0	0	0	0	0
C10 .8	Malignant neoplasm overlapping lesion of oropharynx	0	0	0	0	0	0	0	0	0	0	0	0	2	0	0	0	0	0
C10 .9	Malignant neoplasm of oropharynx unspecified	0	0	0	0	0	0	0	0	0	0	0	0	2	0	0	0	0	0
C11	Malignant neoplasm of nasopharynx	0	0	0	0	0	0	0	0	0	0	0	0	2	0	0	0	0	0
C11 .0	Malignant neoplasm of superior wall of nasopharynx	0	0	0	0	0	0	0	0	0	0	0	0	2	0	0	0	0	0
C11 .1	Malignant neoplasm of posterior wall of nasopharynx	0	0	0	0	0	0	0	0	0	0	0	0	2	0	0	0	0	0
C11 .2	Malignant neoplasm of lateral wall of nasopharynx	0	0	0	0	0	0	0	0	0	0	0	0	2	0	0	0	0	0
C11 .3	Malignant neoplasm of anterior wall of nasopharynx	0	0	0	0	0	0	0	0	0	0	0	0	2	0	0	0	0	0
C11 .8	Malignant neoplasm overlapping lesion of nasopharynx	0	0	0	0	0	0	0	0	0	0	0	0	2	0	0	0	0	0
C11 .9	Malignant neoplasm of nasopharynx unspecified	0	0	0	0	0	0	0	0	0	0	0	0	2	0	0	0	0	0
C12	Malignant neoplasm of pyriform sinus	0	0	0	0	0	0	0	0	0	0	0	0	2	0	0	0	0	0
C13	Malignant neoplasm of hypopharynx	0	0	0	0	0	0	0	0	0	0	0	0	2	0	0	0	0	0
C13 .0	Malignant neoplasm of hypopharynx, postcricoid region	0	0	0	0	0	0	0	0	0	0	0	0	2	0	0	0	0	0
C13 .1	Malig neoplasm aryepiglottic fold, hypopharyngeal aspect	0	0	0	0	0	0	0	0	0	0	0	0	2	0	0	0	0	0

C13.2	Malignant neoplasm posterior wall of hypopharynx	0	0	0	0	0	0	0	0	0	0	0	0	2	0	0	0	0	0
C13.8	Malignant neoplasm overlapping lesion of hypopharynx	0	0	0	0	0	0	0	0	0	0	0	0	2	0	0	0	0	0
C13.9	Malignant neoplasm of hypopharynx unspecified	0	0	0	0	0	0	0	0	0	0	0	0	2	0	0	0	0	0
C14	Mal neo oth ill-def sites lip/oral cavity/pharynx	0	0	0	0	0	0	0	0	0	0	0	0	2	0	0	0	0	0
C14.0	Malignant neoplasm of pharynx, unsp	0	0	0	0	0	0	0	0	0	0	0	0	2	0	0	0	0	0
C14.2	Malignant neoplasm of Waldeyer's ring	0	0	0	0	0	0	0	0	0	0	0	0	2	0	0	0	0	0
C14.8	Malig neo, overlapping lesion of lip, oral cavity & pharynx	0	0	0	0	0	0	0	0	0	0	0	0	2	0	0	0	0	0
C15	Malignant neoplasm of oesophagus	0	0	0	0	0	0	0	0	0	0	0	0	2	0	0	0	0	0
C15.0	Malignant neoplasm of cervical part of oesophagus	0	0	0	0	0	0	0	0	0	0	0	0	2	0	0	0	0	0
C15.1	Malignant neoplasm of thoracic part of oesophagus	0	0	0	0	0	0	0	0	0	0	0	0	2	0	0	0	0	0
C15.2	Malignant neo of abdominal part of oesophagus	0	0	0	0	0	0	0	0	0	0	0	0	2	0	0	0	0	0
C15.3	Malignant neoplasm of upper third of oesophagus	0	0	0	0	0	0	0	0	0	0	0	0	2	0	0	0	0	0
C15.4	Malignant neoplasm of middle third of oesophagus	0	0	0	0	0	0	0	0	0	0	0	0	2	0	0	0	0	0
C15.5	Malignant neoplasm of lower third of oesophagus	0	0	0	0	0	0	0	0	0	0	0	0	2	0	0	0	0	0
C15.8	Malignant neoplasm overlapping lesion of oesophagus	0	0	0	0	0	0	0	0	0	0	0	0	2	0	0	0	0	0
C15.9	Malignant neoplasm of oesophagus unspecified	0	0	0	0	0	0	0	0	0	0	0	0	2	0	0	0	0	0
C16	Malignant neoplasm of stomach	0	0	0	0	0	0	0	0	0	0	0	0	2	0	0	0	0	0
C16.0	Malignant neoplasm of cardia of stomach	0	0	0	0	0	0	0	0	0	0	0	0	2	0	0	0	0	0
C16.1	Malignant neoplasm of fundus of stomach	0	0	0	0	0	0	0	0	0	0	0	0	2	0	0	0	0	0
C16.2	Malignant neoplasm of body of stomach	0	0	0	0	0	0	0	0	0	0	0	0	2	0	0	0	0	0
C16.3	Malignant neoplasm of pyloric antrum	0	0	0	0	0	0	0	0	0	0	0	0	2	0	0	0	0	0
C16.4	Malignant neoplasm of pylorus	0	0	0	0	0	0	0	0	0	0	0	0	2	0	0	0	0	0
C16.5	Malignant neoplasm of lesser curvature of stomach, unsp	0	0	0	0	0	0	0	0	0	0	0	0	2	0	0	0	0	0
C16.6	Malignant neoplasm of greater curvature of stomach, unsp	0	0	0	0	0	0	0	0	0	0	0	0	2	0	0	0	0	0

C16.8	Malignant neoplasm overlapping lesion of stomach	0	0	0	0	0	0	0	0	0	0	0	0	2	0	0	0	0	0
C16.9	Malignant neoplasm of stomach, unspecified	0	0	0	0	0	0	0	0	0	0	0	0	2	0	0	0	0	0
C17	Malignant neoplasm of small intestine	0	0	0	0	0	0	0	0	0	0	0	0	2	0	0	0	0	0
C17.0	Malignant neoplasm of small intestine, duodenum	0	0	0	0	0	0	0	0	0	0	0	0	2	0	0	0	0	0
C17.1	Malignant neoplasm of small intestine, jejunum	0	0	0	0	0	0	0	0	0	0	0	0	2	0	0	0	0	0
C17.2	Malignant neoplasm of small intestine, ileum	0	0	0	0	0	0	0	0	0	0	0	0	2	0	0	0	0	0
C17.3	Malignant neoplasm of small intestine, Meckel's diverticulum	0	0	0	0	0	0	0	0	0	0	0	0	2	0	0	0	0	0
C17.8	Malignant neoplasm overlapping lesion of small intestine	0	0	0	0	0	0	0	0	0	0	0	0	2	0	0	0	0	0
C17.9	Malignant neoplasm of small intestine, unspecified	0	0	0	0	0	0	0	0	0	0	0	0	2	0	0	0	0	0
C18	Malignant neoplasm of colon	0	0	0	0	0	0	0	0	0	0	0	0	2	0	0	0	0	0
C18.0	Malignant neoplasm of caecum	0	0	0	0	0	0	0	0	0	0	0	0	2	0	0	0	0	0
C18.1	Malignant neoplasm of appendix	0	0	0	0	0	0	0	0	0	0	0	0	2	0	0	0	0	0
C18.2	Malignant neoplasm of ascending colon	0	0	0	0	0	0	0	0	0	0	0	0	2	0	0	0	0	0
C18.3	Malignant neoplasm of hepatic flexure	0	0	0	0	0	0	0	0	0	0	0	0	2	0	0	0	0	0
C18.4	Malignant neoplasm of transverse colon	0	0	0	0	0	0	0	0	0	0	0	0	2	0	0	0	0	0
C18.5	Malignant neoplasm of splenic flexure	0	0	0	0	0	0	0	0	0	0	0	0	2	0	0	0	0	0
C18.6	Malignant neoplasm of descending colon	0	0	0	0	0	0	0	0	0	0	0	0	2	0	0	0	0	0
C18.7	Malignant neoplasm of sigmoid colon	0	0	0	0	0	0	0	0	0	0	0	0	2	0	0	0	0	0
C18.8	Malignant neoplasm overlapping lesion of colon	0	0	0	0	0	0	0	0	0	0	0	0	2	0	0	0	0	0
C18.9	Malignant neoplasm of colon, unspecified	0	0	0	0	0	0	0	0	0	0	0	0	2	0	0	0	0	0
C19	Malignant neoplasm of rectosigmoid junction	0	0	0	0	0	0	0	0	0	0	0	0	2	0	0	0	0	0
C20	Malignant neoplasm of rectum	0	0	0	0	0	0	0	0	0	0	0	0	2	0	0	0	0	0
C21	Malignant neoplasm of anus and anal canal	0	0	0	0	0	0	0	0	0	0	0	0	2	0	0	0	0	0
C21.0	Malignant neoplasm of anus, unspecified	0	0	0	0	0	0	0	0	0	0	0	0	2	0	0	0	0	0

C21 .1	Malignant neoplasm of anal canal	0	0	0	0	0	0	0	0	0	0	0	0	2	0	0	0	0	0
C21 .2	Malignant neoplasm of cloacogenic zone	0	0	0	0	0	0	0	0	0	0	0	0	2	0	0	0	0	0
C21 .8	Malig neo, overlapping lesion of rectum, anus and anal canal	0	0	0	0	0	0	0	0	0	0	0	0	2	0	0	0	0	0
C22	Malignant neoplasm of liver and intrahepatic bile ducts	0	0	0	0	0	0	0	0	0	0	0	0	2	0	0	0	0	0
C22 .0	Malignant neoplasm, liver cell carcinoma	0	0	0	0	0	0	0	0	0	0	0	0	2	0	0	0	0	0
C22 .1	Malignant neoplasm, intrahep bile duct carcinoma	0	0	0	0	0	0	0	0	0	0	0	0	2	0	0	0	0	0
C22 .2	Malignant neoplasm, hepatoblastoma	0	0	0	0	0	0	0	0	0	0	0	0	2	0	0	0	0	0
C22 .3	Malignant neoplasm, angiosarcoma of liver	0	0	0	0	0	0	0	0	0	0	0	0	2	0	0	0	0	0
C22 .4	Malignant neoplasm, other sarcomas of liver	0	0	0	0	0	0	0	0	0	0	0	0	2	0	0	0	0	0
C22 .7	Malignant neoplasm, oth spec carcinomas of liver	0	0	0	0	0	0	0	0	0	0	0	0	2	0	0	0	0	0
C22 .9	Malignant neoplasm, liver, unspecified	0	0	0	0	0	0	0	0	0	0	0	0	2	0	0	0	0	0
C23	Malignant neoplasm of gallbladder	0	0	0	0	0	0	0	0	0	0	0	0	2	0	0	0	0	0
C24	Maligt neoplasm of other and unspec parts biliary tract	0	0	0	0	0	0	0	0	0	0	0	0	2	0	0	0	0	0
C24 .0	Malignant neoplasm of extrahepatic bile duct	0	0	0	0	0	0	0	0	0	0	0	0	2	0	0	0	0	0
C24 .1	Malignant neoplasm of Ampulla of Vater	0	0	0	0	0	0	0	0	0	0	0	0	2	0	0	0	0	0
C24 .8	Malignant neoplasm overlapping lesion of biliary tract	0	0	0	0	0	0	0	0	0	0	0	0	2	0	0	0	0	0
C24 .9	Malignant neoplasm of biliary tract, unspecified	0	0	0	0	0	0	0	0	0	0	0	0	2	0	0	0	0	0
C25	Malignant neoplasm of pancreas	0	0	0	0	0	0	0	0	0	0	0	0	2	0	0	0	0	0
C25 .0	Malignant neoplasm of head of pancreas	0	0	0	0	0	0	0	0	0	0	0	0	2	0	0	0	0	0
C25 .1	Malignant neoplasm of body of pancreas	0	0	0	0	0	0	0	0	0	0	0	0	2	0	0	0	0	0
C25 .2	Malignant neoplasm of tail of pancreas	0	0	0	0	0	0	0	0	0	0	0	0	2	0	0	0	0	0
C25 .3	Malignant neoplasm of pancreatic duct	0	0	0	0	0	0	0	0	0	0	0	0	2	0	0	0	0	0
C25 .4	Malignant neoplasm of endocrine pancreas	0	0	0	0	0	0	0	0	0	0	0	0	2	0	0	0	0	0
C25 .7	Malignant neoplasm of other parts of pancreas	0	0	0	0	0	0	0	0	0	0	0	0	2	0	0	0	0	0

C25 .8	Malignant neoplasm, overlapping lesion of pancreas	0	0	0	0	0	0	0	0	0	0	0	0	2	0	0	0	0	0
C25 .9	Malignant neoplasm of pancreas, unspecified	0	0	0	0	0	0	0	0	0	0	0	0	2	0	0	0	0	0
C26	Malignant neoplasm of other and ill-defined digestive organs	0	0	0	0	0	0	0	0	0	0	0	0	2	0	0	0	0	0
C26 .0	Malignant neoplasm of intestinal tract, part unsp	0	0	0	0	0	0	0	0	0	0	0	0	2	0	0	0	0	0
C26 .1	Malignant neoplasm of spleen	0	0	0	0	0	0	0	0	0	0	0	0	2	0	0	0	0	0
C26 .8	Malignant neoplasm, overlapping lesion of digestive system	0	0	0	0	0	0	0	0	0	0	0	0	2	0	0	0	0	0
C26 9	Malignant neoplasm of ill-def sites within digestive system	0	0	0	0	0	0	0	0	0	0	0	0	2	0	0	0	0	0
C30	Malignant neoplasm of nasal cavity and middle ear	0	0	0	0	0	0	0	0	0	0	0	0	2	0	0	0	0	0
C30 .0	Malignant neoplasm of nasal cavity	0	0	0	0	0	0	0	0	0	0	0	0	2	0	0	0	0	0
C30 .1	Malignant neoplasm of middle ear	0	0	0	0	0	0	0	0	0	0	0	0	2	0	0	0	0	0
C31	Malignant neoplasm of accessory sinuses	0	0	0	0	0	0	0	0	0	0	0	0	2	0	0	0	0	0
C31 .0	Malignant neoplasm of maxillary sinus	0	0	0	0	0	0	0	0	0	0	0	0	2	0	0	0	0	0
C31 .1	Malignant neoplasm of ethmoidal sinus	0	0	0	0	0	0	0	0	0	0	0	0	2	0	0	0	0	0
C31 .2	Malignant neoplasm of frontal sinus	0	0	0	0	0	0	0	0	0	0	0	0	2	0	0	0	0	0
C31 .3	Malignant neoplasm of sphenoidal sinus	0	0	0	0	0	0	0	0	0	0	0	0	2	0	0	0	0	0
C31 .8	Malignant neoplasm, overlapping lesion accessory sinuses	0	0	0	0	0	0	0	0	0	0	0	0	2	0	0	0	0	0
C31 .9	Malignant neoplasm of accessory sinus, unsp	0	0	0	0	0	0	0	0	0	0	0	0	2	0	0	0	0	0
C32	Malignant neoplasm of larynx	0	0	0	0	0	0	0	0	0	0	0	0	2	0	0	0	0	0
C32 .0	Malignant neoplasm of glottis	0	0	0	0	0	0	0	0	0	0	0	0	2	0	0	0	0	0
C32 .1	Malignant neoplasm of supraglottis	0	0	0	0	0	0	0	0	0	0	0	0	2	0	0	0	0	0
C32 .2	Malignant neoplasm of subglottis	0	0	0	0	0	0	0	0	0	0	0	0	2	0	0	0	0	0
C32 .3	Malignant neoplasm of laryngeal cartilage	0	0	0	0	0	0	0	0	0	0	0	0	2	0	0	0	0	0
C32 .8	Malignant neoplasm, overlapping lesion of larynx	0	0	0	0	0	0	0	0	0	0	0	0	2	0	0	0	0	0
C32 .9	Malignant neoplasm of larynx, unspecified	0	0	0	0	0	0	0	0	0	0	0	0	2	0	0	0	0	0

C33	Malignant neoplasm of trachea	0	0	0	0	0	0	0	0	0	0	0	0	2	0	0	0	0	0
C34	Malignant neoplasm of bronchus and lung	0	0	0	0	0	0	0	0	0	0	0	0	2	0	0	0	0	0
C34 .0	Malignant neoplasm of main bronchus	0	0	0	0	0	0	0	0	0	0	0	0	2	0	0	0	0	0
C34 .1	Malignant neoplasm of upper lobe, bronchus or lung	0	0	0	0	0	0	0	0	0	0	0	0	2	0	0	0	0	0
C34 .2	Malignant neoplasm of middle lobe, bronchus or lung	0	0	0	0	0	0	0	0	0	0	0	0	2	0	0	0	0	0
C34 .3	Malignant neoplasm of lower lobe, bronchus or lung	0	0	0	0	0	0	0	0	0	0	0	0	2	0	0	0	0	0
C34 .8	Malignant neoplasm of overlap les of bronchus & lung	0	0	0	0	0	0	0	0	0	0	0	0	2	0	0	0	0	0
C34 .9	Malignant neoplasm of bronchus or lung, unspec	0	0	0	0	0	0	0	0	0	0	0	0	2	0	0	0	0	0
C37	Malignant neoplasm of thymus	0	0	0	0	0	0	0	0	0	0	0	0	2	0	0	0	0	0
C38	Malignant neoplasm of heart, mediastinum and pleura	0	0	0	0	0	0	0	0	0	0	0	0	2	0	0	0	0	0
C38 .0	Malignant neoplasm of heart, mediastinum & pleura, heart	0	0	0	0	0	0	0	0	0	0	0	0	2	0	0	0	0	0
C38 .1	Malignant neoplasm of anterior mediastinum	0	0	0	0	0	0	0	0	0	0	0	0	2	0	0	0	0	0
C38 .2	Malignant neoplasm of posterior mediastinum	0	0	0	0	0	0	0	0	0	0	0	0	2	0	0	0	0	0
C38 .3	Malig neo heart, mediastinum & pleura,mediastinum,part unsp	0	0	0	0	0	0	0	0	0	0	0	0	2	0	0	0	0	0
C38 .4	Malignant neoplasm of pleura	0	0	0	0	0	0	0	0	0	0	0	0	2	0	0	0	0	0
C38 .8	Malig neo, overlapping lesion of heart, mediastinum & pleura	0	0	0	0	0	0	0	0	0	0	0	0	2	0	0	0	0	0
C39	Malig neo oth + ill-def sites resp sys + intrathorac orgs	0	0	0	0	0	0	0	0	0	0	0	0	2	0	0	0	0	0
C39 .0	Malignant neoplasm of upper respiratory tract, part unsp	0	0	0	0	0	0	0	0	0	0	0	0	2	0	0	0	0	0
C39 .8	Malignant neoplasm, overlap lesion of resp & intrathor orgs	0	0	0	0	0	0	0	0	0	0	0	0	2	0	0	0	0	0
C39 .9	Malignant neoplasm of ill-def sites within the resp sys	0	0	0	0	0	0	0	0	0	0	0	0	2	0	0	0	0	0
C40	Malignant neoplasm of bone and articular cartilage of limbs	0	0	0	0	0	0	0	0	0	0	0	0	2	0	0	0	0	0
C40 .0	Malignant neoplasm of scapula and long bones of upper limb	0	0	0	0	0	0	0	0	0	0	0	0	2	0	0	0	0	0
C40 .1	Malignant neoplasm of short bones of upper limb	0	0	0	0	0	0	0	0	0	0	0	0	2	0	0	0	0	0
C40 .2	Malignant neoplasm of long bones of lower limb	0	0	0	0	0	0	0	0	0	0	0	0	2	0	0	0	0	0

C40.3	Malignant neoplasm of short bones of lower limb	0	0	0	0	0	0	0	0	0	0	0	0	2	0	0	0	0	0
C40.8	Malignant neoplasm, overlap les bone and artic cart of limbs	0	0	0	0	0	0	0	0	0	0	0	0	2	0	0	0	0	0
C40.9	Malignant neoplasm of bone and artic cart of limb, unsp	0	0	0	0	0	0	0	0	0	0	0	0	2	0	0	0	0	0
C41	Malig neo bone + articular cartilage of oth + unspeci sites	0	0	0	0	0	0	0	0	0	0	0	0	2	0	0	0	0	0
C41.0	Malignant neoplasm of bones of skull and face	0	0	0	0	0	0	0	0	0	0	0	0	2	0	0	0	0	0
C41.1	Malignant neoplasm of mandible	0	0	0	0	0	0	0	0	0	0	0	0	2	0	0	0	0	0
C41.2	Malignant neoplasm of vertebral column	0	0	0	0	0	0	0	0	0	0	0	0	2	0	0	0	0	0
C41.3	Malignant neoplasm of ribs, sternum and clavicle	0	0	0	0	0	0	0	0	0	0	0	0	2	0	0	0	0	0
C41.4	Malignant neoplasm of sacrum and coccyx	0	0	0	0	0	0	0	0	0	0	0	0	2	0	0	0	0	0
C41.8	Malignant neoplasm, overlap lesion bon and articular cart	0	0	0	0	0	0	0	0	0	0	0	0	2	0	0	0	0	0
C41.9	Malignant neoplasm of bone and articular cartilage, unsp	0	0	0	0	0	0	0	0	0	0	0	0	2	0	0	0	0	0
C43	Malignant melanoma of skin	0	0	0	0	0	0	0	0	0	0	0	0	2	0	0	0	0	0
C43.0	Malignant melanoma of lip	0	0	0	0	0	0	0	0	0	0	0	0	2	0	0	0	0	0
C43.1	Malignant melanoma of eyelid, including canthus	0	0	0	0	0	0	0	0	0	0	0	0	2	0	0	0	0	0
C43.2	Malignant melanoma of ear and ext auricular canal	0	0	0	0	0	0	0	0	0	0	0	0	2	0	0	0	0	0
C43.3	Malignant melanoma of other and unspecified parts of face	0	0	0	0	0	0	0	0	0	0	0	0	2	0	0	0	0	0
C43.4	Malignant melanoma of scalp and neck	0	0	0	0	0	0	0	0	0	0	0	0	2	0	0	0	0	0
C43.5	Malignant melanoma of trunk	0	0	0	0	0	0	0	0	0	0	0	0	2	0	0	0	0	0
C43.6	Malignant melanoma of upper limb, including shoulder	0	0	0	0	0	0	0	0	0	0	0	0	2	0	0	0	0	0
C43.7	Malignant melanoma of lower limb, including hip	0	0	0	0	0	0	0	0	0	0	0	0	2	0	0	0	0	0
C43.8	Malignant melanoma of skin	0	0	0	0	0	0	0	0	0	0	0	0	2	0	0	0	0	0
C43.9	Malignant melanoma of skin, unsp	0	0	0	0	0	0	0	0	0	0	0	0	2	0	0	0	0	0
C44	Other malignant neoplasms of skin	0	0	0	0	0	0	0	0	0	0	0	0	2	0	0	0	0	0
C44.0	Other malignant neoplasms of skin of lip	0	0	0	0	0	0	0	0	0	0	0	0	2	0	0	0	0	0

C44 .1	Other malignant neoplasms of skin of eyelid, incl canthus	0	0	0	0	0	0	0	0	0	0	0	0	2	0	0	0	0	0
C44 .2	Oth malignant neoplasms of skin of ear & ext auricular canal	0	0	0	0	0	0	0	0	0	0	0	0	2	0	0	0	0	0
C44 .3	Oth malignant neoplasm of skin of oth & unsp parts of face	0	0	0	0	0	0	0	0	0	0	0	0	2	0	0	0	0	0
C44 .4	Other malignant neoplasms of skin of scalp and neck	0	0	0	0	0	0	0	0	0	0	0	0	2	0	0	0	0	0
C44 .5	Other malignant neoplasms of skin of trunk	0	0	0	0	0	0	0	0	0	0	0	0	2	0	0	0	0	0
C44 .6	Oth malignant neoplasms of skin of upper limb, incl shoulder	0	0	0	0	0	0	0	0	0	0	0	0	2	0	0	0	0	0
C44 .7	Other malignant neoplasms of skin of lower limb, incl hip	0	0	0	0	0	0	0	0	0	0	0	0	2	0	0	0	0	0
C44 .8	Other malignant neoplasms, overlapping lesion of skin	0	0	0	0	0	0	0	0	0	0	0	0	2	0	0	0	0	0
C44 .9	Other malignant neoplasms of skin, unspecified	0	0	0	0	0	0	0	0	0	0	0	0	2	0	0	0	0	0
C45	Mesothelioma	0	0	0	0	0	0	0	0	0	0	0	0	2	0	0	0	0	0
C45 .0	Mesothelioma of pleura	0	0	0	0	0	0	0	0	0	0	0	0	2	0	0	0	0	0
C45 .1	Mesothelioma of peritoneum	0	0	0	0	0	0	0	0	0	0	0	0	2	0	0	0	0	0
C45 .2	Mesothelioma of pericardium	0	0	0	0	0	0	0	0	0	0	0	0	2	0	0	0	0	0
C45 .7	Mesothelioma of other sites	0	0	0	0	0	0	0	0	0	0	0	0	2	0	0	0	0	0
C45 .9	Mesothelioma, unspecified	0	0	0	0	0	0	0	0	0	0	0	0	2	0	0	0	0	0
C46	Kaposi's sarcoma	0	0	0	0	0	0	0	0	0	0	0	0	2	0	0	0	0	0
C46 .0	Kaposi's sarcoma of skin	0	0	0	0	0	0	0	0	0	0	0	0	2	0	0	0	0	0
C46 .1	Kaposi's sarcoma of soft tissue	0	0	0	0	0	0	0	0	0	0	0	0	2	0	0	0	0	0
C46 .2	Kaposi's sarcoma of palate	0	0	0	0	0	0	0	0	0	0	0	0	2	0	0	0	0	0
C46 .3	Kaposi's sarcoma of lymph nodes	0	0	0	0	0	0	0	0	0	0	0	0	2	0	0	0	0	0
C46 .7	Kaposi's sarcoma of other sites	0	0	0	0	0	0	0	0	0	0	0	0	2	0	0	0	0	0
C46 .8	Kaposi's sarcoma of multiple organs	0	0	0	0	0	0	0	0	0	0	0	0	2	0	0	0	0	0
C46 .9	Kaposi's sarcoma, unspecified	0	0	0	0	0	0	0	0	0	0	0	0	2	0	0	0	0	0
C47	Malignant neo peripheral nerves and autonomic nervous syst	0	0	0	0	0	0	0	0	0	0	0	0	2	0	0	0	0	0

C47.0	Malignant neoplasm of peripheral nerve of head, face & neck	0	0	0	0	0	0	0	0	0	0	0	0	2	0	0	0	0	0
C47.1	Malignant neoplasm of peripheral nerve,upp limb,incl should	0	0	0	0	0	0	0	0	0	0	0	0	2	0	0	0	0	0
C47.2	Malignant neoplasm of peripheral nerve of low limb, incl hip	0	0	0	0	0	0	0	0	0	0	0	0	2	0	0	0	0	0
C47.3	Malignant neoplasm of peripheral nerve of thorax	0	0	0	0	0	0	0	0	0	0	0	0	2	0	0	0	0	0
C47.4	Malignant neoplasm of peripheral nerve of abdomen	0	0	0	0	0	0	0	0	0	0	0	0	2	0	0	0	0	0
C47.5	Malignant neoplasm of peripheral nerve of pelvis	0	0	0	0	0	0	0	0	0	0	0	0	2	0	0	0	0	0
C47.6	Malignant neoplasm of peripheral nerve of trunk, unspec	0	0	0	0	0	0	0	0	0	0	0	0	2	0	0	0	0	0
C47.8	Malignant neoplasm,overlap lesion periph nerve & auton ns	0	0	0	0	0	0	0	0	0	0	0	0	2	0	0	0	0	0
C47.9	Malignant neoplasm periph nerve & autonomic ns, unspec	0	0	0	0	0	0	0	0	0	0	0	0	2	0	0	0	0	0
C48	Malignant neoplasm of retroperitoneum and peritoneum	0	0	0	0	0	0	0	0	0	0	0	0	2	0	0	0	0	0
C48.0	Malignant neoplasm of retroperitoneum	0	0	0	0	0	0	0	0	0	0	0	0	2	0	0	0	0	0
C48.1	Malignant neoplasm of spec parts of peritoneum	0	0	0	0	0	0	0	0	0	0	0	0	2	0	0	0	0	0
C48.2	Malignant neoplasm of peritoneum, unsp	0	0	0	0	0	0	0	0	0	0	0	0	2	0	0	0	0	0
C48.8	Malignant neoplasm of overlap lesion retroperit & peritoneum	0	0	0	0	0	0	0	0	0	0	0	0	2	0	0	0	0	0
C49	Malignant neoplasm of other connective and soft tissue	0	0	0	0	0	0	0	0	0	0	0	0	2	0	0	0	0	0
C49.0	Malignant neoplasm of conn and soft tiss head, face & neck	0	0	0	0	0	0	0	0	0	0	0	0	2	0	0	0	0	0
C49.1	Malignant neoplasm of conn and soft tiss upp limb,inc should	0	0	0	0	0	0	0	0	0	0	0	0	2	0	0	0	0	0
C49.2	Malignant neoplasm of conn and soft tiss,lower limb,inc hip	0	0	0	0	0	0	0	0	0	0	0	0	2	0	0	0	0	0
C49.3	Malignant neoplasm of conn and soft tiss of thorax	0	0	0	0	0	0	0	0	0	0	0	0	2	0	0	0	0	0
C49.4	Malignant neoplasm of conn and soft tiss of abdomen	0	0	0	0	0	0	0	0	0	0	0	0	2	0	0	0	0	0
C49.5	Malignant neoplasm of conn and soft tiss of pelvis	0	0	0	0	0	0	0	0	0	0	0	0	2	0	0	0	0	0
C49.6	Malignant neoplasm of conn and soft tiss of trunk, unsp	0	0	0	0	0	0	0	0	0	0	0	0	2	0	0	0	0	0
C49.8	Malignant neoplasm, overlap lesion connective & soft tiss	0	0	0	0	0	0	0	0	0	0	0	0	2	0	0	0	0	0
C49.9	Malignant neoplasm of connective and soft tissue, unsp	0	0	0	0	0	0	0	0	0	0	0	0	2	0	0	0	0	0

C50	Malignant neoplasm of breast	0	0	0	0	0	0	0	0	0	0	0	0	2	0	0	0	0	0
C50.0	Malignant neoplasm of nipple and areola	0	0	0	0	0	0	0	0	0	0	0	0	2	0	0	0	0	0
C50.1	Malignant neoplasm of central portion of breast	0	0	0	0	0	0	0	0	0	0	0	0	2	0	0	0	0	0
C50.2	Malignant neoplasm of upper-inner quadrant of breast	0	0	0	0	0	0	0	0	0	0	0	0	2	0	0	0	0	0
C50.3	Malignant neoplasm of lower-inner quadrant of breast	0	0	0	0	0	0	0	0	0	0	0	0	2	0	0	0	0	0
C50.4	Malignant neoplasm of upper-outer quadrant of breast	0	0	0	0	0	0	0	0	0	0	0	0	2	0	0	0	0	0
C50.5	Malignant neoplasm of lower-outer quadrant of breast	0	0	0	0	0	0	0	0	0	0	0	0	2	0	0	0	0	0
C50.6	Malignant neoplasm of axillary tail of breast	0	0	0	0	0	0	0	0	0	0	0	0	2	0	0	0	0	0
C50.8	Malignant neoplasm, overlapping lesion of breast	0	0	0	0	0	0	0	0	0	0	0	0	2	0	0	0	0	0
C50.9	Malignant neoplasm of breast, unspecified	0	0	0	0	0	0	0	0	0	0	0	0	2	0	0	0	0	0
C51	Malignant neoplasm of vulva	0	0	0	0	0	0	0	0	0	0	0	0	2	0	0	0	0	0
C51.0	Malignant neoplasm of labium majus	0	0	0	0	0	0	0	0	0	0	0	0	2	0	0	0	0	0
C51.1	Malignant neoplasm of labium minus	0	0	0	0	0	0	0	0	0	0	0	0	2	0	0	0	0	0
C51.2	Malignant neoplasm of clitoris	0	0	0	0	0	0	0	0	0	0	0	0	2	0	0	0	0	0
C51.8	Malignant neoplasm of overlapping lesion of vulva	0	0	0	0	0	0	0	0	0	0	0	0	2	0	0	0	0	0
C51.9	Malignant neoplasm of vulva, unspecified	0	0	0	0	0	0	0	0	0	0	0	0	2	0	0	0	0	0
C52	Malignant neoplasm of vagina	0	0	0	0	0	0	0	0	0	0	0	0	2	0	0	0	0	0
C53	Malignant neoplasm of cervix uteri	0	0	0	0	0	0	0	0	0	0	0	0	2	0	0	0	0	0
C53.0	Malignant neoplasm of endocervix	0	0	0	0	0	0	0	0	0	0	0	0	2	0	0	0	0	0
C53.1	Malignant neoplasm of exocervix	0	0	0	0	0	0	0	0	0	0	0	0	2	0	0	0	0	0
C53.8	Malignant neoplasm, overlapping lesion of cervix uteri	0	0	0	0	0	0	0	0	0	0	0	0	2	0	0	0	0	0
C53.9	Malignant neoplasm of cervix uteri, unsp	0	0	0	0	0	0	0	0	0	0	0	0	2	0	0	0	0	0
C54	Malignant neoplasm of corpus uteri	0	0	0	0	0	0	0	0	0	0	0	0	2	0	0	0	0	0
C54.0	Malignant neoplasm of isthmus uteri	0	0	0	0	0	0	0	0	0	0	0	0	2	0	0	0	0	0
C54.1	Malignant neoplasm of endometrium	0	0	0	0	0	0	0	0	0	0	0	0	2	0	0	0	0	0

C54.2	Malignant neoplasm of myometrium	0	0	0	0	0	0	0	0	0	0	0	0	2	0	0	0	0	0
C54.3	Malignant neoplasm of fundus uteri	0	0	0	0	0	0	0	0	0	0	0	0	2	0	0	0	0	0
C54.8	Malignant neoplasm overlapping lesion of corpus uteri	0	0	0	0	0	0	0	0	0	0	0	0	2	0	0	0	0	0
C54.9	Malignant neoplasm of corpus uteri, unsp	0	0	0	0	0	0	0	0	0	0	0	0	2	0	0	0	0	0
C55	Malignant neoplasm of uterus, part unspecified	0	0	0	0	0	0	0	0	0	0	0	0	2	0	0	0	0	0
C56	Malignant neoplasm of ovary	0	0	0	0	0	0	0	0	0	0	0	0	2	0	0	0	0	0
C57	Malignant neoplasm of other and unspec female genital orgs	0	0	0	0	0	0	0	0	0	0	0	0	2	0	0	0	0	0
C57.0	Malignant neoplasm of fallopian tube	0	0	0	0	0	0	0	0	0	0	0	0	2	0	0	0	0	0
C57.1	Malignant neoplasm of broad ligament	0	0	0	0	0	0	0	0	0	0	0	0	2	0	0	0	0	0
C57.2	Malignant neoplasm of round ligament	0	0	0	0	0	0	0	0	0	0	0	0	2	0	0	0	0	0
C57.3	Malignant neoplasm of parametrium	0	0	0	0	0	0	0	0	0	0	0	0	2	0	0	0	0	0
C57.4	Malignant neoplasm of uterine adnexa, unsp	0	0	0	0	0	0	0	0	0	0	0	0	2	0	0	0	0	0
C57.7	Malignant neoplasm of other specified female genital organs	0	0	0	0	0	0	0	0	0	0	0	0	2	0	0	0	0	0
C57.8	Malignant neoplasm, overlapping lesion female genital organs	0	0	0	0	0	0	0	0	0	0	0	0	2	0	0	0	0	0
C57.9	Malignant neoplasm of female genital organ, unspecified	0	0	0	0	0	0	0	0	0	0	0	0	2	0	0	0	0	0
C58	Malignant neoplasm of placenta	0	0	0	0	0	0	0	0	0	0	0	0	2	0	0	0	0	0
C60	Malignant neoplasm of penis	0	0	0	0	0	0	0	0	0	0	0	0	2	0	0	0	0	0
C60.0	Malignant neoplasm of prepuce	0	0	0	0	0	0	0	0	0	0	0	0	2	0	0	0	0	0
C60.1	Malignant neoplasm of glans penis	0	0	0	0	0	0	0	0	0	0	0	0	2	0	0	0	0	0
C60.2	Malignant neoplasm of body of penis	0	0	0	0	0	0	0	0	0	0	0	0	2	0	0	0	0	0
C60.8	Malignant neoplasm, overlapping lesion of penis	0	0	0	0	0	0	0	0	0	0	0	0	2	0	0	0	0	0
C60.9	Malignant neoplasm of penis, unspecified	0	0	0	0	0	0	0	0	0	0	0	0	2	0	0	0	0	0
C61	Malignant neoplasm of prostate	0	0	0	0	0	0	0	0	0	0	0	0	2	0	0	0	0	0
C62	Malignant neoplasm of testis	0	0	0	0	0	0	0	0	0	0	0	0	2	0	0	0	0	0
C62.0	Malignant neoplasm of undescended testis	0	0	0	0	0	0	0	0	0	0	0	0	2	0	0	0	0	0

C62 .1	Malignant neoplasm of descended testis	0	0	0	0	0	0	0	0	0	0	0	0	2	0	0	0	0	0
C62 .9	Malignant neoplasm of testis, unspecified	0	0	0	0	0	0	0	0	0	0	0	0	2	0	0	0	0	0
C63	Malignant neoplasm of other and unspec male genital organs	0	0	0	0	0	0	0	0	0	0	0	0	2	0	0	0	0	0
C63 .0	Malignant neoplasm of epididymis	0	0	0	0	0	0	0	0	0	0	0	0	2	0	0	0	0	0
C63 .1	Malignant neoplasm of spermatic cord	0	0	0	0	0	0	0	0	0	0	0	0	2	0	0	0	0	0
C63 .2	Malignant neoplasm of scrotum	0	0	0	0	0	0	0	0	0	0	0	0	2	0	0	0	0	0
C63 .7	Malignant neoplasm of other specified male genital orgs	0	0	0	0	0	0	0	0	0	0	0	0	2	0	0	0	0	0
C63 .8	Malignant neoplasm, overlapping lesion male genital orgs	0	0	0	0	0	0	0	0	0	0	0	0	2	0	0	0	0	0
C63 .9	Malignant neoplasm of male genital organ, unspecified	0	0	0	0	0	0	0	0	0	0	0	0	2	0	0	0	0	0
C64	Malignant neoplasm of kidney, except renal pelvis	0	0	0	0	0	0	0	0	0	0	0	0	2	0	0	0	0	0
C65	Malignant neoplasm of renal pelvis	0	0	0	0	0	0	0	0	0	0	0	0	2	0	0	0	0	0
C66	Malignant neoplasm of ureter	0	0	0	0	0	0	0	0	0	0	0	0	2	0	0	0	0	0
C67	Malignant neoplasm of bladder	0	0	0	0	0	0	0	0	0	0	0	0	2	0	0	0	0	0
C67 .0	Malignant neoplasm of trigone of bladder	0	0	0	0	0	0	0	0	0	0	0	0	2	0	0	0	0	0
C67 .1	Malignant neoplasm of dome of bladder	0	0	0	0	0	0	0	0	0	0	0	0	2	0	0	0	0	0
C67 .2	Malignant neoplasm of lateral wall of bladder	0	0	0	0	0	0	0	0	0	0	0	0	2	0	0	0	0	0
C67 .3	Malignant neoplasm of anterior wall of bladder	0	0	0	0	0	0	0	0	0	0	0	0	2	0	0	0	0	0
C67 .4	Malignant neoplasm of posterior wall of bladder	0	0	0	0	0	0	0	0	0	0	0	0	2	0	0	0	0	0
C67 .5	Malignant neoplasm of bladder neck	0	0	0	0	0	0	0	0	0	0	0	0	2	0	0	0	0	0
C67 .6	Malignant neoplasm of ureteric orifice	0	0	0	0	0	0	0	0	0	0	0	0	2	0	0	0	0	0
C67 .7	Malignant neoplasm of urachus	0	0	0	0	0	0	0	0	0	0	0	0	2	0	0	0	0	0
C67 .8	Malignant neoplasm, overlapping lesion of bladder	0	0	0	0	0	0	0	0	0	0	0	0	2	0	0	0	0	0
C67 .9	Malignant neoplasm of bladder, unspecified	0	0	0	0	0	0	0	0	0	0	0	0	2	0	0	0	0	0
C68	Malignant neoplasm of other and unspecified urinary organs	0	0	0	0	0	0	0	0	0	0	0	0	2	0	0	0	0	0

C68 .0	Malignant neoplasm of urethra	0	0	0	0	0	0	0	0	0	0	0	0	2	0	0	0	0	0
C68 .1	Malignant neoplasm of paraurethral gland	0	0	0	0	0	0	0	0	0	0	0	0	2	0	0	0	0	0
C68 .8	Malignant neoplasm of overlapping lesion urinary organs	0	0	0	0	0	0	0	0	0	0	0	0	2	0	0	0	0	0
C68 .9	Malignant neoplasm of urinary organ, unspecified	0	0	0	0	0	0	0	0	0	0	0	0	2	0	0	0	0	0
C69	Malignant neoplasm of eye and adnexa	0	0	0	0	0	0	0	0	0	0	0	0	2	0	0	0	0	0
C69 .0	Malignant neoplasm of conjunctiva	0	0	0	0	0	0	0	0	0	0	0	0	2	0	0	0	0	0
C69 .1	Malignant neoplasm of cornea	0	0	0	0	0	0	0	0	0	0	0	0	2	0	0	0	0	0
C69 .2	Malignant neoplasm of retina	0	0	0	0	0	0	0	0	0	0	0	0	2	0	0	0	0	0
C69 .3	Malignant neoplasm of choroid	0	0	0	0	0	0	0	0	0	0	0	0	2	0	0	0	0	0
C69 .4	Malignant neoplasm of ciliary body	0	0	0	0	0	0	0	0	0	0	0	0	2	0	0	0	0	0
C69 .5	Malignant neoplasm of lacrimal gland and duct	0	0	0	0	0	0	0	0	0	0	0	0	2	0	0	0	0	0
C69 .6	Malignant neoplasm of orbit	0	0	0	0	0	0	0	0	0	0	0	0	2	0	0	0	0	0
C69 .8	Malignant neoplasm, overlapping lesion eye and adnexa	0	0	0	0	0	0	0	0	0	0	0	0	2	0	0	0	0	0
C69 .9	Malignant neoplasm of eye, unspecified	0	0	0	0	0	0	0	0	0	0	0	0	2	0	0	0	0	0
C70	Malignant neoplasm of meninges	0	0	0	0	0	0	0	0	0	0	0	0	2	0	0	0	0	0
C70 .0	Malignant neoplasm of, cerebral meninges	0	0	0	0	0	0	0	0	0	0	0	0	2	0	0	0	0	0
C70 .1	Malignant neoplasm of spinal meninges	0	0	0	0	0	0	0	0	0	0	0	0	2	0	0	0	0	0
C70 .9	Malignant neoplasm of meninges, unspecified	0	0	0	0	0	0	0	0	0	0	0	0	2	0	0	0	0	0
C71	Malignant neoplasm of brain	0	0	0	0	0	0	0	0	0	0	0	0	2	0	0	0	0	0
C71 .0	Malignant neoplasm of cerebrum, except lobes & ventricles	0	0	0	0	0	0	0	0	0	0	0	0	2	0	0	0	0	0
C71 .1	Malignant neoplasm of cerebrum, frontal lobe	0	0	0	0	0	0	0	0	0	0	0	0	2	0	0	0	0	0
C71 .2	Malignant neoplasm of cerebrum, temporal lobe	0	0	0	0	0	0	0	0	0	0	0	0	2	0	0	0	0	0
C71 .3	Malignant neoplasm of cerebrum, parietal lobe	0	0	0	0	0	0	0	0	0	0	0	0	2	0	0	0	0	0
C71 .4	Malignant neoplasm of cerebrum, occipital lobe	0	0	0	0	0	0	0	0	0	0	0	0	2	0	0	0	0	0

C71.5	Malignant neoplasm of cerebrum, cerebral ventricle	0	0	0	0	0	0	0	0	0	0	0	0	2	0	0	0	0	0
C71.6	Malignant neoplasm of cerebrum, cerebellum	0	0	0	0	0	0	0	0	0	0	0	0	2	0	0	0	0	0
C71.7	Malignant neoplasm of cerebrum, brain stem	0	0	0	0	0	0	0	0	0	0	0	0	2	0	0	0	0	0
C71.8	Malignant neoplasm of cerebrum, overlapping lesion of brain	0	0	0	0	0	0	0	0	0	0	0	0	2	0	0	0	0	0
C71.9	Malignant neoplasm of cerebrum, brain, unspecified	0	0	0	0	0	0	0	0	0	0	0	0	2	0	0	0	0	0
C72	Malig neopl spinal cord, cranial nerves & oth parts of CNS	0	0	0	0	0	0	0	0	0	0	0	0	2	0	0	0	0	0
C72.0	Malignant neoplasm of spinal cord	0	0	0	0	0	0	0	0	0	0	0	0	2	0	0	0	0	0
C72.1	Malignant neoplasm of cauda equina	0	0	0	0	0	0	0	0	0	0	0	0	2	0	0	0	0	0
C72.2	Malignant neoplasm of Olfactory nerve	0	0	0	0	0	0	0	0	0	0	0	0	2	0	0	0	0	0
C72.3	Malignant neoplasm of Optic nerve	0	0	0	0	0	0	0	0	0	0	0	0	2	0	0	0	0	0
C72.4	Malignant neoplasm of Acoustic nerve	0	0	0	0	0	0	0	0	0	0	0	0	2	0	0	0	0	0
C72.5	Malignant neoplasm of other and unspecified cranial nerves	0	0	0	0	0	0	0	0	0	0	0	0	2	0	0	0	0	0
C72.8	Malignant neoplasm, overlapping lesion brain&other part CNS	0	0	0	0	0	0	0	0	0	0	0	0	2	0	0	0	0	0
C72.9	Malignant neoplasm of Central Nervous System, unspecified	0	0	0	0	0	0	0	0	0	0	0	0	2	0	0	0	0	0
C73	Malignant neoplasm of thyroid gland	0	0	0	0	0	0	0	0	0	0	0	0	2	0	0	0	0	0
C74	Malignant neoplasm of adrenal gland	0	0	0	0	0	0	0	0	0	0	0	0	2	0	0	0	0	0
C74.0	Malignant neoplasm of cortex of adrenal gland	0	0	0	0	0	0	0	0	0	0	0	0	2	0	0	0	0	0
C74.1	Malignant neoplasm of medulla of adrenal gland	0	0	0	0	0	0	0	0	0	0	0	0	2	0	0	0	0	0
C74.9	Malignant neoplasm of adrenal gland, unsp	0	0	0	0	0	0	0	0	0	0	0	0	2	0	0	0	0	0
C75	Malignant neo other endocrine glands and related structures	0	0	0	0	0	0	0	0	0	0	0	0	2	0	0	0	0	0
C75.0	Malignant neoplasm of parathyroid gland	0	0	0	0	0	0	0	0	0	0	0	0	2	0	0	0	0	0
C75.1	Malignant neoplasm of pituitary gland	0	0	0	0	0	0	0	0	0	0	0	0	2	0	0	0	0	0
C75.2	Malignant neoplasm of craniopharyngeal duct	0	0	0	0	0	0	0	0	0	0	0	0	2	0	0	0	0	0
C75.3	Malignant neoplasm of pineal gland	0	0	0	0	0	0	0	0	0	0	0	0	2	0	0	0	0	0

C75 .4	Malignant neoplasm of carotid body	0	0	0	0	0	0	0	0	0	0	0	0	2	0	0	0	0	0
C75 .5	Malignant neoplasm of aortic body and other paraganglia	0	0	0	0	0	0	0	0	0	0	0	0	2	0	0	0	0	0
C75 .8	Malignant neoplasm, pluriglandular involvment, unspecified	0	0	0	0	0	0	0	0	0	0	0	0	2	0	0	0	0	0
C75 .9	Malignant neoplasm of endocrine gland, unspecified	0	0	0	0	0	0	0	0	0	0	0	0	2	0	0	0	0	0
C76	Malignant neoplasm of other and ill-defined sites	0	0	0	0	0	0	0	0	0	0	0	0	2	0	0	0	0	0
C76 .0	Malignant neoplasm of head, face & neck	0	0	0	0	0	0	0	0	0	0	0	0	2	0	0	0	0	0
C76 .1	Malignant neoplasm of thorax	0	0	0	0	0	0	0	0	0	0	0	0	2	0	0	0	0	0
C76 .2	Malignant neoplasm of abdomen	0	0	0	0	0	0	0	0	0	0	0	0	2	0	0	0	0	0
C76 .3	Malignant neoplasm of pelvis	0	0	0	0	0	0	0	0	0	0	0	0	2	0	0	0	0	0
C76 4	Malignant neoplasm of upper limb	0	0	0	0	0	0	0	0	0	0	0	0	2	0	0	0	0	0
C76 5	Malignant neoplasm of lower limb	0	0	0	0	0	0	0	0	0	0	0	0	2	0	0	0	0	0
C76 .7	Malignant neoplasm of other ill-defined sites	0	0	0	0	0	0	0	0	0	0	0	0	2	0	0	0	0	0
C76 .8	Malignant neoplasm, overlap lesion oth & ill-defined sites	0	0	0	0	0	0	0	0	0	0	0	0	2	0	0	0	0	0
C91	Lymphoid leukaemia	0	0	0	0	0	0	0	0	0	0	0	0	0	2	0	0	0	0
C91 .0	Acute lymphoblastic leukaemia	0	0	0	0	0	0	0	0	0	0	0	0	0	2	0	0	0	0
C91 .1	Chronic lymphocytic leukaemia	0	0	0	0	0	0	0	0	0	0	0	0	0	2	0	0	0	0
C91 .2	Subacute lymphocytic leukaemia	0	0	0	0	0	0	0	0	0	0	0	0	0	2	0	0	0	0
C91 .3	Prolymphocytic leukaemia	0	0	0	0	0	0	0	0	0	0	0	0	0	2	0	0	0	0
C91 .4	Hairy-cell leukaemia	0	0	0	0	0	0	0	0	0	0	0	0	0	2	0	0	0	0
C91 .5	Adult T-cell leukaemia	0	0	0	0	0	0	0	0	0	0	0	0	0	2	0	0	0	0
C91 .7	Other lymphoid leukaemia	0	0	0	0	0	0	0	0	0	0	0	0	0	2	0	0	0	0
C91 .9	Lymphoid leukaemia, unspecified	0	0	0	0	0	0	0	0	0	0	0	0	0	2	0	0	0	0
C92	Myeloid leukaemia	0	0	0	0	0	0	0	0	0	0	0	0	0	2	0	0	0	0
C92 .0	Acute myeloid leukaemia	0	0	0	0	0	0	0	0	0	0	0	0	0	2	0	0	0	0

C92.1	Chronic myeloid leukaemia	0	0	0	0	0	0	0	0	0	0	0	0	0	2	0	0	0	0
C92.2	Subacute myeloid leukaemia	0	0	0	0	0	0	0	0	0	0	0	0	0	2	0	0	0	0
C92.3	Myeloid sarcoma	0	0	0	0	0	0	0	0	0	0	0	0	0	2	0	0	0	0
C92.4	Acute promyelocytic leukaemia	0	0	0	0	0	0	0	0	0	0	0	0	0	2	0	0	0	0
C92.5	Acute myelomonocytic leukaemia	0	0	0	0	0	0	0	0	0	0	0	0	0	2	0	0	0	0
C92.7	Other myeloid leukaemia	0	0	0	0	0	0	0	0	0	0	0	0	0	2	0	0	0	0
C92.9	Myeloid leukaemia, unspecified	0	0	0	0	0	0	0	0	0	0	0	0	0	2	0	0	0	0
C93	Monocytic leukaemia	0	0	0	0	0	0	0	0	0	0	0	0	0	2	0	0	0	0
C93.0	Acute monocytic leukaemia	0	0	0	0	0	0	0	0	0	0	0	0	0	2	0	0	0	0
C93.1	Chronic monocytic leukaemia	0	0	0	0	0	0	0	0	0	0	0	0	0	2	0	0	0	0
C93.2	Subacute monocytic leukaemia	0	0	0	0	0	0	0	0	0	0	0	0	0	2	0	0	0	0
C93.7	Other monocytic leukaemia	0	0	0	0	0	0	0	0	0	0	0	0	0	2	0	0	0	0
C93.9	Monocytic leukaemia, unspecified	0	0	0	0	0	0	0	0	0	0	0	0	0	2	0	0	0	0
C94	Other leukaemias of specified cell type	0	0	0	0	0	0	0	0	0	0	0	0	0	2	0	0	0	0
C94.0	Acute erythraemia & erythroleukaemia	0	0	0	0	0	0	0	0	0	0	0	0	0	2	0	0	0	0
C94.1	Chronic erythraemia	0	0	0	0	0	0	0	0	0	0	0	0	0	2	0	0	0	0
C94.2	Acute megakaryoblastic leukaemia	0	0	0	0	0	0	0	0	0	0	0	0	0	2	0	0	0	0
C94.3	Mast cell leukaemia	0	0	0	0	0	0	0	0	0	0	0	0	0	2	0	0	0	0
C94.4	Acute panmyelosis	0	0	0	0	0	0	0	0	0	0	0	0	0	2	0	0	0	0
C94.5	Acute myelofibrosis	0	0	0	0	0	0	0	0	0	0	0	0	0	2	0	0	0	0
C94.7	Other specified leukaemias	0	0	0	0	0	0	0	0	0	0	0	0	0	2	0	0	0	0
C95	Leukaemia of unspecified cell type	0	0	0	0	0	0	0	0	0	0	0	0	0	2	0	0	0	0
C95.0	Acute leukaemia of unsp cell type	0	0	0	0	0	0	0	0	0	0	0	0	0	2	0	0	0	0
C95.1	Chronic leukaemia unsp cell type	0	0	0	0	0	0	0	0	0	0	0	0	0	2	0	0	0	0

C95.2	Subacute leukaemia unsp cell type	0	0	0	0	0	0	0	0	0	0	0	0	0	2	0	0	0	0
C95.7	Other leukaemia unspecified cell type	0	0	0	0	0	0	0	0	0	0	0	0	0	2	0	0	0	0
C95.9	Leukaemia, unspecified	0	0	0	0	0	0	0	0	0	0	0	0	0	2	0	0	0	0
C81	Hodgkin's disease	0	0	0	0	0	0	0	0	0	0	0	0	0	0	2	0	0	0
C81.0	Hodgkin's disease, lymphocytic predominance	0	0	0	0	0	0	0	0	0	0	0	0	0	0	2	0	0	0
C81.1	Hodgkin's disease, nodular sclerosis	0	0	0	0	0	0	0	0	0	0	0	0	0	0	2	0	0	0
C81.2	Hodgkin's disease, mixed cellularity	0	0	0	0	0	0	0	0	0	0	0	0	0	0	2	0	0	0
C81.3	Hodgkin's disease, lymphocytic depletion	0	0	0	0	0	0	0	0	0	0	0	0	0	0	2	0	0	0
C81.7	Hodgkin's disease, other Hodgkin's disease	0	0	0	0	0	0	0	0	0	0	0	0	0	0	2	0	0	0
C81.9	Hodgkin's disease, Hodgkin's disease, unspecified	0	0	0	0	0	0	0	0	0	0	0	0	0	0	2	0	0	0
C82	Follicular [nodular] non-Hodgkin's lymphoma	0	0	0	0	0	0	0	0	0	0	0	0	0	0	2	0	0	0
C82.0	Follicular non-Hodgkin's small cleaved cell lymphoma	0	0	0	0	0	0	0	0	0	0	0	0	0	0	2	0	0	0
C82.1	Follicular non-Hodg mixed sml cleavd & lge cell lymphoma	0	0	0	0	0	0	0	0	0	0	0	0	0	0	2	0	0	0
C82.2	Follicular non-Hodgkin's large cell lymphoma	0	0	0	0	0	0	0	0	0	0	0	0	0	0	2	0	0	0
C82.7	Follicular non-Hodgkin's other types of lymphoma	0	0	0	0	0	0	0	0	0	0	0	0	0	0	2	0	0	0
C82.9	Follicular non-Hodgkin's unspecified lymphoma	0	0	0	0	0	0	0	0	0	0	0	0	0	0	2	0	0	0
C83	Diffuse non-Hodgkin's lymphoma	0	0	0	0	0	0	0	0	0	0	0	0	0	0	2	0	0	0
C83.0	Diffuse non-Hodgkin's small cell (diffuse)lymphoma	0	0	0	0	0	0	0	0	0	0	0	0	0	0	2	0	0	0
C83.1	Diffuse non-Hodgkin's small cleaved cell (diffuse) lymphoma	0	0	0	0	0	0	0	0	0	0	0	0	0	0	2	0	0	0
C83.2	Diffuse non-Hodgkin mixed sml & lge cell (diffuse) lymphoma	0	0	0	0	0	0	0	0	0	0	0	0	0	0	2	0	0	0
C83.3	Diffuse non-Hodgkin's large cell (diffuse) lymphoma	0	0	0	0	0	0	0	0	0	0	0	0	0	0	2	0	0	0
C83.4	Diffuse non-Hodgkin's immunoblastic (diffuse) lymphoma	0	0	0	0	0	0	0	0	0	0	0	0	0	0	2	0	0	0
C83.5	Diffuse non-Hodgkin's lymphoblastic (diffuse) lymphoma	0	0	0	0	0	0	0	0	0	0	0	0	0	0	2	0	0	0
C83.6	Diffuse non-Hodgkin's lymphoma undifferentiated (diffuse)	0	0	0	0	0	0	0	0	0	0	0	0	0	0	2	0	0	0

C83 .7	Diffuse non-Hodgkin's lymphoma, Burkitt's tumour	0	0	0	0	0	0	0	0	0	0	0	0	0	0	2	0	0	0
C83 .8	Other types of diffuse non-Hodgkin's lymphoma	0	0	0	0	0	0	0	0	0	0	0	0	0	0	2	0	0	0
C83 .9	Diffuse non-Hodgkin's lymphoma, unspecified	0	0	0	0	0	0	0	0	0	0	0	0	0	0	2	0	0	0
C84	Peripheral and cutaneous T-cell lymphomas	0	0	0	0	0	0	0	0	0	0	0	0	0	0	2	0	0	0
C84 .0	Peripheral and cutaneous T-cell lymphomas, mycosis fungoides	0	0	0	0	0	0	0	0	0	0	0	0	0	0	2	0	0	0
C84 .1	Peripheral and cutaneous T-cell lymphomas, Sezary's disease	0	0	0	0	0	0	0	0	0	0	0	0	0	0	2	0	0	0
C84 .2	Peripheral and cutaneous T-cell lymphomas, T-zone lymphoma	0	0	0	0	0	0	0	0	0	0	0	0	0	0	2	0	0	0
C84 .3	Periph & cutan T-cell lymphomas, lymphoepithelioid lymphoma	0	0	0	0	0	0	0	0	0	0	0	0	0	0	2	0	0	0
C84 .4	Periph & cutan T-cell lymphomas, peripheral T-cell lymphoma	0	0	0	0	0	0	0	0	0	0	0	0	0	0	2	0	0	0
C84 .5	Periph & cutan T-cell lymphomas, oth & unsp T-cell lymphomas	0	0	0	0	0	0	0	0	0	0	0	0	0	0	2	0	0	0
C85	Other and unspecified types of non-Hodgkin's lymphoma	0	0	0	0	0	0	0	0	0	0	0	0	0	0	2	0	0	0
C85 .0	Oth & unspec types of non-Hodgkin's lymphoma, lymphosarcoma	0	0	0	0	0	0	0	0	0	0	0	0	0	0	2	0	0	0
C85 .1	Oth & unsp types non-Hodgkin's B-cell lymphoma, unsp	0	0	0	0	0	0	0	0	0	0	0	0	0	0	2	0	0	0
C85 .7	Oth specified types of non-Hodgkin's lymphoma	0	0	0	0	0	0	0	0	0	0	0	0	0	0	2	0	0	0
C85 .9	Non-Hodgkin's lymphoma, unspecified type	0	0	0	0	0	0	0	0	0	0	0	0	0	0	2	0	0	0
C88	Malignant immunoproliferative diseases	0	0	0	0	0	0	0	0	0	0	0	0	0	0	2	0	0	0
C88 .0	Waldenstrom's macroglobulinaemia	0	0	0	0	0	0	0	0	0	0	0	0	0	0	2	0	0	0
C88 .1	Alpha heavy chain disease	0	0	0	0	0	0	0	0	0	0	0	0	0	0	2	0	0	0
C88 .2	Gamma heavy chain disease	0	0	0	0	0	0	0	0	0	0	0	0	0	0	2	0	0	0
C88 .3	Malignant immunoproliferative small intestinal disease	0	0	0	0	0	0	0	0	0	0	0	0	0	0	2	0	0	0
C88 .7	Other malignant immunoproliferative diseases	0	0	0	0	0	0	0	0	0	0	0	0	0	0	2	0	0	0
C88 .9	Malignant immunoproliferative disease, unspecified	0	0	0	0	0	0	0	0	0	0	0	0	0	0	2	0	0	0
C96	Oth & unspec malig neop lymphoid, haematapoietic & rel tiss	0	0	0	0	0	0	0	0	0	0	0	0	0	0	2	0	0	0
C96 .0	Letterer-Siwe disease	0	0	0	0	0	0	0	0	0	0	0	0	0	0	2	0	0	0

C96.1	Malignant histiocytosis	0	0	0	0	0	0	0	0	0	0	0	0	0	0	2	0	0	0
C96.2	Malignant mast cell tumour	0	0	0	0	0	0	0	0	0	0	0	0	0	0	2	0	0	0
C96.3	True histiocyt lymphoma	0	0	0	0	0	0	0	0	0	0	0	0	0	0	2	0	0	0
C96.7	Oth spec malig neop lymphoid h'poietic & related tissue	0	0	0	0	0	0	0	0	0	0	0	0	0	0	2	0	0	0
C96.9	Malig neop lymphoid haematopoietic and related tissue unspec	0	0	0	0	0	0	0	0	0	0	0	0	0	0	2	0	0	0
C90	Multiple myeloma and malignant plasma cell neoplasms	0	0	0	0	0	0	0	0	0	0	0	0	0	0	2	0	0	0
C90.0	Multiple myeloma	0	0	0	0	0	0	0	0	0	0	0	0	0	0	2	0	0	0
C90.1	Plasma cell leukaemia	0	0	0	0	0	0	0	0	0	0	0	0	0	0	2	0	0	0
C90.2	Malignant plasma cell neoplasm, extramedullary plasmacytoma	0	0	0	0	0	0	0	0	0	0	0	0	0	0	2	0	0	0
B15.0	Hepatitis A with hepatic coma	0	0	0	0	0	0	0	0	0	0	0	0	0	0	0	3	0	0
B16.0	Acute hep B with delta-agent (coinfection) with hep coma	0	0	0	0	0	0	0	0	0	0	0	0	0	0	0	3	0	0
B16.2	Acute hepatitis B without delta-agent with hepatic coma	0	0	0	0	0	0	0	0	0	0	0	0	0	0	0	3	0	0
B19.0	Unspecified viral hepatitis hepatic with coma	0	0	0	0	0	0	0	0	0	0	0	0	0	0	0	3	0	0
K70.4	Alcoholic hepatic failure	0	0	0	0	0	0	0	0	0	0	0	0	0	0	0	3	0	0
K72	Hepatic failure, not elsewhere classified	0	0	0	0	0	0	0	0	0	0	0	0	0	0	0	3	0	0
K76.6	Portal hypertension	0	0	0	0	0	0	0	0	0	0	0	0	0	0	0	3	0	0
K76.7	Hepatorenal syndrome	0	0	0	0	0	0	0	0	0	0	0	0	0	0	0	3	0	0
I85	Oesophageal varices	0	0	0	0	0	0	0	0	0	0	0	0	0	0	0	3	0	0
I85.0	Oesophageal varices with bleeding	0	0	0	0	0	0	0	0	0	0	0	0	0	0	0	3	0	0
I85.9	Oesophageal varices without bleeding	0	0	0	0	0	0	0	0	0	0	0	0	0	0	0	3	0	0
C77	Secondary and unspecified malignant neoplasm of lymph nodes	0	0	0	0	0	0	0	0	0	0	0	0	0	0	0	0	6	0
C77.0	Sec & uns malig neoplasm of lymph nodes of head, face & neck	0	0	0	0	0	0	0	0	0	0	0	0	0	0	0	0	6	0
C77.1	Sec & uns malignant neoplasm of intrathoracic lymph nodes	0	0	0	0	0	0	0	0	0	0	0	0	0	0	0	0	6	0
C77.2	Sec & uns malignant neoplasm of intra-abdominal lymph nodes	0	0	0	0	0	0	0	0	0	0	0	0	0	0	0	0	6	0

C77.3	Sec & uns malig neoplasm of axillary & upp limb lymph nodes	0	0	0	0	0	0	0	0	0	0	0	0	0	0	0	0	6	0
C77.4	Sec & uns malig neoplasm of inguinal & low limb lymph nodes	0	0	0	0	0	0	0	0	0	0	0	0	0	0	0	0	6	0
C77.5	Sec & uns malignant neoplasm of intrapelvic lymph nodes	0	0	0	0	0	0	0	0	0	0	0	0	0	0	0	0	6	0
C77.8	Sec & uns malig neoplasm of lymph nodes of multiple regions	0	0	0	0	0	0	0	0	0	0	0	0	0	0	0	0	6	0
C77.9	Sec & uns malignant neoplasm of lymph node, unspecified	0	0	0	0	0	0	0	0	0	0	0	0	0	0	0	0	6	0
C78	Sec malignant neoplasm of respiratory and digestive organs	0	0	0	0	0	0	0	0	0	0	0	0	0	0	0	0	6	0
C78.0	Secondary malignant neoplasm of lung	0	0	0	0	0	0	0	0	0	0	0	0	0	0	0	0	6	0
C78.1	Secondary malignant neoplasm of mediastinum	0	0	0	0	0	0	0	0	0	0	0	0	0	0	0	0	6	0
C78.2	Secondary malignant neoplasm of pleura	0	0	0	0	0	0	0	0	0	0	0	0	0	0	0	0	6	0
C78.3	Secondary malignant neoplasm of oth & unsp respiratory orgs	0	0	0	0	0	0	0	0	0	0	0	0	0	0	0	0	6	0
C78.4	Secondary malignant neoplasm of small intestine	0	0	0	0	0	0	0	0	0	0	0	0	0	0	0	0	6	0
C78.5	Secondary malignant neoplasm of large intest & rectum	0	0	0	0	0	0	0	0	0	0	0	0	0	0	0	0	6	0
C78.6	Secondary malignant neoplasm of retroperitoneum & peritoneum	0	0	0	0	0	0	0	0	0	0	0	0	0	0	0	0	6	0
C78.7	Secondary malignant neoplasm of liver	0	0	0	0	0	0	0	0	0	0	0	0	0	0	0	0	6	0
C78.8	Secondary malignant neoplasm of other & unsp digestive orgs	0	0	0	0	0	0	0	0	0	0	0	0	0	0	0	0	6	0
C79	Secondary malignant neoplasm of other sites	0	0	0	0	0	0	0	0	0	0	0	0	0	0	0	0	6	0
C79.0	Secondary malignant neoplasm of kidney & renal pelvis	0	0	0	0	0	0	0	0	0	0	0	0	0	0	0	0	6	0
C79.1	Secondary malignant neoplasm of oth & uns urinary organs	0	0	0	0	0	0	0	0	0	0	0	0	0	0	0	0	6	0
C79.2	Secondary malignant neoplasm of skin	0	0	0	0	0	0	0	0	0	0	0	0	0	0	0	0	6	0
C79.3	Secondary malignant neoplasm of brain & cerebral meninges	0	0	0	0	0	0	0	0	0	0	0	0	0	0	0	0	6	0
C79.4	Secondary malignant neoplasm of oth & unsp parts nervous sys	0	0	0	0	0	0	0	0	0	0	0	0	0	0	0	0	6	0
C79.5	Secondary malignant neoplasm of bone and bone marrow	0	0	0	0	0	0	0	0	0	0	0	0	0	0	0	0	6	0
C79.6	Secondary malignant neoplasm of ovary	0	0	0	0	0	0	0	0	0	0	0	0	0	0	0	0	6	0
C79.7	Secondary malignant neoplasm of adrenal gland	0	0	0	0	0	0	0	0	0	0	0	0	0	0	0	0	6	0

C79.8	Secondary malignant neoplasm of other specified sites	0	0	0	0	0	0	0	0	0	0	0	0	0	0	0	0	6	0
C80	Malignant neoplasm without specification of site	0	0	0	0	0	0	0	0	0	0	0	0	0	0	0	0	6	0
B20	Human immunodef virus dis result infectious parasitic dis	0	0	0	0	0	0	0	0	0	0	0	0	0	0	0	0	0	6
B20.0	HIV disease resulting in mycobacterial infection	0	0	0	0	0	0	0	0	0	0	0	0	0	0	0	0	0	6
B20.1	HIV disease resulting in other bacterial infections	0	0	0	0	0	0	0	0	0	0	0	0	0	0	0	0	0	6
B20.2	HIV disease resulting in cytomegaloviral disease	0	0	0	0	0	0	0	0	0	0	0	0	0	0	0	0	0	6
B20.3	HIV disease resulting in other viral infections	0	0	0	0	0	0	0	0	0	0	0	0	0	0	0	0	0	6
B20.4	HIV disease resulting in candidiasis	0	0	0	0	0	0	0	0	0	0	0	0	0	0	0	0	0	6
B20.5	HIV disease resulting in other mycoses	0	0	0	0	0	0	0	0	0	0	0	0	0	0	0	0	0	6
B20.6	HIV disease resulting in Pneumocystis carinii pneumonia	0	0	0	0	0	0	0	0	0	0	0	0	0	0	0	0	0	6
B20.7	HIV disease resulting in multiple infections	0	0	0	0	0	0	0	0	0	0	0	0	0	0	0	0	0	6
B20.8	HIV dis resulting in oth infectious and parasitic dis	0	0	0	0	0	0	0	0	0	0	0	0	0	0	0	0	0	6
B20.9	HIV disease resulting in unspec infectious or parasitic dis	0	0	0	0	0	0	0	0	0	0	0	0	0	0	0	0	0	6
B21	Human immunodef virus dis resulting malignant neopl	0	0	0	0	0	0	0	0	0	0	0	0	0	0	0	0	0	6
B21.0	HIV disease resulting in Kaposi's sarcoma	0	0	0	0	0	0	0	0	0	0	0	0	0	0	0	0	0	6
B21.1	HIV disease resulting in Burkitt's lymphoma	0	0	0	0	0	0	0	0	0	0	0	0	0	0	0	0	0	6
B21.2	HIV dis resulting oth types of non-Hodgkin's lymphoma	0	0	0	0	0	0	0	0	0	0	0	0	0	0	0	0	0	6
B21.3	HIV dis result oth mal neo lymphoid haematopoietic rel tis	0	0	0	0	0	0	0	0	0	0	0	0	0	0	0	0	0	6
B21.7	HIV disease resulting in multiple malignant neoplasms	0	0	0	0	0	0	0	0	0	0	0	0	0	0	0	0	0	6
B21.8	HIV disease resulting in other malignant neoplasms	0	0	0	0	0	0	0	0	0	0	0	0	0	0	0	0	0	6
B21.9	HIV disease resulting in unspecified malignant neoplasm	0	0	0	0	0	0	0	0	0	0	0	0	0	0	0	0	0	6
B22	Human immunodef virus dis resulting in other spec dis	0	0	0	0	0	0	0	0	0	0	0	0	0	0	0	0	0	6
B22.0	HIV disease resulting in encephalopathy	0	0	0	0	0	0	0	0	0	0	0	0	0	0	0	0	0	6
B22.1	HIV disease resulting in lymphoid interstitial pneumonitis	0	0	0	0	0	0	0	0	0	0	0	0	0	0	0	0	0	6

B22.2	HIV disease resulting in wasting syndrome	0	0	0	0	0	0	0	0	0	0	0	0	0	0	0	0	0	6
B22.7	HIV dis resulting in multiple diseases classif elsewhere	0	0	0	0	0	0	0	0	0	0	0	0	0	0	0	0	0	6
B23	Human immunodef virus dis resulting in other conditions	0	0	0	0	0	0	0	0	0	0	0	0	0	0	0	0	0	6
B23.0	Acute HIV infection syndrome	0	0	0	0	0	0	0	0	0	0	0	0	0	0	0	0	0	6
B23.1	HIV dis result (persistent) generalized lymphadenopathy	0	0	0	0	0	0	0	0	0	0	0	0	0	0	0	0	0	6
B23.2	HIV dis result haematologic / immunologic abnorm NEC	0	0	0	0	0	0	0	0	0	0	0	0	0	0	0	0	0	6
B23.8	HIV disease resulting in other specified conditions	0	0	0	0	0	0	0	0	0	0	0	0	0	0	0	0	0	6
B24	Unspecified human immunodefiency virus [HIV] disease	0	0	0	0	0	0	0	0	0	0	0	0	0	0	0	0	0	6
F02.4	Dementia in human immunodef virus [HIV] disease	0	0	0	0	0	0	0	0	0	0	0	0	0	0	0	0	0	6
Z21	Asymptomatic human immunodef virus [HIV] infect status	0	0	0	0	0	0	0	0	0	0	0	0	0	0	0	0	0	6

opcs	description	cci_1pad	cci_2renal	cci_3liver
L222	Revision of prosthesis of bifurcation of aorta	1	0	0
L223	Revision of prosthesis of abdominal aorta NEC	1	0	0
L224	Removal of prosthesis from aorta	1	0	0
L235	Revision of plastic repair of aorta	1	0	0
L16	Extra-anatomic bypass of aorta	1	0	0
L168	Other specified extra-anatomic bypass of aorta	1	0	0
L169	Unspecified extra-anatomic bypass of aorta	1	0	0
L208	Other specified other emergency bypass of segment of aorta	1	0	0
L209	Unspecified other emergency bypass of segment of aorta	1	0	0
L21	Other bypass of segment of aorta	1	0	0
L213	Bypass of segment of suprarenal abdominal aorta by anastomosis of aorta to aorta NEC	1	0	0
L214	Bypass of segment of infrarenal abdominal aorta by anastomosis of aorta to aorta NEC	1	0	0
L215	Bypass of segment of abdominal aorta by anastomosis of aorta to aorta NEC	1	0	0
L218	Other specified other bypass of segment of aorta	1	0	0
L219	Unspecified other bypass of segment of aorta	1	0	0

L23	Plastic repair of aorta	1	0	0
L231	Plastic repair of aorta and end to end anastomosis of aorta	1	0	0
L232	Plastic repair of aorta using subclavian flap	1	0	0
L233	Plastic repair of aorta using patch graft	1	0	0
L236	Plastic repair of aorta and insertion of tube graft	1	0	0
L25	Other open operations on aorta	1	0	0
L251	Endarterectomy of aorta and patch repair of aorta	1	0	0
L252	Endarterectomy of aorta NEC	1	0	0
L258	Other specified other open operations on aorta	1	0	0
L259	Unspecified other open operations on aorta	1	0	0
L26	Transluminal operations on aorta	1	0	0
L261	Percutaneous transluminal balloon angioplasty of aorta	1	0	0
L262	Percutaneous transluminal angioplasty of aorta NEC	1	0	0
L263	Percutaneous transluminal embolectomy of bifurcation of aorta	1	0	0
L265	Percutaneous transluminal insertion of stent into aorta	1	0	0
L266	Transluminal aortic stent graft with fenestration NEC	1	0	0
L267	Transluminal aortic branched stent graft NEC	1	0	0
L268	Other specified transluminal operations on aorta	1	0	0
L269	Unspecified transluminal operations on aorta	1	0	0
L288	Other specified transluminal operations on aneurysmal segment of aorta	1	0	0
L289	Unspecified transluminal operations on aneurysmal segment of aorta	1	0	0
L18	Emergency replacement of aneurysmal segment of aorta	1	0	0
L183	Emergency replacement of aneurysmal segment of suprarenal abdominal aorta by anastomosis of aorta to aorta	1	0	0
L184	Emergency replacement of aneurysmal segment of infrarenal abdominal aorta by anastomosis of aorta to aorta	1	0	0
L185	Emergency replacement of aneurysmal segment of abdominal aorta by anastomosis of aorta to aorta NEC	1	0	0
L186	Emergency replacement of aneurysmal bifurcation of aorta by anastomosis of aorta to iliac artery	1	0	0
L188	Other specified emergency replacement of aneurysmal segment of aorta	1	0	0
L189	Unspecified emergency replacement of aneurysmal segment of aorta	1	0	0
L20	Other emergency bypass of segment of aorta	1	0	0
L203	Emergency bypass of segment of suprarenal abdominal aorta by anastomosis of aorta to aorta NEC	1	0	0
L204	Emergency bypass of segment of infrarenal abdominal aorta by anastomosis of aorta to aorta NEC	1	0	0

L205	Emergency bypass of segment of abdominal aorta by anastomosis of aorta to aorta NEC	1	0	0
L206	Emergency bypass of bifurcation of aorta by anastomosis of aorta to iliac artery NEC	1	0	0
L19	Other replacement of aneurysmal segment of aorta	1	0	0
L193	Replacement of aneurysmal segment of suprarenal abdominal aorta by anastomosis of aorta to aorta NEC	1	0	0
L194	Replacement of aneurysmal segment of infrarenal abdominal aorta by anastomosis of aorta to aorta NEC	1	0	0
L195	Replacement of aneurysmal segment of abdominal aorta by anastomosis of aorta to aorta NEC	1	0	0
L196	Replacement of aneurysmal bifurcation of aorta by anastomosis of aorta to iliac artery NEC	1	0	0
L198	Other specified other replacement of aneurysmal segment of aorta	1	0	0
L199	Unspecified other replacement of aneurysmal segment of aorta	1	0	0
L254	Operations on aneurysm of aorta NEC	1	0	0
L27	Transluminal insertion of stent graft for aneurysmal segment of aorta	1	0	0
L271	Endovascular insertion of stent graft for infrarenal abdominal aortic aneurysm	1	0	0
L272	Endovascular insertion of stent graft for suprarenal aortic aneurysm	1	0	0
L275	Endovascular insertion of stent graft for aortic aneurysm of bifurcation NEC	1	0	0
L276	Endovascular insertion of stent graft for aorto-uniiliac aneurysm	1	0	0
L278	Other specified transluminal insertion of stent graft for aneurysmal segment of aorta	1	0	0
L279	Unspecified transluminal insertion of stent graft for aneurysmal segment of aorta	1	0	0
L28	Transluminal operations on aneurysmal segment of aorta	1	0	0
L281	Endovascular insertion of stent for infrarenal abdominal aortic aneurysm	1	0	0
L282	Endovascular insertion of stent for suprarenal aortic aneurysm	1	0	0
L285	Endovascular insertion of stent for aortic aneurysm of bifurcation NEC	1	0	0
L286	Endovascular insertion of stent for aorto-uniiliac aneurysm	1	0	0
L50	Other emergency bypass of iliac artery	1	0	0
L501	Emergency bypass of common iliac artery by anastomosis of aorta to common iliac artery NEC	1	0	0
L502	Emergency bypass of iliac artery by anastomosis of aorta to external iliac artery NEC	1	0	0
L503	Emergency bypass of artery of leg by anastomosis of aorta to common femoral artery NEC	1	0	0
L504	Emergency bypass of artery of leg by anastomosis of aorta to deep femoral artery NEC	1	0	0
L505	Emergency bypass of iliac artery by anastomosis of iliac artery to iliac artery NEC	1	0	0
L506	Emergency bypass of artery of leg by anastomosis of iliac artery to femoral artery NEC	1	0	0
L508	Other specified other emergency bypass of iliac artery	1	0	0
L509	Unspecified other emergency bypass of iliac artery	1	0	0

L51	Other bypass of iliac artery	1	0	0
L511	Bypass of common iliac artery by anastomosis of aorta to common iliac artery NEC	1	0	0
L512	Bypass of iliac artery by anastomosis of aorta to external iliac artery NEC	1	0	0
L513	Bypass of artery of leg by anastomosis of aorta to common femoral artery NEC	1	0	0
L514	Bypass of artery of leg by anastomosis of aorta to deep femoral artery NEC	1	0	0
L515	Bypass of iliac artery by anastomosis of iliac artery to iliac artery NEC	1	0	0
L516	Bypass of artery of leg by anastomosis of iliac artery to femoral artery NEC	1	0	0
L518	Other specified other bypass of iliac artery	1	0	0
L519	Unspecified other bypass of iliac artery	1	0	0
L52	Reconstruction of iliac artery	1	0	0
L521	Endarterectomy of iliac artery and patch repair of iliac artery	1	0	0
L522	Endarterectomy of iliac artery NEC	1	0	0
L528	Other specified reconstruction of iliac artery	1	0	0
L529	Unspecified reconstruction of iliac artery	1	0	0
L531	Repair of iliac artery NEC	1	0	0
L532	Open embolectomy of iliac artery	1	0	0
L538	Other specified other open operations on iliac artery	1	0	0
L539	Unspecified other open operations on iliac artery	1	0	0
L54	Transluminal operations on iliac artery	1	0	0
L541	Percutaneous transluminal angioplasty of iliac artery	1	0	0
L542	Percutaneous transluminal embolectomy of iliac artery	1	0	0
L544	Percutaneous transluminal insertion of stent into iliac artery	1	0	0
L548	Other specified transluminal operations on iliac artery	1	0	0
L549	Unspecified transluminal operations on iliac artery	1	0	0
L58	Other emergency bypass of femoral artery	1	0	0
L581	Emergency bypass of femoral artery by anastomosis of femoral artery to femoral artery NEC	1	0	0
L582	Emergency bypass of femoral artery by anastomosis of femoral artery to popliteal artery using prosthesis NEC	1	0	0
L583	Emergency bypass of femoral artery by anastomosis of femoral artery to popliteal artery using vein graft NEC	1	0	0
L584	Emergency bypass of femoral artery by anastomosis of femoral artery to tibial artery using prosthesis NEC	1	0	0
L585	Emergency bypass of femoral artery by anastomosis of femoral artery to tibial artery using vein graft NEC	1	0	0
L586	Emergency bypass of femoral artery by anastomosis of femoral artery to peroneal artery using prosthesis NEC	1	0	0

L587	Emergency bypass of femoral artery by anastomosis of femoral artery to peroneal artery using vein graft NEC	1	0	0
L588	Other specified other emergency bypass of femoral artery	1	0	0
L589	Unspecified other emergency bypass of femoral artery	1	0	0
L59	Other bypass of femoral artery	1	0	0
L591	Bypass of femoral artery by anastomosis of femoral artery to femoral artery NEC	1	0	0
L592	Bypass of femoral artery by anastomosis of femoral artery to popliteal artery using prosthesis NEC	1	0	0
L593	Bypass of femoral artery by anastomosis of femoral artery to popliteal artery using vein graft NEC	1	0	0
L594	Bypass of femoral artery by anastomosis of femoral artery to tibial artery using prosthesis NEC	1	0	0
L595	Bypass of femoral artery by anastomosis of femoral artery to tibial artery using vein graft NEC	1	0	0
L596	Bypass of femoral artery by anastomosis of femoral artery to peroneal artery using prosthesis NEC	1	0	0
L597	Bypass of femoral artery by anastomosis of femoral artery to peroneal artery using vein graft NEC	1	0	0
L598	Other specified other bypass of femoral artery	1	0	0
L599	Unspecified other bypass of femoral artery	1	0	0
L60	Reconstruction of femoral artery	1	0	0
L601	Endarterectomy of femoral artery and patch repair of femoral artery	1	0	0
L602	Endarterectomy of femoral artery NEC	1	0	0
L603	Profundoplasty of femoral artery and patch repair of deep femoral artery	1	0	0
L604	Profundoplasty of femoral artery NEC	1	0	0
L608	Other specified reconstruction of femoral artery	1	0	0
L609	Unspecified reconstruction of femoral artery	1	0	0
L621	Repair of femoral artery NEC	1	0	0
L622	Open embolectomy of femoral artery	1	0	0
L628	Other specified other open operations on femoral artery	1	0	0
L629	Unspecified other open operations on femoral artery	1	0	0
L631	Percutaneous transluminal angioplasty of femoral artery	1	0	0
L632	Percutaneous transluminal embolectomy of femoral artery	1	0	0
L633	Percutaneous transluminal embolisation of femoral artery	1	0	0
L635	Percutaneous transluminal insertion of stent into femoral artery	1	0	0
L638	Other specified transluminal operations on femoral artery	1	0	0
L639	Unspecified transluminal operations on femoral artery	1	0	0
L651	Revision of reconstruction involving aorta	1	0	0

L652	Revision of reconstruction involving iliac artery	1	0	0
L653	Revision of reconstruction involving femoral artery	1	0	0
L742	creation of arteriovenous fistula nec	0	2	0
L746	creation of graft fistula for dialysis	0	2	0
M01	Transplantation of kidney	0	2	0
M011	Autotransplantation of kidney	0	2	0
M012	Allotransplantation of kidney from live donor	0	2	0
M013	Allotransplantation of kidney from cadaver NEC	0	2	0
M014	Allotransplantation of kidney from cadaver heart beating	0	2	0
M015	Allotransplantation of kidney from cadaver heart non-beating	0	2	0
M018	Other specified transplantation of kidney	0	2	0
M019	Unspecified transplantation of kidney	0	2	0
M026	Excision of rejected transplanted kidney	0	2	0
M027	excision of transplanted kidney nec	0	2	0
M084	Exploration of transplanted kidney	0	2	0
M172	Pre-transplantation of kidney work-up - recipient	0	2	0
M174	Post-transplantation of kidney examination - recipient	0	2	0
X401	Renal dialysis	0	2	0
X402	Peritoneal dialysis NEC	0	2	0
X403	Haemodialysis NEC	0	2	0
X404	Haemofiltration	0	2	0
X405	Automated peritoneal dialysis	0	2	0
X406	Continuous ambulatory peritoneal dialysis	0	2	0
X41	Placement of ambulatory apparatus for compensation for renal failure	0	2	0
X411	Insertion of ambulatory peritoneal dialysis catheter	0	2	0
X412	Removal of ambulatory peritoneal dialysis catheter	0	2	0
X421	Insertion of temporary peritoneal dialysis catheter	0	2	0
G10	Open operations on varices of oesophagus	0	0	3
G101	Disconnection of azygos vein	0	0	3
G102	Transection of oesophagus using staple gun	0	0	3
G103	Transection of oesophagus NEC	0	0	3

G104	Local ligation of varices of oesophagus	0	0	3
G105	Open injection sclerotherapy to varices of oesophagus	0	0	3
G108	Other specified open operations on varices of oesophagus	0	0	3
G109	Unspecified open operations on varices of oesophagus	0	0	3
G144	Fibreoptic endoscopic injection sclerotherapy to varices of oesophagus	0	0	3
G174	Endoscopic injection sclerotherapy to varices of oesophagus using rigid oesophagoscope	0	0	3
G437	Fibreoptic endoscopic rubber band ligation of upper gastrointestinal tract varices	0	0	3
J111	Transjugular intrahepatic angioplasty of portal vein	0	0	3
J114	Transjugular intrahepatic creation of portosystemic shunt	0	0	3
J118	Other specified transjugular intrahepatic operations on blood vessel of liver	0	0	3
J119	Unspecified transjugular intrahepatic operations on blood vessel of liver	0	0	3
