# Supplementary material for: Partner Bereavement and Risk of Herpes Zoster: Results from Two Population-Based Case-Control Studies in Denmark and the United Kingdom
Source: Clin Infect Dis. 2016 Dec 15;64(5):572–9. doi: 10.1093/cid/ciw840 (PMC5850543; doi:10.1093/cid/ciw840)
Supplement: Supplementary_Appendix_4 [file ciw840_suppl_supplementary_appendix_4.pdf]

Protocol

Partner bereavement and risk of herpes zoster: a nationwide Danish case-control study

Authors: Sigrun Alba Johannesdottir Schmidt,[1] Henrik Søndergaard Pedersen,[2] Morten Fenger-Grøn,[2] Henrik Carl Schønheyder,[3] Sinéad Langan,[4] Henrik Toft Sørensen,[1] Mogens Vestergaard[2]

Sigrun A J Schmidt,[1] Mogens Vestergaard,[2] Henrik S Pedersen,[2] Henrik C Schønheyder,[3] Sara L Thomas,[4] Liam Smeeth,[4] Kathryn E Mansfield,[4] Henrik T Sørensen,[1] Harriet J Forbes,[4] Sinéad M Langan[4]

Affiliations:

[1]Department of Clinical Epidemiology, Aarhus University Hospital, Aarhus, Denmark

[2]Research Unit and Section for General Practice, Department of Public Health, Aarhus University, Denmark

[3]Department of Clinical Microbiology, Aalborg University Hospital, Aalborg, Denmark

[4]Faculty of Epidemiology and Population Health, London School of Hygiene and Tropical Medicine, United Kingdom

Protocol

Amendments

October 31, 2016: We have changed the prescription-based definition to include the zoster-specific tablet doses only (acyclovir in 800 mg tablet dose in packages with 35 pills or a 500 mg tablet dose of valacyclovir or famciclovir) in the main analysis (instead of as a sensitivity analysis). This change is based on preliminary data from an ongoing Danish validation study, which suggests that the algorithm including only the zoster-specific tablet doses is more specific than the broader definition including all tablet doses.

Protocol

BACKGROUND

Herpes zoster (HZ) is a frequent neurocutaneous disease caused by reactivation of varicella zoster virus (VZV), which lies latent in the sensory ganglia after the primary infection chickenpox.[1,2] Reactivation occurs when the cell-mediated immunity towards VZV wanes below a certain critical level. Accordingly, the risk of HZ is increased among elderly because of immunosenescence, among patients with inherited cell-mediated immunodeficiency, and among patients with immunosuppression acquired by drugs (*e.g.*, patients receiving corticosteroids or chemotherapy), radiotherapy, viral infections (*i.e.*, human immunodeficiency virus [HIV]), or malignancy.[2,3]

Psychological stress is associated with activation of the hypothalamic-pituitary-adrenal axis and thereby suppression of the immune system,[4-6] which could in turn increase the risk of HZ. For example, it has been demonstrated that VZV-specific cell-mediated immunity is reduced in patients with major depression[7] and correlates negatively with severity of depressive symptoms.[8] Furthermore, the immunological response to the zoster vaccine is reduced in patients with untreated depression compared with individuals treated with antidepressants and individuals without depression.[9] Data on the association between psychological stressors other than psychiatric diagnoses and HZ are lacking.

Bereavement of a spouse or partner is considered one of the most stressful life events.[10] However, only four studies have examined spousal bereavement as a risk factor for HZ, showing conflicting results.[11-14] Comparison of the studies is hampered by study limitations and differences in methods used. First, previous studies may have been affected volunteer bias.[12-14] Second, various exposure windows and definitions have been employed by grouping spousal death together with other catastrophic health events in a spouse,[11] other spousal events (divorce, separation or marriage),[13] or other negative life events (*e.g.* health troubles or becoming unemployed).[12,15] Third, exposure definitions may have been prone to misclassification of spousal death due to recall

bias,[12,13,15] lack of interviewer blinding,[12,13] or use of claims data to identify spousal events.[11] Finally, studies have been limited by small study size[11-13,15] and only one study performed detailed analysis according to timing of the life event.[11]

OBJECTIVE

The objective of this study is to examine whether experiencing the death of a partner is associated with an increased risk of herpes zoster. Specifically, we will examine whether the association varies with time since bereavement, underlying immunosuppression, and diagnosis of depression/anxiety.

MATERIAL AND METHODS

Setting and design

We will conduct this study in Denmark, which has a population of approximately 5.6 million inhabitants. The Danish welfare system guarantees unfettered access to hospitals and general practitioners, as well as reimbursement of medication to various degrees.[16] Healthcare services provided are registered in various databases using the unique central personal register (CPR) number assigned to all residents at birth or upon immigration.[16] This study will be designed as a density case-control study using such databases. The study period will commence on January 1, 1997 with continuation until December 31, 2013, which ensures at least two years of prescription history for all study participants. All registry codes for the study are provided in the Supplementary Table S1.

Data sources

The Danish National Patient Registry (DNPR) records data for the Danish hospital sector, including data on all admissions to non-psychiatric hospitals with nationwide coverage since 1978,

admissions to psychiatric wards since 1994, and visits to all outpatient hospital-based specialty clinics and emergency rooms since 1994.[17] Each registry record includes data pertaining to the patient (*e.g.* CPR number), dates of admission and discharge or start and end of outpatient follow-up, procedures, the primary diagnosis, and one or more secondary diagnoses.[17] Diagnoses are classified according to the *International Classification of Diseases*, 8th revision (ICD-8) until the end of 1993 and the 10th revision (ICD-10) thereafter. Surgical procedures are coded according to a Danish classification (1977 through 1995) and a Danish version of the Nordic Medico-Statistical Committee (NOMESCO) Classification of Surgical Procedures (from 1996 on). General practitioners and practicing specialists do not report to the DNPR.

The Danish Psychiatric Central Research Register (PCRR) collects data on all admissions to psychiatric departments since 1970 and on outpatient treatment and emergency room contacts since 1995.[18] The registry structure resembles that described for the DNPR, providing data on relevant dates and diagnosis codes for each hospital contact.[18]

The Danish National Prescription Registry provides information on computerized prescription drugs since 1995.[19] Each time a prescription is redeemed at the pharmacy, the patient's CPR number, the date, and the type and quantity of drug redeemed is recorded in electronically for the database.[19] The type of drug is registered according to World Health Organization's *Anatomical Therapeutic Chemical Classification System*. Indication codes for treatment are available since 2004, but are used inconsistently (73% complete in 2012) and can be too unspecific for use in research.

The Danish National Diabetes Register includes data on diabetic patients since 1995.[20] The registry population is identified using an algorithm that combines diagnostic data from the DNPR, blood-glucose measurements from the National Health Service Register, prescriptions for anti-

diabetics according to the Danish National Prescription Registry, and civic data from the Civil Registration System.[20]

The Civil Registration System data on age, sex, address, and vital statistics for the entire population since 1968.[16] The registry also contains data on civil status (single, married, divorced, widow or widower, registered partnership, or dissolved registered partnership), CPR number of spouse or registered partner, and CPR numbers of children. As of 1989, information in the Civil Registration System is updated daily.

Linkage of all registries will be performed on the secure servers at Statistics Denmark, the central authority on Danish statistics, which collects, processes, and publishes information relating to the Danish society. Statistics Denmark also collects data from Danish education registers, such as the Population Education Register,[21] which was included in the present study.

Herpes zoster

Cases will be defined through two sources:

1) DNPR: all patients with a first-time hospital diagnosis (inpatient, outpatient, emergency room) of HZ during 1997 through 2013. For inclusion of cases with hospital diagnosis of HZ (see Table below for codes), we will only consider those with a first-listed (primary=A) diagnosis of HZ (to avoid including long-term complications with inaccurate diagnosis date of primary HZ). The date of admission or start of outpatient follow-up will be considered the diagnosis date. We will restrict to patients aged 18 years or older because of differences in the epidemiology of HZ in children and adults.
2) The Prescription Registry: all individuals aged ≥40 years with a first-time prescription record for acyclovir, valacyclovir or famciclovir during 1997 through 2013.

**Codes used to identify case population (all sub-codes included unless otherwise stated)**	
Herpes zoster	
Hospital diagnosis (DNPR)	ICD-8: 053; ICD-10: B02, H03.1F, H13.1M, H19.0D, H19.2D, H19.2J, H22.0C, H62.1B, G05.1I, G05.1M
Treatment in general practice (Prescription Registry)	
Acyclovir	ATC: J05AB01 (HZ-specific dose: 800 mg in packages of 35 tablets)
Valacyclovir	ATC: J05AB11 (HZ-specific dose: 500 mg)
Famciclovir	ATC: J05AB09 (HZ-specific dose: 500 mg)
Postherpatic neuralgia (PHN)	ICD-10: G530 or B022 (if specifically coded, if B02 is coded without further specification it is not defined as PHN)

We will only consider patients with no previous codes for HZ or PHN in the DNPR (ICD codes in any position (inpatient, outpatient, emergency room, primary and secondary diagnoses) or the Prescription Registry (ATC codes). Thus, if the earliest mention of HZ in the data is a secondary 053, B02 or H03.1F, H13.1M, H19.0D, H19.2D, H19.2J, H22.0C, H62.1B, G05.1I, G05.1M code in DNPR or a primary or secondary PHN code, we will exclude the patient from the case population and the person will no longer be at risk of HZ (that is, not eligible as a control either).

The date of diagnosis (DNPR) or prescription (Prescription Registry), whichever comes first, will be considered the index date for HZ cases.

Population Controls

We will use the Civil Registration System to individually match up to 4 population controls to each HZ case by age (±2 months) and sex. We will sample controls using risk-set sampling; that is, only individuals alive and with no previous primary or secondary diagnosis of HZ or PHN (ICD-8: 053; ICD-10: B02, G530) and no previous HZ related prescription (ATC: J05AB01, J05AB11, J05AB09) ever before the index date of the case will be eligible for inclusion. Controls will be assigned an index date identical to that of their case.

Partner bereavement

We will use the Civil Registration System to retrieve information on marital status, corresponding partner, and all partner deaths for cases and controls prior to the index date. Partners will be identified using an alogirthm developed by Statistics Denmark. From the DNPR, we will ascertain the entire hospital diagnosis history for deceased partners at one month before their date of death and compute their age-adjusted Charlson Comorbidity Index (ACCI)[22] as a proxy measure of whether the death of the partner was unforeseen. The original Charlson Comorbidity Index assigns 0 to 6 points to a range of chronic diseases according to their ability to predict death.[22] In the age-adjusted index points are also given according to age (1 point: 50-59 years; 2 point: 60-69 years; 3 points: 70-79 years; 4 points: 80-89 years; 5 points: 90-99 years).[22] The codes used for the index are shown below. Based on the total ACCI score, we will the group study participants into three categories of risk of partner death: lowest risk (0–3 points), intermediate risk (4–6 points), or highest risk (≥7 points).

The age-adjusted Charlson Comorbidity Index

Score 1	
Myocardial infarction	ICD-8: 410; ICD-10: I21, I22, I23
Congestive heart failure	ICD-8: 427.09, 427.10, 427.11, 427.19, 428.99, 782.49; ICD-10: I50, I11.0, I13.0, I13.2
Peripheral vascular disease	ICD-8: 440, 441, 442, 443, 444, 445; ICD-10: I70, I71, I72, I73, I74, I77
Cerebrovascular disease	ICD-8: 430–438; ICD-10: I60–I69, G45, G46
Dementia	ICD-8: 290.09–290.19, 293.09; ICD-10: F00–F03, F05.1, G30
Chronic pulmonary disease	ICD-8: 490–493, 515–518, ICD-10: J40–J47, J60–J67, J68.4, J70.1, J70.3, J84.1, J92.0, J96.1, J98.2, J98.3
Connective tissue disease	ICD-8: 712, 716, 734, 446, 135.99; ICD-10: M05, M06, M08, M09, M30, M31, M32, M33, M34, M35, M36, D86
Ulcer disease	ICD-8: 530.91, 530.98, 531–534; ICD-10: K22.1, K25–K28
Mild liver disease	ICD-8: 571, 573.01, 573.04; ICD-10: B18, K70.0–K70.3, K70.9, K71, K73, K74, K76.0

Diabetes type 1 and 2	Registrations in the National Diabetes Register
Score 2	
Hemiplegia	ICD-8: 344; ICD-10: G81, G82
Moderate to severe renal disease	ICD-8: 403, 404, 580–583, 584, 590.09, 593.19, 753.10–753.19, 792; ICD-10: 12, I13, N00–N05, N07, N11, N14, N17–N19, Q61
Diabetes with end organ damage	ICD-8: 249.01–249.05, 249.08, 250.01–250.05, 250.08; ICD-10: E10.2–E10.8, E11.2–E11.8, E14.2–E14.8
Any tumor	ICD-8: 140–194; ICD-10: C00–C75
Leukemia	ICD-8: 204–207; ICD-10: C91–C95
Lymphoma	ICD-8: 200–203, 275.59; ICD-10: C81–C85, C88, C90, C96
Score 3	
Moderate to severe liver disease	ICD-8: 070.00, 070.02, 070.04, 070.06, 070.08, 573.00, 456.00–456.09; ICD-10: B15.0, B16.0, B16.2, B19.0, K70.4, K72, K76.6, I85
Score 6	
Metastatic solid tumor	ICD-8: 195–198, 199; ICD-10: C76–C80
Acquired immune deficiency syndrome	ICD-8: 079.83; ICD-10: B21–B24
Age-scores<br>1 point: 50-59 years<br>2 point: 60-69 years<br>3 points: 70-79 years<br>4 points: 80-89 years<br>5 points: 90-99 years	

Covariables

Using the DNPR, the Prescription Registry, and the Danish National Diabetes Registry, we will identify previous records of: rheumatoid arthritis, systemic/subacute lupus erythematosus, inflammatory bowel disease, chronic obstructive pulmonary disease, asthma, diabetes (type I, type II, or unknown type), chronic kidney disease (chronic kidney disease stage 3 or higher, renal failure, chronic uraemia, dialysis or renal transplantation), human immunodeficiency virus infection, haematopoietic stem cell or bone marrow transplantation, solid organ transplantation (excluding renal transplants), or other cellular immune deficiency at any time before index date; leukaemia, lymphoma or myeloma within two years before the index date; and prescription records for oral glucocorticoids, other immunosuppressant drugs, or inhaled glucocorticoids within 90 days before the index date. For chronic obstructive pulmonary disease, we will include diagnoses of chronic

bronchitis and emphysema and required that patients were aged 35 years or older at first diagnosis. To capture active asthma, we will require that patients with a record of asthma also have an asthma-related prescription within the year before index date. Asthma patients are also required not to be classified as having chronic obstructive pulmonary disease. Diabetes will be identified using the Danish National Diabetes Registry. For descriptive purposes, we aimed to classify the type of diabetes as type I, type II, or unknown based on information on age at first record, age at first treatment and type of treatment. We will define type I diabetes as (1) age at first diagnosis ≤35 years and exclusive treatment with insulin prior to index date or (2) ≥2 insulin prescriptions at age ≤35 years but no diabetes diagnosis. Type II diabetes was defined as (1) age at first diabetes diagnosis >35 years or (2) exclusive treatment with oral anti-diabetics at age >35 years. We will classify remaining patients with diabetes as having unknown type.

We will also categorize educational level based on highest achieved education at index date as short (≤10 years), medium (>10–15 years), or long (>15 years). Educational level is included as a measure of socioeconomic status, which may be associated with health-seeking behavior.

We hypothesize that psychiatric disease, particularly depression and anxiety, may partly mediate the association between partner bereavement and HZ. We will therefore identify recent history of depression or anxiety, which we define as a diagnosis of depression, diagnosis of anxiety, or use of antidepressants within the past 90 days. We will exclude tricyclic antidepressant prescriptions among the antidepressants, because the competing indications insomnia and/or pain.

Code lists for all covariables are presented in the Appendix.

Statistical analysis

In the following, each step of the statistical analyses is described:

Protocol

1) We will produce descriptive statistics for all variables, including exposure. We will also stratify further by partner bereavement (overall and within 30 days before index date). With this information we can produce Table 1 and Appendix Table e1.

Table 1. Characteristics of herpes zoster cases and matched controls, Denmark, 1997–2013

	**Cases (n=)**	**Controls (n=)**
**Total**	x (x.x%)	x (x.x%)
**Matching factors**		
Sex	x (x.x%)	x (x.x%)
Males	x (x.x%)	x (x.x%)
Females	x (x.x%)	x (x.x%)
Age at index date (years)		
30–49	x (x.x%)	x (x.x%)
50–59	x (x.x%)	x (x.x%)
60–69	x (x.x%)	x (x.x%)
70–79	x (x.x%)	x (x.x%)
80–89	x (x.x%)	x (x.x%)
≥90	x (x.x%)	x (x.x%)
**Herpes zoster risk factors**		
Rheumatoid arthritis	x (x.x%)	x (x.x%)
Lupus erythematosus	x (x.x%)	x (x.x%)
Inflammatory bowel disease	x (x.x%)	x (x.x%)
Chronic obstructive pulmonary disease	x (x.x%)	x (x.x%)
Asthma	x (x.x%)	x (x.x%)
Chronic kidney disease	x (x.x%)	x (x.x%)
Diabetes	x (x.x%)	x (x.x%)
Type I	x (x.x%)	x (x.x%)
Type II	x (x.x%)	x (x.x%)
Unknown	x (x.x%)	x (x.x%)
Inhaled corticosteroids	x (x.x%)	x (x.x%)
Solid organ transplantation	x (x.x%)	x (x.x%)
HIV infection	x (x.x%)	x (x.x%)
Leukemia	x (x.x%)	x (x.x%)
Lymphoma	x (x.x%)	x (x.x%)
Myeloma	x (x.x%)	x (x.x%)
Hematopoietic stem cell/bone marrow transplantation	x (x.x%)	x (x.x%)
Other unspecified cellular immune deficiencies	x (x.x%)	x (x.x%)
Oral corticosteroids	x (x.x%)	x (x.x%)
Other immunosuppressive treatment	x (x.x%)	x (x.x%)

For presentation in text also compute:

- Median (interquartile range) of age.
- Partner status
- Highest achieved education

Highest achieved education (DK)		
Short	x (x.x%)	x (x.x%)
Medium	x (x.x%)	x (x.x%)
Long	x (x.x%)	x (x.x%)
Missing	x (x.x%)	x (x.x%)
**Civil status**		
Partner	x (x.x%)	x (x.x%)
No partner	x (x.x%)	x (x.x%)
Recently bereaved*	x (x.x%)	x (x.x%)

*Civil status is updated 1st of January each year. Thus, a person carries the civil status from January to the end of the year. For persons who loose their partner (bereaved persons) in the index year, we will not be able to know if a person is single or has already found a new partner. Thus a category for "recently bereaved" is included showing if a person was bereaved within the last year.

Supplementary Table e1. Characteristics of herpes zoster cases and matched controls, Denmark, 1997–2013, by previous partner bereavement (yes/no)

	**Cases (n=)**			**Controls (n=)**		
	**Partner bereavement, ever (n=)**	**Partner bereavement, previous 30 days (n=)**	**No partner bereavement (n=)**	**Partner bereavement, ever (n=)**	**Partner bereavement, previous 30 days (n=)**	**No partner bereavement (n=)**
**Matching factors**						
Sex	x (x.x%)	x (x.x%)	x (x.x%)	x (x.x%)	x (x.x%)	x (x.x%)
Males	x (x.x%)	x (x.x%)	x (x.x%)	x (x.x%)	x (x.x%)	x (x.x%)
Females	x (x.x%)	x (x.x%)	x (x.x%)	x (x.x%)	x (x.x%)	x (x.x%)
Age at index date (years)						
18–50	x (x.x%)	x (x.x%)	x (x.x%)	x (x.x%)	x (x.x%)	x (x.x%)
50–59	x (x.x%)	x (x.x%)	x (x.x%)	x (x.x%)	x (x.x%)	x (x.x%)
60–69	x (x.x%)	x (x.x%)	x (x.x%)	x (x.x%)	x (x.x%)	x (x.x%)
70–79	x (x.x%)	x (x.x%)	x (x.x%)	x (x.x%)	x (x.x%)	x (x.x%)
80–89	x (x.x%)	x (x.x%)	x (x.x%)	x (x.x%)	x (x.x%)	x (x.x%)
90–100	x (x.x%)	x (x.x%)	x (x.x%)	x (x.x%)	x (x.x%)	x (x.x%)
**Herpes zoster risk factors**						
Rheumatoid arthritis	x (x.x%)	x (x.x%)	x (x.x%)	x (x.x%)	x (x.x%)	x (x.x%)
Lupus erythematosus	x (x.x%)	x (x.x%)	x (x.x%)	x (x.x%)	x (x.x%)	x (x.x%)
Inflammatory bowel disease	x (x.x%)	x (x.x%)	x (x.x%)	x (x.x%)	x (x.x%)	x (x.x%)
Chronic obstructive pulmonary disease	x (x.x%)	x (x.x%)	x (x.x%)	x (x.x%)	x (x.x%)	x (x.x%)
Asthma	x (x.x%)	x (x.x%)	x (x.x%)	x (x.x%)	x (x.x%)	x (x.x%)
Chronic kidney disease	x (x.x%)	x (x.x%)	x (x.x%)	x (x.x%)	x (x.x%)	x (x.x%)
Diabetes	x (x.x%)	x (x.x%)	x (x.x%)	x (x.x%)	x (x.x%)	x (x.x%)
Type I	x (x.x%)	x (x.x%)	x (x.x%)	x (x.x%)	x (x.x%)	x (x.x%)
Type II	x (x.x%)	x (x.x%)	x (x.x%)	x (x.x%)	x (x.x%)	x (x.x%)
Unknown	x (x.x%)	x (x.x%)	x (x.x%)	x (x.x%)	x (x.x%)	x (x.x%)
Inhaled corticosteroids	x (x.x%)	x (x.x%)	x (x.x%)	x (x.x%)	x (x.x%)	x (x.x%)
Solid organ transplantation	x (x.x%)	x (x.x%)	x (x.x%)	x (x.x%)	x (x.x%)	x (x.x%)

HIV infection	x (x.x%)	x (x.x%)	x (x.x%)	x (x.x%)	x (x.x%)	x (x.x%)
Leukemia	x (x.x%)	x (x.x%)	x (x.x%)	x (x.x%)	x (x.x%)	x (x.x%)
Lymphoma	x (x.x%)	x (x.x%)	x (x.x%)	x (x.x%)	x (x.x%)	x (x.x%)
Myeloma	x (x.x%)	x (x.x%)	x (x.x%)	x (x.x%)	x (x.x%)	x (x.x%)
Hematopoietic stem cell/bone marrow transplantation	x (x.x%)	x (x.x%)	x (x.x%)	x (x.x%)	x (x.x%)	x (x.x%)
Other unspecified cellular immune deficiencies	x (x.x%)	x (x.x%)	x (x.x%)	x (x.x%)	x (x.x%)	x (x.x%)
Oral corticosteroids	x (x.x%)	x (x.x%)	x (x.x%)	x (x.x%)	x (x.x%)	x (x.x%)
Other immunosuppressive treatment	x (x.x%)	x (x.x%)	x (x.x%)	x (x.x%)	x (x.x%)	x (x.x%)

2) We will use conditional logistic regression to compute unadjusted odds ratios (ORs) with 99% confidence intervals (CIs), associating partner bereavement with HZ. We will then fit a multivariable conditional logistic regression model with adjustment for HZ risk factors. We will preform analyses overall and by time between bereavement and index date. The reference group for all analyses will be persons who have not previously experienced partner death. From this we can produce Table 2.

Table 2. Odds ratios for the association between partner bereavement and herpes zoster

	**Cases, n (%)**	**Controls, n (%)**	**Unadjusted odds ratio (99% confidence interval)**	**Adjusted odds ratio (99% confidence interval)***
No previous partner bereavement			–	–
Partner bereavement				
0–7 days				
8–14 days				
15–30 days				
31–90 days				
91–365 days				
366–1095 days				
>1095 days				

*Adjusted for rheumatoid arthritis, lupus erythematosus, inflammatory bowel disease, asthma, chronic kidney disease, diabetes, inhaled corticosteroids, solid organ transplantation, HIV infection, leukaemia, lymphoma, myeloma, hematopoietic stem cell/bone marrow transplantation, Other unspecified cellular immune deficiencies, oral glucocorticoids, other immunosuppressive treatment.

3) We will stratify the results for bereavement within 30 days before the index date by categories of risk of partner death, age, sex, and recent depression/anxiety. We will additionally perform this analysis for bereavement within 90 days before index date and present in Appendix Table e2.

Table 3. Odds ratios for the association between partner bereavement within previous 30 days and herpes zoster, subgroup analysis

	**Cases, number (%)**	**Controls, number (%)**	**Unadjusted odds ratio (99% confidence interval)**	**Adjusted odds ratio (99% confidence interval)***
Partners ACCI				
Low				
Intermediate				
High				
Partner terminal				
No				
Yes				
Sex				
Female				
Male				
Age at index date (years)				
18–50				
50–59				
60–69				
70–79				
80–89				
90–100				
Recent diagnosis of depression/anxiety				
No				
Yes				
*Adjusted for rheumatoid arthritis, lupus erythematosus, inflammatory bowel disease, asthma, chronic kidney disease, diabetes, inhaled corticosteroids, solid organ transplantation, HIV infection, leukaemia, lymphoma, myeloma, hematopoietic stem cell/bone marrow transplantation, Other unspecified cellular immune deficiencies, oral glucocorticoids, other immunosuppressive treatment.				

Supplementary Table e2. Odds ratios for the association between partner bereavement within previous 90 days and herpes zoster, subgroup analysis

	**Cases, number (%)**	**Controls, number (%)**	**Unadjusted odds ratio (99% confidence interval)**	**Adjusted odds ratio (99% confidence interval)***
Partners ACCI				
Low				
Intermediate				
High				
Partner terminal				
No				
Yes				
Sex				
Female				
Male				
Age at index date (years)				
18–50				
50–59				
60–69				
70–79				
80–89				
90–100				
Recent diagnosis of depression/anxiety				
No				
Yes				
*Adjusted for rheumatoid arthritis, lupus erythematosus, inflammatory bowel disease, asthma, chronic kidney disease, diabetes, inhaled corticosteroids, solid organ transplantation, HIV infection, leukaemia, lymphoma, myeloma, hematopoietic stem cell/bone marrow transplantation, Other unspecified cellular immune deficiencies, oral glucocorticoids, other immunosuppressive treatment.				

4) We will perform a sensitivity analysis repeating the main analysis among those who have a partner to see if using only persons who have a partner in the reference group affects the results.

Supplementary Table e3. Sensitivity analysis restricting to persons with partners

	**Cases, n (%)**	**Controls, n (%)**	**Unadjusted odds ratio (99% confidence interval)**	**Adjusted odds ratio (99% confidence interval)***

No previous partner bereavement			–	–
Partner bereavement				
0–7 days				
8–14 days				
15–30 days				
31–90 days				
91–365 days				
366–1095 days				
>1095 days				
No partner identified (vs. those with partner)				

*Adjusted for rheumatoid arthritis, lupus erythematosus, inflammatory bowel disease, asthma, chronic kidney disease, diabetes, inhaled corticosteroids, solid organ transplantation, HIV infection, leukaemia, lymphoma, myeloma, hematopoietic stem cell/bone marrow transplantation, Other unspecified cellular immune deficiencies, oral glucocorticoids, other immunosuppressive treatment.

5) We will perform a sensitivity analysis adjusting for education as a measure of socioeconomic status. In the analyses, persons with missing data on education will be included in the lower group.

Supplementary Table e4. Sensitivity analysis additionally adjusting for measures of patient level socioeconomic status, measured by the length of education in the Danish data

	**Adjusted odds ratio (99% confidence interval)*, fully adjusted model**
No previous partner bereavement	–
Partner bereavement	
0–7 days	
8–14 days	
15–30 days	
31–90 days	
91–365 days	
366–1095 days	
>1095 days	

*Adjusted for rheumatoid arthritis, lupus erythematosus, inflammatory bowel disease, asthma, chronic kidney disease, diabetes, inhaled corticosteroids, solid organ transplantation, HIV infection, leukaemia, lymphoma, myeloma, hematopoietic stem cell/bone marrow transplantation, Other unspecified cellular immune deficiencies, oral glucocorticoids, other immunosuppressive treatment.

6) Finally, we will perform two sensitivity analyses to examine the robustness of our prescription-based definition of HZ. In a stepwise manner, we will aim to increase the positive predictive value of our algorithm by making it more and more restrictive by each step. After each step, we will compute the adjusted odds ratio, to assess the effect of the increasing restriction. The steps are as follows:
    a. First, we will restrict to those with prescriptions for acyclovir in 800 mg tablet-dose in packages with 35 pills, valacyclovir in 500 mg tablet-dose, or famciclovir in 500 mg tablet-dose. This will be done by using the specific product number for the agents, which are:

Treatment in general practice (Prescription Registry)	VNR (Varenummer)
Acyclovir (J05AB01), 800 mg in packages of 35 tablets	005404, 007109, 044597, 057554, 078015, 082158, 106864, 397653, 434183, 447144, 470021, 480533, 496455, 515258, 516328, 560359
Valacyclovir (J05AB11), 500 mg	All VNR for the ATC code except for 030449, 172940, 447695, 498063, 534343
Famciclovir (J05AB09), 500 mg	088196, 455584, 494756, 550906

    b. Second, we will <u>also</u> restrict to cases with indication codes explicitly stating HZ (INDO 0000135 and 0000314):.
    c. Third, we will <u>also</u> restrict the analysis to those diagnosed in 2000 or later to ensure a prescription history of at least five years for ascertaining first-time prescription for antiviral drug and covariables.

Table e6. Sensitivity analysis testing the robustness of the prescription-based definition of herpes zoster <u>in Danish data</u>.

	**Adjusted odds ratio (99% confidence interval)***		
	**Step 1.** Restricting to herpes zoster specific doses	**Step 2.** Restricting to those with indication codes explicitly stating herpes zoster	**Step 3.** Restrict study period to 2000 or later.
No previous partner bereavement			

Partner bereavement, overall			
Partner bereavement, by time			
0–7 days			
8–14 days			
15–30 days			
31–90 days			
91–365 days			
366–1095 days			
*Adjusted for rheumatoid arthritis, lupus erythematosus, inflammatory bowel disease, asthma, chronic kidney disease, diabetes, inhaled corticosteroids, solid organ transplantation, HIV infection, leukaemia, lymphoma, myeloma, hematopoietic stem cell/bone marrow transplantation, Other unspecified cellular immune deficiencies, oral glucocorticoids, other immunosuppressive treatment.			

DATA SOURCES

- The Danish National Patient Registry
- The Danish Psychiatric Central Research Register
- The Danish National Prescription Registry
- The Danish National Diabetes Register
- The Civil Registration System
- Danish education registers at Statistics Denmark

APPROVALS

The study is approved by the Data Protection Agency.

REFERENCES

1 Serraino D, Franceschi S, Talamini R, Frustaci S, La Vecchia C. Non-occupational risk factors for adult soft-tissue sarcoma in northern Italy. *Cancer Causes Control* 1991; **2**: 157–64.

2 Wilson JF. Herpes zoster. *Ann Intern Med* 2011; **154**: ITC31–15.

3 Sheu J-J, Keller JJ, Lin HC. Increased risk of cancer after Bell's palsy: a 5-year follow-up study. *J Neurooncol* 2012; **110**: 215–20.

4 Smith JB, Fenske NA. Herpes zoster and internal malignancy. *South Med J* 1995; **88**: 1089–92.

5 Reiche EMV, Nunes SOV, Morimoto HK. Stress, depression, the immune system, and cancer. *Lancet Oncol* 2004; **5**: 617–25.

6 Glaser R, Kiecolt-Glaser JK. Stress-induced immune dysfunction: implications for health. *Nat Rev Immunol* 2005; **5**: 243–51.

7 Irwin M, Costlow C, Williams H, *et al.* Cellular immunity to varicella-zoster virus in patients with major depression. *J Infect Dis* 1998; **178 Suppl 1**: S104–8.

8 Irwin MR, Levin MJ, Carrillo C, *et al.* Major depressive disorder and immunity to varicella-zoster virus in the elderly. *Brain Behav Immun* 2011; **25**: 759–66.

9 Irwin MR, Levin MJ, Laudenslager ML, *et al.* Varicella zoster virus-specific immune responses to a herpes zoster vaccine in elderly recipients with major depression and the impact of antidepressant medications. *Clin Infect Dis* 2013; **56**: 1085–93.

10 Miller MA, Rahe RH. Life changes scaling for the 1990s. *J Psychosom Res* 1997; **43**: 279–92.

11 Harpaz R, Leung JW, Brown CJ, Zhou FJ. Psychological stress as a trigger for herpes zoster: might the conventional wisdom be wrong? *Clin Infect Dis* 2015; **60**: 781–5.

12 Lasserre A, Blaizeau F, Gorwood P, *et al.* Herpes zoster: family history and psychological stress-case-control study. *J Clin Virol* 2012; **55**: 153–7.

13 Schmader K, Studenski S, MacMillan J, Grufferman S, Cohen HJ. Are stressful life events risk factors for herpes zoster? *J Am Geriatr Soc* 1990; **38**: 1188–94.

14 Schmader K, George LK, Burchett BM, Hamilton JD, Pieper CF. Race and stress in the incidence of herpes zoster in older adults. *J Am Geriatr Soc* 1998; **46**: 973–7.

15 Schmader K, George LK, Burchett BM, Pieper CF. Racial and psychosocial risk factors for herpes zoster in the elderly. *J Infect Dis* 1998; **178 Suppl 1**: S67–70.

16 Schmidt M, Pedersen L, Sørensen HT. The Danish Civil Registration System as a tool in epidemiology. *Eur J Epidemiol* 2014; **29**: 541–9.

17 Lynge E, Sandegaard JL, Rebolj M. The Danish National Patient Register. *Scand J Public Health* 2011; **39**: 30–3.

18 Mors O, Perto GP, Mortensen PB. The Danish Psychiatric Central Research Register. *Scand J Public Health* 2011; **39**: 54–7.

19 Kildemoes HW, Sørensen HT, Hallas J. The Danish National Prescription Registry. *Scand J Public Health* 2011; **39**: 38–41.

20 Carstensen B, Kristensen JK, Marcussen MM, Borch-Johnsen K. The National Diabetes Register. *Scand J Public Health* 2011; **39**: 58–61.

21 Jensen VM, Rasmussen AW. Danish Education Registers. *Scand J Public Health* 2011; **39**: 91–4.

22 Charlson M, Szatrowski TP, Peterson J, Gold J. Validation of a combined comorbidity index. *J Clin Epidemiol* 1994; **47**: 1245–51.

Appendix. Codes used to identify covariables

Table 1. International classification of diseases (ICD) version 10 codes

Depression

DF0003	Dementia in early onset Alzheimers disease with depressive symptoms
DF0013	Dementia in late onset Alzheimers disease with depressive symptoms
DF0023	Dementia in atypical Alzheimers disease with depressive symptoms
DF0093	Dementia in Alzheimers disease with depressive symptoms, without further specification
DF0103	Vascular dementia with acute debut with depressive symptoms
DF0113	Multi-infarction dementia with depressive symptoms
DF0123	Subcortical vascular dementia with depressive symptoms
DF0133	Mixed cortical and subcortical vascular dementia with depressive symptoms
DF0183	Other vascular dementia with depressive symptoms
DF0193	Vascular dementia with depressive symptoms, without further specification
DF0203	Dementia in Picks disease with depressive symptoms
DF0213	Dementia in Creutzfeldt-Jakobs disease with depressive symptoms
DF0223	Dementia in Huntingtons disease with depressive symptoms
DF0233	Dementia in Parkinsons disease with depressive symptoms
DF0243	Dementia in HIV with depressive symptoms
DF0283	Dementia with depressive symptoms in other disease
DF0393	Dementia with depressive symptoms, without further specification
DF0632	Organic depressive mental disease
DF1054	Alkoholbetinget psykotisk tilstand, med depressive symptome
DF1154	Psykotisk tilstand opioidbetinget, med depressive
DF1254	Cannabisbetinget psykotisk tilstand med depressive
DF1354	Sedativa-hypnotikabetinget psykotisk tilst, depressi
DF1454	Kokainbetinget psykotisk tilstand med depressive
DF1554	Stimulantiabetinget psykotisk tilstand, depressive
DF1654	Hallucinogenbetinget psykotisk tilstand, depressi
DF1854	Snifferstofbetinget psykotisk tilstand, depressi
DF1954	Psykotisk tilstand flere/andre stoffer, depressive
DF204	Post-skizofren depression
DF2040	Kontinuerlig post-skizofren depression
DF2041	Episodisk post-skizofren depression m fremadskridende defekt
DF2042	Episodisk post-skizofren depression med stabil defekt
DF2043	Episodisk remitterende post-skizofren depression
DF2044	Post-skizofren depression med inkomplet remission
DF2045	Post-skizofren depression med komplet remission
DF2048	Post-skizofren depression med andet forløb
DF2049	Post-skizofren depression med ubestemt forløb

DF25	Skizoaffektive psykoser
DF250	Skizoaffektiv psykose af manisk type
DF2500	Skizoaffektiv manisk psykose med samtidige symptomer
DF2501	Skizoaffektiv manisk psykose med varige skizofrene symptomer
DF251	Skizoaffektiv psykose af depressiv type
DF2510	Skizoaffektiv depressiv psykose med samtididige symptomer
DF2511	Skizoaffektiv depressiv psykose med varige skizofrene sympt.
DF252	Skizoaffektiv blandet manisk-depressiv psykose
DF2520	Skizoaffektiv bland. man-depr. psykose m samtidige symptomer
DF2521	Skizoaffektiv man-depr.psykose m varige skizofrene symptomer
DF258	Skizoaffektiv psykose af anden type
DF2580	Anden skizoaffektiv psykose med samtidige symptomer
DF2581	Anden skizoaffektiv psykose med varige skizofrene symptomer
DF259	Skizoaffektiv psykose UNS
DF2590	Skizoaffektiv psykose UNS med samtidige symtomer
DF2591	Skizoaffektiv psykose UNS med varige skizofrene symptomer
DF32	Depressiv enkeltepisode
DF320	Depressiv enkeltepisode af lettere grad
DF3200	Depressiv non-melankoliform enkeltepisode af lettere grad
DF3201	Depressiv melankoliform enkeltepisode af lettere grad
DF321	Depressiv enkeltepisode af moderat grad
DF3210	Depressiv non-melankoliform enkeltepisode af moderat grad
DF3211	Depressiv melankoliform enkeltepisode af moderat grad
DF322	Depress. enkeltepisode af svær grad u psykot. sympt.
DF323	Depressiv enkeltepisode af svær grad m. psykotiske symptomer
DF3230	Depress. enkeltepisode, svær grad, stemn.kongr.psykot. Sympt
DF3231	Depress.enkeltepisode, svær grad, stemn.inkongr.psykot.sympt
DF328	Depressiv enkeltepisode af anden type
DF329	Depressiv enkeltepisode UNS
DF329A	Depressiv tilstand uden specifikation
DF33	Periodisk depression
DF330	Periodisk depression i episode af lettere grad
DF3300	Periodisk non-melankoliform depression i episode af let grad
DF3301	Periodisk melankoliform depression i episode af let grad
DF331	Periodisk depression i episode af moderat grad
DF3310	Periodisk non-melankoliform depression, moderat grad
DF3311	Periodisk melankoliform depression i episode af moderat grad
DF332	Periodisk depression i episode af svær grad u psykot. sympt.
DF333	Periodisk depression i episode af svær grad m psykot. sympt.
DF3330	Periodisk depression, svær grad, stemningskongr.psykot.sympt
DF3331	Periodisk depression, svær grad, stemn.inkongr. psykot.sympt
DF334	Periodisk depression i remission
DF338	Periodisk depression af anden type

DF339	Periodisk depression UNS
DF341	Dystymi
DF3410	Depressiv personlighedsstruktur
DF3411	Kronisk lettere depressionstilstand
DF3810	Affektiv sindslid. m. tilbagevend., kortvarige depressioner
DF530	Lettere psykisk lidelse i barselsperioden IKA
DF530A	Barselsdepression
DO993B2	Depression som komplicerer graviditet, fødsel eller barselsperiode

Anxiety

DF064	Organisk angsttilstand
DF40	Fobiske angsttilstande
DF400	Agorafobi
DF4000	Agorafobi uden panikangsst
DF4001	Agorafobi med panikangst
DF401	Socialfobi
DF402	Enkelfobi
DF402A	Parasitofobi
DF408	Fobisk angst af anden type
DF408A	Venerofobi
DF409	Fobisk angst UNS
DF41	Andre angsttilstande
DF410	Panikangst
DF4100	Moderat panikangst
DF4101	Svær panikangst
DF411	Generaliseret angst
DF412	Lettere angst-depressionstilstand
DF413	Anden blandet angsttilstand
DF418	Anden angsttilstand
DF418A	Fødselsangst
DF418B	Angsthysteri
DF419	Angsttilstand UNS
DO993B3	Angsttilstand som komplicerer graviditet, fødsel eller barselsperiode

Rheumatoid arthritis

DG737D	Myopathy, arthritis rheumatoides
DI328A	Rheumatoid pericarditis
DI398E	Rheumatoid endocarditis
DI418A	Rheumatoid myocarditis
DI528A	Rheumatoid carditis
DJ990	Rheumatoid lung disease
DM05	Seropositive rheumatoid arthritis
DM050	Felty's syndrome
DM050A	Arthritis rheumatoides med splenoadenomegali og leukopeni
DM051	Rheumatoid lung disease
DM051	Reumatoid lungesygdom

DM051A	Reumatoid artritis med Caplans syndrom
DM051B	Effusio pleurae rheumatoides
DM051B	Reumatoid artritis med pleural effusion
DM051C	Fibrosis pulmonis interstitialis diffusa rheumatoides
DM051C	Reumatoid artritis med interstitiel diffus lungefibrose
DM051D	Pleuritis rheumatoides
DM051D	Reumatoid artritis med pleuritis
DM051E	Noduli rheumatici pulmonis
DM051E	Reumatoid artritis med noduli rheumatici pulmonis
DM051F	Granuloma pulmonis rheumatoides
DM051F	Reumatoid artritis med granulomer i lungerne
DM052	Rheumatoid vasculitis
DM052	Vasculitis rheumatoides
DM052	Reumatoid vaskulitis
DM053	Rheumatoid arthritis with involvement of oth organs and sys
DM053	Arthritis rheumatoides med involvering af andre organsyst
DM053	Reumatoid artritis med involvering af andre organsystemer
DM053A	Arthritis rheumatoides med endocardit
DM053B	Arthritis rheumatoides med myocardit
DM053C	Arthritis rheumatoides med myopati
DM053D	Polyneuropathia rheumatoides
DM053E	Arthritis rheumatoides med pericardit
DM058	Other seropositive rheumatoid arthritis
DM059	Seropositive rheumatoid arthritis, unspecified
DM060	Seronegative rheumatoid arthritis
DM061	Adult-onset Still's disease
DM062	Rheumatoid bursitis
DM063	Rheumatoid nodule
DM068	Other specified rheumatoid arthritis
DM069	Rheumatoid arthritis, unspecified
DM080	Juvenile rheumatoid arthritis
DM080A	Juvenil reumatoid artritis med rheumafaktor
DM080B	Arthritis juvenilis chronica uden rheumafaktor
DM082	Juvenile arthritis with systemic onset
DM082A	Stills sygdom med debut før det fyldte 16 år
DM082B	Still's sygdom uden specifikation
DM083	Juvenile polyarthritis (seronegative)
DM084	Pauciarticular juvenile arthritis

Lupus erythematosus

DL931	Subacute cutaneous lupus erythematosus
DG058A	Encephalitis, systemisk lupus erythematosus
DG737C	Myopathia, systemisk lupus erythematosus
DI328B	Pericarditis, systemisk lupus erythematosus
DI398C	Endocarditis, Libman-Sacks, systemisk lupus erythematosus
DJ991C	Lungesygdom, systemisk lupus erythematosus

DL932	Anden form for lokaliseret lupus erythematosus
DL932A	Lupus erythematosus profundus
DL932B	Lupuspanniculitis
DL932C	Lupus erythematosus tumidus
DM32	Systemic lupus erythematosus
DM320	Systemisk lupus erythematosus forårsaget af lægemiddel
DM321	Systemisk lupus erythematosus med organinvolvering
DM321A	Libman-Sacks' endocarditis (systemisk lupus erythematosus)
DM321B	Glomerulonephritis ved systemisk lupus erythematosus
DM321C	Pleuritis ved systemisk lupus erythematosus
DM321D	Polyserositis, systemisk lupus erythematosus
DM321E	Lupus pericarditis
DM328	Systemisk lupus erythematosus, andre former
DM329	Systemisk lupus erythematosus UNS
DN085A	Glomerulonephropathia ved systemisk lupus erythematosus
DN164B	Nephropathia tubulointerstit ved syst lupus erythematosus

Inflammatory bowel disease

DK50	Crohn's sygdom
DK500	Crohns sygdom i tyndtarmen
DK500A	Crohns sygdom i duodenum
DK500B	Crohns sygdom i ileum
DK500C	Crohns sygdom i jejunum
DK500D	Ileitis terminalis
DK501	Crohns sygdom i tyktarmen
DK501A	Colitis granulomatosa
DK501B	Colitis regionalis
DK501C	Enteritis regionalis coli
DK501D	Enteritis regionalis recti
DK508	Anden form for Crohns sygdom
DK508A	Crohns sygdom i både tyndtarmen og tyktarmen
DK508B	Enteritis regionalis, både tynd- og tyktarm
DK508C	Crohns sygdom med forandringer i mundhulen
DK508D	Crohns sygdom med ileocøkal lokalisation
DK509	Crohns sygdom UNS
DK51	Ulcerøs colitis
DK510	Ulcerøs pancolitis
DK511	Ileocolitis (chronica) ulcerosa
DK512	Ulcerøs proktitis
DK512A	Proctitis haemorrhagica ulcerosa
DK513	Ulcerøs proktosigmoiditis
DK513A	Rectosigmoiditis haemorrhagica ulcerosa
DK514	Inflammatoriske polypper
DK515	Venstresidig ulcerøs colitis
DK515A	Venstresidig proktocolitis
DK515B	Venstresidig hemicolitis

DK518	Anden form for ulcerøs colitis
DK518A	Pancolitis ulcerosa
DK518B	Ulcerøs colitis med forandringer i mundhulen
DK519	Ulcerøs colitis UNS
DM074	Arthritis enteropathica ved Crohn's sygdom
DM075	Artropati ved colitis ulcerosa
DM091	Juvenil artritis ved Crohns sygdom
DM092	Juvenil artritis ved ulcerøs colitis

Chronic obstructive pulmonary disease

DJ41	Kronisk bronkitis uden obstruktion
DJ410	Simpel kronisk bronkitis
DJ411	Mukopurulent kronisk bronkitis
DJ418	Blandet simpel og mukopurulent kronisk bronkitis
DJ42	Kronisk bronkitis ikke spec.
DJ429	Kronisk bronkitis UNS
DJ429A	Tracheobronchitis chronica
DJ429B	Kronisk trakeitis
DJ43	Lungeemfysem
DJ430	Enkeltsidigt lungeemfysem
DJ430A	Emphysema pulmonum unilaterale
DJ431	Panlubulært lungeemfysem
DJ431A	Panacinært emfysem
DJ432	Centrilobulært emfysem
DJ438	Anden form for lungeemfysem
DJ439	Lungeemfysem UNS
DJ439A	Bulløst lungeemfysem
DJ44	Kronisk obstruktiv lungesygdom
DJ440	Kronisk obstr. lungesygd. m. akut nedre luftvejs infektion
DJ441	Kronisk obstruktiv lungesygdom med akut eksacerbation UNS
DJ448	Anden form for kronisk obstruktiv lungesygdom
DJ448A	Kronisk obstruktiv bronkitis
DJ448B	Kronisk astmatisk bronkitis
DJ448C	Kronisk bronkitis med emfysem
DJ449	Kronisk obstruktiv lungesygdom UNS

Asthma

DJ45	Astma
DJ450	Asthma bronchiale allergicum
DJ450A	Erhvervsbetinget allergisk astma
DJ451	Asthma bronchiale non allergicum
DJ451A	Erhvervsbetinget ikke-allergisk astma
DJ458	Astma af blandet type
DJ459	Astma UNS
DJ459A	Astmatisk bronkitis
DJ46	Status astmaticus
DJ469	Status asthmaticus UNS

Chronic kidney disease

DL298C	Uræmisk kløe
DG638A	Neuropathia uraemica
DE853B	Amyloidosis ved hæmodialyse
DT825A	Malfunktion af a-v fistel
DT825B	Malfunktion af hæmodialyse kateter
DT825C	Malfunktion af peritonealdialyse kateter
DT856C	Complicatio katheterisationis intraperitonealis dialyseos
DI120	Morbus hypertensivus renalis med nyresvigt
DI131	Morbus cordis et renalis hypertensivus med nyresvigt
DI132	Mb cordis et renalis hypertens med hjertesvigt og nyresvigt
DI770	Erhvervet arteriovenøs fistel
DI770A	Aneurysma varicosum
DN165	Nefropati ved afstødning af transplantat
DN180	Terminal nyreinsufficiens
DN183	Kronisk nyreinsufficiens, stadie 3
DN184	Kronisk nyreinsufficiens, stadie 4
DN185	Kronisk nyreinsufficiens, terminal stadie 5
DN188	Kronisk nyreinsufficiens, andre former
DN188A	Neuropathia, uræmisk
DN188B	Pericarditis, uræmisk
DN189	Kronisk nyreinsufficiens UNS
DN19	Nyreinsufficiens UNS
DN199	Uræmi uden specifikation
DT824	Complicatio katheterisationis vasorum dialyseos
DT861	Svigt eller afstødning af transplanteret nyre
DT861A	Insufficientia transplantationis renis
DT861B	Afstødning af nyretransplantat
DZ49	Dialysebehandling
DZ490	Forbehandling m henblik på dialyse
DZ491	Ekstracorporal dialyse
DZ492	Dialyse, anden
DZ94	Tilstand med transplanteret organ eller væv
DZ940	Tilstand m transplanteret nyre
DZ940	Nyretransplanteret
DZ992	Afhængighed af renal dialyse
DT817E1	Lymphocele efter nyretransplantation

Diabetes type 1 (used to identify subtypes)

DE10	Type 1-diabetes
DE100	Type 1-diabetes med koma
DE100A	Coma diabeticum, hyperosmolær ved IDDM
DE100B	Coma diabeticum ved IDDM med ketoacidose
DE100C	Coma diabeticum ved IDDM uden ketoacidose
DE100D	Coma diabeticum, hyperglykæmisk ved IDDM

DE100E	Coma diabeticum, hypoglykæmisk ved IDDM
DE100F	Diabetes mellitus insulino dependente med coma diabeticum
DE101	Type 1-diabetes med ketoacidose
DE102	Type 1-diabetes med nyrekomplikation
DE103	Type 1-diabetes med øjenkomplikation
DE104	Type 1-diabetes med neurologisk komplikation
DE105	Type 1-diabetes med komplikationer i perifere karsystem
DE105A	Type 1-diabetes med perifer angiopati
DE105B	Type 1-diabetes med fodsår
DE105C	Type 1-diabetes med gangræn
DE105D	Type 1-diabetes med mikroangiopati
DE106	Type 1-diabetes med anden komplikation
DE107	Type 1-diabetes med multiple komplikationer
DE108	Type 1-diabetes med komplikation UNS
DE109	Type 1-diabetes uden komplikationer
DE109A	Type 1-diabetes UNS
DO240	Graviditet, fødsel el. barsel m. forud best. type 1-diabetes
DO240A	Svangerskab med forud bestående type 1-diabetes
DO240B	Fødsel med forud bestående type 1-diabetes
DO240C	Barsel med forud bestående type 1-diabetes

Diabetes type 2 (used to identify subtypes)

DE11	Type 2-diabetes
DE110	Type 2-diabetes med koma
DE110A	Coma diabeticum ved NIDDM uden ketoacidose
DE110B	Coma diabeticum, hypoglykæmisk ved NIDDM
DE110C	Coma diabeticum, hyperosmolær ved NIDDM
DE110D	Coma diabeticum, hyperglykæmisk ved NIDDM
DE110E	Coma diabeticum ved NIDDM med ketoacidose
DE111	Type 2-diabetes med ketoacidose
DE112	Type 2-diabetes med nyrekomplikation
DE113	Type 2-diabetes med øjenkomplikation
DE114	Type 2-diabetes med neurologisk komplikation
DE115	Type 2-diabetes med komplikationer i perifere karsystem
DE115A	Type 2-diabetes med perifer angiopati
DE115B	Type 2-diabetes med fodsår
DE115C	Type 2-diabetes med gangræn
DE115D	Type 2-diabetes med mikroangiopati
DE116	Type 2-diabetes med anden komplikation
DE117	Type 2-diabetes med multiple komplikationer
DE118	Type 2-diabetes med komplikation UNS
DE119	Type 2-diabetes uden komplikationer
DE119A	Type 2-diabetes UNS
DO241	Graviditet, fødsel el. barsel m. forud best. type 2-diabetes
DO241A	Svangerskab med forud bestående type 2-diabetes
DO241B	Fødsel med forud bestående type 2-diabetes
DO241C	Barsel med forud bestående type 2-diabetes

Protocol

HIV

DB20	HIV sygdom medførende infektiøse og parasitære sygdomme
DB200	HIV-sygdom med mykobakteriel infektion
DB200A	HIV-sygdom med tuberkulose
DB201	HIV-sygdom med andre bakterielle sygdomme
DB202	HIV-sygdom med cytomegalvirus sygdom
DB203	HIV-sygdom med andre virus infektioner
DB204	HIV-sygdom med candidiasis
DB205	HIV-sygdom med andre svampeinfektioner
DB206	HIV-sygdom med Pneumocystis jirovecii pneumoni
DB207	HIV-sygdom med multiple infektioner
DB208	HIV-sygdom med andre infektiøse og parasitære sygdomme
DB209	HIV-sygdom med infektiøs eller parasitær sygdom UNS
DB21	Humant immundefekt-virus (HIV) sygdom med neoplastisk sygdom
DB210	HIV-sygdom med Kaposi's sarkom
DB210A	HIV-infektion med Kaposi sarkom
DB211	HIV-sygdom med Burkitt's lymfom
DB212	HIV-sygdom med andet non-Hodgkin's lymfom
DB213	HIV-sygdom med anden neoplasi i lymfoidt og bloddannende væv
DB217	HIV-sygdom med multiple neoplasier
DB218	HIV-sygdom med anden neoplasi
DB219	HIV sygdom med neoplasi UNS
DB22	HIV sygdom med andre specificerede sygdomme
DB220	HIV-encephalopati
DB220A	HIV-demens
DB221	HIV-lymfoid interstitiel pneumonitis
DB222	HIV-wasting syndrome
DB227	HIV-sygdom med multiple sygdomme klassificeret andetsteds
DB23	Andre tilstande ved HIV-sygdom
DB230	Akut HIV-sygdom
DB231	HIV-sygdom med (persisterende) generaliseret lymfadenopati
DB232	HIV-sygdom med hæmatologiske og immunologiske forandr. IKA
DB238	Anden eller ikke specificeret symptomatisk HIV-sygdom
DB238A	HIV-associeret lipodystrofisyndrom (HALS)
DB24	HIV-sygdom og AIDS uden specificering
DB240	HIV infektion, uden symptomer
DB249	AIDS UNS
DF024	Demens ved HIV-sygdom
DF0240	Demens ved HIV-sygdom uden andre symptomer
DF0241	Demens ved HIV-sygdom med paranoide symptomer
DF0242	Demens ved HIV-sygdom med hallucinationer
DF0243	Demens ved HIV-sygdom med depressive symptomer
DF0244	Demens ved HIV-sygdom med blandede symptomer
DZ21	Asymptomatisk HIV-infektion
DZ219	Asymptomatisk HIV-infektion UNS

Lymphoma

DC81	Hodgkin lymfomer
DC810	Nodulært lymfocytdomineret Hodgkin lymfom
DC811	Klassisk Hodgkin lymfom med nodulær sklerose
DC812	Klassisk Hodgkin lymfom med blandet cellularitet
DC813	Lymphoma Hodgkin 'lymphocytic depletion'
DC814	Klassisk lymfocytrigt Hodgkin lymfom
DC817	Andet klassisk Hodgkin lymfom
DC819	Hodgkin lymfom UNS
DC82	Follikulære lymfomer
DC820	Follikulært lymfom, grad I
DC821	Follikulært lymfom, grad II
DC822	Follikulært lymfom, grad III
DC825	Diffust follikelcenter lymfom
DC826	Kutant follikelcenter lymfom
DC827	Andet follikulært lymfom
DC829	Follikulært lymfom UNS
DC83	Ikke-follikulære lymfomer
DC830	Småcellet B-celle lymfom
DC830B	Lymfoplasmacytært lymfom
DC830C	Nodalt marginalzonelymfom
DC830D	Splenisk marginalzonelymfom
DC831	Mantelcelle lymfom
DC831A	Centrocytisk lymfom
DC831B	Malign lymfomatøs polypose
DC832	Lymph mal non-Hodg af diff type mixed small and large cell
DC833	Diffust storcellet B-celle lymfom
DC833A	Anaplastisk diffust storcellet B-celle lymfom
DC833B	CD30-positivt diffust storcellet B-celle lymfom
DC833C	Centroblastært diffust storcellet B-celle lymfom
DC833D	Immunoblastært diffust storcellet B-celle lymfom
DC833E	Plasmablastært diffust storcellet B-celle lymfom
DC833F	T-cellerigt diffust storcellet B-celle lymfom
DC834	Lymphoma mal non-Hodgkin af diffus immunoblastær type
DC835	Lymfoblastært lymfom
DC835A	Lymfoblastært B-celle lymfom
DC835B	Lymfoblastært T-celle lymfom
DC835C	Lymfoblastært lymfom UNS
DC836	Lymphoma mal non-Hodgkin af diffus udifferentieret type
DC837	Burkitt lymfom
DC837A	Atypisk Burkitt lymfom
DC837B	Burkitt-lignende lymfom
DC838	Andet ikke-follikulært lymfom
DC838A	Primært effusionslymfom (af B-celle type)
DC838B	Intravaskulært storcellet B-celle lymfom
DC839	Ikke-follikulært (diffust) lymfom UNS

DC84	Modne NK/T-celle lymfomer
DC840	Mycosis fungoides
DC840A	Mycosis fungoides associeret med follikulær mucinose
DC840B	Pagetoid retikulose
DC840C	Granulomatøs slack skin (lymphoma malignum)
DC841	Morbus Sézary
DC842	Lymphoma mal T-zone
DC843	Lymphoma mal lymfoepiteloidt
DC844	Perifert T-celle lymfom UNS
DC844A	Storcellet Pleomorft CD30+ T-celle lymfom
DC844B	Storcellet Pleomorft CD30- T-celle lymfom
DC844C	Storcellet immunoblastisk CD30+ T-celle lymfom
DC844D	Storcellet immunoblastisk CD30- T-celle lymfom
DC844E	Middel-småcellet pleomorft T-celle lymfom
DC844F	Lymfoepitelioidt lymfom
DC844H	Modent T-celle lymfom UNS
DC845	Andet modent NK/T-celle lymfom
DC845A	Storcellet anaplastisk CD30+ T-celle lymfom
DC845B	Storcellet anaplastisk CD30+ T-celle lymfom, primært i huden
DC845C	Subkutant pannikulitrelateret T-lymfom
DC845D	Angiocentrisk T-/NK-celle lymfom
DC846	Anaplastisk storcellet lymfom, ALK-positivt
DC846A	Anaplastisk storcellet lymfom, CD30-positivt
DC847	Anaplastisk storcellet lymfom, ALK-negativt
DC848	Kutant T-celle lymfom UNS
DC849	Modent NK/T-celle lymfom UNS
DC85	Andre og ikke specificerede non-Hodgkin lymfomer
DC850	Lymphosarcoma
DC851	B-celle lymfom UNS
DC851A	Follikelcenter B-celle lymfom
DC851B	Marginal zone B-celle lymfom (immunocytom)
DC851C	Diffust storcellet B-celle lymfom
DC851D	T-cellerigt B-celle lymfom
DC851E	Storcellet intravaskulært B-celle lymfom
DC852	Mediastinalt (tymisk) storcellet B-celle lymfom
DC857	Andet non-Hodgkin lymfom
DC857A	Reticulosis lymphatica follicularis
DC857B	Polymorf posttransplantations lymfoproliferativ sygd. (PTLD)
DC859	Lymfom (neoplasi) UNS
DC859A	Malignt lymfom uden specifikation
DC859B	Lymfom uden specifikation
DC86	Andre NK/T-celle lymfomer
DC860	Ekstranodalt NK/T-celle lymfom, nasal type
DC861	Hepatosplenisk T-celle lymfom
DC862	Enteropati-type (intestinalt) T-celle lymfom
DC863	Subkutant panniculitis-lignende T-celle lymfom

DC864	Blastisk NK-celle lymfom
DC865	Angioimmunoblastært T-celle lymfom
DC866	Primært kutane CD30-positive T-celle proliferationer
DC866A	Lymfomatoid papulose
DC866B	Primært kutant anaplastisk storcellet lymfom
DC866C	Primært kutant CD30-positivt storcellet lymfom
DC88	Maligne immunoproliferative sygdomme
DC880	Waldenströms makroglobulinæmi
DC881	Paraproteinaemia alpha heavy chain
DC882	Anden heavy chain disease
DC882A	Gamma heavy chain disease
DC882B	My heavy chain disease
DC883	Neopl mal immunoproliferativum intestini tenuis
DC883A	Alpha heavy chain disease
DC883B	Middelhavs lymfom
DC884	Ekstranodalt marginalzone B-celle lymfom
DC884A	Lymfom af mucosa-associeret lymfoidt væv (MALT-lymfom)
DC884B	Lymfom af hud-associeret lymfoidt væv (SALT-lymfom)
DC884C	Lymfom af bronkie-associeret lymfoidt væv (BALT-lymfom)
DC887	Anden malign immunoproliferativ sygdom
DC889	Malign immunoproliferativ sygdom UNS
DC96	An/ikke-spec maligne neoplasier fra lymfoidt/hæmatopoidt væv
DC960	Multifokal og multisyst. Langerhans-celle histiocytosis
DC960A	Histiocytosis X, multisystemisk
DC961	Histiocytosis maligna
DC962	Malign mastcelle tumor
DC962B	Mastcelle sarkom
DC963	Lymphoma mal histiocyticum verum
DC964	Dendritcelle sarkom (accessoriske celler)
DC964A	Interdigiterende dendritcelle sarkom
DC964B	Langerhans-celle sarkom
DC964C	Follikulært dendritcelle sarkom
DC965	Multifokal og unisystemisk Langerhans-celle histiocytose
DC965A	Hand-Schüller-Christian sygdom
DC965B	Histiocytosis X, multifokal
DC966	Unifokal Langerhans-celle histiocytose
DC966A	Eosinofilt granulom
DC966B	Histiocytosis X, unifokal
DC966C	Histiocytosis X UNS
DC966D	Langerhans-celle histiocytose UNS
DC967	Anden malign neoplasi fra lymfoidt eller hæmatopoietisk væv
DC967A	Histiocytært sarkom
DC968	Malign histiocytose UNS
DC969	Malign neoplasi fra lymfoidt eller hæmatopoietisk væv UNS

Leukaemia

DC91	Lymfatiske leukæmier

Protocol

DC910	Akut lymfoblastær leukæmi (ALL)
DC911	Leukaemia lymphatica chronica
DC911A	Lymfoplasmacytær leukæmi
DC911B	Richter syndrom
DC912	Leukaemia lymphatica subacuta
DC913	Leukaemia prolymphocytica
DC914	Hårcelle leukæmi
DC914A	Reticuloendotheliosis leucaemica
DC915	Leukaemia T-lymfocytær hos voksne
DC915A	Adult T-celle lymfom/leukæmi (HTLV-1-assoc.), akut variant
DC915B	Adult T-celle lymfom/leukæmi (HTLV-1-assoc.), kron. Variant
DC915C	Adult T-celle lymfom/leukæmi (HTLV-1-assoc), lymfomatoid var
DC915D	Adult T-celle lymfom/leukæmi (HTLV-1-assoc), smouldering var
DC916	Prolymfocyt leukæmi af T-celle type
DC917	Leukaemia lymphatica anden form
DC917A	Leukaemia aleukaemica lymphatica
DC917B	Large granular T-celle lymfocytær leukæmi
DC918	Moden B-celle leukæmi af Burkitt-type
DC919	Leukaemia lymphatica uden specifikation
DC92	Myeloide leukæmier
DC920	Leukaemia myeloblastica acuta
DC920A	Akut myeloblastær leukæmi med minimal differentiering
DC920B	Akut myeloblastær leukæmi med modning
DC920C	Akut myeloblastær leukæmi, AML1/ETO
DC920D	Akut myeloblastær leukæmi, AML M0
DC920E	Akut myeloblastær leukæmi, AML M1
DC920F	Akut myeloblastær leukæmi, AML M2
DC920G	Akut myeloblastær leukæmi, AML med t(8;21)
DC920H	Akut myeloblastær leukæmi UNS (uden FAB-klassificering)
DC920J	Refraktær anæmi m overskud af blaster i transformation
DC921	Leukaemia myeloides chronica
DC921A	Kronisk myeloid leukæmi med blastkrise
DC922	Leukaemia myeloides subacuta
DC923	Myeloidt sarkom
DC923A	Chloroma
DC923B	Granulocytært sarkom
DC924	Leukaemia promyelocytica
DC924A	Akut myeloblastær leukæmi M3 med t(15;17) og varianter
DC925	Leukaemia myelomonocytica acuta
DC925A	Akut myeloblastær leukæmi M4 Eo med inv(16) eller t(16;16)
DC926	Akut myeloblastær leukæmi med 11q23-abnormalitet
DC926A	Akut myeloblastær leukæmi med variation af MLL-gen
DC927	Leukaemia myeloides anden form
DC927A	Leukaemia aleukaemica myeloides
DC927B	Kronisk neutrofil leukæmi
DC928	Akut myeloid leukæmi med multilinje dysplasi

DC929	Leukaemia myeloides uden specifikation
DC93	Monocytære leukæmier
DC930	Leukaemia monocytica acuta
DC930A	Akut monoblastær, AML M5a
DC930B	Akut monoblastær, AML M5b
DC931	Leukaemia monocytica chronica
DC931A	Kronisk myelomonocytær leukæmi, CMML-1
DC931B	Kronisk myelomonocytær leukæmi, CMML-2
DC931C	Kronisk myelomonocytær, CMML med eosinofili
DC932	Leukaemia monocytica subacuta
DC933	Juvenil myelomonocytær leukæmi
DC937	Leukaemia monocytica anden form
DC939	Monocytær leukæmi UNS
DC94	Leukæmi, anden spec
DC940	Akut erytroid leukæmi, M6 (a)(b)
DC940A	Erytroleukæmi UNS
DC941	Erythroleukaemia chronica
DC942	Leukaemia megakaryoblastica acuta
DC943	Mastcelle leukæmi
DC943A	Mastcelleleukæmi
DC944	Panmyelosis acuta
DC945	Myelofibrosis acuta
DC946	Uklassificerbar myelodysplasi/myeloproliferativ sygdom
DC947	Leukaemia anden spec form
DC947A	Leukaemia eosinophilica
DC947B	Aggressiv NK-celle leukæmi
DC947C	Akut basofil leukæmi
DC95	Leukæmi uden specifikation
DC950	Leukaemia acuta uden specifikation
DC950A	Blastcelleleukæmi
DC950B	Leukaemia acuta, stamcelleleukæmi
DC950C	Akut bilineær leukæmi
DC950D	Akut leukæmi af blandet linearitet
DC951	Leukaemia chronica uden specifikation
DC952	Leukaemia subacuta uden specifikation
DC957	Leukaemia anden form uden specifikation
DC959	Leukaemia uden specifikation

Myeloma

DC90	Maligne plasmacelle neoplasier
DC900	Myelomatose
DC901	Plasmacelle leukæmi
DC902	Plasmacytoma extramedullare
DC902A	Myeloma solitarium
DC903	Solitært ossøst plasmacytom

Stem cell transplantation

DT860	Afstødning af knoglemarvstransplantat
DT860A	Graft-versus-host reaction
DZ948C	Knoglemarvstransplanteret
DZ948C1	Tilstand med transplanteret knoglemarv, allogen
DZ948C2	Knoglemarvstransplanteret med autolog knoglemarv
DZ948	If not DZ948A, DZ948B, DZ948C and if coded as a B-diagnosis or additional diagnosis together with one of the following A-diagnoses C770, C81–C96, D45–D47, D50–D85, D87–D89, T86.0, T86.0A, or T88.8N

Solid organ transplantation

DT862	Svigt eller afstødning af transplanteret hjerte
DT862A	Svigt af hjertetransplantat
DT862B	Afstødning af hjertetransplantat
DT863	Svigt eller afstødning af hjerte-lungetransplantat
DT863A	Svigt af hjerte-lungetransplantat
DT863B	Afstødning af hjerte-lungetransplantat
DT864	Svigt eller afstødning af transplanteret lever
DT864A	Svigt af levertransplantat
DT864B	Afstødning af levertransplantat
DT868B	Svigt af transplanteret tyndtarm
DT868D	Svigt af transplanteret pankreas
DT868E	Svigt af transplanteret lunge
DT868G	Afstødning af transplanteret tyndtarm
DT868I	Afstødning af transplanteret pankreas
DT868J	Afstødning af transplanteret lunge
DZ941	Hjertetransplanteret
DZ942	Lungetransplanteret
DZ943	Hjerte-lungetransplanteret
DZ944	Levertransplanteret
DZ948A	Pancreastransplanteret
DZ948B	Tyndtarmstransplanteret

Other immunosuppressive disease

DD611	Aplastisk anæmi, medicininduceret
DD612	Aplastisk anæmi forårsaget af anden ydre påvirkning
DD613	Idiopatisk aplastisk anæmi
DD618	Anden aplastisk anæmi
DD619	Aplastisk anæmi UNS
DD619A	Pancytopeni UNS
DD619B	Hypoplastisk anæmi UNS
DD619C	Medullær hypoplasi
DD619D	Panmyelophthisis
DD81	Kombinerede immundefekter
DD810	Svær kombineret immundefekt (SCID) med retikulær dysgenesi
DD811	Svær kombineret immundefekt (SCID), lavt antal T-/B-celler
DD812	Svær kombineret immundefekt (SCID), lavt/norm antal B-celler
DD813	Adenosindeaminase (ADA) mangel

DD814	Nezelofs syndrom
DD815	Purinnukleosidfosforylase (PNP) mangel
DD816	Histokompatibilitetskompleksmangel, klasse I
DD817	Histokompatibilitetskompleksmangel, klasse II
DD818	Anden kombineret immundefekt
DD819	Kombineret immundefekt UNS
DD819A	Svær kombineret immundefekt (SCID) UNS
DD820	Wiskott-Aldrichs syndrom
DD821	Di Georges syndrom
DD821A	Aplasia thymi med immundefekt
DD822	Immundefekt med korte ekstremiteter
DD83	Almindelige variable immundefekter
DD830	Immundefekt med overvejende abnormt B-celletal og -funktion
DD831	Immundefekt med overvejende forstyrrelse i T-celler
DD832	Immundefekt med autoantistoffer mod B- eller T-celler
DD838	Anden almindelig variabel immundefekt
DD839	Almindelig variabel immundefekt UNS

Table 2. International classification of diseases (ICD) version 8 codes

Depression

296,09	Melancholia involutionis
296.29	Psychosis manio-depressiva, typus depressivus
296.99	Psychosis manio-depressiva
298.09	Psychosis reactiva depressiva
300.49	Neurosis depressiva
295.79	Schizophrenia, typus schizo-affectivus
300.19	Neurosis hysterica

Anxiety

300.09	Neurosis anxiosa
300.29	Neurosis phobica

Rheumatoid arthritis

712.09	Arthritis rheumatoides juvenilis (Morbus Still)
712.19	Arthritis rheumatoides cum splenomegalia et leukopenia (Syndroma Felty)
712.29	Arthritis palindromica
712.39	Arthritis rheumatoides alia et non specificata
712.59	Fibrositis rheumatoides chronica nodularis

Lupus erythematosus

734.19	Lupus erythematosus disseminatus

Inflammatory bowel disease

563.01	Enteritis regionalis granulomatosa (Crohn)
563.19	Colitis ulcerosa, proctocolitis haemorrhagica
569.04	Proctitis haemorrhagica

Chronic obstructive pulmonary disease

491	Bronchitis chronica
491.00	Bronchitis chronica non purulenta
491.01	Bronchitis chronica purulenta
491.02	Bronchitis chronica cum emphysemate
491.03	Bronchitis chronica asthmatica
491.04	Tracheobronchitis chronica
491.08	Bronchitis chronica alia definita
491.09	Bronchitis chronica
492	Emphyaema pulmonum
492.00	Emphysema pulmonum generalisatum
492.01	Emphysema pulmonum localisatum (bullosum)
492.08	Emphysema pulmonum aliud definitum
492.09	Emphysema pulmonum

Asthma

493	Asthma

493.00	Asthma bronchiale
493.01	Status asthmaticus
493.02	Asthma bronchiale cum rhinitide allergica
493.08	Asthma aliud definitum
493.09	Asthma

Chronic kidney disease

584	Sclerosis renalis
792	Uraemia
997.7	Complicatio post transplantationem renis
Y95.09	Transplantatio renis

Diabetes type 1

249	Diabetes mellitus, lnsulino dependente
249.00	Diabetes mellitus, insulino dependente, sine complicatione
249.01	Cataracts, retinopathia diabetics. insulino dependente
249.02	Nepbropathia diabetics, syndroma K.immelstiel-Wilson, insulino dependente
249.03	Neuropathia, polyneuritis wabetica, insulino dependente
249.04	Angiopathia diabetics extremitatum. insulino dependente
249.05	Gangraena diabetics. insulino dependente
249.06	Coma diabeticum sine ketonuria, insulino dependente
249.07	Coma (inclusum praecoma) diabeticum, insulino dependente
249.08	Diabetes mellitus, insulino dependente, cum complicatione alia delinita
249.09	Diabetes mellitus, insulino dependente

Diabetes type II

250	Diabetes mellitus, lnaullno lndependente
250.00	Diabetes mellitus, insulino independente, sine complicatione
250.01	Cataracta, retinopatbia diabetics, insulino independente
250.02	Nepbropathia diabetics, syndroma K.immelstiel-Wi.lson, insulino independente
250.03	Neuropatbia, polyneuritis diabetica, insulino independente
250.04	Angiopathia diabetics ex.tremitatum, insulino independente
250.05	Gangra.ena diabetics, insulino independente
250.06	Coma dlabeticum sine ketonuria, insulino independente
250.07	Coma (inclusum praecoma) diabeticum, insulino indepeodente
250.08	Diabetes mellitus, insulino independente, cum complicatione alia definita
250.09	Diabetes mellitus, insulino independente

HIV

079.83	AIDS (Erhvervet immundefekt syndrom)

Lymphoma

200	Retlculosarcoma et lymphosarcoma
200.09	Reticulosarcoma
200.19	Lymphosarcoma

201	Lymphogranulomatosis maligne Hodgkin
201.99	Lymphogranulomatosis maligna Hodgkin
202	Neoplasmata alia telae lymphatlcae
202.09	Lymphoma giganto-folliculare Brili-Symmer
202.19	Mycosis fungoides
202.29	Neoplasma malignum primarium telae lymphaticae aliae
202.99	Lymphomata alia definita
202.20	Neoplasma malignum lienis primarium
202.90	Neoplasma benignum sive non specificatum medullae osseae

Leukaemia

204	Leukaemia lymphatics
204.09	Leukaemia lymphatica acuta
204.19	Leukaemia lymphatica chronica
204.99	Leukaemia lymphatica
205	Leukaemia myeloldes
205.09	Leukaemia myeloides acuta
205.19	Leukaemia myeloides chronica
205.99	Leukaemia myeloides
206	Leukaemia monocytlca
206.09	Leukaemia monocytica acuta
206.19	Leukaemia monocytica chronica
206.99	Leukaemia monocytica
207	Leukaemia alia alva non specificata
207.09	Leukaemia acuta (stamcelleleukaemi)
207.19	Leukaemia chronica
207.29	Erythroleukaemia (Di Guglielmo)
207.99	Leukaemia non specificata

Myeloma

203	Myelomatosis, myeloma multiplex
203.99	Myelomatosis

Solid organ transplantation

Y95.49	Transplantatio pancreatis
Y95.89	Transplantatio alia definita
997.79	Complicatio transplantationis organi

Other immunosuppressive disease

284.01	Anaemia aplastica acquisita primaria
284.02	Anaemia aplastica acquisita secundaria
284.08	Anaemia aplastica alia definita
284.09	Anaemia aplastica
758.30	Aplasia thymi

Table 3. Anatomical Therapeutical Chemical Classification System codes

Antidepressants

N06AA
N06AB
N06AF
N06AG
N06AX (excluding N06AX05 and N06AX12)
N06CA01
N06CA02
N06CA03

Antidiabetics

A10A
A10B (excluding A10BE01)
B04AX07
C10AX04

Inhaled corticosteroids

R03BA*
R03AK06
R03AK07
R03AK08
R03AK09
R03AK10
R03AK11

Oral corticosteroids

H02AB* (excluding injections)

Other immunosuppressants

L01*
L04*
V02CA01
V02CA02

Table 4. NOMESCO surgical codes

Chronic kidney disease

KJAK10	Laparotomi med indlæggelse af kateter til peritonealdialyse
KJAK11	Laparoskopisk indl. af kateter til peritonealdialyse
KJAK13	Laparotomi m. omlejring af peritonealt dialysekateter
KJAK14	Laparoskopisk omlejring af peritonealt dialysekateter
KTJA30	Perkutan indlæggelse af kateter til peritonealdialyse
KTJA32	Omlejring af peritoneal dialyse kateter (ikke operativt)
KTJA35	Fjernelse af peritonealdialyse kateter
KKAS	Nyretransplantationer og op. i forbindelse hermed
KKAS00	Autolog nyretransplantation
KKAS10	Allogen nyretransplantation m. nyre fra kadaverdonor
KKAS20	Allogen nyretransplantation m. nyre fra levende donor
KKAS40	Excision af transplanteret nyre
KKAS41	Perkut. endoskopisk excision af transplanteret nyre
KKAS50	Pyelocystotomi på transplanteret nyre
KKAS60	Operation for lymfocele v. transplanteret nyre
KKAS61	Perkut. endoskop. op. for lymfocele v. transplanteret nyre
KKAS70	Uretertransposition til transplanteret urinleder/nyrebækken
KKAS96	Anden operation i forbindelse m. nyretransplantation
KKAS97	An. perkut. endoskop. op. i forb. m. nyretransplant.

Solid organ transplanation

KFQ	Transplantationer af hjerte og lunger
KFQA	Transplantationer af hjerte
KFQA00	Ortotop hjertetransplantation
KFQA10	Ortotop hjertetransplantation m. bicaval anastom.
KFQA20	Ortotop hjertetranspl./rekonstr.af forbind. til atr./sys-vv.
KFQA30	Heterotop hjertetransplantation
KFQA40	Hjertetransplantation med levende donor
KFQA96	Anden transplantation af hjerte
KFQB	Transplantationer af hjerte og lunger
KFQB00	Hjerte-lungetransplantation
KFQB10	Hjerte-lungetransplantation m. bicaval anastom.
KFQB20	Hjerte-lungetransplant./rekonstr. af forbind. atr/syst-vv.
KFQB30	Hjerte-lungetransplant./revaskularis. af bronkial-arterier
KFQB96	Anden transplantation af hjerte og lunge
KFQW	Andre op. v. transplantation af hjerte og lunger
KFQW96	Anden procedure v. transplantation af hjerte og lunger
KGDG	Lungetransplantationer
KGDG00	Transplantation af enkelt lunge
KGDG03	Transplant., en lunge m. revaskularisering af bronkial-art.
KGDG10	Transplantation af begge lunger
KGDG13	Enbloc transplant., lunger m.revaskular. af bronkial-art.

KGDG30	Lungetransplantation fra levende donor
KGDG96	Anden lungetransplantation
KGDG99	Lungetransplantation, anden
KJLE	Transplantationer af pancreas og op. i forb. Hermed
KJLE00	Allo-total transplant. af pancreas m. pankreatikocystostomi
KJLE03	Allo-total transplant. af pancreas m. pankreatikoenterostomi
KJLE10	Allogen segmental transplantation af pancreas
KJLE16	Allo-segmental transplant. af pancreas fra levende donor
KJLE20	Allogen ø-celle transplantation
KJLE30	Xenogen ø-celle transplantation
KJLE40	Total excision af transplanteret pancreas
KJLE50	Okklusion af pancreasgang på transplanteret pancreas
KJLE56	Omlægn. af pankreatikocystostomi til pankreatikoenterostomi
KJLE96	An. pancreastranspl. el. op. i forb. Hermed
KJLE99	Pancreastransplant. og dermed relaterede operationer, anden
KJJC	Levertransplantation og op. i forbindelse hermed
KJJC00	Allogen levertransplantation
KJJC10	Allogen partiel levertransplantation
KJJC20	Allogen partiel levertransplantation fra levende donor
KJJC30	Xenogen levertransplantation
KJJC40	Xenogen partiel levertransplantation
KJJC50	Resektion af donorlever i forbindelse m. transplantation
KJJC60	Fjernelse af transplanteret lever
KJJC96	An. levertransplantation el. op. i forb. Hermed
KJJC96A	Bench kirurgi (back-table kirurgi) mhp. Leverimplantation

Table 5. Procedure codes

Chronic kidney disease

BJFD2	Dialyse ved kronisk nyresygdom
BJFD20	Hæmodialyse ved kronisk nyresygdom
BJFD21	Kontinuerlig ambulant peritonealdialyse, CAPD
BJFD22	Intermitterende peritonealdialyse, IPD
BJFD23	Natlig peritonealdialyse, NPD
BJFD24	Kontinuerlig cyklisk peritonealdialyse, CCPD
BJFD25	Daglig ambulant peritonealdialyse, DAPD
BJFD26	Hæmodiafiltration
BJFD27	Automatisk peritonealdialyse, APD
BJFZ	Delprocedurer ved dialysebehandling
BJFZ0	Tilslutning af dialyseapparatur til patient
BJFZ00	Tilslutning af hæmodialyseapparatur til patient
BJFZ01	Tilslutning af peritonealdialyseapparatur til patient
BJFZ1	Fjernelse af dialyseapparatur fra patient
BJFZ10	Fjernelse af hæmodialyseapparatur fra patient
BJFZ11	Fjernelse af peritonealdialyseapparatur fra patient
BJFZ4	Delprocedure vedrørende dialysekateter
BJFZ40	Anlæggelse af hæmodialysekateter
BJFZ40A	Anlæggelse af tunnelleret hæmodialysekateter
BJFZ41	Skift af hæmodialysekateter
BJFZ41A	Skiftning af tunneleret hæmodialysekateter
BJFZ42	Skylning af hæmodialysekateter
BJFZ43	Fjernelse af hæmodialysekateter
BJFZ43A	Fjernelse af tunnelleret hæmodialysekateter
BJFZ44	Omlægning af hæmodialysekateter
BJFZ45	Anlæggelse af peritonaldialysekateter
BJFZ46	Skift af peritonaldialysekateter
BJFZ47	Skylning af peritonaldialysekateter
BJFZ48	Fjernelse af peritonaldialysekateter
BJFZ49	Omlægning af peritonealdialysekateter
BJFZ4A	Tætning af peritoneal dialysekateter uden omlægning
BJFZ5	Procedure vedrørende dialysekateter
BJFZ50	Skift af hæmodialysekateter
BJFZ51	Skylning af hæmodialysekateter
BJFZ52	Skift af peritonaldialysekateter
BJFZ53	Skylning af peritonaldialysekateter
BJFZ6	Slangeskift ved dialysebehandling
BJFZ60	Slangeskift ved peritonealdialysekateter
BJFZ9	Tilpasning af dialyseapparatur til patient
BJFZ90	Programmering af kort til individuel dialysebehandling

BJFZ91	Justering af individuel dialysebehandling
BJKB	Pædagogiske handlinger i relation til dialyse
BJKB0	Oplæring i brug af kronisk peritonealdialyse, CAPD
BJKB1	Oplæring i brug af maskine til peritonealdialyse
BJKB2	Oplæring i brug af maskine til hæmodialyse
BUBA2	Hæmodialysediæt
BUFC1	Diætvejledning ved dialysebehandling
BUFC10	Initial diætvejledning ved dialyse
BUFC11	Opfølgende diætvejledning ved dialyse
BWDC5	Tilsætning af medicin til peritonealdialysevæske
ZZ0151A	Kontrol i forbindelse med hjemmedialysebehandling
ZZ0151A1	Kontrol i forbindelse med hjemmehæmodialysebehandling
ZZ0151A2	Kontrol i forbindelse med hjemmeperitonealdialysebehandling
ZZ4341	Hæmodialyse, kronisk
ZZ4342	Hæmodialyse, kronisk i dialysecenter
ZZ4343	Hæmodialyse, kronisk i hjemmet
ZZ4346	Peritonealdialyse, kronisk
ZZ4347	Peritonealdialyse, kronisk i dialysecenter
ZZ4348	Peritonealdialyse, kronisk i hjemmet
ZZ4350	Haemofiltratio

Stem cell transplantation

BOQE	Behandling med stamcellekoncentrat
BOQE0	Behandling med stamcellekoncentrat fra knoglemarv
BOQE00	Behandling med stamcellekoncentrat fra autolog knoglemarv
BOQE01	Beh.m. oprenset stamcellekoncentrat fra autolog knoglemarv
BOQE02	Beh.m.stamcellekonc.fra aut.knoglemarv,stimul.in vitro m.vækstfakt
BOQE03	Beh.m.oprens.stamcel.konc.fra aut.knoglemarv stim.in vitro m vækstfak
BOQE04	Behandling med stamcellekoncentrat fra allogen knoglemarv
BOQE05	Beh.m. oprenset stamcellekonc fra allogen knoglemarv
BOQE06	Beh.m.stamcellekonc.fra allogen knoglemarv stim.in vitro m.vækstfakt
BOQE07	Beh.m.oprens.stamcellekonc.fra allog.knoglemarv stim.in vitro væ.fakt
BOQE1	Behandling med stamcellekoncentrat fra autolog knoglemarv
BOQE10	Beh.m.uoprenset,ikke-stim.stamcellekonc.fra autolog knoglem.
BOQE11	Beh.m. oprenset stamcellekoncentrat fra autolog knoglemarv
BOQE12	Beh.m.stamcellekonc.fra aut.knoglemarv,stim.in vitro/vækstf.
BOQE13	Beh.m.oprens.stamcel.konc/aut.knoglem. stim.in vitro/vækstf.
BOQE14	Behandling med stamcellekoncentrat fra allogent perifert blod
BOQE15	Beh.m.oprenset stamcellekonc.fra allogent perifert blod
BOQE16	Beh.m.stamcellekonc.fra allo.perif.blod stim.in vitro m vækstfakt
BOQE17	Beh.m.oprens.stamcelkonc.fra allo.perif.blod stim.in vitro vækstfak
BOQE2	Behandling m. stamcellekoncentrat fra autologt perifert blod
BOQE20	Beh.m.uoprenset,ikke-stim.stamcellekonc/autologt perif.blod

BOQE21	Beh. m. oprenset stamcellekonc. fra autologt perifert blod
BOQE22	Beh.m.stamcellekonc./aut.perif.blod stim.in vitro m.vækstfak
BOQE23	Beh.m.oprens.stamcel.konc/aut.per.blod stim.in vitro/vækstf.
BOQE3	Beh.m.stamcellekonc. fra allogen knoglemarv fra familiedonor
BOQE30	Beh.m.uoprens.ikke-stim.stamcel.konc/allo.knoglem/fam.donor
BOQE31	Beh.m. oprens. stamcellekonc/allogen knoglemarv/familiedonor
BOQE32	Beh.m.stamc.konc/allo.knoglem/fam.donor/stim.in vitro/vækstf
BOQE33	Beh.m.oprens.stamc/allo.knoglem/fam.donor/stim.in vitro/v.fa
BOQE4	Beh.m.stamcellekonc./allogent perifert blod fra familiedonor
BOQE40	Beh.m.uoprens,ikke-stim.stamc.konc/allo.perf.blod/fam.donor
BOQE41	Beh.m. oprens. stamcellekonc/allogent perf. blod/fam.donor
BOQE42	Beh.m.stamc.konc/allo.perf.blod/fam.donor/stim.in vitro/v.fa
BOQE43	Beh/oprens.stamc/allo.perf.blod/fam.donor/stim.in vitro/v.fa
BOQE5	Beh. m. stamcellekonc. fra allogen knoglemarv/fremmed donor
BOQE50	Beh.m.uoprens.ikke-stim.stamcel.konc/allo.knoglem/frem.donor
BOQE51	Beh.m. oprens.stamcellekonc/allogen knoglemarv/fremmed donor
BOQE52	Beh.m.stamc.konc/allo.knoglem/fr.donor/stim.in vitro/vækstfa
BOQE53	Beh.m.oprens.stamc/allo.knoglem/fr.donor/stim.in vitro/v.fak
BOQE6	Beh.m.stamcellekonc.fra allogent perifert blod/fremmed donor
BOQE60	Beh.m.uoprens, ikke-stim.stamc.konc/allo.perf.blod/fr.donor
BOQE61	Beh.m. oprens. stamcellekonc/allogent perf. blod/frem.donor
BOQE62	Beh.m.stamc.konc/allo.perf.blod/fr.donor/stim.in vitro/v.fak
BOQE63	Beh/oprens.stamc/allo.perf.blod/fr.donor/stim.in vitro/v.fak
BOQE7	Behandling med stamcellekoncentrat fra placenta og navlesnor
BOQE70	Beh.m.uoprens. stamcellekoncentrat fra placenta og navlesnor
BOQE71	Beh.m. oprens. stamcellekoncentrat fra placenta og navlesnor
BOQF	Knoglemarvstransplantation (KMT)
BOQF0	Autolog knoglemarvstransplantation
BOQF1	Allogen knoglemarvstransplantation
BOQF11	Allogen knoglemarvstransplantation, familiedoner
BOQF12	Allogen knoglemarvstransplantation, matched unrelated donor
BOQF2	Non myeloablativ knoglemarvstransplantation (mini-KMT)
BOQF21	Non myeloablativ KMT (mini-KMT), familiedonor
BOQF22	Non myeloablativ KMT (mini-KMT), matched unrelated donor

Other immunosuppressants

BOHJ*	Antibody or immune-modulating therapy
BWG*	Cytostatic treatment
BWHB*	Biological therapies
	Or any ATC code for L01*, L04*, V02CA01, or V02CA02 used as additional code in the DNPR
