# Supplementary material for: Partner Bereavement and Risk of Herpes Zoster: Results from Two Population-Based Case-Control Studies in Denmark and the United Kingdom
Source: Clin Infect Dis. 2016 Dec 15;64(5):572–9. doi: 10.1093/cid/ciw840 (PMC5850543; doi:10.1093/cid/ciw840)
Supplement: Supplementary_Appendix_1 [file ciw840_suppl_supplementary_appendix_1.doc]

**Supplementary Appendix 1. Additional details about settings and data sources**

**Description of Danish data sources**

Denmark has a population of approximately 5.6 million inhabitants who have unfettered access to education and medical and social service [1]. There is a long tradition for keeping records of residents’ utilization of these services in various nationwide databases. Because data is recorded at the individual-level using the unique civil personal register (CPR) number assigned to all residents at birth or upon immigration, details regarding several aspects of life can be linked accurately to perform epidemiologic studies [1].

The Danish National Patient Registry records data for the Danish hospital sector, including data on all admissions to non-psychiatric hospitals with nationwide coverage since 1978, admissions to psychiatric wards since 1994, and visits to all outpatient hospital-based specialty clinics and emergency rooms since 1994 [2]. Admissions to psychiatric wards between 1970 and 1994 are available in the Danish Psychiatric Central Registry, which was merged with the Danish National Patient Registry in 1995 [3]. These hospital registries include data pertaining to the patient (eg*,* the CPR number), dates of admission and discharge or start and end of outpatient follow-up, the primary diagnosis, any relevant secondary diagnoses, surgical procedures, other treatments (eg*,* cancer treatments and psychotherapy) and examinations [2]. The treating physician is responsible for recording relevant diagnoses and treatments at the time of discharge, outpatient contact or surgery. Diagnoses are classified according to the *International Classification of Diseases*, 8^th^ revision (ICD-8) until the end of 1993 and the 10^th^ revision (ICD-10) thereafter. Surgical procedures are coded according to a Danish classification (1977 through 1995) and a Danish version of the Nordic Medico-Statistical Committee (NOMESCO) Classification of Surgical Procedures (from 1996 on).

The Danish National Prescription Registry provides information on prescription drugs since 1995 [4]. Each time a prescription is filled at the pharmacy, the patient’s CPR number, the date, the number of packets/units and the Nordic article number of the dispensed product is recorded and transferred electronically to the registry [4]. The Nordic article number encodes the name, Anatomical Therapeutic Chemical code, the number of tablets or units, numerical strength per tablet or unit and formulation of the drug. Indications and instructions for use are recorded, albeit incompletely, since 2004. In the present study, case selection commenced on January 1, 1997 and continued until December 31, 2013. This ensured that all study participants had at least two years of prescription history without any antiviral prescriptions, thus reducing the chance of including treatment for reactivating herpes simplex infections.

The Danish National Diabetes Registry combines data from several nationwide registries to identify patients receiving care for diabetes since 1995 [5]. The registry population includes individuals with a diagnosis of diabetes in the Danish National Patient Registry; registration of chiropody (reimbursable for diabetics), ≥5 blood-glucose measurements in a 1-year period, or ≥2 yearly blood-glucose measurements in five consecutive years in the National Health Service Register; or ≥2 prescriptions for any oral anti-diabetic drug or ≥2 prescriptions for insulin in the Danish National Prescription Registry [5].

The Civil Registration System includes data on age, sex, address and vital statistics for the entire Danish population since 1968 [1]. The registry also contains data on civil status (married, divorced, widow or widower, registered partnership, or dissolved registered partnership), CPR number of spouse or registered partner, and CPR numbers of children. As the Civil Registration System includes data on all residents, it facilitated selection of population controls for the present case-control study.

We linked all registries on the secure servers at Statistics Denmark — the central authority on Danish statistics, which collects, processes, and publishes information relating to the Danish society. Statistics Denmark also collects data from Danish education registries, such as the Population Education Registry [6], which we also included in the present study.

**Description of British data sources**

The UK has a population of approximately 61 million inhabitants. As in Denmark, tax-supported medical care is provided free at the point of delivery. However, while Denmark has a long tradition of recording especially hospital contacts, the UK has well established primary care databases

[7,8]. One of these databases is the Clinical Practice Research Datalink (CPRD), which was used in the current study

[8]. The CPRD was established as a smaller dataset in London in 1987 and was expanded to become the General Practice Research Database in 1993 and finally the CPRD in 2012

[8]. It currently holds data on over 11 million patients from almost 700 practices across the UK. Approximately 4.4 million patients (7% of the UK population) are considered to be active (alive and currently registered) and to have data that meet quality standards put forth by the database. These standards include primarily checks for non-missing data for core variables such as registration dates, birth year, and sex. Participating practices provide electronic health record data to the CPRD through secure servers on a monthly basis. Data collected include various symptoms and diagnoses, tests, health-related behaviours (e.g.*,* smoking status) and anthropometric data, written prescriptions, immunizations and referrals to secondary care. General practice staff are responsible for recording data at the time of patient encounter, using mainly Read codes. The Read code system is a clinical classification system used in general practice in the UK and includes over 96 000 codes hierarchically grouped. Read codes are also converted to medical codes by CPRD. Written prescriptions are coded using the Multilex Product Dictionary, which is translated into product codes.

Sixty percent of practices in the CPRD participate in a linkage scheme, which enables linkage of the primary healthcare records to other data sources, such as the Hospital Episode Statistics database and the Index of Multiple Deprivation

[8]. The Hospital Episode Statistics Database was established in 1987 and includes hospitalization data for inpatients treated at National Health Service hospitals in the UK [9]. Linked data are available since 1997. Primary and secondary diagnoses for the admission are coded using the ICD-10 system and the OPCS (Office of Population and Censuses and Surveys) Classification of Interventions and Procedures version 4 is used to record operations, procedures and interventions. Coding based on hand-written clinical notes is typically outsourced to clinical coders hired by the NHS. Reliable data on contacts to outpatient hospital clinics are not available.

The Index of Multiple Deprivation records data on socioeconomic status at the practice and patient level using quintiles of the Index of Multiple Deprivation Score [10]. This index measures the level of deprivation in small geographical areas (with around 1 500 inhabitants) called the lower layer super output areas. The patient level score is assigned by mapping the home postcode to these areas. The deprivation score is computed by weighing 38 separate indicators within seven domains of deprivation, such as income, employment, crime and living environment. Deprivation increases with increasing score. In the current study, we used the Indices of Deprivation version 2010. Practice level scores were controlled for indirectly through matching by practice and are available for all participating practices in the 2010 dataset.

**References**

1. Schmidt M, Pedersen L, Sørensen HT. The Danish Civil Registration System as a tool in epidemiology. Eur J Epidemiol **2014**; 29:541–549.

2. Schmidt M, Schmidt SAJ, Sandegaard JL, Ehrenstein V, Pedersen L, Sørensen HT. The Danish National Patient Registry: A review of content, data quality, and research potential. Clin Epidemiol **2015**; 7:449–490.

3. Mors O, Perto GP, Mortensen PB. The Danish Psychiatric Central Research Register. Scand J Public Health **2011**; 39:54–57.

4. Pottegård A, Schmidt SAJ, Wallach-Kildemoes H, Sørensen HT, Hallas J, Schmidt M. Data Resource Profile: The Danish National Prescription Registry. Int J Epidemiol **2016**;

5. Carstensen B, Kristensen JK, Marcussen MM, Borch-Johnsen K. The National Diabetes Register. Scand J Public Health **2011**; 39:58–61.

6. Jensen VM, Rasmussen AW. Danish Education Registers. Scand J Public Health **2011**; 39:91–94.

7. Lewis JD, Schinnar R, Bilker WB, Wang X, Strom BL. Validation studies of the health improvement network (THIN) database for pharmacoepidemiology research. Pharmacoepidemiol Drug Saf **2007**; 16:393–401.

8. Herrett E, Gallagher AM, Bhaskaran K, et al. Data Resource Profile: Clinical Practice Research Datalink (CPRD). Int J Epidemiol **2015**; 44:827–836.

9. Hospital Episode Statistics. Available at: http://www.hscic.gov.uk/hes. Accessed 11 May 2016.

10. The English Indices of Deprivation 2010. 2011. Available at: https://www.gov.uk/government/uploads/system/uploads/attachment_data/file/6871/1871208.pdf. Accessed 11 May 2016.
